# Identification of Genes in a Partially Resistant Genotype of *Avena sativa* Expressed in Response to *Puccinia coronata* Infection

**DOI:** 10.3389/fpls.2016.00731

**Published:** 2016-05-31

**Authors:** Yolanda Loarce, Elisa Navas, Carlos Paniagua, Araceli Fominaya, José L. Manjón, Esther Ferrer

**Affiliations:** ^1^Department of Biomedicine and Biotechnology, University of AlcaláAlcalá de Henares, Spain; ^2^Department of Life Sciences, University of AlcaláAlcalá de Henares, Spain

**Keywords:** plant-pathogen interaction, partial resistance, transcriptome, *Avena sativa*, oat, crown rust

## Abstract

Cultivated oat (*Avena sativa*), an important crop in many countries, can suffer significant losses through infection by the fungus *Puccinia coronata*, the causal agent of crown rust disease. Understanding the molecular basis of existing partial resistance to this disease might provide targets of interest for crop improvement programs. A suppressive subtractive hybridization (SSH) library was constructed using cDNA from the partially resistant oat genotype MN841801-1 after inoculation with the pathogen. A total of 929 genes returned a BLASTx hit and were annotated under different GO terms, including 139 genes previously described as participants in mechanisms related to the defense response and signal transduction. Among these were genes involved in pathogen recognition, cell-wall modification, oxidative burst/ROS scavenging, and abscisic acid biosynthesis, as well genes related to inducible defense responses mediated by salicylic and jasmonic acid (although none of which had been previously reported involved in strong responses). These findings support the hypothesis that basal defense mechanisms are the main systems operating in oat partial resistance to *P. coronata*. When the expression profiles of 20 selected genes were examined at different times following inoculation with the pathogen, the partially resistant genotype was much quicker in mounting a response than a susceptible genotype. Additionally, a number of genes not previously described in oat transcriptomes were identified in this work, increasing our molecular knowledge of this crop.

## Introduction

Crown rust, caused by the obligate fungal biotroph *Puccinia coronata* Corda. f. sp. *avenae* Eriks, is a widespread destructive disease of cultivated oat (*Avena sativa* L.) and infections may cause considerable losses in yield and important reductions in grain quality (Gnanesh et al., 2015). More than 96 major genes have been identified in oat that confer complete, gene-for-gene interaction-based, race-specific resistance and many have been used in oat improvement programs (Carson, 2011). However, many *P. coronata* populations have adapted to these resistance genes, resulting in the emergence of new, virulent races of the pathogen. These once-resistant cultivars have thus become susceptible to the disease (McCallum et al., 2007).

Partial resistance (PR) is a type of incomplete resistance, frequently under polygenic control. In oat it is expressed only in adult plants but is believed to have broad-spectrum effectiveness against all physiological races of *P. coronata*. In other species this form of resistance has been selected and used to provide a layer of resistance in the absence of monogenic-mediated resistance (Poland et al., 2008). PR is commonly manifested by a reduction in pathogen multiplication and symptom severity. However, it allows the pathogen to survive, presumably slowing evolution toward full virulence (Portyanko et al., 2005). Indeed, PR can provide protection even when cultivars are grown for prolonged periods in environments favorable to the spread of crown rust (Leonard, 2002).

In oat, PR to *P. coronata* is a quantitative trait and is inherited in a polygenic manner. Over 25 quantitative trait loci (QTLs) for crown rust from 11 different segregating populations have been mapped in oat chromosomes (Zhu and Kaeppler, 2003; Portyanko et al., 2005; Barbosa et al., 2006; Jackson et al., 2007; Acevedo et al., 2010; Lin et al., 2014; Babiker et al., 2015). However, the lack of oat genetic maps with a high density of markers has meant that none closely linked to these QTLs could be identified until recently (Lin et al., 2014; Babiker et al., 2015) when a single nucleotide (SNP) platform became available (Oliver et al., 2013). To date, no race-specific crown rust resistance gene (*R* gene) has been cloned, nor have any candidate genes been identified as underlying the QTLs for PR.

Extensive research into host resistance in numerous pathosystems has revealed many components of the complex molecular mechanisms that operate in active plant defense. As reviewed by Jones and Dangl (2006), the primary immune mechanism used by plants, known as PAMP-triggered immunity (PTI), is induced by general elicitors or PAMPs (pathogen-associated-molecular-patterns). PAMPs are highly conserved molecules that are indispensable to the pathogen. PTI, which frequently involves ion fluxes, the production of reactive oxygen species (ROS), protein phosphorylation and callose deposition soon after PAMPs are recognized at the cell surface by pattern recognition receptors (PRR), limits the growth of non-host pathogens as well as the disease caused by adapted pathogens (Douchkov et al., 2014). However, resistance can be overcome by adapted pathogens capable of delivering virulence-enhancing effector molecules into the host. When these latter effectors are recognized by plant disease resistance proteins encoded by *R* genes, the second layer of plant innate immunity is activated. This effector-triggered immunity (ETI) is a rapid and robust reaction, often associated with a hypersensitive response (HR) leading to cell death at the site of infection. Any lack of efficiency in activating the described defense mechanisms leads to host susceptibility.

Few studies have been performed on the molecular mechanisms controlling PR in any plant pathosystem. There are evidences of the involvement of PTI-associated genes in PR. For example, genes for PRR receptors have been isolated and functionally characterized demonstrating an important role in PR (Cao et al., 2011; Dubouzet et al., 2011). Moreover, a combination of large-scale genetic mapping and marker sequencing has revealed candidate genes underlying QTLs for PR to different pathogens in *Arabidopsis* (Häffner et al., 2014). These genes code for proteins involved in PTI, such as receptor-like protein kinases (RLK), and downstream elements of the defense pathway, such as proteins involved in cell wall reinforcement. However, two wheat genes confer PR against stripe and leaf rust respectively have been cloned, and these define new classes of resistance genes different in both structure and function to previously cloned *R* genes (Fu et al., 2009; Krattinger et al., 2009). It therefore remains unclear exactly which genes are involved in PR, or even whether they are the same as those involved in the PTI and ETI responses.

The molecular mechanisms involved in the response of oat to pathogens have been little studied. It is known, however, that pathogenesis-related β-1,3 glucanases and chitinases show no alterations in expression when oat plants are infected with *P. coronata* in compatible interactions, or in non-host interactions (Fink et al., 1990). In addition, in observations of the oxidative stress caused by *P. coronata* infection in oat cultivars with PR, Figueiró et al. (2015) reported ROS accumulation to be reduced compared to that seen in susceptible genotypes. Further, an increase was seen in the content of cell wall phenolic compounds that eventually caused the death of the pathogen and indeed the plant cells, thus limiting disease progression.

One way to gain insight into oat PR to crown rust would be to identify genes that show changes in expression during infection. However, genomic resources for oat are still scarce (Gutierrez-Gonzalez et al., 2013) and no microarrays for gene expression analysis are available. Alternative strategies are therefore required. A relatively simple and commonly used method to isolate condition-specific genes involves the generation of suppression subtractive hybridization libraries (SSH) (Diatchenko et al., 1996). This method has been successful in isolating plant genes specifically expressed in response to non-host interactions (Neu et al., 2003), incompatible interactions (Yan et al., 2009; Yu et al., 2010; Dmochowska-Boguta et al., 2015) and partial compatible interactions (Huang et al., 2013; Al-Taweel et al., 2014) in different pathosystems. SSH reduces the number of housekeeping genes returned compared to conventional library-involving techniques, as well as the genes commonly expressed under both infection and non-infection conditions. As a result, SSH significantly improves the chances of obtaining genes differently expressed under the conditions of interest. The SSH libraries used by the above authors were obtained after cloning subtracted transcripts and sequencing by traditional Sanger methods. In the present work, the aim of which was to identify transcripts specifically regulated during the PR response of oat plants infected with *P. coronata*, attempts were made to obtain a large number of differentially expressed transcripts at low cost by combining the SSH technique with next-generation sequencing. Preliminary testing was also performed to determine whether, during infection, differences in the expression patterns of selected genes existed between PR and susceptible genotypes.

## Materials and methods

### Plant materials, pathogen isolates, and inoculations

The oat lines used in this work were: (1) MN841801-1 (a reselection of MN841801), which shows PR to crown rust that has remained partially resistant for over 35 years in the face of infection by different *P. coronata* races (Leonard, 2002) and (2) Noble-2, a reselection of Noble, a cultivar highly susceptible to crown rust. Both lines were provided by Dr. H. W. Rines of the Dept. of Agronomy and Plant Genetics, University of Minnesota (St. Paul, MN, USA). Seeds were grown in 0.5 L pots filled with peat:sand (3:1) kept in a growth chamber (14 h light at 19°C and 10 h dark at 18°C; 65% humidity). Seedlings were grown for 1 month and a half to the five leaf stage.

A virulent *P. coronata* isolate, MN93B236, was provided by G. E. Ochocki of the Cereal Disease Laboratory, Agricultural Research Service, US Department of Agriculture (St. Paul, MN, USA). Inoculations were performed by gently applying about 10,000 fresh urediniospores per cm^2^, deposited on a smooth adhesive tape, to 12 cm^2^ of leaf surface. The tape was kept in contact with the leaves for 12 h. Mock-inoculated plants were treated in the same way but without spores. Under these conditions, spore germination does not require higher humidity or greater darkness (Manjón, unpublished). Inoculated and mock-inoculated leaf segments were harvested at 24, 48, and 72 h post-inoculation (hpi), quickly frozen in liquid nitrogen, and stored at −80°C. A batch of inoculated and control plants were grown for checking symptoms and the control of inoculation success at 12 days after inoculation. All experiments were performed in triplicate.

### Generation of a subtracted library

Total RNA was extracted from harvested leaves using the TriPure Isolation Reagent (Roche Applied Sciences, Basel, Switzerland) according to the manufacturer's instructions. It was then quantified using a Thermo Scientific NanoDrop ND-1000 spectrophotometer (NanoDrop Technologies, Wilmington, DE, USA). Total RNA was assessed by formamide denaturing gel electrophoresis, and mRNA isolated from the total RNA using Dynabeads Oligo (dT)_25_ isolation beads (Invitrogen, Thermo Fisher Scientific, Whaltman, MA, USA). An SSH cDNA library was constructed using the PCR-Select^TM^ cDNA Subtraction Kit (Clontech, Mountain View, CA, USA). Equal amounts of total RNA sampled from inoculated plants were pooled. Two micrograms of this mRNA pool was used for cDNA synthesis. cDNAs were also derived from mock-inoculated leaves harvested at the same time points. Subtractive hybridization was performed using sample cDNA (tester) from the inoculated plants, which was subtracted with cDNA (driver) from the non-inoculated plants. This forward subtraction identifies genes induced (upregulated) during the infection process.

### Sequencing and bioinformatics

The cDNA pool resulting from the subtractive library was sequenced according to the Roche 454 GS FLX+ protocol (Roche 454 Life Sciences, Brandford, CT, USA), by Lifesequencing SL (Valencia, Spain). For *de novo* assembly, the sequencing reads were assembled using Newbler 2.6 software (Roche 454 Life Sciences) using the cDNA mode option, a minimum overlap of 40 bases, and 95% similarity, with the remaining computational parameters set to the default options. In order to minimize redundancy after the BLASTx analysis (see below) the final dataset of isotigs (putative distinct transcripts) and singletons (single reads not included in the isotigs after assembly) was subjected to further assembly using a CodonCode Aligner 5.1 (CodonCode Corporation, Centerville, MA, USA) employing a relaxed similarity parameter (>85%) and a minimum overlap length of 30 nt. Sequences that resulted redundant were kept as individual sequences but clustered into groups, named isogroups. To use a common terminology, those sequences that remained as unique sequences after testing the redundancy were also termed isogroups.

BLAST2GO software (Conesa et al., 2005) was used for the automatic annotation of the sequences during BLASTx searches (*e*-value cutoff of 1e-5) in protein databases, including the NR (non-redundant, NCBI) and Swissprot databases. Gene Ontology (GO) mapping was then performed and the sequences functionally categorized according to Biological Process (level 2), Molecular Function (level 3), and Cellular Compartment (level 2). GO enrichment analysis was performed using Fisher's exact test as performed by Bast2GO software.

### Quantitative RT-PCR

Relative quantification of gene expression was performed for selected isogroups. RNA was extracted as described above. cDNA was synthesized from 1 μg of total RNA using the Transcription First Strand cDNA Synthesis Kit (Roche Applied Sciences). Sense and antisense primers were designed using Vector NTI software (Thermo Fisher Scientific). Alignments between each selected isogroup and the most similar gene from *Brachypodium distachyon* were performed by BLASTn analysis and using the NCBI Refseq_genomic database. Table 1 shows all the primers used. Assuming the intron-exon genic structures to be conserved between *Avena* and *Brachypodium*—which is quite feasible given their close relationship (Gutierrez-Gonzalez et al., 2013)—one of these primers was designed to include segments of two consecutive *Brachypodium* putative exons. Thus, genomic DNA carrying introns would not be a target for this primer and, therefore, even minor contaminations of genomic DNA would not mask the cDNA amplification.

**Table 1:** **Primers used in RT-qPCR for validation of transcripts obtained in the SSH library and for comparing expression patterns in partially resistant and susceptible genotypes**.

**Functional category**	**Isogroup**	**Gene description**	**Primer sequences forward/reverse**
Signal perception and transduction	00243^*^	LRR-PK	5′-TCGATTTCGAAAACCTTGGAGCA-3′/5′-TGGCTATACTGCAACAGCAACGAAC-3
	00265	LRR-serine-threonine PK	5′-CAGCCATACAGCCTGACAAG-3′/5′-CAGACAAGGGAACCGTGAAT-3′
	00320^*^	*Xa21*-like	5′-CCAGGCCCTTGAAATCAATACTGG-3′/5′-TGTCGCCGTGAAGGTGCTCA-3′
Ca^2+^mediated signaling	00152	Ca^2+^-dependent PK	5′-TGCTCGCTAACAAGGACGACGA-3′/5′-TGGTCCGTATTTCTTGCAGAGCAC
	01007^*^	Calmodulin-binding protein	5′-CCAAAGATGGTATTCTTAAGGCAATATGG-3′/5′ACTTCAGTAGGCTTCTCATCTTGTTCGAT-3′
Hormone biosynthesis, signaling, and response	00269^*^	Lipoxigenase	5′-GGTCTGAACTCAGCCGGAAGGA-3′/5′-TTGGCAGAAGGCGAGATGGC-3′
	00355	Cysteine-rich receptor-PK	5′-TTCCTGGCGATGAAGAAATAGCAGT-3′/5′-GTTCTTCCAAGCAAACACCGACAA-3′
	00902^*^	4-Coumarate CoA ligase	5′-TACTGAATTCACAATAAGGCAAGGTTGG-3′/5′-CTTCAGCATCAGGAAACGGGATAAC-3′
	00003	Plasma membrane H^+^ ATPase	5′-GAGAAGAGCTGAGATTGCAAGGCTG-3′/5′-TGTCCAATGTAGGAATTTCCTCACACTG-3′
Response to oxidative stress	00430	Gluthatione-S-transferase	5′-AGTTCAGTCTGGTGGACATTGCATATG-3′/5′-TCATCCTTGGTCACACCTTCACTCA-3′
	00565	Gluthatione peroxidase	5′-TCTGTAGCTTACTTCCTTGCAAGAGATACAA-3′/5′-TGATTGCATGGGAACGCCAA-3′
	00011	NADPH cytochrome p450 reductase	5′-TTTAGGGGCTTCTTGCAGGAAAGA-3′/5′-CTTCATATATGTAGTCCATTTCACGGTTCC-3′
Cell wall biosynthesis and modification	00480	Cinnamoyl-CoA-reductase	5′-TACTCCTCTGTGCAACCTTCAAGTAACC-3′/5′-TTGGATGTGGTGGCGGTCAA-3′
	00791	Phenylalanine ammonia lyase	5′- CTTGGTGATGGCCTCGAGGATC-3′/5′-GTGGAGCTCCTCAGGCATCTGAA-3′
Primary metabolism	00169	Anthranilate synthase	5′-ATGCACATCAGCTCAACGGTCAGT-3′/5′-CACAATGGTGCGGAGAGCAAGA-3′
	00001	Phosphoribulokinase	5′- ATTTGCATGGAAAATTCAGAGGGACA-3′/5′-CTTCTGCGGATCAATAAATGCATCA-3′
Photorespiration	00002	Victorin-binding protein	5′-AAGGACAACCTGTCTGCTCTGATGGT-3′/5′-GGCTTGTCAACCCAACCTGAGC-3′
	00232^*^	Serine hydroxymethyltransferase	5′-CTGCTTGTTACAGATCTTGCGCATG-3′/5′-GGTGGACATCTTTCTCATGGTTACCA-3′
Protein metabolism	00192	Heat shock protein	5′-CAGATAGGCTTCTGCTTGTTGACCAA-3′/5′-CCGCATCAAGGATCTGATCAAGAA-3′
Transport	00218^*^	ABC transporter G family member	5′-TCTGTAGCTTACTTCCTTGCAAGAGATACAA-3′/5′-ACCACAGGTAGCAATGCAGAACACA-3′

* Isogroups in the novel file.

qPCR was performed in a 96-well thermal cycler 7500 Fast Real Time PCR System (Thermo Fisher Scientific). Each sample was run in duplicate along with two non-template controls containing either water or RNA instead of cDNA. Inter-run calibrations were performed to correct for plate-to-plate variation. Primer efficiencies were calculated from the linear regression of the log-transformed expression values obtained from a series of four, 1/4-fold dilutions of cDNA. PCR was performed using SYBR Green Master Mix (Roche Applied Sciences) in reaction volumes of 10 μl containing FastStart DNA polymerase, 10 ng of cDNA, and the gene-specific primers (2.5 μM each). The PCR conditions were 10 min of enzyme activation and cDNA denaturation at 95°C, followed by 40 cycles of 15 s at 95°C and 1 min at 60°C. Immediately after the final PCR cycle, melting curve analysis was performed to determine the specificity of the reaction. The number of amplification steps required to reach the threshold cycle number (Ct) was calculated and the relative expression level for each gene in different templates calculated using the qBase Plus method (Hellemans et al., 2007) employing two reference genes for normalization. These genes—coding for *MDH* (malate dehydrogenase) and *CDC48* (CDC48 ATP-ase)—were chosen for their previously assessed stability in these same samples (Paniagua et al., to be published elsewhere). The mean relative expressions of the *P. coronata*-inoculated and mock-inoculated samples (two repetitions) at each time point were compared. Relative expression was deemed significant when there was a minimum two-fold change between the *P. coronata*- and mock-inoculated samples.

## Results

### Identification of genes differently expressed in response to *P. coronata* inoculation

#### Sequence analysis

SSH was used to identify the oat genes induced by *P. coronata*. cDNA from inoculated oat leaves was used as the tester, and cDNA from non-inoculated control oat leaves as the driver. The resulting cDNA library, with an average sequence length of 949 nt, was subjected to 454-FLX pyrosequencing. In total, 155,411 reads were obtained with an average length of 542 nt. These have been deposited in the NCBI Sequence Read Archive under the accession number SRX1719783. 90.75% of the reads were of sufficient quality to be used in the ensuing assembly process to give a set of unique ESTs (performed using Newbler 2.6 software). This initially provided 745 isotigs; 10,061 sequences remained unassembled. These unassembled sequences were subjected to selection by length (>150 nt) and differences in similarity (>95%), leading to the detection of 2146 singletons. In total, 2891 sequences were obtained, ranging in length from 152 to 1579 nt. A total 30% of these sequences corresponded to different single entries in the GenBank non-redundant protein database (identified using a BLASTx search). This indicates that duplicates (i.e., >95% similarity for >97% of the sequence length) corresponding to either splice variants, sequences belonging to the different subgenomes of the hexaploid oat (homeologs), or sequences belonging to highly related multigene families (paralogs), clustered separately in the assembly process. Since the lack of an oat reference genome precluded the determination of the causes of redundancy for each sequence, we tried to minimize redundancy by generating clusters that would eventually contain splice variants, paralogous or homeologous sequences. The analysis—using CodonCode Aligner software for clustering the isotigs and singletons with more relaxed parameters than those used above (i.e., >85% similarity and a minimum overlap length of 30 nt)—returned 1057 non-overlapping groups of sequences contemplated as isogroups. In 71% of these, only a single sequence (either an isotig or a singleton) was included, while 8% of the isogroups contained a variable number of similar sequences ranging from 4 to 185. The level of redundancy within each isogroup was low since most of the isogroups with more than one sequence involved only two or three similar sequences (21%). In such cases, the longest sequence was deemed representative. The representative sequence of each of the 1057 isogroups is in Supplementary File 1.

#### Functional annotation of isogroups

The 1057 isogroups were compared with those in the GenBank non-redundant protein database using BLASTx software. 96.2% of the isogroups matched (*e* < 1e-5) with some of the protein sequences in the database. Predicted protein families and functional domains were assigned for 72.19% of the sequences after comparisons involving the InterPro database. With respect to species annotation, the best matches involved sequences from *Brachypodium distachyon (25%), Hordeum vulgare* (18.9%), and *Triticum tauschii* (10.2%). The isogroups with the largest number of reads showed similarity to phosphoribulokinase (isogroup 00001), victorin-binding protein-glycine dehydrogenase (isogroup 00002), plasma membrane ATPase-4 (isogroup 00004), and serine hydroxymethyltransferase (isogroup 00232). The transcripts were extracted from a normalized library, thus precluding the making of accurate deductions regarding the relative expression of the corresponding genes from the reads obtained. However, those isogroups contained sequences resulting from different parts of a putative single transcript, and had similar numbers of reads, indicating their transcripts were abundant in the library prior to PCR amplification.

**Figure 1:**
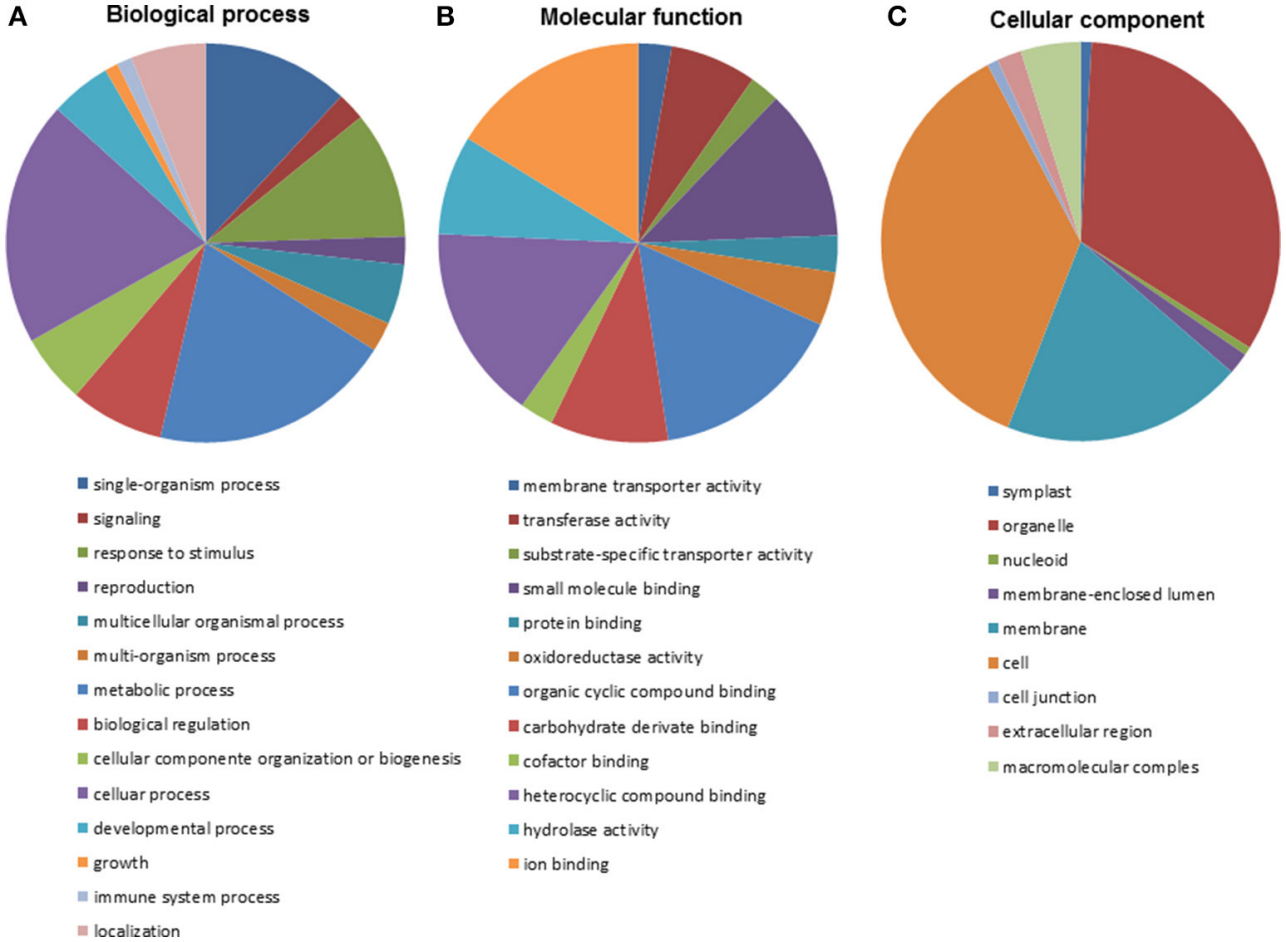
**Annotation of isogroups based on Gene Ontology classification within the categories of Biological Process (A), Molecular Functions (B), and Cellular Components (C)**.

The many positive BLASTx results obtained allowed most (929) of the isogroups to be fully annotated using BLAST2GO software. This allowed additional insights into the putative functions of the corresponding genes (Supplementary File 2). A total 760 (71.9%) isogroups were annotated under all three categories, namely, Biological Process, Molecular Function and Cellular Component. In terms of the Biological Process category, most isogroups were associated with cellular and metabolic processes (Figure 1A; 40% of all annotations). Isogroups involved in energy and primary metabolism were the most abundant within the Biological Process category. Some 20% of the sequences were annotated under the GO term “Response to stimulus” (GO:0050986), and 39 under “Defense response” (GO:000695) and a number of subterms related to resistance. Moreover, some sequences were annotated under terms such as “Multiorganismal process” (5%) and “Signaling” (2%) involved in the articulated response to third party organisms, as well as under, for example, “Signal transduction” (GO:0007165). The most common matches within the category Molecular Function (Figure 1B) involved terms related to molecular binding. Within these terms there were sequences with hydrolase activity (23%), transferase activity (20%) and oxidoreductase activity (12.3%), showing that diverse and intense metabolic activity was underway during the infection process. Moreover, most of the sequences annotated under the term “Molecular transport activities” fell into the subterm of “Transmembrane transporter activity” (GO:0022857). Fourteen ABC transporters of different families were identified, including members of families C and G, which are thought to be involved in detoxification and plant immune reactions. In the Cellular Component category, 34.03% of the isogroups were annotated under the term “Organelle” (GO:0043226) and 24.77% under “Membrane” (GO:0016020; Figure 1C). Within the latter, the two best represented subterms were plasma membrane (GO:005886) and cytoplasmic membrane-bound vesicle (GO:0016023). Activities annotated under these subterms are involved in defense since the cell membrane is a key mediator of communication between plant and pathogen.

#### Oat isogroups homologous to previously described defense-related genes

**Figure 2:**
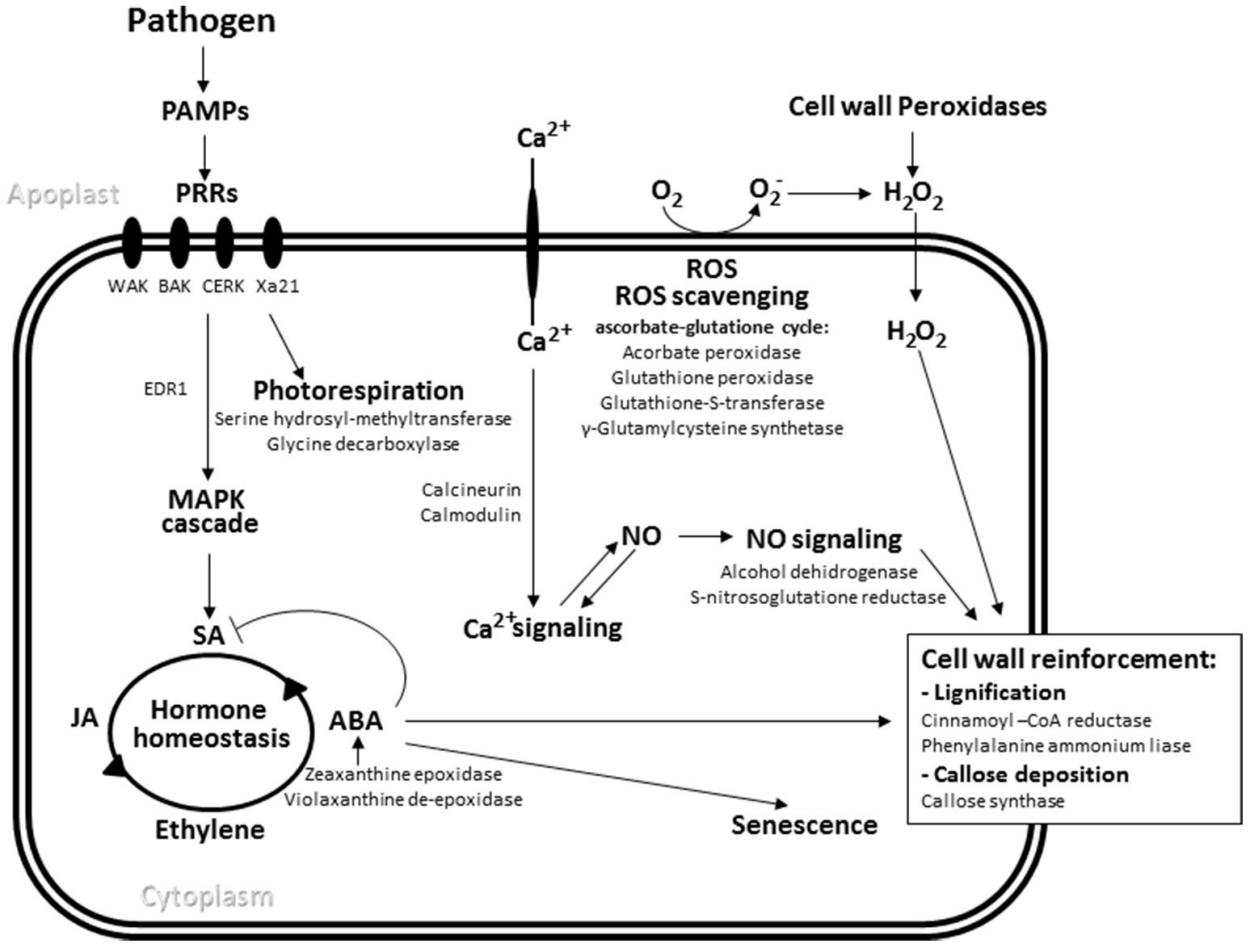
**Pathogen-induced defense response pathways**. The main oat isogroups found in the SSH library with putative roles in these pathways are indicated. PAMPs, pathogen associated molecular patterns, PRR, pattern recognition receptors, MAPK mitogen-activated kinase, ROS, reactive oxygen species, NO, nitric oxide, SA, salicylic acid, JA, jasmonic acid, ABA, abscisic acid.

BLASTx analysis determined that 139 isogroups in the oat SSH library were homologous to genes previously annotated as either being involved in defense pathways or in responses to biotic stimuli in other plants (Figure 2; Supplementary File 3). Among the more interesting were sequences putatively coding for receptor-like kinases involved in PAMP perception (e.g., chitin elicitor receptor kinase, BRI1-associated receptor kinase 1 [BAK1], the broad-spectrum *R* gene of rice *Xa21*, and wall-kinases) and receptors able to recognize more specific effectors very similar to those produced by the *Arabidopsis* resistance genes *RPS2* and *RPP8*. Several of the detected isogroups would appear to participate in signal transduction performed by different protein kinases, with those related to Ca^2+^-mediated signaling pathways, including those involving calmodulin and calcineurin as Ca^2+^ sensors, particularly important. Other sequences found were linked to redox reactions involved in defense-related oxidative bursts and the generation/detoxification of ROS, especially those involved in the biosynthesis and redox status of glutathione. Among the cell wall biosynthesis and modification elements detected, several isogroups coding for enzymes potentially involved in cell wall strengthening (including cellulose synthases, glucanases and, importantly, callose synthase) were encountered. Additionally, genes involved in the first steps of the phenylpropanoid pathway, such as phenylalanine ammonia-lyase (also involved in the biosynthesis of salicylic acid) and cinnamoyl-CoA reductase were also present in the library.

A number of isogroups annotated under the GO term “Defense response” were also annotated under others related to the biosynthesis and signaling of hormones described as important regulators of the defense response, namely salicylic acid, jasmonic acid and ethylene. A group of defense genes related to abscisic acid biosynthesis and signaling was also found, suggesting an involvement of this hormone in PR.

Isogroups associated with primary metabolic pathways were also annotated under the “Defense response” term. These isogroups were identified as involved in energy production, such as carbohydrate metabolism, and the biosynthesis of certain amino acids such as tryptophan—from which phenylpropanoids are derived—and glycine and serine whose synthesis is linked to photorespiration. Several oat isogroups related to this process were sequenced in the SSH library and a gene encoding the mitochondrial enzyme serine hydroxymethyltransferase, which catalyzes the conversion of serine to glycine during photorespiration, and another coding for glycine dehydrogenase, an important enzyme in the photorespiratory cycle, returned the largest number of reads (see Section Functional Annotation of Isogroups). Finally, the importance of protein synthesis and metabolism during the defense response is reflected in the expression of the genes for heat shock proteins HSP70 and HSP90. These have a significant role for folding nascent proteins.

#### Identification of novel genes in *A. sativa*

**Figure 3:**
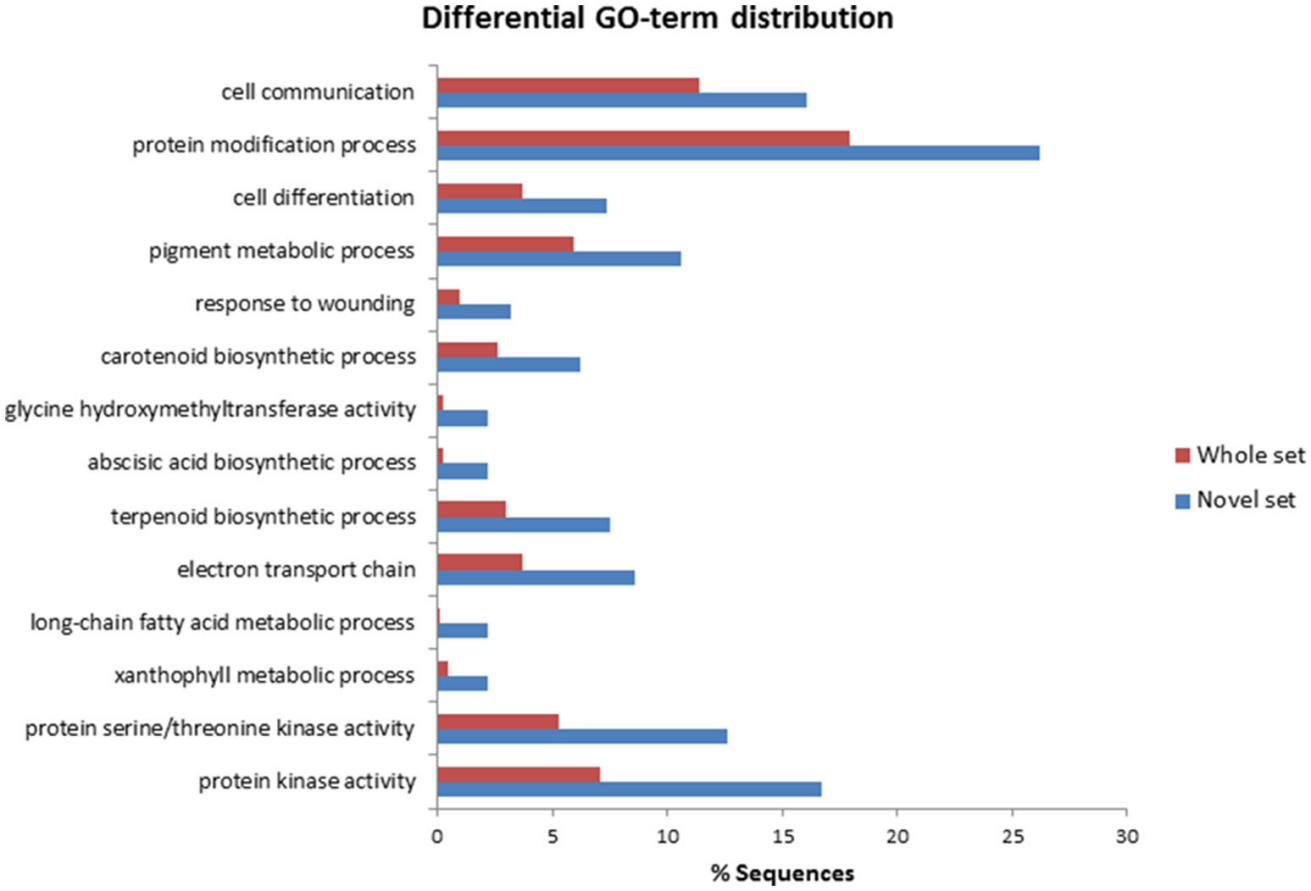
**Enriched Gene Ontology terms in the novel oat set constituted by isogroups showing no similarity to previously described oat sequences**. Data were obtained using the Fisher Exact test (performed using BLAST2GO software).

To identify *A. sativa* genes preferentially expressed during oat-*P. coronata* interactions, the whole set of 929 annotated isogroups were compared by BLASTn analysis with two collections of EST sequences of *A. sativa*. The first used was the *OatSeedRefer90* collection developed by Gutierrez-Gonzalez et al. (2013). This is a compendium of 31,935 non-redundant oat seed sequences in different developing stages that combines information from different sources. The second collection consisted of 7634 sequences deposited in GenBank (as of April 2015). These sequences showed no similarity (*e* < 1e-5) to the first cited collection, they were obtained from tissues other than seeds, mainly leaves and roots. A total of 187 annotated isogroups were not found in the databases explored; this set was termed the “novel set” (see isogroups typed in bold in Supplementary File 1). The distribution of these 187 isogroups within the three main GO categories described above was similar to that obtained for the whole set of sequences. However, a larger proportion of sequences in the novel set were annotated under the GO term “Defense response” (12.8% compared to just 8.9% of the entire set). In addition, comparison of the whole and novel sets using Fisher's enrichment test showed that several GO subterms were significantly over-represented in the novel set. Figure 3 provides a summary of the terms and subterms differently represented in these data sets, e.g., subterms related to the biosynthesis and metabolism of terpenoids and pigments, and sequences involved in the biosynthesis of abscisic acid and long-chain fatty acid metabolism. Within the Molecular Function category, the largest number of isogroups in the novel set was annotated under terms such as “Protein kinase activity” or subterms such as serine/threonine protein kinase activity. This result suggests that a significant part of the pool of protein kinase transcripts obtained by SSH was specifically expressed in leaves in response to the pathogen.

### Expression profiles of differently expressed sequences

**Figure 4:**
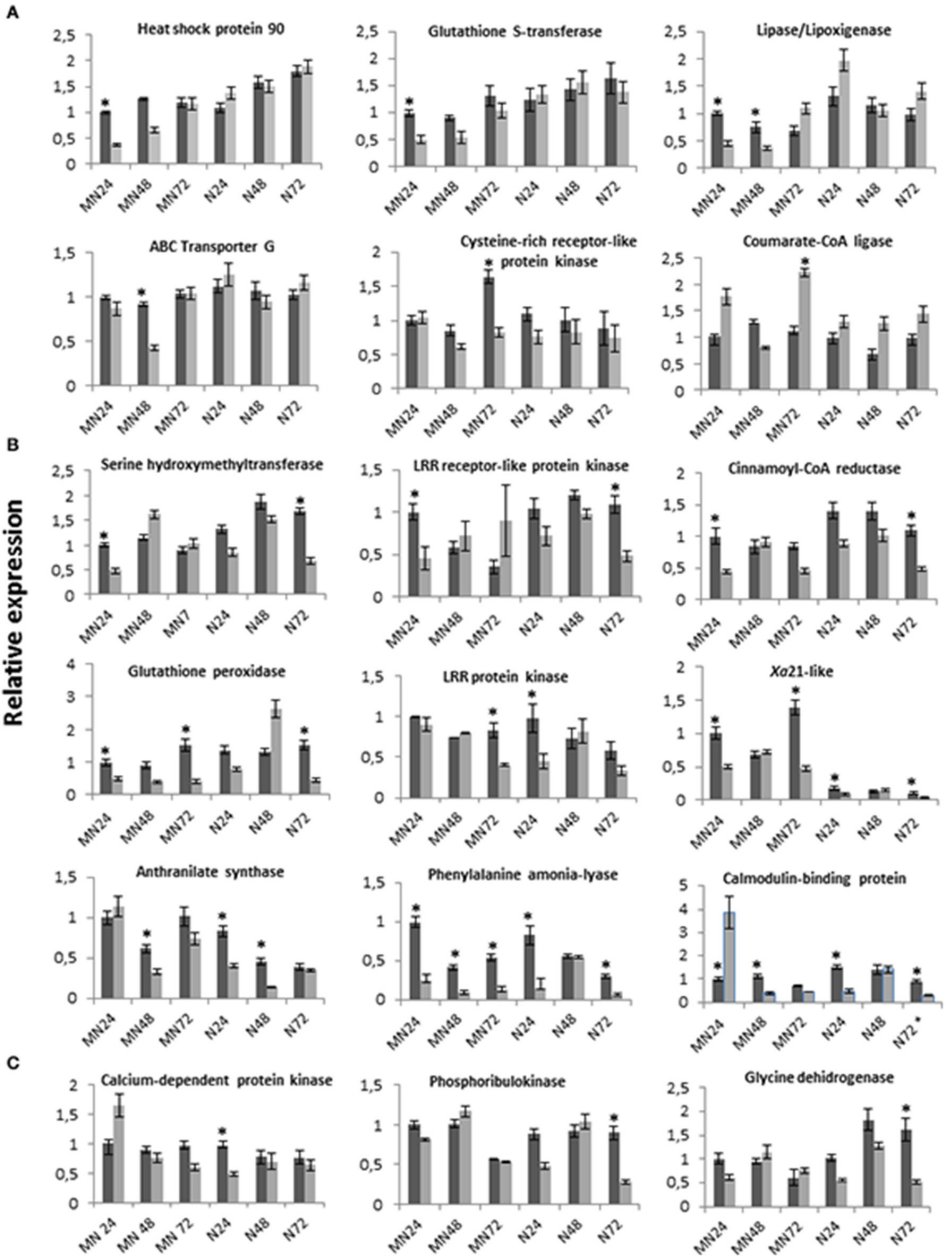
**Time-course expression profiles of 18 selected isogroups in the PR genotype MN841801-1 and the susceptible genotype Noble-2 following inoculation with *P. coronata* (dark boxes), and after mock inoculation (clear boxes)**. The relative expressions of genes are represented as the mean ± SE for the results of two independent RT-qPCR experiments. Within each diagram data are in relation to the expression level of the sample MN841801-1, 24 hpi after inoculation (relative expression = 1). A gene was considered significantly induced or repressed when the ratio between the inoculated and control plants was >2 or < 0.5, respectively. Significant differences are indicated by an asterisk. **(A)** Isogroups upregulated in MN841801-1. **(B)** Isogroups upregulated in MN841801-1 and in Noble-2. **(C)** Isogroups upregulated in Noble-2.

RT-qPCR experiments were performed on 20 isogroups with three purposes: to validate the SSH library, to analyze expression patterns during the fungus-oat interaction at several time points in the PR genotype MN841801-1, and to detect differences in expression patterns between the latter genotype and the susceptible genotype Noble-2. Isogroups were selected from those annotated under highly represented functional GO terms, if they showed similarity to genes involved in plant processes previously demonstrated to be involved in the response to biotic stresses (Table 1). Results obtained for 18 sequences out of the 20 analyzed are shown in Figure 4. To determine whether the genes of the novel set showed characteristic expression profiles in response to infection in comparison with genes of the whole set, seven isogroups belonging to the novel set were included in the analysis. These were annotated under GO terms more strongly represented in the novel set than the whole set.

Expression levels of the selected sequences were examined in inoculated adult leaves of the PR genotype MN841801-1 at 24, 48 and 72 hpi, and compared with expression levels in the leaves of mock-inoculated plants at each time point. Fifteen sequences were differentially expressed between *P. coronata*- and mock-inoculated oat leaves at one or more of the time points (Figure 4). In the PR genotype leaves they were mostly upregulated. Indeed, only one of the sequences, for 4-coumarate-CoA ligase, was downregulated at 72 hpi, while the calmodulin-binding protein was down-regulated a 24 hpi, although expression increased significantly again by 48 hpi. The most conspicuously induced expressions were detected at 24 and 48 hpi, suggesting that genes with putative functions in the partial defense response might be triggered rapidly and play an active role during the early phases of plant-pathogen interactions. Moreover, 9 of the 15 sequences showed induced expression only at one time point indicating that these genes participate in the defense response only at certain points in the infection process.

To examine the changes in gene expression that occur during the PR response to *P. coronata*, the expression patterns of the 20 selected sequences were also evaluated in the susceptible genotype Noble-2. Significant results were obtained for the 18 sequences shown in Figure 4. According to their expression patterns, these sequences belonged to four types. The first (Figure 4A) consisted of six sequences that were significantly upregulated in infected PR plants whereas no differences in expression were seen between *P. coronata-* and mock-inoculated Noble-2 leaves. Most of these were induced at 24 hpi. Only the isogroup for 4-coumarate-CoA ligase was downregulated at 72 hpi. The second expression pattern type (Figure 4B) involved nine sequences that were induced in both genotypes during infection. Within this pattern type, differences between genotypes were seen for most sequences in terms of the time of induction. In general, the induction of expression occurred earlier in the PR than in the susceptible plants and those that showed similar induction times—*Xa*21-like and phenylalanine ammonia-lyase (PAL)—showed higher expression level (*Xa*21-like) or remained upregulated at all time points in the PR genotype (PAL). Only three isogroups—LRR-PK, anthranilate synthase and calmodulin-binding protein—were induced earlier in the susceptible than in the PR genotype. The third pattern (Figure 4C) was seen for three isogroups which were induced only in the susceptible plants. The fourth group was represented by two sequences—NADPH cytochrome P450 reductase and plasma membrane H^+^-ATPase—which showed no expression differences between the pathogen- and mock-inoculated plants in any of the two genotypes (data not shown). On the whole, most of the isogroups analyzed revealed a different expression pattern between the PR and susceptible plants. The PR response involved gene expression changes either of a qualitative nature (i.e., expression compared to no expression or *vice versa*) or depending on the time point. In general, however, gene expression levels induced by the pathogen were only slightly different in the PR and susceptible oat genotypes, even among pathogen- and mock-inoculated plants, suggesting that minor changes in transcription regulation for genes involved in defense might produce different plant responses at the time points examined.

## Discussion

### SSH generation and sequencing by 454-Roche FLX chemistry

The oat genome is polyploid and large, and it has not undergone comprehensive sequence analysis. No oat transcriptome expressed in any defense response including PR has been reported previously. Here, using the SSH method, 1057 unique oat sequences expressed in response to *P. coronata* infection were identified. SSH technology has previously been employed in a wide range of studies, including those on other plant-pathogen interactions (Neu et al., 2003; Yu et al., 2010; Huang et al., 2013; Al-Taweel et al., 2014; Dmochowska-Boguta et al., 2015). In the present work, SSH combined with high-throughput RNA-seq technology using pyrosequencing was shown to be a better alternative than conventional, laborious and time-consuming dye sequencing technology. The sequencing effort made in this work, as revealed by the number of reads obtained, was about one tenth that described in whole transcriptome sequencing studies (see Li et al., 2012; Almeida et al., 2014), and produced at least three times the number of isogroups reported in previous analyses of SSH libraries. For example, in work involving wheat libraries obtained after inoculating with different pathogens, some 200–2250 clones were sequenced by the Sanger method resulting in a number of genes ranging from 126 to 793 (Yu et al., 2010; Huang et al., 2013; Al-Taweel et al., 2014; Dmochowska-Boguta et al., 2015). In the present work, the relatively high proportion of isogroups representing singletons agrees with the purpose of SSH library construction, i.e., to enrich samples in low abundance transcripts that might not easily be obtained in conventional cDNA libraries (Diatchenko et al., 1996). The quality of the sequencing work performed is underscored by the fact that most of the oat ESTs obtained (96.2%) showed significant similarity to sequences in the NCBI protein database (indicating that sequencing errors were few).

The line MN841801-1 developed less than 10% as many uredinia as the susceptible cultivar Noble-2 confirming its previous characterization as a partial resistant genotype (Portyanko et al., 2005; Acevedo et al., 2010). Uredinia density is the best way to differentiate between levels of crown rust resistance in oat cultivars in both field and greenhouse tests (Leonard, 2002). Microscopic analysis of oat cultivars with PR has suggested that this low density might be the outcome of the action of different single mechanism/responses or their combination (Sánchez-Martín et al., 2012). Most of these mechanisms operate before and during the penetration phase of the infection process, which lasts from 12 to 48 h (Kochman and Brown, 1975). Accordingly, sampling time points for SSH library construction were selected to cover transcription during pre-penetration, i.e., during the formation of appressoria (12–24 hpi), and penetration, i.e., during the development of substomatal vesicles and haustoria (48 hpi). Moreover, leaves were also sampled at 72 hpi to obtain transcripts involved in the transduction and exchange of signals directed toward blocking the development of any fungus that had successfully penetrated. At 72 hpi the capture of transcripts governing the late accumulation of phenolic compounds able to prevent fungal colonies developing is likely (Graichen et al., 2011). The identification of 139 defense-related isogroups, and the differences seen in the transcriptional levels of most of the isogroups with respect to the mock-inoculated samples at either 24, 48, or 72 hpi (as analyzed by qPCR; Figure 4), show the selection of the sampling points used to have been appropriate. However, it cannot be ruled out that additional responses occurring at later or earlier point-times were missed.

### Partial resistance is associated with the expression of genes related to pathogen perception and subsequent defense signaling

Inoculation with the isolate MN93B236 of *P. coronata* promoted an active response in the PR cultivar MN841801-1. Transcripts of genes acting in different layers of defense were identified in the present SSH library (Figure 2; Supplementary File 3). Since the varying efficacy of PTI suppression by pathogen effectors may explain PR (Niks and Marcel, 2009), components of the PAMPs response rather than ETI defense were expected to be found. Accordingly, the SSH library contained several receptor-like kinases (RLK) well characterized as PRRs. In particular, a sequence was identified with similarity to the RLK gene *Xa21* of rice which confers broad-spectrum resistance against the most of the isolates of bacterial pathogen *Xanthomonas oryzae* pv. *orizae*. An ortholog to this gene has been identified in an SSH library in barley following leaf rust infection in a non-host interaction setting (Neu et al., 2003). Taking into account the close relationship between non-host resistance and PAMP-triggered immunity (Niks and Marcel, 2009; Zellerhoff et al., 2010; Douchkov et al., 2014), these observations suggest that homologs to this gene play a role in basal resistance mechanisms against fungi in different cereal species including oats. Two other interesting sequences were: one similar to brassinosteroid insensitive 1-associated kinase 1 (*BAK1*) which functions as a signal enhancer of PAMP perception in *Arabidopsis* plants (Böhm et al., 2014), and a sequence similar to the chitin elicitor receptor kinase (*CERK1*) which recognizes chitin oligosaccharides and cooperates with other chitin-receptors to trigger the chitin response in a wide range of *Arabidopsis* and rice cells (Liu et al., 2013). Additional transcripts showed evidence for defense signaling via protein kinases. Singularly it was detected a transcript for a member of the mitogen activated protein kinase cascade (an MPKKK) that showed similarity to the *EDR1* gene of *Arabidopsis*. This gene is involved in the fine-tuning of responses to biotic and abiotic stresses via the control of the MAP kinase cascade (Zhao et al., 2014). Elicitor-host binding proteins with a potential role in recognition were also identified in the SSH library, such as wall associated kinases (WAK). Involvement of these receptors in partial resistance is evidenced by the maize gen *ZmWAK* which confers quantitative resistance to the soil-borne pathogen *Sporisorium reilianum* (Zuo et al., 2015).

Ca^2+^ and its binding proteins (such as calmodulin and the calcineurins) participate in early recognition of pathogen infection and pass the signal to downstream target molecules. The oat glutamate receptor identified here might acts in the manner of those recently found to participate in pathogen recognition via the mediation of calcium signaling and nitric oxide (NO) production, a mechanism similar to that seen in mammals (Vatsa et al., 2011). Moreover, the expression of alcohol dehydrogenase III was detected, the involvement of which in NO signaling (under the guise of S-nitrosogluthatione reductase) is required for basal resistance and non-host penetration resistance to different pathogens in *Arabidopsis* (Feechan et al., 2005). An ortholog gene in soybean shows different expression levels in genotypes susceptible and with PR to *Phytophthora sojae* (Wang et al., 2012).

The presence of all these transcripts in the oat SSH library suggests that the increased expression of proteins involved in PAMP perception and the derived cell signaling processes are important components of the defense response of the PR genotype MN841801-1. Most of the transcripts described so far are part of the oat novel isogroups described in this work, suggesting their specific function in pathogen response.

Elements of the early defense response (designed to interfere with the invading pathogen) were also detected in the oat subtracted transcriptome. These included transcripts for enzymes involved in controlling ROS production (cell wall peroxidases), ROS-scavenging via the ascorbate-gluthatione cycle, NO signaling and cell wall modification. The production of ROS in the apoplast has been observed upon infection with various pathogens and in treatments with different PAMPs (Wu et al., 2014). Levels of apoplastic ROS produced in response to pathogens are influenced by various oxidative enzymes found in the plasma and cell wall (Mahalingam and Fedoroff, 2003). No transcripts for the enzyme NADPH oxidase (Rboh), which has been envisaged as the main source of apoplastic ROS, were found in the present library. Similarly, genetic analysis of the expressed genes associated with quantitative resistance to different pathogenic fungi in wheat failed to detect Rboh (Huang et al., 2013; Kugler et al., 2013); neither were such transcripts detected in the response to non-host pathogens in *Lotus* (Bordenave et al., 2013). In contrast, peroxidase expression has been connected with quantitative resistance in wheat (Huang et al., 2013), This supports other evidences indicating essential roles for peroxidases in the PTI response, both in the deposition of callose, the oxidation of phenolic compounds, and the expression of defense genes (Daudi et al., 2012). Figueiró et al. (2015), who studied the role of ROS in the activation and establishment of resistance in oat cultivars showing contrasting responses to crown rust, reported that hydrogen peroxide and superoxide levels were not substantially increased in the partially resistant oat URS21 after inoculation with *P. coronata*. Together, these observations suggest that the PR showed by the oat line MN841801-1 is linked to a reduced ROS level compared to those seen in ETI responses, in which strong and prolonged oxidative bursts caused by Rboh correlate with the hypersensitive response (Katagiri and Tsuda, 2010). Moreover, the strong expression of enzymes involved in photorespiration would help to minimize ROS accumulation in chloroplasts. For example, in *Arabidopsis*, a loss-of-function mutation in the gene coding for serine hydroxyl-methyltransferase resulted in broad spectrum susceptibility to biotrophic and necrotrophic fungi (Moreno et al., 2005). Similarly, the inhibition of the photorespiratory enzyme glycine decarboxylase is central to the action of the fungal toxin victorin, a pathogenicity determinant of the fungus *Cochliobolus victoriae* (which causes Victoria blight in oats) (Navarre and Wolpert, 1995). Homologs to transcripts for these two enzymes were highly redundant in the oat SSH library, suggesting photorespiration is activated by the pathogenic fungus used in this work.

The modification of plant cell structures elicited by fungi is thought to reinforce the cell wall at fungal penetration sites (Underwood, 2012) and to bear close relationships with ROS signaling pathways. The present analysis identified several genes responsible, following infection, for reinforcing the cell wall via lignification and callose deposition. Two lignin biosynthetic transcripts, cinnamoyl-CoA reductase and PAL, were found, as were transcripts coding for callose synthase (involved in the synthesis of callose using UDP-glucose as a substrate). The expression of PAL has been reported in wheat during race non-specific *Lr34*-mediated resistance to leaf rust (Hulbert et al., 2007) and in adult plant resistance to stripe rust (Huang et al., 2013). PAL is a key enzyme in the phenylpropanoid pathway, which in oat also leads to the biosynthesis of avenanthramides. These phytoalexins are induced in oat leaves by spores of incompatible races of crown rust (Mayama et al., 1982). The enzyme hydroxycinnamoyl-CoA:hydroxyanthranilate N-Hydroxycinnamoyl transferase (HTT) catalyzes the final step in avenanthramide biosynthesis. No HTT transcripts were found in the present SSH library, suggesting that, in oat quantitative resistance, the phenylpropanoid pathway plays a greater role in cell wall lignification for preventing the invasion or expansion of pathogens than in the synthesis of secondary metabolites with antimicrobial activity (at least at the stages of infection studied here). Further, deposits of callose would potentiate lignification at relatively early stages of pathogen invasion. It has been reported that callose deposition mediated by ROS does not require any H_2_O_2_ produced via the action of Rboh (Luna et al., 2011). These observations agree with the absence from the present SSH library of induced transcripts for this enzyme, as discussed above, and the presence of those involved in callose production.

### Abscisic acid, but not salicylic acid, may be an active player in partial resistance

It is well established that hormones that are active players in the ETI response may also affect the PTI and PR responses. Thus, variation in the levels of salicylic acid, jasmonic acid, ethylene and brassinosteroids may influence the expression of defense-related genes and decide the outcome of an infection (Pieterse et al., 2009). Moreover, it has been reported that barley genotypes showing PR to *P. hordei* activate signaling pathways related to a broad range of plant hormones (Chen et al., 2010). The various defense pathways induced by these hormones are interconnected through hormone-mediated signaling pathways forming complex regulatory networks with shared elements (Bari and Jones, 2009; De Vleesschauwer et al., 2014). In agreement with this, about 40% of the transcripts found in the present SSH library that were annotated under GO terms related to defense have previously been described as expressed in response to one or more of these hormones. Several of them are even involved in their biosynthesis and direct signaling pathways (Supplementary File 3). However, within these oat isogroups, no homologs were found encoding essential components of either of these phytohormone signaling pathways (as would be, for example, the *Arabidopsis* genes *NDR1, NPR1, EDS1, PAD4*, or *JAR1*). These genes are mainly involved in the race-specific response mediated by *R* genes (Bari and Jones, 2009). Similarly, no transcription factors of the TCA family, nor with WRKY domains, nor consequently the pathogenesis-related genes regulated by them (i.e., those coding for PR1 protein, chitinase, or thaumatin) were found. The absence in the present SSH library of crucial regulators of these pathways indicates that the crown rust isolate used induces expression levels of these host key genes similar to those seen in mock-infected oat plants. Interestingly, genes involved in these hormone signal transductions were also missing from an SSH library of wheat showing adult- resistance to stripe rust (Huang et al., 2013). In this context, the PR response at the time points studied would appear more similar to that expected for a susceptible response than for an ETI response mediated by *R* genes.

It is noteworthy, however, that isogroups related to abscisic acid biosynthesis and signal transduction mediated by this hormone are well represented in the present library. Some of these genes, such as those coding for zeaxanthine epoxidase and violaxanthine de-epoxidase (enzymes involved in abscisic acid biosynthesis) were found in the novel isogroup set. The role of abscisic acid has been controversial and a wide range of mechanisms that might explain its effects have been proposed (review by Asselbergh et al., 2008). Some evidence indicates that the accumulation of this hormone at the site of the infection suppresses the defense responses to biotrophic fungus mediated by salicylic acid. This might explain the lack of the specific regulatory elements of this defense pathway among the transcripts detected in the present work. However, a positive role for abscisic acid in PR is supported by the observations of Häffner et al. (2014) on quantitative resistance to *Verticillium longisporum* in *Arabidopsis*. In general, the positive influence of abscisic acid is exerted on early-acting defenses related, for example, with callose biosynthesis (Flors et al., 2005), and again supports the essential role of the cell wall-associated defense system in the observed oat PR response.

Important manifestations of the PR shown by oat genotype MN841801-1 include high levels of chlorosis and the early formation of telia, leading to the replacement of infective urediniospores by teliospores (Manjón, unpublished). These signs have previously been reported in oat (Šebesta and Bajer, 1992) and might be caused by the induction of a late defense response leading to premature plant cell death and senescence close to the infection points. This would eventually stop the invasion of any fungi that penetrated and colonized successfully. The expression of genes related to the biosynthesis of abscisic acid suggests that an accumulation of this hormone might play a role in promoting early senescence in PR. This would be in agreement with observations on the promotion of leaf senescence exerted by abscisic acid under different stresses (reviewed in Lim et al., 2007).

### Diversity of expression patterns of defense-related genes in partial resistance and susceptibility

Differences between PR and susceptible plants in terms of the relative expression of isogroups selected from the different functional categories (Table 1; Figure 4) were, generally, quite similar to those observed in studies of other host-pathogen interactions involving partial and quantitative resistance (Wang et al., 2012; Jubault et al., 2013), and less strong than those described between resistant and susceptible plants in pathosystems based on *R* genes (Zhang et al., 2013). The present results confirm those of microarray studies suggesting the existence of substantial overlap between the expression of defense response genes in susceptible and PR genotypes following infection (Chen et al., 2010). The genes induced under both conditions have been deemed to produce basal defense transcripts (Coram et al., 2008), an idea with which the results of the present work agree. The identification of the most important genes requires detailed observations be made of the overall pattern of expression at several time points. The present study, which is limited in terms of the number of studied genes, the time points sampled, and the number of genotypes analyzed, shows complex expression patterns even for genes putatively involved in the same defense response pathway (Table 1, Figure 4). For example, isogroups involved in pathogen perception, such as those coding for transcripts with a role in signal perception and those involved in the Ca^2+^ mediated response, showed no strictly coincident expression patterns. This suggests that the ability of both the PR genotype and the susceptible genotype to detect the pathogen is similar. However, a key difference between them is the time to the effective activation of the defense mechanism. The present results indicate that, although most of the genes expressed in response to *P. coronata* were expressed in both genotypes, the activation of 70% of the genes analyzed by RT-qPCR was later and less sustained over time in the susceptible genotype. This result was particularly clear for transcripts from isogroups annotated under hormone signaling, response to oxidative stress, and cell wall modification. These results suggest that, compared to the susceptible Noble-2, the relatively stronger and/or earlier signaling events in MN841801-1 allow the latter to more successfully delay and/or attenuate the effects of *P. coronata* virulence factors. Further observations on the quantitative transcriptional response of oat isogroups are needed to confirm this hypothesis.

In summary, a large number of genes represented by transcripts expressed in a PR oat genotype inoculated with *P. coronata* were identified in an SSH library. The annotation of transcripts allowed them to be related to important steps in the overall plant-pathogen interaction. Of special note were those involved in pathogen recognition, cell-wall modification, oxidative bursts, and abscisic acid biosynthesis and signaling. Preliminary analyses of expression profiles of a set of selected genes were evaluated at different times after inoculation with the fungus. These revealed differences between the PR and susceptible genotypes, especially a shorter time until the activation of defense in the PR genotype. Finally, a number of genes not previously described in oat transcriptomes were identified in this work, contributing toward our molecular knowledge of this crop plant.

## Author contributions

YL and EN undertook the SSH construction work, transcript analysis, the study of the expression profiles and participate in the interpretation of the results. CP was involved in the RT-qPCR experiments; JM was involved in pathogen management, performed the inoculations and the infection analyses; AF participated in the design of the study and interpretation of the results; EF conceived the study, participated in its design and the interpretation of the results, and coordinated the work. All authors have read and approved the final manuscript.

### Conflict of interest statement

The authors declare that the research was conducted in the absence of any commercial or financial relationships that could be construed as a potential conflict of interest.
