# Supplementary material for: Identification of Genes in a Partially Resistant Genotype of Avena sativa Expressed in Response to Puccinia coronata Infection
Source: Front Plant Sci. 2016 May 31;7:731. doi: 10.3389/fpls.2016.00731 (PMC4885874; doi:10.3389/fpls.2016.00731)
Supplement: Supplementary file 1 [file DataSheet1.docx]

**SUPPLEMENTARY FILE 1. FASTA SEQUENCES OF ISOGROUPS. IN BOLD ARE THE SEQUENCES OF THE NOVEL SET**

>Isogroup_1 TCGAGCGGCCGCCCGGCCGAGGTACTTGAATCACCAGTTGGAGCCGAACCACGATAGAAGCTCGCAATACCAAAGTCTCCCAAGTGAGCAGTCATATCATCATCAAGAAGGATATTGCTCGGCTTCACATCACAATGGATAATCGACCTGCTTCCGCTATCATTGTGCAGATAATCTAAAGCATCAGCAATGTTAACAATTATGTTTGTCCTCTGAGTTATATCTAGACACTTCTTATATTTCCCATCGGCTTTATGATGCAACCATGTGTCCAGGTTTCCATTTGGCATGAATTCATAAATGAGAGCTTTGAAAATTCTTCCTTCCATATCCAACGTCGAGCATACCGTTAGGATAGAAACAAGATTACGATGCTTAATGTTTCTCAATGCTTCGCATTCTGACAAGAAACTTTTCTCTGCACCGTGCATATCAAGGTCAAGCACCTTTACTGCCACTTCTAGTTTTCCTTGGGCCAATTTTCCCCTGTAAACCGAACCATAGCCTCCTCTTCCAATGAGGTTACTCTCTGAGAAGTTCTGTGTAGCTTGAGCTAAATCATCGTATGACACTTTAAGGAGATGTTCACTGAAAGGAGGAACAGATTGGCCTGGTCTTGCCATCTTCTCGGTGAGCAGAAAGTAGACCAACAGTACTGCAATGAACTTCTACCGTCTTCTAGCTGTAATATTTCCAAACTAGTTACAAGGGTGGATGCTCATTCTCATTGAGTTGGTGATGAAATGATTTGTGAAATCTGAATTTGTGGCTCATCAAACTTTGGCTGCTTGAGCAGGTGCGCCGACCCTCTCAGCAATGATTTGTTCATAGAGATCTCTAATCTTCAGACCTACGATGGTTTGGAAGAGACCAGTTCCGTTGTTGCTGCCTGGGAAGTCTGCATGCTTTAGCATTTGCTGTGTCACCTCCCCGTAGAACTTGGTCGAGAGGTTGCTTAGGTGGCTCTCAACATAGATGAGTTCATCTAGTCTGTCAAATTGCCCATCCATCTCCAGCACCGATACCTCTTGGCCAAAGTAAGTGTCTGGGCCATAGGAAAATTTGATGCCAGGGTAAGAGCATGTAAGCTTTCTTCCACAAGGGATCCAATTGATGGTTGATCCTTCATCGAAGAGGTAAACCGGGTTGAAGAACTTCACGCCTTCTTTCATGATCAATTTAACACGCAGCACCTTTCCTTCATTGTCATCAGGAATCAGCTGGGTTGGCAGAACTTCAATCACAGCATCGGCGTATTGCTTCTGCGGATCAATAAATGCATCAAAATCTGGTTTCCGGGCTTCAATGCTAGCCTTGATGCTTTCAAGGCTGTGCCCACGCTCTGCCATGTCCCTCTGAATTTTCCATGCAAATTTAACCTCATTGCTGATGTCTAAGTAGATGCTGAAGTCAAGGAGGTCTCTCACACGTGCATCGTACCTGCCCCGGGG

>Isogroup_2 CAATAAGATTGTAATGTAATAAGAATCTTTCACCGGGAGGATTGACAGAAAAAAACTGTATCAGAAAAAGAAGATTGTTTACATCTGCACAACCCACCTAATCTATAAAACACAAATCCTGCAAGTGCTGCTATGTGTGGCATGAAGATTATTACTGGTCGGAACTGACTCCAAAGGTAACATTAACATTCCGGAGTTGAAGAAAGCTGCCGAAGCAAACAAGGACAACCTGTCTGCTCTGATGGTTACCTATCCTTCAACCCATGGAGTCTATGAAGAAGGCATTGATGAGATATGCAGGATCATTCATGACAACGGTGGGCAGGTCTATATGGATGGGGCCAACATGAATGCTCAGGTTGGGTTGACAAGCCCTGGTTTTATTGGAGCAGATGTTTGCCATCTTAACCTTCACAAGACATTTTGCATTCCACATGGTGGAGGTGGTCCTGGCATGGGTCCTATTGGTGTTAAGAAGCACCTGGCACCATTTTTACCATCTCATCCAGTGATCCCAACTGGTGGCTTTCCCCTCCCTGAGAAAACCGACCCTCTTGGCTCCATTTCTGCTGCTCCATGGGGATCTGCTTTGATTCTTCCAATTTCCTACACATACATAGCCATGATGGGGTCTCAGGGACTCACTGATGCTTCAAAGATTGCGATCTTGAATGCAAACTACATGGCAAAGCGTCTGGAGAAACACTACCCAGTTCTTTTCCGTGGAGTCAATGGAACTGTTGCCCATGAGTTCATCATTGATTTAAGAGGGTTCAAGGCAACTGCTGGTATAGAGCCTGAGGATGTTGCGAAGCGTTTGATGGACTATGGATTCCATGGACCAACCATGTCATGGCCTGTTCCAGGCACACTTATGATTGAACCCACTGAAAGTGAAAGCAAGGCTGAACTAGACAGGTTCTGTGATGCCCTTATTTCCATCAGGGAAGAAATTGCACAGGTAGAAAATGGCATAGCAGATGTGAACAACAATGTCCTGAAGGGCGCTCCTCACCCACCCCAACTCCTGATGAGTGACGCATGGACTAAGCCATACTCCAGGGAGTACCTGCCCGGCGCCGACTCGATAGCGTGGTCGCGGCCGAGGTACATTCGAACGACAAAGTGCTGGCTTGATGGTAAGATGAAACATGAAGCGTTTCCTCGGATGGATGCGGACTTGTGTCAGAATATTGAAAGTGCAATGGTTTCGATGATTTTAGCATTGCCTTCAGGTGACCAGTCAGATATCCTGTCGGAGTGGATGCTGAAAGCAGACCAGTTCAGATACCCTGACCTCACTGAGGCATTTGAGATGTGGTGTTACCGCAGTAAAACAGCAATCAGACGGCTGAAGCCAAGGATAGATAATGGTGACAAACCTATCAGATTGTAAGGCTACCATTAATTTGATCATTCTGTTGTAGACTGTAAGAGCGAAATTAGCTGGTTG

>Isogroup_3 GAGACGCCGACCACACCACGACTAGCGTGGTCGCGGCCGAGGTACCTGAGAAAACAAAAGAATCTCCTGGTGGACCATGGCAATTCATTGGTCTGTTGCCCCTGTTTGACCCCCCGAGGCATGACAGTGCTGAGACCATTCGCAAGGCACTTGTTCTTGGTGTCAATGTCAAGATGATCACTGGTGACCAGCTTGCTATTGGAAAGGAGACCGGAAGGAGGCTCGGTATGGGCACAAACATGTATCCTTCCTCTGCATTGCTTGGCCAAAGCAAGGACGCTTCACTTGAGTCACTTCCTGTTGATGAGCTGATTGAGAAGGCTGATGGTTTTGCTGGAGTCTTCCCTGAGCACAAGTATGAGATCGTCAAGAGGCTTCAAGAGAAGAAGCACATTGTTGGTATGACTGGAGATGGTGTCAATGATGCTCCTGCGCTTAAGAAGGCCGACATTGGTATTGCTGTCGATGATGCTACAGATGCTGCCAGGAGTGCTTCCGACATTGTGCTCACTGAGCCAGGTCTTAGTGTCATTATCAGTGCTGTTCTCACCAGCAGATGCATTTTCCAGAGGATGAAGAACTACACCATTTATGCTGTTTCCATCACCATCCGTATTGTGCTTGGCTTTATGCTTATTGCTCTCATCTGGAAATTCGATTTTGCTCCCTTCATGGTGCTTATCATCGCCATTCTCAATGATGTACCTCGGCCGGGCGGCCGCT

**>Isogroup_4** GAGACGCCGACCACACCACGACTAGCGTGGTCGCGGCCGAGGTACCTGAGAAAACAAAAGAATCTCCTGGTGGACCATGGCAATTCATTGGTCTGTTGCCCCTGTTTGACCCCCCGACGGACGGACTAGTACGAACGTAGGTCGTCGTAGGAACCGAACCTTACGTTCCGAACGGAACGGACCTTACGTTTTCGTTTTCGGTTTGGGTTCGTAACTAAGTTCGTAACGAAAGTAGTAGTACTACCTACGGTTGGGTACCGAACCGACGTTCGTTCGTAC

>Isogroup_5 GAGACGCCGACCACACCACGACTAGCGTGGTCGCGGCCGAGGTACCACCATCATGACCTGATATCTGAATTACGTCTGCGTTTGCCTTCGATACTCCAGAGGCTACAGTTCCAATCCCTGCTTCAGCTACAAGCTTTACCGACACCTTCGCTTTAGGATTGATCTGATGGAGGTCAAAAATTAACTGCGCAAGATCCTCAATAGAATAGATGTCATGGTGTGGTGGTGGGGATATGAGAGGAACACCAGGTTTGGAGTTTCTCAACCGAGCTATGTATGCACTGACTTTTTTTCCCAGGGAGTTGACCCCCTTCACCAGGCTTTGCCCCTTGTGCAATCTTTATCTCGATTTGTTCAGCATTAACTAAAAATGTTGGTGTAACACCGAAACGCCCGGATGCAACCTGCTTAATGGCACTTGTGGCAGTGTCCCCGTTCTGAAGACCTTTGAGATGAGGAAGTGTTGGAGAATACCCATCAACAACATCTGCAAGGGGACTCCAGCGGATTGGGTCCTCGCCACCTTCTCCTGAATTAGATTTCCCACCTATTCTGTTCATAGCAATTGCAATTGCTTCATGTGTTTCTCTGGAAATAGCTCCCAAAGACATTCCACCTGTGCAAAAACGTTCGACAATAGAGGTTGCAGGCTCAACTTTGCCTATAGGAATAGGGGGCCTTTCACTCTTCAACTCTACAAGATCTCGAAGAACATTGACAGGGCGGCTTGCAAGATGTTGTTGGTAGATGGTGTACCTCGGCCGCGACCACGCTAGTCGTGGTGTGGTCGGCGTCTC

>Isogroup_6 GTCGCGGCCGAGGTGTGAGTTCCACCCCTCCCAGCCGTCATCTGCAAACTACACCATGGCTCCTCTGACTCCCAGACTTGTAGTGCCCATAGATGTGAAGAAGAAGCCTTGGGAGCAAAAGGTTCATCTCCACAACCGCTGGCATCCAGATATCCCTCCTGTTGCTGATGTAACTGAAGGGGAATTATTCCGTGTTGAGATGGTCGATTGGACTGGAGGACGGGTTAGAGATGATGACTCCGCAGATGATATCAAGTTTCTGGACCTCACAATTACTCATTATCTTAGTGGCCCCTTTAGAATAGTTGATGCTGAAGGGGTCCCAGCTTCACCCGGTGATCTTCTTGCGATAGAGATCTGCAACCTTGGTCCGCTTCCTGGTGATGAGTGGGGTTATACTGGAATATTTGAAAGGGAGAATGGAGGTGGATTCTTAACAGACCACTTCCCAAGTGCAAGAAAAGCCATTTGGTATTTTGAAGGAATTTATGCATACTCCCCACAGATACCTGGTGTTCGGTTTCCGGGTTTAACTCATCCTGGTATAGTGGGAACTGCACCATCTGCTGAACTTCTTAATATATGGAACGAAAGGGAAAGGAAATTGACCGAGACAAGTCATGAGTCTCTGAAACTGTGTGAAGTTCTTCACCAGAGGCCCCTTGCTAGCTTACCAACGTCCAAGAACTGCTTACTTGGAAAGATTGAAGAAGGGACTGCTGAATGGCAAACGATTGCAAATGAAGCAGCAAGAACTATTCCTGGAAGAGAGAAGGGGGGAACTGTGACATTAAGAATCTAAGCAGAGGTTCCAAAGTTTATCTCCCAGTATTTGTTGAAGGAGCAAATTTGAGTACCTGCCC

>Isogroup_7 CAAATCTCTGCTGGCTGCTGGTGCTGGTGATAATGCTCTGGTCTCTATCTATGCCGAGGACAATTATCAAGTAAGATGAGGTTTACATCTTGTGGCATGCAATGGCAATGCAATCAGTAGGCCATATGATTGCCCAACTGTTGGATATGCAGCTGGTTCAGCTACAAGATACAAGATGATGCAAAGCCTGGTCAGCGACATTGGTGGTGGTTGTAGTGCTGTGGCTTTTGGTCCATATTGCTTGTGTTGGGCATCTACATCTCTGATCGGCGTGTGCAATTTGTAAGATACAAAACAAGATTCATTAGCCTCCCCTGCAAAAGGTTATGAAAGTGATAGGCAGTCATGGTCCCATCAAGTGAACTAGGAGGCTTCCCAAATAATCATACCTAGGTTAGCTGAAATGTAAATAGCCATCCGCATTTGGATTGTTGTTCTCTTAGTCAGTTTTCTTCCTTTCCCAACTGTAAAAAGGTTTGCCGATGCTTCATATCATAAGTCGGGTTGGCGGGGCCTGCTCGGTCAAGTGGTTGTTTATATTGGGCCAGGGAGGAAGAATGCATGCGCATGCCTCTTCACAGATGTTCTCCATAGATAGATGCTCCTTTTCCAGCTAATATAGCAGTAGAGGTGAGTTGTTACCATGGCTTGCTTGAATCAGTATTCTCCAGGTCATTCATTGTAAGCATTGGACTATCATCCAGTTTCGTCGTCACAGCTCGGTCGTCTTCGTTGTCCATTTG

>Isogroup_8 AGGTACATAGTCCCACCATAACCATGGCTTGCCAGTGTCATTAGCAACCTTGAATACACAAACTTGATGTAGATTTTGAACCACGACATCTTTTCCATCATATCCTTTGCTGAAATCCTGCTCTGGGTCCGGTGCACAATATCTGCCATGGTTGATGCACTGGGATTTGCATTGCTTGCTGACAACAAAAGCTTCTGGGCAATACCATGTAATATAATGTGGAGTGAACTGGGTGTAACCCTTCTGCTCAAGAACTTGTGCAGTTCCTCTAAAGCTCTTCACAAAGTCCATTTGCATATCACATTTAGGACCACATTCATCATTACTGTTTGTCCAGAATTCATACTCTACACGCTCATCAGGATGGGGAAGTGACTCCCTCCAGTCCAAAAGGACACTAACCATTTCACCTTTTTTCAGCAGCTTTCTTGAGGTCATCGCCCAATTTCTTTGTTATAAGCACTGAGGGAATAGTGATGTTTTCCATGTGTTTTGTGCCTGTATCTTCTGGGTTGTCCATTGTGATTAAAGGCTCATCCTTGCTATCCACAACAAGAACTGCTGCAGCTCCAGCAAGTTGTGCATTCCATGCCTTTATTGTGAAAAAGCACTCTCCGCGGTCTATGAGGACGAAGTTTGGTCGCTCTCCTGATTTGTGCGACTTGAAGGAGGTTTCGAACTCCTTGCAGGCGTCCTTGTTGCCCTTGGGGTAGACGACCCATCCCTGCAGGGTTCCCCCG

>Isogroup_9 GAGAGACGCCGACCACACCACGACTAGCGTGGTCGCGGCCGAGGTACAGAATTTGTTTGGTGAGCTTGGTATTAATGACCGCTTGCAATGGAAGGAGCACTCTATGATATTTGCCATGCCAAACAAGCCAGGAGAATATAGCCGTTTTGATTTCCCAGAGGTTTTGCCAGCGCCCTTAAACGGAATATGGGCCATACTGAAGAACAATGAAATGCTTACTTGGCCGGAGAAGGTGAAGTTTGCTATTGGACTTCTACCAGCAATGCTTGGTGGCCAAGCTTATGTTGAAGCTCAAGATGGCTTAACTGTTTCAGAGTGGATGGAAAAGCAGCACAGGGTGTTCCTGATCGAGTCAACGATGAGGTTTTTATTGCAATGTCGAAGGCGCTCAATTTCATAAACCCTGACGAGTTATCCATGCAGTGCATTCTCATTGCTCTAAACCGGTTTCTCCAGGAGAAGCATGGCTCGAAAATGGCATTCTTGGATGGTAATCCACCTGAAAGGCTATGCATGCCTATTGTTAACCACATTCAGTCTTTGGGTGGTGAGGTCCGCCTGAATTCTCGCATTAAAAAAATTGAACTGAACCCCGACGGGACTGTGAAGCACTTTGCATTGAGTGATGGGACTCAAGTAACTGGAGATGCTTATGTTTGTGCAGCACCAGTTGATATCTTCAAGCTTCTTGTACCTCGGCCGCGACCACGCTAGTCGTGGTGTGGTCGGG

>Isogroup_10 CGTGATGGTCGGGAATGTGCCGCTGGGCAGCGATCATCCCATAAGGATTCAGACTATGACCACTTCAGACACCAAGGATGTCGTCAAGACCGTGGAAGAGGTCATGAGGATAGCAGATAAAGGGGCTGATTTTGTTAGAATAACCGTCCAGGGTAAGAAGGAGGCTGATGCCTGCTTTGAGATTAAGAACACCCTTGTTCAGAAGAATTACAACATTCCCTTGGTCGCCGATATTCATTTCGCACCAGCAATAGCTTTAAGAGTGGCTGAATGCTTTGACAAAATTCGTGTTAACCCAGGAAATTTTGCTGATCGCCGTGCACAATTCGAGCAGCTGGAATATACTGAAGACGATTATGAAAAGGAGCTTGAGCATATTCAGAAGGTCTTTTCTCCATTGGTTGAGAAGTGCAAGCAGTATGGAAGAGCCATGCGTATTGGAACAAATCATGGTAGTCTTTCTGACCGCATAATGAGCTACTATGGAGATTCTCCAAGGGGAATGGTTGAGTCTGCTCTGGAATTTGCTAGGATCTGTCGCAGTTTGGACTTCCATAACTTTGTATTTTCAATGAAAGCAAGCAACCCTGTTGTCATGGTCCAAGCATATCGATTGCTTGTAGCAGAAATGTATAACCTAGGATGGGATTATCCTTTGCACTTGGGAGTTACTGAAGCTGGTGAGGGTGAAGATGGGAGGATGAAGTCTGCTATTGGCATCGGAACACTTCTGATGGATGGCGTGGGTGATACAATCCGTGTATCCCTCACAGAACCACCAGAGGAGGAAATTGATCCTTGCACTAGATTGGCAAAACTTGGAACTCAGGCTGCAAACCTTCAAATAGGGGTGGCCCCATTTGAAGAAAAACATAGGCGCTATTTCGATTTCCAGCGTAGAAGTGGTCAGTTGCCTTTACAGAAGGAGGGTGAGGTAGTAGATTACAGAGGTGTCCTGCATCGTGATGGCTCTGTTCTGATGTCAGTTTCATTGGATCAGCTGAAGGCTCCAGAGCTCCTTTATAAGTCTCTTGCTACGAAGCTTGTGGTTGGAATGCCTTTCAAGGATTTGGCAACTGTAGATTCAATCCTTTTGAGGGAACTTCCCCCTGTAGAAGATGCTCAGGCTAGGCTTGCACTCAAAAGATTAGTGGACATCAGCATGGGTGTGTTGACTCCCTTATCAGAGCAATTATCAAAGCCACTCCCACATGCAATTGTGCTCGTCACCCTGGACGAACTATCAAGTGATTCACTCAAGCTATTGCCAGAAGGCACTAGATTTGCTGTTACGCTCCGTGGAGATGAATCATACGAGCAGCTAGATGTTCTTAAGTGTGCTGACAATATAACAATGTTGTTACATAACGTTCCATATGGTGAAGAGAAGACTGGCAGAGTACCTCGGCCGCGACCACGCTACGAGCGGCCGCCCGGGCAGGTACTCCTGTTCGATTCCGAACCATGGCACTTGTGAAGAAACCTTGGGGTCACTGAAAATTTGTGCAGCCCTGTGGCGCTTGTTAGTAGGGATGGGCTCCCCTTGTGGTGT

>Isogroup_11 CGAGGAGAAGCTTAAGAAGGAAACGTTTGCGCTCTTCTTCCTAGCAACATATGGCGATGGGGAACCTACTGATAATGCGGCAAGATTCTACAAATGGTTCACTGAGGGGAAGGAGAAGGAAATTTGGCTTAAAGATTTTAAATATGCTGTATTTGGGCTCGGGAACAGGCAATACGAGCATTTTAATAAGGTTGCTAAGGTGGTGGATGATCTACTACTGGAGCAGGGTGGCAAGCGTCTTGTTCCTTGTGGCATGGGTGATGATGACCAGTGCATTGAGGATGACTTCACCGCCTGGAAAGAATTAGTATGGCCAGAACTGGACCAACTGCTCCGTGATGAAGATGATACTGCTGGTGCATCCACTCCTTACACTGCTGCCATACCTGAATACAGAGTTGTATTTATTGATAAATCAGATTTAGTGTTTGAAGATAGATCCTGGACACTTGCCAATGGCAATGGTGTTATTGATGCCCAACACCCTTGCAGGTCTAATGTTGCTGTTCGGAAGGAACTCCATAAACCAGCTTCAGATCGCTCTTGTATTCATCTGGAATTTGACATTTCAGGCACTGGTCTCATGTATGAAACAGGAGATCATGTTGGGGTTTATTCAGAAAATTCTGTTGAGACAGTGGAGCAGGCTGAAACACTGCTTGGTTCTTTCCCCAGATACAGTTTTTTCCATACATGCAGACGCAGAAGATGGATCTCCTCGTAAAGGGGGTTCCTTGGCTCCACCATTCCCATCTCCATGTAGCCTACGGACTGCACTATTAAAATATGCTGATCTACTCAATTCACCAAAGAAGGCTGCTTTGACTGCTTTAGCTGCTCATGCTTCTGACCCGACTGAAGCTGAACGACTAAGGTTTTTGGCTTCTCCTGCTGGAAAGGATGAGTATGCTCAATGGATCGTAGCTAGCCAGAGGAGCCTTATTGAAGTTATGGCTGCATTCCCTTCAGCTAAGCCTCCCTTGGGAGTATTCTTTGCAGCGGTAGCTCCTCGTCTGCAGCCACGATTCTACTCCATATCATCATCTCCGAAGATGGCTCCCTCCAGAATTCATGTGACATGTGCCTTAGTTTATGGGCCTACACCAACAGAAAGGATCCACCAGGGGGTTTTGTTCGACATGGATGAAGAACACACTTCCATTGGAATATAGTGAAGAATGCAGCTGGGCACCAATTTTTGTGAGGCAATCAAACTTTAAGTTACCTGCAGACCCATCCACTCCAATTATCATGATTGGTCCTGGGACTGGGCTAGCACCTTTTAGGGGCTTCTTGCAGGAAAGATTAGCTCTAAAAGAATCTGGAGTTGAATTGGGCTCTTCAGTCCTTTTCTTTGGATGCAGGAACCGTGAAATGGACTACATATATGAAGATGAGTTGCAAAATTTCATTCAGGAAGGGGGCGCTTTCGGAGCTAGTTGTCGCCTACTCTCGTGAAGGACCAACCAAAGAATATGTGCAGCATAAGATGGTGGACAAGGCCACAGAGATTTGGAACATCATCTCTGAGGGTGGTTATTTATATGTTTGTGGTGACGCGAAGGGTATGGCAAAAGAC

>Isogroup_12 AGCGGCCGCCCGGCGAGTACAGGTTGGTAATCAGTGCAGAGTGCAGAATGATGGGCATGTTCGAAGTGTAGAACAGCTTGATTGGATATGAGCCTTGCTGTCCACGGGCATTCTTTGATCTCACTGGAAGCACAACACGGAAGCCTTGGAAATAGATGACTATGAGGAAGACCAAGACAGTAGCAAGCAAATTGGTCACATTTGGCAGATTTTGACGGTAGAAAGCCTCTCGTAGGGCACGGACTTTGTCAGATCGAGTAATCAACAGATGGAACAATGCAATGACAGCCCCTTCAAATTCAGCACCACGTCCACTGTTGATGGTTGTGGGGCTAAACGCCTTCCAGATGATGTTCTCACAGATATTGGTAGCAATGAACAGAGAAATGCCAGAGCCCAAACCATAGCCTTTCTGGAGAAGTTCATCCAGACAGATGACAATGATGCCAGCAAAGAAAAGCTGAAGTATAATGAGAATAGCATTTCCAGTTCCAAGTTGGCTCACACTGCCATACATTCCAGACAGAACATATGCCACTGCTTCCCCAATGGCAATCAGGATGCCAAGCAACTTCTGTGCACCATTCAGCAGAGCACGATCCTCTCTCACACTGTTGTCAACTTCAATAATCTTGGATCCCACCAGAAGTTGCATAACCATTCCAGATGTCACAATTGGAGTAATACCCAGCTCCATGACA

>Isogroup_13 GACGCCGACCACACCACGACTAGCGTGGTCGCGGCCGAGGTACTACCCGTGTGTTGGAGGCATGCCGTTTCAACAATGAGCTTATCTGGCTCCACAAGGAATTTCTGGAACCAAAGAGCCGCACCCTAACTGATTTCGTGGACCCTGTTCTAACAAATTCCAGCTCCCGGGATACACCACGTTCTTCATAGCTGGGGTCATCAAGAAATGGGAGGTCAACCAAACCGCCAGACTCCACATTTTTAAGCCATCCTCGGAGTAAATAGTTTATAACCTCAGGAACCTCATCGTGAGGACAGTGGCCGGCAGGGCTGATTTGATAATAGGGTGCTTCTGGCACCTGTTGCTTGACTCTAAGACCCCAAAAAGGTCCTACCCAAGGATCTTCTTTCCCATACATAAGAGAAATGGGAATACCCTGCCTTTGGCACCTAGAGAGTGCCTCCTGGAAGGATATCTGACCCATTGGAGCACACATAATGGAGGCAAATGATGCAGCAGCTGCTGGGTGTTCAGTTGTCTCTATAATACGCGAGAACACTTTATCAACATTTGTTGAGTGGTCAGCATATACTTGCCTGAGTATCTTCTGTATACTTCTCGGGTCACGTATCTTATGCCACACTGTTTCAGTAAGTTTCCTCACAAATGATGGAAGAGGAAATGTCCCAGCCCATGGAAAAATCTTTGACAAATGAGGAGATCTTGACGGGTTCGGAAGGAAACCCCAAAATGGTGTTGCATTAAGCAATGTGACCCCCTTTACAAGGTGTGGACAGG

>Isogroup_14 CACCATAAACAGCACTTAGACGTGCAAAACCCTGTGGCAGCTCACCCAGCCCATATAACGGATAAATATATGGTGATCCCCCTTGAAAACGTGCAAGAGACTCTGAATATAATTTCATCCTTTTCACAGTATCAAGTGCGGGTTCATTGAGGTGACGATCGTCCCTATGGAGAGCAAGCGCATGGCCAATGAAATCCACTGTATCATCACTTAAACCGTATTTTGCTATCAATTCTTTAGTTGTCACCGTAGTAAGGTCCAATCCCTGATGTGTTTTTGGATCAGCTTCATTGTAATCTTGGACAAAAATGAAGAAGTTCCTTGCTCTGCGCTTCTCGAAAAGACCCATCAATGGAGACTTCAGAGCCTCCATATCAGTGGCGGGAACCTTGTGGATCTTCCCTTTGCTGAAGACAAAGCTACCATCAACAGCTTTAAATGACAAATACTTCGTTACATCAGTGTGAATGAGCGTCCGAACCAATGTCCCGTTTGCCATCATAAACTTTGGAACCATGTCAACATTGTAATCTTTGCTTGAGCCTAGATGTGCCGGGGGTTTGTCTTCCCCTCTAAATTTCTTCCAGAGCTGGTTAAGGTTGAGAGAAGTGGAATCTCCGCCATAGTAGTCATTTCTATCCATGTGCAACACCTTGAGGCCGTCGACGGAGAGGAGGCCGCTGAGGATGCACTCCTTGAGCCCCGTGCCCAGCACGATCACGTCG

>Isogroup_15 GACGCCGACCACACCACGACTAGCGTGGTCGCGGCCGAGGTACACACATTCAGCTGCAGGATGGTTCAGTTGTGCCGTTAGATGAGAAGTCAAGAAAAGCTATTTTGGCAAGTCTCCATGAATTGTCAACAAAAGCTCTGCGCTGCCTCGGTTTTGCATACAAGGAAGATCTTGGAGAATTTGCGACATATGATGGTGAATTCCATCCTGCTCACAAGCTTTTGCTGGATCCAGCCAATTATGCGGCAATTGAGACAGACCTGATATTTGCTGGTCTTGCTGGCCTAAGGGATCCTCCGAGGGAAGAGGTCTTTGATGCTATTGAGGACTGCAGAGCTGCGGGTATCCGTGTTATGGTGATAACAGGAGACAACAAAGAAACTGCTGAGGCAATATGCCATGAAATTGGTGTATTTTCACCTGATGAAGACATTAGCTTGAAGAGCTTTACAGGGAAGGAGTTCATGGCGCTTGAGGATAAGAAGTCACTGTTGCGAAGGAAAGGTGGCCTTCTGTTCTCTAGGGCAGAGCCTAGGCACAAGCAAGAGATTGTGAGGCTGCTAAAAGAAGATGGTGAAGTTGTTGCTATGACTGGAGATGGAGTAAATGATGCCCCTGCTCTAAAACTTGCTGACATTGGTATTGCGATGGGTATTACCGGCACTGAGGTTGCCAAGGAGGCTTCTGACATGGTACCTGCCCGGG

>Isogroup_16 GGCCGAGGTACATAGCGAAGAAATATAGCAACTGCAGTGTTACAGGGATATGCAACTCAACGACACAAAAGGCTTTTATAGAAGAGCGGTGTAGGGAAAATGAGCTCTCCAATGTTGAGATAATTGTAGCAGATATCAGCAAGTTTGAGATGGAGCGTTCTTTTGACAGGATCATATCCATAGAGATGTTTGAGCACATGAAAAACTACAAGGCACTTCTTAAAAAGATATCCAGATGGATGAAAGATGATGCCTTATTATTTGTTCACTACTTCTGCCACAAGACATTTGCGTATCACTTTGAGGACAAGAATGATGATGATTGGATCACAAGGTATTTCTTCACTGGAGGAACAATGCCATCAGCAAATCTCCTTCTCTACTTTCAGGAAGATGTATCTATTGTCAATCATTGGCTTGTCAGTGGCACACACTATGCTAGAACTAGTGAGGAATGGCTGAAACTTATGGATAAGAACATCACTACCATAAGGCCAATCTTTGAGAAAACTTATGGTAAGGAGTCAGCTACCAAATGGATAGCTTATTGGCGGACATTCTTCATCTCAGTCGCTGAACTTTTTGGTTATAACAATGGTGACGAATGGATGGTTGCACACCACTTGTTCCGGAAGAAGTAGAAGTTACCCCTGGCATTTCTAGAGCAAAGTATAATTG

>Isogroup_17 CAGGAAACAACGAATTCCACAAAACAGTGTAGGCACAGTAAAAACTGGACAATAATGGTTGTAAATCGGCAAATTGGATTATACTGATAGAGCATTCGAGCACTAGCAAACTTAAGTCCCAGCTTTCCGACGGCCAACCTGCGCCACGAATCCAGCCTTGATAGGAGCAACTGACCTGGACGAACCGCACTGCTCACAACGAAGGAAGAACAACCGATTCTCCTTGGAAAGGATTGTATCTGGACTCTTGCATCCATTACATATGACATATTCATTGATGTATCTCCTGAGTATTGCTTCAAAGTTCTTTGGAGCAAATCTTCCTTTGATAACCAACCTTTGTTGCCCATCAAGGGATCCACTTGTTCCCATCTCAGCAAGTAAGAACATCATCACATGTTCAGGTTGCCTATGCATTGTTTTGCACAAGTCCATGAAGTTCACAAAAACTGTCTTCTTTGTGCCTTCTCTAAGAACTTGAGGGGGACGCATAACTGTTCTTCGTCTATCACCAGCAAGATCTGGATTATTCTCACGCAGGATATTGAAGACCCTGCCAAGCAGCTCGTCATATTTGTAATCTCTATCAGTCCCTTCCCATGGGTATTGGGTTGTTACTGTTCCACCAAGTACCT

>Isogroup_18 ACATAAAATAAATTAGCACCTCTGCCACTGGCAGGATTTGCAAAATTTGAACCCGCATCCACAAGGGTGCATTACCTCTTACATCTATACCGCGCGCCTTAATGTGCAGCCTACATCATCTATACTCATCCGAAGCAACAACCAACAAATATATTGCTGCGAGGGCAGCATGCCACACGCAAAATCACAGAGCAGCCTGTAGGCCTCGTGTTTCCTTCATGATACAAGATGCACCCTTTTGCCCTCCAAATTGAATTTCCTGCTTCCTTGGAGCTTCCTCTTCAGGAATGATAGAGCTTCACGCCTACAGGAGAAGTCGGAGAATATAGTTGATTGTTCTTCAACAGCTTCCTTAAGCTCCTTCAACTTCATGGATTGAGATGGAGACTGACGAAGTATTTGTTTGCAAAGTTTCTTAAATTTGATTCTAGGTTTAGCATCCACAGGAACTGCTTTCACTGTTTTCTGCTTTATTTCATTTACTTCAGCATCACTATCAGATCCTTCCTCATCTTCATCCTCTTCCAATTCTGATAGTTCAGACTGGGTTGAATTTTCCTCATCGCTATCACCGAGAGATTTTTTGTTCCCTTTCCAACGGTGGCCAGCAATTTTCATTGGCAGGTCCTTGATGCCAAGACCCTGCTTTCCAGATGTAGCTTTGTCCTGGACAAGGTTATATAGATTTTCTTGATCGTCTTCCCCAAACATCTGGCGGACATTGGAAGAACCTTTTTGCGATTTCTTCTTGCGAGATTTTGCTCCTAAAAAAGCCCCCTGATACAAATCCGAACTTGTGCCCCCACCAGTTCACATCTTCAGCTTGGGATACTGCAGCCTGACACATGATGATATCAGTCTCTTCCACACATGACGGTTGAACTTCCTGATCCACCTCACAATCACTTCCATTTTTCCGAACAAGTATGCCTTGAAGATCAACTGCAGAGTAACCCTTGACACTTTTTCCCCTCTCTCTTTTCTTATATCTTCCTTGAGGTCGGGTAACTTTAGCGACTTCATCTTTTGCAGGTTTATCTTTCTTGGGTG

>Isogroup_19 TAGCATTTATTTCTGCACATCTGAGCTCTCTCGGTGACAATACGACGGATACCACTCTTACCATCTGCAATGTTGCTGTTCAACCACATACCGGGAACAGTTGAGCAGATAACAGAAAAAACAAAACAAAAACAAAATAACGAAGAGGAGACTCCATTGTCTCCGTCAATGCAAGGCTAGCACCAGAACCATCCCGGCCATATTCACAACAACCACATCACGCGCTCTTCGCCCTTTTCTCCTCCTCGATCTTAGCCTTGATGTATGAGTAGATGGCAACACCAGCTATTGCAACACACGTCCCGATACCAGTCTGTGTGGTAATTCTGTTGCCAAAAATGATGATCGAGAAACCAATGACGAACACCCTTTTCAACACATTCCCAACAGCATGTGTCAGAGGGGCCACCCGCTCCAATGTGTTTGTAGCAAGCTGGTTATAGAGATGGTAGAACAGTCCTACCAGGAAAAGGTCTGAAACAAATTTTGTCAGACCTACTTTTGCAATTGCATCATTTAACCCATACTGCATAAGTTGGGGTCCTTCAATAATAATTGCTGGTGGTATGCAGACGATAAGAGCAATTATTGAGATGTAAGCATACACGTTGGTGCTATCCATGTCGGTCATGGCTTTCTTGGAGTAAATGCTACGGTAAGTGAAGGAGATGTTTGAGATCATGGCATTGATGAAACCCTTCCAGTTAAATGAAAGTTCCGTGAGGGATGCCATTGAAACACCAAGCACGACGGGGGCAAGAGATAGCCACAGAGACAAGGGAACTGTCTGCCCAAGAACAAATTGGGTAGCAGCTGCATTGAAGAAGGGCTCCAAAGCTTTGATAGTGTGGGCAAATGAGACTGCAACAGTAGCAAAGGACACGTTGCTTGTGACATGACCAAGAGCATGGCACAAAGCCACAGGGAAGAGCAGCTTCAGGAGCGTAGAATTAATAGGCGCGCGCTTTGGGAGACCGACGGCCCAGCTGATGAGGCA

>Isogroup_20 CCTTTCCGCAACATGCCCTATAACATGAATCAGATGTATCCCAAGGGAAACAATGGTAATGTCAATTCATTCAAGATGAATGCTGGGGTCAACAAATATTCGAACAACATAAATGGGAAAGAGGCAAATGGAAAACACAATGGTGGTAACAACAACAATGGAAGCAACAGCAACAGCAACTCCATTGACAAGCGCTTCAAAACATTGCCAACAAGCGAGATGCTACCGAGGAATGAAGTTCTTGGTGGATACATTTTTGTCTGCAACAATGATACCATGCAGGAGGATCTCAAGAGGCAGCTTTTTGGTTTGCCAGCAAGATATCGTGATTCAGTCCGAGCAATTACTCCTGGTCTACCTCTTTTCCTCTACAACTACACGACCCATCAGCTCCATGGGGTGTTTGAGGCTGCTAGTTTTGGAGGATCCAACATTGATCCCACTGCTTGGGAAGATAAAAAGTGTAAAGGCGAATCCAGATTCCCAGCACAGGTGAGGATCCGCATTAGAAAGCTTTGCAAGGCTCTGGAAGAGGATGCTTTTAGGCCAGTGTTGCACCACTATGATGGTCCTAAATTTCGCCTTGAGCTCTCCATAGCAGAGACACTGTCACTGCTAGACCTGTGCAAGTCCGAAGACCTCTGAGTTTGCTGCAGAGGTGTGGTTGCCTTGTGGTTTTAGTAAATATCATCCCTGTAATGTTGTGGTGGAAGGTGGTTCGCGACGTCCTCTCAGTTCATGAAGCGCAAGCTGTTGTTGGTTGCATTTTCCCCAGAAAGGACCCGGTGCTTGGAAAGGCCTCTAAGATGTGAGCCTGTCTGTGTCGTGCCTGTCCCTAATCTAGGTGAAGTGTATGTTCTTCTATTTATGTATTGTAGTTGCAGTACCTGCCCGGG

>Isogroup_21 CTTGATCAACCTGATTGATTCACCTGGCCACGTTGATTTTTCTTCTGAAGTCACAGCTGCTCTTCGTATTACTGATGGTGCTTTGGTGGTGGTTGACTGTATTGAGGGTGTCTGTGTGCAGACTGAAACTGTTCTGCGCCAGGCTCTTGGTGAGAGGATTAGGCCTGTCCTTACTGTCAACAAGATGGACAGGTGTTTCCTTGAGCTACAAGTGGATGGCGAGGAAGCTTATCAGACTTTCTCCCGTGTCATTGAGAATGCCAATGTCATCATGGCAACATATGAAGATGTTCTCCTTGGTGATGTCCAAGTCTACCCAGAGAAAGGGACTGTTGCTTTCTCTGCTGGTTTGCATGGGTGGGCTTTTACCCTTACAAACTTTGCTAAGATGTATGCCTCCAAGTTTGGTGTTGATGAATCAAAGATGATGGAGAGGCTGTGGGGTGAGAACTTCTTTGACCCAGCCACAAAGAAATGGACGACAAAGAGCACTGGCACAGCTACCTGCAAGAGAGGTTTTGTTCAGTTCTGCTATGAGCCAATCAAGCAAATCATCGAGATCTGTATGAATGATCAGAAGGATAAGTTGTGGCCTATGTTGAAGAAGCTTGGTATTGTCATGAAGAATGATGAGAAGGACCTAATGGGCAAGCCTCTGATGAAGCGTGTCATGCAAACTTGGCTTCCTGCCAGTCGTGCTCTGCTTGAGATGATGGTGTATCACCTGCCCTCTCCCTCGAAGGCCCAGAGGTATCGTGTTGAGAACTTGTATGAGGGCCCCCTTGATGATATCTATGCAAATGCTATCAGAAACTGTGACCCTGATGGTCCTCTTATGCT

>Isogroup_22 GTGGCCAAGATTTTGAGAAGGCTGGGGAACTAAGAGACAAAGAAATGGAACTGAAGGCACAGATCACAGCCATCATTGGCAAGAGCAAGGAAATGGTTAAGGCAGAGACCGAGTCTGGTGAAGTCGGCCCTTTGGTCACAGAGGCAGATATTCAGCACATTGTCTCCTCTTGGACTGGAATACCTGTTGAGAAGGTCTCCTCTGACGAATCTGACCGTCTCCTGAAGATGGAGGAAACACTGCATCAACGTGTCATCGGTCAGGATGAGGCTGTCAAAGCTATTAGCCGTGCAATCCGTCGAGCCCGTGTTGGCCTCAAGAACCCAAACAGACCTATTGCCAGCTTCATATTTTCTGGACCAACAGGTGTTGGTAAATCAGAACTGGCTAAAACTTTGGCATCATACTACTTTGGTTCTGAGGATGCCATGGTTAGACTTGACATGAGTGAGTTCATGGAGAGACATACTGTCTCCAAACTAATTGGTTCGCCACCCGGATATGTTGGCTATACTGAGGGTGGTCAATTAACCGAAGCAGTTCGTCGTCGCCCTTACACAGTAGTTCTCTTTGATGAGATTGAAAAGGCACATCCAGATGTCTTCAACATGATGCTTCAAATACTTGAAGATGGAAGATTGACCGACAGCAAGGGACGGACAGTTGATTTCAAGAATACACTATTGATCATGACGTCAAATGTTGGAAGCAGTGTCATTGAGAAGGGAGGCCGCAAGATCGGATTCGACCTTGATTACGATGAGAAGGACACCAGCTACAACAGGATCAAGAGCCTGGTGACTGAGGAGCTGAAGCA

>Isogroup_23 GAGACGCCGACCACACCACGACTAGCGTGGTCGCGGCCGAGGTACGTATGTGATTATATACTTGGTGGCCAACTTGATGGGTCTAGCTCAACAAGAGAGGAGTTTCTTGAGAAATTCAAGAACGCTATTTCTCCAGGATTCGATCCTGATGTTGACTTGGAAAAAGTTGGTATTGCAAATCAAACAACAATGCTTAAAGGAGAAACTGAAGAAATTGGAAAACTTGTTGAGAAGACTATGCTGCGTAAGTTCGGAGTTGAGAATGCCAATGAGCACTTCGTTGCCTTCAACACTATTTGTGATGCCACGCAGGAAAGACAAGATGCAATGTATCAGTTGGTGAAGGACAAAGTTGACCTTATTCTAGTTGTTGGGGGATGGAACTCCAGTAACACTTCTCATTTGCAAGAGATTGGAGAACTCAGTGGAATTCCATCCTACTGGATTGACAGTGAACAAAGAATTGGACCAGGAAACAAGATCAGCTACAAGCTAAATCATGGTGAGCTTATTGAGACAGAAAACTGGCTACCACAAGGGCCTATCACAATTGGAGTTACTTCTGGTGCTTCAACTCCAGACAAGGTTGTTGAGGACGCTCTACACAGGGTGTTTGAGATCAAGCGGCAAGAACTTTTGCAGGTTGCATAAATTTTCAGGAAAATTTGTCAGGGGATAATAGATAATGGCCGAGCAAAGGTTGGTAGAACCATCACACAGCCTATTCCTGTACCTGCCGGCGCCGCT

>Isogroup_24 AGCTTTAGGTGGTAGTGAGGCTCGATTAGTCAAAGCTGCAACTGAGCAATTAAACAAGTTGCCCTTCTATCACTCCTTTTGGAACCGCACGACCAAACCAACATTGGATCTTGCAGATGACCTTCTTAGCATGTTTACTGCAAAGAAAATGGGGAAGGCATTCTTCACAAATAGCGGTTCAGAAGCAAATGACTCGCAGGTCAAACTAGTATGGTATTATAACAATGCATTGGGGAGGCCAGACAAGAAAAAGTTTATTGCACGATCGAAAGCATATCATGGATCGACATTGATATCAGCTAGTCTCACTGGTCTTCCTGCGTTGCACCAGAAATTTGATCTACCAGCACCTTTTGTTCTGCACACAGACTGCCCTCACTACTGGCGCTTCCATCTTCCAGGTGAGACAGAAGAAGAATTTGCAACAAGACTTGCCAATAATTTGGAGAATCTTATCCTCAAGGAAGGACCCGAAACAGTAAAGTCGTTACCAGAGCTTATATTATGCAACAGGTCCCAAGTATACTACTTGACAAATCTATTTTCCTCCTTTCAGATTGCTGCGTTCATTGCTGAGCCTGTGATGGGTGCTGGCGGTGTCATCCCTCTTCCAAAGACCTATTTTGATAAGGTTCAAGCAGTGGTTAAGAAGTATGACATCCTTTTCATAGTAGATGAGGTCATTACCGCATTTGGAAGGTTGGGAACCATGTTTGGATCTGATATGTATAATATCAAGCCAGATCTAGTCACCTTGGCCAAGGCTCTTTCATCTGCCTATGT

>Isogroup_25 CAGTGGGTAACATGTCACTGGTGATGATTTGCAAACTCAGTGTGTCATTGATCATCAAGCACTCCCCTGCCTGTTGAACCTCTTGACCACCAATCACAAGAAAAGCATCAAGAAAGAAGCATGCTGGACAATCTCAAACATCACAGCTGGTAACCGGGACCAGATTCAGGCTGTGATCAATGCAAACATAATTGGGCCTCTGGTGCATCTTCAAGGTGCTGAATTTGACATCAAGAAGGAAGCAGCTTGGGCAATTTCAAATGCCACTTCTGGTGGAACACATGATCAGATTAAGTTCCTTGTTAACCAGGGTTGCATCAAGCCACTCTGTGATCTGCTGATTTGCCCAGATCCCAGAATCGTAACAGTTTGCTTGGAAGGTCTTGAGAACATCTTGAAGGTTGGGGAGGCAGAAAAGAACCTTGGGGCTGGGGATGTCAACAACTATGCGCAGATGATTGATGATGCTGAGGGACTGGAGAAGATTGAGAACCTTCAGAGCCATGACAACACTGAGATATATGAGAAGGCGGTTAAGATGCTCGAGTCCTACTGGTTGGAGGAGGAAGATGAGGCCATGCCTTCAGGTGAGACCCCCCAGAACGGCTTCAACTTTGGAGATCAGCAGAACTCGGCTCCATCTGGCGGGTTCAACTTCGGCTGAAGATACCGGGGTGGACCGTTGGTACCTCGGCGCGA

>Isogroup_26 GAGCGGCCGCCCGGCAGGTACATCAACGGTGGTGGATGGCTCTTTCATGTCATCTCTTTCTTCTGATAATTCTGTTGAAGAAAGCAGCTTTCGACAACTTCAAGATGCAGTTAGTCAGCTGGATGTAAAAACCAAACTGTGCATTAGAGATGGTTTATACCGCCTGGCAAGAAGTGCGCAAAATAGACCAGTTTTTTCCAATGCGGTGAACAGCCATGGAGATAGCCAAGATGTAAAGGCTATGCAAAATACAGATACTTCGGGCAGGTTTGTTGATCGTAGGAGCATTGAGACGCAGACAAATCCAATTGATCGTTCTATAGCACTGCTGCTTTTTCATCAGCCATCGGACCAAGTGGCTGGAGCTGTTGACGATCCTCGGCACTAAAATCATGTTAGCAGATGTTCCCTTTTCTCCGTGATAGTAATTCATTTGCTATTATGACTAATGCAGCAACATAAATCAGGAGCAATTTGTGCCCTGCAGAATGAGAACCACCAGGCCTCTGCTAAAACAGCTAGATTGTGATAGGATGGCTGGTGGAGATTCAGTGATCGCATCTATTCTTATATTGTAGTCATGGTCGCTGTGAGTGCAGGGGAGCAATTGTAATCGCTATCCGCTTTGAGCTGGTTAAGCAGTTTACAGCTTCCAAATGTAGCATTACCGTTTTAGTTAGTCGGCTCAAGCAAGAAATATGTACCTCGGCCGCGACCACGCTAGTCGTGGTGTGGTCGGCG

>Isogroup_27 TGATGCACTCCCTGGGACACCCTCAGGCCACATTGGCACCACGACATAAACAGTAAACTTTTCCCCAGCTTCAATCTTGCTGACAATCTTCAACGAAAGCTCCTTAGGAATCACATGCAGGGCACCAATATCTTCAGGCTTGATATCTTCAGGTTTCCAGGCATAGGAACTACCAAGGAAGTATTGGTTCTCAATGTAGATGAAGTCCTTTGCCCGCCGGATGGCATTAATGTATGCATCCTGGATGCTCCTGTCAATGATTTGATCCTTTCCACTTACAAGCCCAGCTCTTGCAGCTTCCTCGGGGGTATCAGGGAAGCCAAATGCAGCACCACCATCAATAGATCTGAAGAGCTGAACATTCCATACATCTCTGTCCTCTGGGAACATGACAGGAGAAGGCGGGATAATTATGTCAGCAAGATCCCTGATCTGCACGAGAATATCTTTGCCACCCTGCTTTCTCCATCTCTGCTCGAAATTGTATAGGACATCCCAAGCAATTGGACCCTCCAGCCGTGAATGGATATCATGCCATGGCTCTCTCGGGCCACCCTTCGTGATGGATGCAGTCTCAAAGTTTGGCTGGTGGAAGTCATCATGGTGAACGGTGTCAAGTGTCCTAAACAAGGAATGGTACCTGCCCGGGCGGCCGCTCGAG

>Isogroup_28 CGCCTGCACCGTCTCAAATGAAACTAATGTTGCAGTCTACTGATGAATTTCCAATCAACGTGGGATTCACCGGAAAGGGAAATACTGCAAAACCTGATGGATTATCTGAGATCATTAGGGCAGGTGCAATGGGTTTGAAGCTGCATGAAGATTGGGGAAGCACTCCAGCTGCAATAGATAACTGCTTATCTGTTGCAGAAGCTTTTGATATCCAAGTCAATATCCACACAGATACCTTAAATGAAGCAGGTTGTGTGGAGCATTCAATTGCAGCTTTTAAAGATAGGACCATACATACATATCACAGCGAAGGTGCTGGTGGTGGTCATGCCCCAGATATCATTAAAGTATGTGGGGTAAAAAATGTGTTACCTTCTTCAACAAACCCAACCCGGCCATTTACTTCGAATACTGTTGATGAGCACCTTGATATGCTGATGGTTTGCCACCACCTTGATAAAAACATTCCGGAAGATGTAGCATTTGCTGAGTCAAGAATTCGAGCAGAAACAATTGCGGCTGAGGACATTTTGCATGACATGGGAGCGATCAGTATCATATCATCTGATTCACAGGCCATGGGTCGAATTGGAGAGGTGATAATCCGAACATGGCAAACTGCAAACAAGATGAAAGTCCAAAGAGGTAGGTTAGCTGGGTCTGGTGACTTTGATCCTGCCAAGGACAATGATAACTTCCGTATAAGAAGATACATAGCAAAG

>Isogroup_29 TTCATTGTGCACAAGATAAATCCCTGCCAACAGCTGACGAGCAATTCAATGCTAATTATCCATTCGACGAGCAAAAGAATGTTAATGTATATGGAGAACAGGCAAGTGAATTGATTATTCTTTTCTCTCACTTTGTTTTATTGGTTGGGGCAGTTCAAGCCTGTATCGTTTTCTTTTTGCTATCAATATTTTTATATACCATAGGCTTACCAAGGGGATGCAATGGAATTGTTGTCCACTGACCAGATATCCATTGGAGATGGAAATCTTGATATGATTGGAGAGCGATATTCATCAGAAAATTCCATGCAACAGTTTGAGGACAGGAAGTTTTCTATATCTTCAGGATCTCAATTAAGCTCCTCACATAATTTGTTGACGCAAAAGCTTCACTCGGATTCAACTTCACCAAGCAACATTACTTCTGAGTGCTATCCACATAGAAGTTGCCAGTTCAGTCCGTCTGGATCATTTGCACAGAGGAACTCCATGGTGCAACAACTAGGACAGCCCATAAATGATAAAGAACAACTTGGTCAACAAACATTGAGTAGAAGAGCTTTGTATCCTTGTGAGAACTATGAGGTTGGAAAAAAAGGGCTTAGGAAAAAGAAGTATGGGGGATCGTCAGGAAGCACTGGGTACCTCGGCCGCGACCACGCTAGTCGT

>Isogroup_30 AGAATGCAAGGAGGTGGTGAGAGAGTATGGAGAGATGATCCTTCAGATGCTTGTAGCACAGACAAGGCCACAGAAAGTGTGTAGTCAAATCGGTCTCTGCGTGTTTGATGGTAGTCACTCTGTCAGTAACCAAATTGAATCTGTTGTTGAGAAAGCGAAGAAGCGTGGTCGCGGCCGAGGTACTGTGTTGAAGGCGATCAACGCTTGCTCGTTTATGAATATATGCCATTGGGATCACTAGAAGACCATCTGCACGATCTACAGCCCAATCAAGAACCTCTAGATTGGACAACACGGATGAAGATAGCTGCTGGTGCGGCAGCTGGTTTGGAATACCTGCATGATAAGGCAAATCCACCAGTTATTTACCGAGATATTAAGCCATCGAACATTCTTCTTGGTGAAGGGTATCATGCAAAGTTGTCCGACTTTGGACTTGCTAAACTTGGTCCTGTGGGTGATAAGACTCATGTGACAACCCGAGTCATGGGGACTTATGGTTATTGTGCTCCTGAGTATGCCTCGACTGGACAGCTAACGATTAAATCAGACATCTATAGTTTTGGTGTGGTGTTCCTCGAGCTAATTACAGGACGAAGAGCACTTGACAGCAATCGGCCTCGTGAGGAACAAGACTTGGTCTCATGGGCTCGTCCATTATTCAAAGACCAAAGGAAATTTCCAAAGATGGCTGATCCTTTGTTGCATGGCCGCTTCCCAAAGAGGGGTTT

>Isogroup_31 AGAATGCAAGGAGGTGGTGAGAGAGTATGGAGAGATGATCCTTCAGATGCTTGTAGCACAGACAAGGCCACAGAAAGTGTGTAGTCAAATCGGTCTCTGCGTGTTTGATGGTAGTCACTCTGTCAGTAACCAAATTGAATCTGTTGTTGAGAAAGCGAAGCGGGGTTCTGATCTTCTCTGCACTGCCTGTGAGATGGCTGTTGTCTGGATACAGAATCAGCTTCGACAAAACAAGACGGAGGAGCTTATCTTGCAGTATGTGAATCAGCTCTGTGAGCGTCTACCGAGCCCCAACGGAGAATCTGCTGTCGATTGCCATCAGATCTCGGAGATGCCTAATCTTTCCTTCACCATTGCAAACAAGACTTTCACCTTAACGCCAGAGCA

>Isogroup_**32** GCGGCCGCCCGGGCAGGTACTGAAAATGAAAGGAGTGGTAGGACAAAGCAAAAAGAGGTCCAGCTATCTCGTGAAGTTATCAATATTTCACCTCTGTCAACTGCAATCATCTCTCATAGGTCAGGTGATGGCCTTGAGGGAAAGACTGGGTATGTTAGGTTAGCTGCCTTTTCTCAGACTGCTGCTGCTGAAATGGAAAGTGCCGTCAAAAGGATGGAGGATGAAGGTGTCCAGTCATATATTTTAGATCTGCGGAACAATCCAGGTGGTTTAGTAAAAGCTGGTCTTGATGTGGCCCAAATTTGGTTAGATGGAGATGAAACTCTAGTGAACACCGTTGATCGGGAGGGCAATGTGCGGGCAATAAATATGGTCCAAGGCCAATCTTTAACACATGATCCTCTTGTGGTGCTTGTAAACGAAGAAAGTGCCAGCGCAAGCGAGATCTTGGCCGGAGCGTTGCATGACAACGGCCGTGCGATTCTGGTAGGCCATAAAACATTCGGCAAGGGAAGAATACAGAGTGTAACGGAGCTGGATGACGGGTCTGCTCTGTTCATCACGGTCGCAAA

>Isogroup_**33** GCGGCCGCCCGGGCAGGTACTGAAAATGAAAGGAGTGGTAGGACAAAGCAAAAAGAGGTCCAGCTATCTCGTGAAGTTATCAATATTTCACCTCTGTCAACTGCAATCATCTCTCATAGGTCAGGTGATGGCCTTGAGGGAAAGACTGGGTATGTTAGGTTAGCTGCCTTTTCTCAGACTGCTGCTGCTGAAATGGAAAGTGCCGTCAAAAGGATGGAGGATGAAGGGTGGTTTAGTAAAAGCTGGTCTTGATGTGGCCCAAATTTGGTTAGATGGAGATGAAACTCTAGTGAACACCGTTGATCGGGAGGGCAATGTGCGGGCAATAAATATGGTCCAAGGCCAATCTTTAACACATGATCCTCTTGTGGTGCTTGTAAACGAAGAAAGTGCCAGCGCAAGCGAGATCTTGGCCGGAGCGTTGCATGACAACGGCCGTGCGATTCTGGTAGGCCATAAAACATTCGGCAAGGGAAGAATACAGAGTGTAACGGAGCTGGATGACGGGTCTGCTCTGTTCATCACGGTCGCAAA

>Isogroup_34 CATTACCATGTAACCTCCGAGGTAAGAAACATGCATATCCAGTTGGTGCGCTCCACACATTCAGGAAAATATGGTCCCTCGATTTTCCGAAAAGTCGAGGGTTGAGTGTGTCCTGAGAAACATGAAAATAGAAATGCACACTCCCTTCGCAGGCATTATCCTGATCAGGTTCAAAGGGACTTTCTCAGCCCGTATCACAACAATCCAGGCCAAGGGCCCTTCAGATTGTTGAGAATACAGGCACCCCTGGTGCAACTTTCATCACTAACAATCCCATAATGATACAAAAAGAGAAATATAAAAAACAGAGGACAATAGAGTTCTCTTTATTACAATCCATAACGACATTAAAACTGAAGCTAAGTGACACCAGCTTTATTGAGGCATATATCCTGCTTACCACCTCCTAACAAAGGATGGTAGAAGTGCTGCTGGCAAACATCCTAGCCATCAGACCTGCATCACCGGGAAGAGACGGTTCTCATGGAGATCTTATTTTTTAGCTAGGTAGCTTTCTGGAACATAGATTTCCCCGCCAGCTTCACCATGTTGTTCTCCACTAATTGTTTTCCTTGCAGCCAAGAATTCAGCACTCATATCACTCATTCCTTGTCTCACAGCTTCCTCTACTGTCCCGTAACCATGTTCATCAGCATACTTCCTAATATCTTCGGTGATTTTCATCGAACAAAACTTGGGACCACACATCGAGCAGAAATGTGCTACTTTGGCACCATCAGATGGTAATGTTTCATCATGGAAAGACATAGCAGTGACTGGATCCAGAGATAAAGCAAATTGGTCCAACCATCTAAACTCAAATCTTGCCTTGCTTAATGCATCATCCCATGCTTGTGCGTAGGGGTGACGCTTTGCCAAATCAGCAGCATGAGCAGCAATTTTATAGGATATCACACCTGTCTTAACATCATCACGGTTAGGCAACCCAAGATGCTCCTTTGGTGTTACATAACAAAGAAGTGCAGTGCCAAGAGCCCCAATGTTGGCAGCACCAATGGCTGAGGTGATGTGATCATAACCAGGTGCAATATCGGTTGTCAATGGACCCAATGTATAGAAAGGTGCTTCATTGCACCACTCCAGCTGTTTCTCCATGTTTTCAGGAATTTTATTCATCGGAATATGCCCTGGGCCTTCATTCATCACCTGCACATCTTTCTCCCATGCTCGGCGTGTTAGCTCCCCTTGAGTCAGCAGTTCTGCAAACTGAGCACTGTCATTCGCATCATAAATAGAACCAGGCCTCAAGCCGTCACCAATTGATAGTGCCACATCATACTGATTGCATATGTCAAGAATGTCATCCCAATGCTCATAAGCAAAGTTCTCCTTGTGATAAGTTAAGCACCATTTTGCATGGATCGATCCACCACGTGAAACTATGCCAGTCATTCTCTTTGCTGTAAGAGGAATATAACGAAGCAACACACCAGCATGGATTGTGAAATAATCAACACCCTGCTCAGCTTGTTCAATTAAAGTTTCCCTAAAGACTTCCCAGCTCAGATCTTCCGCAATACCATTAACTTTCTCAAGTGCTTGGTAAATAGGAACAGTCCCAATAGGAACTGAAGAGTTACGAATAATCCATTCCCGGGTCTCATGGATATGGCACCCTGTTGAAAGGTCCATGACAGTATCAGCTCCCCACATTGTGGCCCACTGGAGCTTGTGGACTTCCTCCTCAATGGAGCTCACAACAGCTGAGTTTCCAATGTTTGCATTCACCTTTACCAGGAAGTTTCTTCCAACAATCATGGGTTCCAATTCCAGGTGCCTCTTGTTTGAAGGAATAATGGCTCGTCCACGGGCGACCTCAGACCGGACAAATTCAGGAGTGAGGTTCTCACGTTTGGCACA

>Isogroup_35 ACTAATTACTAAGACTGAGGAAGTCGCAATTAATGCAAAAACAGCTAATTGGAAAGCAATAGTCATATTTCTAATCCTCCAAGCTACCATCAAATAAAGTTGACTACATATTTGATCCCTCACTTAACCAAAATTGTAAAAAAATACAAGAAGTAGGAGGGGTTTAATCATGAATCCATTGATTCTTCTCTTTAATTAAAACTTATTTTTACTTACCGCTTTTTTTATTTGGATATGGGGTGAGGAGAGGGGTTTAGTCTTTATTTCACAAATTTCACAAGCGGGTATAGCGGATCATGTATCCGTGTATACAGTATACAGAGATATGTCGAAAAGGGACTTGAATCTGAGATCTTTCTATAGGTTAGTAGAGCTTTTTATATGGCTATGTTCTATTTGTAGGAGTAAAATAGGGATTAGGTTGTGGAGAGATGGCTGAGTGGTTGATAGCTCCGGTCTTGAAAACCGGTATAGTTCTAGGAACTATCGAGGGTTCGAATCCCTCTCTCTCCTTTTGCTTATTGAATATGTTTGTTTCTTTAATTTTTTTTCTTTATTCTGTCCCTTATCTTTCTTTCATAAAAAGGAATGAATGGCTCGGCTAGATGGAATAACCGAGCCAGAACAGAAAAAAAAGAAAACATTAGAACAGGTATAAATAAGAAAATCTTAGTTAAGAGGGTTCATGTAAAGAACAGGTTCCAAATCACGATCGATTCCCTTTTCAAAACCTGCTGCAGCAGCTCGGGCTCTTCCTGCATGCCACAAATGGCCCACAAAAAAGAAGAATCCTAGAACAAAATGAGAAGTCGATAACCAACTTCTAGGAGAGACATAATTAACTGCATTGATCTCGGTAGCTACGCCACCCACAGAATTTAAAGAGCCTAAAGGCGCGTGGGTCATATATTCTGCTGAACGTCGTTCTTGCCAAGGTTGTATGTCTTTTTTTCAACCTACTCAAGTCCAAACCGTTGGGGCCCTTAGAGGTTCTAACCATGGAGCGCGAAGGTCCCAAAAACGCATAGTTTCCCCTCCAAAGATAACCTCCCCAGTTGGCGAACGCATTAGATATTTACCTAAACCTGTGGGTCCTTGAGCGGATCCGACATTAGCTCCAAGACGCTGGTCTCTAACTAGAAAAGTAAATGCTTGAGCTTGAGAAGCTTCTGGCCCGGTGGGTCCATAAAACTCACTCGGATAAGCCGTATTATTGAACCATACAAAACAACAAGCGATAAAACCAAAGAGAGATAAAGCAGCTAAACTATAAGACAGGTAAGCTTCCCCAGACCATACAAACGCACGGCGAGCCCATGCGAAGGGTTTGGTTAAGATATGCCAAATTCCGCCAAATACACAAATGAAGCCCAACCATATATGCCCACCAATTATATCTTCTAAATCATCCACACTAACAATCCACCCTTCTCCCCCAAAAGGGGATTTTAGTAAATAACCAAATATAACACTGGGGCTAAGGGTCAAATTGGTAATTTTTCTTACATCTCCCCCCCAGGGGCCCAGGTATCATATACACCGCCAAAATAAAGAGCCTTGA

>Isogroup_36 AACCGGCTTCTCTGAGCACTGATTCGCTCAACACCCTGGACTAATTATTATTTTCCCCAAAAAAATACGAGTGAGGAAAAAAAATCGTTACTGTGCTACAGTCAAGTCTCGCACATGAACTCATGTTACAGTGTCACACTCGTCGTTCACCGGCGAAGCAGACATCAAGAACACCTCTGATCCCAGGTCCAGATTTAACATCCAACTATGGCAGGAATGGGCAGTCCTCACAAGCACTTCTTGACGTCTGAAAATAGCGCCTTCTCTTTATTCGCTGCCTCGACATCATCAGGTGATGCAACAGCGACCACGACTCCATTGTCGTTGATGGTTTCGACAGCATCAGCAAACTCCTTGAAATCCTTCACGCTGGTTGCGAGTATTTCTTCACGTCTTTGCTGACGTTCCTCCTCAGTGATACCCAACAAATAACGCATTAGACTGCTGTAACCTTTAGCATCTGGTAGCTGGTAGGAATCAACATCACCTATGGTGCCAATAATAGCTTTTGTGAGAGCATCATCATCTATCTCTAGTTCTCTGAGGAAGTTGGCAGTCCCATCATAGATCTCCAGTGTTTTCAATAAGTTCGGGTCACGGTATGACAAATAGGAGAACACGCCTGAATGAGTGTCAAAGTCACAAAACCCTCCATATGCTCCACCACTAACTCGAACACGGTCCCATAACCATGTATTGCTTATGTGCTTGGATATGACATACGCGCTTCCATTGAGCTGATATCCACCTTGGTATAGGTTTCCAGCTTTTCCAACATAATTAACCTGAGTAGGGATAACAATGGCTTCATTAACAGAAGGCAGTCGAGAGAGCCATGGATCACTTCCCAGGGATGGAGCACTTGGTAGTGAATCAAGAAATTTGGCAATATATTGACCAGATTTTTCAAGATTTTTTGAGTCACTTGTTATGTTAATTAAACAACCTTCCTTAGAAAATAAAGACGTCCTCATTTCCTCGAGTGAAGAAGATATTGTGTCCCAATCTTGATCAATCTTTGTTTCCAGGTCTCGTAAATACTCAAGATAACTAACACCACCCATTTGTTCACCAATCCATCCAGCTGCATTTAACTTAGCATCCATTCTAGCAGCTGCTATACCGTGGCCACTACCCCTCAATCGATTCTCCATCCTTGCTTTACTTTGGGAAACAAACTGTTTGAACCTCTGTTGTTCTGTGAACTGAACATCCTGAAGAAGGCAGTTCATCAGATGAAACAAATCTTCTACTCGCGTTGACATTGCCTTTCCCCGAACAACAATACGAGTAAGAGGCTCTTCTTTTCCCTTTATTGAAGATGTTAAAGGGTAAACTGATATGCCTCCAGTTTTTCTTCCAACTAACTGATTAAGCTGCACGAAGTCCATGTCTTTTGTGCCCATCTCCAGTAAGGATTGGCAGAACAAAA

>Isogroup_37 TTGGTTCCGTTTTGACCCCCTTTGTCTCGTGCACGTTCTGCATTGTAAGATTTGGTCCGGTTCGTCTGGGATAGTTCTTCAGGACCTCTATAGGACCGAGCTTGTTCTTGTGGCTGAACTCAATTAAGTCCTCCATGCGATTCACCACCTCTGATATCTGCAAGCAGCGAACATATCTTTTAGAGAGGCCATGTTCATTTAAGCTGTGGTGGTCTAGATATTTCGCTAGTTGCCGTGATGCTGACACAAACAAGTCGCAAATGGCTTGTGCATCATTGGTCGACACCCCAGCAGGCCCACTTTCATTGTTAGCAGCTTGATACTTCTGGGCAACTTGCAGCATATGATTAACCTGTGGTGTTAGAAACCTGCGAGTGATATACTCGTCATGTCGTCGTGAACAGAACTCCCAAGACATAATCTTTAATTCCGGGGTGAATATTATTCTCAGTTGCCCCTCGTGTGTAACATGTAGATGATCATAGATGCTCTTCTGAACAACTTTAGTATGCTCAAGGAGAAGCAATCCATTGGGCAACCGGAATTCATTGGGCATGTCCAGGAATAGATATTCATCTATAACACCATGGTCAAATCGAATCTGACAGAGTCTAGGAAGTATTTCATAGGTAGCCTCATGTCCTTTCCCTGCATGTGTATTGCAAATATCACAACGCCATGTATCCTGAGCTGTCTGCTGAATAGATACCGGAGAGTTCCCTCTTTTTTCATATGATGACACGCACCATCTTTCTCTTGCTCGTGGTGCAAAATACTCATCAATAAGCTTCCTCCAGTATGTTATGGGGTTATCCTCTGGCCGGTGGCGCTTATGATACAAATACTGCATTAACCTTCTAGAACAAAGCCCGCTCTCAACAGGAGTCCTAACAGGTCCAGCTAGCTGCATTCCAGGTTGTGCTAGCGGCAGCCTCAACTGCGGCTGCCGTGGTATGCCAATTTGTGATTGCTGAATTTGAGGAAATTGTTGTGACAACTGCTGCTGCTGTAGATGCTGAATATGTGCCAGTTTCTGCTGCCGGATCAAAGCTTGAAGCTGTGGGTTATGTTGGCCCTGGAGATGAAGAGAACTTTGACCTTGGAGCAGCTGCTGAATCAAGTGCTGTTGCAGGATATCATCTTGCCTAATATCAACCCTTGGCTTCTTCTGAGCATGAAATGCAGCAGCAGCCTCAGTCCCAGGTTGAGATGTTACACTAGATGATCTCCGCTTCTGCATCTGCTCTTGGGGTGGACTTTGCTGCATGATGGATCCATCAATGACTGACGAGCCAGATATGTTATTGGATGAAAATGACATCGGGGAGGCAGGAAGGACCTCGGCCGCGACCACGCTAGTCGTGGTGTGGTCGGC

>Isogroup_38 TTGAAACCACAGCACCCAAGCTCTACAGTATCAATGATGGTCAATTAAAGTTGGAAGACGTCGCTCTTACAAAAGCAGTCCTTGAGAACACAAGATGTTTCTTACTTGACTGTGGGGCTGAAATGTTTGTATGGGTTGGTCGTGTCACACAATTGGAGGATAGAAAAGCTACCACTAAAGCTGTTGAGGAATTCATCATTAGTCAGAAAAGGCCAAAGACAACAAGAGTGACTCAAGTGATTCAAGGTTATGAGAGTCATGCATTCAAGTCGAAATTTGAATCATGGCCAGCTGGCAATGTAACAGGGAGCTCAGGCGCAGAGGATGGGCGTGGAAAAGTTGCAGCTTTATTGAAGCAACAAGGTGTCGATGTAAAGGGAGCTCCAAAAAGCAGCACTCCAGTAAACGAGGAAGTTCCTCCTTTGCTTGAAGGCGGTGGAAAACTTGAGGTGTGGTGTGTTAATGGAAATGCTAAGACCGCACTGCCAAAACAGGACAATGGAAAATTTTACAGCGGAGACTGTTATATCATTCTTTATACTTATCATTCAGGTGACAAGAAAGAAGAATATTATCTCAACTACTGGATTGGGAAGGATAGCACAGCGGATGATCAACTGATGGCACTTGAATTAGCCAATACAATGTGGAATTCACTGAAAGGAAGGCCTGTGCTGGGTCGTATTTATCAAGGGAAGGAACCACCACAATTTGTTGCTCTTTTCCAGCCCATGGTTATCCTGAAGGGTGGAATCAGTTCTGGATACAAGAAGATTGCAGATGAAAAGGGCGTGGGCAGTGGAACTTATTCTGCTGAAGGCATAGCTCTAATTCGTGTATCCGGAACAGCAATCCATAGTAATAAAACGCTTCAAGTTGAGGCGCTAGCTACATCTTTAAGCTCTACAGACTGTTTTGTATTACAGTCTGGAAATGCTATGTATACGTGGCATGGTAATTCTAGCACTTATGAGCAACAGCAGTGGGCAGCAAAAGTTGCTGAGTTCTTAAAGCCAGGTGTAGCAGTGAAACACTGCAAGGAGGGAACAGAGAGTTCCGCTTTCTGGTTTGCTCTTGATGGAAAACAGAGCTATACAAACAAAAATCCCGCACAGGATGTTATTGTTAGAGAGCCTCATTTGTATGCCTTCTCATCTAAAAAAGGGAGACTGGAGGTTACTGAGATCTTCAACTTTTCCCAAGATGACATGTTGACTGAAGACATGATGATTCTTGATACACATGGTGAGGTCTTTATTTGGGTTGGTTAGTGCGTGGAATCAAAGGAGAAACTTAAGGCATTTGATATTGGCCAGAAATACATAGAGCATGCTATGTCTATTGAAGATCTTTCCCCATAT

>Isogroup_39 CATTGATATGGATAGGACAACATAGGAAAGGGAATATAAACAATGCACTACATGTGAAGCTGAATGACTGGGAGAACTGCTAAACCAGCAAACCTCGAGCAAAACAAGATGGATTGTATACTTGCATAGTAGACAGCTTTGCCTCACAGTATGCCGCTCAAGAAACCCCAGAATCCAGAGGACGCATTTTCCTTTTTTGGTCCTTTGAAGGCATTGGTGATGTCATCAGCCGTTGGATTCTCCGTAAATTCAGCTAGCAAATCATACTTCTCTTTTCCATTAGGTTCATCAACCCTTCTATAAATCTCATATTTATTTGGGTAAGCCATCCTTAAAGCGCCCATGATAGGATAAAATGTGCCAGCATAGAAGAGAAGCCGGAAAGAATAACAAGTCTCAAAGGATGCAGCATACTTGAGCCTCAAGTCTCTTCCCATTGTTTGCATTACGCCACTTGATGCAGGCATATCCTTTAGGCGAGGATTTACAAGAATCACAGGTCTGTCACCAGCAGCATCAGTCATAGCTTTCATATCATCAATTATGCAGTTTCCAACCGCATTCTGGGGAGCAATGAGGACAAACATGTCATCTTCTTTATCGACCTCACTAGCACCTACTGCTCCAATATTAATGAAGGCACCCTTCGCCCCATAGTCACCCCAATCCATGATTTCCAAAATCTTTCTGGTTCCAGCAAGCTGAAGTGGAATACCAGCAAAAGCACCTTGCCCCATGGAGCCTTGAACACATACCTTGACACGTTTTCCGTCATTGGCAAAGGTAAGAGATAAATCTCTTACAAGTTCCATGAGGGTTCCTATACGATAGACATCCATTTCAGGGTTAAGTTCCGGAATGTTGATATCTATCTGCAACCGGGTCTTTTTATCTTCAATAGCACGCTTTGTAGCTTGTAGCACATCGTCATAGAAATATTTCCTGATCTCTCTGGAGTGTGGAATGTTATTTAATCTCGTCGGAATTCCACGATACTCATTTTTTGAAGCTGTTGTATGCACAGAATCTGGTGGTTTTCCGTTTGTTTTGGCCAACTTGTCTTCTGAAGCATTTGCACATATCTTCTGTAACAGGGAATCTATTTCATGAAAAACTATTTCCACTTGGCGATTTCCATCAATCTGGTTAAGCAAATCTGAGTATGTCGGAAGAACAGCTTCTGAATTTTGTTTGTAAGTCTCAAGACGTGATTTAACCTTCTCAAATGTATCATCGGAGCGTGTAATAAGCCTGTCAGAAATTTCCTCATTCTCTGGGGGGAAGTTCTTTACATGATAAATTTTTCCTGTTACAGGATCCAGTCTTCTTCCAACACATCTATCGATTAGATTGTCA

>Isogroup_40 TTTGAGTGAAGGCGGCTGTGTTGGTAACTTCTCTAGGGAACAGGTTGAACTCTTCTGTGTGAACAATCTCATGAACTGTTCATTGGAATCCAATGAGGAGTTTATCTTTATGGAATTTAGGTTTGCTTTAAGAGATAATGGCATGCGTGCAGCTTTCCAGCTCCTACATATGGTCCTTGAGCATAATGTGTGGCTAGAAGATGCATTCGATAGAGCAGCTCAGTTATATTTGTCTTACTACCGCTCAATCCCCAAAAGTTTGGAGCGTGCCACGGCTCACAAGCTTATGGTAGCCATGTTGAACAACGATGAAAGGTTTGTAGAGCCATCTCCACATTCATTGCAGAAGTTGACTCTTCAATCAGTTAAGGAAGCCGTCATGAACCAGTTCGTGGGTAGCAACATGGAGGTCAGTGTAGTTGGTGATTTCACAGAAGAAGAGGTAGAATCTTGTGTTCTTGATTATCTTGGGACTGTCAGGGCCTCAAATTCTTCTAACACAGCGGAGCATATTGAGAAGATATCATTCCTACCGTTTCCTTCGGATCTGCATTTCCAGCAAGTATACATAAAGGACACAGATGAGAGAGCTTGTGCATATATTGCTGGCCCAGCACCTAACAGATGGGGGTTTGCTACTGAAGGAAAGGACCTGTTTAATGTCATTCGGAGTTCTAGCACAGATGAAATCTCTGAATCAGCAAGCACGGATATAGTGGGGAAGACACATATTAATGTCCGCAACCATTCTCTTTTCTTTGGCATCACTTTGAGTTTGCTGGCTGAAATTATCAATTCCAGGCTATTTACGACAGTTCGAGATTCCATGGGATTAACCTATGATGTTTCTTTCGAATTAAATCTTTTTGACAAACTGGATCTTGGTTGGTATGTGATCGCGGTAACTTCAACTCCGAGCAAGGTCCATAAAGCTGTCGATGCATGCAAAGGTGTTCTCAGAGGTTTGCATAGTAACAAAATTGTTGAAAGAGAGCTGGACCGGGCAAAGAGGACACTGCTAATGAAACATGAGGCTGAGACAAAGACAAATGCCTATTGGCTTGGTTTGCTAGCCCATCTGCAATCTTCGTCCGTGCCTAGAAAGGATATATCCTGTATTAAGGAATTGACAACACTGTATGAAAGTGCCACAATTGAGGACTTGTATCTTGCGTATGAGCACTTGAAAGTTGACGATTCATCTTTATTTTCCTGCATCGGTATTGCTGGTGCCGAGTCTGGTGAAGACATGAATGATGATGAGCCTGATATGGGGCTTCCTGGTATGGTTCCCATGGGAGGCCGGGGTCTATCAACTATGACCAGACCGACCACATGAGCT

>Isogroup_41 AAGGTTACAAATTATCCATTTCCTGCTCAGTTGCAGCGTTTGGTCTTCTATAATGCTCCTACTACGTTCAGCTCCTTGGCCATTGCTGGTGTGCGTGGCCGTTGGTCGTGTCGATGATATGCAGGTTGTAGCATCACGGCATACCAGGGGCATGCCTGGAACCAGCGTAGCACTTTGCCGCCAGGGGTGCATGGAGCTGCGAATGGCGCCTTCAATAAGAGACCATGGCACCTCAATTCCACCTCTTTGCGTTTTTCATCGATTTCGATTTCTGCTTGCTTTGTTGGAAGGCCTGCACCTTTTGTTTCTTCCTTGCGATCTTCTCCGCCTTGGACATTTTTGACTTGGCCTTTACTTTGGGAGCTTTTTCCTCAAGCTCTTCTGCACGTGCAAGTTCCCTCTCTCTGGCCTCAAGGTTCCTTTCAAGGTAATAATTGTAATCTCCTTGATAATCCTGGATAGTTTGATCTCTCACTTCGATGACTCTATTAACTATTTGTTTTACGAAATACCGATCATGAGACACTGTTATCACGGTGCCAGTGTATTCTGATATTGCTTCCTCAAGCATTTCTTTTGATGGAATATCGAGGTGATTTGTTGGTTCATCCAAGATCAGTAAAGTAGACGGAGTCACCATGAACTTGCAAAAGGAAAGCCTTGCCTTCTCTCCACCACTTAAAAACCGAACCTTCCTATCTAGCATGTCATCCCTAAAGTTACAACGACCAAGGAGACCTTTGATGTCATCGATTTTCCAGTCCTCTGCAGCGTCAGCCACAGTATCCAGCACGGTTTTATCTAAATCAAGAGCTTCTGCCTGATTCTGCTCAAAATAGTTTGGCAGCACATTATGCTCCCCAAGAATCACTTCACCTTCTTGTGGTTTCTCCATTCCCAAAGCAAGTTTCAGTAATGTGCTCTTTCCACATCCATTGGGGCCAATGATGGCTATTTTTTCTCCTCTCTCAACTATCAGGTTAGCATTGTTGAACAATGTCTTATCCCCAAATCCAAATTTGAGATTGTTTATTGCTAACACAGTTCTGCCGCTTCTCCCACGCTCAGGAAACCTGATCTTTAACTGCTTTCTTTGGAAAGGTTTCTCTATCAACCCTTCTTTCTCAAGCTTTTCCAATTTCTTCTGCTCACTTGAAGCACGCCCTGAGCTAGCTCCAGCCCCAAGCCGAGCTATCAGATCCTTTGTGTGTTCAATCTCCTTCTGCTGCTTCTCCCATGCCGCGCGCTGAGTTTCCACCCATATCGCCTTTTCTAGAACATATTCTGAATAGTTACCCTTATACGTTTTTGAGACGCCAAATTCAGTCTCTACTATCTTCG

>Isogroup_42 AGAACTTGAAGCAATGGCAGCCAGCACCATTGCTCAAAGAGGTGTTCAACTTGTTGGAACTGCTCATGGGGTGACAATTGACAGCATAATTAAAAACCCTTGCTTGCAAATGCTTGTTGGTGGGATTGAGAGTGTGACTCTTGGAGATGAAGAAGCAAAGAAGCGCAAAGTTCAGAAGACAATTCTTGAGAGAAAAGGGCCCCCAACATTTTCATGTGCTGTTGAGATGGTATCGAAGACTGAATGTCGAGTGCATCACAAGTTAGAAACTACAGTCGATGCTATTCTTGCAGGTAAGCCTCCTAAATTTGAAGCTCGCATGATGGGTAGCAATGCAGCCGAATCGGAAGGATCTTTGGTGATACCTGAGAAAGAGAGTAAAACGGAACACTTGCCTTTGTATCTGCACCAACTGGTCACCACGGCAATCACATCAGAAGATAAATTCGTTGATGATGTTGGTTCCTCCAGGCAAACAATAAGCAAGGGCATCCCATCAGATGAAAATGTCAATGCTGATTTTGGTTCTAGAAAGAAAACAAAAGGCAAAACATATGTGGCTGGAACGGCTCCAGTCCGTGTATACACATACCAGGTTTCAGAAGCTGATATCTTGCAAGTAGCAACAGTGATGGGTTTTGAAGATGAATTAGACATAACAGATGACATTGGAGCGGCCAATGTGATTCTTGCATCAAGATCTGAAATGAAGCTGAATCCATGGATCCGTAATGTTGCCAAATACCACAAACTTCCCATATTTGTCGTTAAGACAAATACGATGGCTCAGATAGTAAAGGCTGTCAAAATGATTGTTGGAAAAGACAAACTTAATGCACCATCACGCAAGCAACCTAAGGTTTTGGAAGGAGAGATAGAGATTCAAGATGATGCTCCAAAACGGAAGCCATCACTAGAGGAAATTGATGCGTTGGAGGAGGCCCGGCTGGCAATTGAATATATTGTTATTCCAGGCGGGGAGCCCGTTGAACTCCTCCCAAGGTGTTCAGAAATAGTTGCTCGTCAGCTTGAGCTTGTTGAGAGCTACCAGCTACTTGCCGAAACCTTCGGAACCGACCCCAATTCGAGGTTGCAAATTCTTCCCGTGAAAATAACCAAGAAGAGTTCAAATCAGGGCGCTCAGGGTCCGAAGTCCACCAAGAAAAATGGCTCGGATCTGATCGTCAGTGAGAATGGTGGCGGCTTCAGCTTCTCTCGGCTACCCTTCCTACCCAAGTGACATCCTAGTTTGACATTCTGATCTCTTCTCTCTTCCTTCTTGGTGTCGTTGTT

>Isogroup_**43** AAGATTTTCGTGCTGTGCCATGGACAGTGCTTCAACCTCTGCACTGAAATCCCTTTCCATCAGACATGTTTCACCGTTGAGCTTTTTGACTGCCAGCTTGGAGCCATTATGTAGCTCTGCCTTGTAGACTAACCCATAACCTCCACATCCAATGACGTTCGCCCTGTCAAAGTTATTTGTAGCTTTCAAGATGTCAGCAACTTTGAGCTCGTTTTCTTCTCCCTTGCCTTGTGGCATCCGCATCACCACTAATGTTTGCTCTGAACTGGAGTAGAATGAAGTTTCTTCAACATCTCCGTTATTATCTCTTCTATTTATTGTTGTAATGCCCTTCATGCTGATTGGAACAAGGAGGCGCCCCAGCAACAAAAGAACGGCGATGCCTCCAAAGAACACGCCAAATGCGATAGCAAAAACAGCCTTCTTATCTCGTTGTTTTGTGGAGGATGGAGCTATTGAATCTGAACCACATTTGTGAGTAAGAATAGAGCCACACAGCTCTGGATTCCCATCAAAACTGGAATTCTGAAATGTATTGAACTGGCCTCCAGATGGAATAGGCCCTTCTAGGTCATTGTTTGAAACATTGAATGCTGAAAGGAAATGCAGGTTGTTCAATGCAGCTGGGATAGCACCTGTGAGATTGTTGCTGGACAAGTCTAGCACCTGCAGTTTTGTGAGATTGCATATTGATTCTGGTATCTCTCCTGTCAAGTCATTGAAGCTGAAATTAACTGAAATGAGGGCTTTCAACTGACCAATCTCCAGGGGTATCTCACCAGTGAATTTATTGTTGCTTAGATCCAGCAATTTAGCAAAATCAGTTGGCACGAGGTATTGAAGTGACGGACCATTGTAAACAGGAAGTTCGAAGACCGTTGGGTCTAAATGTGCTTTAGTCTTCTCTGGCTTTAGCATTAGCATATCCATCAGTGCTTTTGGAATTTCTCCTGTAAGATTATTGTTTGATATATCTAGATAGAAGAGGTATTTAAGGGTTTCTATCCATGCTGGTATTGATCCACTGAGTTGATTACCAGATAAGAATAACATCTCTAAATTTCCTAGCTCTGATATCCAAAGAGGTATTTTCCCAAACAGTGTGCAATCTGAGATGTCCAAAACCTGAAGTTTCCCATAACCACTAATCTTGTCATCCTCTAGCAGGAGCTCTCTCTTGAAGTTCTGCCCGATAAACATGGTTGTAAGGTTTCTGCAGCGCTGAAGGATCCGAAGTGCATTCGTGACATTTTTGAAAGAATTTTTAGCAAGTGAGAGGAAGGTGAG

>Isogroup_44 TTGAACCTCGGATCAAGTATGACGGGAATGCAAGCTGACTTGTATGTCTCTATCCAATACTGATTGAACTTGATTTGCATTTGTTTTATCATACGTGCAATTGTTAGATCCTGGGTTGATGGTTGTCCATTCAATAGCTTTCTATTCAGATTAGATTCCTCTTTATTCAACATCTTCTTAATTTTCCAAAACTGGTAGAAATGAAGATGAGAGGTGGGATACTTCGAGCCTGAAAGAATCATTGTTGCTGTGTAGAAAACCTGCAACAACTTGCATATAGCTTCAGCCATTTTCCATTCAGAGGGTGAAGGGGCAAATGTATAATCCTTATCCTATTTTTCTAGGGAATAGAATGCATTTCTAAGAGGAAATGCAGCTTCAAGCATCAAATAAGTAGAATTCCAGCGAGTAGTCACATCAACAGAAGGACTTTTATATTTAGCAGTCAGTCCTTCTTGTATGATTATCTCAAGAAACTTCTCAACCCTGCCTTGTGAACTTTTGACAAACTTGGCACTTTTCCTAATGTTATTGACTACGGGTTTTATTTCCCGTAAACCATCTTGGACAATTATGTTGAGAACATGTGTTCTACAGCGGACATGAAGTAACTTTCCTTCTATTGGTAAAACCCCCTTGTTCTTCAAGTTCTCTCTCAACAGATTGACCATTGCGGTATTGACTGAGGCATTGTCCAATGTAAAGCTAAACAACTTATCCTCTAAGTTCCATTCCTGAATGGTCTTTAGCACACCTGCCCGGGCGGCCGCCCGGGCAGGTACCGTTCCGTGAAAGGAGCTAAAGTTATTATTGATGCAAACACAGGCCGTTCTAGGGGCTATGGATTTGTTAGATTCGGAGATGATAAGGACAAATCTCATTCAATGACTGAAATGAACGGCGTATATTGCTCTACCAGGCCGATCCGTGTTGGACCGGCAACACCTAGAAGAACTGGTGATTCTGGCTCCTCAGCACCTGGGCATTCAGATGGTGATTCTACTAACAGAACGGTTTATGTTGGCGGGCTCGACCCAAATGTTAGTGAAGATGAACTGAGGAAAGCATTTACCAAATATGGTGATGTTGCCTCTGTCAAAATTCCTCTAGGGAAGCAATGCGGCTTTGTTCAGTATGTCAACAGAATCGATGCGGAAGAAGCACTACAAGGATTGAATGGGTCCGTCATTGGAAAGCAGACAGTTCGCCTTTCATGGGGCCGCAGCCCATCACACAAACAGTCAAGGGGTGATTCCGGCAACAGGAGGAACAACAT

>Isogroup_45 AGCGGCCGCCCGGCGAGGTACAAGTTACAGATGTCTGTGCCACCAGCAAGATCAATCGACAGCGGCACTAGGGAATCCACCTGGAGAAGCCAATGCGGCATGCCATGGCACAAAATATAGCATTCTTAAATAGCAGTAGCTTGTTAGGCACACGCGCCAGATGATGCCGAGCCCAGCATGCAGAGCCGATGATCAAGCCAAGTATCGGTAGACATTCGGGTTCTCCTTGTCCCTCTCCAGGTAATCCCTGGTTATAAGATCCTCAATGCGCTTCTTTATTGCTTTGAAGTCAGGCTTGAACATGCGACCAAGCTGCTCCACACATTCCATTACTAGTGTCTGGTGACCCAAGACTTTACGGCTCTTCATAATACGCACAATGGATGCATCAATCGCATATCTTCGATCCTTGTCAACATCTTCGACTACCTTCTTTTTCTCATCAACTGGTGGTAGAGGTATCTTAATTCTTCTCATCTTGTCAGTAAATTTTGAGTTGAACTCAAATACGTCATTAGGAGAAATAGTTCTGTTACTTGGTTCTTTACTAAGAATCTTGTATTTCGCACAAGAGAGAGAGTGAAGCAGCCTCACTACATCGTCATCTGACAGGTTCAGTTGTGTTACTATCTCGGAGTAACTAAGTCTATCAGCTCCATTGAACAGTAGCAACAAAGCAGCCTGGTAAGTTGTAACAATGAGCTCAATAGTCTTGGCCTCAAACTTTGCAGTGATGTGGCAGATACCCAAGGAATATATCCATGTAAGTTTTCTATGCTTAGTTCTTGTTTGATAGAACTCTTTGAAAACCTCGACACATCTCACCATTTCAGCAGGAAGGTTTATGTCAAACGATTTATAAGTTGGCCAGAATCCTGTTGTCAGAACAGTGACCGCCAAATCTATCCCAGGATTCAATTCAGAGTGTGTGCTTATAAACTCTTCAAACTTGGTTTGATGATCTCTTGCAACAGTAAGATCGGTGACCATGCCCTCCATTTTTGAAGTAAATTGGCCACCACACTGTTGTTTTAGCTTGGTCAGAATGCTTCTTTCATGCTCATCATTAGCGCTCTTGTCAAATAGCAATCTTCTTGCCAGCTTCTTCCTGTAGAACTCAGCAAAAAGGTCCTTATCACTGATGTAGGCCAGTAATCTCACAACCTTCTCCAGGGTATCTTCAATAGCTTCATCACTGAGTTTCTCACTCCCGCCTTTCTTCAAAATATTATCACAGAATGTAGCAAGTAATTCAGCACTT

>Isogroup_46 ATATATTTCAGGTAAAGTTTTTTTTTCTACAAATATAAATCGATAATCTCGACTAGGCAGATAAGCCTAAAGTAGTATACATGGTATAAACAAGTAGGCACTTTCTTACACCTCAATTAGTGTATGCTTACAATGAATTGAAAAATCGAACACAGAATCAGTCACAACAAGATATGTTCTTCAGGCACCCTATGCTAGAGACGTGGCTTCCTGCATCTATCCTCAAGCAGTGGAATGGAATCTACAAATTCTGCCAAGACTGAGATAGTAACAAATTCGATAGCCAATGTCTTTCAGTATCTGATCCCTTTTGCGACCTCGAAGAAATGGGCAACATTTTCCTCAGGAGTCCCCACCTTAATGCCATGACCAAGGTTCAATACATGTCCTTCACTACCAGCCTTCTGCACAGTGTCATAAATCCTCTTGGTTATGAACTCTTTCGATCCGAAAAGAACACCAGGGTCCACATTTCCTTGAACTGCTATGTTGGATCCCAATCTTTTCCGGCCCTCAGCCATATCAACCGTCCAGTCCAAACTAACAACATCAACACCTGTCAATGGTAGCCTTTCCAGTAATCCACCAGATCCACTTGCATATAATATCAGAGGCAAGTCAGGATGTGTTTCTTTAACAGAATCCACAATTTGTTTCAGATATGGCAAGCTGAATTCTTCAAAATCAACTGGGCTGAGTTCTGTAGCCCACGAGTCAAAAATTTGGACTGCTTGTGCGCCATTGTCCGCTTGGTATTTAATGTAGTTAGCCATGGAAGTTGTAAATTTCTGTAGCAGACTATGTAGTATCTCTGGCTCAGCGAAGGCCATTTTCTTAATCTTCGAGAAGTTCTTTGATGAACCTCCTTCCACGCAATAAGATGCCAAGGTAAAGGGAGCTCCAACAAACCCGAGCACAGCAGCTTCCTTCTTAACCTCTTCTCGCAGTATACTTAAAGCCTGTCCTACATAAGGGACCCACTCTCCTGGAACAAATTCCCTGACTTCATTCACAGCCGCTGCTGTTCTCAAGGGATCATATATAACTGGACCCTTTCCTTTCACAATGTCAAAAGGTATGTTCATCCCAGGAAGTGGAGTAAGGATATCAGAGAACAAGATAACTCCATCAGGCTTGAAAACTTTCCATGGTTGCAGAGAGATCTCAACAACAAGGTCAACATTTTCTGATCTTTCACGGAACAAAGGATATCGCTCACAGAGATTTTGATAGCTCTTCAT

>Isogroup_47 CATCGACATGATTGCAAATCCCGAGGTTGCTGATGTGTTCCGAACAAGAGCAAAGGTTGTTTCTGAAATCCGCAAGACAATGGAATCATTTGGTTTCATTGAAGTTGAAACACCAGTTCTACAGGGAGCAGCAGGTGGTGCTGAAGCAAGGCCTTTTATAACTCACCATAACTCGCTCCAAAGGGATCTTTACTTGAGGATTGCAACAGAGCTACATTTGAAAAGAATGCTGGTTGGAGGGCTCGAGAAAGTTTATGAGATTGGAAGAATATTCCGCAATGAAGGCATCTCTACTCGTCATAATCCTGAATTTACCACTATTGAAATGTATGAAGCATATTCAGACTATGAAAGCATGATGAATTTGGCTGAAGAAATCGTAACTCGGTGTGCTATGGTTACTATTGGAAAGCTTAAGGTCGATTACCAGGGAACTGAAATCTCTCTTGAAAGACCATGGAGGAGGGAAACCATGCATCGTCTTGTTGAAGAAGCAACTGGCGTTGACTTCAATAGCTTCGGAGATATTGAATCCGCTAAAAATGCCGCAACAGGGCTGCTGGGGATCAAAACAGATAGCCGTGAGAATACTTCATTGCAAGCTTGTTCATCTGTTGGACATGTTCTCAACGAGGTCTTTGAGACTGTCGTTGAACCAACTCTTGTGCAACCAACATTTGTTCTTGACTACCCAGTTGAGATATCACCGTTGGCAAAACCACATCGGAGGCATGCTGGTCTTACGGAACGATTTGAACTTTTCATATGTGGCCGTGAAATTGGAAATGCATTTTCTGAGCTCACAGACCCGATTGACCAGAGGAGTCGTTTTGAAAATCAAATAAAACAGCACAATGCTAAACGTGCTGCAATGGGTAAGAAGGTTAAATCTGCCGAGAGTAAGGGGGATGATGATGATGAGTATTCGTATGAAGTTTCCTTGGATGAAGACTTCTTGACTTCTTTGGAATACGGAATGCCACCTGCTTCTGGAATGGGCCTTGGGATCGACCGGTTAGTAATGCTTTTGACAAACTCTGCCAGCATAAGGGATGTTATTGCTTTTCCTGTCCTGAAGCTTCAGCAATAGGAGGACCTTTGCTCGGGGTAAGAAGAGACGCAATTTTGACAATGCTGAGAAGACATTTTCTGCACCCTGTGCCTTGTGTTCAAAAGCAGATTTGCGTTTGATCAACTTTTTTTGTTTTTGCTGGTAGAC

>Isogroup_48 AAGCATTATTTCCATCAAATGCAAATTGTAGTCTCATCTTTTGATGTGGTTGCTGGGCCTGGATCTGCAAAACCTTATACTGCAGTTGCCCTTCAGACAATATCGCGGCATTTCCGATGTCTGAAGGATGCTATCAACGACCAGGTCAATGTTATCCGAAAGAAACTTGGAGAGGAGGAAAACTCATCCGGCAGAGAGGGCAAATTAACTCGCTTACGTTACATTGATCAGCAGTTAAGGCAACAACGAGCTTTCCAACAGTATGGTATGTTACAGCAAAATGCTTGGAGGCCGCAGAGGGGACTGCCTGAAAACTCAGTTTCAATTCTCCGTGCTTGGCTGTTTGAACACTTCCTTCACCCGTATCCAAAAGATTCAGAAAAGCTAATGCTAGCGAGGCAAACTGGTTTAACAAGAAGTCAGATTTCAAACTGGTTCATAAATGCCCGTGTCCGCCTCTGGAAACCAATGATCGAAGACATGTATAAAGAAGAGACTGGGGAGGCAGAGCTTGACTCAAACTCCTCCTCTGACAATTTACCAAGAAGCAAAGACAAAGCGGTATCTTGTGAAGAAAACGAAGATCTGAGATGCTCCAGGAGTCAGGGTTACCAAACCAGTGAATCCAAAGCCAACATTGGGATGATGGTGGGTTTCACTGGCGCACCAGCCAGCTTCCACAACGAGGCGAACTCCGATGATGGCTTCATGAACCTGCTGTTGAAGGACCAAAGACCGGACGAGGCGGATGACGGTCTCCTCCATGATGCCGCTGTCCATCACGCCGACGAGAGCGCGCGGTTCATGGCCTACCATTTGGCGGAGCTTGGGGGATACCGGAACAGCAACGTGTCGTTGACACTAGGGCTGCAGCATACCGAGAACAGCCTTTCAGCTCCAAACACCCATCAGCCGGGTTTCCCTGGCGTGGAAGAGGGCATCTACAACACAGCATCGCATCCCGGAGTTGCCGCCGCCTCCTCAGACTACGAGTCGGCGAACCAATTAGACCAGCGGCAAAACTTTGAACCGTCGCCTCTACTTCATGATTTTGTGGCCTAGTAAATGCTCGTCGGTAAAAGATTTGGATGGAGGAGCATTTGTAATCTGTAGGGCAAATGATAACACGCCTGAAAAGCAATGGGAATTGTAGCTGTAGTCACATTTGGTACCTCGGCCGCGACCACGCTAGTCGTGGTGTGGTCGGCGT

>Isogroup_49 CGAATGATGGTCTGGCCCCAATTCTGTGTGTAACTAAACTCACTGCTAACCTCAACCGATGCCTTCGCAACTTCTGGGATTCCAGCTTCAATTGTGGTGGTCACACCTAACTTGATGGAGGCACTTGAGTCCCATTTGGTCTCCCGGCTGACATTGTAGTTGAGGGATAAGGTTTCCTTGTCGTCCCTACTGCCACGGTTAACTGCCTCTTGAGTAATCAAAGTGTTGAGCTTCTTGCCTTGAATTTTGGCATCCTCGGTGCGGTAACGGACGTCGTAGATCCTCCGAGAAAGAACAGCTTCCTCCATCTTGAGGCATGCCTCATTGTGGATGGATGGAGAAGAGGCGTTGAGGCAGCTTTGCTTGTAGTCGCAAGTCAGCCTGTGGCAGTAGTTGTCGTTGCCGACGTTCTGGAGTGCATACATGCCTTCTCCATCAAGCTTGACTGGACGGAAAAGGGTGTCAGGGTCGTTGCTGCTTTGGTCATCAGAGTCCACCCAGATCCAGTTGGGACTACGCCTCCAGAACTTGCCATAGTAGTCAGACTTTATGCGGATGGTGCCATCCCTGTTGGGGTAGATGGTGTGGCTGCATCTCTTATCATTCTTGTCCTGAGGGTATACGTAAAACTGCAAGAAAACATAATCTTCGCCTGGAGGGGTGACGGCGGCCATGTAGTTGCCATTGTCTCCTTTGAAACAAACGCGCTTAGGCAATTGTTTCCCCATGTTGCCTTGCCGGATGGAACGGAGGCTGATGAGACAGAAGTGAGTAGGTGAGGCGAGACCTCGGCCGCGACCACGCTATCGAGCGGCCGCCGGCAGGTACCACACAGAGCACGAGCTTCTCCGTTACCTGCACAAGCTGCAAACCAAGGATCTCTCACTATGCCACAGTATGATTCCTCTTGGTTCTTGCACCATGAAACTGAATGCTACGGTCGAGATGATGCCCGTCACCGATCCCAAGTTTGCCAACATGCACCCATTTGCCCCTATTGATCAGGCTGCAGGCTATCATGAAATGTTTGACAACTTGGGTGAGCTGTTGAACACGATCACCGGCTTCGACTCTTTCTCTTTGCAACCAAATGCTGGTGCTTCTGGAGAGTATGCTGGACTGATGGTTATTCGGGCCTACCACAGGGCAAGAGGAGACCACCACCGAAATGTCTGCATCATTCCTGTCTCGGCACATGG

>Isogroup_50 GGGTCCTGATTCCTATATGGGTGCGAACATAGCAGACCTATTTAAGAGAATGACTATTATGTCAGATGAAGAGATTGCTGAGATACATTCAGATCACAACAGAGAAACTATAAGTTCATTGTTACCACGTCTACATTACTATCAGAATGGTAACTGTATTGTCCATGATATGTTTGGCCATGAAGTTGTGAAGAAAATAAAGGAGGAATATTGTGATGCATTTCTAACAGCACACTTTGAGGTCCCAGGAGAAATGTTCTCTTTGTCCATGGAAGCAAAGACTAGGGGAATGGGAGTTGTTGGATCCACCCAGAATATCTTAGATTTCATTAAAAAATCATTTGAAGCAAGCTCTGGACCGGAATGTTGATGATCATCTCCAGTTTGTGTTAGGAACAGAATCAGGAATGATCACTTCGATTGTTGCTGCTATCCGCAAATTATTTGATTCCTATGAATCTTCCAAAGAATGCGCTAACATTGAAGTAGAAATTGTGTTGCCTGTTTCACCAGATGCAGTTTCTAAGACATCGGTTGATGATTCTCATAATTTTTGCTCTTCAGTAATTACTGATTTAGAACAAATTACTATTGTCCCTGGTGTGGTTTCTGGTGAAGGTTGCTCTATCCATGGGGGCTGTGCATCATGCCCTTACATGAAGATGAACTCTCTAGGGTCGCTACTTAATGTCTGCCAGCAGCTTCCTGATGAAGACAACATACTATCTGTCTATCGGGCTAGTCGATTAAATGCCAAGACGTCTCATGGGAAATTGGTTGCGGAGGTTGGGTGTGAGCCAATTTTACATATGAGGCATTTCCAGGCGACAAAGAGATTACCAGACAAGCTTGTTTACCAGGTTATCCATGGTAACGGAAGGGAATCTTCGTGATGGCCGTTCCTCATATCCTCCCTGTTATTTTCGTTCAGGTATGTCTGGACGGTGTGCTGCTTGCGGGGTGCAGTCTGTTTTTTGTAGTTAATATTGTCTCCCATTCATAGATCTGACACCCAGATGTTGAAACATACGCGAGACAACATTTGGATTGAGAATAAATAACTGGTCAGCTCAATTTGTGAGGAACGGCCATCCGTTTTTTGTAGTTAATATTGTCTTCTATTCGAAACTCCGTAAGTGTCACTATGCACCTTGTAATATCAGCAGCGGATAACAATGTATACAATAAAGTGGCTCAACG

>Isogroup_**51** AGAGATGTGAGGTCTGCGGTTGACTTGTGCCATCGTGATGGAACTTTAAAGCGAATGGTTGCAAAGGAACCTAGCAGGTATATCAATGAGGACTTGGCAATTGTGCCAATGCTTGAAATGCTAAGAAAATCAGGACGCTCAACATTCTTGGTAACAAATAGTTTGTGGGACTACACTGATGTTGTCATGAACTACCTATGTGGGCCATATATATCGGATTTGGGCTCAAATCACAAGCAGAAGTGGCTTGAGTATTTTGATGTTGTTATAACAGGCAGTTCAAAGCCAAGTTTTTTTCATGATGATAATCGAGCAGGACTGTTCGAAGTTGAACCTCATTCTGGAAAACTTCTAAATGCTGATCTTCAGGTTGGGAGCCCAAGATCAAGTCAACCTCTGCCAAAACCAATTCGCAAAGTTTACCAGGGAGGCAATGTTGGTCATCTTCATAAATTACTTTCTGTTGCTTCTAGTTCACAGATCCTATATGTTGGCGATCACACATATGGAGATATACTACGCAGCAAGAAAGTCCTAGGTTGGCGGACTATGCTGGTAATACCTGAGCTGGAGCAAGAGGTGAAGCTTCTTTCCGAATCAAAGTCTACTCGAAAGGAGCTTAGACGACTTAGGATGGAGCGTGATTCGATTGAAGACAGAATCCATCGCCTTGAATGGTCTCTTCAATTTGAAAATCTTGCAGAAAATGACAAGGAAAAGTTGTTATCTGAGCATGCCAATCTACTGCAAAAGCTGAAGGGTATCCGCTGCGTTCTCCGAGATGCTCAGCTGCAACATCATCAGAAGTTTCACAAGGTGTGGGGGCAGCTTATGAAGACTGGATACCAAAATTCTCGTTTTGCTCATCAGGTTGAGAGATTTGCGTGCTTATACTGCAGCCAAGTTACAAACTTTGGTTTATATTCTCCCAACAAGTATTACCGCCCAAGCGAAGATTACATGCCCCATGAGTTCGATGTGCTTGGCCTGTAGGAACTAATTTATGCTCATGTTGAATCAAAGTTCTCCTTGGAAGTCTCGGCTGCAGCAAGATATGAGCATGATTTTAGCGTGACACTGAATAACAATCTAGTGTCCTATATTACCACCTTATACAAAGGATGAGGTTACCTGGTGAGACTTCACCCATTTTACTATAGTTGCTCATAGAAAATTTACATGTTTCCCTTTTTT

>Isogroup_52 TTTGACGAAGTTCTTCCAAGACTAGTGAAACAAGGAAGTGACGGCAATTCTGGATCGAGTGCCCTTTGTGATACGAACTGCTTGCAGGCACTCTCTAAAAGAATCCACTATGGGAAGTTTGTGGCAGAGGCCAAGTTTTCAAGAGTCCCCTGAAGCTTACATGCCTGCAATACTAGCTCAGGACGGTGATCTACTAATGCAGCTCCTCACCTACGCGACGGTTGAGCGTGCAATTGAACATAGGGTAGAAACCAAGGCCAAAATATTTGGGCAAGAGGTGAATATAGGAGCCGAAGCCAAGGGCTTGCCACCTGTCTACAAGATCAGACCCAGCTTGGTCGCTGAACTGTACCACATAAAGAATCAATTTCATCAATGAAGATAATGGATGGAGCATTTTCGCGAGCCATTTGGAAAAGATTAGCAACTAATTTCTCGCTCTCTCCCATCCATTTTGAAACAAGGTCTGATGAAGATATGCTGAAGAACGTTGAATCAGCCTCAGTAGCTACAGCTTTAGCCAAATAAGATTTTCCTGTTCCTGGTGGACCGTCATATCACATCCGCTCCCTTAAATACCACATCCTTGTGTCCTCTGAAACCACCTCCACATTCACACTCTCCTGGAGAACACAAACAGGATACACCTGTCTTACCACTCCAATGGCCACCACCACCATGTCTGTCTCCTCGTCGACCTTCGCTGGAAAGGTAGTGAAGAATCTGCCATCGTCCACACTTTTTGGCAAGGCCCGCATTACGATGCGCAAGACTGCAGCAAAGGCTAAGCAGGTCTCCTCCGGTAGCCCATGGTAGGGTGCTGACCGTGTGCTCTACCTCGGCCCGCTATCCGGTGAGCCCCCAAGCTACCTAACCGGTGAGTTTCCCGGTGACTATGGCTAGGACACCGCTGGACTTTCCGCTGACCCCGAGACTTTCGCGAAGAACCGGGAGTTGGAGGTGATCCACAGTCGTTGGGCCATGCTCGGGGCACTCGGTTGTGTCTTCCCTGAACTTCTTGCTCGCAATGGCGTCAAGTTTGGCGAGGCCGTTTGGTTCAAGGTTGGCTCCCAGATCTTCAGTGAGGGTGGCCTCGACTACCTCGGCAATCCTAGCCTCGTCCATGCACAAAGCATCCTTGCTATTTGGGCATGCCAGGTAGTGCTCATGGGTGCTGTTGAGGGG

>Isogroup_53 CAGACTTTTGATGCTGTTGATGGTAAACAAGCGAGGCGCACCAGCTCATCAAGTCCTCTAGGAGAACTTTTTGATCATGGATGTGATGCCCTTGCCTGTGCTTTTGAAGCCTTGGCACTTGGAAGCACCTTGATGTGTGGAAGGTTGACACTCTGTTACTGGGTGGTTGCTGCTGTCCCATTTTATCTGGCAACATGGGAACACTTCTTTACAAATACGCTCATACTTCCTATAATAAATGGACCAACGGAAGGCCTGATGCTGATCTATGTCTCCCACCTTTTTACCTTTTTTGTTGGTGCTGAATGGTGGGCGCAAGATTTTCGGAAATCTCTCCCCCTAATTAGTTTGGTTCCACTTCCATTTGTTCCAGAAATCCCCTTGTATGTTATTGTGCTGATTCTTATGATCCTATTTGCTGTAATCCCAACAGTTGGATCCAATATTGGCAATGTCCAAAAAGTAGTTGATGCACGAAAAGGAAGCATGGGACTAGCATTAGCAATGCTCCTTCCGTTTATTGCACTGTTAGCTGGAGTGGCTGTCTGGTGTTATCTTTCCCCTTCAGATATCATGAAGAACCAGCCACATCTGCTTGTGATCGGAACTGGTTCTGCTTTTGGATATTTGGTTGGGAGGATGATACTTGCCCATTTATGTGACGAGCCCAAAGGTCTAAAAACTGGAATGTGCATGTCTCTGGTGTTTCTTCCATTCGCGATAGCGAATGCTCTCGCCGCAAAGATTAACAGCGGGACTCCTTTAGCTGATGAGCTTCTGGTTCTTCTCATGTATACTGCATACACAGTGGGTCTTTACTTGCATCTTGCCTTTTCTGTTTGTCGTGAAATCAAGGAGGCTCTTGGAATCTACTGCTTCAGGATAACCAGAAAAGAGGCTTGAGTGTGTTTTAGTTCCCACATACCATGGGATGCATTCAGCAACATTATATTCTCCCAAGATATTGTCTGCCGTCTACCAGGCCAACGACACAACGGCTCGTCGCCGCGCATGCTGGAAGAACCCTGTCTCTGGGACAGCGTGGTGACTAGGGAGTCATTTAGCTTGTGTTCAGGTAGCTTATAGATCTTGCGAGGAATGTAATGCAGATTTATCTGAAGTAGCCTATGTACCTCGGCCGCGACCACGCTAGTCGTGGTGTGGTCGGCGTCTC

**>Isogroup_54** CAAATGATTTGCAATAGATCAATCGTAGAACCGCTGCTAATAATGCACAGCTAAAGCTAAGTAGGATCGACTTCACTTCTAGAAGCTCGTGTATATTTTTCCTGTGGTTGTATATCTATTTCAATTTTCAAACCTCTGACTAAAGTGTGAAAAGAAGTGTAAAATTGAATTTCTACTCTGATCTACCATTCGGCCATTCACAGAACAACTCCACTTCTCATTATGAGATTCTTGTAAGCTCGGCCAAAACATTCTAGACCATAAGCTATTACCAAAAGGCAGGATTTATGTCATCGTTGTAAACCTCAGCTTTCAGTTGCGGTCAACCTTCATCTCATCTTCCTCGTCGACAGATTAAACATCATCTGAGGCTCCTGTCGTTGAGCCATCAACTTCTTTCAGGATGTTAGGCTCTGTCTTTGGTTGCTCAGACTGTTCTTTGTTATCTGAATCTCCTATCGTTGAGCCATCAACTTCTTTCAGGATGTTAGTCTCTGTCTTTGGTTGCTCAGACTGTTCTGTGTTATCTGATGGTTTCTTACAAATTTGTGGCTTGCTATTAACAGGATTTGACTGTTCTTCGGAGACTGAAGTTGTCCCAATCTCATCCGTGGCAGCTGGATCAACATGCTGAATTTGGCCTGATGATTCCTTGCTTCCATTTGAAGATTCGAAATCGCTGTTAACCTCAGGGAAAGATTTTCCTTCAACTGGCACATCTTGCTTATCTCCTTGCATGCATGGTAATAATACAGGTTGAGGCGCTTTAACTGCAACCTCGGTAGCTGATGGTTTTCGGCTCACAGATTCAGTTTCTTTCTTGGGCAAAGGCATTGATTCAGCATTTCCGGATTTAACTGAGACAGGTGCTGCTGCAACAACTCCAGCACCAATGTATGGCCTTGGAATGAGAGGGATGATGTGCTTAGCAGTAGATTTCTCGACAGGTCTCCTCATCTGAAGAGGCTTTGCAATTGTAACATCCTCCAAGCGGACAGGCAAGGTAGAACACGCTTGCCTGTGGCTTACAGATGCCTTCTTTTGAACTTGACTTGGCCGGAGCATGGGGAGGTCAGGGGCAAGATCATCCATCGGTGTTATTGGGCGTAGGTTATTTGCATCTGGGATCCAACCTCTGAGCACCTGATTGTCCTTCACCTTCACCGTAATAA

>Isogroup_55 TGCTTCTTGTTCTTCAATTTTCATTGCCACTTCATCAGTGAAACTCTTAGCTTCCTCTTCATCTTTTGTCTTCTGAACACAAGTCTTCTCTCCTGCCATAACTGATTTATCTTCCTTTGAACTTTGTTCTTCATCTGTGCTGAATTTTGGTTTTGCCATTTCCACAATATTTTCTTCAGCCTCAGGAGCTTCTTTTGCTGAAACCACATCAAGACCACGTTCCTGAACCTGTGGCATAAATATTTCTTGCTCTTCACTGGATTTCGTATTCACTGTTTTCACTTGGGTTTCGACTGTTCTATTTACATTCTCTTCTTTGATAACAGGCTCTGTTATGGCCAAACAATTCTGATCACCTCCATCAGATTCTTTAGGAGACTCTTGTCCAATTTTGGTTTCCTGCTCCTCATGCAAATTAGACTCTCCTGGTTTGTCTAGCAGGTTGTTCTTCTCCATCACAATTTGGTCTCCAGCAAATTCATCTTGCTTATCTTGGTTTGTATGCAAGCTGGAACCATCTGACTGAATATCCAAAGCTTTCTCAGAGCTGTGTAATTCACTGTTAGATAACTTAGAGCTTTGTGTTTCTTCCTCACATTCTGAGCCTGTAAAAATCTCCTTTTTATGAACCAAATTTATCTCAGTGCCCATAACTGGTTCAGTAATTTCAGCTGTCTGCCCATCAGATATTGCCTCTGCTCCTTTCTGCGAGTTACAATGCGAGTTTTCTATCTGTCCTTCAGTAGCTCCACCTGTGCTGGTTGTATGCTGTTTCTCTTCTTCCTTCTCTACTTCATTTTCCAGTTCCGCCTCGATAGCATTTTCAGCCTCAGTGATGGAAGATTTAGTGAACTCTTCTCTTTTTGTGCTCAGAGGACTTTGAAGATCATCACTGTCCTTTGGCAATTTCTTTTCATAACTGGTATCCGGTTCTATCGACAAGAGTGTTTGCTCTGCCACTGCATCAGAACTTATGTCTTCAGGTAAAGACTCATGCTGAAGGTCTGTTCTTCCTTCCTCTAATGCATCCTCTTGAGAAACCTTTGGGTTTCTTGAGTCATCAAGGTCGTTTCTCAAGCTTCCTTCTTCAGAATTGGTTGTGTTTTCACCTAGCTTGATGTCTCTAGTTTCCACTTCTGTCATCAGGTCATGTCCACCTG

>Isogroup_56 CTGAGAATGGAATATCTACGCCTGGTAATCAATGGGATTCTCCACCTGAAAGGTCTGCAGGACTGGAAAAAAGGCACACCATCACCAGCCAGGAGGGTCTCACTTTCAGTTGAAAGGTCTCCTCAGGCTCATTCATCTGATAAACTGGACTCACCACGACATGCACAAGATGATGGTAATTTGGCTCGCTCTCGAAGCCCACCAGCAAGACATCGGTCCCGATCTCTTGAAAAGCGTGATACCCACCGTAGAAGAGCTTCGTCACGTGAATCGTCTCCACATGCACGGGATAGCTCTCCTCCAGAAAAAAAAGCACAGTCTCGACGTAGAGATGGGTCACCACGACGAAAATCTGCATCCCCTAGAAGAATAGATAAGTCCCCACGACGAAGATCTGCATCCCCTAGAAGAAGAGATGGATCACCACAACGGAGATCAGCATCCCCTAAAAGAAGAGATGATTCACCACACCGAAGATCTGCATCCCCTAAAAGAAGACATGGGTCACCCCGACGAAGATCTCCATCCCCTAAAAGAAGACACGGGTCACCACGACGAAGATCTCCATCCCCTAAAAGAAGACATGGGTCACCACGACGAAGGTCTCCATCCCCTAAAAGAAGACATGGGTCACCACGACGAAGATCTCCATCCCCTAAAAGAAGATCCTCACCCAAGAGGAGGAGTTCACCAAGGAGGAGGGATTCCCCATCAAGGAGGAGGGATTCCCCACCAAGGAGGAGGGATTCCTCTCCAAGGAGAAAACTCCTCACCAAGAAAGAGAGACTCATCACCAAGAAGAAGGGACTCCCCAACAAGGAAGAGGGATAGGTCAAAAATCAAGGTCACCTTCAAGGAAAACTGATTCATCTAGACATAGAAGGGAACATGGCAGATCTCGATCAAGGTCTCCACACTCAAGGGATCACCATAAAAGATCTCCAAGAAGAAGGCATTCACCAAGGCACAGATCACCTCCAGCAAGTCATCGTCAGCATTCTCCTAAAAAGACCTTGGTCACCACCTGCCAATAGGAAGACTGGATTGGGTAAGCCTGGAAAGAATCTGTTTGTTGCAGGTTTTAGCTATGCCACAACTGAGCGAGAATTGGAGAAGAAATTCGCTAAGTTTGGACGTGTGACACGT

>Isogroup_57 TCCCGGAAGCTCCTCGAGGCTGATGGTTGGATCGTGAATCGTATAACTTTACTGGCTAATCCTAATCAAGTGAGACCAACGAGGTTTTGGGGTGTCTACACAAAGCTAAAGATATTCAACATGACAGACTACAAAAAAGTTGTTTATCTTGATGCAGACACTATAGTTGTAAAAAAGTATTGAGGATGTTTTCAAGTGTGGAAAGTTCTGTGGGAATCTGAAGCACTCTGAAAGAATGAATTCTGGAGTGATGGTTGTAGAGCCATCTGAAACTCTTTTCAAGGATATGATGAACAAAGTGGACCAGTTGCCTTCCTACACTGGAGGGGACCAAGGTTTTCTTAATTCGTATTATGCTGATTTTGCTAACTCCCGTGTGTATGAGCCAGACTCACCTTTAACACCAGAACCTGAGACACAGCGCCTTTCTACCTTGTATAATGCTGATGTTGGTCTTTACATGCTAGCCAACAAGTGGATGGTTGATGAGAAGGAACTAAGAGTTATTCACTACACACTAGGTCCCCTTAAACCCTGGGACTGGTGGACAGCTTGGCTTGTAAAACCCGTGGAAATATGGCAGGATATTAGGCAAAAGCTTGAAGATTCTCTTCCGGGAACTGGTGGGGGAGGAACCCTCATGACCAGCTGATGGTCAAATTTTTATTCATTATTCCCTTCTGCCTGCTGTTATTTGGTTATTACCAATCATGTTTTCAGAACAATAAGACGTTTCCAAGTGTGCAGTCTTTATGTGCGTTTGCTAGAAGAGGCCGTCACAAATACAAGTCTGAAGAAGCACTTCCATCTTATTCAGCAGTTGGTGTTTCATCATCTACATTTTCTAATTCAAATCAAAGATTCTCAAATGGGCCATATTTGAAGCTGCCCTCTTATTTCGGGGCGATTGCTGTGCTAGTCTGTTTTATGTCCGTTGGTGTCTCTCTCGCCTTTGCTTTCACTATCATTCCACGGCAAATTATGCCATGGACAGGTTTACTCCTGATGGTTGAGTGGACCTTTGTGGCATTCTTCTTACTGTTTGGTAGTTACCTCCGTTTTGTCTATCGGTGGGGAAGTATCAGTGCAAATTATTCTGATTCATCAGAGAATCGTGCGGG

>Isogroup_58 TTTTATGCCCCCTGGTGTTACTGGAGCAATCGATTGAAACCTTCATGGGAAAAGACTGCGAATATAATAAGAGAGAGGTATGACCCTGAAATGGATGGGAGAATTCTTCTCGGCAAGGTTGACTGCACCGTGGAAGTTGAGCTGTGTAAGAGGCACCATATACAAGGTTACCCATCAATTCGCATTTTCCATAAAGGGAGTGATATGAAAGAGAACCAGGGTCACCATGATCATGATTCATACTATGGGGAGCGTGATACTGAAAGCTTAGTCGCGGCAATGGAAACTTATGTTGCAAATATTCCAAAAGATGCCCATATGCTTGCTTTGGAAGACAAATCCAACAAGACTGTTGATCCTGCAAAGCGTCCTGCTCCAATGACCAGCGGATGCAGAATAGAAGGTTTTGTGCGGGTCAAAAAGGTTCCTGGGAGTGTTGTAATATCAGCTCGATCTGGTTCACACTCATTTGATCCATCTCAGATAAATGTTTCCCACTACGTGACACAGTTCTCATTTGGCAAAAGGCTCTCAGCAAGGATGTTTAATGAACTGAAAAGACTATTTCCCTATGTTGGTGGACACCATGATAGATTAGCTGGTCAGTCTTACATTGTTAAGCATGGTGATGTCAATGCAAATGTTACTATTGAGCATTATCTACAAATCGTAAAGACAGAGCTGGTTACGCTGAGAGCCTCGAAGGAGTTGAAAGTGCTTGAAGAATATGAATACACAGCGCACAGCAGCTTGGTGCACAGCTTCTATGTTCCTGTCGTGAAGTTCCATTTTGAGCCTTCTCCCATGCAGGTCCTGGTGACTGAACTTCCCAAATCCTTCTCCCACTTCATCACAAATGTCTGTGCTATTGTTGGTGGAGTTTTCACGGTTGCTGGAATACTTGACTCCATCTTGCACAACACCCTACGGTTGGTGAAGAAGGTTGAGCTAGGAAAGGACATTTGAACAAGAGCGTTAGATATATTCCCTGAAGGTTGCACTAGCTAGTCATTATTTTTGTTATACTGGGTAGCAATTCTTAATGCATGAAACTAGTATATACAGAGGGGGTGGGGTTGAGTTGACGGGAAGACGGCGTCATGATATAACCGGG

>Isogroup_59 AGGTTAATCTCGACTTCAGTTCAGAGCAAGATATGATTAATAAATTCCGTGCTAGCCTCGCATTGCAGCCTATCGCGACAGCGATATTTGCAAATTCTCCCTTCAAAGAAGGAAAACCAAATGGGTTTCTCAGTTTAAGAAGCCATATTTGGACTGATACTGATAACAACCGCTCAGGGATGCTTCCTTTTGTTTTGATGACTCGTTTGGATTTGAGCAATACGTGGACTATGCATTAGATGTCCCAATGTATTTTGTATACCGGAACAAGAAATACCTTGACTGCACCGGAATGTCATTTCGGGATTTTATGGTAGGAAAGCTCCCACAGGTTCCAGGGGAGTTGCCCACTTTGAACGACTGGGAGAACCATCTAACAACAATTTTTTCCTGAGGTTAGGTTGAAGAGATATATCGAGATGAGAGGTGCAGATGGTGGCCCATGGAGGAGACTTGTGTGCCTTGCCTGCATTTTGGGTTGGGTTGTTGTATGATGATGAATCACTACAAAGCATTTTAGACATGACTGCTGATTGGACAAAGGAAGAAAGAGAGATGCTAAGACGGAAGGTGCCAGTAACTGGTTTGAAAACACCATTCCGCGATGGCTATGTAAGAGATTTAGCTGAAGATGTTCTCCAGTTGGCTAAGAATGGACTAGAAAGAAGAGGATACAAGGAGGTTGGTTTTCTGAGAGAGGTCGACGAAGTGGTTAGAACAGGCGTGACACCCGCTGAGAGGCTTCTGAACCTGTACCTCGGCCGCGACCACGCTAAGCGGCCGCCCGGGCAGGTACAAGAACAACGTTTCGGGGAATATACCTCCAACACTCGGGAAGTTGAAGTCCCTTGTATTCTTGCGGCTCAATGGCAATCGTTTGACCGGACCAATTCCAAGGGAGCTGGCTGGAATATCTAGTCTCAAAGTTGTTGATGTTTCTGGTAATGATCTGTGTGGAACAATTCCCACCACTGGACCATTTGAGCACATTCCTCTCAGCAACTTCGAGAAGAACCCGCGTTTGGAAGGTCCAGAACTTCAAGGCCTGGCTGTATATGATACCAACTGCTAGATGGCAACATGAAGGAAAAGAATGCTAGAAAGTAACGGGC

>Isogroup_60 CTCGTCGGAAACATCGTCGAGATTTTGGAAATGAACCCTGAAGATGAAGCTGAAGAGGATGGTGCTAACATTGACCTGGACTCACAAAGGAAAGGAAAATGCGTTGTTCTGAAGACATCCACAAGACAAACTATATTCCTTCCTGTGATCGGGCTAGTTGACCCTGACAATCTCAAGCCTGGTGATTTGGTTGGAGTCAACAAAGACAGCTACTTGATATTAGACACACTACCTTCAGAGTATGACTCTCGGGTAAAAGCAATGGAGGTTGATGAAAAACCTACAGAAGATTATAATGACATCGGTGGCCTTGAGAAAGAGATTCAAGAGCTCGTTGAAGCTATTGTGTTGCCAATGACACACAAGGAGCAATTTCAGAAGTTGGGAATCCGTCCTCCTAAAGGAGTTCTTTTATATGGACCACCTGGGACAGGAAAGACGTTGATGGCTCGTGCATGTGCTGCACAGACTAATGCAACTTTTCTTAAATTAGCTGGTCCGCAGCTAGTCCAGATGTTCATTGGTGATGGAGCAAAGCTTGTCCGAGATGCCTTTGAGCTAGCAAAGGAGAAGGCACCCTGCATTATTTTTATTGATGAGATCGATGCAATTGGAACAAAGCGTTTTGATAGTGAAGTTAGCGGTGACAGAGAAGTGCAACGGACTATGTTGGAGCTGCTGAATCAGCTAGATGGATTCAGCAGTGATGAGAGGATAAAGGTGATTGCTGCAACAAATCGTGCGGATATTCTTGATCCCGCTCTACTGCGTTCTGGTCGCCTGGATAGAAAGATTGAGTTCCCTCACCCGAAGGAAGAAGCAAGAGCTAGGATTTTGCAGATCCACTCGAGAAAGATGAACGTGAATCCTGATGTCAACTTTGAAGAGTTAGCTCGTTCAACTGATGATTTCAACGGTGCACAGCTGAAGGCTGTCTGCGTAGAAGCTGGCATGCTTGCTCTACGCAGAGATGCTACAGAGGTGATCCATGAAGATTTCAATGAAGGCATTATCCAAGTGCAAGCAAAGAAGAAGTCCAGCCTGAATTATTATGCTTAAATTCCCCTTAAAAATAGTGAATACGCTTAAGGGAACAGTTGGTCTGCTG

>Isogroup_61 ACATTGCTGACAAAGAATACTTTGATAAACTCCAACAAGCACTTGAAGACTATGATTGTGTTCTGTATGAAATGGTGACTAGTCGGGATAATTTGAACAATCAAAAGGACCCAACATTTGCGAAGAGGCTGAAATCTTCACGCAAAGGTTTCAGCATTCTTGGTTTTATACAGAAGCAAATGGCTCGTATCCTTTCACTGGACTACCAGTTAGATTGCCTTGATTATGGCGATGAGAAGTGGCAACATGCTGACCTTGACTACGAGACATTCAAGCAGCTTCAGACTGAAAGAGGTGAAAGCTTTTTCTCATTTGTGGTCGACATGACAATGAAATCTACTAAAGCCTTGATACAACCAAGTTTGCCAGATGGTCTTGATTTCTGGAGATCTAAGTTGCTATGGGCTTCACGTGTTCTACCAATGCCACTTGTTGGACTATTTGTTATCACCGGGCTTTGTTTGCCAGTTGAAAATCAAGATGGATATCCTGAGCTTGAAGCATTATCCAAACTAGATTTAGGAGCTGCTCTAAAGATTTTCCTGGCTAAGCAATTGACTTCTGATTTCACAGTTATGACTGCTCCTGCCGAGGATAAGTCGGTTATTATTGGGGAGAGGAACATAGTTGCCACTAAGAAGATGAAGTGTGCGATAAATAGAGGCTACAAGAGGATAGCGGTTCTGTATGGAGGTGGGCATATGCCAGACCTTGGAAGACGTCTTCAAGAAGAGCTAAATATGGTCCCATCAGATGTGCAATGGGTCACAGCATGGTCAATAAGAAGCCGGGAGCTGGATAGAAAATCCCTTCCATTCTTGAAGACGATGGCTGAGGTTTCAGGGTGGCCTTTGAATAGATACGAAACACTTGCTCTGCTCATCTTTTCTTCAGTTCTTGCCGTGGATCTTTGGTTCTGGGAGCTTCTTGTGGAAACAGGTTTGAACTGGTTATCTTTTGCTGGTTCCTGGATTGATCAATTTAGTGGCTTGTTTTAACGAAGAAATTGTCTGAAAAGAGACGGAAGAAGTTTTACGGAACACATACTGTATATGTGACATCACAATGAAATATTGTAAATCTATTTAAGGTTTGCAAGCTT

>Isogroup_62 AACCTAAGAGGCAAACTCTTATGTTCACTGCCACTTGGCCAAGAGAGGTGAGAAAAAATAGCATCAGATTTGCTGACCAACCCTATTCAGGTTAACATTGGGAACACTGATCAGCTGGTTGCAAATAAGTCAATCACTCAGCATGTGGAAGTTACCACAAGTATGGAGAAAGGGAGACGCCTTGATCAGATCTTGAGATCACAGGAGCCTGGATCCAAAGTCATAATATTTTGCTCCACAAAGAGGATGTGTGATCAGCTGTCACGAAATCTATCACGGCAGTATGGTGCATCTGCCATTCATGGTGATAAATCACAGGCTGAGAGGGACTCAGTGTTGAGTGAATTTCGAAGCGGCAGGTGCCCAGTTCTTGTAGCTACTGATGTGGCTGCTCGTGGCTTAGACGTAAAGGATATCAGAGTTGTGGTTAACTATGATTTCCCAACGGGTGTCGAGGACTATGTCCATAGAATTGGGAGAACAGGTAGAGCTGGTGCTACAGGAATTGCATATACATTCTTCTGTGAACAGGATGCAAAGTATGCTTCAGATCTCGTGAAGATTTTGGAGGGCGCAAACCAAACTGTTTCACAGCAGTTACGAGATATGGTTGGCCGCGGAGGGTATGGTGGCAGGCCGCGGGGGCGTTGGGCATCTTCTAATGATTCCTATGGTGGCCATGGATCTTTTGGCGGCCAGAGTCGGGATAGTTCAAGCTTTCAGTCGAGTGCTTATGGCAATCAACATGGTGGCACTCCTAGCTTCAATACCAGTAACAACAATCAGTCCAGTGCCGTTTCAAGCTTCCCTGCCAGCGGTGGCAACAATCAATCTGGTGAAGGTCTTAGCTTCCATGAAAGGTTCTATAGCTCTTCATCAAGGGGTAGTGACCGTGCGAGGAGCAGGAGTCCTCCCAAGGCTGTGGGAGTCTCCAACTGGTAAAATGTTGCCCTCAAGATCCGACCTAGCCTCACGGATGGTGACGAAAGGGAGTAGATGGAGTTGGTTCCCTAACCAAATTATAGTTATCTCTTTATGGTCGTTTGAGCAAAGAATGTCCTGGAACTTTGCTGACTTAGGTTAAGTTG

>Isogroup_63 CTTTTGAAGAAAATAGATCATCGAAGGTTGGGGGTTAGGTTTGATGAACAAATTCCAGGTGGTATTGATCTTGGAGGCAATTGCGAAGTTGATCATGGCTTATTTTGTTCAGTTGATTCTTTATGCCTTGACAGTCCAGGATGGGAAAATAGATCCCAGCACCCATTTGATGTGATTATCCAGTTTCTCTCTGAAGAAATACAGCATGGTCCTATAATCCTGTTTCTGAAGGACACAGAAAAAATATGTGGAAATAATGATTCCTATTATGGTCTGAAGAGCAAGCTTGAACATTTCCCAGCAGGTGTTTTTATAGTTGGTTCTCATATCCAGCCTGACAGTCGCAAAGAAAAGGCCAATGCTGGATCTCTTTTCCTTTCAAAGTTCCCATACAGCCAAGCAATACTTGACCTTGCACTGCAGGACTTAGATCGTGGACATGATAAAAATAAAGAAATGTCAAAGGCGATGAGACATCTAACGAAGATTTTCCCAAATAAAGTGACGATACAGCCACCACAAGATGAAGTTGAGCTTTCACGGTGGAACCAGATGTTAGATCAGGACGTTGAAATTCTTAAAGCAAATGAGAACACTTTAAAAATACGCTCGTTTCTAACGCGACAAGGCTTGGAATGCACAGATCTTGAGACAGTGTGTGTCAAAGATCGTATCCTTACAAATGAGTGTATTGATACAATAGTTGGTTTTGCTTTGAGCCATGAACTAAAGCACTTCACGCCTAAAAACCCAGACCCGTCAATTGATTTGCATTTCCCCCTATCTAGTGAAAGCCTTAAGCATGGAGTTGATATGTTGGAGAGTATCCAATCTGGCCCTAAGAGCAACAACAAAAGGAAGTCACTTAAGGATATTTCTACGGAAAATGAATTTGAAAAGAGGCTTCTCGCCGATGTCATCCCTCCAGATGAAATTGGAGTTACCTTCGAGGACATTGGGGCATTGGAAAGTGTAAAGGGGACTCTGAAGGAGTTAGTGATGCTACCCTTGCAAAGGCCTGAATTGTTTACAAGAGGACAACTTATGAAGCCATGTAAAGGAATTTTACTTTTTGGCCCACCTG

>Isogroup_64 CTAGAGATTATGAGCGTGTCCTTTTGCAACAAGATCAGATGGTGCCATCAAATAGTTATCTGCAACTGGAATAATAGTATCTTTCCATTGACCAAGGCCAACAAAATAGTTACTAATATATGCAAGGTAGTCGGCTGAAGTGATCAAGCAGAATGATAGGCTCCCTTTGACTCCTGTGTATCACCTCATCCGCATCAATCTGCTGAGCAAGTTCTCCCGACAAGACCACAATAACAGTATACCTACCAAAAAACAGGAAGCGGCATTAACAAAGAGTATCATTTGTTAAATCAGCAAATGTAGCACACACAATAGGAACAATCCTTCCATCTGTCAGCCGAAAGTTCATCTTTGCCTTAGGGACGCCAAGACATCAAATGTCACAATGTCAGTAATCCCAGTGATATGACCAACGATGTCTAACTAAAGAACCCTTAATGTTATTCTTCTTGCCCAGACCTTCGTTCCAGAAGTATACCTTCCCATGGTTGCACATAGGCGCTAGGAAAGAGGAGAAAAACGCATTCGACCGTTATGCGGAATAGTGTGAGAAGGAGGCTACGCGGCCATGGTTAAGGCCCTCCTGGTAGGGCTCTCACCCTACGTATGGCGGCATTGTTGCGGAATAAAAAGGATATCCTTCAATGTTCCCTAGAAATGATCTCCTTGCTCACACAATCAAATTAGACCATCCCAATTTCACCACCTTCAGATGCGAGAGATGCAAATAGGCTCCATGGGAGAAACCACATGCCGGCAGCACGAAGACTGCAAGCCAGGTGCTCGCCGTCGACACAACCCCTCCCACCTCCTTTCGTGCTGAGCATCGCCACGAGCTCCACATGGAACTTTGGCTAAAACATGCTTACGGAGAACGGGTATGTGCACTAACTATCTTTAGCAGCATGAGAAAATAGTTCTTGAATCATTTCAAGCACATGAACCAGAGGATGAGAGGTTATAAATTGGTCCTTCCAATGTCTTTCGGGTCAAGTCTGGCCAATGCGAGGAAGCAAATGTTGTGCAGATCTGAGGATAGGCTTTATCTCAAGGAAGATAATCTCTCAAGAAGTCTCTC

>Isogroup_65 CTCTTCTCCTCCACGCCCGTGACGCCCATTTGTATCTCGATTATGGTATGGAGTGGCATCTCCATGTCTGGAACTTCCCTCAGAGGCCTGTCGGCAAAATACGAGTAGAGTGCTCGCAGCACAGCCTGGTGGGATATAACAACCACTGGTGCACGCTGCCGCTCAAGCTCGATGATGACAGGTTCCAACCTCTGAATCACATCAAGGTAAGATTCCCCACGGGGGTAGCGATATCTCAACTTGTCCTTCTTGCGTGACTCATATTCCTCTGGCATAATTTTCTTTATCTCTTCATATGTCATCCCATCGCACACACCAGAATTGATCTCATCAAGAGCACGCCATTGTATCTTTGGGAATCCAACAATTGGAGTTGCTGTCAAAATTGTTCTCTGTAGAGTGCTGGTCCAAATAGTTGCAGTTTTCTCAGATTTTAGGCGCTTTTCAATAAAGTTGGCTAGTTTCTTCGCATAAAGCTCTCCGTCTTCACTCAGGACCGTGTCACCACCAACTCTTCCTCTAACATTGTGCAAACTCTCACCATGCCTGGTAAGCAAAATAGGTCGAGGCGCAAGATGAGAATTCACCAAGAAAAATACAATACGACCAGGGAGATAGCCGCTGATATTGTTCACCTGTAACTGACCACCCTCCCTTTTACCATATCAATCATTTTGATATAAGAACCTTCCTCCACTGGCTCGTAGACCTTCTCATAGTTTGTCAGACGTTCAAGGAAATCCTGCAATCCAGCTTCATAGTCTGGCTGGTCAGCATAGTCAGGGCTTTGCTGAATTTTCAGGCGCACATTCCTTTCGATTATGTTCCGATCGTTACATATTGTCTCCAGAAATATTATCTTACAGTTCCCTTCAGCCATCTTCATTAGCATCTGCCTTCTTTTACTTGTGTTGTTTGTTGCATCAAATATACCAACCTGACCTCCTCCGTGCATCCAGTCTATCATGTCTTCCATCGCTAAAGCAGCCACCTCATTGCGTGCCTCAATCCCTTCTTGGTTATCGGGACGGAAAAAAGTCTGCAGACTGATTTGCTCCATGCTTCAGACGGCGG

>Isogroup_66 GACGACATTGCGCACAACCCGGAGAATCCCAGGCCGGGTGTCATCATCAACCACCCCCAGGGTGGAGATGTCTATGCTGGGGTCCCAAAGGACTACACTGGGAAAGAGGTCAATGTCAAGAATTTCTTTGCTGTCTTGCTCGGTAATAACGACCGCTGTGACTGGTGGGAGCGGCAAAGTTGTGGACAGTGGCCCCAATGATCGACATTTTCGTTTTTTACAGTGACCATGGGGGCCCTGGGGTTCTCGGGATGCCTACCTATCCATACCTTTACGGTGATGACCTCGTAGATGTCCTGAAGAAGAAGCATGCTGCTGGAACTTACAAAAGCCTGGTATTTTACCTTTCGAGCGGCCGCCCGGGCAGGTACACCAATGTCCTTCCATCTGCCGTCAATGTCATGTGTGCTACAGGTTAGCCAGATCATCTACCTTCTTTCCACGCAAATCGGCCCAGAGCATCCTCAGAAAGATCAGATAACTACAACGCGTTCCTCTGCCCCACGAATTTCCGCGCGACGCCGAAGATGACAGCCTTCGTCAAGAGGCCCTGGTCGAGGGTGCACGCCAGAGCCACGGCCCCGCCGAGCACAAGGAACCTCGCCACACGGTTCTTCACGGGTTTCTCCTCGGCTTCCATGATGTCTGATTGGTCGCTGTTGGATTCCAGTCCTCGGACAAAATTGTCACCGACCTTGGTGTTGATCCGTGCCATCAAGGCGCTCTTCGAAGGTGGCTCTCCTGCAGCCCTGGCTTTCTGGTAGGCACGGAGTCCGGGCTCGATTCTTTTGACACATCCCCACATGCCCTGCCGGATACCGATCTTTGCAATCTCGTTTGGGATGCCCATATCCTCATGGTGGAACAAGAGAATCTCGCAGGCAGTCGATCCACCATTCCCTCGTCTTGACTCCACTGCACGGATGCACCAGCTTGAGTAATACAAATCTACTCTACGTGGTTTGCTGCGTCGCGGGATGGATGAACATGGCACACCCTTTGTAACGCAGTAGTACCTCGGCCGCGACCACGCTAGTCGTGGTGTGGTCGGC

>Isogroup_67 CACCATGAGGACGATGAACCAGAAGACGAGTTCTCTGTGCTTTCTCGCAGGTCCGCAAGGGATGGCGTAGCAGCACAGAGCAACCTGTCCTCTGTTCCACGAAATGAGAGGCCATATGCAAGCCCGCTTCTTCCTCCACCACCGTCGACAAAAAGGCCTGTTTACACAGAGGCAAGCAGTGTTGACTATCTAAGTGGAGACTCCTACAAGTCTGAAAAAGTGCCAGATGAATTTGTCAACCCAACGGCCCCAGCCAATATCTCAGCACCATCTTATTCAAAAACAGAAAAAGATCAACAGCCAAGTTATGATAGCGTGCCTGATGACTTCATAAACCCAACTGCAGTGCCCAGCTTTTCTATGCCTTCTCGTCTTACGAGTGAGTCGAATAGTTCATCTGTGAATAAGCTTGAGAGCCTTCCTGACGATGACTTTATAAACCCCACCGCATTACCTGGTTTTTCTTCGTCTTCAACTAATGAAGATCTTCCAAAAGCTCCATGGGAAGCGCAAGCTCCAGTCTCTCTACCGCCCCCTCCTGCAAGGTATGGCCAAAGACAGCAGTTCTTTGAGCAACAACATGGTTTTCCCGGTGGTAGCAATGGAGATGGCTATGATGACCTAATGACGCAGACAGAAAGCCTTTCTCTTAATCAAAGGAGAACTGAGAATGAGAGGATTGCACCGGTGTCGACCGTGTCCCGCCAGCCCAAACCTGAGGATTCCCTGTTCAAGGATCTTGTTGATTTTGCCAAGAAGAATCCGTCTTCTCCATCTAAACCAGCCAACAGCCGCAGGACTCGCTGATGGATGAGTGTTTCAGTGATGTAACACACATAATTGAGTTGCCAGGCAGCTGGTTTTGCTACAGGCTAGCAACTTAGATATACCATCAAATAAGAGGGTTCGGACTGTTTTTCATTGCCTTGGGAGTTATTTGGTTTGCGCATTGGACAATTGGAATCATAGATGTCTGATACAAAGCATGATACTGGGGTGTGGTGTGTATTTTAGTTGCCTTTGTCTTTCTTTTTTCTCTCTCTCTTTCTGG

>Isogroup_68 TGTTGAACTTTAGCTATCAGCTTGCCAAGGTCCCAACTATCCCTTGGGGTATCAGGACCTATGTTTGCCTCTAGTATGTCGTCCATCGTTAATTCAGCATATTCCACAATAAGTGATTCCAGACTACCAGATGCAAGAGCACGTCTTCTTTCTGCGTATACCCGATCTCTCTGGCTATTTAATACCTCATCATACTCAAATAACTGCTTCCTGATATCAAAAAACGTAGTTCTCAACTTTCCGTTGAGCTTCATCTAGGGCCCTTGTTAGCATCTTTGATTCAATAGGAAGATCTTCAACTCTGAAGGCTTGCATCAGACCCTGTATCCGGCCACCTCCAAAGATCCGGAAGATATTATCCTCAAGACTAAGAAAGAAGCGAGAGCTCCCAGGATCCCCTTGCCTGCCACTTCTACCACGAAGCTGATTGTCAATTCTGCGTGACTCGTGCCGTTCAGTTCCTACTACATGAAGCCCACCTGTCGCGATTACCTTCTTCTTTTCCTCCTCTGTGTAAACCTTAAATTCATCTGCTATCTTCATGAAAGCATTTCTCAGAGTTGCAATTACTTCATCACTTGTTGGTCCCTTCTCACACGAGTAGGAGAGACGCTCTTCAGCTTCTAGTTCAGGTAATGATTTTTCTCCCCATTCTTTGACAGCCACTTCAACTGCATCTTTAACACATGAAAGTGTGTCTTCTGAAAGTTCACATGGGAACAAGCTTTCATTTGTCTTCCATGTCTTTCTTGGTGGAAGCTGCTTTTTGGATACAATGACACCATCAATTGGATTGACAATTCTTGGCATAAGCATCTCACGCAACTTCAGCCTGGCCATGAATTCTGCATTTCCTCCAAGGATAATATCAGTGCCACGTCCTGCCATATTGGTGGCAATTGTCACTGCACCAAGGCGTCCACTCTGAGCTACAATCTCTGCCTCCCTCTCCACATTCTCTGGTTTAGCATTCAGAACCTCGTGAGGAATTCCTGCTTCACTTAGCTGTTCTGACAAAGCTTCACTTTGCTCAACACTAGTT

>Isogroup_69 AGATGGCTCATCATCTGAAGGTGACAATGTTGATGTTGGACTTGTCTTGTCATCATTCAGTACCTGCCCGGGCGGCCGCTCGATAGCGTGGTCGCGGCCGAGGTACAGGCTACAAAGGACTGTGTAGCTAAGACATTGAAGAGGCTAGAGGAGAAGATAAATGGACGAGTGTTAAAACACTTGGTTTATCTCATGAGAGTAGGAGAAAAATCTGTTCAGAGGCGTGTTGCTCTGGCTCTTGCACACCTCTGTGCTCCTGAAGATCAAAGAACAATTTTTATTGATAATAACGGTTTCGACTTGCTTCTTGATCTTCTGGTTTCGGTGACCCCAAAACATCAACAGGATGGTTCGGTAGCACTATACAAATTGGCTAACAAAGCTGCATCACTCTTGCCGATGGATGCGGCACCTCCATCTCCTACACCACAGATTTATCTTGGCGAGCAATATGTGAACAGTTCGACACTTTCAGATGTCACTTTTTTGGTGGAAGGAAAGCGCTTTTATGCACACAGAATTGCTTTGCTAGCTTCTTCAGATGCATTCCGTGCAATGTTCGATGGTGGATATAGGGAAAAAGATGCAAGAGACATAGAAATTCCAAATATCAGGTGGGATGTGTTCGAACTCATGATGAGATTTATCTATACAGGGTCAGTGTTAGTGACAAATGATCTTGCTCAGGATCTTCTTAGAGCTGCCGATCAGTATCTGTTAGAAGGCCTCAAACGCCTCTGTGAATATACAATTGCGCAGGATGTTAATTTGGAGAACGTGTCTGATATGTATGACCTATCAGAAGCCTTCCATGCCATGTCACTGAGACATACATGCGTCTTGTTTATTCTGGAGCAGTTTGACAAGATCTGCATAAGACCTGGTTTCTCTCAGCTAATCCAGCGTGTCATCCCTGAGCTCCGCAACTTCTTCGCTAAGGCACTAAGACCAAGCCACCGGAGTGCGCAGCCATGAGCCCCGCCCACATTCGTTGACAACACCACCACCATCTGACAGATGCTGCTGGATATCTTG

>Isogroup_70 CGCTCCATTGCTTTTGATTGGCTTGGTTTTGGATTCTCAGACAAGCCTCAACCTAAATATGGTTTTGACTATACTCTGGATGAATATACTTCAGCCCTGGAATCGCTAATCGATGCTGTTGCTCCTGATAAGCTCTCCATCGTTGTTCAGGGCTACTTCGCTCCAATTGTGGTCAAATACGCCAACGAACATCAAGACAAGTTGAATCATCTTATACTAATTAATCCACCGATAACTGACAGGCATGCAAAGCTTCCATCTACCCTTGCATGTTTCAGCAATTTCTTGTTGGGTGAGATATTTTCCCAGGATCCTCTGAGGGCTAGTGATAAGGCCTTGACTAGTAGTGGTCCATACATGATGAAGGAGGAGGATGCTACTGTATATAGAAGGCCATACCTTGTCTCAGGTTCATCTGGTTTTTGCACTGAATGCAATAACAAGGGCTATGGGAAAGGATCTTAAGGTTTACATTGAATCCATGAGGAGCATATTAGCGAGTGATTCGTGGAATACAAAGACAACGATATGTTGGGGCATGAGAGATCGTTGGCTCACATATGATGGGGTGGCAGATTTCTGTGACAACCTAAAACACAAGTTTGTAGAGCTGCCGATGGGTGGACACCATGTTCAGGAAGATCGCGGTGAAGAGCTAGGCAATATAATTAAACGTATACTGAGAGGATGACTACAATAATATGGGCCCAGTCTTCTAGAACATGCCGAAAACAGGGGTCAAAATGTGGCAGAGCAATAGCCAATATTGACATGCACTGTTCTTGGTATGAACAAGAGAAGCTGCACCGCCCCATTGAAGGCCAATCCACTCCATGGATAAGACAGGTCCCTAAAGTGGTGCTGAATGTCTCCGGACAAGAAACGGCAATCACCGGTTCATCAGTGTGCCCTATGTTTTTCAGTGTTTGACCTTTGTTTTTTGCATGATTTGAATGCTATTAAATTAGACCAAAGCTAGATGGCCAAGATGATAACTCTGCTGACTGCAATTTCTCAACTAAATGTTGGGAAT

>Isogroup_71 AGATGAAGATGTGAAGCGACGTATGGAAATGTAAAATTAGGATCTCCATAAATGTTGATGTCAGGAGCTGATCCGTATTCCCTCAAACAAATGGAGCGTGCATCCTCACTAGCAATTTGAGCTACCTGTATGGGGGATAATTTTGTATTAATGAGGCCTATGACTCCGGGCTCTGGTTCAGGATCATGCAAAGCCACATGCTGCCCTATGAGCATGCGAATACCAATCCTTGACATGTAAAACCGATCAAGGAACCCATGGATCTCATCGAATCCTGGGGGAAGCTTCCTTGAGCTGAACTGCTCGTTTTTCAGCTGTTGCACTCCCAAAGCCATTGTGGGAACCACATTATTGTGCCGCACTTTGATCATCTTAATCATCTGCGTGAAAGCAACCTCGTCGTCCTGGTTCCTCACTTCTGGAAAGTACCTGCCCGGGCGGCCGCCCGGGCAGGTCGGTGATGTAACAGTAATAGATATATTGGCTGAAACTATCACAACCGATGGCTCTAAAGGTGAAAAAATTCCATCAGCTGAGGCTACAGGGTTCCAGTTGGACACTGAAGAAAATGATAACCTTGTTCCTGGATTTCTGGGTTCGCTAATTGGGATTCGTCCTGGTGAGACTAGATCATTTCCTATTCAGTTCCCTGAGTCCTTTGATCAAGAAAGTCTGCAAGGTGTCCGCGCGCAGTTTACTGTTGTTTGTAAAGAACTCTTCTTCAGAGAGTTGCCAGAGTTGGACGATTCGCTTGCCGCAAAGCTCCTTCCAGGATGCACCACCATGGATCAGGTTCGAGAACGAATTTTGGAAAGATGCAAAGAAGTGGAGAAGACAGCTATAGAACAAGCGACAGATAATGCAATCCTCGACCAACTGGGAAAGTTGGTTGAGGTTGATGTTCCGCGTGCCATGTTTCAAGAACAAGGCCAGCAGTTGTATGGAGCCAAACTTCTGCAACTTCAGGCAGAAAGGCAGCTAAATAAAGATCAGTTAGCATCCCTTTCAAGCGAAAGGTCGGTCCAGGA

>Isogroup_72 TCAATCTCGAGCACATAAAGGCGATCGTTACTTCTGAAGAGGTTTTACTCAGAGATCCTTCAGATGAAAATGTAATTCCTGTAGTAGAGGAGCTCCGGAGGCGACTCGCGCCTTCAGGTGCCTCTCACCTTGATGGAAAGGATAATTTAAGTGGCCAGCATGATGGAGAAGGCGCTGAAGAAGATGAATCTCCCTTTGAGTTCCGTGCACTGGAGGTTACTTTGGAAGCAATCTGCAGTTTTTCTTGATGCACGCACTACTGAGCTGGAGACCGATGCTTACCCAGCTTTGGATGAACTGACATCAAAGATCAGCAGCCGCAACTTGGATAGGGTGCGAAAGTTGAAAAGTGGTATGACAAGATTGACTGCAAGGGTTCAGAAGGTGAGAGATGAGCTTGAACAATTATTGGATGATGACGATGACATGGCTGATCTTTACTTGTCTAGAAAGTTGGCTGGGGCATCATCCCCTATCAGTGGCTCTGGTGGACCAAATTGGTTCCCAGCATCTCCAACTATTGGTTCAAAAATATCAAGAGCTAGTAGAGCCAGTGCAGCAACTATACATGGAAATGAGAATGATGTGGAAGAGCTGGAGATGTTGCTGGAGGCATATTTCATGCAGATTGACGGCACATTAAACAAATTAACAACGCTGAGAGAGTATATTGATGACACGGAAGATTACATCAATATTCAGCTTGACAACCATCGTAATCAGTTGATTCAGCTTGAGCTATTCTTGAGTTCTGGAACTGTCTGCTTGTCACTTTACTCGTTGGTTGCTGGAATCTTTGGTATGAACATACCTTACACTTGGAATGATGACCATGGATACGTCTTTAAATGGGTTGTTCTTGTATCAGGGCTATTTTGCGCCTTCATGTTCGTCTCCATTGTCGCTTATGCTCGGCACAAGGGTCTCGTTGGGTCTTGAGTAGCTGGAATAACCGCATTACACGCTTTTCTCAGCTGTAAATCTTGAGCTAAAGCATCAACTTTGCGGATGATTCATCTCTT

>Isogroup_73 GTACACTTGCACTGCCCTTGATATGGTAAAGGGTAGAACCAACTGGCATTCAAAAGGAATTTTCGCACAGAAAACTAAGCTGCCAAACTTTTCGAAGCAATCTCATAGACATTTTCAGAGAGTCCATTGGCTGAGATGATCATCTCCAGCTGGGCCTTAGCAAGAGCTTGTCTGGTCTCATCATATCGCCTCCAACGAGAGAATGCTGACACCATGCGGGATGCGACCTGAGGGTTTATTTTGTCGAGCTGCAAGACAACTTCGCCCAAGAACTTGTAACCAGAACCATCTTTCGCATGGAAACTCACAGGTGACCCGCAAAATCCTCCAATTAAAGAGTATACCTTGTTGGGATTCCGCATGTCAAAAGCAGGGTGAGCCAGCAGCTTTTGAACATTGGCTACATTTCCAGGAATGTCTGAGGTAGCTTGCAGAGCAAACCACTTGCTTACAACCAAATAGTCATGCTGCCACTTGCTATAGAAGTCCAAAAGGGCCTCATCCCGAACTTGACCTGGGTTTTGAGATAATGCTGCTAAGGCAGCAAACTGCTCGGTCATGTTAGTAGCAGAGTGGTATTCACTCAATGCAAGTTCAGTGACTTCTGGCTCATTCAGGGATACAAGATAAGCAAGACATGTGTTTTTCAATGCACGGCGAGCCACACTATCATGGTTAAATGCGTAAGCTTCAGAACTCCTGTTGCTTGTTACAGCTGCAAGAAGATCATCTTTGAGTTGAAAGGCGAGTTCCTTCTTAATAAAGGTGCGAACAGCATGAACTGCATCGGGATCTGCAACTGGCATCATGTCCATAATCTCCCCTAGACCAGGCAGAGTTATTGCTTTTGCAATAAATTCTTTATCCAAGCTTGTGCTGCGCAGGATTGCTCGAAGTCCATCAACAAATTTTGGATTAAGAGCCAGTGTTTTCTGTTGCTGGAAGTCCGCCACAAGACTGAGCATCAATTTGCGTGCTAATACTTGACCTGCTTCCCATCTGTTAAACTCATCTGAAA

>Isogroup_74 CTTTCGCCCATGGGTTGAAGAAGCTCGGGACAGTGACCGTGCAGGAGCTGCCCTACTTTGACACCGTCAAGATCACTTGCGCTGATGCCAATGCTATTGCTGAGGAGGCTCGCAAAAACGAGATGAACCTTCGCGTCGTAGACGCAAACACGATCACCGTTGCCTTTGATGAGACAACCACCTTGGAGGATGTTGACAAGCTGTTCAAGGTGTTCTCTGGTGGCAAGCCAGTGGACTTCACAGCCGAATCCATTGCACCTGAAGTCTCAAGCTCCATTCCTAGCAGCCTTGTGCGCGACAGCCCCTACCTAACTCACCCAATCTTTAGCATGTACCACACAGAGCACGAGCTTCTCCGTTACCTGCACAAGTTGCAAACCAAGGATCTCTCACTATGCCACAGTATGATTCCTCTTGGTTCTTGCACCATGAAACTGAATGCTACGGTTGAGATGATGCCTGTCACCGATCCCAAGTTTGCCAACATGCACCCATTTGCCCCTATTGATCAGGCTGCAGGCTATCATGAAATGTTCGACAACTTGGGCGAGCTGTTGAACACGATCACCGGTTTTGACTCTTTCTCTTTGCAACCAAATGCTGGTGCTTCTGGAGAGTATGCTGGATTGATGGTTATTCGGGCCTACCACAGGGCAAGAGGAGACCACCACCGAAATGTCTGCATCATTCCTGTCTCGGCACATGGTACAAATCCTGCAAGTGCTGCTATGTGTGGCATGAAGATTATTACTGTCGGAACTGACTCCAAAGGTAACATTAACATTCCGGAGTTGAAGAAAGCTGCCGAAGCAAACAAGGACAACCTATCTGCTCTGATGGTTACCTATCCTTCAACCCATGGAGTCTATGAAGAAGGCATTGATGAGATATGCAGGATCATTCATGACAACGGTGGGCAGGTCTATATGGAGTGGGGCCAACATGAATGCTCAGGTTGGGTTGACAAGCCCTGGTTTTATTGGAACAGATGTTTGCCATCTTAAC

>Isogroup_75 AGGGTTGCAAGACAGTATGCACATAACCTCAGTGTTGCTGCAAGAACCTTACCATATGAACACGTTTCTTCCTAGGTTGCTTCAACTGAAAGTATCTCAAGCTCTGCCCACCAAAGCGGAGATAATCTTGGTGTCGCTGCCTCCGGTGAAATTGCTATCACTTCTCGCAGTTTAGTGACCACCTCTCTCATACTTGGTCTCTTTTTCGGATCGCTTTGGATGCAATCCTGGATCAACTCGCAAATTACTTCTAGATCGTTTTCTTTGTGAGTCTTCAAGGTAGGATCAAGCAAACAAGATATACTCCGGTCATCCTTAATGCATTCCATAGCCATGTTCATAAGAGAGCCCTTGTGTTCACAATATGGAGGCTTTCCTGAGATGATTTCCAACATGAGTAGACCAAAACTGCATACATTCCCAGCTGGGTCAGCAGGTATTGGGTCATTGTGCTCAATAATGTCATCATTCTTGGGCATCTTTTCTTTGGCGATTACATCTTGCCAAACACTCATGTCTGCTATCTTTGCAGCTCCATCCTCGGACAATAATATGGCACTTGACAGTAGATCAGGGTGTGTTACCGAAGGGTTGAGCTCATGCATGTGTTGGATGCAGTATGCTACTCCCATAATTACTCTCATCCTACCATTCCAATCAATAGGATCGAACCCCTCAACATGGAGATGCTCATAAAGTGTTCCATTGGGTGCGTATTCTAACACCATCATCCTCATGAAAGGATATTCCTCCTCGCAGTATCCAAGCAGGTTGATGAAGTTCTTATGATTAATTCGTGAAAGCGAGTCTATCTTTTTTCCTGAATCTCCCCTCGGAATGCTTTGACCAATCTTTGCTGGATGCGAGCACAGTCGATACAACTGCTATCTCAACTCCACTTGATAAGGTTCCCTTGTAGACAGTGTAGTGTGGATAGGTTGCCACAATGTTACTGAAGTCCTCACAAGCACCTTCCAGCTCTGAGCGTTGCAACTTGGG

>Isogroup_76 ATGGTGAAGCTGGAGTTGCTAGCGGCCAAGAACCTGATCGCGGCGAATTTGAACGGGACGTCCGACCCTTACGCTCTCATCACTTGCGGCGAGGAGAAGCGTTTCAGCTCCATGGTCCCTGGATCAAGAAACCCAATGTGGGGAGAGGAATTCAATTTTGTTGTTGACAGTCTTCCTGTAAAGATAAAGGTGAAAATATACGATTGGGACATTGTATGGAAGAGCACAACACTTGGTTCAGTTACTGTTCTGGTTGAGTCTGAAGGGCAGAGTGGACCAGTTTGGCATACGCTTGATAGCTCATCTGGCCAGGTGTGTCTTCATATTAAGGCAATCAAGGTTCATGAGAGTTCCTCCAGGGTTCTAAATAACTCTGCTGAGGCTGATGCTCGTAGAAGGATCTCCATGGACAAACAAGGCCCCACTGTAGTTCACCAAAAGCCAGGTCATTTGCAGACAATTTTTAGCCTCCCTCCGGATGAGGTTGTTGAACATCATTATTCATGCGCACTTGAGAGGTCATTTCTGTATCACGGTCGCATGTATGTGTCTTCATGGCACATTTGCTTCCATTCGAACGTCTTCTCTAAGCAGATCAAGGTTGTGCTCCCTTTGAGAGATATTGATGAGATACGGAGAAGTCAACATGCAGTTATTAATCCAGCAATTACGATATTTCTTCGAATGGGAGCTGGCGGACATGGGGTTCCTCCCTTAGGATACCCTGATGGAAGAGTCAGATATAAGTTTGCATCGTTTTGGAACAGAAACCACACTATTAGAGCATTGCAACGAGCTGTCAAGAACTTCCATACGATGATAGAGGCTGAGAAACAGGAACGTGCTCAATCTGCATTACGCGCGCTCAGTAGCTCAAGAAAAAATAGCAGGAAGGAGATAAATGTTCCAGAAGAGTGTGTTGATTTAACAGGGCAACTACAACCTTTTGTCAAAGAAGGAGTTCTAGTATCTGTATTCGATGGAACATTTCCGT

>Isogroup_77 ACTCACTTCACGACAGAGGGTGAGGTGGAATTTAGGAGTGTTATTTACATTCCAGGAATGGCACCGCTTAGTAATGAGGAGATTATGAACCCCAAAACCAAAAATATCCGGTTATATGTCAAAAGAGTATTTATCTCCGATGATTTTGATGGTGAACTGTTTCCTCGATACTTGAGCTTTGTAAAAGGTGTAGTTGATTCAAATGACCTTCCCCTCAATGTTTCCCGGGAGATTCTTCAAGAAAGCCGAATTGTAAGAATTATGCGCAAGAGACTTGTCAGGAAGACATTTGATATGATTGAGGAAATCTCTGAAAAAGAGGACAAAGAGGACTACAAGAAGTTCTGGGAGAGCTTTGGAAAGTTCATCAAGCTTGGTTGCATTGAGGACACAGGAAATCACAAGCGTCTTGCCCCACTGCTACGCTTTCCCACTTCTAAAAATGAGCAGGATATGGTTAGCCTTGATCAATATGTAGAGAACATGTCTGAGAACCAAAATGCAATCTATTACATTGCTACGGATAGTCTCCAGAGTGCAAAGACTGCTCCTTTCTTGGAAAAGCTGGTCCAAAAAGACATTGAAGTTCTTTACCTTATCGAGCCAATTGATGAGGTTGCTATTCAGAACTTGCAGACCTACAAAGAGAAGAAGTTTGTTGATATAAGCAAAGAGGACCTAGAATTGGGTGATGAAGACAAGGATAGTGAAGAAACCAAGCAGGAATACACTCTTCTGTGCGACTGGGTAAAACAACAGCTTGGTGACAAGGTGGCAAAGGTTCAAATATCAAAGCGTCTCAGCTCTTCACCATGTGTTCTTGTATCTGGCAAGTTTGGTTGGTCCGCCAACATGGAAAGACTTATGAAGGCACAAACACTTGGTGACACATCAAGCTTGGAGTTCATGCGGGGAAGAAGAATATTCGAAATTAACCCTGACCACCCAATTGTCAAGGACTTGAATGCTGCTTGCAAAAATGAACCCGAGAG

>Isogroup_78 AGTTCACTTCTGGAGGAGGATACAAGTGATGCCGTGGCCCAGATAAACCCAGCCTTGCCTCAGTGGATGTGGGCTTCTCATGTGGCTATCTACTCAAATCACGGGAGAGATGGTTCCCCTGACAGGCCATTGTGGGGTTGGAATGAGGAAGGTAGAGTGGATACCTCCACGCTTAACTTCTCCAAGCTATTTTTGTTTTTGGAGCTACCCTTGACAATACCAAGAAAGTTGACAATTCCAATAGTTGAGGAAGACCGCTGGTCAAAAGAATATGCTGTTGCTAGTGCTGGCTTGGCGCCTGTTCTTTTGGCATTTCTGTGGAACAGTCAGGACGGAGTTTCCACAGGGGCTAGCATAGCAGCATATGTGATAGCTGGCGTCTTCGGGATTGCATTTGCTGCTCTCGGTTACAAATTCACCAGTCCTGATTGCCCTCCAAGAAGATGTTTATTCCCATGGGTGTTTGGAGGGTTTGTTATGAGCATCACTTGGTTCTACATCATTGCCAATGAGCTGGTTGCACTGTTGGTTGCATTTGGAGTGATACTTGGGATTAATCCTTCCATTCTTGGGTTAACGGTGTTAGCATGGGGCAATTCAATGGGTGACTTAATGTCAAATGTAGCCTTGGCAATGAATGGGGGAGATGGTGTGCAGATTGCGATGTCTGGATGTTATGCAGGACCTATGTTCAATACACTTGCTGGCTTAGGAATATCGATGCTGCTTGGAGCATGGTCAACGGCGCCCAACTCTTATGTTCTTCCGCAAGACAGCTCTCTCATTTACACGATGAGCTTCCTCGTTGCTGGATTGATCTGGGCTCTTGTTATGCTGCCACGGAGCGGCATGAGGCCTAACAAGGCACTTGGAGTTGGCCTCATTACTCTTTACTCGGTTTTCCTTTTCATTAGAGTAAGCAATGCTATGGGGATATTTCCACTACCAGGCTTGAATTAATTGTTTGTGTCTATT

>Isogroup_79 ACAAGATCGAAATTGCAGGCCCACTCCAACAGCAGAAATAGCTCTACTCTGGGATCATCTGTTCAGTGTCTGCATCGCCTTCCGTTTCAATCTGAGGTTGCTTGGGAGCACCACCTCCAGGAGCCTGCTTCTTCTTGATTCCACTGACAATGTCGTCAATCCTTAGAAGCATACAAGCTGACTCAATAGCTGTCTTGAATGTCTGTGCTTTTACGCTGTAAGAATCCCAGATCTTCCGCTCTTTCATGTCAACAATATCACCAGTCCTTCCATCAACGCCAACCCAGGGATTTTCACCATTAGCATGCTTTCCCTGGAGTTGTGTCATCGTCCTAATAACATTCAAACCACAATTTTGAAGTAAAGTTCTTGGGATTGCTTCGAATGCTAAGGCAGCAGCTTCATAAGGCCACTTTTCTACACCTTCAACTGAGGAACTCTTTTGCTTTAGAGTTGCCGATACAGTCAATTCAGAAGCACCACCTCCAGGTAGAAGTTTTGGGTTTTTCAAAATGTTCCTTGCAACAGACATGGCGTCCTGAAGGTTTCTCTCCACTTCGTTCAAGACGTCTTTGCTTGCTCCCCTCAATAGAACAGTGCATGCTTTAGGATCTTGGCAGTCAATAATGAAGGAAAAGAATTCATCACCAATCTTCTTAACCTCAAAGAGACCAGCTCGTGTTCCCACATCCGATTCTTGAAGCTCCTCTGGCCTGTTCACTATAACTGCGCCACAAGCCTTAGCAATCCTGTTGTTATCAGTCTTCCTAAGTCTACGGATTGCACTAACACCAGCCTTGCTTAAATAATGAATAGCAAGGTCACTGAGACCTTTCTCTGTGACAACCAAGTCAGGCTTGAATTTCAAAATTTGCGCACAGAGATTCTTTATGTATTCTTCCTCCAACTCTAGGAGAAGCTGCCAATCTTCTTCCTTCATCAACTCAGCATTTGTCTGATTTTCTCCTTTCTT

>Isogroup_80 CTTTTGATGTTTCTCTCCTTACTATTGAGGAGGGTATTTTTGAGGTGAAGGCCACAGCTGGTGACACTCACCTTGGTGGTGAGGACTTCGACAACCGTATGGTGAACCACTTTGTCCAGGAGTTTAAGAGGAAGCACAAGAAGGATATCAGTGGAAACCCCAGGTCTTTGAGAAGGCTTAGGACTTCATGTGAGAGGGCGAAGAGGACCCTGTCTTCCACTGCCCAAACTACCATTGAAATCGACTCACTCTTTGAGGGAGTTGATTTCTACTCTACCATCACCAGGGCCAGGTTTGAGGAGCTCAACATGGACCTCTTCAGGAAGTGCATGGAGCCTGTGGAGAAGTGCCTTAGGGATGCAAAGATGGACAAGAGCACCGTCCACGATGTTGTCCTTGTCGGTGGTTCCACCAGAATTCCCAGAGTTCAGCAGCTGCTGCAGGACTTCTTCAATGGCAAGGAACTCTGCAAGAGCATTAACCCGGATGAAGCTGTTGCCTATGGTGCTGCTGTTCAGGCTGCAATCCTCAGTGGCGAGGGGAACGAGAAGGTGCAGGACCTTCTGCTCCTTGATGTTACCCCACTGTCTCTTGGTCTTGAGACTGCTGGAGGAGTCATGACTGTCCTCATCACCAGGAACACCACCATTCCTACCAAGAAGGAGCAGGTCTTCTCCACCTACTCTGACAACCAGCCTGGTGTGCTGATCCAGGTGTTTGAGGGTGAGAGGACAAGGACCAGGGACAACAACCTCCTTGGCAAGTTTGAGCTCTCTGGTATCCCACCTGCTCCCAGGGGTGTTCCTCAGATCACTGTCTGCTTTGACATCGATGCCAATGGTATCCTTAATGTCTCTGCTGAGGACAAGACAACTGGCCAGAAGAACAAGATCACCATCACCAACGACAAGGGAAGGCTCAGCAAGGATGACATCGAGAAGATGGTCCAGGATGCCGAGAA

>Isogroup_81 GAACTACTTGCACATATGTAGCACACTATCAGGAGGAGAAAGGGTGCTTCTCCAGGGACTAACCCCAGCATATATTACAACCAGAACAGGACTGGGATTTCGCTTGCAAAATACATTCTTTCCCACAAAATGCTGCTTAGTTCTTTGGCAGGATACAACATCAGGTCACCCTTTGTCCTCTGGCTGATTCTATGGTTGCTCGATAAGATGGCTCACATCGCATACACGATCTCTCCACTGCTGTCAATATCAAGATCTGAGTCAGATAGGTCATCCATGTCGTCGTCCATGTCGAGTCCATCATTTAGGATGTGGTTTATACGAGAACAAGCAGGCGGTATGGTGGCCTCTGTTAGAATTTGCATGATCTGGTCCATCGTCTGCATTGGCTGATCAGGCTCCTCATATTGGCTGCTCATACTATCATCGTCTATAAGATCAGCGGGGAGGTTCTTCATGAACCAGCTATGGTTCCGTATTTCAGGAATGGTTATCCTCAAAGCAGGATCCCCAACAAAAATCCTGGAAATTAGGTGCCTGCACTCTGGAGATATGTCCACATTATCTGGAATTGAATACTGAACACTCAGGATACGCTGAATTGTCTTCCGGAAGTTCTTGGGTTCTTCTGGATCCTCAAAAGGATATGCACCAACTACCATGACATAAAGAGTCACACCACAGGACCATACATCAGCGATCTTGCCATCATATTCTTTCTTCAACAGAACTTCAGGTGCAATATAAGCAGGTGTTCCTACAGTTGATTTTGGTTGTGAATGGAGAACTGAAGACTTAGAATAGCCGAAATCACATATCTTCAGGCGAGGAGCAGGACTGCCATCCAACAGTGTGTTCTCAAGCTTCAAGTCACGGTGACATACTTGCATTGAGTGGCAGTAACTGACTCCAGATATAAGCTGCTGGAAAAAGTAACGAGCCTCATCTTCAGTAAATCG

>Isogroup_82 CAATACAAGAACAAGTCAGGTGAAGAACTAAAAGCTCATGCACCATTGACAATTGTATGTGATGGTTGCTTTTCAAATTTACGACGTGCGCTTTGTTCTCCAAAGGTTGAGTCGCCATCTTGTTTTGTTGGACTGGTCTTGGAGAATTGTGAACTTCCTCACCGAAATCACGGCCATGTTATCTTGGCCAATCCTTCACCGATCCTATTTTACCCAATAAGCAGCACCGAGGTTCGCTGTTTAGTTGATGTCCCTGGTCAGAAGGTGCCTTCCATTGCAAGTGGTGAAATGGCAAATTATCTCAAGACCGTGGTTGCACCTCAGATCCCTCCAGAAATCTATGATTCCTTTGTAGCAGCCATTGACAAGGGAAGCATAAGAACAATGCCAAATAGGAGCATGGCAGCTGCCCCACATCCAACACCGGGTGCACTTTTGATGGGAGATGCTTTCAATATGCGACACCCTTTAACTGGTGGAGGAATGACAGTTGCGTTATCTGATATAGTTGTCCTACGTAATCTTCTGAAACCTCTACGCAATCTGCATGATGCCTCTTCACTGTGCAAATACCTTGAATCATTCTATACACTTAGGAAGCCGGTTGCTTCTACGATAAATACATTGGCTGGTGCTCTATACAAGGTCTTCAGTGCCTCACCTGACAAGGCTAGAGACGAGATGCGCCAAGCTTGCTTTGATTACCTAAGCCTTGGAGGTGTCTGCTCGAATGGTCCTATTGCTCTACTCTCTGGTCTTAATCCTCGGCCACTGAGCTTGGTTGCACACTTCTTTGCCGTTGCTATCTTTGGTGTTGGAAGACTAATGCTCCCTCTTCCTTCACCTAAAAGGATGTGGATTGGGGCGAGATTAATTTCGGGCGCATGTGGTATCATCTTCCCGATCATCAAAGCTGAAGGAGTGAGGCAGATGTTCTTCCCTGCTACCGTCCCCCGC

>Isogroup_83 TCGTGGAACTGTGCCCTCCGGGCCTGCTCGCAGACGATTAGGTTAGCAGCTGAGCCTAGCAGCGTGAGATTACCGGCCACCGTGCTCACCCAGGCTAGTATGAGCCAGGCTTTCTTCTCGGAAGCGGGGGAGATCGCACCAGCTGATGCTGCCACTCTAGTGCCCAGCAGCAGCACTGTTGGAACATTCGAAGCAACGTTTGAAAGAACAAGAATGACAACCGCAAGAAGTGCAATCCCTTTGGCGCTATCGATCCTCGCGTGCGGTTCCACCAGCTCCCAGAGCGCGTTGGGTATACCAGTTCTGTTGAAGCCATCCACCGTTATGAACATCCCGCAGAAGAAGATCAACAACGAGTAAGATACCTTCTCGAGGCAAGCTTGTGCGTCGGTGAAATCAAGCGCGAGAAGGACGAGGGCAGCGGTGATGGCGGTCCAGGACATGTTGAGCCCCATGAGGAGCGAGATGAGCATCCCGAGGGTGGTCAGGTAGACGGCCGTCTTCCAGACGAACTCCTTCCACGTCTTGCGCTTCACCTCTTCCTCCTCCTGCGGCTTCTCCTTCTCGTGGAAGGCCTCCTTCTCTGGGGCGTCCTCGATGATCACGCTCCTCTGGTGGCTGGTCGTCCTCGTGACCTTCCGTGGGCCTTCGTCGCGCCCACGGTCACAAACTGTGGAAACCTCCACCATCTCCTGCGACATGCTGGACGCGCGCAGCGACCTGATGGCTACCTGGATGTCGCGGTCACCCTCCGAGTTGTAGCTCCTGCTCCGGAGGGTGTCACCGCCCCTGCTCATGCTGTCACTCCTGCGCATCGTCTCGCTCATGTAGTCGGCGTCGCCGTTGACGGAAGAGGCGTGCGACATCCTGGCCGGTGTGAACCGGTGCGAGCTGACCTCGTCATCGGCGACAACGACCTCATGTCCGGCATCCTCCTGGTCCCTGACCTGCCCGG

>Isogroup_84 AAATAACATGAACATGGTCCAGACTTGGCATATATCATTTTCGACTTGCCTCTGGGATTTCACCTCAACAGGTTTGTAGAATGAAAACCTTCCCTAGAATCCACAACAAATGTTTTAATCCAACATTTTTTTGCTGTTCAAAATATCCATCGGTTAGTGCTAATACTGAACCTGGGGCGGATCTGCTGTTTTATGTCGTCACAAAGCCAAAATGTTGGCTATCTCTAACATATACCAGCAAAATATGTGATTTTGCGACATCAGCCTAGCACATTTCATTGTTTTTTGTATGGTTTTCTATTGTTATATGACTCTACTCAGCCAATGTTGTGCTATGATTCTGCTGTAACTCGTGAGTTTGGGGGGCTATGACACATAAACATTCAAAGGATTTAGCATCCTTGTGCCTATGTGCCTTCCTGTCAGAACAACGAGACACTGAATTATCTCTGTAGCTCACATAAGCCGTCACAACCTTAATTTTAAGATATTACAACAAACAGATCATCAAACAGCCTGAACAGCTGAGACACTCTTGTCTATGTTAGTAAGCCTTATGTTGTGCATATCCATAAATACTTACTTTTAAAGCACCATGGACTCTACTTCCACATTTGGCTATTACAAGAGCAGTTTATGGTTATTCTGAGATATGTGAGTTCCTAAAGTTGCTTGTGCTGAGGTTTTGGAAGAATAGCTTCCGTTAAGAAACAGTCATTTCATAACTTATTACTTTGTGTTTAATTCTTGTAGATAAGGTGGAGCGTGCTGTGAGTAATGCATTATATTTCCTGCTACCTGCTGGTGAGATTGCCCGGCTGCACCGCATCCCTTGCGCTGAGACATGGCACTACTACATGGGGGAGCCTCTCACGATATTTGAGGTGCATGATGATGGGCAAGTAAAGATGACAGTCGTCGGTCCTGATCTACGTGAGGGGCAGAGACCGCA

>Isogroup_85 CTCCTTGGTTTGCTGAGTGTGTGGAATGTTTCATTTCTCGGATATCCGGCTAGAGCAATATTGCCGTATTCTCAAGCACTCGAGAAACTTGCACCACATATTCAGCAGCTTAGCATGGAGAGTAACGGAAAAGGTGTTTCCATCGATGGCGTTCGACTTCCATTTGAGGCTGGTGAAATTGATTTTGGTGAACCTGGAACTAACGGGCAACACAGCTTCTACCAATTAATTCATCAGGGAAGAGTTATTCCTTGTGATTTTATTGGCGTCATAAAAAGCCAGCAGCCTGTTTACTTGAAAGGGGAAACTGTTAGCAATCATGATGAGCTGATGTCCAATTTCTTTGCTCAGCCTGATGCTCTTGCTTATGGAAAGACTCCTGAACAATTACGCAGTGAGAACGTTTCTGAAAATCTTATCCCTCATAAGACTTTTCAGGGCAACCGTCCATCACTGAGTTTCTTGCTGTCCTCATTATCTGCCTATGAGATTGGACAGCTTTTATCCATTTACGAGCACCGGATCGCAGTTCAGGGTTTCATATGGGGAATCAACTCGTTTGACCAGTGGGGAGTGGAACTGGGCAAGTCACTGGCTTCTCAAGTGAGGAAACAGCTGAATGCATCACGGATGGAAAGAAAGCCCATCGAGGGCTTCAACCCCAGCAGTGCAAGTCTGCTGGCCCGGTATCTTGCGGTTGAGCCATCCACTCCATACGACACCACGGTGCTGCCGAAAGTGTAACCTGGTGCTGAGATCGCTAGACAAGCTAGGTTGAGCTTGTGGCCATGACAAGTTTTCTTCCTTCTTTCTTGCTGAGTTGTCACAACCAGTTTGGTTGCACTGTAATTTTGAAGTTTCTCTTGCTCCGGCTTGGAGAAAGATAAGTGAATCATCACGATAATGAAATAAATACGTTGACCCATAACAGCAAGGAAAAAAAAACTTTG

>Isogroup_86 ATTCTTGGATAAAAGCAAGCATCCTCCTTTAGTAAGGATATAACAGCAGCATTGTTCGTATGTATCATTGTGTTAAGACTTGCCATAGTGGCATCATCTAATATTCGCGGTAATATAACATCCTTCAGATAGCCTATTCTGTATGTTTGATGTATCCTCGAAACCACAGAAATATTTTTTATAGGTATGGCCTCTTTGAAAATCACATGATCCTTCAGAAAAAATGCGATGCTTTTGCACTTTGGCAACCTCTGGATCATATTCAAGGGCGCCTATGATGTCAAGAATAAACTCATTGGAGAATATCTTATCAAAGATTGATGGGCTGTTCAACAATATGATTCCCCTGACTAACTTGAAAATCATGTGAAGATCATCTAAATTCTCTAAATCCTCGCACATTCTAAAAATCTCCAGCAGCTTCGGGAAAAAGTCACGATCTTGTGTAATCAACTCCGCCACACGCATTTGATCTGTAATGCCACCCTCCAATATAGTCTTCAAGATCAAAGGAAGATTGGACAAATCAACAGGTGGAAGCTCCCTCAATTCACCGTTAACAGAGCGGTAGGACTCGTCATGTGAATGCAATACTCTAGAGGCTTGCAGATGTTCCGATGGTGGGCGAGGACCAACTTCAAGAGCACCAAGGTTACTAAACTGAAGGTTTCTCTGAATATCACAGATGTTGTCCCATATGTAGGAGCACCCTGCAGCTTCCTGGAAGCTCAATGCTAATTCTGTTGCTGCTTCTGGATCCCTCCATGAGATGATTGTTTCTTCTTGCTTTCTGTAGACATCTTCAGATGTAATATTGTGGACAAGCAGTGTTTCATTGTCATCTTCATCCAAAACAGTCAAACCAAGATCTTTTGAACCCTCCACATAGTCAATAGCAACATGTCCTGTGCCCTGATCATCCCATTTCCCTCCATCCCTCAAACG

>Isogroup_**87** ACTACATGACCTTCTTCACATTGACGTTAGTGTTAAATGCTTGTTGTCATGGGATGATCGTATTAGGATTGCAGTAGAGGCAGCAGGGGCACTTGCTTATCTGCACTCGGCTGCAGCAATCCCAATTTTCCATAGAGATGTGAAATCTGCTAACATACTTTTGGATGGCAGCTTTGCTACAAAGATTTCTGACTTTGGTGCTTCAAGGGCTGTTTCACTCGATGAAACTCATGTGGTGACTACTGTCCAAGGAACATTTGGCTACCTAGATCCAGAGTATTACCATACTGGGCAGTTAACTGAAAAGAGTGATGTATACAGTTTTGGAGTAATACTCGTTGAACTTTTGACAAGGAAAAAGCCTATTTTTATCAATGATCTAGGTGGAAAACAAAACTTGTCCCATTATTTTGTTGAAGGATTACAAGAGGGAGATGCCATGGAAATAATGGATCCCCAGGTTGTCCAAGAGTCAAATAGAGAAGAGATTGATGATATTTGCTCACTTATAGAAGCATGCTTGAGAGTCAAAGGAAGAGCTAGGCCTACTATGAAAGAAGTAGATATGAGGTTGCAAGTCCTTAAAAGTAAGAGGATAAAGAAATGCAGAATTCTCCCAGCCAGTGATGAAGAGATTGAGCATTTTTTGTGCCCAAATGTTGGCCATTCCAATGCACCGTTGAATATTGTCAAAGCTGGTAATTTAAGATCAAAAGGCATCTCGAGTTGCGATAGGCTAGAGCAAGAGCATTCATCCTCACTCACTATGCCACGCTAAGTATATATGTGCAATACCACAATCGTGTCCTTCCTATTATTCATTTGAATTACCATTTATTTTGTATGGAGTTGTATTATGTTACATAGTTCACTTTAGGCGAGAGTATGTCATGTAGTTTCGCTTCCTGTATATTTGAATAATTTAATTGTATCCGATGAATTGT

>Isogroup_88 CATGTAATATTACACAAAGAAGAAACATGCAAGACCGGCCGATAGAAGCCTGCATGTTCCAACTTGACAGCAAGTGAATGCCCTTGGATGATAACAAGACAAACTCAAAAGTAAAATCCACACATACGTAGAGCAGATGGCACAATAGATAAGGTTCCGCAGGATCTTGTGCCTTATTGTATCAAACCAGATAACGATCCGCGGGATCTAATGCTTTATTGTATCAACCCAGTTTAACCACATAAGGCTGCGGCGATGGCAGACTTCCCACGGGGGCCACCAATCGTAGCAATCACTGAGGTTCCAGTCTCCTTGTCAATGACTTTAGCACATATTCCAGCCTTATTCAAGTTCTGTTCAACCTTGTCCCAGTTGATATTATTGAACTTAATCTTACGGCCACACTCTGCATAAATCCTCTGACCCGTAGCCTTGGTGTCAGTGAAAGCAAGGAGCCATCCACATTCAACACCAGTTTTGTTCTTGCCGGAATACACCACAGCAGCCTTCACACCCTTATCAGACATGGCTGACATGGCAAAAGCACCAGTGCCGAGAGTGCCAGGGAAGCCCTTGACAATGTGCCCTGAATATTTCTTGCTATACATAATATGCTTGGGCTCTTTTCCTTGATTCCTCAAAACACCTCCTACTACGGTAACTTTAAGCCCGGCAAACTGTGGCCCGGCAAACTTGGGCTGGGTAAGGCCATCATTTTGGGCAACCACGGCATGACATTCATTCATAAGTTGCTGAGACTCCAAGGAGCACTCAGCTTGTTTCAGGATCTCATCTAAAGTTTCGGTCTCCTGAAAACTGAGTTGCACCGCGATTTCCTCAATCCTTTCACACTGGCTTGATGCCATTTCCTTGTTTCACTAACTTAGGGATGAAGAGGTTCTTCTAGAGTGCAGAGAGTTCCTTGCCTCTTGTGTGAATGGAAT

>Isogroup_89 AGGAACCCAAAGGAGTTTGCCAATGATGTGAGGCTAACATTTCACAATGCCATGACCTATAATCCCAAGGGGCAGGATGTGCATTTCATGGCTGAGCAGTTGTTAGGAATCTTTGAAGCTCAGTGGCCTGATATTGAGGCTGAGGTTGAGTATCTTGCGTCGTGCCCGCCGATGCAGAAGAAGTTTTCACCTCCCCCACTTGACTTGCGCTTGCTAGAGAGGTCAGATTCCTTAAGGCACCATATGGCACTGGACTCCAAGTCAAGGCCATTAAGTCATACTCCCACTTATGGTGCTCGCACTCCATCATTGAAGAAGCCAAAGGCGAAGGACCTAAACAAGAGAGATATGACAATAGATGAGAAGCGTAAACTTAGCAATAATCTTCAGAACTTGCCTCCGGAAAAACTTGACATTATTGTGCAGATCATTAAGAACAAGAATCTTTCAGTTAGGCAGCATGAAGATGAGATTGAGGTTGAGATAGATAGCATGGATGCGGAGACACTTTGGGAGCTCGATAGATTTGTGGCTAATTTCAAAAAGAACCTGAGCAAGCAAAAGAGGAAGGCTGAGCGTGCAATGCTTGCAAGGCAAGATGTGGAGTTGCGTGCACTGCATGCTGCACAACAACCAAGTCAGCAACCTACTATTGGTGAAAAATCTCCAAAGCTAAATTTGATGGTTAGCGAGCAATTAACAACTCCTGTGCCAGAGCAAAATAATAATAATGGACCAAATGCAACTAGATCTAGCAGCTCAAGCAGCTCAAGCAGTGATTCAGATTCCTCTTCTAGTGACTCGGACAGTGACAGCTCTTCCACAGATGGATCAAATGCTGCTGCCAATTCATCTTGAAGCATAGGTTCAGGAACTCCATCCATCCAGTAGACAATAACTGTCTAATTGTATTTGGCATGAGAAATGCCTCCTTAAGACTG

>Isogroup_**90** GCCGGAACGTCTGGCCCTGCGTTGGTTCTAGTTCATGGTTTCGGGGCAAACAGTGACCATTGGCGGAAAAATATTCCTGTTCTTGCCATGGAAAATAGAGTTTATGCAATTGATCTTATTGGTTATGGCTATTCTGATAAGCCTAATCCACGTGACTTTGAAGAGAGCTTCTATACTTTTGAGACTTGGGGAGAACAGTTAAATACATTTTGTGCTGAAGTCATCAAGAGTGACGCTTTCTTCATATGCAATTCAATCGGAGGGCTCGTTGGTCTGCAGGCCGCTGTTATGGAACCTCAAATGTGTAAGGGTATTGTCTTACTGGATATTTCACTAAGGATGCTTCATATTAGCAAGCAGCCATGGTATGGAAAGCCTTTCATCAGATCATTCCAAAACCTCCTAAGGAACACTGTTGTTGGAAAGCTCTTCTTTAGTTCTGTTGCAACACCAGAGTCCGTGAAAAATATTCTTTCTCAGTGCTACCATGACACATCCGCTGTCACAGATGAGCTTGTTCAGTGTATTCTGCAACCAGGGCTTGATCCTGGTGCAGTGGATGTATTCCTTGAGTTCATCTGCTACTCTGGAGGTCCTCTACCTGAAGATCTACTTCCCATGGTGAAGTGCCCAGTTTTGGTAGCTTGGGGGGAGAAGGATCCGTGGGAGCCTGTGGAGCTTGGAAGAGCCTATGGATCTTTTGATGCTGTTGAAGATTTTGTTGTCTTGCCAAATGTTGGACACTGCCCGCAGGATGAAGCACCTCAGCTTGTTAATCCACTTGTCGAATCATTCGTTAAGCTCCATAGTTAGCAGATGGTTGGATTATTTTCTCTCTTTATTAATGACCTTGGTAAACTGGAGCCAAGAGTTGCTGGAAGATGGGAATGTCTATTTTATTGCAGGATAACAGATGTGCCGTTCTTTTATTGGCCATG

>Isogroup_91 GTTGTGTTTTCAACAGAACAGCTATCTATGGTTATGAACCCCCAATTAAGCCAAAGAAGGGTGGTTTCTTGTCATCACTGTGTGGGGGCAAGAAGAAGGCAAGCAAGTCGAAGAGAAAGAGCTCAGATAAGAAAAAGTCGAACAAGCATGTGGACAGTTCTGTCCCAGTATTCAATCTCGAAGACATAGAGGAGGGTGTTGAAGGCGCTGGGTTTGATGATGAGAAATCAGTTCTCATGTCTCAAATGAGCTTAGAGAAGAGATTTGGCCAGTCTGCAGCATTTGTTGCCTCCACTCTGATGGAATATGGTGGTGTTCCTCAGTCTTCAACTCCAGAGTCTCTTTTGAAAGAAGCTATCCATGTCATTAGCTGTGGCTATGAGGACAAGTCTGAATGGGGAACTGAGATTGGTTGGATCTATGGATCTGTTACAGAAGATATTCTTACTGGATTCAAGATGCACGCACGAGGCTGGCGGTCAATCTACTGCATGCCTAAGCGCCCAGCTTTCAAGGGATCTGCCCCCATCAATCTTTCAGATCGTCTGAACCAAGTGCTCAGGTGGGCTCTTGGTTCTGTGGAAATTCTTTTCAGCCGGCATTGCCCCTTATGGTATGGCTACGGGGGACGCCTCAAGTTCCTAGAGAGATTCGCTTACATCAACACAACCATTTACCCACTAACGTCGATCCCGCTTCTAGTCTATTGTATATTGCCTGCTATCTGTCTGCTCACTGGAAAGTTCATCATGCCAGAGATTAGCAACTTGGCCAGTATCTGGTTCATCTCTCTCTTCATTTCAATTTTCGCCACTGGTATCCTTGAGATGAGGTGGAGTGGTGTTGGCATCGATGAGTGGTGGAGAAATGAACAGTTCTGGGTTATTGGAGGTATCTCTGCCCATCTGTTTGCTGTCTTTCAGGGTCTTCTGAAGG

>Isogroup_92 TACTTATTGATGTTCGGCCTGAGGACTTAAGGGTGAGAGATGGAGTTCCTGACCTACGCCGTGCAGCTAGATCTAAATATGCAAGTGTTGCTTCACCTGAGATTGAAGGCCCAACAAGGAAGCTACTTAAAGGTGGAAGCGATGTTGATGATGCTTTAGTTGCAGTTGTCATCCGCAACTTGAAGTTGGTTAAGGGTGATTCAAAGGTCATAGTTATGGATGCTAACGGAACCCGATCAAAGTCCATTGCTAGGTTACTGAAGAAGCTTGGTGTGCAGCAACCTTACCTGGTTAAAGGTGGTTTCCAATCTTGGGCGAAGAACCTTCGTGTGAAAGAACTGAAGCCTGAGACTGCATTAACAGCACTAAATGAGGATGCTGAGGAAATCCTCGAAGGCATAAAGCCCACTCCGACATTTGTTGTTGGGTCTCTTTTGGGCCTCTCAGTAGCGACTTATGCTTTGCTAGAATGGGAAACGACTTTGCAGTACCTCGGCCGCGACCACGCTGAGCGTGGTCGCGGCCGAGGTACGACCACTTGCATAGAGGGAGAGCACGGCCTGAATTGCGACATACATGGCAGGAACATTGAAAGTCTCAAACATAATCTGGGTCATCTTCTCTCTGTTGGCTTTCGGGTTCAAAGGAGCTTCAGTGAGCAACACAGGGTGCTCCTCGGGTGCCACACGGAGCTCATTGTAAAAAAGTGTGATGCCAGATCTTCTCCATGTCATCCCAGTTACTCACAATACCATGCTCGATTGGGTATTTCAGAGTGAGGATACCTCTCTTGGACTGTGCCTCATCACCAACATAGGCATCCTTCTGTCCCATCCCTACCATAACACCAGTGTGGCGGGGGCGGCCAACTATGCTAGGGAAAACAGCCCTTGGTGCATCATCTCCAGCGAAAACCAGCCTTGACCATTCCGG

>Isogroup_93 CTTCTTTTGAATTGTCCTATCAGTCATGTCGACGTCAACATGAAAACCAGCTGCACGTATCTCAGCATGAACCTTTTTTCGCATATTCTAGTGAATTGGAAGATACACAACAAACAATGGCTTGGCGAGGGCTTAACCACAAAGGCCATTTACCATTGTAGTGCTCCAGAAGGATGGCAAACATCCTTTCAACTGATCCTAGTATTGCCCTGTGTATCATCACAGGCCTCTCAAGCTTGGCTTCATCCTCTGCAGAATAAGTCAACTTGAAGCGACTGGGCAATTGAAAATCAAGCTGCAGAGTTGCACACTGAAATTTCCTCTTGAGGGCATCAAACACACCAATATCTATTTTTGGACCATAGAAAGCACCATCCCCTTCATTGATCTTCCATTCCTTCCCAAACTCAACCAATGCTTCAGTCAGTTGTTGCTCTGCTTTGTTCCAGGTCTCAATGTCACCTAAATACTTCTCTGGTCTCGTTGATAACTCTAACTCATATTTAAACCCAAATATTTTATAAACATAATCAACGAATTCCAAAGCAGCCCGAACTTCATCCTTGATTTGGCTCTCCGTGCAAAAAATATGGGCATCGTCCTGCTGGAATCTTCTAACACGTGTCAAACCTGTAAGTGCACCACTAAGCTCATTCCTGTGCAGAACCCCAAAATCGGCCATGCGGAGAGGCTAACTCTCTATATGATCGAACCTTGTGGCCAAACATTAGGCAATGGCCAGGACAATTCATTGGCTTAAGGCCGAATTCTTGTTTCTCGATCTAAAAAACGAACATGTTGTCCTTGTAGTTTGCAGCATGTCCAGAGGTTTCCCAAAGTTGCATATTGTAAATATTTGGGCTCAAAACCTCCTGGTAGCCTCTCTTTATATATTCCTGTCGCATAAAATCTACCAATTTGTTATATATTATA

>Isogroup_94 CAGCCTTTGGAAGTAGCTACAAGTTAGGAATGGAAGCTTTCCATGCACAGAAAGAGCTCCAGGCAATTGCCATGGCCAGTATACTCAATGTGCAGATACCAGGATTCAGCTACCTGCCCACAAAGAGGAATAGGCGGAAGTGGATGCTTGAAAAGAAGCTTAGAAGCACGCTCATCCATGTCATAAACTCACGGTTAGCATCACAAGGGACCGGATATGGAAATGATCTGCTTGGTTTGATGCTTGGGGCTTGCACTGCAACAGACAAAGGGGGAGAGCTCAGCTTGAGCATGGATGAGATCATACACGAGTGCAAGACGTTCTTCTTCGCGGGTTATGAAACCACATCAATTCTTCTTACCTGGACTGTGTTCTTGCTCAGTGTGTATCCAGAATGGCAGGAGAGGCTACGGAAGGAGGTCTCGAGGGAACTTGGAAAAGATGACCCTAGTGGTGATAACCTTAGCAAACTGAAAGAGATGACAATGGTCCTCCTTGAAACCTTGAGGCTCTACGGCCCTGCTCTTTTCATGCAACGGAAGCCTATAACTGATATGGCAGTGGGAGACATAATAATACCCAAAGACTATGCAGTGTTCATAGCCTCCCCATTTATGCACAGAGACAAGAAGATATGGGGTGACGATGCACATGAGTTCAACCCGTTAAGATTTGAGAATGGGGTCTCAAGAGCAGCAAAGGTTCCACATGCCCTGCTAGCATTCTCGATAGGGCCGAGGGTGTGCATCGGTCAGAATTTCGCGATGCTGGAAGCCAAGTCAGTAATGGCCACGATTCTACACAAGTTCTCCTTTGCTCTTTCCCCAAGATATGTGCATGCTCCTGTGGATTTACTGACTCTGCAGCCCAAGTTTGGTCTCCCTGTTGTTCTGAAGCTACTGGATGTATGAATCGGACTATTTCGAATACTG

>Isogroup_95 ATGACAACGAATTTTCTAAAATTAACGAGCTTGTGCAAACCCAGGAAGTGCTCAACCCACCAGAATTGGCTCATCTACAAGATCATGGTTTGAATGAGACTGGTGAGGGAAGCTATGGTGACAGTGCTATTTCTCTCGAGGAGATCTTGCAGGAACCTTTGCCAAATGTCAGTCTAGAAAATATAGGTGTGCCTGAGGAGCAGAATGCCATCGATGATCACTTCAGTCTTGCAGATTTGTCTGGATACCCAAGTCAAGATGATGGATATGCAGGTCAGGATGGGCCTATCCATTGGAGTGACCCTGTAAATGGTGATCAGGCACAGTGGCCTCTGAGAACTTATGGTAGTCAAAACCAGGCCAACGGGACTCTTAGTGCTGATGAGTTCTTTGACCCAGGGAATGATACCAATGCATATTCAGGAGGGCAGCAAGCTTGTCCTCCAGATGACCAGAACTTGTATTTGCAAACCAATGGTCTGCCAGGTCCTCAGCAAGTGGATGACAACATGGCGTTCTATGATGCATCAAGCAATCACAAATGGGAGGATAGAAAGGACGATTATCTAAATGTGAATGGCCTCATCTACCAGCCAATTGACGACGAGTCTCTCTTTGATGTGGGAGATGATCTGATGGCTTTCTTCGATGCACCAGAGGGTGATTTCAACTTGGATCTTTTGGGCCCAGTTGGAGGCTCTGATTCTCAACTTCCAGACATGTCGAACTTTGCTCAAAAGGATGAAAAATTCGACCATTTTATGTTCGATGGGATCTCCAAGACTTCAAATGCAAATGCTCAAAATGGCGCATCTTCATCTGGCTCCCAAGGGAATCTATATCCAGACACTGCTCTTTCAGGTGTGCCAATGGATGATAAGGTTGACAATAGTATTGGAAAGCGCCTTGCCAACATGCTGCACTC

>Isogroup_96 TGGAGGCGAGTGGATCAAGCGATTGGATACTCCTATCTGTAGTCCCAGGCCTCGCAGTGTCAACTAGAGCAAGGGAAAGAGTTGTATCAATGTTGTCACCATCTTCATCCAACCGAATTGCGGCCATTGTAGAAAGCAACTTCATGGAAGCTGAACGAGCAGTTTTTTGTAATTGCTTTTATGTCTTCCTTTCCTGTCCATACTCTTGGCATGGAATCAGCATCACGACTGAATAACATCGTAAACCTGTCCTTCATGCGGATCAAGACCCTTGCAGCTTCTTCTTTTGCCTTTGACTCAACAACGCCTTTTCCATGATTCTCCAGCCTCGAAAGGAGTTCTTTCTCAGTAGCTTCATCGAGCTCAAAAGTTGAAATGGCAGATTCAAGACCTGAAACAGCAGCTTGTGTCTCACGTTGAAGAAGCTTTCTAATAGCTGGCCAGGTGTCCTCACTGGCTGAATCTAGAAGAGCTTCAACAGGTTCTGCCAGAGCTTTGGTAAGTTTCCCCCTCATATCCAGCACAAAGTTCCGAAAGCTTTGTAGCACGAACTGACACGACATGCGCCTCAATGTCACGCTTAAGCTTATCCTTAACTTTCGAGGAGTCCCAGTTCACTTGTTGAATAGTAGCATCTTCAGATCCTAGGTCGAACTTCTCCAAGAAAATGCGGACAGAATTGCGAGCAGCAACAGCAAATCCATCCTTTTCAAGGGCCTTTTCAAAAGAATCCTTAAAGGCTTCTAAAGTTCTAGCCCGTAAATGACCCAGAAGAGATTGGTATGCAGGATTGACAAGCTGCAAGAGTTTAGACTCAAGTTGTTGTCTTTTTGATGTTCTGACACCTTCATCAAAATAAATAGCTTCCATGTCATACTCAGATAAACATCTATCAAGAAGGCTGCTGATCCTCTTCCCAAACCCCCCG

>Isogroup_**97** CCACCCTTTATCCTGCTCAATGCAGGATTCTAAGCTCACCGACATTGACGAGTGCTTCCGATTTCCTCACCTCCATACTTGGCCTTCCTAGAGAAGGCCCGTTGTAAATATCACAACACAGCAGATTAAATCAAACCACAGTTGGCTGCAGCGCTTTTATTGTTCTCCTCTGCCATGTGTCGGTCGCGGGCTCAAAGGTGAGCTCCCAGGTCGGCCAGTAGTGAAGGTCCTGTTTCAATGTCAGCACACGAGGTTTTCCATTCAGATGGACGGTTTTCACCCTAACAAATTCGCGGTCTTCCAATCCCTTCACCACTTGTATAAAGGACTCCAGATCTGGAGTTGGTTTCCCATTAATTTCAACTATCCACTGGAGAGCATAGAGACCATATCTATGCACGGGGCTTCCATGACACCATCTAGCAACATAAACTCCATGACCTTCCTCTGGCAAAAAGCCAAGTGCACGCACTGCTGAATGAGGATCCTGAATTATAGATCCACACCAATTCACCATCCGTGTTGAACCATTACCATCTCTTACATCAGTTCCAACAATAAGGTCAATTTCTTTTCCCTGACGGAATATTGTCATGTTAAGCACTCCATCGGAATCGATGGATTGGTCCAACTTTTGGCAAGCATTCTCTATATCAAGGAAGCATGTTATTGGTTCCTTATTGATAGCAAGAATCATATCTCCCTGTTCCAGAAGATTTTCCGCTTTTGATCCAGCCAAACAACCTTTGACCCGCAAGACCTGTCTTCGCACAGGGTCCTTCTTAGCGAGAGCCTGCACCCAGTTATCACTCAATCCATAACTTCTTGCCTTTGAGAGCAAAGTTGGATAAAGTTCCACCTCCAGAAGTCTGACAAATGGCATCGGTCTCTGAACTCCATTGATGAGACGAAATGGTCCAGGA

>Isogroup_98 GCCTGGGCAGGTACCTCAAGATCGCTATATGGAATCACTGAAATGTAATTACTCTATTGGGATGAGATTTAAGATGAGGTTTGAAGGTGAAGAGGCTCCAGAGCAAAGATTCACTGGGACTATAGTTGGAATTGGCGACTCTGATCCAGCAGGCTGGGCTGAATCACAATGGCGTTCCCTTAAGGTGCGATGGGACGAAGCTTCTTCCATTCCGCGTCCTGAAAGAGTTTCTCCCTGGCAAATAGAACCTGCTGTTAGTCCTCCTCCAGTCAATCCGCTTCCAGTGCCAAGAACCAAGAGACTCCGTCCAAATATTATAGTTTCTGCACCTGACTCTTGTGCTCAGACAAAAGAAGTTGCTCCCAAAGTAGCAGTGGAGACTCAACAACATGCTTTACAGAGGTCTTTCCAGACTCGAGAGAATGCAAACTCAAAATCTCCTTTTGGCGATGGCAGCGAGTTGGACAATGCACACAAATCAATCCTCTGGCCTTCAGGATTTGAACGTGAGAAGAATAGCATACCTACACAGAGAAAACTAAGTTCAGATGGTTGGATGCAGATGAACAGACCTGAAAGTTATGGTGAGATGTTATCCGGATTTCAGGCACCTAAAGATGCACAAACTCAGCACGGTTTCTGTTCTTTACCTGAGCAGGTTGCTGCAGGACATTCAAACTTCTGGCACACAGTCAATGCTCAGTATCAACATCAGCAAGGCAATCACAACACATTTCCTGGTTCATGGTCCTTGATGCCTCCAAACACTGGCTTTGGGCTGAACAATCAGAGCTACCCAATGATGCAGGAGGTTGGTGGGTTGCCTCAAAGGGCTGGAAATACGAAGTTTGGTAACGGAGTATTTGCTGCACTGCAAGGCCGCGGCATAGAGCAATACTCGGCAGGATGGTTTGGCCACATGG

>Isogroup_**99** TTAAAACTACTATATGAATTTGATCCCTAGACATTTTGCTTATGGGAATGTTACATCATGTAGTAGAGAGGCTTGTGAATTTGATTCCTGAATCGCTAGAGCTGTAGAGCAAGAACTGTTTGTATGAGCTGAAGAAATATAAGTGAAGATAGGCCCGAAGAGGAGTTTCTGATAGCCTATTGAGAGCATGTTACACTGATCATGATGCTTGAGAATCAACATAGCTATCATAAAAATGACAGCTGACACTTATGGCCAGACGAACTTCACTCAAACCAAGTCATATTCCAGCGCAACAACGATGTCTTTACTTGTCCGTCTGATGATATCATGGACTCGGATCTTCAAATCTATTGTCGAGAGTTGAGCAAGGCTTTTGTCCAAGCATCCCCAACCAAGTTTAAGAAGGTAATCTTCCAGCTGAGGTAGAGTGGAACCGCACTCTCCATCAATGATCTCCATAACATCTCCTTCTCTAATGACATTCCTCCAAGCAGAAGAAACAGAACGCACCTTATTAACAATAAAACCCCTTTTGATGTTGTAGTGGACTGAGAGCTTTTCTTTGTGCTCAATTTCCAGCAACTCCACCGACCTCAAGTTCATATCGATCATAGGGCGAGCAATGCGGCTGAACTTATCCAACATGTCAATGCATGTTTTGACCGTTGTGATAGACATAATAGCTACTTGAAGATTATGAGCAACAACCATTCCTACCATATTACCATAATTGTCAAGAACTAGCCCTCCTGTGCAATCCAGGTCCTGGAGAGCGCCAGATTCACAACTAAGGAACATATAATAGTTACATCGTAAAAAAGAATGCTCCAACAATGAGATCGATCCACGTTTTACAACTATGGAACACTCTTCATCTCTACCCAATAAATAGACCTCCTGACCATATTGTGGCAT

>Isogroup_100 ATATCTGGCCCTGGAGCTTCAGCTATAGTAAGGCAGGCTGTTTTCATGAACATGCTGAGCATGTAAGGGCCAGGTTTCCGAAGCAGAAACTATACTCTGTGTCCTGCATATGCCGCACTAATTCATCTAGTCGAATTCGGTGGCGTGATCCCTTGATCTAAGTAATACTTCTTCTCGGCATCGGCCAACCTTGCCTGGAATATCCCCCATTCTTTTCTACAGCGTCCCCTTACCCCTTCCCATGGTGCTCTTCCATTCTCTGACTTGCCCTGGTTACCAAGTGATGGAACACTCTGATGAACAATCCACTGAGCATCAACAACTCCGATTTTCTCATGAGCTGGCTCCACACACTTCCTAAGAGCAAAATCAAGACCCCATCCATGGACCAAGTCGTTCTGAATCATATGCCACACACAGCGCCATGCATCCCTAGAGAACACAGTCGCCATAATTTCTACGAATGCTGCACAGGGTGGTAGATGGGGATCGGAACACCAGCCTGGCCTCTCCTCAGTTACTTTGTGAACTTCTCGATCACCTCGCCGCTTAGTCATTTGCCATGTCAGACCTCTATCAGGTTCCAAACCAGGTTGAGAGATCTCCAGCCCATGCTTCCTAACAAGCTTGATGTATGCCTCTGCATTAAAATGTTGAACACCTAGATCCTCGTCCCAGATAAATATGTAGTCATAACGGGCAACAACATCAGGATGTAAAAATCGCTTGGCATACCACCATTTAGTTTGCTTGCTAACACTCACATGGATAGCCCTTTTTGACCAGTCAAATTCATCCCATTCAGTAGTCCGACCATCATAGTGGAACAGCATAATTGTGAAGTTCTCCGAGAACTTCTTGACTGCTGCATCAATATTTGCCTTTTGCCCAATTCCAACTGTAAATGTAACAAGG

>Isogroup_**101** TGTAGCAATCAAAGTTCTAAATCTTGGCAAACGAGGAGCTACCAGAAGCTTCTTGACAGAGTGTGAAGCCCTAAGGAAGATTCGGCACCGGAATCTTGTCAAGGTGATCACTGTTTGTAGCAGTTTGGACCGTAATGGTGATGAGTTCAAGGCACTTGTCCTGGAATTTATCTGCAATGGGAATTTAGAGGAGTGGCTGCATCCAAACACAACGACTAACAGGATGACCTTTAGAAGAGTAAGCTTGATGGAAAGGCTATGCATTGCTCTTGATGTTGCGGAGGCATTGGAATATCTTCACCATCAAATTGGGCCGTCCATAGTTCACTGTGATATCAAACCATGCAACATCCTTCTAGATGATGACATTGTCGCACATGTTACCGACTTTGGTCTAGCAAAGATAATGCATACTGAAGAATGCAAGCGCAGTGCTGGTGGAACTGAAAACAGCTCACTTATGATTAAAGGCACAATTGGATACGTCGCACCAGGTTAGCAATGTTTTTATATTGGAACTAATACTGTTGCAGTTCAATAATTTTTAGCCGGTTCATATTCTTTGATAAACTGATAATGAGGTTAGTGTATCCTAAAGAGCTAGAAAAATGCAATGTCATTATAATATTTAACTAAATTGAATTCTCGAGTGCAATCTGCCAAATTTATGTCTTTAGTCATTGCATAGCATTATCCAGTAACATAGTTTTTTTTATATGTTTGTCAGAGTATGGCTCGGGATCTGAAGTCTCCACAGCTGGTGACGTATATAGCTACGGTATCTTGCTGTTGGAAATATTTACTGGAAGAAAGCCAACTGACAGTTTCTCAGATGGTGTGACAAGTCTCGTCAGCTATGTCAAGATGGCCTATCCTGACAATCTACTGGAAATACTTGATGATAGTGCAAC

>Isogroup_102 CAGGTGGCCCATATTCCTGGCGCGCTGTTCTTCGACATAGATGGCATTGTCGATCGGACAACTGATTTGCCACACATGCTGCCATCAGAAGAGGCTTTTGCAGCAGCAGTTTTCCGCGCTTGGTATAAAGAACACTGATAAAGTTATTGTTTATGATGGAAAGGGCTTCTTCAGTGCACCACGTGTTTGGTGGATGTTCAGAGTTCTTGGACACAATGAAGTTTGGGTCTTAGATGGAGGTTTACCTCAGTGGCGAGCTTCTGGATTCAATCTTGGAAGCAACTGTCCTGATGATATACTTCTGAAATCCAAAGCTGCCAATATCGCTGTTGAAACAGCTTACAATGGTGAATTGACAAATGCTGCCACATTTCAGACTGAATTTCAACCTCAGCTATTCTGGACGCTGGAAAAGGTAAAAAACAATGTAGCTGCTCAGGCTCACCAAGTAGTTGATGCACGAGCAAAGGGCAGATTTGATGGTGTAGCACCAGAACCAAGGGAAGGGGTAAGAAGTGGCCATATACCTGGAACTAAATGTGTTCCATTCCCTGAGATGTTTGATGGGGCACCAATGCTTCTCCCCGCAGATGAGCTATCCAAAAAGTTCGAGGAAACAGGGATTTCCCTTGATCATCCGATTGCCGTCACTTGTGGATCTGGCGTAACTGCGTGCATAGTTGCTCTGGGGCTCTATAGAATCGGGAAGCATGATGTTCCAGTTTACGATGGTTCTTGGACAGAATGGGAAGCTCAGCCAGATAGTGATTACCCAAAAGTTACTTCCACCGCCTCTTAAATCAACCATTCTGAAGTGATGAATGCAGCTGTTGCATCCTGCAGCATTCAGCTCAGTTTGTCAGCGCAATGATATTTCTCTGTGGCAAGCTTTGCACCCAAGCCAATACATATT

>Isogroup_103 CTTAAACCAGCGTAAAAAGATTGAAGCTGAGGAGAGGCGTGTCAAGCAGAGGAAAGCACGTGATGATTTTATCACAATGCTGGAAGAATCTAAGGACCTTACATCCTCGACAAGATGGAGCAAAGCAACAACTATGTTTGAGGATGATGAAAGGTTCAAAGCTGTTGATCGCCCGCGGGAGCGTGAAGATTTATTTGAGAACTATCTTGTGGAGCTACAAAAGAAGGAAAAAGCAAAGGCTGCTGAAGAGCACAAAAGACACGTAGCGGAATATAGAGCTTTTCTTGAATCATGTGATTTCATCAAGGTAAACACCCAATGGAGAAAAGTTCAAGAGCGGCTAGAGGATGATGAACGCTGTTTCCGACTTGAGAAGATTGATCGCTTGGATGTTTTTCAAGAGTATATACGGGATCTGGAGAAAGAAGAGGAAGAGCAGAGGCGAATACAGAAGGAGCAAACAAGGAGACAAGAGCGGAAAAACCGTGATGAGTTCCGCAAGATGTTGGAAGACCATGTTGCTGATGGCACACTTAATGCAAGAACTCGCTGGCGTGACTACTGTGCACAGGTAAAAGATTCACATGCTTACCTGGCTGTAGCTTCAAATTTGTCTGGTTCCAAGCCAAAAGATCTCTTTGATGATGTCATTGAGGAGCTTGATAAGCAGTATCAAGATGACAAGACTTTAGTTAAAGATGAAGTGAAGTCTGGAAAGATTCATATGACTGCCTCGTGGACACTAGAGGAATTCCAAGCTGCTGTTACAGAGGATAACAAATGTAAAGGAGTATCAAAAATAAATATGAAGCTTATCTATGAGGACCAAATTGAAAGGCTCAAGGAAAAGGAACTAAAGGAGGCTAAGAAGCGTCAGCGACTCGGTGATAACTTTCTGGATCTTCT

>>Isogroup_104 TCTATCCATTGTTAAGGGAAGTCAGTATTGGAATAGATCCTTATGTGATCTTCGAAGATGCAGATTGGGCCCTTCTAATCGGCGCAAAACCCAGAGGACCTGGAGTAGAGAGAGCTGCCTTACTAGATATCAATGGTCAAATCTTTGCTGAACAGGGGAAAGCACTAAACGCTGTGGCATCGCGGAATGTGAAAGTCATAGTTGTTGGAAACCCCTGTAACACTAATGCTTTGATTTGCTTGAAAAATGCTCCCAACCTATCAGCAAAAAACTTCCATGCATTGACAAGGTTGGATGAAAACAGAGCCAAGTGCCAGCTAGCACTAAAAGCTGGTGTATTTTATGACAAAGTATCAAATATGACTATTTGGGGGAACCATTCAACAACCCAGGTTCCTGATTTCTTGAATGCCAAAATTGATGGGAGGCCCGTAAAAGAAGTCATAGATGATACAAAGTGGTTAGAAGAAGATTTCACTATAACTGTTCAAAAGCGTGGAGGAGTCCTCATCCAAAAATGGGGCAGATCTTCAGCTGCATCAACCGCTGTTTCTATCGTTGACGCTATGAGGTCCCTTGTGACTCCTACCCCGGAAGGAGACTGGTTCTCTACAGGGGTTTATACTACCGGAAATCCTTACGGCATAGCAGAGGACATCGTATTCAGCATGCCATGCAGATCGAAGGGTGATGGCGACTATGAACTAGTTAAAGATGTCATAATGGACGATTTCCTCTGGAACCGGATCAAAAAGAGTGAGGCTGAACTGATCGCAGAGAAAAGATGTGTTGCCCATCTTACAGGGGAGGGTAATGCATTCTGTGATCTTCCTGGAGATACAATGCTTCCTGGAGAGATGTAGAGCTATCACTCATAGATATCTACCAATTATCTACCTCGTC

>Isogroup_105 CCCCAGAAGTTTACACAATTGCATTGAGAAGCTGTAGCTTGACTGGAGATTTAGGGTTTGCATTGAAGATCTATGAAGATATGAACAAGATCGGAGTTCAACCTGATGAGATGTTTCTCAGTGCTCTAGTTGATGTAGCGGGACATGCTAAGAGGGCTGATGCTGCTTTCGAAATAATCAAAGATGTCAGAGCAAAGGGATTTCATGTTGGAATCATGGCATACAGTTCCTGCATGGGCGCTTGCTGCAATGCCAAGGACTGGAAAAAGGCGCTGCAGCTATTTGAGGAGATTAAGGCGATCAAATTAATTCCAACAGTTCCGATGATGAATGCTTTGATCACTGCCCTTTGTGATGGGGATCAAGTTTTGAAATCTGCTAAGGTCCTTAATGAGATAAAGGAACTTGGTGTCTGTCCAAATGAGATAACCTATTCTGTCTTATGTGTGGCTTGCGAAAGAAATGGTGAAGCACAACTAGGTCTTGATCTATTTGAACAATTAAAAGGAGATGGTATTGACCTCAATCCCACCATTGTTGGTTGTCTTACTGGTTTATGTCTACAAATGTTCGCCAATGATCTTTCACTTGATAACATCATTGTCAGATTCAACCAAGGGAAGCCACAGATTGACAATAAATGGACCTCCTCAGCAATTATGGTCTTCCGTCACGCAATCACCACCGGCCTGTTACCCTCAAGTGATGTTTTATCTCAAGTGCTAGGATGTTTGAGGTTTCCTCATGACAGTTCATTGAAAAAATACTTTCATTGATAACATGGGAATAAGTTGTGATATGCCACATCACCCCAATACAAATTCACTGTTGGAAGGATTCGGTGAATATGATATTCGTGCTTTTTCAATACTGGAGGAAGCTGCTTTGTTAGGAGCAGTTGCAA

>Isogroup_106 ATCAGGAACATCAACACTCACCTGTCTGTCAAAGCGTCCAGGTCTAAGTAAAGCTGAATCCAAGATGTCAGCCCTGTTGGTGGCAGCAACAACAATGATTCCAGTGTTTCCCTCAAACCCATCCATCTCAGTCAACAGCTGGTTGAGAGTCTGCTCCCTTTCATCATTCCCACCACCGATACCTGTTCCTCTTTGCCTTCCAACAGCATCAATTTCATCAACAAATACGATGCAAGGAGCATTCTCCTTAGCCTTCTTGAAGAGATCACGAACTCGGGAGGCACCAACACCAACAAACATCTCCACAAACTCAGATCCCGATATCGAGAAAAACGGCACGCCAGCTTCCCCTGCGATGGCCTTGGCAAGCAAAGTCTTACCAGTTCCAGGAGGACCAACAAGCAGCACACCCTTAGGAATGCGGGCACCAACAGCGGTGAACCTCTCTGGCTTCTTCAAGAACTCAACCACTTCCATGAAGTCTTGCTTAGCCTCGTCGACACCAGCAACATCATCGAATGTAACACCGGTGTTGGGTTCCATCTGGAACTTGGCTTTAGACTGGCCAAAACCAAGGGGAAAGCCAGGACCATTAGGTCCGCCCATGCCACCCGATCCTCTTCTTGACAGCAAGAACAGACCACCGATAAGGATAAGCGGGAAGGCCAGATTCCCGATAAGGTTGAAGAGAAGAGAACCAGAGTCCTCCTGCTGGTTGTGCGCAGCAAAATCAATGTTCTTCTCCCTCAGCTTCTGGAGAAGCTCCTGGCTCAGACCGGGGAGCTGCACACGAACCCTCTGCACACGGTTGCCGAGCTCGGGAGAGATAGCCTCCACAATAGCAATGGTCCCATTCTCAAACAGATCGACCTTCTTCACCCTGTCCTTGTCGAG

>Isogroup_107 AAGAGGCGCAGCCATACAAAATCTGCGCTTGGTCTTGCCTGGAATGAAGTGGTGAGGAACGTTCTAGCTAGCTCAAGTGCAGACGAAACTGTTAGACTTTGGGATTTAAATACTGGTAAATGTGCAGTCACACTGCAACATCATGATGACAAGGTCCAATCAGTTGCCTGGAGGTCACCTGAAGTTCTTCTCAGTGGATCTTTTGACCGATTTGTTATCATGAGTGATATGAGAGATAATGGACAAACTTATCATAAATGGTCTGTCGAAGCAGATGTAGAAAGCTTAGTTTGTGATCCACACAATGAACACTCTTTTCTGGTTAGTCTTGACAATGGGATGGTTCAAGCGTTTGATACGCGGACAGCTTCGTCAAATTCAAATCGTGGACAGCCCACATTTACTCTGCATGCACACAATAAGGCTGCTTCTGCAATATCCTTCTGCCCATCTAAGCCTAATTTCATTGCAACATGTGGAACGGATAAAATGGTGAAACTCTGGGATATATCAAACAACCAGCCTTCATGCGTGGCTTCGCTGAATGCTAAACTCGGAGCTGTATTTACGGTAACATTTTGCAACGACAGCCCCTTCTTGTTAGCGTGTGGAGGATCCAAAGGCAAATTGAAGATCTGGAACACGCTGTCGGAACCAGCGGTGGCTAGTAAGTTTGGCAAGTAGACACAGCAAAATGGATCAGCTTCCTGGAGACTCGAGAGACTTGGTATCTATCGAAGTTACATGCTGTAGTTGTTTCTGCCTATGTGTGAGGATAGATAGATTTTCGCCCTACGGCAACAGTTTCGTGCATCAGGTGAATGTCCCAGTTTTGTTATGGAGCGAAATGGAATCATGGGCTGTGCCATGTATTACTATCTGTTTTGGCGTTT

>Isogroup_108 CTTGATGATATTGCCATTCTAACCGGAGGAACTGTAATCAGAGATGAGGTGGGCCTGTCACTTGACAAGGCGGACAAATCGGTTCTAGGAACGGCTGCAAAGGTTGTCCTTAACAAAGAGTCAACAACAATAGTTGGTGATGGCAGCACCCAGGAAGAAGTGACTAAAAGAGTTGCACAGATCAAGAATCTCATTGAGGCAGCAGAACAAGATTATGAAAAGGAGAAACTCAATGAGAGGATAGCAAAGCTCGCTGGTGGTGTTGCTGTCATTCAGGTTGGAGCGCAAACAGAAACTGAACTTAAGGAGAAGAAGCTGAGAGTTGAGGATGCTCTAAATGCAACTAAGGCTGCCGTTGAGGAAGGTATTGTTGTTGGTGGCGGATGCACTCTTTTGAGACTGGCAGCTAAGGTTGATGCCATCAAGGACACCTTGGACAACGAGGAGCAGAAGGTTGGAGCCGAAATCGTTAGGAGGGCGCTGTGCTACCCACTTAAACTGATTGCCAAGAATGCAGGTGTCAATGGCAGTGTTGTCACTGAGAAGGTTCTTTCTAACGACAACGTCAAGTTCGGGTATAATGCTGCTACTGGGCAGTACCTTCGCAAGTTTACCACGCGTAGAAGGGAATAGAAGTCAATGCGAGCGCTTCCCATCCACCTGAACTTTTCTGACTCAATATCAATATCAGCTACCAAACCCCATGTAAGCATCATGACACTAAAAAACTTTGTCTTTCCCTGCACAACAGAAGTGACATCGAGGGCACGCTTGTGACCTGTGTTGCGGTAAGGACGCATCAGCCTCTGATGATTGCAAAAACAGCATTTGACATAGAGAAGGGTTCACCAGAGGAATGCAACAGAGCTTGCACCATTCCATTCCCAG

>Isogroup_109 CCTGACTGGCGTGGATCGATTTCTTCCTTCCGTTGAAATTGAGGAGCGCAGCATAACACAAGAAAATTGAGCCTATCCTTCCTATGGGAGATTGTTTTGATTTCAAGTATGGCTTTCGCGAAATGCAAAAACTGACTGGGAAAAAGCCTAACTTAGGTTGACGGAATGAAATTGAGGAGCACAGCAGTAACACGAGCAAATTCAGCCTCTCTTCCTATGGAGGACCGTGTCGATTTTAAGTATGACGGTGTCAGTGGCGGCCAAGAGCGTAAGGGGGGGCATGGCATCCCAAAGGTTTCCATGGTCCCCCTCGTCTTCCTCATATTCTATGAAGTCTCAGGGGGTCCCTTTGGGATTGAAGATAGTGTCAAGGCTGCTGGACCACTCCTCGCAATTGTGGGTTTTCTTCTGTTTGCGCTCATATGGAGCGTTCCAGAATCCCTAATCACTGCTGAGATGGGGACTATGTTCCCTGAGAATGGTGGCTATGTCGTCTGGGTCTCTTCAGCTCTTGGGCCCTTTTGGGGTTTTCAGCAAGGCTGGGCGAAGTGGCTCAGTGGTGTCATAGATAATGCTCTCTATCCAGTCCTTTTTCTTGACTATGTCAAGTCCAGTGTTCCAGCTCTCAGTGGCGGGCTTCCAAGGACTTTTGCGGTGCTTGTCCTCACGGTTGCCCTTACTTACATGAACTACAGAGGATTAACAATAGTTGGCTGGGTGGCTGTCTTTCTTGGGGTGTTCTCTCTGCTCCCTTTCTTTGTAATGGGATTGATAGCTATTCCACAGATTGAACCTTCAAGATGGTTTGAAATGGACCTGGACAATGTGAATTGGGGATTATATCTGAACACTCTGTTTTGGAACCTCAACTATTGGGATTCTATCA

>Isogroup_110 AGAGCTGCTACTTGCTCCACACTGTCACTCAGAATGTAATCAAACTTTAGGAAACAGGAAGATCATATGAACATGAATCCTAAGTTCCTAACAAGTAACAACTGTAGATCCTTGGATATATTTATATATGTGCTATCCGGATCAACGAACTTCTGCCATCTCTACAACACAATTCCTACAGCTGTAAACATCAAAGGCGTTTTGTTCTGGGGCTATTTGGAGCAGCGTGAGGGAGGATGGTTTAACTGATCATCAAGATTGTGCCAAATCGTCCTCAAATGCTTTCTGCTTCTGAAACTAGAAAGTTCTCAGAGTATGGGTCATGGTGCTAAATCTTTGCATACGTGTCGGTGCGTGCCCTCAGAGCCTCGGTGCGTCTACTCTTTCCCATTTCTTCTACCAACCAGTCGATACCAAGTTTGATCCCCGTCCCATCATAGGCAGATCCAGCCACGAACATATATGGCCTCTCATCAAACTCTTTAAGGTGAAGATGTCTGTCCAATTCTTCTTCTGTGACTGCTGCTGGTAAATCCTGTTTGTTTGCGAATATAAGTAGTGGTGCATCTTGCAGATCCTCATGACGAAGCACTTTCTCAAGAGCAGATTTGGCATCTTCAAATGTTGATGCGGAGGCAGAGTCAATGACATATACTATAGCATGTGCCTCGTCATAATATTTCTCCCATATTGTTCTTAGGCCAGGCTGACCTCCCAAATCCCAGAAAACAAGTTTTACATCTGCATCTTCAATACGTCCAATATTAAGCCCCACAGTTGGAACAACACGATCATGTGGAAGCCCTTCTCCTTTCAAATAGATTGACTTCAACTTTTCTAGCAAGGTCGTCTTCCCAGCCTTATGGACTCCAAGTATGA

>Isogroup_111 CTTCAAAGATTGGTGGATTGGTGCGTTGAGTATTGTGACCTTCAGAACAGCATAGGGGTTGCAGTTAATCCAAACCATCAAATATTTTATGCGACCTGTCAGGCTGTGATGTATGTCCTTTGCTTTCGCTTAAGATCTATTATGGATTACCCAAATCTTAAGTCACAGCTTTTTCACATGCCTTTTGGATTTCTTTTGACCCATCCACTGGAACCTCTAAAGGTGTGCTTGCCTTCAATAGTAAACGAGTTCCTGAGACAGGCTAAGGCTGCCAGTTTGTTCACTGCAAATATGGATTCAGCATTGGATGATGCAATTGAATCTGATTTGTCCAGGACTTTTGGAGGAATTGGTAGACTTGACATGTTCTTCCCATTCGATCCTTATCTGCTAAAAGAATCTGACAGATATATGCGTCCAAATTTTGAGTTCTGGTCCATGGTCAGGACAACATACAGCGATGATAATGAAGATGACGACGACGAAGAGCTTGAGGACCTCGATGCCCCTGGAATGAATGTGGGCAGCTTGGACGATCACATTGAGATAGATATCAACAATGACGATGACTTGAATTACTCGATGAATAAGATGTCTATAACTCCATATCGCTCTTTCTTTCATCCAGTGGCCACAGACAGCGATGATGTCCTTAGCATGCCTGCAAGGATCAGACCTTCAGTGAGCCCTCCGGTATGATGGGGCCAGTGCAGGATCATCGTAACGAACTTAATCTTTACTTTATTGGAGAAACAGCTGGATACATGGAGTCGCATGCGGAGCCAATTTGTACAAGGGTATGACCATCAAAACTGATATGGGGAGCTTCCATCGTAGTTTGTGTAGTAGGGTGAACCAACCCATTCATCTGCAAT

>Isogroup_**112** CCTTGAAGGTTCCTTCTGGCCAGCGCCCTGTATATTTTGCGTCAGATGATGACAGAACGGAATCTCAGAATTATGCCAGGCCTTTGCTAAGCTCAAGAGGAGTGTCCAGTAATCTTGAGCATCGACTTATAAATCATACAGTTAACTTCCAGAAAATGTGGAGCAGTGAACAAGAAATTCGGAGCCGGTGTAAACTACTTGGGAAGCAGGTTGCAAACGATGGACGGATATTTAGCATTTGGAGGCCCCTATGCTCTAGTGGGTATGTCTCAATTGGAGATGTTGCACATGCTGGCATCCATCCGCCACATTTTGCCACTATCTACAAAAATGTCAACGGGAATTTTGTGCTTCCTGTCGGCTATGACCTGGTTTGGAGAAATTGCGAAGAGGACTACAGGAGCCCTGTATCATTATGGCAACCAAGACCACCTGAAGGATACATGGCTCTGGGATGTGTGGCTGTGTCGGCTTTCGAGGAGCCCCCTCTTGATTGCGCCTTTTGTGTGAATGAGAGACTTGTGGAAGATGCAGTGTTCGAGGAGCAGATTGTATGGGCTTCCTCAGATGCTTACCCATGGGGATGCTACATTTACCAAGTTCAGTGCAGTTCGCTGCAATTCATGGCGCTCCGTCTGCCAAAAGAGCAGTCTCAACAGAAGCCTAAGAAGATAAAGGAGTCGTATCTACAGCAAGCATCAGGAACTCTATGATCCTGTGAGGATAGAAGCAGACAGCGCTGGGCATGGAGACATGAAGTGCGATGTATATAATATATGAAGTTGCACGAGCCCAGGAAAACTTTGCTAGGCAAAAGGGAAGATCCGGCAATCTCGTGCAAGATAGTTGGTCTTGGATATGTGACATTTTT

>Isogroup_113 CTCTTCAAGATACCGACACACAAGTAAAGGTGGAAGTGTTCTGACAGATAGTGAGTCCACACAACTTCCCACAGGAGCATGATCTGATCGAAACTGAACTCCCTTTTGAATTGTATAAGAACCCAGCGGAAACAGAAGAAGTAGTTTAGACAGTCGTTCTGCCTAAAATAGTTATGCAGCGAAGGATCGAGAAGCTCCACTAGCTTGGATAATCCAAGTAGTTGAGCATGCATGCCATTCTGATCACGATTAAAATTGCCTCCTAGTCGTTCCATCAATGAAGCAAAGCACCAAAATGATTCGGACTCATCTTCCATGACATACAGTATAGGTGCCAAAAAATCGCTCATTCCTTGACAATAACCAAGGTCAAAATTATAGAAGGAATATGTCAACAAGATGTCACGTAGAACTACAACATTCCGATTGTCATCTCCTTCATAGTAGGGAACAGATCGATCTGTTCTCACCACATCTTTGTCAATAAGGCCTTTTCTTTCCCTAAACTTGGTGAACCTTTTAGCTTGCGTGGCTGAGATACTCTTCCACTGGGATTTTATGGCTTCATATTCTTCTCGTTTCATTGCAGCAAGGTATTCCCTCTCAGCTTGTGTTGAATCATATTCATGATACCCCAACAAGAATTTCCAGACCTCTTTTCTCAGAGCATGATCAACTCCCCCCATAGAAAACCTTTTTCCTCAGTGCCTTCGAATCCAAAATTCTACCTTCAGGATCTAAGAAAGCTCTCCACTCTTCAACACTTAATGGGCTGCCTCTCTTTTTTCCCCACACTAGCAGTAACTCATCAGACTCTACTGAGTTTGAGGGCGAAGAAGCATTATCTGGCGTTAATTGCTGATCTTT

>Isogroup_114 CATGAGGCGAAATCTTCTTTTGCACACCATGGTGTCAAGTTCTCCAATCTGGAGATAGATCTGCCTGCAATGATGTCCCAGAAAGACAAGGCTGTTGCTGGGCTTACAAAAGGCATTGAAGGCCTTTTCAAGAAGAACAAGGTTGAATATGTTAAAGGGTTTGGGAAGATTGTCTCCCCGTCAGAGGTGTCTGTGGATTTGGTCGATGGTGGTAGCACTATCGTCAAAGGGAAGAACATAATTGTTGCCACTGGCTCAGATGTGAAATCACTCCCTGGAGTTACAATAGATGAGAAAAAGATTGTGTCATCTACTGGGGCTCTTGCACTTACAGAAATACCAAAGAAGTTGGTGGTTATTGGAGCTGGATACATTGGTCTGGAGATGGGTTCTGTTTGGAACAGGCTAGGGTCTGAGGTCACTGTCGTTGAATTTGCCCCTGATATTGTCCCATCAATGGATGGAGAGGTAAGGAAGCAGTTTCAGCGTATGCTGGAGAAGCAAAAATTCAAGTTCATGCTCAAGACGAAGGTAGTCGGTGTTGATAGCTCTGGAAGTGGTGTCAAGTTAACTGTGGAGCCAGCAGCTGGCGGAGAGCAGACTGTCATTGAAGCTGATATTGTCCTTGTATCTGCTGGCAGAGTCCCATACACCGCTGGCATTGGGTTGGACACTGTTGGTGTTGAGACAGACAAGGGTGGAAGGATCCTTGTTGACAAGCGCTTCATGACAAATGTTAAGGGCGTATATGCGATTGGGGATGCGATACCTGGACCGATGCTTGCACATAAAGCTGAAGAGGATGGTGTTGCATGTGTTGAATTCATTGCTGGGAAGGAGGGCCATGTTGATTATGACACTG

>Isogroup_115 AGCGGCCGCCCGGCGAGTACGTGGAGCACGGATTTGTTTCACACATGTAGATCTCTGGTGCAGAATCAGTTGGCTCTCTAATATCATCCAGCTGTTGTATGGGGAGTAATTCTGAGTTCGCATTGTTGTTATCTACGGCGTTACAGTCGGACACAGCTCCACTGTTTTCACATAGAATATTTCCAACGGAGAAGGACCTCTTTATGAGTGGTCTCACAGTTTCTTCACCTACAGTTCCTTGCCTGCCAATACTGTAATGCTGATCTGAGTCCAACTTCCAAAGTGCAGGTTTGCCTTGTTGAGGTTGGAAATGACCCAGGAACACATTTATAGCATCCTGCTTCTCAGGATCTGTGTAGGCATTATTGTAGTAACGTTGAAGGGTCCGAAGGAACTCCTGGGATTGGGTTGCTGCCTTCCACTGGCCTCTTTGCTCGCAGAAAATCTTGTTGTGAGCAGCAGAACCACCGTATTGTATAGCTAATGTATCACCCATCCTTTCATAGAAATCCATCAGATCATCAGCCAAAGGAGCATGCAATTCAATTTTAGGTGCTTCAGTGAGCCCCAACACATGAAGTTGGCGCCCTAAAGCAGCTAATCCGTATGCAAATTGGGCGACATTTGTGCGGTCTAAGCAGTCTATGCAGTTTGTCCTGAGGACACCCTTCTGTAGTAAAAGAGGCACCAACGTAGATGTGCATAAATCATCATTGCTTTCGACATCACACGACTTGATAACACATCCTTGTCCACTCGAGGCATCCAACGGTTTTGTGATGTAGTTATCTCACAATGGAGGATGTCCGTTAGTTCCAACACATCTGATGCTACCTTGTTTAACAGTGAAAGCACATTGG

>Isogroup_116 CATGTTAAAATTCTCAATGAACTGCTTGACAACATGAGTCACTCGTCTGCGCAGGTAAGAGAAGCGATTGGTGTGGCTATGTCTATCACATGCTCTAATGTGAGACTATCTGGGTCATTTGGGTCTGCATGTTCACCAGGAGAACTTTGTGGGGATGTAAGCATGACTGAACAAACAGGAAATGAGGATTGGTCCAAACGTTTGACAGATGGAACTACTGAATTATCTGTGAGTATACAGAACAATATTCAGTCTAAGCAACTGGAGTTGGCAGCAGATTCAGCCACTGAAAATGGCTTGGACAATAAAGATGAGGCTGATGCTAAAAGAATGGAAACGATTTTTCATTTCATGATTGCATCTCTGAAGTCTGGAAGATCGTCTGTTCTACTAGATGTTATTATTGGGCTCTTCTATCCTGTTCTGTCATTACAGGAAACATCAAATAAAGACTTGTCATTGCTCGCAAAGTCAGCATTTGAATTGCTGAAATGGCAGATTTGGCGGCGTCCTTTTCTTGAAACAGCTATTACGGCTATCCTCTCTTCGGTCAATGATCCTAACTGGCGAACTAGATCTGCGTTATTATCATATCTCAGGACCTTTACATACAGGCACACATTCCTTCTTTCTGGCTCGGAGAAATCACAAATTTGGCAAACAATTGAGAAGTTACTTGCGGATAACCAAGTCGAGGTCAGGGAGCATGCTGCTGGTGTTCTTGCAAGCTTAATGAAGGGTATAGATAAAGATCTATCGAAGGATTTCCGTGATAGGTCATATGCACAAGCCCAACTTATCCTTGATACTAGGAGAAGAAGTCCAAAGTCAGGTCACCCCGTTGCTACCATCCATGGTGCA

>Isogroup_117 CTCAAAGTGCAAAGGCCTGGTCTGAAAGAGCTGTTTGATATTGATCTGAAGAACTTAAGGGTAATAGCAGAATACCTTCAGAAAGTGGATCCCAAGTCAGATGGCGCCAAGAGAGACTGGGTTGCTATATATGATGAATGTGCCAATGTATTATATCAGGAAATAGACTATACTAAGGAAGCATTTAATGCTGAAAAGTTTGCTGAAAAACTTCAAAAACATGGACTATGTAAAGGTCCCATCAATTTACTGGGAGTATACTACACCTCAGGTTCTAACAATGGAATACGTCCCAGGAATCAAGATAAATAGGATACAGCAGATAGATAAGTTAGGACTTGATAGGAAAAGGTTAGGTCGGTATGCTGTCGAGTCCTACCTTGAGCAGATCTTATCGCATGGATTTTTCCATGCTGACCCGCATCCTGGGAACATTGCTGTTGATGATGCCAATGGTGGGAGGCTTATCTTCTATGACTTTGGGATGATGGGAAGTATTAGTCCAAATATCCGTGAAGGGTTGCTTGAAGTATTTTATGGAGTTTATGAAAAGGATCCTGATAAAGTGCTTCAAGCAATGGTTCAAATGGGTGTCCTTGTTCCTACTGGAGATATGACAGCCGTGAGAAGAACAGCTCAATTTTTCCTTGATAGCTTTGAAGAGCGTCTTGCTGCACAAAGGAAAGAGAGAGAGATGGCAACCGTGGAACCAGGATTTAAAAAACAATTATCTAAGGAAGAAAAGTTTGAAAAGAAGAAGCAGAGACTTGCAGCTATAGGAGAGGATCTTTTGGCAATTGCTGCTGATCAACCATTTCGGTTTCCTGCCACCTTTACATTTGTTGTCAGAGCATTCTCA

>Isogroup_118 ACTTATTTCACATCTGGAATCTGTATTCTACAGGCGATCATTGATCGGCATCGACTAACTCTAGCACATGATTTACAGATATTGCTAACATGTGCAATCGATGGCGAGAGAGCGAATGCTATACAGCTCTCCGCGAATGATTAGACTATACATGGACTAAAATGGGGGTTAACTGACACTTCCGAGAAACCACTCGATACTGTGTGACGACTGTGTTCTCACTCCTTGGTGGATGCTGCCTCTGGAAGTTTGAATGTAGGAGGCCTGTCTGCACCTTTCCTCGAGTATTCTCGCCACCTCCTCGCTGCCGCAGCAGCTCGCTTCACTCTCTCCTTTGTGTCCTCATCGTTCTTCTGATATCTTGTTGGTGATCCCCATATATCACTCCATGAGCTACCCTTGTTAGAATCATCTTGGCTCATGCGCATTCTGACCTTATCCAAGACTTTTGCTTGTCTTTTCTCCCAGCGCCTACTTGCCATCCGGAACACATCAACTGAACTTCCCATCCCAGAAAGCATCAATGCAATATTCACTCGCTCCACTCTGCGCATTTCATCCTCAAGGAGTGAAAGTTGTGCTGCTGAAGTCCAATGGATGCAGTCAACCGGACAACTGTCAATTGCATCTTGAATTAGCTCTTGGTTGCCTGATTGGTTGTAAACTCTTGCACGCCCAAAATCCTCCTCGATTTCGAAAACCTTAGAGCACACATTAGCACAGTTTTTACATCCTATGCAGCTAAACTCATCAACAAACACACAGTCCCTGGGTGCATCAGCCAAGAAGGGGTTGGTTGCTGTTGCAGTATAGCCATTGATCTCGTCATACACCGCTCGCTGGATGGGATCGGTAAG

>Isogroup_119 CATGGAGAAATCTATCAAGGCTAGCATTGATGCCTTGTGGGCTCGTTTCCTGGAGGAGAACACCAAACGTGAAAAGGCAGAAAGGGAACGAATGCAGCAAATGACCACTCTCATCACAAGCTCAATAAGCAAGGACCTTCCCGCTATGTTGGAGAAGTCACTAAAGAAAGAAGTTTCTTCACTTGGACCTGTCATTGCGCGAACAATGACACCTATTATTGAGAAGTCACTAGCCTCTATAATTGCTGATTCAGTTCAGAAAGCGGTGGGTGACAAGGTGGTCAATCAGCTGGACAAGTCTGTTACTGCAAAACTTGAAGCTACAGTTGCTAGGCAAATCCAATTGCAATTTCATACATCTGTAAAGCAGATTCTTCAGGATGTGTTACGCACCAGCTTTGAATCATTGCTTGTTCCAGCATTTGAACAATCCTGTAATACAATGTTTGAGCAAGTAGATGGTGCATTCCAGAAAGGAATGTCTGAGCATGCTGTTGCTATTCAGCAGCAGGTTGAGGCAGCATATACGCCATTGGTGCTCACTTTAAAGGAAACTATCACTTCTGCGTCGTCAATCACCCAGAGTCTTACATCAGAGTTACTTGATGGCCATCGTAAACTATTGGCACTGGTTACATCTGGGAACGCCAATGCGCAAAATACAAATGTTTTGCAGCCCAACAATGGCCCTATCACTGGTCCCCCCAGAGGTTGAGGCTCCACTAGATCCGATGAAAGAGCTGGGAAGGCTGATATCTGAACGCAAATTTGATGAGGCTTTTACAGTGGCGCTTCAAAGAAGTGACGTGTCAATTGTGTCTTGGTTATGCTCTCAGGTTGATTTGCGTGCATTAT

>Isogroup_120 TGTCCGGAAACCAAAGCCACCCGTGTTAGCAGCTGCTCTATCCGCGTGGACATTTTTGCTAACAACTATTGGTTCATGGCGGATAAACACCGATAGCTGGAAGGAGCCAATTGCATTCCTCTCTACTCTTCTAGGAGCAGAGGATCGTGCTGTTCGAATGGCTGCTGGTGAAGCATTGGCTTTGTGTTTTGAGTTAAATCTACTTGATGTCTCTGCTTGTGAAGATGATGATGACACTGGAGTAGCTGGTAGCTCCAAGAGTAAACTTTTCCTGGATATGCAAGCATTGAAAGCCAAGATATCAGGCCTTGCCTCTAATCTTTCTGCGGAGGCAGGGGGGAAAGGTGCAGACAAGAAAAATCTTTCTGACCAAAGGGATCTGTTTCAACGGATTTTGGATTTTGTTAAGTATGGCGAGTGCCCTGAAGAATCACTGAAGATTGCTGGAAAACGTGATGTTTTAAGGGTTTCGTCATGGTCTGAATTGATTCAGTTGAACTTCTTTAAGCATTTTCTTGGGAGAGGCTTCCTGAAGCATGTGAAGGACAATGGACTTCTTCAAGATATCTTCAGTATCAAGACTGACAAGGCTGAAACTCTGTCATCCAGTGACAAGAAAATCTTCAGATCTGGAGAAGAGAAAGGAAGAGCTCTGAAGCTGAACAAGGATCGTCGTCAAGCACAGGAGAGGAAGAACGCTGCTCTGTTGAACGAGTAATAAGGACGAGGAAACACAAGCTTATTGGCCTGGCATTTTACTCCTAAAGCTGGAATTTGTTTATTTCTGTAAACAACTACAAGCAGGAAGTCTAGAGTCTGCTCTATATTCTTGTGATTCCTTGCGTACCTCGG

>Isogroup_121 CACAAGATGCGGCTTTCAAATCAGAGCTCTGTGACTTGTATGCCAGCTTTGTTTACTCAGTGATTCCTCCAGGACATGGAGATCTCAAAGGTAACGAGGTTGAAGCTATTATAAAGTTTAAAAGAGCTCTTGGACTTGATGATGTAGATGCTGCAAATATGCACATGGCGGTTGGTAGACGCTTATACAGAGAGAGGCTCGACGCGTTCCAAAAATTAATTTTTGTGTCAAATCTTGTCTTCGGAGATGCATCTGAGTTCATACTTCCATGGAAACATCTTTTCGGGATCACGGATTATCAGATCGATATTGCTATGCGGGAGAATGCCAAAAGTTTATACGCGTTAGAGCTCAAGTCTATTGGAAGAGGTCTTGACATAGGCACGCTCATTGAAGTAAGGAGGGTATAGATTGCATATAAACTTTTTGACGAGATTGCTGCTGACATGTTCAGGGAGCATGCGAAGAGCTTGATCCAAGAAAACATTTCATCTGCTCTATCCATATTGAAATCCAATTCGGGTTCGACGAACATTCCAACAGAGGTTATTAGTGAAGTGAATAGCATTCTTGCATTCAATAGTTTGCTAACAGTTCTAAGCAAGTTCCCTCAGGGAGATCGATTTGCACGTGGGCTTGGACCAGTTTCGTTAGCTGGAGACTTTGATCATGATAGGATGGTTGGTGATCTAAAGATACTCTACGCGGCCTATGCAACGGAAATACTTTCAGATGGAAACCTTGATGATGAGAAGTTTGTTCGCTTGAGTGAATTGAGGAATATATTTGGACTGGGGAAGCGTGAAGCAGAAGCAATCATAGAAGGAGTCACGTCAAATGTTAAATCTCAA

>Isogroup_122 CATAAAAAGCATGAACTCCGCTACCAAACGTAATTCCTTCAACAGAAAATGCATCAACTTGTTGTCTCCCTTACTTTGAAAGACCAGCTGGGAAGGCATTAGCAGTTCTCCGAATTAAGAGACGAGGTGGATAAGCAGCCTCGCACTTCTCTCGCAGCTGAGCCTCTTTCCATCGAATTAGCAGGACTATCTTCTGACGTTGAATTCAGGGCTCCGGGATTCATCCGCTGGACCTCCTCCCGTGTGTAGATGAAGATCTTGTAAACTATATTGCAGAATTCATTCCAGGGGTCATCCCCGACCAGCATCATGTCACCTTCATGGTCAGTGTATACAACCATCCACTCCTTGTTAGAACTCTTCAGCTCGCCTTTGAAGTCAAACATGCCATCCAGCTCAGCAACCAACTCCTCATAGCCATTAAACTTAGTCAGATCTACAGACCTGCCAAGGGCAATACCTTGCTTGTGGACCTTCTTGCAGCTTCTAGCCGATCCGCCCTGTGACTTGCACGACATGTTTCTTGAAGCTTTCTGACAAGAGAGATGTTTCTCAGGACCAGAATCTGCATGCAGTGATTCAAGTGGCCCCAGAATCTTGGGATCAGATAATTTGTCTAGTTCAGTTGCATCCATTGGACACCATTCAGCAGCCCCGGAAGTTTGTGGCGTCCCATCATATGCAACACTCGGTGGAGATTTCAGAGGTTCAGATTTTGCAGGGCTGTGAAGGTGAATTCCAAATAGCTTGCATGAAGTGCGTTTCATTTTCTGCATATCACCATGAGCAACGACCAAAGGCTGAGGCTTGATCACACACGGCTGTGTGTCATCCATGTGAGAACTTGGTACC

>Isogroup_**123** ACTCACTTGCTGTTCACAGTCCGCAATTGCTGTCTTTCAGCATCCATTGCCCGTAAAGCCTCGCTAGCCTGGTGACGAGCACGTTCCAGTTCCTGTTGTGCAGCTAGTTTCAGAGAAGCAGCTTGTTCAGCCTTCTGATTAATTTCATTAAACTGAGCTTCCAGATCATCTTTTTCCTTCTGTATATCTTGTATACGGTGTTTTGCGCGCTTGTGAAGCCTCTGGAACTTGGTATCCAACTCAACATATTTGTCATCTCGTTCTTTTAGTTCTTTTTGCATCTTCAGTTGAGCTTCGCTGAGCTTCGCCGTGAGCTCCTGGACCTTGCCATCGGCCTCGGCGTAGGACACGTTGACGTGCTCAAGGGCCGCCTCCACGACCTTCTGAGTCAGCTTCTGCTCCTGAACCTCCTTGCTCAGCCTCTCAACCTGCTCCTTCAAGCTCCTAACGTGCTCGGAGTCCTCCTCATCCTCCGTCACATTGCCTCCTTCCATATCTGCGCTTCTTACTGGCTGCTGAGCACCGGCGATCTGGGACTTGAGATGCTCGTTCTGGATACTGAGCTCCGTGACCTTCCGCTCGAGCTCTTTGCGCATACGCTCCCCCTCCTTCCCACTCGGCCCTTCACCTTCTGCCGCTTCCGTGTCCGTGCCCGTGCCTGCCATCTTTTGGTCTACCGGCGCCGAACGCTCTTCGACCACCGGCGCCGCACTCTCTTCGTCCGCTGTTGCCCCCTTCTCTTCGTCCACCGGCTCCCCGTTCTCTTGGTCTACCTGCACCGCACTCTCTTCGTCCACCGGCTCCGCACTAACTTCGTTCGCTGGCGCTGAACTCCCCACCTGCCCGGGCGG

>Isogroup_124 CTGAACAGAAAGGATGCCGGCCCTCAGCGATCTCAACAAGGGTCTCGGGCCCAGTGCCAAGATGCTGATGGTCATAGTTGAAGTCTGCTGGAGCACCAATGTAGCCCACCTTTGCTTGTGTTGCTCTAACAGTCTTCTGAATCCTTGCATTGACTATAGCAGCTTCAACTCTAGGCTGTGTTCCAACCAAAAGGAAGACATCAGCTTTCTCAAGCCCAGCAATGCTAGTATTCATTAGGTAGTTTGATCGAAGATCTGCTGGTGGATTCGGACCATTTCCCTCACAGAGCACCTTGTCTGAACCCATTTTGTTGACAAAATCTTTCAATGCCATCATGGATTCTGCATCGGAAAGTTTGCCAGCGACTCCAGTAATCTCTTCGGGTTTAACTTGGTTCAAAACCTCAGCAACAACCGCAATAGCATCACGCCATGTCACTGCCTTAAATCTGCCATCAGAACCACGAATCATAGGATCATTTAGTCTTTGCCTCTTCAGACCATCATAACAAAAACGCGTTTTGTCAGATATCCACTCCTCGTTGATATCCTCATTGATGCGTGGAACAATGCGCATAACTTCAGGACCTCTGCTGTCAACTCTAATGTTTGAACCTACTGCATCGGTGACATCAATAGTCTCAGTGCCCTTCAGCTCCCAGTTCCTAGCTTTAAATGCAAATGGCTTGGATGTAAGAGCTCCAACAGGGCAGATATCAATAACATTTCCAGATAGTTCACTTGTCATAAGCTTCTCCACATATGTTCCAATTTCTTCACCACTACCACGACCTAACATACCCAGGTCTTGAACACCAGCAACCTCAGATGCAAACCTAACGCACCTT

>Isogroup_125 CAAATTGTGATGACAACCAGCCTTTCATCAGATGTGCCGGTTGGCTACTTTTCATGGGCTGAATACGATATCATGGCACCCGTGCCTCCGAAGACTGAAGAAGCCCTAGCTGCAGCCTTTATTTCCAACTGCGGTGCACGCAACTTTCGTTTGCAAGCCCTTGAGATGCTTGAAAGCTTAGATGTAAAAATCGATTCTTATGGTAGCTGCCATCGTAATCGTGATGGCAAAGTGGACAAAGTGGAGACTTTGAAGCGCTACAAATTCAGCTTGGCTTTTGAGAATTCTAACGAGGAAGATTATGTTACAGAAAAGTTTTTCCAGTCACTGGTAACAGGGGCCATTCCAGTTGTCGTTGGTGCACCAAATATTCAAGAGTTTTCTCCAGGAGAAGGTGCAATATTACACATTAAGGAGCTTGATGACGTTACTTCAGTTGCTAGGACAATGAAACATATTGCATCAAATCCTGACGCTTTTAATCAATCTTTGAGGTGGAAGTATGATGGTCCATCTGATTCTTTCAAGGCACTTATTGACATGGCAGCGGTTCATTCATCATGTCGCCTCTGCATACATATCGCTACGAAGATTCATGAAAAAGAAGAAAGGACTCCAAAATTTATGAATCGCACATGTAACTGTTCCAGCAAAAAAGGAATAGTATACCACTTATTTGTTAGAGAGAGAGGGAGGTTTAAGACAGAAAGCATTTATCTAAGATCAAACAAATTAACTTTACAAGCTTTGGAGACTGCTATACATGCCACATTTAGATCCATCAACCATGTTCCTGTATGGAAGGATGAAAGGCCATCAAGCATACGAGGAGGGGACGAGTTGAAGGT

>Isogroup_**126** TCCAGTAAGGATGATTACTGGGATCTAACTGTGGCAGTTGGTTCTGCTGAGTATTCCGAGTCCATGCCGGCAGCAACTGAACTTTTCAGGACCCCACTGGTATCATTTCTTTATCAGAGAGGATGGCGCCAAAATTTCATATGGGGTGGTTTCCCAGGCCTGGAGAGAGAGTTTGACATGGCAAAAACTTATTTGAAGCCAAAAACTGGAGGTGTTATAGTTGATGCAAGTTGTGGAAGTGGCTTATTTTCAAGATTGTTTGTCAAAAGTGGACTATATTCTCTTGTCGTGGCACTGGATTTCTCAGAGAATATGTTGAAGCAGTGCAATGAATTTATCAAGCAGGAAAACATTTCCGATGAGAGATTAGTCTTGGTCAGAGCTGATATATCCAGACTCCCTCTTGTGAATGGTTCAATTGATGCTGTGCATGCTGGTGCTGCAATTCATTGTTGGCCATCCCCAGCTTGTGCTGTAGCGGAAATTAGCCGAGTCCTTCGCCCTGGGGGTATTTTCGTGGCTTCTACTTTCATAGCAGACGTTCTGCCACCAGCTGTTCCGGTATTGAGGATTGGACGTCCGTATATTGGTCAAATCACTGGCAGCAATATCTTCTTATCAGAAGGGGAGCTTGAAGACCTTTGCAGAGCATGTGGGTTGGTTGATTTCACATTTGTCAGAAATGGATTTTATATAATGTTCTCTGCAACTAAAGCAAGCTAGTCAAATAAGTGCAGAGTCTTCTCTAAGGGTTCAGCCTGTAGTGGAAGTCGTGAATACACTGTTGGATTTTGGAAGTCGTGAATACACTGTTGGATTATTCATCTGAAAAATTATACTAC

>Isogroup_127 CTTAGCACAAAGAACACAATCCTTAAGAAATATGATGGCAGGTTCAAGGACATATTCCAGGCGATCTATGAAGCTGACTGGAAATCCAAATTTGAGGCTGCCGGAATATGGTATGAACACCGTCTCATTGATGACATGGTTGCCTATGCACTTAAGAGCGAAGGGGGCTATGTTTGGGCTTGCAAGAACTACGATGGAGATGTGCAGAGTGATTTCTTAGCTCAAGGTTTTTGGCTCTTTGGGCTTGATGACATCAGTATTGATGTGCCCTGATGGTAAAACTATCGAAGCTGAAGCTGCCCATGGCACAGTTACCCGTCATTTCCGTGTTCACCAGAAAGGTGGTGAAACCAGCACAAACAGCATTGCTTCAATCTTTGCTTGGACAAGAGGACTTGCACACAGGGCAAAGCTAGATGGCAATGCTAGGCTTCTTGATTTCACACATAAACTTGAGGAAGCATGTGTCGGAGCTGTGGAGTCAGGGAAGATGACGAAGGATCTAGCACTTCTTGTTCACGGATCTTCAAAGGTTACAAGAGGAGACTACTTGAACACTGAAGAGTTCATTGACGCAGTTGCTGCGGAGCTCCAAGCAAGATTGGCAGCCAACTGAACATTCAAGAAAACCTCGTCCACTTCAGTGCTTCAATAGTCATTACCCAACGATTCACGTCAGTTTGGTTATGTCGCCATTATCTGTCATGCTTTCTTCCTGAACAGAATGAGCTTTTACTTGCTCCATCGCAAATTATTGTCAAAATAGCAAGCAGTGCTGTAGATGCTGATGATATGTAAGTGGAGCTCTAAATAAAAGAACGTATAGAACGATTGTTCAGCTC

>Isogroup_128 CAGGTGGCTCATATCCCTGGTGCCTTATTCTTTGATGTTGATGGGATATCTGACAGGGCATCCAGTTTGCCACACATGCTGCCATCTGAAAAAAGCATTCTCTGCTGCCGTATCTTCTCTTGGCATCTACAACAAAGATGGAATAGTAGTTTACGATGGAAAGGGGCTATTTAGTGCTGCTCGTGTTTGGTGGATGTTTCGTGCTTATGGGCATGACAAAGTTTGGGTTTTGGATGGAGGTTTGCCCCAATGGCGTGCTTCTGGGTATGACGTGGAATCAAGTGCCTCCAGCGATGCTATCTTAAAAGCCAGTGCTGCTAGCGAAGCAATCGAGAAGGTTTACAAGGGACAGTCTGTTGGTCCCTCCACATTTGAAGCGAAGTTGCAGCCTCATCTTCTATGGAATCTTGATCAGGTGAAAGAGAACATCGATACCCAGACACATCAACTGATAGATGCTCGAGGGAAGCCTAGATTTGACGGTGCAGTTCCAGAGCCACGCAAGGGAATAAGAAGTGGACACGTGCCTGGGAGCAAATGTGTTCCTTTTCCTCAGGTGCTTGACAGTTCTCAAAAGCTATTACCTGCAGATGAGCTCCGTAAACGATTTGAGCAAGAAGGTATATCACTGGATGAACCCCTTGTGACCTCTTGCGGCACTGGTGTGACTGCATGTATATTAGCTTTGGGTCTTCATCGCCTTGGCAAAACCGATGTTGCAGTATATGATGGATCATGGACTGAATGGGGTGCTCACCCTGATACCCCAGTTGCTACCGCTGTTTAGTCTTATGTGCAGTTCATGGAGGGCCATCTATGGTAATTGCGCTTCACCGCGATT

>Isogroup_**129** CCTTTCTTAACGATACACTGTCATCATCCTTCCATTATCCGAAATGGAACAAGGGGCGAATCATATGCCTGAAGTCCCAGTTGAAAAAGCTGCAAGCAGCCATAGATTCCTCAAACGGAGCGGCCACTCCTGGATTAGTCCACATGACTGATTCCCGTTTGCCGTCGCCTGGCTCAAAAATCTACACACGGGAGCACCAAGTCGAGAACGGCTCGGCCCATTCCACCACCGAATCATCAGTGGAAGAGGAGCCAGAACTCCTTGCGCTTTGAGCTCTCAGAATTTCAGATATGATATTATCTGTCTGTCTGAACAAGGGAAGTTGAGGTGCAGAAGTAGCAGTTTTGAGGTTTGGGCATTCAGTCTAATTCAGATCTTCATGCCGTGCGACAAGAATGGCCACACCATGCCTTTGCAGCGCCTCACTTACTTCAATCATACACGGGTGGATGCTATGTCACTGCCGATCTTGCACTTCTTGCCCGGAACAGCGCACGTGCGAGAGCTCTTGACACCTTTGGGCAGTGCTTGACTTACAGGTGCATCGCAGGGTTTAGCTCTCTGTAGTTAGAATCAGATTACGCCCTTGTTGTCTAGTGGTAGTAGTTGCTAAGTTGCCCTATATGCTATCTCGTGTGTGTTTGATACGGCATAGGTCTTCAGTGCAAACCTGTAAACAATCGCAGCTTGGTCTTTCTGAGGAGTTTCCACAGAACATCTGAGTTGATGTTACATTCTGACGGTGGTTAAGGTAGGGAGTTTTGCAGAGCAGCATGATAAATGTGTTCTGATATCTTTTCCTGAATCTCAGTCATATGTCAAGCCATGAGCCATAGGGTA

>Isogroup_130 CTAATCAACTGCTGCCGTATAATATCAGCATGTATGACCATGGCATCATCCATTTTCACTCTTCTCTTGGGTGTCGTCTTTGGCGTCACAGTCTTCGGTGTTAATCTTTGGCGTTTCAAAGATGATGCATTAGGTGGCAATGGTGTTGAATCAAGCGTCAATCCAGGCGTTCTTCTACCAACTAAAATGGATGCCAGCAAATCGTCATCGTCAGGAATTGCATCAGCAGGTCTTTTACCATGTGGTCTGCTAGGCAAATTTTCAGTAGTCCTACTCTGTGGAGCTGGCAAACTGTCCTCCAAGTGACGTTTTCTGCTAGTAAGGCTACCTATTTTGTTACTTCCATCAGATTCAGTGATTCCTCTTTCTGCAGTAATCTGCCCCAAGTCATTTGTCAGGTGGAATTGAGTATCTGGAGCTGACAACATTGTCTCACGCTCTAGGAAATTTGAGGTCGACAAGTCATTTGGTCTATGATTATATGACATGTATTGTGGTATTGTTCCAGCATTCTGTTGGAAAACTGCTCCAGCATTCTGTTGGAAAACTGTTCCGGCATTCTGTTGGAAAACTGTTCCGGCATTCTGTTGGAAAACTGTTCCGGCATTCTGTTGGAGAACTGCTCCAGTGTTCTGTGGGAATATTGTTCCAGCATTCTGTGGGAATATTGTGCCAGCGTTCTGTGGGAACATCGTGCCAGCGTTCTGTGGGAATATCGTGCCAGCGTTCTGTGGGAACATTGTGCCAGCATTCTGTGGGAACATTGTGCCAGCGTTCTGTGGGAACATTGTGCCAGAGTTCTGTGGGAACATTGTGCCAGCGTTCTGTGGGAACATTG

>Isogroup_131 TTCCTCCTTTCCAAAATATAGTGCATATAAGATTTTACAAAAGTCAAACATCAAGTTTGGCCAAGCATGTAGGAAAAAATTATCACCATCTAGAATAACAAATCAATACCATTAGATTCATCGTAAGATATATTTTCATATGGTATATATTTATTATTATAGTTGTAGACAAATTTTTCACTAAACTTAGTCCAACTTGATAATGTTTGACTTTTCAAAAAACCTATCTTTTGCGGGTAGACAAACTTGATAATGTTTGACTTTTCAAAAAACCCTATCTTTTGCGGGTAAACTTTTCAAAGAACTGATATATGCACCATATTATGGCAAAGAGGGAGTAGTGTTGTGTATGCCTTGGCTCAAGTAACCGCTCCACTTTAATTTACCTGTCATCTAGTTGACCGATTCCTTCTCCTTATTGATTGTCATTATTTCCTTGACTGGAAGCACAATCCTTGATGATCCGGAGGGATTCACTGAAGTCGTCTAAAAGTTGACCATGATCTGGGAAATGTTCTTCGTCAACCCCTAGAATTACCTTGATGGTGTTATTGTAACTCTGGTAGTGCACTATGAGAGCCCCTGGTTGGCCGTAGACGCTAGGCGCAATGGAGACAACTGGGTGCCCATAGAATTCTACTTGTTCAGCCGGACCAATCATGTTTGAAAATGATATGGTGGTGTGCGCTATCATACGATGGAAGATAACACCTGCTGCCTTTATGCCAAGAGTTTTCAAGATAACTTCACAAAGCATACGTGTGAAGATCGCTTCTAGGGATCTCTTCTTCCTGTCCACTGCCTTTTTAGCCTCGCGGACATATTCGAGTGGATCCTC

>Isogroup_132 CTACAAATGTAGCAGTGTGAGGGGCTGCCCAGCAAGGAAGCATGTTGAACGTTGCGTGGACGATCCGTCGATGCTCATTGTGACATACGAGGGTGAACACAACCATACGCGAATGCCAGCTCAATCTGCACAAGCTTAGGGAATCCCTTTGGTTATCTCACACTCTCTTCAAGAAATGCCAACTCAGCCAGCCCTTGTCGATGGGCTGACTGCACTGTTCTTCTAAATTAGGATTGCAAAGTGACAACAGGGGTATCCATTTGAGCAGTTGATATGTTGATTGTCCTCCTGACCTACATGTTTTGTAGTGAGTGCAAGGGAGAGAATTAAAAATGTTCTCCCTTAAGGAGGTATCAAAATGATGTGACATTGTAGAACAAGATGTTAGATAGGAGATATTTTTCTCTGGAGGGAGTGAGCAGAGTTGGAATGGGAGATACAAATTGAGGGCGACAAGTGTGTAAGGTTGGAAATTTAGAAGATTACCGACTCTCCCTCCTTTTGTACATTGATCCCGTGCCAATAGCCTCTGCTTCAATAGCACAGGTTCATGGGGCTAGGTTGAAGAGCTCTCAGAAAGATGTTGTGATTAAAGTCCTAAAGCCTGGTATTGAGGATACTTTGGTCGCCGATCTGAATTTTATCTATGTTGTTGCACGAGTTTTGGAGTTTCTAAGCCCTGAACTAGAGAGGACTTCACTGGTTGCTATCATCAAGGATATAAAGGAATCAATGCTTGAAGAAGTTGATTTTAGAAAAGAGGCTGTAAATATGGAGGCTTTCCAGAGATACATCAATGCAATGGGATTTGATAGGCAGGCCAAGTCTCCATTTGT

>Isogroup_133 GCGGCCGAGGTACCTTGGCAGTCAAGGACAATTTGCTGGTAGCTGGCGGATTTCAAGGAGAGCTAATATGCAAGCACCTTGATCGAGAAGGAATAAGCTTTTGCTGTCGGACAACATATGATGATAATGCTATTACTAATGCAGTTGAGATATTCAATACTTCTAGTGGCGCTGTTCATTTCATGGCATCGAATAATGACTCTGGTGTAAGAGACTATGACATGGAGAGGTTCCAGCTATGCAAGCACTTTCAGTTTGATTGGCCAGTGAATCATACGTCACTGAGTCCCGACAGAAAGCTTGTTGTTATTGTGGGGGATGATCCTAATGGTTTACTTATTGATGCCAATTCAGGAAAGACACTTCATTCCATGAAAGGCCACCATGATTACTCATTTGCATCAGCCTGGAGCCCTGATGGCCTAACATTTGCTACTGGGAACCAAGACAAGACATGCCGAATTTGGGACGCCAGAAACTTATCAGAAGCCGTCCATGTATTGAAGGGTAACCTTGGAGCCATTAGATCAATCCGCTTTACTGCAGACGGGCAGTTCCTGTCAATGGCAGAACCAGCAGACTTTGTCCACATCTTTGACGTGAAGAGCGACTACAAGAGAAGACAAGAGCTGGACTTCTTTGGCGAGATATCTGGGATGTCTTTCAGCCCAGACACAGAGGCTCTTTTTGTCGGCGTGTGGGATAGAACATATGGTAGCCTCCTCCAGTTTCGCCGTTTGTATAACAACAACTCATATCTTGACTCGATGCTTTGAGGTGAAAGCGGAGCAGATTGCTGTCCTTTCAGCTGCACTGAATCTTGCAGCATAAATTAGG

>Isogroup_134 CGCCCGGCGAGGTGCACTTGAATGTTCTGTCCTTACCTGGTAACAAGATTACTGGTGGCATACCAGAGCAGTTTGGCAACCTATCTAGTTTGACAAGCTTAGATTTGGAAGATAACCTGCTTGTTGGAGAAATTCCAGCTTCTCTTGGCCAGCTTACGAAGCTCCAACTCTTGATACTGAGTCAAAACAGTCTAAATGGGTCTATTCCTGATACCCTGGCAACCATATCAAGCTTGACAGACATTCGGTTAGCCTACAACAGCCTCTCTGGTCAAATACCTGCTCAACTATTTGAAGTTGCACGATACAACTTTTCTGGTAATAACTTGACTTGTGGAGCAAACTTCGCCAACCCTTGTGCATCAAGTCCGTCTTATCAAGGTGCATCCCGTGGTTCGAAAATAGGCATTGTCCTTGGAACAGTCGGGGGCGTGATAGGGCTACTCACCATAGGGGCTCTGTTTATTATCTGTAATGGAAGGAGAAAGGGCCATCTACGTGAAGTATTTGTGGATGTATCAGGTGAGGACGACCGAAGAATCGCATTTGGACAGTTGAAAAGATTTGCGTGGCGAGAATTACAACTCGCTACTGATAACTTCAGTGAGAAAAATGTTCTCGGACAGGGGGGCTTTGGGAAAGTATATAAAGGAGCACTTCCAGATGGCACCAAGATTGCGGTAAAGCGGTTAACTGATTATGAAAGTCCTGGTGGGGAGGCTGCTTTCTTGCGTGAAGTTGAGCTGATCAGTGTTGCAGTTCACCGGAATCTTTTAAGATTGGTTGGCTTCTGTACCTTCGGCCGCGACCACGCTAGTCGTGGTGTGGTCGGC

>Isogroup_135 AATGACATTGTTCCTGACCCTGAGAGTGCAGAGATCATATTTATCAACACCTGTGCAATCCGTGAGAATGCAGAGCAGAAAGTTTGGCAGCGCCTCAACTACTTTTGGTTCCTCAAAAGGCAATGGAAAGCTAATGTTGCTGGAGGGAGATCGAAATCTCTGCGTCCTCCCAAGATTGCTGTTCTTGGGTGCATGGCAGAGCGGTTGAAGGAGAAAATACTTGATTCAGATAAGATGGTTGATGTTGTCTGTGGACCTGATGCGTATAGGGACCTGCCTAGGTTGCTCCAGGAAGTTGATTATGGGCAGAAGGGTATCAACACACTCCTCTCACTTGAAGAGACTTATGCTGACATCACTCCAGTTAGGATTTCCAGCAATTCGGTTACCGCATTCGTTTCAATTATGAGGGGTTGTAATAATATGTGCTCGTTTTGCATTGTTCCCTTTACTCGAGGCAGGGAGAGGTCGCGTCCAGTATCTTCTGTTGTCCGAGAAGTTGGTGAGCTCTGGGATGCAGGCGTGAAAGAAGTAATGCTTCTTGGTCAGAATGTAAACAGTTATAATGACACTTCTGAAGTTGAGGAGTTGGAGCCTGGTCAAAACTGGCAGCTCAGCGAAGGATTTTCCAGCAGGTGCAAGGTGAAGAATATGGGATTGCGTTTTGCTGATCTCCTGGACCAGTTGTCCCTGGAATACCCTGAGATGCGGTTCAGGTTTACCTCTCCACATCCAAAGGATTTCCCTGATGAGTTGCTATATTTGATGCGGGATAGGCACAACATTTGCAAACAAATTCACTTGCCAGCACAATCAGGCAGCACAGAGGTACC

>>Isogroup_136 ATATGCTCCTGTGCACTTCAGAGGTGGTGACTGGAAGAGTGGAGGGAGTTGCCACCTGGAAACGCTTCCAGATGTGACACCTTTAAAATCACTGGAGGAATGGGCAGACCAGCTTCAACCTGTGCAAAATGTTCTTGGGAGCAGCATCAGACCCAAGTTGCCTGGGTTAGCAATACTGAATGTGACACAGATGACAGCGCAGCGAAAAGACGGTCACCTCTCGATATACATAAGCCCTTCAGGACCTGTCCCTCTCCACAAACAGGATTGCAGCCATTGGTGCTTGCCTGGAGTCCCTGATACTTGGAACGAGCTTCTCTACGCTGTCTTCATGAAAAGGCAAACGATGATGGACCAAAACGTTTCCCTTGCTGGCTCAAGAACACTGAACACTGGTTGACTAAATAGATGAAGTAGGCTACATGATGCTTGCCCGGTGAAGCTTGGAGTCTATGTCCTGCAGGTAGGTCATCATGAAAAAGCAAAGATTCGTGATTCTCCCGCAGGACGACTCTGTCAATCTGCTTCCCAGTCAGGTAGCCCAATGGAAAAAAACATTTTTGTAAGGTAGGAGATGTGGCTTGCAGGAAGCTGCACCACTCTACTATGTTGTGTGCTGAAAGGTTAGTGAGTATAAGAGCAAAATTTAAGGAATGCATATAGCTTACTGTGATTTGTATGTTTCACAGAGGCGGCAAAAGTGTCAGCCGACGTGATCATATATGTTAGGGGCTGCAGTCAGTGATGTAAGTAAGTAGACTGGGTGCATTGATATTGGGGGTATTACCATGGCGATTGTATGTAATACAGGAATTTCCCTCCCTGATGA

>Isogroup_137 CGAGGTACTTGTTGAGAGGAGCATCTATCTCGAACCTCCGGGGCAGGTCAGGATTGGATAAAAACAAGCGCCCGTATGCAACCAGATCAGCATAGCCATCAGCGATTGCCTTATTTCCATCATCCCTACCATATCCACCAGCTACAATAAATGTTCCCTTAAAAGCATCCCTTATGGGGCGAAGACTATGGGGAGTTTCAAACTTTTCACCAATATTCACCATCCGTGGTTCCACCATGTGGCAATAGAGAATTCCAAATTTGTTCAGTGCATGTGCCATGTATAGGCCTAGAGCTTCTGGGTTTGAGTCTGATGCATTTGAGTAACTTGCAAAGGGTGAGAGCCTTATCCCAACCTTATCAGCTCCAATTTCATCAACTACGGCTTGGACTACTTCCAGCGCAAAACGGCAACGATTCTCTAAGCTCCCACCATATTTGTCAGTGCGATCATTGACTTGGTCCTTTAAGAACTGATCAATCAAATAACCATGAGCTCCATGAATTTCAACACCATCAAATCCAGCTTCAATTGCATTTCTAGCGGCAACCCTGAAATCATCAATGATCGAAGGGATTTCATCAGTCTCTAGTCTCCGAGGAGTTGAAAACGTAGCCACATCTATGCCGTTCGCTCTCGCTACAGGTTTGACTGGCTTATCGGTGCTCGAAATTGGAGCCTGCCCATTAGGCTGAAAAGTATGATTAGAGACTCTTCCTACATGCCAAATCTGACAGAAAAATATTCCTCCTTTCCCACGAACCCCGCTCACGATCGGCTTCCATGCTTCTACCTGCTTCTTTGTCCAAATGCCAGGAGTATCTTTT

>Isogroup_**138** CAGTGAATATAGCTGCAAAAGCAACGGTAGCAAAAAGCACAGCAACAACAGTTACAGAATTCGTTGCATTATTGATGCCTTCTCTGTGCAACTTTCTAAGTTCCTTAGCAATGCCATGCACATTTTTGTTTGTTTTCCTTGTCTGTTCAAGCTGTGTGTGGACATCTTTCTTTATCTCCGTCACTGTCTTCCTGAGTTCATCTCGAGGCTGATTCAGCTCCCTAGATCTGAGAGCGCCATGTTGAGACAAAATATCCTTAATTTCACAAGACTCCTCACATTGTGGCAACCCTTCAGCTATATCATAAGCAGTCTTGTGATCTCTGTTCAGTGCATTGACATGGGTATCTGGTAGCCGCAAAAGAACAATAACTATCTCTGCTCGCTTTTTCCTTGTTGCTACATGCAAGGCAGTTTTTCCATTTTTGTCTGGCAGCATTACGATTGCTGGATCAGCATCAACAAGTGCTCTAAGAACATCACAATTAGTTCCTTTTACAGCCATATGCAGTGCTGTTTGACCTTTCTTGTCATTTCGTCTTGCTAGCTGTGGATCTTTCTCAAGTAATGCTTTAACAATTTCTGTATGTCCCTGTCGAGCAGCAAAATGTAAAGCATTTTTGCCATTGTCTTTGGCCATTTCCCCCAATCCAAAATCATCTTGTTCTAGTAACAGTTTGACAACTTCAGCATGTCCCCTCGTAGCTGCTGATATAAGCGGGGTAGTATTTGCAGGGCCAAATGTTTTGGCAATCATCCTATTGTAACGTAACATTTCCTGCACAACAGCATGATGCCCTTCTCTAGCCGCGACGTGCAGCGCGTC

>Isogroup_139 TGCCAACGGCAGTTAAAGATGGAGAACCTTTACCTGAGGGTCTCCTTTGGCTTCTTTTGACCGGAAAGGTGCCAACCAAGCAGCAAGTTGATGCACTATCAAAGGAATTGGTTAGCCGTTCGACTGTTCCAGGTCATGTCTATAAGGCGATTGATGCTCTTCCTGTTACTGCTCATCCAATGACACAGTTTACCACTGGAGTGATGGCACTCCAAGTTGAGAGTGAATTTGCAAAGGCTTATGACAAGGGAATTCCTAAATCAAAGTTCTGGGAGCCTACATATGAAGACAGCTTAAATTTGATTGCCCGGCTTCCAAAAGTGGCTTCATATGTCTACCGGAGGATTTTCAAGGACGGGAAAACTATTGCAGCTGATAATACACTGGACTACGCAGCGAATTTTTCACACATGCTTGGTTTTGATGACCCCAAAATGCTGGAGTTGATGCGCCTATACATAACAATTCACACTGATCACGAAGGTGGGAATGTCAGTGCTCATGCTGGCCATCTGGTTGGAAGCGCTCTGTCAGATCCTTATCTTTCTTTTGCAGCGGCACTGAACGGTTTAGCTGGGCCACTGCATGGCCTGGCTAATCAGGAAGTGTTGCTATGGATCAAATCTGTGATGGAAGAAACCGGGAGTAACATTACAACTGATCAGCTTAAAGAATACGTATGGAAGACACTGAAGAGTGGAAAGGTTGTTCCTGGCTATGGTCATGGAGTTCTACGTAATACAGATCCACGATACTCGTGCCAAAGGGAGTTTGCACTTAAGTATTTACCCGAGGACCCACTTTTCCAACTGGTCTCCAAGTT

>Isogroup_140 CAACAAATGAATTGGATGCTCGCATAGGTTCAGCAAAAAATATGGGTGAACTCCTGCTTTTCCTCCTACTTTTCTTATTAGCTTCTTCTGGAAGAAAGACCTGCGGTCTGCAAGATGTCAGACAAGACAAATCAAAGTCTGTCAAGGAGATGTGCCCGTCCCTGTGAAGTAAGATATTCTCTGGCTTCAAGTCTCGGTAGATTATTCCTTGGCAATGCAAGTATTCAAGTGCAATGACAACTTCAGCAGCATAGAACCTGACGGCATCTTCCCGCAGAACCTTTAGAGGTTGTCTATCTAGGAGCAGAAAGAGCTCCCCGCCAGGGTAGTAGTCTGTAATGAGACATATATGTGTCTTTGTCTGAAATGACGCATATAATGTCGGAAGGAATGGGTGATCCAACATATCAAGGATTTCTCGTTCAGCGTTAGCTCTATGAACCTTATTGCGGTTAAGCATGACGTTTTTATCCATAGCTTTCATGGCAAAGTATTCACCTGTGTTTAATAACTCCACCAAGTGCACGCTTCCAGTGTCACCAGATCCCAAAGGCTTTACAGGCCTGAAATGCTTCAAATCAATGTTTTCTCCACCCTCAAGAACTTTTTGGATGGCTCTCCATGATGCAGAATCCTTCATATGTGGCTTTGGCAAAACTACTTTTGAGTGGTTAGCCCACAAATCCTCTGGTCTCAAATTAGCATCTGGAAGTTCTTTTGCCGCCTCATCAATATTTTCTGCAGTTTTCTTAATCAGCATGACTCCCTCTCTCTCGGCAGCATCTCGGACATGCTCAGTTCCATCCAACTGAACCCCAATAAA

>Isogroup_141 ACCACACCACGACTAGCGTGGTCGCGGCCGAGGTACAAACAACTGACTCTGGAAGAGGCTGAGGAGAAGATGAATAAGAGAAAGACTAGTGCAACTGGTTATGAACGTTGGATGATGAAAGCAGCTACAAATGGTCCAGCTGCCTTTGGCTCTGATATGAAAAAACTTGAGCCTACTAATGATGGGGAAAAAGAAGGTGCTCGTCCTAAGAAGGGGAAAGACAATGAGGAGGGTAATAATTCTGATAAAGGTGAGGAGAATGAAGAAGAGGAAGCCGCTCGCAAAGATAGGCTTGGGCTCTCTAAAAGGGGCATGGATGACGACGAGGAAGGTGGAAAAGATTTTGATTTGGATTTGGATGATGACATTGAGAAAGGCGATGACTGGGAGCATGAAGAAACATTCACTGATGATGATGAGGCTGTGGATATCGACCCAGAGGAACGGGCTGATTTAGCTCCTGAAATTCCTGCCCCACCTGAAATTAAGCAGGATGATGATGACAACGAAGAAGAAGAAGGTGGTTTGAGCAAGTCAGGGAAGGAATTGAAGAAGCTGCTTGGCCGTGCTTCTGGACAAAATGAATCAGACGCCGATGAGGATGATGAAGATGATGATCAGGACGATGAGTCATCCCCAGTGCTTGCTCCAAAGCAGAAGGATCAACCGAAAGATGAACCTGTTGATAACAGCCCAGCTAAATCCACACCTTCATCAGGACATGCCCGTAGCACTCCTCCTGCATCGAAATCAAAACAAAAGAGGAAATCAAGTGGTGATGATGCAAAAACATCTGTCACGTACTCGCCGGGCGGCCGCT

>Isogroup_**142** CACCCAGCTCTATTACCTTATTCATTATCTTAATAGCTAACTCAACCTCCCATTCTTGTGACCATTTGCGTGGCATCTTATTCTTTAACTCTCTCTCCCATGTCCATCCCCAGGCATCCGCAGTAGTATACATATCTGCTACATCAAACATTCGTGTCTCACGCATAACCTTGAAAGCTTCAATTGGATCTTCAGGAAACCAATCTTCGTCATCATCAATCTCGGGTATTTTTTTTAACTTCTTTGTTGAACCAGATGTCTTCTCTAGATCCTTCAATAACTGGACTCCTATCATTTGAAGATGCTGATTTGGTCCTTTTGCTGGTTTGTCCTTAGTCTCATCATCACCAGCTTGATTTCTTGTTCGATCAATGACCTCTTCATCATCTATCTCATCTTCCTCTGATTCTTTGGCGTCATCATCATCATCGTCCTCATCTAACTCGTCATCCAAATCTGGATCTTCTTTGCTATCTTCTTCACAAAGATGGTTTCGAGTTTCTCCCAAAAAACGCCGTTTCCAGAACTCTGTATTTCCATCTTCTAGCTTGATCCGTGAGATCATTTCATCTAACTCTGCATCGACCTCTTCTTCATCTTCCACGGGAGGAACCCAAAGTGGTATACCACGTGAACGATTGATTCTCCTGGCCTTTTGAACTCGTTGGTAAAGAACCTGTCTAGTTCCATCTGTAGGCAACCCTTGCGCCTCCAGTTCAATTTTCAACTCAGATACTACCATTTTGCTGGCTGCCTTGGGCTTAACAACATTCTTCTTTGGCCCTACAACTAATTTCTTTAATCTTTCTACTACTCTTTG

>Isogroup_143 CTAGGTGATATCTCGTTATCCAGGACAACCTGTTTGGCCATGTCAAAATTCCCAAGTCTTGAATATGCATTAATAAGTGCCATATATGCATGCTTGGTCATTTGAATACCATCTTGTTGTAGTTCATCAAGATACTTTGATACTTTCTCTTCAGAATCACAGTTCAAGATCAAATAACTAAATGTTTCAGAGTCCGGCTTTACTCCAGCACTCTGCATTTCTGCAATGACCCTTTCTGCTCCATTGTGATTTTTCTCCCTAAAATATCCAGCCATGATAATATTATACAGGGTTACAGTGGATGTCTCCCCAGACTCTTCTGCATCTGAGAGTATATTGTAAGCGCCATCAAAATCTTTCAGCTTTACACTTAATGCTATCATGCTCCTGAATGTTTCGTTTTTCAGTTTCAACTTGTGGCGACTCATCAGATCATATAAAGGCCGAACCATGTGAAGCTCGTAGCTCTGCTCACAGGCTTGTATTATTGGATTAAGCATTTCGATCGGCACATTCGGATTGGATTTACAGAGGCACTCAGCAACGTCAAGAGCTAAGCTAATCTTTGATGAACTGCAACAGAAAGAAATGATCCTGTCATATGCACCAACAGAAGGTGCTAGCTCAAACTTCTCTTGCCATTTATTAAACGTTGCTATGACATCTTCAGCCACTATATTCGGTATGTTTGCAGCATAGTCAAAAGTTAAGGATGAAACTTTTCCATCAGCACATACTTTTGACGTCATCTCCCGAAGAAGTTTCTCGGCACAATTCTCCAGTTCAAACCTGCCCATATACTTGAATACACTAGATAT

>Isogroup_144 AGCGTGATCGCGGCCGAGGTACCTTGCAACTGGAGAATAAATATCTAACGACAAACATACAGGGTCAAGAAGATGGAAGGGTTTGGCTATGTATCATATGTAAGCAAGAGAGTTCCTGTCACCGGCAGAATGCGGCCGTCCTCTAGTTGGTGTAGGTGGCCGTTGTATGTTAAGTTCCTGCATCCATGTGGCATCCATGTGGCGTTCAGGGGATGACTCAGGAGAATTAATAGGTGGTGGAAGTGGATGAATTAACTTCGGGACAATAACTAGTCCCCTTCCCACCACAAGTATGGCGCCAGCGTTGTTTGCAGTGCCACAATAATTAGTGGCTGAAAATAAAGTGATCAACTGTCCAGAGGCGAAACGCTCAAATCCATCCATAACACACTCATGAGCTCTTATGATCAGTTGCAATTTGTTCCGTTTACAGAATTCTGTCACTCGGTCTGGCCCAAATGTTACTAAGCCAGGACCTCGTGCATTTGGCCTCAAACCCTCTACACTATCATTCTCTGTTGGATCAGACCATAAAAGGTCCATGAGGATAATAGATCCAACATCCATTGTGATAGGCCTTTCAATTTTCTCAATTTGCTCCACACTGTTTATTGACCTCCCTATGCCACCATGCATGCAAATTATTTTCTTCTCTATCATGGCAGCCAGTGGGAGATAATTAAACAGTTGGTTGAATCTAGTCCAAGCCCATATACCATCACTTTCACCCATTCTCTCAATGCATTCAAGGCGGAAGCCAAAAAAGAGCATTGATGTCAGCTGCCTCGTGATTTCCTCTAATCAAGTGGACATGTTCAGGG

>Isogroup_**145** TTGCTGCACATTCCACTCGAGTAACTTATAACTGCCAAGATTTTCAGGCAACTGAACATGATGTTGAGTTAGCCGATAAATGATACCCATCGCTGTTGCATTAGACATAGCATTAGAAGATCCAGCAGGGAAAATTTCCCATCTGATTAGAGTAGAGTTTCCTTGGCCTGTAACATTCATAACTTGAACCTGACGGGAATCGATCTCCAGCTCTTTAGCTAACAGATCAGCAAGCTCTTGCAGGTGAGGTTTCAGGTTTGGATAAGTAACATTTATCGACATATAAACAGTGATGAGACCAACATCAAATTCTGGCAAACCACTAGGAGCAGGAGCTGGTGCAAAATCTGCTTGTGAACCTGGATCACTTGAGGATGCTGGTGAAGGAGCTCCACCAACATGGAGCCTCTCCCACAACTCAGAACAGTTAGTTTTCCAAAATCCAATCTTGTCATTGTGGCGGTCATAGGTTACAAGAGTATTACGAACAACGATTCCCCCTAATAGAGTTGTTGGATCTTTCCCATTTTGGAACACGCCCAAGCAATAAGCCCCTTCAACTTTGGAGTGCCGGAACAAATAATTTTCAGGTGAAAGTGAGAGCTTCTGTCCATTTCCAAATACCATGTCAACATCTGGAAATACCTCTGATAACTTGGAGACGTTCCTTCCAGCACCTGCAAAGCAGATGTCCTTGTAATTTGGGTCAGGCCCACGGATTTTCTTGAGAGCGTTTACTTTGCTTGTCACAGCATCTTTAAAAGCTAGAAAAGCTTGTTCTGGGAGGTATGCGTATGTAGTCCCACTGTCCAGCACA

>Isogroup_146 CTTATACATTATACATACTTATATATATAACCACTTCAAACACTAAAAGAAAGGACCACCATGACAACCATCGTATATCTTTAAAGAGACTGCATACTTCAATGGAGTAGCAAACAAGCAATGCGTTGAACAAAGGACGCAAGGATTCATCTTCTTTACAGATACTTGATGAGAAGCCAAACAATCACTACGAGCAGCACTATGGCGGCAACAGATAGCAGCAAGGCCATGCATGAGCAGCCTCCTTTGGTGCGCTTGTTCAGAATCGCAAGCTTCTTTTGCACACGCTGAAGACGGGAGCTTGTAACGTCCACGTGATCTTCAAGGTCATCAATCAATCTTGTGTGCAGAGTAAGTTCTTCATTGACTGCTAATGCGATATGCTTTGTGCTCAATACTGTCTCCTCCAGCTTTTCAAGACCCTCATCTTGTTCTTTCATAACTTGCCTCTGAAGGCCAACAATCCCTTGGTTATCCAACCCAGCAACTCTGCTCATATCATCAGCTGCTTTCTTACCCTGACCAAGTAAATCCTCCCTGTTTGCAAAGTTGGACACGTTGAAACTTGTCCCCATCTGCTTTGCTTTCAATTTCAAGTTCGAAAGTGCATCTTGGCGCTTGTGCATCTCCTTGTCAGTGAGTGACTTTGGAGGAATCCTGGACAACAAAGTCACCAAACTATCCAGTCTAGTCCCAAGAATAGTTATTTTCCTCCGAATGGCTGATGTGTGACGCATAATTTCGGGGCCTGATTGTGGAAGAGAACCTCTATCGGCAATCATGGAGCTTATGTCATCAACAAGCTTAGATGCTTCAGT

>Isogroup_147 CGCCGCCACCTCTTCCCTTCTCTCCTGCTCTCTTCCTCGACCACCACCTAACCACCACGATGACGGCCCAGACTGCCGAGGAGCTCGCCGCCCAGATCGAGCAGGAGCAGCTAGAGGCAAAGAAGGCAGAGGCAGAGGAGGTCGTAGTTGAGGATGATGAGGATGACGATGACGACGATGACGAGGATGATGACAACGATGATGTGGAGGGACAAGATGGTGATGCTAGTGGCAAATCAAAGCAAAGCAGAAGTGAGAAGAAGAGCCGCAAAGCAATGCTGAAACTTGGCATGAAATCCATCACCGGCGTAAGCCGTGTCACCGTGAAGAAAAGCAAGAACATATTGTTCGTCATCTCAAAGCCAGATGTCTTCAAGAGCCCAAACTCCGAAACATATGTTATATTCGGCGAGGCCAAGATCGAGGATCTCAGCTCCCAGCTGCAGAGCCAGGCTGCTGAACAGTTCAAGGCTCCTGACCTGAGCCAAATGATTTCAAGTCCTGAGGCATCGGGTGCAGGGCATGACGACAACGAGGATGTCGATGAGGAGGGAGTCGAGCCAAAGGACATCGAGTTGGTTATGACGCAGGCGGCTGTCTCTAGGGCCAAGGCCGTCAGGGCTCTCAAGGCTGCCAATGGAGACATTGTCACCGCCATTATGGAGGTGACAACCTAGAGGTTTTTTACTTTTTTTCCCCATAGTTAGACTGTTTCTCTTATAAGCCGTTTGCAGCTTGAGTGTTATCTGCGAACCATATGCTTGGATAAGTTTGTCGCGATTGCCTCTCCTATAAGGCCTTCTCAAACGTGATTCTGC

>Isogroup_148 CAAAGCTGCAGAACAAGCTGATGCTGATTGGTATCGGAGAGCTCAACTTGCTCTTCAAAAGGGTGATGAGGATCTTGCTCGTGAAGCCCTTAAACGGCGTAAATCATATGCTGACAATGCGAGCTCCTTGAAGGCCCAGCTTGATCAGCAGAAGAGTGTCGTTGACAATCTTGTTTCAAATACCAGGCTTCTTGAGAGCAAAATAGCGGAGGCCAAGCAGAAGAAAGATACTCTGAAAGCCCGTGCTCAATCGGCTAAGACCGCAACAAAAGTGAGTGAAATGCTGGGGAATGTAAATACAAGTAGTGCCCTGTCAGCATTTGAAAAGATGGAGGAAAAAGTTATGACGATGGAGTCCCAAGCCGAGGCACTTGGTCAATTAGGAGCTGATGATCTAGAAGGAAAGTTTGCAATGCTTGAGACTACATCGGTTGACGACGATCTTGCACAAATGAGAAAAGAACTCTCCGGGAGCTCTTTGAAAGGCGAGCTTCCTCCAGGTAGAACCATGGCCAGCAAACCAGGTAGCCCTTTTCGAGACAGAGAGATTGAGAATGAGTTGAATGAGCTGCGGAAGAAGGCCAAAGAGTATTAGACTTCCCAGGCTATATCCATGTGCCAGGCTTGATGAAAAATCTCCTACCGAAGAACCCAAGAAGTATAGCCTTTGAACCTCCGGTGCATATTTGTTCGATGTGCATTTAAACCTCTGGTGCACTTTTGTTCGATGTGCATTTGAACCTCCGGTGCACTTCTTTTTTGGTAAAGGTTTGGGTGAAAAGTTCTCTCTGCATTTTTGCCGTGAATGACACTG

>Isogroup_**149** TGAAAGTATACCTTATCACGATTATACATTTATATTGCAACGGTCCGTCAAAAACATGTGCTAACTGTGCGGAGTGTCTTCATTTATTTGGAACCCACTAGAGTTTCCAAATACTTCTCCACATTGTCCAAACCGCATAGCTCCTTCACCACAGGCATGATTGCGGGAACTTCCAACTCAAAGAAGGAAGGAAGTGAAGAAGATCCAGATCCAGAACAGTGGATTTTGTTCCTTGCTGCAGCGATCGCGTTCCTTGTTGCACAGTGAACTGAAGATGCAAGAAGCAAAGGTGGCTCCCCAGATGCTTTTGAAGATAGCACTCTCTTCTTGTGAAATCCACTGTTAAGAAGCTCAACATTGAACTGTCTAGGGATGGTGTCCACAGTTGGGATCTTGTATGTCCAAGTCCCATCCGAAATGACCAGCCCATCCGAATTGGTCACATACTCCTCGCTCATGAAATAACCAATTCCCTGAACGAAGGCCCCTTCCACCTGCCCCAAGTCCACAGCAGGATTTAAGCTCTGGCCACAGTCATATATAAGATCACTACGCAGAATTGTAGTTGCTCCAGTGAGAAGATCAATTTCTACCTCACTAGTAGCAGCTCCATAGTTTAGATATGAACCAGAAGGCCCAGGAATGTAGTATTCTCTTGCTGATAAATCCACAGCAGCCATTTTTGCCTGTGAAATTAGTCCATCCCATGAAACTCTGCCTTGCTTTTCTTGTAATTTCTCCTTGAGTGGTTTCAGCCTGTCAACCATGATACCGCAGGCACGTCTAACTGCTTCACAGCTGCATTCTGATGTG

>Isogroup_**150** CGCCGACCACACCACGACTAGCGTGGTCGCGGCCGAGGTACCATTTCCTTTCCCGTATGGTCCATATGTGTCAAGATTGGTGACAATCTTGAGTGAGTTCACAACAACATGGCCTCCAAAATCACCCGTTGTTCCCATGATTTTTTTCACGGTCTCAGTAGGTTTAAGCTTGATCGTTTCAGAAATCCCACCATTTCCACCCCATGGACTAGTGGTGTGTGTCTTGCCAGCCTCGTCAACATAGGAAAATGAAATTGAATCGATGACATCTCCGTGTCGGATTGTGACACTCAGTAGACGCCGGGGCTTGTCTTCAACGTCAACAACGTTCCCTCCATTCCCACCCACCAGCGGTTCAATCTTTACAGCATGGGTCTGTTTTGTGGAGATGGGGCACACAAAACGGATATGTAGCTCCCTGACCCCTCCTGGGACTTCTGGGGACGTAACATATACGTAGGTAACAGTCCCATTAGCACCAGCAAACTCTCCAGTTCCTCCAACCACTGCCCATTCGCCGTTTGATTGAGTCAGAATTCCGTCTAGTTTGAGACCAGAATCCTTGAACCTAGCGTCATTGAACACCAGGTGGAAAGAAACAAGCCAGCTTGCTTCGTTTATTCCAGCCCCAAGTTGTAGACCCTTGGCACGTGCCACCAGCTTACCCTTGGATGGAATATCATCGAAAACGGTCCAGTCGATAGCGACGGCGTAACCAAAATAATTGGCTTGGGTTCCTGGCACTATGATTTTCTGGTTACCATCTGGCGTCCCTTCTATATTCTGTGACATGTACCTCGCCGGGCGGCCGC

>Isogroup_151 ACCCCTATCCTCAAGCAGGTCAGGAGTGATGTTACACGTTATAGATATGGTGACGAGCAGCATCTTGATGAAGCGCTGAAGCGGATCTTTCAATAGTGGTTTGGGTGGTGGCATTCCAAGACGTAGTGCACCTATATTACAAAAAATTCGAGAAGAAGTAACTGATGACGGGAAATACTGTAAGCGTGGTCGCGGCCGAGGTGGAACATGCCCACCGCGCAGGTCAAGCGAATCGCTGAGGACCCGCAAGACCAGACGGAGGAGTCAGCGCTCGTGATCTCTGGTATCACCGGGAGGATTAACAGGGCTGTGTGGGGGCCGGGGAATCGGACCATCATCACCGCTGGCGAGGATGCCACCATTCGTATCTGGGACTCTGAGACTGGAAAACTGCTGAAGGTGTCAGACAAGGAGGAGGGACATCAGAAAGCAATTAGTTCACTGTCGAAATCCTTAGATTGGTCTCATTTCCTTACAGGCTCCTTGGATAAATCTGCTAAGCTATGGGATGCAAGAACACTGACCCTGATAAAGACATATGTCACAGAGCGACCGGTTAATGCCGTCGACATATCTCCAACCCTTGATCATGTGGTTATTGGATGTGGTCAAGATGCTATGAATGTGACTATGACAGACCGCCGTGCGGGTAAATTTGAGGCTAAATTTTTTCCACAAGATTTTACAAGAAGAAATTGGTGGTGTTAAAGGACATTTTGGACCAATCAACGCATTGGCATTTAATCCTGATGGCCGGAGCTTTTCGAGCGGTGGTGAGGATGGTTATGTAAGGCTGCACCATTTCGACCCTGA

>Isogroup_152 ATCGTGGAAGTCTAGAGTGCACCAGGTGCGTCCCTCATGCTGATATTCAGGCTGTTTCTCATTGTTCTTCGCCCGACACCCATATTGCCCTTGGTCATCATGGCAACAAATTCTCCATAGTCAATGCGGCCATCATTGTCCTGGTCAGCTTCTTTAATGACATCATCAAGAAAAGCATCTGGCATGTTATGCTCTTGGCAAGCTTGCTGCAGTTCGTCTACTGTGATGTAGCCACTTCCATCTTTGTCAAAATATGAAAAGGCTGCCACTAGATGTTCCTCGCGCTCCAGTTTATTCAGATGCAATGTTGCAGCAATGAACTCTATATAGTCAATGGTCCCGCTGTTGTCCACATCCGCCGCTTCCATAAGATCACGTATCTCAGTATCCTTTAGTGTGGAGCCGTATTTTCGCAAGCCTTCTTTGAGCTCATCATACGTAATTGCACCGCTGTTATCAGCGTCCATAGTCTGGAACATTTCCTTCAACCCAGCAATTTCCTCCTCTGACAGGCTCTCAGCTATTACCCGCAAGGCCATCTTCTTCAACTTATTCATTGCAGAGAATTGCTTAATGCGAGATAGGACAGCTGTGTCAAGAGGACGGTCAGGAGCAACTCCATGATCACGAATCCATGGATGGCATAGAACTTCATGTGCTGACAAGCGTTCTGCAGGGCGAGGATTGAGCATTCTTGTTATCAGATCTTTTGCACTATCAGAAATCACAGGCCAGGGTTCAGAATCGAAATCAATGGCACCTTTCAATACAGCATCAAATATTCCTTGCTGTGTCTCTGCCCAAAATGGGGG

>Isogroup_153 ATTTTCTCCTACTGATTCAACAAGGTTCATCACATTGGAGAAAAATGGGATAACTCTTGAGCTGTTCCCTCATGAAGTTTCTACTGATCAAATTGCTGCCATAGAGGATGCATACTTTAGTATGGCATCTGCTTTGTCCAAGGCTGATGGTATTGACTACACAGATCCTGATGAGCTCGAATTATTAGTGGCGGCTCTTATTGACCTGGATGCCATGGATGGGAAAAAGAGTGTTTCCTTGATAGCTGAATGTTCAAGCTCTCCAGATGTTAACACCAGGAAAGCTTTGGCTAATGCATTGGCTGCAGCTCCGTCCATGTGGACCCTAGGGAATGCTGGGATGGGCGCATTACAGAGATTGGCTCAAGATTCCAATTACGCCGTAGCTAGCTCTGCATCAAGAGCCATTGATGAGCTCAGAAAGCAGTGGGAGCTTGAAGAGGGTGACAGTCTGAGGTTTGTGATGAACCAAAGCTTGGATTCTGAAGAGACAGATGATGACGGTTCAGCAGCAGATGACATCCCAACATGACGCATAACGTTGCCATACAGACCAATCAAAACCCGGCCCAGCAGCAACAGATACAGGCTGTTAGATTAGATCCGAAGAATGTGCAGATGAATCAGCCTCATGTTAAGCTGCAGCACATAATGGACCATCTCGCCAGAAGGCATGCCAAGGTATCTTGTAAAGTTGTAAGCAAATGGGAAGTGTGTGTAATGATAACACATTTCCTGGGAGGTGCAGATGTATAGATGAAGCAGAAGAATATGGTGTAAGGATATACTTTCCCAGAGGCCTAT

>Isogroup_**154** CAGAACAATCGATTATTTATATCAGACAGTAACAACAATCGAATTGTGGTCACCAATCTGGGCGGGGAATTTATATGTCAAGTTGGAAGTTCTGAAGAAGGACTACTTGATGGTCAATTCGATGCTGCCTCATTTAATCGCCCTCAAGGTCTTGCATACAATTCCAAAAAGAATATCCTATACGTTGCAGACACTGAGAATCACGCACTACGAGAGATTGATTTTGTTAATGAGACCGTCAGAACACTAGCTGGAAACGGAACCAAAGGTTCCGATTACAAAGGAGGAGCCCAGGGAACTAATCAGGTTTTAAATTCACCATGGGATGTCTGCTATGCTCCCTCCGAGGAGACTGTGTATATTGCCATGGCTGGACAACATCAGATCTGGAAACACAACATACTTGATGGTGTAACAGAAGTTTTCAGTGGTAATGGCTTGGAAAAAAATCTGAATGATTCAAAAAGCTCGACCAACACATCGTTTGCGCAGCCATCTGGGATTTCATTGGATCATGAATTGCAGGAGTTATTTGTTGCTGATAGTGAAAGCAGTTCCATTCGAGCTGTTAATTTGAAGTCAGGCGGGTCAAGATGGCTTGCTGGGGGAGACCCAAATTTTCCAGATAATTTGTTCAGGTTCGGAGATCATGATGGGACTGGATCAGATGTGCTACTCCAGCATCCCTTGGGTGTTGTCTATGCTAGCGATAAGCAAATATATGTTGCAGATTCTTACAATCACAAGATAAAAAGGTTAGATCCTGTCACAAGAAAAGTTACAACGATTGCTGGTACCTCGGCCGC

>Isogroup_155 ACGCTGCTTCACGGGAGGCAAGGCCATCGAGACCTTGGCACCATGACTCATCGTTGGAACCAACAAGTGGCACAAGACCCTTGTCAACCTTGATACCGGGAACAATTCCCTGCTCGACAAGAATGTCAACGATCTTCTTTCCATCAACAGTCGACTGGTAGAGGGTCTCCTCAAAGAGGATAGCACCAGAGATGTAATTTCCCAGGCCTGGTGGAGTGACAAGGAGGGTCCGGTAAGCCTGCCGGTTAGCCTCGGTGTTCTCAAGGCCAATCGATGCAAGTCTCTTGCCACAGGTGGCGTTCGACTCATCCATGGCAAGGATACCCCTTCCTGGTGATGCGATGGTTTTCGCGGTTTTGACAAGCTCATCGGCGTATGCGCTGGCACGGACAACCATGGAGACGGTCATCGGCTTGGGAGCAGCAGCCTGGCGTGTGGCGCCCCATTCGGATTTCTTGGGAAGGAAAGACGATTTGAGGAGAGTAGCAGACGCCATTGTTTCTGAAGGCAAGCAGATCAGGTGCTACCCTCTTCGACACACCTTGCAATTGCAGCACCACACGATACGATATGATATGCTGCGTGTAAGCATCTGTAGGCAGCTTAGAAGCCGTGGATCTTGATGAGCTCCTTCTTCGCGATCCCAGCCTGGACTAGGAAAGTAGCAACATTCTTGCGCTGATCACCCTGAAGCTGAATGACCTGACCTAGCTCGGGGTCCTGGACTACTGTGCCATTGCAGCAGAATTCCTTCTTGAGATCCTTGAGGATCTTGTTGTAGCTGTACTCGCCGGGCGGCCGCT

>Isogroup_156 CTTGTAACATCGGGTGCTGTTCTTGCTCTCTGCTCGACCTTGATGGGATCAATTCTGCCACAGCCAAGAATATTGATGGCAATGGCGAGAGATGGGTTGTTGCCATCCTTCTTTGCTGATGTTAGCGAGAAGACCCAAGTTCCTGTCAAGAGCACAATCATAACTGGCATCTGTGCGGCTTCTCTGTCTTTCTTCATGGATGTTTCGCAACTGGCCGGAATGGTCAGTGTAGGCACACTCCTCGCGTTCACCATAGTCGCTGTCTCCATCTTGATCCTCAGATATGTTCCACCAGATGAGGTGCCCCTGCCATCTTCTCTGCAAGCGTCATTCCGTTTGAACCAAGAATTTGATGAGGAAAAGGCAAGAGATGAGAATCGTGAACAGGGGACATCGGATGTGATTGTAGTAGAATCAATCAATGACCCTCTTATTGAGAAGCAGCTATATGCAAGCAAATTGGACGAGGTAAAACGGTGCAAAACAGCTGCTTGTAGCATAGCATCTGTATGTGCGGGGGTTCTAATCCTGACGTCTTCTGCTTCTGTAACATTCCTGCCATTCCTGGTGATGTGCTTCGCCTGTGTCTTTGGTGGCCTGCTCCTCCTAGCTGGTCTTGGTATGCTCTGCTGGATCGACCAAGATGACGGAAGGCACTCATTTGGTCATTCCGGAGGATTCATCTGCCCATTTGTTCCAATGTTGCCAGTGATGTGCATTCTCATAAACACATACCTGCTAATAATCTGGGGGGTGGCACATGGATGCGAGTTGGGGTATGGCTTGTGATGGGGGTCTTCGTG

>Isogroup_157 AGCGCCTTGATCTCCTCCACGTCGTCGTAGCTGCCGAGGAAGATCGGGGATCTCTCGTGGATATTGGTGGGGATCAGGTCGAGGGACCGTCCTTTGCCTGTGAATGACTGGCCTCCAGCTTGCTCCATCAGGAATGACATGGGGAAGACTTCATACATCACACGGAGCTTGCCATTTGGGCTCTTCTTGTCTGCGGGGTACAAGCTTTTTTTTTTTTATTTTTTTACCAAAGCAAAGGTGGAAGACATTAACAGCAGGGGCAAAGCAAAATAACCCCAGAGCAGTCTTACAGAAGAACACATGTAACTTTCATTTTGTGGAACTAACGGCGAGATACGGCTAATCAGGGCACCGTGTGTGTCTCAGACTATGTGCTCAGCCAACGCCAAGATCTTGAGATGCTTCTTCTTCCTTAGCTCATGTGGAACCGGACCGAAGACGCGAGTGCCAATCAGCTCGCCCTTGTTGTTCACGATGACTATAGCATTGTCATCGAACTGGACCTCGCTGCCGTCACTGCGTCCTTTCTTCATAGCGGCACGGACAACCACCCCGTAGACCACATCACCTTTCTTGACCTTACCACGAGGCTGGGCTTCCTTCACAGAACCAATTATCATGTCCCCGAGCCTCGCTCCTTTCTTCCCCCCTCAAGGACTGTATGCACATGACCCGCTTGGCTCCAGAGTTGTCTACCACCTTGAGGTTGGTTCTCATCTGGATGAATGTCCTGATTTGCTGACTGATAGCATCACAGCGAGATGATCCCGTCACTGTTTCAATGGACGAGTTAACACCCCCCC

>Isogroup_158 ACAGGCCACCTTTGGAACCTGAAGGGCTTCCTCATGTGAAGGGACCAGCAACGGAACTTTGTGATCCAATGGCACCAGCAACTGCACCTCTGAACCTGGAACTTTGTGATCCTGATGGATCAAAATGTAAGCTTTCGGGTGCAAAATAAACACCCAAAAACTTGAGTGAAAACAGACTGTAGTATCCAAGATTCTCATAGTCAGTTGTCAACTATGATCTCTGTAAGGAAAATTAAATACTAACATACCTATTTCGCACTCGCACGAAAGTTCAACTTACAACTGTGGTTTGGATCGGGTGAAATCTCCGGAGACAAAAGCACTAACGCGCTACATGGGAAGCCAAATAGTGATACAAGCTAGCACTTTCAGAAGCAGTGTGCTAACTAAATAGTAATTGTCAATCCTTTGTATGAAGTGATAGGAAAATAAAAAATATTAGTCAGTTGCAAAACAATTTTAACTCCTGCAGATACATGCCAAAATTTCGGAGACAGATTAGTAGATATTACAACTTTGCAAGAAGAGAAGGACGTCAACGGTCTGTATAGTGTGCCAAAGTATACTTCCTGAATAACTACCAAGCACCACTCATCCGGCCAGAGGAACAGAAACGGCACAAAATATGGTCTTACCACAGGTGCCTCTCTGCATGAAGGCTCAGGAGCAGCTTTGTTCAGGAGCCTCACCCAGGTGAGTGTTGTGTCGACGTAGCTGACGAGAGGGAAAAGCTGGAGGTAGAAGGTGATCTCACCATGCACCTCGCGTAGCTCGTCGGCGTGCGTCGCCCCCATGAAGCACCA

>Isogroup_**159** ACTGCTCTGAGATTAGAATCGCTTGAATGGCCTGGTAATAACAAGCCTCAGGTGTTGCTGGTTGATCAATCTGAAAGATTTGTATTTAAGCCCATGCTGTATGAGCTCTTATCAGGAGAGGTAGATGTTTGGGAAATAGCTCCATATTTTACGGAGTTACTGAAGAACACCAGTGTCCAATTTGTCAAGGATAGTGTAAAGCTTCTGCGCCCCTCTGATCACTTAAGAAGAGAGCCTGGAGGATCATGCACTGGTGGAGTTGTTCATCTGGAAAGTGGCACTGTTATTGAATATGATTGGCTCGTTCTAGCTTTAGGGGCTGAAGCTAAAATTGATACTGTTCCAGGATCTGCCGAATATGCACTTCCTTTCACGACTTTGGAACATGCTTTGAAAGTTGAAAGTGAATTGACAATGTTAGAAAGGAGTAGGTTTGGTAAGAATTCCCCAGCTATTCAGGTAGCTATTGTGGGACTTGGTTACTCTGGTGTTGAGCTAGCTGCTACTATCTCTGAGAGGTTAAAGAACACTGGGACTGTCAAAGCAATCAATTTTCAAACAACCGTATGTCCCAGTGCACCACCAGGAAATTGTGATGCTGCTCTGAAGGTTCTTGAGTCCCAGAATATTCAACTTTTCTTGGGATATTCAGTGAGCAGCATCAGAGAGGTTTATACATCTGATGATCCAGGTAGCATGGCTACAGATGCAAAAGAAGCTGGCAGTGATGACAAGAAACTACTTTTGGAACTTCAATCTGCTCAAAGAGGCCTCCAGAGTCAGGTTCTGGAGGCTGATCTCG

>Isogroup_160 TTGCACGATGACCCGCAGAGGAAGACGCTCATTCTGCGCCGCATGGGTGCAAGCCTCTAGGGAGAGTTTCTGGCAGTCCATCACACGGCAAAGTTCTTCCTTCTCTGATTCCGTGAGATGGGGATGCGCCTTCAGATAGATGTCAACAGCACGATAAAGTCCATCATCTATTGGCCGAGCATAATCTGGTATGGCAGCAGCCAAAGACAGAAACTTCGACAACTTTAAGTTGACATCTGGTGCAACTTCAGCTAGGTAGCCGTCAATTAACTTAGCAACCATAGTTATCGGCAGTAGAGATGGAGAAGCTAATAGTTGCCCGTCATCACCAAGACCAGGGGAAGCTCCACCAGTTTCTTGATCCATTGCCAAGAAGTGGTCAAGAATCCTATGGACGCAATCCACATCATAGAGTGTTTCATCAGATTCAGACATGTTTGTTATCAGAAGATCTTCCAGAGTGGCCTTGTCAAGCTGTAAACCTATCCGCCGCTCCAAGTTAGATACACACAAGGAGCTGGCTTTAAGAATGATCGCGGTGCGCAAAAGGCCAAACAATAGCCTGCAAGATGTAGCACCCCTTTGGAAAGGCAACAGTCTATCAATCTCCTCAAGAAGATACTTCTGCTCCTCTTCAGAAGGCATGGCTGTTAAAGTTGTTGCTGAAAGGCAGCTACTGACATCACCAATGCTTTTACGCCGATTTAGACCAGGTAAACGCCTCTTGGCATAATGGTTAAGGGATCCAACAATGATGTCCTGACTGATGCCTCGGTATTCCATGGCTGAGATTACCTTCTTG

>Isogroup_161 CACTGTGTATGCTCCGACCCCAGCAAAAACAAGGCCAAAGAAAATGACCCACATGAAGCTCCAACCAACTGAGGAGGCAGCCTGTTTGCTGATACAAGTGTCTTGCTCTCTCATGTATAACATATTGTTATCACCACAGCTGCATTCATAGCTCCCCCATGTATTTTTGCAGCTGCAGCCATCACACTTGCAGGCGGTTTTTGTTTTGCATTCATCAATATCTTCACAACTTTTTACACCATCGCCCGTGAAACCTACTGGGCATTTGCAACCTTTAGATTCTTCATGTAAGCAGGCAGATATTGTCTTTCCATCTTTAGTATCCTCCCAGCACCCTCCATTGTTGATCTGACACCTCCCAATACCGGAAGCTTCACAATTGGTATATCCATCACCAGCAAACTTGACACCTTTGACAATAGGGCACTCACAAACACGCCCACGGAAGGTATCCTTGCATGCGGTGACGTTATTAGCCTTGTCAAGCCAGCAACCTCCATTGTTGTCCAAGCACTCATTTGTTTGAACGTCTTCACTCAAACAAATAGCAGGCTCAGTTGTCTCCTCAAATCCTGAACAGATAGCTTTGAGCACAGCACCTTTGTCCAACTTACCTCGGTATTGCCTATTGTTGACAACAAGCGTTGGCAGTATCGTGACATCTCCACGTTTACCATGACCAATTTGGGCATCTTGTTCTGCCTTTAGAATTGGGTTTTCCTCATCAGCTTCAGGATCTCCAACACACTTGTCTACCTTATCAAGGTCCAACCCAAGTGACTTAATAACATCAGTTGCACA

>Isogroup_162 AAGCCCGTGTTGGCCACTTCCGACGACCTCGACAAGTTCCACTATCAGCTCCAGCTCCTATGTGAGCTGGATCATCCTGGGTTGGCAAAACTGATTGCAGCACATGCACGTCCTCCAAATTACCTGATGTTCTTTGAGTTCTTTGAGCCTCCAAACCTAGCAGACAAGATACATGTCGAGGAGTGGAGCCCTTCTATCCAGCAAGTGGTCAACATTGCCAGCTATCTAGCAAACACTCTCCAATACCTACAAGTCCTTGGGATAGTACGCCGAGAAGCGAACTCAAAAGAGATACTTGGAGATGTAGTATTTGGAGGTGGTAGTGGTAGTAGTGGTGGCAGCCTTGGAACTGGAAACAATGGCCTTGGTGCTGGAGCTGGTCTTGCCATTGGAGGCGGCAGTAGCGGTGTTCCTGTCCCCCAGCCAGTGCTGCATGTGCACCCCGATGGCGTCCAGGTACAAGCTGATAGTGAAAGTGATGATGAAGACCAACAAATATACAACCCTCTCAAGTTACCAATGGGTTGGGATGGAAAACCTATCCCCTATTGGCTGTATAAGCTTCATGGTCTTGGTCAGGAATTCAAGTGCGAGATATGTGGCAACCACAGTTACTGGGGGAGAAGGGCTTATGAGCGCCATTTCAAGGAGTGGCGTCACCAGCACGGGATGCGATGCCTTGGTATTCCTAACACTAAGAACTTCAATGAAATCACATCCATCGAGGAGGCGAAGGCGCTCTGGGAGAGAATACAATCAAAGCAAGGGCTGAACAAGTGGCGGCCAGACTTGGAAGAAGA

>Isogroup_163 GTGTTCTTGGAGTAGCTGTTGGGCTGCTCAGTGCCTACACTATTAAGAAACTTTGTTTTGCAAGACACTCAACTGACAGAGAAGTTGCTATCATGATGCTCATGGCATACCTTTCGTATATGCTCTCAATGCTGCTGGATCTCAGTGGCATTCTGACTGTGTTCTTCTGTGGAATAGTAATGTCGCATTACACTTGGCATAATGTGACAGAAAGCTCTAGGGTTACTACCAAGCATACTTTTGCAACCTTATCATTCATTGCTGAGCTGTTTCTTTTTCTCTATGTTGGGATGGATGCATTGGACATTGAGAAATGGAGATTAGCTAGTAGCAGCCCCAAAAAACCAATTGCTTTAAGCGCTGTTATATTGGGCTTGGTTATGGTGGGAAGAGCAGCATTCGTATTCCCTTTATCTTTTATATCCAACTTAAGTAAAAAGGAGGCCCGTCTGAAGATTTCCTTCAGACAACAGGTAATAATATGGTGGGCAGGTCTCATGAGAGGAGCAGTTTCAATTGCACTTGCTTATAACAAGTTTACAACATCTGGTCATACTGCAGTGCGAGTTAATGCTATCATGATCACAAGCACAGTCATTGTTGTTCTGTTCAGCACAATGGTTTTTGGCCTGCTTACGAAGCCTCTGATTAACCTCCTCATCCCACCAAGGCCTGCAAATGCAGCTGATATCTCAAGCCAGTCATTCCTTGACCCACTTCTGAGTAGTTTGTTGGGTTCCGACTTGGACATTGGCCAGTACCTCGGCCGCGACCACGCTAGTCGTGGTGTGGTCGGC

>Isogroup_164 TGATAAAGAACAGCGCCTAAGCCATCAACTATGGTTTTGAAGTTTAGCTCACTCACTGTTGAGTCCAAAGCATCATCAAAGAAATTCTTGAGGGCAGGCACGATTGGGGAAACATCCACTTCAGGTTTCAGAAAATCAAGCGCATAATAATCACGAGCCATCGCTTCATAGTCCCGATTAACCATGTGAACAACATGTCCTATGATGGCTACTCTTGCATCCTCTGGGGTTTCACTCATCATGCCGAAATCAAGAAAAGCAAGCTTCCCTTCAGGTGTTGCCAAAATATTACCAGAATGCGGGTCAGCATGAAAGTATCCATACTCTAGGAGCTGCCTTAAGCTACACTGGATACCAATGTTTACCAAGTCCAGAACCTTCAAACCTTGGCCTTCTATGGCAGCTTGTTGATTTAGTTTTACACCCTCAATCCATTCCATTGTCAAGACCTTTGCACTTGTATAGTCCCAAAATATATCAGGCACCAATATGTCTTGCTTGTCAGCATATAACTTTTTAAACCTTCGTGCATTTTGACCTTCCTGGACATAATTAAGCTCTTGGAAAACTCTACAAGCAAATTCATCTATCAAAGCAACAGCATCAGTGGTTACAATGTCAACATACTTATTTATTAGAAAACCAAGTCCTCTCAGTAGGTAAAAATCACGCCCTATGGCATCCTCGATACCAGGTCTTTGAACCTTGACAGCTACCAGTTGCCCAGAGTTTTTCAACCGTGCCTTATAAACTTGACCTAAACTTGCTGCAGCAATTGGCGAAGGTGACATTGCTGA

>Isogroup_165 CATACTGGACTAGACAACTCATGACATTCGTGTAAACATAGACATTGTTAGATAATGTTACAGGTGCAACTCAAGGTGCAGGGCAGATTTTGAAGCGAAGAGGATATGCATGGAATCAAAGTGAGCAAATAGAATCTGAGAAGAAATACCTCTGACTTGATGAACTGGGCATCCTTCCTTTCACGATCAAACAGATACACATTTGTAGTCAATCTCATCCCTGGCGATAACTTTACCATAAACCTCAACCGGGTATGTGCAATTAGGGCCAACTTTGACCACCCTAATGGAAACGACTTCAGTGGAATAATCCGCCAGCTCATAATGTGTCCCATTTGCTGCAACATATTTAGTCAACCTCGTCCCTGAGGGTAAAAGGGTGGGGTTGATACCGTGGCCCAAGTTCAATGGTTGACTTCTCGTCAAGGTCGAATCCAGCTAGGTGGATGCTGCAGAAGCGGCTAGGGACACGACACTTCCTCTTGGGATCATAGACCATGAGGGCCTCTAGTTTTTCCTTGTGCAAATCATCCAGTGCACAGGCATCCATGCCCTCCACCAGTTCCTTCAAATCGGTGGGCACATCCTCTACAAACTCCTGAAATTCGATGGGCAGATCCTCCACAAATTCCTGAAAATCGATGGGCAGGTCCCTACGTTGAATCTTCGGAGGGCGCTGCATCTCGCCGGCCGAAAAGCAAGCGCTATTTTTAGTCGTGAGGTGCCTAATGATAGCCCTAGCGGCGATATCCGTACCTCGGCCGCGACCACGCTAGTCGTGGTGTGGTCGGCGTCTC

>Isogroup_**166** CGGCGAGGTACATATGAATCCTTACTCTTGGAACAAAGTTGCTAATATCTTGTTCCTCGATTCACCGGTTGGTGTTGGTTATTCATATTCAAACACCTCTAGCGATATTTTAAGCAATGGGGATGAGAGAACTGCCAAGGATTCATTGGTGTTTCTAACAAAGTGGCTTGAACGATTTCCTCAATACAAAGAGCGTGAATTTTATTTAACCGGAGAGAGTTATGCTGGTCACTATGTTCCTCAGTTGGCTCAAGCCATAAAGAGGCACCATGAGGCCACTGGAGACAAATCAATCAATCTTAAGGGTTATATGGTAGGAAATGCCCTGACTGATGATTTCCATGACCACTATGGAATATTTCAATATATGTGGACCACTGGCTTGATTTCTGATCAAACATATAAGCTACTGAACATTTTCTGTGATTTCGAGTCCTTTGTGCATTCATCTCCACAGTGTGATAAGATTCTTGACATTGCTAGCACTGAAGCTGGGAACATTGATTCATATAGCATCTTCACACCTACTTGTCATTCATCTTTTGCCTCGTCAAAAAACAAAGTGGTGAAAAGGCTTCGCTCTGATGGAAAAATGGGAGAGCAGTATGATCCATGCACTGAAAAGCATTCAATTGTATACTTCAATCTGGCTGAGGTGCAGAAGGCACTTCACGTTAATCCGGTCATTGGCAAATCCAAATGGGAGACCTGCAGTGAAGTTGTTAACACTCATTGGAGGGACTGTGAAAGATCTGTACCTCGGCCGCGACCACGCTAGTCGTGGTGTGGTCGGCG

>Isogroup_167 ATGCCCACGCATGCATACAACAAAAAAGGGCTCACACAAAGAAATGATACCCCAAAACGAAAAGAACATATGATCACAAGAAAACTATGGCGATTACAGTGAGACTCGGAAGTGTTTGAAGACGCTGCTTTGGATTATTATCTGGTCAAAACTGATGGATGTCTTGTGGTATCAACAGTGCACCGCAATCATCTGCCATTACCTTGTATTGCTGACCATGATAGGTATATTCGACAAGCAGTAGCTTAGGATCTCCAGGGCAAGGATCATAGAAGCCCATTATTCCAGACTTCTTTATCCCCTCGTGAAGCTTGAGCTGACCTGCCTCAGTCACGAGGAAGTTCAGTGGGATCGTCACATCCAATACTTGTGAAGCAACATTATCATCTATGTCACTCAATTCACTACTCTCTTTGATCTTTTTACGATCTCCATACAGAGCTTTCGTGATTACCAAACCATCATTCTCCAGCTGTTTATTTTTCTTGCGATTGGAGACAGGTTCCAGCAATCTTTGTGCTTTTTTAGCTGCTTGCCTAGCCTCTGTTAACTGTGTAGACAAGCTATCCATTTTTTCAAGTGTCTTCTGTTTTTCTCGCTTTAGATAGTAAGGCTTCACAACATATGTCTGAAGTAGAAAATATAGTGTTGAAGGAATAGCAAATGCGCCAGTGACAAATAATGCATTGAAGTCAGTTGAAAGCAAGACTGGAATAACTAACTTTTGCCCAGCACGATTTAACTCAAATTTCCAAGAGACACCCTGGATTCCTATATTATACAGCATCCTTACT

>Isogroup_168 CATCGAACTGATGCAACGGGAGGAGCAAGATGAAGACAAGGAAAAAGCCAAGTCCAAGTCCAAAGAGAAAGAGAAAAAGAAGGCAGAAGACAAGGTAACATTATCCGGGCTGCTCAATTTTGTTGATGGCCTGTGGTCCATAAGCGGAGAGGAAAGGATCATCATCTTCACCACCAATTACAAGGAGCGTCTTGACCCAGCACTTTTGCGGCCTGGCAGGATGGACATGCACATCCACATGGGATACTGCACCCAAGAGGCTTTCCGGATCCTTGTCAATAACTACCACGCCATCGACTATCATGCAATGTATCCAGAGATTGAGGAGCTGATCAAAGAGGTGACTGTGACGCCTGCAGAGGTTGCCGAGGTTCTTATGAGGAACGATGACACTGATGTCGCGCTGGATGATCTTGTTGAGCTCCTAAACTCAAAGAAGAAAGAAGCCAATGAGATCAAGACTGAAGACAAGCAGGTGGACGAGAAGAAAGATACCAATGAGATCAAGACTAAAAGTATGCAGGTGGATGAAAAGAAAGATGGCGATGGGATCAAGACTGAAAGTGTGCAGGGAGAGGAGAAAAAAGAATGACAAAGAGGTTGTCTTGAAGAATGATTCCTTCACAGAAGACAGAAGCAGTTAGGAGTGCGGAAGCTAACAGTGACCAGAGTGTGTGTGCAATGTAGATAGTTCCTACAGGATGTCCATTGCAAAATAATGTGATCATTTTGGGTAGATCCATTCGGAAATATGTGGTCATTTTGTACTACTAAATAACATCATGTTTTTTGGAGC

>Isogroup_169 TCCCATGTCATGCACATCAGCTCAACGGTCAGTGGAGAGTTAGATGATGATCTCCAAAGCTGGGATGCGTTGCGAGCAGCTTTGCCTGTAGGAACAGTCAGTGGAGCACCAAAGGTGAAAGCCATGGAGCTGATAGATCAGTTGGAGGTGACAAGGCGAGGACCATACAGTGGCGGGTTAGGAGGGATATCATTTAACGGCGACATGATGATCGCTCTTGCTCTCCGCACCATTGTGTTCTCAACAGCTCCAAGCCACAACACGATGTTCTCATACAAAAACTCAGATAGGCGCCGGGAGTGGGTCGCTCACCTGCAGGCTGGTGCGGGCATCGTCGCTGATAGTATCCCAGACGATGAGCAAAAAGAATGCGAGAACAAGGCGGCTGCTCTAGCTCGGGCTATTGATCTTGCCGAGTCAGCTTTTGTAGACAAGGAATAGAGTGTATTTTCGTGAATTTATTGTTAATTCTTGTCCATGGTATCTTCACCCAGTTCATGTGGGGAAGAAAAATGTCGTCAGTCGGTGGAGGGAAGCAAATAAGCTTATGTTGTCCTGTGAATTGAAAAAAGTCTAGAACTGTAATAAGATGTTTTTGTTTATCGGGAAGGTCCCCAATAGATTTTCCTCACGAAGAGTTGGAGCATGAGAAAGCTTAAGCAACGACAGCAGGTCCTTTGGCTGATTGTTTCGATAAGCCGTGGCTTGTAACTGTGCATTTCACTATTGTCTTTTTTTTTCTTTGGGGAGTTATTTTTCCAGGGAGTCTTGTCAGATTTG

>Isogroup_170 ATCCTAATGCCGGGTGGACTGCTGGACACAACCCTTACTTTGAGAACTATACTATTGAGCAGTTTAAGCATATCCTGGGGGTGAAACCCACACCTCCGGCTTTACTAGCTGGTGTTCCAACCAAAACATATTCAAGGTCAATTGATCTCCCTAAGGAGTTCGACGCTAGATCTCAATGGTCTGGTTGCAGCACAATCGGGACCATACTTGATCAAGGTCACTGTGGTTCTTGTTGGGCCTTTGGTGCTGTGGAATGTCTCCAGGATCGTTTCTGCATTCATCTCAACATAAGCATCTCACTTTCAGTCAATGACCTAGTGGCTTGCTGTGGTTTTCTGTGTGGTCAAGGCTGTAATGGAGGATATCCTATCAGCGCATGGCGCTACTTTCGTCGGAAAGGTGTTGTGACTGACGAGTGTGATCCATATTTTGATCAAGATGGTTGCCAACATCCCGGATGCGAACCTGCTTATCATACACCAAAATGTCAAAAGAAATGCAAGGTGCAGAACCAAGTGTGGAACCAAGAGAAGCATTTCAGTGTTAATGCATACAGAGTAAAATCTAGGCCACATGATATCATGGCAGAGGTCTACACAAATGGCCCTGTAGAAGTTGCTTTCACAGTTTACGAGGACTTTGCACACTACAAATCAGGAGTATACAAGCACATCACAGGTGGCGTGATGGGAGGCCATGCCGTCAAATTGATTGGATGGGGAACCAGTGATGCTGGCGAGGATTACTGGCTTCTTGACAAATCAGTGGAACAGAGGCTGGGGTGATGACGG

>Isogroup_171 GCCCGGGCAGGTACCTGGTGATGTCGTCTAAGGCTAGTTTACCAGATGTGCCAGCATCACCATCAGCTGCAGGCAGGTTCCCTGAATACATCTGTCTATAGATGTGGTGCCCAATGTGGTTTGCTATCTCACTCTTGGACATGGATCTCCGGAGCCCTGGCAACTTAAAACCGTTCCAGCTAAGGATGCTTGTTTGTGGCTCGCACATACCAGAAGCGCTGTCTTGTTTAACCACTTCAGTTCCAGTAACTGAATTCGAGAAATGTTGAGGCAAGCCATCTGACGTGCCAGCTTCCTGTCTTCCTTCAGCATCTATCCTACTGGATGCAGAATGGTGCGATAACAGTTCTCGAAATCTTTGGGGTGCTAACTGATCATCATCAAGTTCATCACGGTCAAAATCTTGGCACTTTACATCATCTGGATCTTCAAATATAGAACACCTTGATTCAAAGTATGGATTCTCCAAAGTGATGTCATTTTTCTGGCTTAGCGACAGTAGACGCGGGTCACAGTGGATAAGCTTCTCCACGTGTCTATTCATCATTCCTGGGGCACACTGAAGAAAGTGCCTCCTGTGCATGCTTGCCTGTCCAGAGGTAAAATCGGAGGTTGCCTGCCACAAAGTGTGCTTCCTTGGCTGTGGGTTAGTTTCTCTGAAGAAAAGTGGTTGCCTGGACAATGCAATCTCCAAGGTTCCAGGTTCAGTTTCTGGACAAGCCATCTTAAGTGCACAGATATCAGACCACTGTATCTCAATTTTGCTCTTGAGACCACCATCCAGTACCTGG

>Isogroup_172 CTAATACGACTCACTATAGGGGCAGCGTGGTCGCGGCCGAGGTATGCACTACGGTTCTTGGCATCTGTCGTGTCACAAAGCTTCATAGCTTTTGGTGATATTCTCGAGTTGCATAAGAAATTCCTTGAGCTTTCTGGTGGTATTAATAGGGTCTTTGAACTTGAGGAGCTTCTAAAGGCATCTCAAAGCAATGCTGTCATGCCTTCAAATGTTCTTAGTGTGGCATCGGAAGAAATTATTTCCTTCCGTGATGTCGATATTGTGACCCCATCACAAAAGCTATTGGCTAGCCAATTGTCTTGTGACGTGTCTCAAGGAAAAAGCCTTCTTGTCACTGGTCCAAACGGCAGTGGAAAGAGTTCCATTTTTAGGGTGCTCCAAGGTTTGTGGCCTGTAGCTTCTGGAAGACTTACCGTGCCGTCTGAAGGGATATTCCATGTTCCTCAGCGTCCATATACTTGTCTTGGAACCTTGAGGGATCAGATCATATATCCTCTCTCACGTAAGGAGGCAGAGGCAAAGATGGTTACATTGTCAAAAGCAAGTGATAGATCTACTGCTTCTGGGTCGCTGGATGGCCACCTGAGGACAATTCTAGAGAATGTTCGCTTGGTCTATCTTCTCGAGAGAGAAGGTTGGGATGCTACTCCCAACTGGGAAGATATTTTGTCCTTGGGAGAGCAGCAGAGGCTTGGCATGGCTCGTTTGTTCTTCCACTCTCCTAAATTTGGTATCCTTGACGAGTGCACCAATGCCACAAGTGTCGATGTGGAGGAGCATCTGTACCTG

>Isogroup_173 CGAGTACGAGTTTCCCTTTCACGCTTTCTTGAAGATATCAACTGATTGATCCTCTCATCCCTCTCTTTCCTTAGGCTACTAAACTCAGATTCACGACGATGCACAATTCTTTCCTGGAAAGCGTTCTTATGCTCCAATAGTCGAGAAAGCCTATTTTTCTCCTGCAAGTCACCCGCATGGTGTTGCTTGCTAAGCTCAATCTCTCTCAGCTGCTCTTGCTCATGAAGGATCTTCTCTTCCTCGAGGCGTTTCTGGAAGGCTTCTTCTATCAGTGGTGCTTCCTCCTGCCGTTTTTGCCCTCTCCAAGTGATCCATTTTTTTTCCAAGGGTCTGCAGCTTTCTCTCCATCTCATCACGCTCCTTCTTCTTTAGAAGCATTAACCGCTCAAGGGCACCTTGTTTTGTTATATCCCCCTCAATGACATGTTTCTTCCCTTTCTTTGTATGATGTGTTTTTAGATTATCGCTGGTCGACAACAACAATTGTGCTTCCTTCTGATCCTTTTGTTCTATTTCTCGTTGTATCCTCTGCTTCTCCCTTAGTCTCTGCTCCTTCGAAAGTCTCTCCCTTTCTTCCTCAGCAGATTTCTTCTGAATACTCAATCTCTTTGTCTCCTCTTCTTTCTCCTTCTCCAATATTTGGCGCTCATGGTCTTCTTTGCGTTTTTCAATAATTGACTTCCTTGCAAGTAGCTTTTTATGTTCCTTCTCAACCACTCCAGTAAGACTAATCAAGTTCTCACCAAGTTTGACCTCGGCCGCGACCACGCTAGTCGTGGTGTGGTCGGCG

>Isogroup_174 CGAGATTTTTATGTTCTACTTTCTAGGCATAAGATCTCACTGGCCATAAAAACTCAATTCGTTTGGTTTTTGCCCGATGCATTTAACATGCAAACATTTTTGGGAACATAAGAAGAGTCTTGCGTGTAGGCTGCACAAAAGGGAGCTGTCGGCATAAATGTTTACTCTTCTTGGAGTTATCCTTTGACAAACTCTAATGTGGATATAGAAGCAGCTAAAAGATACCTAGACTTCGTGTTCGGGTGGATTCTAGAGCCCTTTGCATCCGGAGATTATCCAGAAGTGATGAGGACAAATGCTGGGACCCGCCTTCCATCTTTCACAAAGTCCCAATCTCAACTTGTCAAGGGTGCTATTGATTTCGTTGGGATAAATCACTACTATTCTATGTATGTTAATGATCGTCCTCTAGACAAAGGCACACGCGACTATTCAGCAGACATGTCTGTTTACCAGAGAGCTTCTAGAGCAAAACCACCAAGTGGCAAGCAAGTCCCATCATCTTCTTCTCGAAGTGACCCGGAAGGATTGCAGCTTGTGCTGCAATACCTATCAGAATCCTACAGCGACCTTCCAATTTATATCCAGGAGAACGGTAATACTTCTAATGACACTCTCGACGACACCGACAGGGTCGAATACTTGAAGACCTACATGGCTAGCACATTGAAGGCACTAAGGAATGGAGCTAACGTAAGAGGGTATTTCGTATGGGCCTTCCTAGACGTCTTCGAGTTCTTTGGAGGGTACCTCGGCCGCGACCACGCTAGTCGTGGTGTGGTCGGCGT

>Isogroup_175 GATGGAAATTTGGACATTCTTGTTGGAACTTCTTATGGCTTTTTTTTATGTCATTGATCATCGTGGCAAGATTAGGAAAAATTTCCCACTTGAGATGGCTGAGATCCATGCACCAGTCATTGCAGCAGACATCAACGATGATGGGAAGATCGAAATGGTCACTGCTGATGTGCATGGCAATGTAGCAGCTTGGACTGCAGAGGAAGAAGAAATCTGGGAGGTGCATCTTAAGAGCCTTGTTCCACAGCGACCTACTGTCGGGGACGTCAATGGAGATGGCCACACTGATGTTGTGGTTCCAACTGTATCAGGAAACATTTATGTTCTTAGTGGAAAGGATGGCTCAAAAGTTCAGCCTTTCCCATATAGAGCACATGGAAGGATCATGAGTCCCGTTCTGTTACTTGACATGAGCAAACGTGGGGAAAATCCGCAAGGACTAACCCTTGCTACTACGTCCTTTGATGGTTATCTGTATTTGATCGAGGGCTCCAGTGGCTGTGCAGATGTTGTTGACATTGGAGAGACCTCTTATACTATGGTTTTGGCGGACAATGTTGATGGCGGAGATGACCTTGATCTTATTGTTACTACCATGAACGGCAATGTCTTTTGCTTTTCCACTCAATCACCACATCATCCGCTCAAGGAATGGAGGTCATCAAACCAAGGAAGGAATAATGCTGCATATAGATACAACCGGCAGGGTATATATGTTAAGCATGGTTCAAGGACATTCCGTGACGAAGAGGGCAGACATTTCTGGGTAGAGTTTGAGATTGTAGACAAG

>Isogroup_176 TCACTCATGCTGGGAGAAAGCAGGCAGAAGAATTGGGAAGGTTTTTCAGGAACAACATTTATCCAGGGGAAGGAACAGGTTTGCTTCGCCTTCATAGCACATACAGACACGATTTGAAAATTTACAGTTCAGATGAAGGACGTGTTCAGATGTCTGCTGCGGCATTTGCAAAAGGTCTTCTTGATTTGGAAGGACAACTAACTCCCATACTGGTCTCCCTTGTAAGTAAAGACTCATCTATGCTAGATGGACTGGAAGATGCTAGCATTGAGATGGATGAAGCAAAGGCCAGATTGCATGAAATCATCATTTCAAATGCTAAAGCGACGAATACCAATGGATCGGTGGAGCATCCGTGGATGGTTGATGGTGCAGGTTTGCCTGCAAATGCTTCTCAACTTCTCCCGAAGATGGCCAAGTTGACAAAAGAAGTTACTGCACAAGTCAAGCTGCTTGCTGAAGGTGAAGATGAGAAGCTTGCGCTGACAAGCTCATTTTCTAAATACGACCAAGCAAAGGCTCTTGGGAAAACAACGATTGATGTTCCTCGTATTGCTGCTGGGTTACCTTGTGGCAGTGAAAGCTTCCTCTTGATGTTTGCTCGATGGAAAAAACATGAGAGAGATTTCTATAATGAAAGAAAGGACCGATTTGACATTACACAAATTCCAGATGTCTACGACTCGTGCAAATATGATCTGGTGCATAATGCTCATCTCAATCTTGAGGGTTTGGAAGAGCTTTACAAAGTTGCTCAACTTCTTGCTGATGGTGTTATTCCAAATGAG

>Isogroup_177 CAAGTTGGCTCCGCATGCGACTCCATATATCCGGATGTTTCTCCAATAAAGTAAAGATTAAGCTTGTTACGATGATACTGCGCTGGATCATCATACCGGAGGGCTCACTGAAGGTCTGATCCTTGCAGGCATGCTAAGGACATCATCGCTGTCTGTGGCCACTGGATGAAAGAAAGAGCGATATGGAGTTATAGACATCTTGTTCATCGAGTAATCCAGGTCTTCATCATTGTTGATATCTATCTCAACGTGATCATCCAAGCTGCCCACATTCATTCCAGGGGCATCGAGGTCCTCAAGCTCTTCGTCATCGTCGTCTTCATTATCGTCGCTGTATGTTGTCCTGACCATGGACCAGAACTCAAAATTTGGACGCATGTATCTGTCAGATTCTTTTAGCAGATAAGGATCGAATGGGAAGAACATGTCAAGTCTACCAATGGCTCCAAAAGTCCTGGACAAATCAGATTCGATTGCATCATCCAATGCTGAATCCATATTCGCGGTGAACAAACTGGCATCTTTAGCCTGTCTTAGGAACTCGTTTACTATTGAAGGCAGGCACACCTTTAGAGGTTCCAGTGGATGGGTCAAAAGAAATCCAAAAGGCATGTGAAAAAGCTGTGACTTAAGCTTTGGGTAATCCATAATAGATCTTAAGCGAAAGCAAAGGACATACATCACAGCCTGACAGGTCGCATAAAATATTTGATGGTTTGAATTAGCTGCCATCCCTATGATATTCTGAAGGTCACAATACTCAACGCACCAATCCACCAATCTTTGAA

>Isogroup_178 CAATGCTAGCCTTGATGCTTTCAAGGCTGTGCCCACGCTCTGCCATGTCCCTCTGAATTTTCCATGCAAATTTAACCTCATTGCTGATGTCTAAGTAGATGCTGAAGTCAAGAAGGTCTCTCACACGTGCATCGTACATTGGGTGCAAACCCTCGATGACGAAGATGTTAGGGGGCTGGATGGTCTCCGGCGGGTCCAACAGGCCGGTGACATGGTTGTAGATTGGCTTCTCAATAGCCTTGCCTTCTTTGATTGCCTTCACCTGCTCATACATGAGATCAAAGTCGTTTGCCTTGGGGTCCAGGGCGGTCACACCTTTCTCCTTCCTCCCGGTTCTGTCCAAGGAATGGTAGTCATCGAGGCATATCACCGTTGTGGTGTCGCTGATGAGCGTGTTTGAGTCTGGGTTCCCGCCCCTGGGTGGCTCTGCAGCACCTCCAAACACGCTGGTGAGCCGGCGCATGAAGGTGGATTTCCCACATCCTGAGTCTGCTGCCAGGCCAATCACTATTGGCTTGTCAACTGCGCATGAAACCTGGAAGGTCCTTGCTCCATTCCTCGTGTTACTCCTCCTGCTGCTTCTGGTTGTGAAGAGGATGACTTGGTTCTGCCTGAAGCCTGAGTTTGGGATGGTGGTGCGTGGGGAGTGCAGGGATGTGGTGGTGTGTGGGCTGCAGAATGCCATGTTTGCTACCCAACCTTCGCTCCTTGCTGGAGCTGCTCTTCTTCCCTTCAAGCCTTGACCTCGGCCGCGACCACGCTAGTCGTGGTGTGGTCGGCGTCTCTC

>Isogroup_179 AGCCATATGCACAGTAGCAGAGGGAGATCAAAAGATGAACACGACGCTGTCATGGACCCTGTTCTTCTGGTTCTGGGCGACACATTCGGAGATCCACATCAAGTTTCTTATCTCTTGCTCTCGTAACCTCATGTATGCAAAGAAAACGGCATAGTGGAACTGCTGTTCAAATGACAGGCACAACCTCTTCACTTCCTCCTCATAAAAGGCCTTATCCAACATCTGGCTTTCACCATACGAAATCTTGGCAAAAATAGACTGGTATGGAGGATACTTCTCCATTACACCACGCACCTGGTCAACATCTTCACAGACAGCCAACTCTTCATGACCATATGGAAACAATAAACCGAAGTTGGAATATAGCTTCCGGCGGTCATCTCTAGTCAACTCAGTCCCAATACTGTTTATTGTAATGTTAACAGCTCTTCTGTCAGCTTCAAATGAAAGGAGATTACACATGATCTCAGCTGTTGCACCACCTAGTTTCGCGCAAAATTTGTAAAAATCCTCAAGATAAGCTTTGTAGAGTGTGTTCCTCATGATTTCAATATTCATGTCATCCAAATCCTCAGATGTAATGCACTCTGAGAAATATGGTGCTAAGGGTGTATCAACTAGAACCAATCTGTAAAGCTCACGCATATTTTGAGCAACTGCAAGTGATGCAATGCTATCAAACATGCCCAATGGATGGCATTTCTCCAACAGTTCGTTAACATCTCTTTCATGCAATGTCCCAGTAACAATAAGGACAACATTATCAATCATGTGCCCATAAGTTAT

>Isogroup_180 TCGTCTTTGCATTGTATATATGTGTATGTGCAGCCAGTTGTACAATCGACATATGCATGATCCTCCACCTACGTTGATAGGTACTTGCATAATTGTATGGAATCATGAGTAAAACCCTGAATGCAGAGTCGTCAACAAAAAATGAAATGGCATGCGTATGCCATGAGTAAAACCTTGGTATAATTGTATATATGTATATGCTGTTATTCCCTGCTCGGGCGCAGCTTATGCCTTTCCGGACTCCTCGCAACCCGGTGGCTTGAAGGCGATGAAGCTGACGCACTGCACTTGACGGATGTTGTCGAAGCCGATGATGCGGACATAGGCGTCAGGGTACTCCTTCTTCACCTCCTCCACTTCCTTGATGACCTGTGAGGCGTCGGTGCAGCCGAACATGGGCAGCTTCCACATTGTCCAGTACCTGCCGTCGTAGTATCCGGGAGTGCTGCCGTACTCGCGGAAGATGAAGCCAACCTTGCTGAACTCGAGGCAAGGAACCCACTTGGAGCGGATCAAGAAGTCGATCTGCTTCAGGAGGGCCTCCTCTGAGAGTGGTGGCAAGTAAGACAGGGTCTCGAACTTCTTGATGCCCTCAATCGGCCACACCTGCATGCATCTGATCCTTCCACCGTTGCTGACGTTGCCGAGGCTGGAGGCGCTGCGGCGGCTGACAGGGAGTCCTGCGGTGGACTTGAGTCCCTGGAAAGGCGCGACGGAGGTGGCCGACGAGGCCATCACGGCGGGGGCCATGGTTGTAAGTATATTGGTAAGTGGAGGATGAGATA

>Isogroup_181 CCGTGTTACATGGGATTATTACTTTCTGGGCCGTGAGCATACCCTTGAAATCAAAGAATGGGAAAGCAAGGCCGAGTATGAATATGTGAAACGTAATGGAGTGTCGATCTTCCTCATGCCGTCAGGAACAATCGGGACACTCAGAGCACTTTGGGATGTTTTCCCTCTTTTCACAAATACTGGATGGGGTGAGAATGCAAATCTTGCATTTCTCAAGAAGCATATGGGTGCGACCTTTGAGGAACGGCCAAAACCATGGGTTTCAGAATTGAATGTGGATGACATCCACTCTGGAGATTTTTTGGTTTTGTCGAAGATTCGAGGACGCTGGGGTGGGTTCGAAACACTGGAGAAGTGGGTTACTGGAGCTTATGCAGGCCATACTGCAGTTTGTCTGAGGGACTCTGAGGGGAAGCTGTGGGTTGGGGAATCTGGTCATGAAAATGAGCAGGGAGAAGATATTATTGCTATCTTACCATGGGATGAATGGTGGGACTTTGAAGTGACAACGGATGATTCAAATCCTCAGATTGCATTGCTTCCATTACGTCCAGATTTGCGCGCAAAATTTAACGAGACTGCAGCTTGGAATTATGCAAAACAAATGAATGGGAAGCCTTATGGGTATCACAATATGATTTTCAGTTGGATTGATACTATAAGTAATAACTACCCCCCGCCATTGGATGCTCATGTGGTTGCATCTGTTATGACTGTGTGGAACAAGCTTCAACCAGAATATGCCGCCAATATGTGGAAAGAAGCTCTTAATAAACGACTGGG

>Isogroup_**182** CAGCTTACACAAACTCCAGCGGATGCCACAAGGTCGCTACCGTGGCTCACCCCTTGGGTCTAGTTGTTGGAAGACGTTCACGCTGGGGATCTTTTGCAGCAAAGTTGGATTTAGAGAATGGGCCTGCACCCTTGAATCCGACATCACCTTCACTAGGGCAGAGTAGAGAGCAGCTGACTTCTGACGAGTTGAAATCTCTTCTGGCTGATACAGAACGAAGTAAGCTTTTGAGGAAGCTAAGTGAAGCAAATCAACATAACCGGTTTCTCAAGAGACAGTCACAAATAAAGGATGATGCAGTTGTGAAGTTCAGAAGTGAACTTGCTGTTTTGGAACTTGAACTGCAGACATTGGTTGGCCTAGCTGAAGAGATTGCTAATTTTGATGTTCCATCAGGGTCAAGGAAGGTAAATGGAAAATATATTCAGTCTCATCTTCTCTCCAGATTAGAAGCTGTTCATGAAAAGGTTATGTTACAGATAAAGGATGTTGAGTCTTTAAGACCTCGAGAAACCACTGTATACTGGGTTGGCATGGCTGAGAATGTGCAAATTATGGGCTCCTTCGATGGCTGGTCCCATGGTGAGGCAATGTCCAGGGAATATTCAGGAGACTACGCGAGATTCTCGGCGACTCTGAGGCTTAGACCTGGGAGCTATGAGATCAAGTTCTTGGCGGACGGGGAGTGGAAACTGTCATCGGAATACCCGGTTGCTGGCGAGGGATGGACACAGAACAATAAGCTTGTCGTGGAATAGCAGATTATTGTTGCCTTTTAGTAG

>Isogroup_183 CAGCCAAACTGCTACTACACCTTGTCTACTTGGTAACCTTCCCTCATAGCACCACAAACACAAGAAAATGAAACACTAGCACTAATCAATTCCTCCCTCACTTCATAATGGTAACAGAAACATATGATATAAGCAAAAGACGAAATGATGTTAAAAGCCTTGATTTGTTTTTATTGTGCTCATCCACAACCACCCTTGGAACCAGCTCTGCCAACGCAAGTTGTAAAAATGAAGCCCATCTTGGTTAGTGACTCGTAACACCACGAAGAGTAGAAGTGATAGAAAAATCTAACACTTCTAACTTCATGTTACTATCTTTCTGGTTGATCAGAACTCGAAGCTCCATTAGGAGGTGTGGACAAGTTGCTCGTTGATCCATTCTCCACTGGTGGAGGATTTCCCCACTTTCTGTCAGATTCCAATGGCTCCATGACGTAAACAGTGCCATCGGTAAGGCCCAGAGCAAATTGATTTGCTTCTGAAGGATGTGCCGCAACCACAACAGGATGAACATTTGAACTGATATTTGGGGGAAGATAGGAAGCTGGAAGAATTCGGCATTGGAGTCTCAAACTTGATGCGCTAAATATACAGACAGTGGCATCCAGGAAGCTTGCATAAATCAGTTGACTATCACATGAGAATGTGGCATGTGTTATTGGAGCAGAATTTTCCCGAACAGGCCACTGCTTTACGGGTTCTAGTTTTGAAGCATCGTAGATGGCAATCTGGGTTTCATGTACCTCGGCCGCGACCACGCTAGTCGTGGTGTGGTCGGCGT

>Isogroup_184 TCGGTGGTCATTTTCTCACTGTCAGTCACAAAGTAAGGAGAGATATAGCCACGGTCAAACTGCATTCCCTCCACAACATAGAGATTGTTCTCGGAACTTCTTCCTTCTTCGAGGGTAACCACACCCTTCCGTCCGACCTTGCTCATAGCCTCTGCTATCATGTTACCGATTTCATAGTTGTTGCCAGCACTGACTGCAGCAACATCAGCAAGCTCACTATCTTCAACCTCCTTTGACATCTTCTTTAGTTCCACAACAAGTGCTTTCGCTGTTTTCTCGATACCACGAGTAATTTGTACCTGCCCGGGCGGCCGCTCGTAGCGTGGTCGCGGCCGAAGGTACCACCTTTAACATCAGTCAAGTGTTTTCGGAGTAACCTCCATGCAGTATTCATTCTGGTTGCTGATCAGGTGGTTCAATATCTTGATGTCAACTATAGCACCCAAGTTGTCTGAGTTTGCAACGAAGACATACTCCTTTCCCTGCGACAATAAGGTATCAAGTTTCCCACTGTTATTCAAAGAGGGGAAAACATCACCGTGGCCTGGGGGATACCAGCCATCCTTTCCTGTCTGCCCTTTGCTCGGAAGTGGCAAGAAGTCTTCAGTAACAATCCTGGGATATTGACTCTGGTTGAAAGTATGAATGCTGATGTTGGAGTTGGAGTACCTTGTCCCAGGAGGCATAGGCATCGAGGTATCTGACCTTGGAGAGTAGCACGTGCCAGTTCTGCACCGCGTGGCTCGGCTGCTTCTCCGTCGTGCTCGTTGCCGCTATCTT

>Isogroup_185 CGAGTTGAAGTCTGAATGACCAGGGAAGTTTGTATGCAAAACAAACTTCTTTATCTTGTGTGTCTGTCCATCAAATAAGATATCTATACCGCGCGAGAAGTAGTTGTAGAAGTAGTCGCCACAACGAGTAGTCCGAGGGCGGAGGTCCGATGCAGAGTGTATAACCATTTGGTCCACCTGCTTCTGGTGGATACCGCATGGACGACCCAACTCAGTCCAAATATCCTGTGGTGATGCTCCAAAGGGTATATGTTGGCCCCCGATAGTGAACCAAAGCTCTTCACCAAGCTTTGCATGCACTTCCTCCATATATAGACTGCCTGCAGGTAACGCAGGCACCAAAGCCTTGTCCATCAATGACCCAACCCCAACCTTACTATCTGTTGAGCTATCATATACAGATACCCGGCAAGTAACAGGGGTTGTCCCATCAGGAAACTCCAGGGGCAAATCTGCCACTTCACCATTGGTGAAGAAATTGGTATATTGGCTGGGTATTGGGAATGCAAATGACAAACCCGGATAGAAGAGGGTATAAATGCCCCTCTCTTTATCGTATATACCCGGGAAAGTTGGACCGAATAATGCATAAACAGACACAAAAGTTGCTAACGTCGATGGACCGCCAATCAAAGACGTTGCGTATCTCAATTGCAATCTCTTCACATCAAATATCTCTATAAGACGGAGCCTCTGCGACCAAGGATCGAAGCGTAGGTGGAACCCGTGATCCGGAAAGCTTATGACAAAGTCCAACTTCAGAGGCTCCTCGTCGAA

>Isogroup_186 TTGATTGAGTCACATTTCATTTGTGCATATAGGTTGGTTTCGATTCCTCTTCCCATGCCTTCTAGGACGACCAAGTCAGCATCGCTTGCCATATATGCTAGCTCTGGGGACACACTAGATAAATCTATTACAGGCAGATCGTTACCAGAGTTGGCAACAAGGAGGTTAGATACGTCCACACCTGCAAACTTTCCATTTTCGTCCTTGAGCTTGTTTACAATCTCAACCAGCTCTAGGTAAGTGACATCATTGATGGAAGGCATGTCATTGGCAGCAAGTATCACCGTTGTGCCACGTCGGAGCAATTCCCTCGCAAATGGTAGTATGCCCAATATGATATCAGCACCAGAATTATCAACAAAAATAACTGCCTTTTCCCAAGATTTCTTGGTCCATTTGGTTTTGAACGCGTCTAGGTCATCAATAACCCAAGGTCGAGATAAAAGATTCTGGCAACTTGCCAAGAAGGACATACCATCTTTAGCAAAAACTTCTGCAAGCTGTGCAGATCCTAAATCAAAAATATTTCCTGCTAATATTCCACGGATCAAGTTCTCTGCTCGTTTCTCACTGTCTTCTATTTCATCATTCCGCTGAACAACTCCCTCAAACAGTGACATAGCCTTAGCATTCTCCTCATCCTTAACCTTTTTTGAAGATATCGTTAAATCCCAATTCTCAAGGATTAGCTCACGTAGCCTACAAAGAAGTAAGCAGTCAGGAGGTCCTCCATGGCTCTCGGGGTTCTTCTTCAACTCCTCAAGAATAGAAGTG

>Isogroup_187 AGAATAATCTTTCCTTCAACCCACCAGACTCCTTCACAGCATTTGTAATTGCAGAATAAATTCGGTTATATAGCCGGGGGACACTCGCGAATACGGTTGGTCTCAAAGCAGCCAAATCATCCATCAGCTTCAAATTATCCCCTTGGTAAAATCCAATGGCAACACCATAGTGAAGCAGTGCAATTTGGTTGGCCCTCTCGTAGATGTGAGCTAAAGGTAGATACGAGATATACACGTCGGAAGGGTAAAACCTAATACCCAAGCTCGATCCTGCTACATTTGCAATTAAGTTCTCATGAGAAAGAACAGCTCCCTTTGGTGTCCCAGTAGTGCCACTAGTGTAGCAGATAGTGGCAACATCTTCAGGTCTTGGAGGACGGTAAGTTTGAGAACTCATCTTTCCCTGGCTCTGTAGCCTGGAGTAAGTTATAATTTGCACTGCAGTAGTTTCCGTTGAAGATGGTGTATTTGCATCGTCTCCACCAACTACCACTATAAGCCGAACACATGGCATTTGAGTTATGAAGCTCAGCAGAGCGCTTAGTGTTTGAGGAACACAGAAAATAGCTTCTACTGTTGCATGGTTCACAATGAACTGGACTGCATCTGGACCAAGAGTATCATAGAGTGGCACAGATACATATGAATATGCAGCACAAGCATGGTCAACTATGATCCACTCAGGTCTGTTTATAAAATACAGACCAATGCGTGCACCTTCAGGTATTCCATGATAAATAAGACCAGAACCTATTGCGGTCCTGCTAGTACCTG

>Isogroup_188 TCTTCAGGTTGTTTTAATGCCGACTATTGTTGGAGTCTTGGCGCATGAGTATTTTCCCAAGTTTACTGAGCGCATTATCTCCATAACACCGCTGATAGGGGTCATCCTGACCACTTTGCTTTGTGCTAGCCCTATTGGGCAAGTCGCAGAAGTGTTGAAAACGCAAGGTGCTCAACTTATTCTCCCTGTTGCCCTGCTGCATGTTGTTGCATTTGCTCTTGGGTATTGGCTCTCGAAATTGTCTTCTTTCGGTGAATCGACTTCAAGGACCATCTCAATTGAATGTGGGATGCAGAGTTCTGCACTTGGATTTTTACTTGCCCAAAAGCATTTCACAAACCCGCTAGTTGCTGTTCCTTCTGCTGTCAGTGTGGTGTGCATGGCGCTCGGAGGCAGTGCTCTTGCAGTTTTCTGGAGAAACAAAGGGCTTCCGGCCGATGACAAAGACGATTTCAAGGAATGAAACAAAAACAACTCTGAGCTCCAGCTTCTCTAGTCATTATTCTTGTATTAGTTGAGTGCAGTTACATCAGAGCATTTTTTTATGTTATTCTAGATAATACCATATTATTGGTAGGGGGTTTGTTAATGATTTAGTTATGGCTTTGCACTTGAAGTTTGGTGGGTATTGTATTTTTCATCTTCGAAATAAGCTGCTTATCGGCAAAGCAGAAATTATTTTATAGGCTGTTGTCAAGTAGCCCATCCACGTTACGTGGACAATCAGTGTATTTGATTCTCCGGCTGGCTTTTGTTGAATGAAAGCATTTTTT

>Isogroup_189 AGCGGCGCCCGGGCGAGGTACCCTGAGAACTTCTTTCTTCTAAGAGGCAACCATGAATGTGCATCAGTAAACCGCATCTATGGGTTTTATGATGAGTGCAAGCGCAGATTCAGTGTAAAACTCTGGAAAACATTTACAGACTGTTTTAACTGCTTACCAGTATCAGCATTGATAGATGAAAAGATTCTATGTATGCACGGGGGTCTTTCTCCAGAGTTGAACAAGCTGGATCAAATACTCAACCTCAATCGCCCCACAGATGTGCCTGATACCGGGTTACTTTGTGATCTTCTTTGGTCCGACCCTTCCGGCGAAGCTCAAGGGTGGGCTATGAATGATAGAGGTGTCTCATATACATTTGGGCCAGATAAAGTGGCTGAGTTTCTTGAGAAGCATGATTTAGACCTCATCTGCCGAGCCCATCAGGTTGTTGAGGATGGATATGAGTTTTTTGCTGATCGTCAGCTTGTAACAATATTTTCGGCGCCCAATTACTGTGGAGAATTCGATAATGCTGGTGCCATGATGAGTGTAGATGAGACACTGATGTGCTCCTTCCAAATACTCAAACCTGCAAGGAAAATGTTGGGTGGTTCAACTAATACCAAATCTGGCTTCAAGTCATTGAGAGGATGGTGATGGTGATGGTGATGGTGGCGGTGGCGTGGTGATTGGTGAGTGAACTGTGAAATGATCTGAAGGGCACATACTCGAGCGGTTGCGACTAACTGGCATTTTGGCCTGCACCTCGGTCCCGGAACAAGAATGCAGATGG

>Isogroup_**190** CTCACGTGTTTTTTCGTCAGGGCAACTTTTCACAGCTTCTATAAACCTAGCAAGGATGACAGACTCAATGTGTGATTCTGCGAGTGTCAGCAGATGGTTCAAACATCTGTTCCACGCACCAAAACCTCCAAGTGTCTTCATATGCTTCTGGAGTCGAGCAGCAACACTATGCAGTAATCGTGATGTTCGGTATCTGAATGCATCCAGCTGAAAGTTAGGATCCCTTAGATGATCTTCCCCTTCCCATCGGGCAGTAACGGGATTTGGCTGAGACAAGTAGGTGCTCATGGAATCTCTCAAGTAGTTCCAGGTGACTGAGAGTGTTCCTCCCTTAAATTTTTGCTGATATTGCTTCAGGAGATCTCCAGCAACCTGCTGCAGAAGAACTGTATTGTCTCCTTCAAATGTTTGAAATATATCATGGTCATTCCGCAGACCGCCAAAACGATTTACAGCAGCATACCCATGGCCTCCACACGATTCTCGGCATATGCTAATAGCTTTTGCTGTGTATGAAGTTATGTAGGATTTCAACCCAGATGAAAGCACATGGACATCAGCGCTAAGATCCTCATCATCGGTTTTCTTCATCTCTGAATACTTATCTACCAAATACGCTCTGGCAAAGTGAAATGCATACGCTGATGCCAACATAGGCATTAGTTTGTGCTGGTGAGATTGGTAATCCAGCACACTGATTTCAGGTTGCTTAGGAGGACCAAACTGTTGACGCAATAGAGCATATCTTACAGCAATGGTTACTGCAACTTTGAG

>Isogroup_**191** CGCTCTGCCCCAGTGATTATCAGTTGTGTTACGGTAGAATTTATAGAAGGTGTCCCAGTGGCTGCTCTTTATTTCATCTCCACGGAGCCGCTTCATTTTTAAGTTCTGAGCAGGAATCTTTTTTGCGTTCTTGTCTGATATTCTTCCGTTTGGGCTGCTTCAAATCCATCAAAAAATCATCAAAACTTTTGTAGTTCCGGTTTCTCCAATGGTATTGCAACCCAATTCTTTGTAACAAACCCCTATCCTTCAAATTGCCGAATTCACCTTCAGATGGAAAAGTTATATGCAGTGATGACACGTTCAGCTTGGTAGCCAAATTCTTCAAAGCTTTTACTAAAGCATCAAAAACTTGATCCCGGTAGGATGTATCTCGAAGTAATATTCGTTGACCAGTTACTGGAGTAAAAGGCACACAAGACTGCAACTTTGGATAATATTCAAGTCCATAACTGTGGTAAGCTTCTGCCCATGACTGATCAAATACAAACTCGCCCCTAGAATGGCTTTTAAGGTAAAGCGGAACAACACCTACAATATCCCCGTTTTCATCACGTGCAACAACATGCAGAGGTAACCATCCTGTTTCCTTCACTGCAGAACCTGATTCTTCTAAGCTTGAGAGAAATGCATGGGTAAGAAAAGGATTTAGCTTTTCTGGTTCAGCTGAGTCAACTGCACAGGCATCCCACTCTGCCGGAGGTATGTCCATGATCGAAGATACAACAGAAATTGATACCTCACGTGGCTTCATGTCCACCTCTGAGGCTGAC

>Isogroup_192 AGCGGCCGCCCGGCGAGTACCTGAGGAGCTCAGCAATCTTGGTCCTGTTGGTGGAGTCCTCATGGATGCCCAGCTTCAGGTTCTTGGAGAAAGCCTCATAGAACTTGTTGTAGTCCTCCTTGTTCTCAGCAATCTCAAAGAAGAGCTCGATGCACTTCTTCACGAGGTTCTTCCGGATCACCTTCAGGATCTTGTTCTGCTGGAGCGTCTCACGGGAGATGTTCGAGGGGGAGATCCTCAGAGTCAACAATGCCCTTGACAAAGCTCAGCCACTCCGGAATCAGCTCCTCGCAGTTGTCCATGATGAAGACACGGCGCACATACAGCTTGATGTTGTTGGCCTTCTTCCTTGTGTCAAAGAGGTCAAAGGGGGCCCTCTTGGGAACAAAGAGGACAGCCTTGAACTCAAGCTGCCCCTCCACAGAGAAGTGCTTGACAGCAAGGTGCTCCTCCCAATCGTTTGTCAAGCTCTTGTAGAAAGCAGCATACTCCTCCTTGGTGATCTCCTCAGGCTTCCTCATCCAGATAGGCTTCTGCTTGTTGATCAGGGCCCACTCATGAGAAACCTCCTTGATCTTCTTCTTTTTCTTCTCCTTTTCCTCCTTCTCTTCATCAATCTCCTCAATCTTGCCTTCCTCAGTATCGTTCTTCTCTTCTTCATCCTCATCATCAGAAATTTCTTTCTCAGTGGTCTTCTCAGTCCAGAGAGAGATTGGGTAGCTGATGAACTCGGAATGTTTCTTCACCAGATCCTTAAGGCGGCGCTCCTCAAGG

>Isogroup_193 TCCTGAACATGTTCAGCACTTGATTTTGGTTGGCTCTGCTGGCTTTTCATCAGAAACAGATCATAGCTCTGAGTGGTTAACCAAGTTTCGAGCAACATGGAAAGGCATGCTAGTTAACCATCTCTGGGAGTCCAATTTTACTCCTCAGAGAATAGTGAGAGGATTGGGTCCTTGGGGCCCAGATTTGGTTCGGAGATATACCACAGCTAGGTTTGGCACACGATCAACAGGTGAATTACTAACAGACAATGAGTCCAGCTTGCTGACAGATTACATTTACCACACATTAGCTGCAAAAGCTAGTGGAGAGCTGTGCTTGAAGTATATATTTTCCTTGGGGGCATTTGCAAGGAAACCTCTTCTGCAGAGTGCATCCGACTGGAAAGTGCCGACCACTTTCATATATGGCCATGATGATTGGATGAATTACCAAGGGGCACAGCAAGCACGCAAGGACATGAAAGTTCCTTGCGAAATCATCAGAGTCCCACAGGGAGGACACTTTGTGTTTATAGATAACCCAGAAGGTTTCCACTCGGCGATCTTCTACGCATGCCGGAAATTTCTGTCTGCAGGTGCAGAGGAGGGCCTCTCTCTTCCCGATGGCCTGATATCTGCATGAGGCGTCATCTGGTGCCATCTCATGCCGAATAGCGGCGTGAGCATAAAGCAGAGCTATACGACGATAGGAACTGTTAAAAGACAGATGCCACTGGTTTGTTTTTGTATGTATGAGTTGTGTAAATATGTGTCATCCATATGTTCTTGA

>Isogroup_194 TCCGGCTCTTGAGGACAGTCCACATAATAGTGGTCGTGATTATCCGATTGGTTTGAGAGCCTTTTAGCCATTCTGCTTGGGATAGTTTGAAGCATGGCGAAAAGGCTCACAATAAAGACTGTCAGCCATGCAACAATCAAATTCCGAATTCCTTCAAACGTGGTCGGTATATTACTCAAGGATAATGAACCTGATGAAGCAGCTGTTGGACTAGCTCTGGGGCTGCCCCATCCATCATCCACAACCTTGTCAATAACAGGGATGCTTTCTTCAATAGTGGAACGAGTGGCAGATGTGGATGCATGTGCTGCAAATTTTGACTCCTCATGGACAGGGGTCAATAGAGGGTTTGTTATGATGTTCTTTGGTGCAATAGGAGATGCAACATCTTCCACTTCAGACCCAGACTCAGCAGATGCGTCACTACCTTTTCTATTTGGACGATGTGGCCTAGCATAACCAATAATCTTTCCATCGGAGCTTGATATGGTCACGATTTGCCTGTCATAATTCGCCTCGCCATTCAGGACTCTCTTTATTGTGCTGGGATCCTTCCAAGGACCTTTATCTGATCTTTGACAACCTCCGTGTTCGTCACACTTGCATTTGCCACCAAAAAATTCTGGCAATTCACTCTCATCAATTATTTCAAGTAGCTTATTTTGATACTTGGTTCCAAGAACATGAATCTTTGAAGCAGTTTTTGGATCAAGAAAAGATTTTATTGTGCCCCATAACATCTTGAAGCCTTGGCCAGCATTAATTACG

>Isogroup_**195** CAAATACGCATATTATCATCAGAGGAAGGGATTCAAACCATCACTGGTTTGTGCCGATACATTCAGAGCTGGTGCTTTTGATCAGTTGAAACAGAATGCAACCAAGGCGAAGATACCGTTTTACGGAAGCTACATGGAATCGGATCCTGTGAAAATTGCTGTTGAGGGGCTGGAAAGGTTCAGAAAGGAAAACTCCGATCTCATCATCATTGACACAAGTGGACGACATAAGCAAGAGGCTGCACTCTTTGAGGAAATGCGTCAAGTTGCAGAAGCAACGAAACCAGACCTGGTGATATTTGTGATGGATGGTAGTATTGGTCAGGCTGCATTTGATCAGGCACAAGCATTTAAACAAAGTGCTGCTGTTGGTGCTGTGATTGTTACGAAAATGGATGGTCATGCAAAAGGAGGAGGTGCACTTAGCGCGGTTGCAGCTACAAAAAGTCCAGTAATATTTATTGGAACTGGAGAACACATTGATGAATTTGAGGTTTTTGATGTGAAACCATTTGTTAGTCGTCTGTTAGGCATGGGAGACCTGGGTGGCTTAATGGACAAGATCCAAGATGTTATGCCTGCTGATCAACAACCTGAGCTTCTGGCAAAGCTGGCTGAAGGAACCTTCACTCTCAGACTTTTGTATGAGCAGTTTCAGAATCTTCTGAAAATGGGTCCTATTGGCCAGGTCTTCTCTATGTTGCCTGGATTCAGTTCTGAGTTGATGCCAAAAGGACACGAGAAGGAAGGCCAAGCGAAGATTAAGCG

>Isogroup_196 AGACGCCGACCACACCACGACTAGCGTGGTCGCGGCCGAGGTACGTGAAATTCGTGAAGAGATAGCTCTGGTTAAGTTTACGTCTCAGAAGAAGATAGCTGATGCTCAATCTCTTGAGGCTAATTTGGAGGAAAAATCCTTGGAAATCGATGGAAAGCTCCATGCCGCAGACGCGAAGCTTGCAGAGGCCAACCGTAAGAAGTCACAAGCTGACCGGGATTTGGAGGAGGTAGAAGCTCGTCAACGTAGGTTGGAGAAGGAGAAGCTATATTTTGAGACTGAGAGGAAGGCCCGAGAGAAGCAACTCTTGGAGCAGGAGGACTCTCTGCGTGACTGGGAGAAAAAGCTTAAAGAAAGTCAGAGCAGACTTGTTGACTTGCAGAGATCAGTAAATGACCGAGAGGAGAGAGCAAACTTGAACGACCAACTTTGTAAGAAAAAACAGGAAGAACTGGAGGAGGCAAAAAAGATAGTGGAGTCCACTAAAATCACTTTGAAAACAAAGGAGGACGATATTACCAAAAGGCTAACTGAATTACGCTCACAAGAAAAGGATGCTGACTCAAAGCGCAAGACTTTGGAAGAAAGAGAGAGGATTTTATCTGAGAGGGAAGAAAAAGCTAGCGCAAGAGAAAAAATGGGGCTCCAGAAGCTTATCGAGGATCATAAGGTGAAGCTTGAATCAAAAAGACGAGATTTTGAATTAGAACTGGAGAATGAGAGGAAATCTTTCGATGAAAAGATGAAACAGAGAGAAGCTGATTTGG

>Isogroup_197 GTGCCCGTTTTTCGACATAGGCCTCAATCAAGCCCTTCAGCTGCATCTGCCCAGCTGGAGCATTCACTCTCTGCATCTTGACGATCTCCTTGTTAATCTTTGGCACTAGTGTATCATCTTCATCTAGAATGTATGCAAGGCCACCAGTCATTCCAGCTGCAACGTTCCTCCCAACTTGGCCAAGTATAACTACACAGCCACCAGTCATGTATTCGCAGCAATGATCTCCAGTGCCCTCAACCACTGCTTGCCCCAGAGAGTTCCGAACTGCAAATCTTTCTCCTGTCTTGCCTCTCACAAATACTTGACCACCCGTCGCTCCATACAGACAAGTGTTTCCAACTATAGCAGCATCCTCAGGAACAAATCCTGTATCATCTACAGGAACTACAACCAGCTCTCCGCCAGCCATGCCCTTCCCCACATAATCGTTGGCCTCCCCTACTAGGCGAACATTCATGCCAGGGGTCAGAAAACAACCAAAGGACTGTCCTGCACTTCCAGTAAACGTAATGTTCAGCTGGCCTGCAAAGCCTGTGTCCCCATACTTCTTAGCAATGGCACCAGCTACGCGGCCACAGACAGCTCTATCGACATTATAAATTTGATATGTCTTTGAAACCTCTTTCTCATTCTCAATTGCATCGATTACCTCAGGATCTGCGAGGATTGTCTCATCAAGAATAGGGCCATTAGAGTGGACATCCTGGCTTCTTATTTGAGAGCTGCTCCATTTGGGTAATCCAGCATTCATTAGTAGGTACCT

>Isogroup_198 GCGGCCGCCCGGCGAGTACGATGCATGTGAAATGGGAGCTGACAAAGTGCCCTGAGGTATCCACTGATAGATTCTCTCCATTTTGGACGCGTCATATATAGCTAACTGAGTCTCATGGACTACTAACAAGCGACTTTGGTCAGTATTAAACTGGACCTTTGTGTCTCCTGATGGAGTCTTCCCAGCTGGCATTTGTATAGCCACAGATTTTTTCTTTTCCCAAGTATCGTTGGCCCACACACATAACTGTGCATCAGCGCCTGAAGAAACAAGTATATGCAGGCTGTTGGAGAAGGCCAATCCGGTTATCCTCTTTTGATGTCCTTTGAGTCTCATCTACCCTGACATTGTATATGTGGATGGTTGAATCTTCCATTCCAATTGCTATGATGTTATTGTCTTGAGGGTGAAATGCTAAAAAGGTCGACGCTGGTGGAGGTGGCATGAATGTTGTCATCACCTTAAATGTCATCATGTTAAACAATGAGACCTTTCCACCACATGCAGACATCACATAAGAATCATTCTTGGACAGTGCAATGCATGGAACTGACTCTTCAGGATTTGCTTCTCCGATATCATTTGTCATGACAAGACCGCTATTTGGTTGCCAATGCTGTGGCACAACACTGGCTGTGGCCTTCCCATTTGGATTCTGCTCGTTACGACTCCATTTCCACAGCCTTTGAATGGCATTAGACCCTAGAGCTAACAAACTGACACCTGAATTTGTGTACCTCGGCCGCGACCACGCTAGTCGTGGTG

>Isogroup_199 GGATTGCAAAGCTTGCAGCTCCTTGTCTCACAGAAGCATTACATATGTATCCAGATTATAAAATAAAGGTTGTTGGACATTCACTTGGAGGTGGAACAGCAGCTCTCTTGACATATGTCCTGAGGGAGCAGCAGGAGTTTGCCTCTGCTACTTGTGTGGCATTTGCTCCAGCTGCTTGTATGACATGGGAGCTGGCAGAGTCAGGACAGCATTTTATCACTACAGTCATCAATGGAGCTGATCTGGTGCCAACATTTTCAGCTGCAGCAGTAGATGACCTTCGTTCAGAGGTGACAGCATCTGCTTGGCTGAATGATCTTCGTCACCAAATCGAGCAAACCAGAATCCTGAGCACATTCTATCGGTCTGCATCTGCCCTGGGATCACGACTTCCTTCTATGGCAAATGCTAAAGCCAGAGTAGCTGGGGCTGGGGCCATCCTAAGACCTGTATCTACTGGGACACAGGGTTGTAATGAGGAGGGCTCGCAGTGTAGCACAAGCAGCCTGGACAAGACCAGGGCTACAACTATCCTCATGGGCATGTATTGGGCCAAGGCGTCGAAATAATACTACATCTACCTCAACTGTCATGTCAGAAGAAATAATGACTTCCACTGCAAGTGGTGGCTCTGAATCCACTTCACTGTTGTCCGAGACCACTGTGGAAACCACGCAGACAGTTACATCTGAAACCATGCAGTATGCTACATCAGAAGAGGTACCTCGGCCGCGACCATGCGGCCCCTATAGTGAGTCGTATTAG

>Isogroup_**200** GACGCCGACCACACCACGACTAGCGTGGTCGCGGCCGAGGTTGCTTCTGTTGTCAGATGTTGTGAACTGCCTCAAGTTTCTCTCAGGGAAAAGGCTTCAAAAGTATTAAATCTGCTGGTTGGGGAACAACCTACTGGAAACAAACATCTTTCAGAATCAAAAACTACACTACCAACTGTTCAGATGCCTGACTTGATTGATACGGGGGATCAGGATGATCTAGGAACTCAGAGTTCAGCTCAAGAAATCAGTGAGCAGATAATAGGGAATAGCACACATGTTTCTTCAGTTGATGATTTGCTTGGCGGGGAACCTATTGGTAATATCAGTGTGACTAGCAATAGTAATGGAAGTGATCCATTTGCGGATGTATCATTCCATGAGGAAGAGGTTAAGGAGACAAATGACCTATTCTCAGGAATGACAGTAGAGGAAAAATCCCCAGCATTTGTGCATGATAACTCTGTGATGGACAAAATTGAAATGCCAGATATATTTGGCAGCAGCCCTGAGCCATTGTTCCAAGAAAGAGTTGATGATAAAGGAACTGTCGACGATTTAATGGCTGGTTTGAACCTTAATGGCACTGCCCAAGCTCAGCCAGGAGTAAAAGCAGAAACTAATAGTAATCTCAGTGGTTCACAGTTCTTTGACATGAACAGCCAGACTAGCCACGTGGCGAGTGCTGCAGCACTGAATGGTATCCTTGGTCAAAGCTCATTCTATCAGCAGGTTCCCTTGCAGTACCTCGCCGGGCGGCCGCT

>Isogroup_201

CTGGACCAACTTTGTCCACACGACGCCCAAAGCATGCTTGTCAGAATGCGTATGCCATCGAGATTCTTCCACTTAAATGTCAAGATTCTGTGGATGAGGAGACCTTACCATTATCACTTCCCAAGGCTGACAACCACTACCTAATGGAAGAGCTTCCTCTTAGATACAAAGACTTGAATATTTCCTGATGAATATTCCTTTGCCCACATGTGCTAGGAGCCTCATGGCAGCATTTGCCGCGCACAATTTCAGACAGGATTGGTTGACGAGGAACCGGACTTGGAGGTCCTACCTCATCTTTGCTGTTGGATAGAATGAGGTTATGGAAATTACAGATACTATCCTTGCCCCTCCTTGGATGATGGTTTCATCTATTATAAGGAGAAACAAGGGATGATGTCAACCGCTAGAGCAATTAAATGATGGTTATTAAGAAATACCAGTTCTGGAAAGCTGGATAAAGTGTACACTATGATACACAGCTCAAATACTGCTTCGCAGTCCAGTTCTAACCATCTCACAAATCGAAAGCACCTGGCACAAAAGAAAACGAGAACGCCTATCCAAAAGGCTGGAGAGAGACAGACATGAACATGTAGACGGAGCTGACACTAACAAAAATGGAGCTGACACTATGAGCGGCTGAGCTCAAAGTAAAACAGGCCAACCTTACAACAACGTCCCACTCAGGGAGCTCCTAGCCATCAGCTTCACTTATGGCACACCTCGATCATGTCTTCATCCTCCAGGTCAAGGTCTTGAGGT

>Isogroup_202 CTTGACATCGAAGGAACAACAACTCCAATATCATTTGTGACTGATGTTATGTTCCCTTATGCCCGTGATAATGTGCGGAAGCATCTGATTTCTACGTATGATTCTGAAGAAACCAAAGAAGACATGAAACTGTTGCGTATCCAAGTTGAAGAAGACCTGAGAAATGGAGTTGTTGGGGCTGTTCCAGTTGCACCCAGTGATGCTGACAAAGAAGAGGTTATCAGTTCGTTAGTCGCTAATGTCGAGTCCATGATCAAAGCAGACAGGAAGATTACGTCACTGAAACAACTTCAGGGTCATATATGGAGGACTGGATTTGAAAGCAAAGAACTGCAAGGAGTTGTTTTTGAGGATGTTCCCGAGGCACTGAAGGACTGGCAATCTCATGGTGTGAAGGTTTACATATACTCAAGTGGCAGCAGAGAGGCACAGAGGCTACTATTTGGCAACTCATCCTATGGTGATCTGCGACAATACTTGTGTGGTTTTTTTGACACCACAACTGGAAACAAAAGAGAAGCAAGGAGTTACTCTGAGATCACAAAATCGCTTGGGGTGGACAATCCATCTCAAATCTTATTTATCACAGATGTGTTCGAAGAAGCCGTTGCTGCAAAGAGTGCAGGTCTCGAGGTAATAATCTCCATTCGCCCTGGGAATGCACCACTTCCTGAGAATCATGGTTTTAGGACAATCAGTTCTTTCAGCGAGATCTGACAAATGTGCAAGAGACAAGCATGCAGGAGAATCACCTTTGGACGTG

>Isogroup_203

CTGGGGGTCCTGGCCTCCCATCCGCTCTCCCGTGATATCCATATAGAGTCCCTTTCATTAACATTTCATGGTCATGACCTTATCGTGGATTCAGAGTTGGAGCTTAACTATGGGAGGCGTTATGGCTTGCTCGGCTTAAATGGCTGTGGAAAATCTACCCTTCTCACAGCAATAGGCCACAGAGAACTTCCTATCCCTGAACACATGGACATACACCATCTCACCCGTGAGATCGAGGCTTCAGACATGTCTTCGCTGCAAGCTGTTATCTGTTGTGATGAAGAAAGAATCAAGTTGGAAAAGGAAGCTGAAATTTTGTCTGCACAGGATGATGGTGGCGGTGAAGCTTTGGATCTTGTATATGAACGGTTAGAAGCAATGGATGCATCAACTGCTGAAAAGCGTGCTGCTGAGATATTGTTTGGTCTAGGTTTTGACAAGCAGATGCAAGCAAAGCCAACTCGAGATTTTTCTGGTGGTTGGCGTATGAGGATTGCGTTGGCAAGGGCGCTGTTCATGAACCCGACCATCCTTTTGCTTGATGAGCCAACTAACCATCTCGATCTTGAGGCTTGTGTTTGGCTGGAAGAGAAATTAAAGAATTTTGAGCGTATTCTTGTTGTCATCTCACACTCACAAGATTTCCTGAATGGAGTGTGCACTAACATCATCCACATGCAGAACAAGATCCTCAAGTTATATACTGGTAATTATGACCAGTATGTGCAAACCCGCTCTGAACTTGAAGAGAATCAAATGAAGCA

>Isogroup_204 TTACGCAAGTCCGGTGGAAAAACACGAGTATCAAAACTTACATTCTGAGAATTTGCTTCACTTCCTTCACTTGAACTTTCACTTCCACTTTCCCCACTTTGAGAGGTGGCTCCATTGGCTGATGCCCCAGAGGTCTTACCATGTTCGACGCACTTCTTTCCTGTAATCATGTTCAAGCTACCTAAACTTCCTTTGGATCTTTTAATGGGACTCTTTTCTTTTCCTTCAGAGGATTTGCCATCTGTTTCACCACCAGCGGTAGCAGTGGCAGTTCCAGTTACATCAGGATTGCCATTTGGAGTATTCATAGCATACTGAGCAAATGGGTGTGCGCCCGGGGGCATTGATGGGTGAGCATAAATTCCTCCAGGAGGGTAGATGACATATGGCGGAGTCCCATATGGTGGCATCATAGGCTGAGGTCCCCACATGTAAGGATGACCCTGTGGGCTTGACACCACAGGAGAGGGGAAAAAACCCATGTGGTGGAATTGGAGAATATCCCTGAAAGTTGGTCCAATCTGGGTAAACAACTGGCGTTGCAGCACTTGAAGTAGCAGGTGGTTGCTGCTCCTGAGGTATAGATGCTTTGCTTGCCTTAGCAGGTGCATCTGCTTCACTGCTTCCCATGGTAACTCACAGACACACGACAACAATCACTCCATTGCCCAGACACGTGAAGTGGCATAAAGTGAACCCTGAATTGGCCATACAAAGAGAAAAAGTAAGCTAAAATCTACCAAAATTGTATTACTATAACCACT

>Isogroup_205 CAAAAGGTGCACTGGTTCTACAAGTTCAGCATTTCTCAAATATGGTTCCATGAATTTGAACAGATCAACAAGCAGCACATGGAAAAGAGGCCACCCTTTTTGCAAATTGCACGTCAGCAATCTTGGCATGAAGGTTCTATGACTCACCAACTCAAGCCAAGCAAAGCTCAAACCAGGAATTCTCAAGGGTTGCAGTGCATGGAATGCATTTGCAAATGTAGTCAACGCCTGGAAATTAGCACCGTCATGATGTATGTCAGAAGTAGTAAGGTCATTCAACCAATTTATAAAAAGACGGAAGTATGGTCGGGGGTTGAATGAAGTTTTCTTCTCTTCAGCATCTTTCTGAATGACCCTAACTGTCACTGAAAGAATCTTAGGAAGAAGGCTGCCTTTAATTGGTCCTATTTCCACCGGAGAATACTTTAGCACCATAGACACAAGCTTTGAATATGAATCAATGGAGATATATGATATATGAGATGACTGCTGAGATAATCCACCAGGAGCGACAATTTGCTCAGAGATCAGAGAGTGTGTAACAGCAAGTTCCGTGAGAACACGGAAGAAGCGCTCAGTGAATTCATCTCCCTTCAGCAAGCCAATATGTTGCAATTGCATAACATAGCGACTATATGCCGCATCAGAAGCATTTGGGTGATCACATACTTGACACCACTCAGAAAATAGGTGTGCAACCTGGTCTTGAAAAGCTATAGAATCAGCAAGGGTTGTGTCATTAACACTGTTTTCCTCTTTGTTT

>Isogroup_206 CTGAGATTAGCCCAGATTTAGGGTCTCCAATTAAGTGTGAGTCAATTTCTAGCGATGATATATCAAATAACAAAAAAATATAAAGGATAACTTGAATGCTAATAATTTTGATTTAGAACTAGAGATTAAGCCAGAGATGGTCAAACCATCGTCTGGCATTACTTCCTTAGGTGGGGATTTGCAGCCATTGGATGGTGACAAAGAGCTGGTCAAGAACCAGTTGTCTTTGGAAGACATAGATTCCACAGCTAATGTGGATGAAGTTGGCTCTCCAGAGAAATTAAATTTGGACAGGAGTTCAGGTGATGAGTCGATGGAGGAGGATGTTATGGAGATTAAGCAAGTTGAGTCCAATGTTAAATCTGATGATCTCAGAGGAAAGACTGAGCTTAACTCAGAACATGTGAAAGAGGTGACCCTCCCTGATTCTGTTGCTGAGGTTTCCTCTGTTGATACAAAGGAAGTTATAGCTGAAGACAAGCCATCAGCTTCAATTGAAAAGAGGAAACTTCAAGCTGAAGAACCTGTTACAAACACTGAGCCAATCAAACGTCAACGTCGATGGGCTGCAGACAGTGGCAAAGTCCCAGAAAGACAACCATTGAGTCAAAGCGGTTCTGATGCTCCTAAAGAAATTTTCCAGCCTGCTCTAAAACGTTCTTTTGGCAGGTCTGATTCAACAGCAAGTGGAGATTCTCCAAAGGAGCGAATTGTGCCACCATCTCAGAAACCTGCTACAACTTCCTTGAGAATCGACCGATTT

>Isogroup_**207** GGGCATGCTCTCAAGCTGGTGCTGCTGGACCAGCTCCACTGTTAAAGACACTGTCAGACCTAAAGGATTCCTGCTGGAGATTTTTGAGGCCACACACAATTCGAGGAACTGCTTTGGGATCCACGGCTTTGGTTGCTAGAGCCTTGATAGAGAATCCCCAGTTGATAGATTGGCGCTTGGTATTCAAAGCATTATATGGACTTGTGGCATTGATCTGCGGCAACGGTTACATTGTTGGGATTAATCAGATCTATGACATTGGAATCGACAAGGTAAACAAGCCATATTTACCTATTGCTGCTGGTGATCTCTCAGTTCAGTCAGCATGGTTATTGGTCGTAGCATTTGCAGTGGTGGGCTTCTCGATTGTCGTTTCAAACTTTGGATCTTTCATTACCTCTCTTTACTGCCTTGGTCTATTTCTTGGCACTATTTATTCTGTTCCTCCATTCAGACTGAAGAAATATCCGGTCGCTGCTTTTCTTATCATTGCAACGGTTCGTGGATTTCTTCTCAACTTTGGGGTTTACTATGCTACTAGAGCTGCATTAGGTCTTACATTCCAATGGAGCTCGCCTGTTGCTTTTATTACATGCTTTGTCACAGTATTTGCTTTGGTCATTGCTATAACCAAAGATCTTCCAGATGTTGAAGGAGACCGCAAATTCCAAATATCAACTTTGGCGACAAAGCTTGGTGTCAGAAAGATTGCCTTCCTTGGCTCTGGTTTACTATTGGCAAATTATATTGCTGCTATTGTT

>Isogroup_**208** GACGCCGACCACACCACGACTAGCGTGGTCGCGGCCGAGGTACAGCAAGTTCTAGCTACATGAGTTGGTCAGCTCGGTTGAGATCCATGTATGGTGATGAGTTTCTAATCTCATCTGCATCACTGCTAGCGTCAGATTCACCTCCTAGCCCACGGCTGTCAGAACAATCCGAATCCATGTCATGGCAAAAATTATGAGCAGAAATTACAGAGTGCTCGTGGTCTTTATCCTCGCTCTGATCATCTTCGTAGTCAGAACCTTCAGCATCCTCTTCATCCCTGGCATCTTCATCTTCTTCGCTGTAGTCATCATCATGTTCATCAGGATCATCATCGTCGTCGTAACTAGACTCACCGTCATCCTCAGCAGTGGAGCCGACAACCGGGCTGTCAAAGCTCCTGAGATAAGACTCGTCGTCTTCATCCTCTACATCTTCATCCTCGCTCGAATACGCAGAATCTAGTCTGGCATACAGCTCCTCGTCTCCTTGGTCCTTGACGTCTTCAATTTGGACCAGCTCTGATCTGAGCTGATTCACCCTTCTATTTAACTTTCCAATATGACGATTGATGGCCTCCATTCGCTGTAGTTCAATGGACCGAGCTAAGGCCTTCCTCTTTGATAAAAGATTCCTGGGTCGTAGCATGGAAGGCCGTCTATAGTCCTTCCCACGAAACACAACAATGGCATAGCCTTTGGAGACCTTGTCCACAGAAACTAAAACACCCCCACTCTCAACTTCAAGTGACAGTGCTGTCCC

>Isogroup_**209** CACTTGACCATTTACCCCCTTGTCTTGGCCCTCTTCATTCCAAGGCTGTTGTGGAAAGGGGATGCAGAACCAGATCTTTGCATCCCTTCTTCACGAAGGGTGCCATTGAGCATTGCAAGCTCACGGAGCTGCTGCTTCTTGAAGAAGTCCTGAGACTCATCCATTGGCTTTAGCAGATCCTCAAGGATCTCACGGGCTTGCATCAGACGAGCATCAATAATTTCAACAGGCATCTCCGCCTCAACCAATATATGAAGGGGTTCATTCAGATGTTCATATCCTGGCTTCCCTCGCATCATTTCCTCCCGAGCTGGATCTTTAATGCTGCCCCGACCTCTTATTAGCACACGGCAGTCAGTAGTTGCCTCCACTCGCTTCAAGGAATTTCCTCTAGGACCAAGGATGCGACCAACAAAATTGTATGTTGGATATTTGTCTACTGGAATGTCAACCCTCATTTGTTTCTTGAGAATGAATCCAGATGAGCTGCCTTGAGAACCTCCACGCCAGCTATAAGCAGATGAACTTTCTGATTGAAATGCTGATGTCCATCCACTCATGTCAGTTGCTGCTCCGTTTGAATACAATCCTCCCGATGTTAGTGGACTACCATGTTCAAGGCCACTTTGATTTAAAAGGGAGGCGTTCTCCAACAGTGTGGAAACACGTATAATTTCTTGATTCAGCAACCGTATGCTATGAGGGAGCACTGGGACGAACGGGTTCAGCTTGTGGCGCTCCGCGAGCAGTTCTGCAAGG

>Isogroup_210 GGTAACTATGGCAAGGCCAATATGTGCAAACATAAGAATATTCCTGAGTTTCTTATGCTTTAAGTAGCGGTTGTAGATATATGTGCCAAGCATTAGACTGAACCACCCGATCACACGTGCAGTCCCTAGAAAGGATGCCTCCAGATGTAGAACCTCCGTTTGATAATAGAACATGACTGTTGAGATATTTGGCATTGCTGCATTCGAAATGAAAAACCCATGCCATAGGTCGCAAAATGGCTGGCTGCTTAAAAAGCTGTGCATAAACTGAAGAAGGCTGATTTCAGAGACAGGTAGAGCCTTGAATTGATTGCTTTATTTTGCTTCTCAAGAGCTTCAGATCGTTTGGACAATGATCTCCTTTTCCTCTTTTTATGAGCACCCTTTCGCCTTCTAGTGCCCTCATACTTTAATGATTCACCTGAGCCTTCTCCAGCAAAAGCACTATCAATATTCTGATCATTAGCATGATCATGTGCAGCATTATCTACCGTGCTCTCGAATCCTTTAGGAGATTCCTCGACGAAAATACATGAAACTAGTTGGAACAATGGGAGAGCAGAGAAAATGATATATATAGTATTTATCGGGAGATTCGACAATGCGTATCCTCCCAATAAGCTCCCAAATATCCCCCTACAGCCATTGATGACCAAGATAATGACTGGAGATCACCAGCAAATGCTGACCCTGCTGATCGAACTGCCTCTGCAATCATTGCATCTATAACAACATCTGCCATGGCAGATCCCAAGTTCT

>Isogroup_211 GAGAGACGCCGACCACACCACGACTAGCGTGGTCGCGGCCGAGGTACACCAGATGATGCTTGCAGGATTGGTAAAATAGGTGAGCTTTTTGTGTATGAGAACTTCGGACGGGTTCCAGTTGAATCTGTTAGTGCTGGTGACATTTGTGCTGTATGTGGAATCAATGATATCATGATTGGGGAAACTATAGCTGATAAGGTTGCTGGAGTAGCATTGCCCACTATCAAAATTGAGGAACCAACAGTTAGAATGTCTTTCTCCATCAACACTTCACCTTTTGTTGGAAAAGAGGGAAAATTTGTGACCAGCCGAAATCTCCGTGATAGGTTATATCGTGAGCTTGAGAGGAACTTGGCTATGAAGGTAGAAGATGGGGAAACTGCTGATACATTTCTTGTTAGTGGCAGAGGCACACTGCACCTCACCATATTGATAGAGAACATGCGGAGGGAAGGGTATGAGTTTATGATTGGACCCCCAAAGGTCATAAACAAGACAGTTGATGGAAAGGTGCTGGAGCCGTATGAGATAGCTGCTATAGAGGTTCCAGAGGAATATATGGGATCGGCCGTTGAGTTATTGGGGAAAAGGCGTGGGCAAATGCTTGACATGGAAGCTAGTGGGCCGGAGGGAGTCTCATTGCTGAAATACAAGGTTCCAACAAGAGGCCTCATTGGGCTCCGGAATGCAATTCTAACAGCTTCTAGGGGCAGAGCTGTACCTCGGCCGCGACCACGCTAGTCGTGGTGTGGTCGGC

>Isogroup_212 ACCGGCTGGTTTTCGACCAAGGTTCTGTAGCCTTCCTGGGGATCCTTCCTGGCAGCATGGAAAAAGAAGCCAGCCGTGATTGCTTTCCTTATTTTCGTGAAGTTCTTCCCAGCAGCAACAATATCCAATTTATATCTATCCATGATGGTGAGAAGCTGTTTCCTCACATCTTGCGCTCTCCTGAGCGATCTTGACTGAACAAAATTCTCAAAGCACCAAGGCCCTGAAAAGTTCTTTGCCTTCCAAGCCTCATATACAGCAAGAAGAGTAAGATGATCTCCCTCCGGCTGGAAAAATTTGGCCCTTTTCTGATCAGCTTGAGCCTGCTTTTCTCTAGGCCTATAGAAAATATTCCCTGTTTGAATCATCGCTATGATAGTCAGTATCTCATCACTGCAGCCAAGGTCGACGCTAGCTAGTAGCATCTTTGAAAGCGGTGGATCCAGTGGGAATTCAGCCATTTTTCTACCCAGTTTGGTAAGAAGGCCCTCCTCATCGAGAGCACCAAGGCTGTAAAGCTGTTCCATAGCAGAGATGAGTGCTTGAGGTGCTGGGGGGTCCATAAAATCAAAGGATAATAGGTCATTTATCCCCATTGCCTTCATATTAAGCACTGTAGATCCCAAATTGATCCTCTGGATTTCTGGAATCGTTGTGGGAGACATTTCATTGCGGTAAGCACTTTCAGTGTATAGACGATAACATTTGCCAGGTCCAGTACCTCGGCCGCGACCACGCTAGTCGTGGTGTGGTCGGC

>Isogroup_213 AGCGGCCGCCCGGCGAGGTGAGAAGATGCCTGAAGGCGTAAATGACAGCGGCCTTACCTTGACTGGGTTCCTTTTTCTTCATGCTCTTTTCATCGAGAAAGGTCGTCTGGAAACTACATGGACAGTATTGAGGAAATTTGGTTATGATAATGAAATCAAGCTTACAGATGACTTCATTCCAACGTCAATTAAACGTGCTCCTGATCAAACGCTGGAATTTACAAATGAAGTGGTTGATTACCTGAAAGGAATATTCCACATGTTTGACATAGATAATGACGGAGCTTTGCTACCTTCTGAGTTGGAGGATCTTTTTTCAACTGCACCCGAAAACCCATGGACTTCTGATCTATATAAAGACTGTGCTGATAGAAATGTCTTGGGTGGGTTATCACTTGAAGGATTTCTTTCGAAGTGGGCTCTAATGACACTTCTAGATCCAGCAAGTAGTTTTGCCAACCTTGTATATGTTGGCTATTCGGGCGATTTTAATTCAGCATTTACCGTCACAAGGAAAAGACGAGTGGACCGTAAAAAGCAGCAGACTCAAAGAAATGTGTTCCAGTGTTATGTTTTTGGTCCCAAAGGTTGTGGAAAGACTGCATTGCTACAAGCATTCCTTGGAAGGCAACCTTCTGATGCTCTGCCAACTAACAGCGACCGATTTGCAGCAAATACTGTTGAACTGTCTGATGGGACTAGAAAGACACTTGTACCTCGGCCGCGACCACGCTAGTCGTGGTGTGGTCGGCGTC

>Isogroup_214 GGGCAGGTACCTAAAATCAGCCAAATTCATAAGAAGCTCCATCTTTGCAGAACCAAACTAGATATTGCTTCCAACATTCTTCACCACGAAGATGCACGTGTCTTCATGTCATCATATGATCATATGAGCAAAAAAAATCTCTCAAAAAGGAAAAAAAAAGCGAATAAAGGAGAACCTTATTCATCCTATACAAAAGAAGCAATTGATTTGAAGCCACCCTCGTTGATATGGTTGGCTAGAGAGCTGTCACCTGTCTTCACTATCTTGCCACTTTCCATGATGTGGACATAGCTTGGCTTAATAAGATCTAACAGACGTTGGTAATGTGTAATCATCAGAACAGAATTTTGGGGAGTCAAGAGCCCATTCACTGCGTGAGCTACATCTTCAAGTGCATCGACATCCAATCCAGAATCGATCTCATCGAGGAGGGCTAAATCAGCCCCAACAACAGAAAGTTGCAGTATCTCATTGCGCTTTCTTTCTCCTCCACTAAATCCTTCGTTTACGTTACGATCAAGGATCTTTGGTTCCATCTTTAAGGCCTCGACCTTTTGTGATACAACTGAATAAAACTCAAGAGGCCCCAAAGCTGGGAGACCATCCTTTTCTCTGCGAGCATTTAATGCCATGAGCAGAAAATCATAATTGCTCACTCCAGGAATTTCAATAGGCGCTTGGAAGCTCATAAAGAGACCTGCAAGAGATCTTTCCTCTGGCTCCATGTCAATCAAGTTCTCACCCTTGAACAGAGTG

>Isogroup_**215** CTGTTCCTTATGTCAAGCAAGTGAAGTTGTCAAATACTGGTGGGAGGCTTATTATTGCTTCAGACGGCATTTGGGATGCATTGTCCTCAGAGGCAGCTGCAAAGTGTTGCAGAGGGTTGCCTGCTGAACTCGCTGCCAAACAAGTAGTTAAGGAGGCACTAAGGATAAGAGGCCTAAAAGATGACACAACATGTGTCATTGTTGACATAATCCCACCTGATCAAACTATACGACCCCCATCGCCACCAAAAAACGATGAATAAACTGAAGTCTCTGATATTTAGAAGAAAAGTGAAGGACCCCTCAAATAAATTGACCAAGCAGCTTTCTGCAGTGGGTATGGTTGAAGAGATATTTGAAGAAGGTTCAGCGATGCTATCAGAAAGGTTAGGTAACGACTCAAGTGGTGGCAGAATATCCGCAAGCTTGTTTACTTGTGCAATCTGCCAGGTAGATCTTGAACCAACTGAAGGTATATCAGTTGATGCATGTTCATTATTCTCTTCCAGCTCTAAGGCATGGGAAGGCCCTTTCCTTTGTTGTGACTGCCGCGACAAAAAGGATGCAATGGAAGGTAAACGACCGAGCGGCGTCAAGGTTCTGTGATCAATTGCAGCCAACCACCATTGTAGCTTCAGCAAACTCCCCTTTTTCATGTTTAGCGCCCTCGCGTTACAAAATTTTCGTTCAGTTTTAGGAGTTTGGGTCATTTAGGCTACATTTCTTAAGTTATGTGAGGAGCCAACAGTGTAAGTA

>Isogroup_216 TCACATTTGCAGCAAAACTCTGGTTGGGCTGAGTGGCATGGTAGTATCCTAACAAAGCGTAATGCGGTAGAAAATGTTTACCAATGGGTTTGTGGCCGACCGACATCACTGCAGGATCGAGGTAGAGACAGCGACGATGAAGACTTCCGAGACAGGGACTATGATGTGGCTGCGCTTGCTAGCAATCTGAGCCAGGCATTTAAATACGGTATTAGTAATGAAGATGTTGATGAGGCTCAACCATCACAAGAGCGAGATGATGAGGATGTGTATTTTGATGATGAAGCTGCTGAAGTAGTTATCTCCAATATTCGTTTTGCTGATGATCCTGACAGGAGCTCCTTTTTTACGAACTCCAATTGGTTTGCATTTGATGAGGACAAAGCACTGAACGATGACCCAGAAGCTTCCCTTTCTGCAAAACTGGAGTTGCCTTCACCAAATGTGGATGACGATATGGATGAGGTCATTCTTGGTGAACCTGTAGATGGCACAAAAGGTTCGGATTCACTTTTAGCAACCTCTGACATAGACTTGAGTGAAGAGCCTGGTCACACAGTATTGACAAACGGCCCCATTGATAAGTTGGAAGATGACATCAGACCGCCAACTCCAGATGTAAAAGAGAGTCTGCCTGAGTGTGTTGAGTGGACAGAAGATGCTGAACCTGCTGAGGTATCAGTGAATACTGCTGTTCTAAATTCTGAGGTTGGAAATGAGAATGCAATGGATGCCACTGGTGAACTTGTTTCCA

>Isogroup_217 CCGCTGCCTGATGAGCTTGGCTGGCTCTCCGGCGTTGCCACTCTCCAGCTCTCCGGCACTCATCATGATTGGGTTGATGCCCATCTTGGCGAAGACAAGCTCACACTGGAAGGATTTTCCTTGACCCTTGCCTCCCCAGATACCCAAGATGAGTGGGATCTTGATGTTGGGCAGGGTCATGAAGTTCTTGGAGAGGTGGACAACGAGCTTGTCCATGAAAGCTGGGGCGATGTAGAAGCCTCCCATGGTGTTGTCGAAGTCGTACATTTGATTCGGGGGAATCACGAGGCAGCAGATATTAATGCTCTGTTTGGGTTCCGAATAGAGTGCATAGAGCGAATGGGTGAGCGAGATGGAATCTGGACCTGGCATCGTGTGAATAGGTTATTTAATTGGCTTCCTTTGGCTGCCCTAATAGAGAAGAAAATAATCTGCATGCATGGTGGTATCGGACGCTCCATTAACCACATCGAGCAAATTGAGAATCTTCAGAGACCAATTACTATGGAAGCAGGCTCAGTTGTTCTAATGGATCTTCTATGGTCGGATCCAACTGAGAATGACAGTGTCGAAGGACTCAGACCAAATGCTCGGGGCCCAGGTCTTGTCACCTTCGGGCCTGACCGTGTTATGGAGTTCTGCAACAACAATGACCTACAGTTGATTGTGCGAGCACATGAGTGTGTGATGGATGGCTTTGAGCGTTTCGCTCAGGGTCACTTGATCACTCTTTTCTCGGCAACAAACTACTGTGG

>Isogroup_**218** ACCATTAAGCATCATAATGGCGATGCAAAGAGCAAAATAATTGATATTATAACCACCCTTTAGCAGTGCTCCACATCGAGTAATCAACCATACACCAGAATAGTTCTTTGCTCCAGAAATAATGAAAGCTTCTAGTGTCCACTTGGGATAGCACAATCCTTTAATAAATTGTGGAATTTTTGCATCAGTGCCAACCAGGACCAATACCACAGGTAGCAATGCAGAACACAATTGAGCTAATCCTAGCTCAAACCAAATCGACAGAGTATAACCAATGCCAGTTACACAGTAAACTAATGCAAGCAAGACCAAATAATTGTCTCGAAGTGTAGACCGTGGATTGTTGAAGAAATAGAATGTTGAGAGGAAGACAATAGGTTTAACCACGGTGTTGAAATGATCAATTGTATCTCTTGCAAGGAAGTAAGCTACAGAACTCATGCCAGATTCTCTTTCTCTCCAGTATTGCATTCTTTCTGGTGAAAATGATCGCAGTGCTGCAAGCTGGCACAAGAGTGAAACTGCAATTATGGTATACCCATAAGAAGCTACACCGAGCGTATCATCGTTAACTTTTGTGATGGTTCCAATGCATATGCCAGCAATACCCAGTATTAGGTAGTCAACAGCCTGTTGCACAGCTTCACGCAGACGTTGCTTAGCTACCCTGAAAACATAATTTTTCGCAATGGAGTTATGAACTGGACAAAAGCAATTAAGAAATTAAGGAAATCAATTTACCTCCCTAAATA

>Isogroup_219 AATCATGAATTAACAATGTCACTAGTCAGTTTCCACGTCTTCTGTTATTGTAGCTGACTTGAGTTTTTGGCTCACAAACTGCCCTTTAATTCTTGGTCTTTGCTCCGCAAGCTTCTTCCTGCTGTGATATCTCACCTTCTTCTCAAAGCATCTCTCTTTCCTTTTCATGCGGAATTTCATCAAGGCAGCTTCACGACGGGATCGATCACTATCCAGTCCGTTAATACCAAAGTTCTGGATGCCGCTCTCGTTGCCACTTTCTAGGGCAATTATTGTATTTGTGTTTGTATCAGTTTCACCGCTCACTGTCCATCCACTTCCTCTACGGATATCCTGGCTACAGCTAGCACTGTGGTTAGCATGCTCCGCATGGGCTCTCGACAAGTCAACTGGTTCTCCTGATTCCTGAAGAATTTGTCTTGAATGATGCAACTGCTGATTTCCTTCTAGCTGCCTGTGTTCATGAATGTCTGATGGTTTACTAAGATTTTGATGGTAATTAGAGTGATAAGATGCATGCTGAATTGCCATATGGTTGATGCCAGACGGATCACATTGCATAAAAGGAGCCTGGGGGTAGTATATCGGCTGCATAATTGCACCATAGTGATAGGGTATAGCAGCACCAACAGGAATTGGAACAGGTATAAACCCATACGAAGGATGACTCAATGTGTCCTTTCTAGGGGAGGAACTACTTGTGCCAGCATCTTCTCTCGCACTGCTGGAAGACGGGATGGCAACACCCTCT

>Isogroup_220 CTTCTGTCCCGTGCTATACGTGGGAGAAAAGTAGCAACGGATTAATGTGCGGAGAACTGAATGGCCCCGGTATGTAGCTAGTGACTGATCATGTGGATGCTTTTGTTGCTTCTGCTGTTGTGGATATCTTGAATATCTGTAGTCCCAGGCAGGGACTCTATTTTCAGAACCATCAGCATTGGATGTCATTTTCCTAATATCCCACATCTTAATAGCTTGATCTTTCCCATTTGATATGAAACATCGACCATCTCCACGACTGTCAATGTGAGTAATGCCGTGCAAATGTCCAGTCAAAACTCCAGCAGCTTCCCCTGTGGACAAACAACGTCTGTCCCAGACCTTGCATAGATTATCATCGCTTCCAGAATAAATGAGATTACCACTTTCATCAGCGAATGCTACCGTGTTGACATCAGATGTATGAGCGGGCAAACGTAATGTCAGTCTGTCCGCCTGAAGGTCGTAGACATATATTGAATCATCATTACTGCCAGCGACAAGCTCTCGACCATCAGTGGAGAACTTAACAGAGAACATTCCAAACGAATATCTAACATCTTCATGCTGCGAAAAATCTAAAACATCATGGATGTCAGTGATATTAGCATATGATTCTCTTGAAGCAGTCCCAATATTGACAATATGGATAATAGGTGCCAGGCTAGAGTAGACAAGAAACCGTTGATCAGGTGATAATGATGCATCACTAATTGTCCATCTCAAATTTCTAGCATGAATGTCTCTGTG

>Isogroup_221 GAGGTACAGATCATTGATGATGTTTTTTGCATGATGTATAGCATTCTTCAATTGCACAAGAAAAATAGTAATCATTTTCAATCCTCTTTTTCAGGTCACGGTCTTTGCCATTGCTATCTTTAGCCATATCAACAGCTATAGGGACCATGCTGGCAAGCAAAAATGGAGGCCACTGCACAACACGAAGACCCTTATCAGCCACATATGGAACAAGTAGTAGCTCCTTCTCCCTGTTGTCTATAAGGTCTTCATCTCGAAAACTAGTGACAATTTCATTCCACATCTGTGCAAATCTTGCAGCTACCTTCTCCTTGTCTTCGTGTTTCATCTCCTTAAAACGTTTTTGTAAATTAGACTTTAGACCTTTCTTCCTTCTTGCATCAGATGTTTCAGACGGAACTAGACAATCATGCACAGCTAAAGGTATTGAATCAAAGCGAGATCGCAGCATTCCAAGGGTGCGGATCTCTCCAAGCCGTTGGAAAGCACCATAAATTCCACCAAATAGAGTGGAGAAGATTGTATACCAAATTTGCATGTCCATGAAATACACCAGAATGATTGGGACCCAAAGTGCAATCACGACACCAATGTTGCCCTTCGCTCTGGGAAAAAACTCATGCCACTGGAAATGATTTATAGGGAATTTCATGATATCCTTGGTAGGGTCAACAAGAGGCTTGATCTCGACATAGTAGCTAAATACAAACTTTGTCAAAAGAAGAACAACCCAAAACATGGTCGTACTCG

>Isogroup_222 CTCACCCACGGTTTTTGGTTGATGGCTTTGAGGTTGCCAAGAAAGCAACTCTTGAATTTCTTGAGACATTTAAGACGCCAGTCGTTATGGGTGATCAACCTGATATGGACATCTTGAAAATGGTAGCAAGAACAACTCTTAGGACAAAGCTGTATGAAGGATTGGCTGATCAGTTGACTGATATTGTTGTAAACGCGGTTCTCTGTGTCCGCCAAAGTGATGAACCAATTGATCTTTTTATGGTGGAAATAATGCATATGCGCCACAAATTCGACGTGGACACGCGTCTGATTGAGGGTCTGGTCCTTGACCATGGTTCTCGGCACCCTGACATGAAGCGCAGGGCAGAGAATTGCTACATCCTGACAGCTAATGTATCACTGGAATATGAGAAAAGTGAAATAAATGCAGGATTCTTCTACTCAAACGCAGAACAAAGGGAAAAGATGGTTTCTGCAGAGAGACGTCAAGTCGATGAACGTGTGAATAAGATTATTGAATTGAAAAATAAGGTTTGCTCAGGAACTGACAAGAATTTTGTGGTGATCAACCAAAAGGGCATCGATCCTCCATCCCTTGATCTTCTAGCTAGAGCAGGGATTATTGGTCTCCGCAGAGCGAAGAGGAGGAACATGGAGAGACTTGTGCTAGCCTGTGGTGGTGAAGCCATCAATTCAGTCGATGACTTGACAGAAGACTGTCTTGGCTGGGCAGGGCTTGTCTATGAGCATGTTCTTGGAGAAGAAAAG

>Isogroup_223 TGCTGCCTTTTCGCTGCTTCCCTCATCCTCTCCTTCCTCCTCCGCCAGTTTGCAGCGCCTCTCCTCCAGCACATCCCCTGGATAAATACGTTTGATGTAACACCACCAGAAGAATGGTTTCAAATGAATGCTGTTCTTCGTGTCAGCTTGGGCAATTTCTTGTTTTTTGCAATATTTGCTCTTGCGATGATTGGTGTAAAAGACCAGAATGATCGGCGAGATGCATGGCACCATGGTGGGTGGATTGGAAAGTTTGCTGTTTGGGCTGTTCTCATTGTTCTTATGTTCTTTGTTCCGAATGTTGTCATTACTGTTTATGAGATACTGTCAAAATTTGGATCTGGCTTGTTCCTTCTGGTTCAAGTTGTGATGCTTTTGGACTTCACAAATAATTGGAATGACTCGTGGGTTGAGAAGGATGACAAAAAGTGGGAAATAGCTTTGCTGGTAGTGACTGTGGTTTGCTATCTTGCTACATTTGCTTTCTCTGGAGTGCTGTTCATGTGGTTCAATCCCTCCAGTCACGATTGTGGCCTCAACGTGTTCTTTATTGTGATGACAATTATCCTTGCTTTTGCATTTGCAATAACTGCTCTTCACCCCCAGGTCAACGGAAGTGTTATGCCAGCTTCTGTCGTATCTGTTTACAGTGCATACTTGTGTTACACTAGTCTGTCGAGTGAACCAGATGACTATGTGTGCAACGGGCTTCACATGCACTCTAAGCAAGTTTCGATGAGCGCCCTTTT

>Isogroup_224 CCGACCACACCACGACTAGCGTGGTCGCGGCCGAGGTGTGGCGAAAGTCTTGCAAGATGGAAGGCTTTTGGATTTGCCTTTGTCAACAGCATTTTATAAGCTCATACTCGGACAAGAGCTTGATTTGTTCGACATCATCTCGTTTGATGCCGAGCTTGGAAAGACACTGCAAGAACTACAAATTCTTGTTGAGCGTAAGAGGTTCCTCGAATCCACTTCTGGCAAGGATCAGGTAGAAGTTGAAGACTTGCGTTTCCGTGGTGCTCGTATCGAAGACCTGTGTTTGAATTTTACTCTCCCAGGGTTTCCTGATTATGTTCTTAAAGAAGGCGAACAAAACACAACTGTTAATATCCACAACCTGGAAGATTATGTTGCTTCGGTAGTGGATGCGACAGTGAATTCAGGGATAATGAAGCAGGTTGAAGCGTTCAGATCAGGTTTTAGCCAGGTCTTTGATATATCATCCCTCCAAATATTTTCACCTCAAGAGCTTGACTATCTAATATGTGGTCGTCAAGAAATTTGGGAGCCGGAATCGCTGGTGGATAACATAAAGTTTGATCATGGGTATACTGCTAAAAGTCCAGCAATTGTAAATCTGCTTGAGATCATGGCAGAATTTACTCCTGATCAGCAGCATGCATTCTGCCAGTTTGTAACCGGTGCGTCTCGGCTTCCAACTGGTGGTTTAGCTGCCCTTAGCCCCAAGCTTACTATAGTTCGAAAGCACCCCCCGAGCGGGGTCA

>Isogroup_**225** CAGACACTTCTGTTCAGTGCTACTCTACCAGAGTGGGTAAAGAAGCTCTCAATGAGGTTCCTGAAAGCTGACAAGAAGACTGTTGATCTTGTTGGCAACGAGAAAATGAAAGCTAGTGCATCTGTCAGGCATCTCGCTCTTCCTTGTAACCGTGCTGCAAGGCAACGAGTTATTCCAGACATCATTCGATGCTATAGCCATGGAGGGCGAACCATTATTTTCACCGAGACAAAAGATTCTGCATCAGAGCTTGCTGGCTTGATTCCTGGATCCCGTGCCTTGCATGGAGATATCGCGCAAGCTCAACGTGAAGTAGTTATTGCTGGATTCAGAAGCGGGAAGTTTCTTGTTTTGGTTGCTACAAATGTGGCAGCTCGAGGCCTTGATATTAATGATGTGCAGCTTATCATTCAGTGTGAGCCTCCAAAGGATGTTGAAGCTTACATCCACCGGTCAGGTCGGACAGGGAGAGCAGGGAATACTGGCATTGCGGTCATGCTTTTTGAACCCAGATATAAATTCGGTTTGACCAGAATAGAGAGGGAGTCTGGGGTGAAGTTTGAACATGTGTCTGCGCCGCAGCCTAGCGATGTGGCACAATCTGCTGGAAATGAAGCAGCTGAAGCCATTGCAAGTGTGTCTGACAGTGTTATTCCTGTCTTCCGGCAACAAGCAGAGGAGCTGTTAAGCTCTTCCAGCATGTCCCCAGTTGATCTACTAGCCAAAGCCCTTGCAAAGGCAGTTGGG

>Isogroup_226 TATCCTCGCCCTCGCCGCCGAGATCTCCGTCGTGCTGTCCGGAGGACCTGGGTGGAACGTGTTCCTAGGGAGGCTAGACAGCAAGACCTCCAACATCGCCAGCGCTCAGAACTTACCAGGCCCCAACGACGGCCTCAAGAATCTCACACTCAAGTTCAACGCCGTCGGGCTTGACACTACCGACCTCGTCGCCCTCTCAGGTGCCCACACTTTCGGTCGCGTGCAATGTCGGTTCGTCACGGACCGTCTCTACAACTTCAACGGGACAAACCAGCCTGACCCGTCCCTCAACCCGGCTTACCGGGCGTTCCTCATCCAAAGATGCCCACAGGGAGGCAATGGATCATCTCTAAATGACCTCGACCCGACGACACCCAACACCTTTGACAAGAACTACTACACGAATCTCGAGGCACATCGCGGCTTTCTTCCATCTGACCAAGAGCTCAAGTCAGACCCCCTTGCATCGACAACGACGGCGCCGATCGTTGATAGGTTTGCTGGTAGTCAGGACGCCTTCTTTAAGACCTTCGCGAATTCTATGATCAGGATGGGGAACATTCAGCCGCTGACGGATCCCTCTGTGGGAGAAATCCGGAAGCACTGCGCATTTGTCAACTGAAGTTGGCGAGAAGATTACGTGTGGATATACAAGTGTGGCATGTGCTATATCTGTAACCATGGATGTAAGAGATAAATTTATCTCTTATTTTCCTAGTTCAATAATTGGCATATATCTCGAGCC

>Isogroup_227 TTGGAGCAACAAACAGAGCAGATGTTTTGGATCCTGCTCTTCGGCGCCCAGGCAGATTTGACCGTGTAGTTATGGTTGAAGCTCCTGATAAGGTTGGAAGAGAATCTATTCTAAAAGTTCATGTGAACAGAAAAGAACTTCCGCTTGGTAAAGATGTAGATCTCAGTGGCATTGCTGCAATGACAACTGGTTTTACAGGGGCAGACCTTGCAAATTTAGTAAATGAAGCTGCTTTGTTGGCTGGGAGATCAAATAAACAAATAGTGGAAAGGATCGACTTCATCAGTGCGGTTGAGCGTTCAATAGCTGGTATAGAGAAGAAACATGTAAAGCTGAAGGGCAATGAAAAGGCTGTTGTTGCACGGCATGAAGTTGGTCATGCAGTTGTAGGAACTGCTGTAGCAAAGCTGTTGCCTGGGCAGCCACGTGTGGAGAAATTAAGTATACTGCCAAGGTCAGGAGGTGCATTAGGCTTTACATACACTCCTCCCACAACAGAAGATAGATATTTGCTCTTTGTCGATGAACTGCGAGGACGTCTAGTAACACTTCTTGGTGGACGGGCTGCAGAGGAAATAGTTCTAGCAGGACGGGTTTCTACTGGGGCACTTGATGATATAAGACGTGCAACTGACATGGCTTACAAAGCTGTAGCAGAGTATGGTCTTAATGAGCGTATTGGTCCAATATCACTAGGTACCTCGGCCGCGACCACGCTAGTCGTGGTGTGGTCGGCGTC

>Isogroup_228 CGTTTTAAGGTAACTAAAGGAGGATCTGGTTCTGATGGAGCGCAGAAGAGGAAATCTGATACATTCAATGATGACAATAATCCACAGAGGCGTCTTCCTTCTGGTGGGTTCCTCTCGGATTCTCTAGATTTGGACCATAACCTTCAAAAACAGCTAAATCCTGGTTGCCATGCTGAAATCCTGTCACAGGACAGTGGCATAAGGGGCTGTTGGTTTAGATGCTTAATCATAAAGAGGCATAGAGATAAGATCAAGGTGCGATACCAAGATCTTATGAATGCTGAGGATACAGGAAATCTGGAGGAGTGGGTATTGCTAACAAGGATTGCTAAACCTGATCAGTTGGGTATTCGCATCTCTGGAAGACCAATGGTTCGGCCACGCCATACGATGCAACATAGCAAGGATCCATGCTCCCTTGATGTTGGTGCTGTTGTTGATGCATGGTGGAATAGTGGCTGGTGGGAGGGTATTGTGTTGCAGCGTGGAGATGATGGACGCCTTCAAGTTTATTTTCCAGGAGAAAAGCGGGTATCTGATTTTGGCATAGCGGATTTGAGGCAATCATTTGAGTGGGTTGGTGGCAGATGGAATCCCTTGAAGGAAAGAAAAGACATGATGAGCCACTTGCCTCCAGCTCCAGAATGTGAAGATAGAGGCTTTAGCAAACTCGCCCCCCTAAAGGATAATCCATCTCCAGTACCTCGGCCGCGACCACGCTAGTCGTGGTGTGGTCGGC

>Isogroup_229 CGCCGACCACACCACGACTAGCGTGGCGCGGCCAGGTACATCATGACACCGAGAAGAGCAACAACAAATATAAGAATGTATAACGAGTTCATGCTGACAAGAGAGTGCATGAAACCCATGAAGGATGGACCGCTGACTTGTTTCTTCGTTGTTCTTCCTGCTCTATATCCCTCAAGGAATCGGCTTACTTGGGATGCTTGATCACCATCTTCTAACCTTTCTGAACCTTCCACTTGTTCATACTCTTCATTTCTGTCCCACACTAGTAGCTTTGGCAATTGTGAGCTTTTGGAAATATCAAAAGTCTCCACAAACTCCTCCCATTGTTTGACTCCAACATATCCAAACACCAAGTCACGGTTCGCATTAGCAGCAGACCTCAACACTTTTACCAGCTGAGTAGAGTTTGCATCTGAGTCATCCTCCAGAATTGCGAGAACAACTTTTCTGTCATCGTCATCCAACAATTTCAGTGTCTCGGTGTTGATGGGGACAGTCAAAGGCAGCAGTGATTGCTTTATAAAATCTTCTAAGAAATGTCCTTCAAATGGGCCATAGAATACAGTTTGTTCTTTATACTTCGGATGAACTGCAACCAGTGCTGGAGCCTTAACAAAATCATATGTCACCATTAAGTCCTCGGAATAATCTTTTGTGACGGCAAACCATGCTCTTTTCTTGTATTTTCCTCCATATTCAGCAATCAATGACTCATTGACCCCAAAACCAAGGAAG

>Isogroup_230 CGCGGCCGAGGTACAGACAATCGATATGAATCAGGATAATTATTTGGAAGAGGCTATGAAGATGAGGAATTTACTTGAAGAATTCCGTGGTAGCCATGGAATACGTTATCCCACTATTCTTGGTGTGCGGGAACATGTGTTTACAGGAAGTGTGTCTTCGCTGGCGTCATTTATGTCGAAACAAGAAACTAGTTTTGTTACTCTGGGACAACGTGTTCTTGCTTACCTCAAGGTCCGAATGCACTATGGACATCCTGATGTTTTTGATCGGATATTTCATATAACCAGAGGTGGCATTAGCAAGGCTTCCCGGGTGATTAATATAAGTGAAGATATATATGCTGGATTCAATTCAACTCTCCGCCAGGGAAATATCACACACCATGAATATATCCAGGTTGGGAAAGGACGGGATGTTGGTTTGAACCAGATTGCACTGTTTGAGGGGAAAGTTGCTGGTGGTAATGGCGAGCAAGTTCTCAGTCGAGATGTGTATCGCCTAGGGCAGCTGTTTGACTTCTTTAGGATGCTAACCTTCTTTTACACCACTGTTGGGTTTTACGTCTGCACAATGATGACTGTTTGGACTGTATATATCTTCTTGTATGGGAGGGTGTATCTGGCACTCTCTGGACTCGACTATTCAATATCTCGCAAAGCTAGGTTTCTGGGAAACACTGCCCTTGATGCTGCTCTGAATGCTCAGTTTCTGGTCCAGATTGGTATTTTTACAGCCG

>Isogroup_231 CGCCGACCACACCACGACTAGCGTGGCGCGGCCGAGGTACAGGTGGAGACATGTCCCAGCCAAGGTTCATTCACAATGAATCTGGCCGCTTTGAGTGCCGGTTTACCAGTGTGGCCATAGGGGATTCTCCTGCTATAATGTTCAGAGGTATGGAAGGCTCTACCTTGGGCATTTGGGCTGCTCATGGTGAAGGGAGAGCCTTATTCCCTGATGAAAATATTTTATCCGGTATTGTTAACTCTAATCTGGCCCCTCTGCGGTATTGTGATGATGCTAACAATGCCACGGAGGTCTATCCTTTCAACCCTAATGGTTCTCCTCTTGGTATTGCGGCTCTTTGCTCCCCAAATGGAAGGCACCTTGCTATGATGCCACACCCAGAGCGTTCCTTCATGATGTGGCAGTATCCATGGTATCCGAAGGAATGGCAGGTTGAGAAGGGTGGCCCGAGTCCTTGGTTGCGCATGTTCCAGAATGCACGAGAATGGTGTGCATAAGCAGAAAAAATGGTGGTTGCTGGTGCTACTTATTATCCTACTTGCAGAGACCTGGATGGATATATTCTCTTCGGGAGTTAAGAGTGGATAACCCAATAATCTCTCTTCAGTTTGAGCTTCTCCCACCATGATTTGCAGCACTACTAGGGCTATGGCCATAATTTGCAGCACTACTAGGGCTATGGCCATAATTTGCGGGAATCATAGGGTTATTTTCGGACCTGCAGAGACAGTACCTG

>Isogroup_**232** TCCTGAGTAGTTGCCTGCTTAAGCGCAACGGCCAAGCCAGTGATAGTATGGTTGTGAGGCCCACCTTGCAGACCCGGGAAGACAGCAGCATTGATCTTGTCCTCAAAATCATACTTAACCTCTTTCCCTTGCTTGTTTATTTCTTTCACACCCTTCCTGAAAAAGATCATGGCTCCACGTGGTCCACGGAGTGACTTGTGAGTAGTGGTAGTAACGATATCTGCATATTCAAAAGGAGATGGTATGACACCAGCAGCAACTAGGCCACTAATATGTGCCATGTCTGTGAGAAGTATTGCCTTCTGCTTGTTACAGATCTTGCGCATGCGGTCGTAATCATAAAGGCGAGCATACGCACTTGCACCAGCAACAATCAACTTTGGCCTAAAAGAACAGCACTTTTCTCCAACTGGTCATAATCAATCAAACCAGTGCTTTCATCCAGTCTGTAAGGCATTGTCTCAAAGAATATTGAAGTTGCTGAGATTTTCTTTGCGTCAGTCTGGTAACCATGAGAAAGATGTCCACCGTGAGGAAGATCCAGAGCCATGATTCCATCATGTGGCTTCAGCACAGCAGTGTATACTTGGAAGTTCGCAGGTGAACCCGATAGTGGTTGCACATTCACTCCCCACTTCTCTGGGTCCAAATTGAAAGCCTCCAGAGCACGTTTCTGACACAACGATTCTGCCATATCAATGTATTCGTTTCCACCATAGTATCTTGCGCCAGG

>Isogroup_233 CGACCACACCACGACTAGCGGCCGCCCGGGCAGGTCTCTCTCACTAGCACGGACCATAATCTTCTCAGAAACTTCCTGGAAGACTTGTTGGTTTCCTTGCCTTTCCCTCGTCGCAGCGCCTCTGATCTTCCCGCGCGGCATCCAGATCGCTGGCATCTAGTATTGGGATTTCAATAAGAAGGAAAAAAGATACAAGTAATGCCAGAATATGTTCTCGCGTGCTAGAGGGTCATTTTGCTTGGAAATTTGTAGGATAGCTCTTGAAGACGCTGAACCACAAGAGTTGCTCCATGGGCATATGCAGGATGGGCCTAGACGTAGCAGCTCAACCATCTACCATCTTGGTAGGGACGGTTGAAACAATTGGTCATCTCTAGGCTCCTTCCACCTTATTGATCTGTCCTCACCACCTCAAGTTACAGCACACTGCCTTCTGTGTCTGCAACACATTGTGCGTCAAGATGGGAGAGCAGAAAGGAAATATTCTGATGCAGAAATATGAGATAGGAAGATTACTTGGGCAAGGTAGCTTTGCTAAAGTTTACTATGGCCGTAATCTCAAGACTTCACTGAGTGTTGCCATCAAGGTGATCGACAAGGAGAAGATCTTCAAGTGCGGGCTTATGGATCAAGTAAGGCGGGAGATATCGGTGATGAAGCTGGTGAAACATCCAAACATTGTCCAATTGTACCTCGGCCGCGACCACGCTAGTCGTGGTGTGGTCGGCGTCTC

>Isogroup_234 CCAATTATGCTGACTAACGTAGGTGGAGTCCCACCTAAAAGAAGTTTCTTAACTTCACTGGCATTTCTAGATGCCTCCAGCTGAAGCTCAAAAGTGCTACTGCTCTTTCTGTTGCCAGTAGCCTCATCGGCACTCCCTGATTGGTGCTTGTCAGATTGACTTGCACCTGCATTTCCTGGAAGTCCATCCAACTTGGTGAAGAATACACCATTAGGCCAGAGGGTATCTTGAATCCAACGGATTCCTTGTATAATGACCTCATCTCTTCGAAGCCAATTGATCTGTCTTATAATCCAATCATCGATCGCATCTTCCATGACTAACTGCAAGATTTGTTTTGATATCCAAAGAACTTGTTTTCTTATCCAACCACGTCGCTTTAGTTGGAACACCTTGTCTACCAGATTCAGTAAAGACTCGCTCACATTTGTTGGCATCCACTCGGGAGGAATTCCTGAAGGATCCTCAAATGGTTTTACACAACTCTTGAATTCTTCTCTGATTGTCTTCTGTATAAGAGCATCAGTTCGAACATCAACTGCTGCTGTTGCCTCATCCAGAACAAGTATCTTTGCCCTTCGTAGCAATGCACGAGCTAAACTCAGCAGCTGTCGCTGTCCAACGCTAAAATTTTCACCAGCCTCAGAAACCTCAGCATCTAGTCCTAGAGCATTCCTCCTTATGACATCCTTTAGATGAGCCCTTTCAAGAGCCTCCCATAAATCTGCATCGT

>Isogroup_235 AGCGGCGCCCGGGCAGGTACCCAGCAATAGGTGATCGAGGAAACCAAGTGCAAGCTCAGTTTCCACATCCAGTGGCTCCTCTGACAAGAGCCAATTAGTGCACACCATATATTTCTTTTTCAAATCACCTTCTTGACCAGCAGGATACTTCTCTGTGATCCTCACTGGTTTTTTGAAAAGCTTCTGTGGCATAACCTTCGATTCGTTACGAGCAGGGCTGGCTTCAAACAGGTCAAGATACTCACTTAGAATCCGTAATCGCTCTTTTGTGTCATCATCACCGTAGAACCAGATCCTAGCATTGCTCGGATGATAAAACATACTGTGGAACTCCTTGAACTCTTCGAAAGTTAGCTTTGGAATTTCATTTGGGTGACCACCACTATCCACACCATATGTGTTATCAGGGGAAAGTGCCTGTTGAGTTATACGCCCCATTATGTTATCTGGTTGAGAGTAAACTCCTTTCATCTCGTTGAAGACAACGCCTTTGTAGGATATCTCTTCTTCGGGACTGTTAAGCTCATAATGCCAACCTTCCTGTTGAAACGTTTGAAAGTCCTCAACACACTTGGGGAAAAAGACAGCATCAAGGTATACATCTACCAAGTTATAGAAATCCTTGGTGTTTGTTGATGCAACTGGATAACATGTCCGATCCGGATAGGTGAATGCATTCAGGAAAGTATGCAAACTACCCTTCAATAGCTCAACAAATGGCTCTTTCAATGA

>Isogroup_236 AGGTACCATAGGGATCAGCATCATGAGAACAACTTATCTCCTATCAGTTAATGCCCTCCTCAAGTCCATCAGAGCATATCCCCCAGATCTCAATTTTCAATTTGTCCAAGATTTGCAGCATACGCACACTCAGTGTAAGGTGGGTTAAGCGGTTAGACTGAGAGCGGTGCTATATCTGGCTTCAGGCCTTTGACGGCAACTTGTTCATCACAGGAATGATCTTGTCCTCCTGGAGGCCCCTGATAGGATCAATCTTGTAGGTATCGTAGAAGTCCAAGATTTCCATAGAAATATTCTTGACAATGTTCATGTCAGCACGGAGTTCTTCGAACCAGGAAGTTGTGTCCTTATCTTTAAGAACACTTGCGATGTATATGCAGGCCAATGCAATCATATAGGGAGGATATATGAGAATAAGATCCATCTTGTAAGTATCATTGACAAGGCCCCAGGCGAATTGAGTCAGATCTGTTATGCCAGCATCCTGCAATAACTGTAAAATGCACATCTCTCACATCATTGTAGAATATCAAAGGGTAATAGTCGAGCTAAGCACCGGAAGATATAAAGTGTTATTCAAGTGGAAATGCCATTTCATTCAGATGATAGGGGAAGCAAAGTCATACTGTAAAAGAGGACGGTATGGATGGTAAACAACCAAATAATAGTCGAGTGCTTCCAGGAGCTTCATTTCCATTTCAAGGATATCTTTGATCTCAAATCT

>Isogroup_**237** CTGCGACCAAGTTCCTCTGCAGTCACTCCATGCATATCCCTTAAGTAACCCTCCGCGATCCACCTCCTCACCAAGCGACCCAAAATAATTCTGTGGTCTTCAGGAAATATGGAGAGGTATAAAAAAACAGACTTGAGATGGTATGGCAAACCATCATAGCTCCTCATAAGAATTGTCTTTATTATCCTAAGTTTAGGGTTTATCTCCAATTCAACACTAATACTATCATTCATCTTCCTCCATTCAATAGCAGTTTTTGGCTTAGTGGATAAAAATCCACCTATAGTTGATATTGCAAGGGGAAGACCATGACATTTCTTTAGGATAACTCTTGCTTGCTCCATCATGGCACCTAAATCATTTTTCTTAGTTTTGTCCTTGAATACCTTCTTTAAGAAAAGGTCAAATGCAGCATCATCTTCCAAACCTTTAATACTATACATGATTCCGTAATCTCCTGAACAATGTTCGGCAATAATTTTTTCTCTTGTGGTGACTATTATTGTTCCAGCATTATCCAAACACCCTTTGACCAGCTCCCATTCAGAAGTGGATGATATATCATCAAGAACGACAAGGCACTTTTGTGATTTTAGTAGCCGAGCTAACTCCTCCTTCAACGCTTCAAGTTCCATCACTGCTATGCTTTTCTTTTGCACTCCTGTGGTTGTTGCTCCAGCAGGATCTTCTTGAATACTTTTCTGAAGTTGGAAGGCCAAACCTCTAAGGA

>Isogroup_238 TCTGTTCTCAACTTTTGTCACATTAGTGTATTCTCTGAAGAAATCCATAAGCACTTCTTCATCTAGCCTCATCCTCTCAACCCTTTCTTCTTTTATGTAGTTTTTCTGACTGAGAAGATGATCAACATATACAACTATTGTTTCCTCTAGGCAAGACTCCACAAATCTTCGAAATGATCTTTCTTCAACATATAGTTTCACGTCGCCAAAATAATCAGCAAATGTTGCAACCAGATATTCGGTCACCATTCCGTCCATCCAGTCTTTTTGGTAAACCTTTGCAAGCAAATCCTGCACACCTGGGTCTTCGAAAATAACACCAACAGTCTGAAGGACCGCTTCCTTGGCAACCTCAAGAAATCCTTTGCAAGTGTCTTCAAAATTAACCTGTTCAGCATAGTTCTCCGAAAGTGCTTCCAGTGTGCTGCTACTTAGCTCTGATGAAAGTTCGTAGCACCGCAGGTTGTTGTTAATCAAAGCACACAGATTTTCCAAACCGACATCAGAAGCTGGCTCCTCAAGTCTTTGCCTCTCAGCTGCTTGAAAATCAAGCATGACCTGAATAACTGCAAGAGCTATACGATATAGCATGACATCTGTGCTATTTTCTCTAACTATCTGCACTTGCTCTGTCAATATTCTGAATAAATCTACAGCTGCAGGTGTATATAGTTTTCCATCTTCTGTACCTCGGCCGCGACCACGCTAGTCGTGGTGTGGTCGGCGTCT

>Isogroup_239 AGGTACTTACTGAAGCTGGTGCGCCGTGTGGGATAAATTCAGTGTGCCTCTATGGTGGAACTTCAAAAGGACCACAAATATCTGCCCTTAAATCTGGAGTTGAAATTGTCATAGGGACACCTGGTCGTATGAAAGATCTCATTGAAATGGGTGTTTGTCGTCTTCATGAGGTTTCTTTTGTGGTTCTAGATGAAGCAGACCGGATGCTGGATATGGGTTTTGAACCTGAAGTCAGGGCGTTTTTAAGCCAAACATCATCGGTGCGTCAGATGGTTATGTTTAGTGCAACATGGCCTTTTACTGTCCACCAGCTAGCCCAAGAGTTTATGGATCCAAATCCAATAAAGGTTGTTGTTGGATCGGAAGATCTTGCAGCTAACCATGACGTGATGCAAATTGTTGAAGTATTGGATGATAGAGCACGAGATTCAAGATTAGTTGCTTTGCTCGACAAATATCATCGAGCACAGAGCAACAGGGTTTTAGTTTTTGTGTTGTATAAGAAAGAAGCTGGGCGAGTAGAAGCAATGCTTAATAAAAGGGGGTGGAAAGCCGTTTCTGTTCATGGAGACAAGGCTCAGCATGATAGAACAAAAGCCTTATCTTTATTTAAAGAAGGAAAATGTCCTTTAATGATTGCGACGGATGCGGCTTCACGCGGACTTGATATTCCTGATGTCGAAGTTGTCATTAATTATAGCTTTCCTTTGACGACGGAGGACTATG

>Isogroup_240 TGGACCGGATGCGTGGTTACCGCCGGTGAGATGCTCGTCTTGGAGGGGATGTAGTTGTTGCTGCCGACCTTCCTGCGCCACCAATTCAACCCAGTATGGACATATATTTGAGATCTCTAGCTGCAGTTGGCAAGTAGTGGCTACATGTGTATACATGGTCTTGTTCTGTACCTGCCCGGGCGGCCGCTCGATAGCGTGGTCGCGGCCGAGTACCGCTAGCTTCTGCCGAGTAGTAGCTCTCGTTAATTCGAGGAAAGTCCACAGCAGACACCGAAAGTTGCTTCTGTGAAGCAGCACCGGTTTTCATGTTGAATCGCATTTCATACAGCTCGTTTCCAAAGTTCTCAAGCTTTTCATCCTGGTGACCGTTCACCATGTCCAAATCTGGGTTCTCGAGGCGGCAGGTAATGAGAACAACTTCGTCGCCCTCTTCCCAAGCATTAGCATTGTGGAATATGAAGCAATTGGGGAGTTCAAACCATCTGATGCTTTTTTCGTCCTTCTCATAGCGTTGGAGTATACCGAAACGAGCTTTCTTTGTAGGATCAAACTTGTAGATAAATTCACCATTCTTCACCATTTCCTTTGGTCGAAACAACAAAGGCAGGTCCATGAATATGGAATAATTCTCTGTGATGGCAAAGTCATGCATCATTACAGATTCTGGTATTGTTATTGGCACAGGATCAAGCATAACTCCCTCCTTGGTGATGACCCGGTAT

>Isogroup_241 GGTACTATGCACAAGTTACACTTCGTGATTTTCCAAAGACAAAGGCAGTAGGTCCCTTCGCCCGTCAATCATACCATAGGGAAACACCAGCAGCTGATGAAGCTTCCCATAGACCACCATTTTAGCTTTTTATTCACAGAGCCTCAACATGTAGAGCAAACAGCAAAGTCTTCTTTATGGCTCGGGACTTCTAACTTTGATCTCTATTAGTTTCTCAATAGGCTGTCTGCATATGGGGCATTTATCTGACTGGAGACTTAGAGCTTGTGCACATCCACTGCACATACACAAATGCCTACAAGGAAAAACAGCTGTGTCCCTTGGCTCGGTCAAGCAGATGACGCACTCTTTCCCCATGCCATCATCATCGGCGTCAGGAACATCAGCTTCCGTGGAGTTGACAATCCCAAATATTTCTTTTAGCTCATAGCGCACTCCATCAATCCACAATATTTGCTTGATAACTTTGACTTGAAGGTCATCATTATGTTTCGCTATAACAGCAAGAGTGATCTGTGCCCGAGTGGAGTTTTGTGGCTGACCAGCTTCCTCGGGTGGTGGACAAGCTTCTGCGTAGACTACCAGTGGAAATACTTCATCTGAAGGATTTGAAAGCTCATCCAGAGAAAAGAATCCCAAATCAATACCAGATCCAGAGGGCTGGACATAATTTTGAGCCAATCCTTTCTGGAAAGGTATCTTTGTTGGTGTCTGTAAGTCAGGG

>Isogroup_242 GGTACACATCATTCACACAACGACGATATGGATGTTGTGGCTTGAAGATGGTAGCACGCGACAACTTCAAATTAGCCCAGAACTCCTCTGATGGTGAACCACAAAGCTTGAATATTTTGTGCAACTGTTCTACCTCAGTTCGTCCTGGCATGATAGGCTTCCCTGATAGCAACTCCGCAAGGATGCAACCAGCACTCCAGAGATCAACTGTGGCACCATAGTTAGTAGCACCCAGCAAAAGTTCTGGAGGCCGATACCATAAAGTTACAACACGACTCGTCAAATTCTGCTTTTGGTTTGGATTAAAGAATGTAGCTAGACCAAAATCTGCTATTTTGAGGACACCATTATTGTCTAGCAGAAGATTGGCACCCTTTATATCCCGATGCAAAACACCACGACTATGACAGTGGTCAAGTCCAGAAAGTAGCTGCTGCATGTAACACTTGACCTGAGCCTCTGTGAATTTTATGCCAGGTGTAGCTGCAAGTCCCGCAAGATCATGCTCCATGTATTCGAATACAAGATACAAGCTACTAGACATGCGCGATGTGACCAAACCTTCAAGCTTAATAACATTTGGATGATCAAGTCTTCTTAAGATGTGAATTTCCCTCGCCATAAATCGAACACTCTCAGGATCCATATTGGCAAACCGAACTTTCTTTAGTGCCACGAATTTTCCATTCTCAAGATCTCGTGCTTTGTAAACGCTACTGTAT

>Isogroup_**243** TACAAGCACACGCCCAAAGGCTCACTCTATGATCAGTTACACGAAGAGGGAAAAGATTGTAAGATGGATTGGCCTCTGAGGTTAAGAATTGGCATTGGTGCAGCAAAGGGCCTTGCATATCTTCACCACACATGCAATCCTCGAATCCTTCACCGTAATATAAGTTCCAAATGCATCCTCTTGGATGAAGACTATGAACCAAGGATTTCGGATTTTGGGCTTGCTAGGCTTATGAATCCCTTAGACACCCATCTCAGCACCTTTGTCAATGGAGAATTTGGAGATATTGGTTATGTGGCACCAGAATATGGGAGCACTCTGGTGGCCACCCCAAAGGGTGATGTCTACAGCTTTGGTGTGGTTCTGCTCGAGCTCGTCACAGGTGAGAGGCCTACTCAGGTTTCCACTGCTCCAGACAACTTCAGAGGAAATCTAGTAGAGTGGATCACTTACCTATCAAACAATGCGATTCTCCAAGACTCAATCGACAAGTCGCTGATAGGAAAGGACGCTGATAGTGAGCTGATGCAGTTCCTGAAAGTCGCATGTTCTTGCACAGTCCCCACTGCGAAAGAGAGACCCACCATGTTTGAGGTTTATCAGCTCCTCAGAGCCATTGGGGAGAAGTATCATTTCTCGGCCGGGGACGACTTCATTCTGCCACCTCTAACCACAGATGGAGAAACCCCGGACGAGCTCATCGTTGCCATGTAATTACAAAACC

>Isogroup_244 CACCAAGAGCAAGAGGAACTGCTCGTCTAATATTCTGCTCGCCATACTGTAGAAGACGCTCAAGTGCTCGGATAGCCATTTCAGATCCCAGTTCTTCAGACATGGCAATAAGAGCAATTCCAAGGACAGCTGGCCCTTGGTGTGTTTCACCTTTATCAAGATGTTGTGAGCAAATACCAAGAAGTTTCTGAACCTTAAGCACATTCCCTGTTCCAGCATACGCAAGCGACATAAGTGTTACATCACAGTATTTCCTGATCTTCTCATCAAATGTTTTAGCAACCTCTGCAGTAGCCTCGACACTTTCCTGTTTCCCAAGATACAGAAGGCCAAGTGCAACTGGAAGCAGGCGTATAATCGGTTCTGCCAGCTCGGCCTCACTACGCTCCATCAAGAAAAATATGATAGATTGTGCAATCTCCTCATTACAAGAACCAACAAACACCAATCCCAAGGCAACAGCTGTGAAGACCAAGACCTCGAGTGTTGCTTGGGGGTCTCCCAATATAGTGGATAGCTGCGCTCTTAGCTCATCTTTCTGAGAACCAGCATATGCAATACCAAGACCTAAGATTGCCCCAATCCGTATGTTCGAGTCTTCCTTGTTAACATACTCCATGAGAATAGCCATTGCAGGGTCGCAATCATTCTTCACACCGCAGGTGACAACACCAATACCTAGCAAAGCTCCAGCAACAACATGAGTATCATTACTATGCAA

>Isogroup_245 TGACCGTATCCAGCATAGACCCCGACAGCGATGAGACTAAGGATGACAAATCTATACCAAGCTTCTTCATGTAGCTGAGCAAACAAAAACATATTGAAGAAAACAGAGACCACCGGGACCATAGGAACTCCAGGACAAGAAAAGCGAGGTGCTTCCACAGGAACATAAACCTGTCGAAATTGCAGAGCGAAACTGGCAACAATAGCTATCAGAAAAGCTACTATGATGAAGGCAACAGCGTAGCTAAACCGGTAGGATATTCCTGCTATAAAACCACACAGAGCCACTATGACAAGGCACAAAACACCCTCCTGCCAGATTGACATACTTCCAAGGGAGCGAGAATTAGTTGCTTTGTCGCTCCACCGAAGTGTGATCACCCAAGCTGATACAACTGAGTATCCTGTCAGTGTGCCAACTGAGAGAATATGAGAGAGTGCTTGCACATTAAAGAGGCCAGCTAAGACTGCTGCAACACAGCCAACCCAGATCTGAGAGTGCAGAGGAGTATGCAGCGTTGGGTGAACTTTAGCAAATATTGAAGGTAGTAGGCCATCCCTTCCAAGCCCAAGATACAAACGTGACTGAACATACAAACCAACAAGAAGTGTCGTAGTAAGACCAGCAACAGCACCAATACTGATCAACACAGTAACAAATTTCAACCCCTTAGCAGAAAAGGCCTCAGCCAAGGGAGCATCTTCACCGAGTAATGTATATG

>Isogroup_246 GGGCAGGTACTCCTGTCACAAATGCCTTCTGCAGCTGGCCACTGAGCCCAGTTTTCCATGGACCTATTGTTGCCACCGTCTTGTGGCGGCATAGCAAAAGCATGCATGCAATGACAACAAGCAACAGAGCAACTGATGGAATCACAATTGCATATAACCACTTTATAGATCTTCCTTTTGACCCTGCTTCGGAAGGAGTATCAGAAGACTTGGGTGGACTAATTTGAGGATTTATAGCAGGTGCAGGTGCTCTGATGGGGCTCAATGCTGAAAATGAGCCACTTCCAGTAGAAGGAACAGGAACAACAGCTGGAATAGGAGGATTTGGAGAAGGAGCAGCAGCAAGATTGATGTCCTGAAGTAGTCTGCGCCTTCTAAGCTGTGCAGCTGGGTTTCTTGTGGCAAATTGCGTAGGAAGACGAGAGGCGAGATTGATGGTATCTATTTTGTTCTCTTCTGTCGAAGAACCAGACTTGAACCCAGTATCAGCTCTTTGGTTTGCATCAACCCTCCCGAGATGATCATAGATGTCGAAATCAATTTCCTGATCAAAATTCCCATAGGGATTTTGGATTACAGGCCATTGAAATCTGTTGTTGGAAAGCAATAAATGCTTAAGTGATGGCATTTCGGCTATCTCCTGTGGAACTTCCCCATTCAAGTTGTTATTGCTTAAGTCCAGTACCTCGGCCGCGACCACGCTAGTCGTGGTGTGGTCGGC

>Isogroup_247 GAGTACTCTGCTGGACCTACAGAGCGTCAACTGACGCTAGATGAGGCGTTACAGGACAAGCAGACCACCGTTGTCTCTGATGAAAAGTCACCTCCTCCTCAGAAACTGAAGCTGCTCTCAGGTTCTTGTTACCTGCCTCACCCTGCTAAGGAGGCAACTGGAGGCGAGGATGGTCATTTTATTTGCATTGATGAACAGGCAATTGGCGTGGCAGATGGTGTTGGTGGTTGGGCAGATCACGGTGTTGATGCTGGGCTATATGCAAAGGAGCTAATGAGTAAATCAATTGGTGCTATCAAGGATGAACCGGAAGGTGCAATTGACCCGTCAAGAGTTTTGGAGAAGGCCTTTACAAGCACCAAAGCAAGGGGATCATCAACTGCTTGCATCATCACTCTTAAAGAACAGGGTCTTCATGCAGTAAATCTCGGGGACAGTGGCTTCATAGTAGTCAGAGATGGCCACACTGTTCTCAGATCACCTTCACAGCAGCATGATTTTAATTTTACCTACCAGCTCGAGAGTGGGGGTGGCAGTGATCTTCCTAGTTCTGCGGACGTATTTCATTATCCTGTTGCTCATGGTGATGTTATTGTTGCTGGCACCGATGGGCTTTTTGACAATCTGTTCAACAATGAAATTATTGCGGTTGTTGTGGAGGCTCAGAGGTCCGGGCTTGGGGCTCAAGCCACAGCACAGAAAATTGCCGCCCTCGCTCGG

>Isogroup_248 AGTGGCTCGCCAAGGAGATCGATCAACAATACAGCTTTAAATATCACCTTCAAATAAAACTCGCAAGTAACGCATGCAATGCAATCCAGTATAACAAAACGCCAAGTCTCAAGGTCGGTTGTCTCATGAGCAATCTATCACCAGATACCCACATCCCATCAACTCTGTCCGATATAGGTCGAGTGAACTTTGGAACAATGTGTGACATCGTCAGTGACTTGAAACCATGTGCTTTATCATCATCTTCATCATCTAATGGTTTGTCCTTGTGCATCTCTATATCAATAGCAACTTGGTCGTTGTTGACTTTTCGTGCCCCTCGAATCTGAGTGCCAACTGCACTTGGGCTTACTTTGTCATTCATTTGTTCGATCTCTGAAGTGCCTGGTAAGTTTAGAGATCTGCTAGGTTGGCCCGATATCCTGTCTCGACGAAGCAACCCTATCTGAGAATCCTTTTGGCTCAAAGAAGCTTCTAGAGCCTCTGATTTACGCGACAACTCTTCCATTTTGGTCCTCTCGACCTGTAGTGAAGAATCTTTGCTTTGGACAGCAATCCTCAAATCATCTGATTCAGATATCAGAGATCCTATTTTCTTGTCCAACATATCCATATATACAGGAACCTGAGTGCAATCATGATCATCTGTGCTCTCACAATGCACACCAAACTGCAATAGTGCAGTTTCTAACCACTTAACAAAATCATTTATCTGAGATGA

>Isogroup_249 GAGAGACGCCGACCACACCACGACTAGCGTGGTCGCGGCCGAGGTACAAGGAACAAGGTATTGAACTTGTTCTTGGAACAAGAGTGATATCAGCTGATGTGAGACGGAAGACATTGCTTACAGCCACTGGAGAAACTATCAGCTATAAGACGCTTATAATTGCAACTGGTGCTCGGGCTTTGAAGCTGGAAGAGTTTGGAATTAACGGTTCAGATGCTGCAAACATATGTTATTTACGCAATCTTGAAGATGCAGATAAGTTAGTGAATGCGATGAACTCATGTTCTGGTGGAAATGCTGTTGTCATTGGTGGTGGCTACATAGGAATGGAATGTGCAGCAGCATTGGTTACTAATAAGATTAAAGTGACCATGGTCTTCCCTGAAAAACACTGCATGGGTCGGCTATTTACGGAAAAAATTGCAGAATATTATGAAAACTACTACACTTCAAAAGGAGTTACTTTTACCAAAGGAACTGTGCTTACATCCTTTGAAAAAGACTCAACAGGGAAGGTGACTTCAGTAATCCTAAAAGATGGTAACCATCTTCCTGCTGATATGGTAGTTGTTGGTATTGGGATCCGCGCAAATACCAGCCTTTTTGAAGGTCAGCTGTTGATGGAGAAAGGTGGCGTAAAGGTAAACGGGCAAATGCAAACTAGCGACAGCTCTGTGTACCTGGCCGCGACCACGCTAGTCGTGGTGTGGTCGGCGTCT

>Isogroup_250 ATCTAGCTCTTGCTCAAGGCTTTCTAGCTGAGTCTTCTGTCGAAATCCACCAGTTGCGACCATAGACCTGAAGGGCACCCCAGATCGTGATATTGCACGGCAATTCTGAAGGACCTGAGAAGAAAGTTCAGCGGTAGGTGTCAATATTACGACTCGTGGGTTCCTGGGGGATGATTTGACTCCCATAGCTTCTTCCTTCCTTAAGTTTTGAACTATAGGGCAAAGATATGCCAATGTCTTGCCAGATCCACTTTGATCAGCAATAATGCAACTCCTCCCCTCCAAGACAGGACCGTATGCCATAGCCTGAATATGTGATGGTTGAGGGAACCCGAAACTACTCAAGGCACCTAGAATCTCATCGCCACAGTCAATATCCTTAAAAGAGCTGCGACTGAAAAAACCCACTGTCTAAAGGTAACTTTTGCCTCCGATCCTCTCTTCTGTAAGCCGTGGAATCGGCGACTTTACCCCAAACTTTGGGGCCAGAAGTATTCTTTTCATCCTGAAATTCCGTATCATCATCGTCACCTTTGTATTGCCTCAGAACAGAATTCAGAGAAGGCGAGCTCCGGGTTCTTTCATCCCCATAGTTACTATTTGGTCTATCCCTGCGTGCTGGGTCTGAAACGAACTTTCTTCTTCCAGAACTTTTTTCATCTTCATCAGAATCAGCAAAATATTCACTACGCGACGGGGTGGTTGTGCGCCTAGAACTAG

>Isogroup_251 ACCGGGTTGCTTGCAACCGGCCTCACAGCTGATGCAAGGTCCTTGGTCTCCCAAGCAAGAAACGAAGCAGCTGAGTTTCGTAAAAAATGGGGCTATGAGATGCCTGTGGATGTATTAGCGAAATGGATAGCAGACAAAGCACAAATATACACGCAACATGCTTACATGAGACCCCTTGGTGTAGTTGCTATGGTCCTGGGCTATGACGAAGAGAAGAATGCCCAACTCTTCAAGTGTGACCCCGCAGGTCACTTCTTTGGACATAAGGCAACTAGTGCCGGACTTAAGGAGCAGGAAGCAATAAATTTTCTGGAGAAGAAAATGAAGGATAGCCCTCAGTTCACATATGACGAAACTGTTCAGATTGCGATTTCTGCACTGCAGTCAGTTCTGCAGGAAGATTTCAAGGCTACCGAGATTGAGGTTGGTGTTGTGAGGAAAGAAGACCTTGTTTTCAGAGCACTCAAGACGGAGGAGATCGATCAGCACCTAACAGCCATAAGTGAGCGTGACTGAGTGGCCATGAAATTCACTGTTCTGTAGTGCTTTGGCACTTGCTGTTCTGTTTAATAGCCGGCCAAGAAACATTCGGTGGATCATTTCATGTGTAGCAAAAAAGAAACGGGCATACACTTGCCATGTATTAGTTGGATCGATCATCCTGTGTGGTTTAATTTGCTACGATTTAATTTGATGGTGCTGGCAGTTGCATGTTTGAG

>Isogroup_252 CTGAAGATGAGGAGGAGATTGAAGAAGATGCTGCTGATGAGCTTCAGGGTCAAATGGAGCATGATTATGATATTGGATCTACCATAAGGGACAAGATCATCCCTCATGCTGTTTCCTGGTTTACTGGTGAGGCTGTGCAAGCCGAAGATTTTGAGGATATGGAAGGTGATGAGGATGACGAGGATGATGATGAGGATGATGATGACGAGGATGATGATGAGGAAGAAGATGAGGATGAGGATGACGACGATGAAGAAGAACTAAGCAAACCCACGAAGAAGGCTGGCAAACCTAAGGGAGCTGCAAAGGGTGGTGCACAAGGAGAGCAGCCAACAGAGTGCAAGCAGCAGTAGGACTTATCTCCGCCAGGTTGATGCAGTATTTCCCATTGAACTGCCCAGGGGGACATTTGCTGGTTGTGTTGTGCACGGGTGAGATCTTATTGTGGATATACCGTAGGCATGGTTTTATGAGTGGCATGGCGATTGTGCTGGTGGATGGTTTGGGAGCTTGGGATGATGTTGGCGTGTTATTCTCTAGTTATATGCAGTCGAAGGACACCCTAAGATTGTGTAATTATATTTGAGCTCTTCATATAGAGCTCTTAGTATTGTGACATGATCTTGCGTTGATTATTTTTTGAGTTGGGCGCTCGTGGATGTCTGGAGAAAATTATTGTTTGGATCCCTGTTGGTAATAATGGGTTTCTGTTTGAGG

>Isogroup_253 TCATCTCTTGGAGATGCTGGAGGAATCGAAAAGCTTGCTTCTCTAGCGAAAGAGCACGGAAAAAACAATGTTGCTTTCCTCTGCCATTTTATGCTTGGTAAATTGGAACATTGCATACAGTTGCTTGTAGACAGCAATCGTATACCTGAAGCTGCATTAATGGCACGTTCTTATCTTCCCAGCAAAGTTTCAGAGATTGTAGCAATATGGAGAAAGGACCTCAGTAAAGTTAATCCTAAAGCTGCAGATTCTCTAGCAGATCCTGCTGAGTATCCAAATTTATTTGAAGACTGGCAGGTTGCACTTGCTGTAAAAAAGAATGTTGCTGCCCAAAGGGGGCACTATCTTTCTGCTGATGAATACTTGAACCATGCTGAGAAGTCAGACGTTACTCTTGTGGAAGCTTTCAAAAGGATGCAGGTCATTGAAGATGAGGAACCAGTAGATGCACTGGATGAAAATGGAGAACCTGATGAAGAGGTAATGGAAGAGAATAAAACGGAGGAGGACACAGATGAAGCTGTCCAAGTTGATACTGATGAACCTGAAGAAACTGTTCTTGTAAATGGGAATGAGGGTGAGGAACAGTGGGGTATGAACAATGAAGGAACTTCGCCAGCCTAAAAGAATTGTTTGGCAAATTACTAACCCAACATGATGAGTAGGCATCGGTGATGGTACCTCGGCCGCGACCACGCTAGTCGTGGTGTGGTC

>Isogroup_254 CGCCGACCACACCACGACTAGCGTGGTCGCGGCCGAGGTACCACATAGTGAGCATATGACCTGCTGCACTTCATGACGTGAAAGTTCATGCCGCCTAATCACATCAGTTTTAATCGAATTCTTTGATTCATTGTGGCAGTGCCGACAATCAAAAATCTCGTTGCAGCATGGTGCCCGTATGCGGCATCTCCTTCGGTAATGTGCACACCCATACTGCATCAATCCCCTCTCCAGCCTCTCATGATCATCAGGGCTAGAGCCATTGATCCCAGCTGAGTCCTTTCCATCAACTAAAGGGCCTTGGGGAAATACCTCCACATCTAGCTTAGCTTGTGCATGTTGAGAAGCAACAGACTCGAGTTGCACGGCGCCCATGTAGGCAGTTTTGTGCCACTAAAGCTATCACGTTGGAAACACAACACAATACCTGCAAGTATTATTCATTTAGCAGGATGGCTCTCAATACAGAATGCAGTCACAAGTCCAGAAATGTAGGGAACCACTCCATGCATTTATGGTTGATGTATACTCTGGTGAAGACCAGGTATATCAACACAAGGCCAAAGCAACGCCGAACAGCTTCCAATCAGCAAAAACCCCATGTTTTGTTCACCAAATAACCACAGCTAGTATAAACTAGCTAGCTGGATAGTGAGCTGTGAAAGAGCAAATTACACAACTGACATCCATCCAGAGAAGAATCTCCAAGCAATTCGT

>Isogroup_**255** GAGACGCCGACCACACCACGACTAGCGTGGTCGCGGCCGAGGTACCGATCGAAATAAAGAAGCTGCTTCATGAGTAGAGCGAATTCCCTGGGAAATTTCAGCCCATATGACTCACTAATCCGCACCAGATCAAGAAACATTGCATTCATCTGCCTCTCGTCTACAACAACACTAGCTGATACAGCTGTAGCATTAGAACTTCTTGCTGTTGCGACTATGATTTCACTATCCAACTCCTGTAGTGATGAGAATATCTTTTGTAAGTCCTTGGTAAAAGCATCAATATCTATATCATTCCCTGTAGCACCCATTTCGGAAAGGGCAGATGCCATAGCCTTGTAATCCTCAGTTGCGAAAGAAGCCAAAAAGATCTCCATAGCAGCCCAAGTTCTTGGGGATATTCGGCCCACAATTCCAAAATCAATAAATCCAACACGTCCATCACGAAGCAACCATAGATTTCCTGCATGCACATCTGCATGGAAGGATTCACATGAAATCAAGCTTCCAAACCAGACATTGAGGGCAGTGACTAAAGTCAACTCAGGATCAGGAACAAGAGACCTTATAGAATCAAGATCAGTCAGTGGAACGCCATACAATCTTTCCATAGTTAAGACACGCTTTGTGCTACAGTGCCGGTACACAAATGGAGACTTGGCCTGCCTATCGAATCCCATTGCATCGATGTATCTCTGGAAAGCCTCCATATTTAC

>Isogroup_256 GCGGCCGCCCGGCGAGTACGTGAGATAAGTATCCCGCAGAGGTGATAAGCTTCTGTAGGATGATAAAGCGACGCTTGTCTTTACCGTGGTTTTGTTGCTGCCGCTGGAAGTTGGAAACATATCCCTAGTAATGCCAGACCTGGAGCTCCTAACTGACGCTTGATCTTTGACCCAAGGATGTTCTACTAATTGAGCAGCAGTTGGACGTGCAGCAGGATCACGCTGCAAGCAGAGTTTCAGAAAGCTTTTTGCTTCAGAAGAAAGATGATCTGGGATATCAGGTATGTCTTTGCTGTTTCCAATTTTGAATATTGCAGCCACCCCTTCATACTGACTCCAAGGAGGCCTTGCAGTTGCCATCTCAAGAATGGTGCAGCCAAGGCTCCAAATGTCCACTGAAAGGCTATATCCATTGGTATTCATGATAACCTCTGGAGCCATCCAATAGGGGCTTCCTTTGAAGGATTTGATAGATGTGTATGCTGATATATGCTTGGCCATGCCGAAATCTGCAAGTTTGATGTCACCATTAGGATCTACAAGTATATTTGCTCCTTTGATATCCCTGTGCACTGTTTTCCGCCCATGCAAGTATGCAAGGCCAGAAAGGATCTGTGCAGAGTAATTCCGGAGGACTGCCTCCCCAAATGGACCATATTCTTGAAGCAGCTTATGTACCTCGGCCGCGACCACGCTAGTCGTGGTGTGGTCGGCG

>Isogroup_**257** AGCCGTGGTCACCAAAAACAATGCTGCTTTTTGGACCGATGGCCGGTATTTTCTCCAGGCTGAGAAGGAACTGAGCCATGTCTGGACCCTAATGCGTAGTGGAAATCATGGTGTTCCAACCACTATTGAGTGGTTGAATGATGTCTTACCATCCGGTTGTCGAGTTGGTATTGACCCGTTTCTTTTCTCATCTGATGCAGCTGAGGAATTGAAGGATGCTATTTCTGACAAGAATCATGAGTTAATTTTGATTAGAGATTTAAACCTGGTTGATGAGATATGGGGAGAATCAAGGCCAGAGCCCCCAAAAGAACCCACTAGGGTGCATGCCATCAAATATGCTGGTATTGATGTGCCATCAAAGTTATCTTTTGTTAGATCACAACTTGCTGAAAATGGGTCTGACGCTGTGGTTATTTCAATGCTTGATGAGGTCGCATGGTTGTTGAACATGAGAGGAAGTGATGTTCCACATTCACCTGTGTTCTACAGCTACTTGATTGTGGAGATGAACACAGCTACATTATTTGTAGATAGCAACAAAGTATCTGAAGAGGTTTTGGAGCACCTCAAGAAAGCTGGAGTTAAGTTGAAACCATATGAAGAAATTTTATCTGATGTAGAAAGGTTGGCAGAAAAGGGTGCAAAACTGTGGTTGGATTCTTCAAGTGTGAATGCTGCCATAGTCAGTGTATTTAGATCTGGTAGCGACAG

>Isogroup_258 CTTCTTGTATCTTGGATAAATGCATGGATTTGTGGTCCAAGTTTGGGTATATCCCTCGGCTCCATCTTGGAATGAAATTTACTTCAATCAAGCTTCTAGATCCAATACATATTGAGAAGATGTGGCATAGGTATAAAATTGAGGACGGCCTTATTGTTCAAGAGGTGTCACAAGATTCGCATGCTGAGAAACTTGGAATCTGCCTTGGTGATATTATTGAACGTTTTAATGGAGAATGCATATCTACTACAGTTCAGTTGGAAAACATGTTGCTGGGCAGATGCAGGGACCATTTAGATCAAGGAAATCACTTGAATGAGCAAATAGATGTTTCAATTCAAGTATTTCACACGGAGGAACGTCTCCGAAGAAATATAAATTTGATTGTAGATGTATCAGATGGTGGAGAGGTCGTTAAAAGAAGGACTTGCCCCATCACTGTTATAGAAGGGACATCTGCTTCAGGGCAATCTAGCCAAAATTTGGCAGGAAATTTCTGGGAGGCCCACCTCTTTGAATTAAGAGACGCAGCAGACATCTGCTTCCCCGCCTAGCGGCGAACCATCATTCTCTGGGATAATGAAGGAAGACAGGAGCGAGATCCCCGAAGCCTCATTTGCATCTTGCAGTGAGATGAAAGTAACTCTGGACGCGAGGCACTGATTTCTCATCATAGAGATGTAAAATAAGTTAGCATGCCTGCAGTGAGCGTGGA

>Isogroup_259 AGCACACATTCTAAGAACCTGGGGAAAGCAGACCAGCCTTCTCTTGACTTGAACTTAGAACATAGCAAGTATGCAAATTTGAACGATCAGCTCGCGGAAGCTAGTCGTCGACTTAGACAGATGAGAGGTGAGGAGCTTGAGGGGTTAAGTGTTGATGAACTCCAGCAGCTGGAGAAGAACCTTGAGACTGGTCTGCACAGGGTGCTTCAGACGAAAGATCAACAATTCTTGGAGCAGATCAATGAACTGCAGCGAAAGAGCTCACAGCTGGCAGAGGAGAACATGCAACTGAGGAACCAAGTATCCCAGATACCAATAGCTGGCAAGCCAGTAGTTGCTGATACCGAAAATGTAATTGCTGAGGATGGACAGTCCTCTGAATCTGTCATGACCGCATTGCACTCCGGAAGCTCGCAGGATAACGATGATGGTTCGGATGTATGCCTGAAATTGGCGTTGCCCTGCAGTGCATGGAAATAACAACATAAAATCCTGCGGAAAGCTCTTGTGTAATGGGCAAGCCCGAACTGCAATAATCTTGCAGCGGAAGCGAGATCAGTTAACCTGATTTATCATCTTGTGGCTGCATGACGTGTTCTTGTTTTTACCGTTTGCTAGGATGTTAACAAAACTTTTAGATCTATCTGATGTCCATTATTCATGGTATCCATGTACCTCGGCCGCGACCACGCTAGTCGTGGTGTGGTCGGCGT

>Isogroup_260 GAGACGCCGACCACACCACGACTAGCTGGTCGCGGCCGAGGTACCAGTCCTGCTTGCTTGGTTCTTTTCAAACTGTCGAGCTAATTGAAACATTTATGAACTATCAGGAGAACATCCGGAGATGTGTGTGCATTGTGTATGACCCATCTAGGTCTAGTCAAGGAGTGTTAGCTCTCAAAGCTTTGAAGCTTACAGACTCATTTATGGATCTTTATCGTAACAATGGTTTAACTGGAGAGAAGCTCAGGGAAAAGAAATTATCATGGGTTGATATTTTCGAGGAGATACCGATTAAAGTGTCGAACTCCGCGCTTGTCAGTGCCTTCATGAAAGAGCTGGAACCTGAGTCACCTGTTTCACAGTGTGACTTTGACCGTCTCAAATTGTCGACTGCTCCCTTTATGGAAAGAAACTTGGAATTTATGATTGGGTGCATGGACGGTCTTTCATCAGAGCAAAACAAGTTCCAATATTATTATCGCAATCTAGGAAGGCAACAGACACAGCAGCAGGCATGGCTTCAAAAGAGAAGGCAAGAGAACATTGCGAGAAAAAATGCTGGCGAGGAGCCATTGCCAGAAGAAGACCCATCCAACCCTATCTTCAAGCCACTTCCTGAGCCATCACGTTTAGAGGGTTATCTCGTAACCAACCAGATCTCCAGTTACTGCAACCATATCAATGGGGTTGCTGGCCAGAATTTCAACAGACTG

>Isogroup_261 ATTTTCCCTTGATCACCTTGCCACTTTCAGTTTGATCTGATACAGTTGGTTCACTTGGATTCTGGAAATTTTCCACATTGTTCACATGCAACTTGTTAACTTCAGTTTCCCCTTGACGATTCTCAGAATCTGATTTAGTCTCTAGTTCCAAAGGTATGGGAGCTTCAGTATTTTCCATGATTATAGTGTCGGTAGAAGAATCTTTCTTGGAGAGGAGTGTGTCATCTTCATCTTGTTTCCCATTGAAGTCTTGAGGGTTGAATGAATTGTCACCACTCTCCTCATTCCTTGATTGCTCAATGCATTCAGCTGTCTCGTGAAGCTGGGCTGCCTTAGAGTCATCCAACTGTAACTTTTCCTTTACGCTGTCGATTTCTGTGTGTTCTGATACACTTGCATCTGCAGTATGTAAATCTTGCTTGGCAACTTCTGTTTGTCCATTGTAATGAGAGGAGTCTTCTGATTCAAGAGATCTCCATGGTTCATTCATTATCACAGTCCCGGTGGCAGAATCTTTCTCTGAAGGGAGTGTGTCATCTTCATCCGGTTTTCCATTGATGCTTTGAGGGTTGAGTGAACTGTTTCCACTCTCCTCCTCGGGCTTTGATTGGTCAGCACATTCATCTGTCTCATGAAGCTGGGCTATCTTAGACCTATCAAATGGAAACTTTAGCTGATTGTACCTGCCCGGACCTGCCCGAGCGGCCGCTCGA

>Isogroup_262 GGCCGAGGTACCCAACTGCTGCCAGACATGGCGCATAGTGCATTTAGCTTATTGTGGTTTGTTTGGTGAAATTCATGGACCAAGTATGTTGGTGACGGTATATGAACCAATGGCCACTGTTAATATGTGTAGAAGGATTTCGTGATCGATTTGAGTATTAATTGGAGCAGTGCAAGCATGCACTTCATACAAGAACCATCAATTTGCTTGTCATGGTTTGGATGATTAATTTACCAATAGAATGGTTTAAAATCTGGAAAAGTAATGTAAGATCTAATGGTCCCTACCAAATTAGTTGAATCGAAGGTCCTTTTCCAAGATGAATTCAGCTTAAGATTTAGAACTGGCTGTGTGATGAGGGTATCTGGACCCTTGAAAACAAGCTCAGTTCTTATGGTGTTGAGAGAATGCATTTGCAGATAGTCATGATGAGAATTGAAAGGAGAAAAAGGATATTCAAACATAGATCAATTGATGGGGCTGGAAGAAGATGCATGCGCACATAAACAGAACAAAAGGGCACACCAATTTCATGGAGGAACCTTACCAATATCACACATCTGGTATCTATTTAAACGGGCCATGTTTCTACCTGGCATGCTATGACCACCTCCACCTGACTTGACGATATTTCTGGGTCGTCCGAAGCTGATAGCTAGCATTACACCTGTGTCTTAGACATCAAATTATGTTACTTTGTTTATAGCATTGT

>Isogroup_263 CCGACCACACCACGACAGCGTGGTCGCGGCCGAGGTACTTGTCGCTTAAGGTGGTGCTGTCCGGGTTCTCTGAGAAGACCACCTGCGTGCTGGGGTCGACGGTGGACTTGATCGCCGAGAGGATTGTCGTCCCAACAGTGTTGTTGTTGCCAGTCAATCCTTGCCATTCGATTGTCCATCCTCCACACTGGTTGCCCAAGTTGTCGGCGTGGCTTCCGGCGACGAGGATCTTACCAGCCTTCTTTGGGAGAGGCAACAACGGAGTGGAGGCAGATTTTCCATTTTTCAGCAACACCAATGATTTCCTGACGGCTTCCCGAGCGAGTTCTCGGTGTTCTTGCTTTCCGAGTTCACCAGCAAGACTGGGATCAGCATAAGGGCTCTCAAATAGACCCATGGTGAACTTGACCCGAAGAATCCTGTAAACAGCATCGTCGATTCTGCTCATGGGGATAACACCGCTCTTAACTTGGGCTGTCAGATCATCAATGAAATCTGTGTAGGCATAAGGAATCATGATCATGTCAATACCAGCACCAACTCCTGCCTCAACTGAATACGAATAGTTCAGGCCTGGGGGAGTAGTAAGCCTATCAATGCCTTGCCAGTCTGTAATCACAAAACCCCTAAATTTGAGCTTGTTCTTGAGAATATCAGTGATTAGGAAATGGTTGGCATGCATCTTATTTCCATTCCAACTAGAGTACCTG

>Isogroup_**264** CTTGAGGGACCAGGAAGAGCCTCAAGCCAGCCATCACTTGATACTGATACTGCTGAATCATCCAGTTGCCAGATTCCTTCTCATGTTACTGGTCAGAGTAAATTCAGGACTCAGTTGGTTAACTTTGACAAAGCGTGGTGCAGTTATCTTTACCGCTTTGTGGTGTGGAAGGTAAAAGATGCAAGATCACTGGAGGGTGATCTTGTTAGGGCTGCTTGCAAGCTTGAGTTATCAATGATGCAAACATGCAAGTTAACTGTGGATGGGCAGTCAGACAACCTCACCCATGATATGAAGGCGATTCAGAAGCAGGTCACTGACGACCAAAAACTCCTAAGAGAGAAGGTTCAGCATCTGAGTGGTGATGCAGGCATTGAGCGTATGAACTCTGCTCTCTCAGACATGAGGTCGAAATTCTTCGAAGCAAAGGAGAATGGAAGTCCATTGGCAGCACCTGTTGCAAATGTGTCGACTCCTCTGAGTATTAATCCGTGTGGCCAGCTCCCACCTTCTGACGTTAGTGTGAGTTCCAAGACAGATGCAGAAGGATCAAAGTCTGTTGTCCGATCGCTGTTTGGAGCTTCTTCATCGAGCAGCACATCATCAGTCAGTCTGCCAACAGAGAATGAGCAAAAGGTCAATGAGATACTTCATGAGAACGGTGGTACCTCGGCCGCGACCACGCTAGTCGTGGTGTGGTCGGCGTCTC

>Isogroup_265 ATAGAAGAGCTACATTCAGCATCGTCAGTGCTTCTTGTTGAGAATAGTCAGAACAAAGACGCTGGTCGACAATTTCCAGAAGTCTCCCTTGCTGCTTCAGTCTTTCGGCCAAATCAAGAAGATGGAGATACTCTTCACTTGACATGCTATTTGTATTGCTCTCCCCGCTAACAATCTCTAAAGTCACCACACCAAAACTGTAGACATCTGCTTTACGTGTCAAACAACCTCTTGTAGCATATTCAGGAGCCATATATCCAACAGTGCCAGCAATACGAGTGCTCACACGCCCACAGTCATCATTCAGCTTGGCCAAACCAAAATCAGATATTTTAGGTTGTAGTCCTTCGTCAAGAAGAATATTTGACGGTTTGATATCTCTGTGGACGATTTTCAGTGTTGATTCCTCATGAAGATAGGCCAGACCTTTCGCCGTTCCAAGGCAGATGTTATACCTTGTTGGCCAGTCTAATTTCAACTGACGTTCTGCGCGGCCAAATAGTGCACGACCAAGACTGTTATTTTCCAGGAATTCGTATATCAGTAATAGTTGTTCACCATCAATACAACAACCATAGAGCCTCACAAGGTTTGGATGCCTTAGAGAAGATATTATTCCTATCTCATTTAGAAACTCACGGTTCCCTTGGCTGGATTTCGAGGATAATTTCTTGACAGCAACTGTAGTACCTCGCCGGGCGGCCGCT

>Isogroup_266 ATGATTCCCACAAGATCACCAACTTTGCTGATAACCTCCAAGCCTTTAATAAGATCCTCCCTGGATTGGGAAGCTGAGATACATATCCTAGCTCTGGCAAGTAGCAGTGGCGTAGCAGGAAATGCAACAGTAACAACAGCAACATTTTGCCGCAGGCACTCCCTTGAGAAAGCAGGGATTTTAGCAGGGTTGTAAAGCATGATGGGCATGACAGGTGAGTCATTGTCTCCTAGCACCTCAAAACCCATTTTCTGAAGCTCTGAACGGAAAAAATTGCTGTTCTCCCGAATCTGAGCAAGTTTCTTGGCCCCTCTGTTAGATCCATCTTCCCCAAGGACAACTTTTATCGCGGAGATGACCTGCTGGACTGCTGGAGGTGACATGGATGTTGCATATATATGGGCTGGGCATGCATGCTTGAGATGGTCAATGATCTCTTTTGATGCTGCAATGTAACCTCCGCACGATCCAAATGACTTTGTAAATGTGCCCATCATAATATCTACATCAGCTGGATCCACTCCCAGTAGTTCACATACACCTCTCCCTGTTTTTCCAACAGCTCCAATACTGTGTGCCTCATCTAAATATGTGTAAGCCTTATATTTCTTGCAGACAGCCATAATCTCAGGGAGGTTGCACAGCTCCCCTTCCATGCTATAAATTCCCTCAATAATCACAATTATCTTCTTCCATGGCCTGTGTG

>Isogroup_267 GAGACGCCGACCACACCACGACTAGCGTGGTCCGGCCGAGGTACAGCTTACAGGACACAGTGCTGGAATTGGAGTTCCAGGGCATCAAAAAGGAGTTCACTATGCTCCACACATGGCCTGTGCGGACACCAAGGCCTGTTGCGTCAAAACTTGCTGCGGATACGCCTCTTCTAACAGGACAGCGTGTGCTTGATGCTCTGTTTCCCTCGGTGCTTGGAGGAACATGTGCTATTCCTGGAGCTTTTGGTTGTGGAAAAACTGTCATTAGTCAGGCACTCTCAAAGTATTCCAATTCCGACACTGTGGTTTATGTGGGCTGTGGTGAAAGAGGAAACGAGATGGCTGAGGTCCTCATGGACTTCCCGCAATTGACAATGACACTGCCTGATGGACGTGAAGAGTCAGTCATGAAGAGAACAACACTTGTGGCTAACACCTCCAACATGCCTGTCGCTGCTCGTGAAGCCTCCATCTATACAGGAATTACGATTGCTGAATACTTCCGTGACATGGGCTATAATGTTAGTATGATGGCTGATTCCACGTCACGGTGGGCCGAAGCACTGCGTGAAATTTCTGGACGTTTGGCTGAAATGCCTGCAGATAGTGGTTACCCTGCATATTTGGCTTCACGTTTGGCATCTTTCTACGAACGTGCTGGGAAGGTGCAATGTCTTGGCAGTCCAGATCGTACCTGCCCGGG

>Isogroup_268 GCCGACCACACCACGACTAGCGTGGTCGCGGCCGAGGTACACAAACTGAGAGGCAAAGGCCTCGTTCAATTCAAAGAGATCAATGTCATCAATCTCGAGGCCAGCAGCCTTCACCGCAGCAGGTATTGCCACCGCAGGACCAACACCCATGACAGCTGGATCCACTCCAACAGCAACGAAGCTCCTGAAAACACCAAGTATAGGAAGCCCCTTGCTTGCTGCTACAGATCTCCTCATGAGAAGAACAGCTCCAGCACCATCACTCACTTGACTAGAATTGCCTGCTGTTGTAGTCCCATCCTTCTTAAAAACCGTTTTAAGCTTCGCCAGTCCAGATACTGTGGTCCCTGGCCTAATTCCATCATCAACTGATATCACAACTTTCTTTTCCTCTCCAGTTTTTGGGTCAATGATCTTCGTATGCACTGGAATAATCTCATCCTTAAACTTTCCAGAAGCTGTGGCTGCGGCAGCTCTCATATGAGACTCAGCAGCAGCCTTATCTTGCTCTTCTCTGGTTACACCGTATCGCTGAGCAACATTTTCAGATGTTATTCCCATGGGCAGAAGACAATCCTGTGCTTTCTGTAATTCAATTACCCTCGGGTTTACTTGGCCTTCCCAACCAACAGAATTTACCGACATGGATTCCAGACCAGCACCAATCCCTATGTCATAGAAACCAGCTTTAATAGCAGCACC

>Isogroup_**269** GCCGACCACACCACGACTAGCGTGGTCGCGGCCGAGGTACTGCAAACCTCTGAACTCAAGTGTTCCCTCAGGACTTTTAGGAGTATTCAGAGCTCCATGAATCACTTTCAAACTTAAACCATCTAATCTCCATGAACCTGATTCAAGGCCGATCCAGATTTCTTTGACTGTCTGCAATTTGGGACCTTTAAAGGTGATGACATCAACAGAACCACTTTGAAAGGGAAGGGATTGCAATGTCTTCGTGCCATTTGCAGGTTGGTCTCCATAGATAGCTGGAACTCTTTGTAACAAGGAGTCACCGTCAACATCGATTAAGCAGATTAAGATCGCGGCATTTTTATCTAGCAAAGAGGAGCTAAGTTCTCTACTAGTGCTAAGCTCTAGTATATAAAAGGCATCGGATTCTTCCAATCTGACTTTTGATAGTATCTCCTCAGGGACCCTATTGGGAAATGTTTTTGGTTCAACTGTTGGCAGAAGACGAGATGGCTTTACAGAGGCCACTGAATCTTGTAATGAAGAACTTTTGGCCAAAGTAATCAGTCGCTGATTTCTGTTTTGCTTTCTGAGTTTCCTTCCGGCTGAGTTCAGACCGTAAGGAATTTTGGAACTGTTCAGTGATAACCATCCAGATAGGAATTCAGAATGAACCGAAGGAGCAGGGTGGCCAGCACAAAGGATCACCATTTTGCGTACCTG

>Isogroup_270 CCCTAAGATACCCGTCTACAAAGTCGACATCGACTTGGATGGCATTGGAAACAAACTAGGTCCCCTGAAAATATTCTCTGTGCCAACGTTCCATCTGTATCACAATGGTGAGAAGACTAGCGAAGTTGTTGGTGCTGATGTAAACAAACTTCAAGCTGCCATGGAAAGTCTTCACAAGCAACAGTGAAGTTCTGTGTAAGATTGGAGGAGGGAGTGTGGGAAGGTTCTTTTGCATGAAGAGTTGAAGACGTTTGCAATCCTTCTGATTTGTATCAACGCATGTGAAAACTTCTGCTTTGCAATCTTGTTCAGACATCTTGTTATAGTTTGGTTGCGACATCGACATCTGGATGTTTTGCCCTTTTTTAGCACAGGTTTTTGTGGGAGATGGGTGCAACATGGAGGGTATCTTGAATGAAGCTTGCTCATTGGCTGGGCATTGGGGTCTCGGCAAGCTGATTACTTTCTATGATGACAACCACATTTCCATTGACGGAGACACAGAGATTGCTTTCACAGAGGATGTGAGTGCCCGCTTTGAAGCTCTTGGGTGGCACACAATCTGGGTTAAGAACGGCAACAATGGCTATGACGAGATCCGTAAAGCCATTCAGGAAGCTAAATCCGTTACCGACAAGCCCACTTTAATCAAGGTGACTACTACAATCGGTTTTGGATCCCCCAACAAGGCCAACTCATACAGT

>Isogroup_271 AAGTATCAGTTCAAACAGTATGCCTGGACGCACATGATCCTTTTAACGGTTTTTGCGCAATCTTCTTTCACGGTGGCAAATATATTTGAAGGGATGTTCTGGTTTCTTCTCCCTGCTTCACTTATTGTGATCAATGACATTGCTGCCTATTTATTTGGGTTCTTTCTCGGGCGAACACCATTGATCAAGTTATCTCCAAAGAAAACATGGGAAGGTTTTATTGGTGCATCAGTGACAACTATCATCTCTGCTTTTCTGTTAGCAAATGTAATGGGCCGCTTCCAGTGGTTTACATGCCCAAGAAAGGACCTGTCGACAGGGTGGCTTAATTGTGATCCTGGCCCTATGTTTAAGCCAGAGCATTATTTCTTGGGAGGTTGGGTGCCACAGTGGTTTCCATGGAAAGAAGTGTTCCTTTTGCCCGTGCAGTGGCATGCTTTAGCCCTTGGTTTGTTTGCATCGATAATTGCACCATTTGGTGGATTTTTCGCAAGTGGCTTCAAGAGGGCTTTTAAAATAAAGGACTTTGGTGACAGTATACCTGGCCATGGTGGAATTACTGACCGAATGGATTGTCAAATGGTTATGGCAGTTTTTGCATACATATACCACCAATCATTCATCTCACCCCACAATTTCTCTGTCGACGCAATCCTGGATCAGATCATAAGAAACCTGACCTACGAGGAGCAGAAGGCCTTG

>Isogroup_272 TCGAGCGGCCGCCCGGCGAGTACATGATTGGGGTTTATGATCTCAAACGTGATGCCTTTTCGCCGGATACTGTCCTAGATGACCGTCGGTTGTGGCTGAAGATAGATTACGGCAATTACTATGCTTCAAAATCGTTCTTCGACTCGAAAAACAGCAGGAGGGTCATATGGGGTTGGACTAACGAGTCAGATAGTTCTTCAGATGATGTTGCGAAAGGTTGGGCAGGCATCTATGCAATCCCCAGGGCAATTTGGTTAGACAGCGATGCAAAGCAGTTGCTGCAATGGCCAGTTGATGAGATTGAGTCCCTTCGTGGAAATGAAATCAACCATCAAGGACTAGAGCTGAACAAAGGAGATCTTTTTGAGATTAAGGGAATTGACACTACGCAGGCTGATGTAGAGATAGATTTTGAGCTGACGTCCATCGATGACGCCGACCCTTTTGATCCTTCCTGGCTTTTGGACGCTGAAAAGCACTGCCGGGAAGCGGATGCATCAGTTCATGGGGGTATAGGGCCATTTGGGCTTGTTATTCTGGCCTCTGACAACATGGAGGAACACACTGTTGTGCACTTTAGAGTCTACAAATCACAGCAGAAGCATATGATACTCATGTGCTCTGACCTAAGAAGGTCTTCCTTGAGACCAGAACTGTACCTCGGCCGCGACCACGCTAGTCGTGGTGTGGTCGGCGTCTCT

>Isogroup_**273** CGGCCGAGGTACTCCATCACTGACGCCTCGTTCTCCTCTTTAACCATCATGGACTTCAATCTAATAAACCATTCTTCGGTCAATGTCCCTGATAAGTTGTTGGAGGCCAAATCCAGAATCCTTAGCCTTGGAAAATCACAAGTATTTTTCTCGTCAGCAATATAAGATGACACTTGTCCAAATAACTTGTTAGATTTTAGGACGAGGACTTGAAGTCTGGGAAGTGCAGTCATCCAACATGGAAAACAGTCGCTAATTTGATTGTTTCCGATACCAAGGACCTCCAAGAATTTGCAGGAAGCTAGAGATCTGGGCAACTGCCCTTCAATCCGGTTGTCACTTAGCAGTAGTGCCTCAAACGTGCAGCTTTCGTTGATATTATGCGGCAACTCTCCATAAAGTTGGTTTTCTTTCAGATTTAGAACTTCCAGTGCATTGGCATCCTCCATTAGGCATGAAGGGATCGAACCACTGAGGTTATTGTAAGACAGATCAAGAATTTCTAGTGTTAGGCTACAGAAAGATGGTGGAATATTTCCTGAGATGTGGTTTGTTGATGCCTTGAAATAGTATGTATCATCAAGTTGAGTAGAAATATTAAACGGCATGGATGTGAAACGATTACTTGAGTAATCAAGCACCTCAGAAGTTTGGGGTATAGGTATTGGCCCCTCAAACATGTTAAAACTGAGATCCAAAA

>Isogroup_274 TAACAATGAAAAATTACTTAACGATGAGTTCTACATTGGGATTCGGCAAAGACGTGCCACTGGGGAGGAGTATCATGAGCTCATGGAAGAGTTCATGGATGCTGTTAAGCACATATATGGTGAAAAGGTTCTCATCCAGTTTGAAGACTTTGCCAATCACAATGCGTTTGATTTGCTCGCAAAATATAGCAAGAGCCATCTTGTTTTCAATGATGACATCCAGGGAACAGCATCTGTGGTCCTTGCAGGTCTGCTATCTTCACTGAAGGTTGTTGGTGGAACCCTAGCAGAGCACACTTATTTGTTCCTTGGTGCTGGAGAGGCTGGAACTGGTATTGCGGAGCTCATTGCTCTTGAGATTTCAAAACAGACAAATGCTCCAATCGAAGAGTGCCGCAAGAAAATTTGGTTGGTGGATTCAAAGGGTTTGATCGTTAACTCCAGAGAGAACTCCCTTCAGCCATTCAAAAAACCTTGGGCACATGATCATGAACCTTTGACAACCCTGTTAGATGCCGTCGAGTCCATCAAACCTACAGTGCTGATCGGAACTTCTGGAGTTGGCAGGACTTTCACAAAAGAAGTTGTTCAGGCTATGGCTTCTTTCAACGAGAGACCTATCATTTTCTCATTGTCAAATCCAACATCGCACTCTGAATGTACCTCGGCCGCGACCACGCTAGTCGTGGTGTGGTCGG

>Isogroup_275 CAGGTACCTGCCAGTGTAAAATCCTGCTGTATACCATGCATTTAATACAACAGTAAGATCACTGTCTGCTCCTGTGGCGTCGAAATTTGGGTGCATGCCCACAGTTGTGTTATTCTCCTTCCTAGTTTCACCTTCATTTCTTGGAAAGGAGCCAGGATCTCCAGATATTGTAGTTCTCATGAAATTCATGGCACCTTCAGCAGCTAGCTTTGCAGCTTTAGCAACTTGATCATCATTATTAGACACCTTCGCTGGGAGCTGCACGAAGGTTGCACCAGTAGGATATGTGGTGCTTGCTCCAGGGCATTGATCACAAGTCAATGATATGCTGCAGCCTCCAGTGGAATCACCGCTTTGCTGTCCAGTTGAACAACAGGAGACTGACACCATGGGGACATTCAAAGAGCAGCATGAGGGTTGAGTGGTTGAAGTATATGGATCAGGTGCTGTGGCACTATAGGAAGGAACTTGCTCCTGTTGGTATGCTGAAGATTGTCCAGGGATTTGATAATTCCAGTAATTTGTCTGATGGAGTTGCTCAAGAACTTCCCGGCTTTGCACCTCAAGTTCATAGTATTTCCTGAGTAATTCGTTATAGTCCGATGACTGCTGGTTTGAGCTATCCAGTTTCTCAGTATCATTAACATCTGAGGAGTACCTCGGCCGCGACCACGCTAGTCGTGGTGTGGTCGGCGTCTC

>Isogroup_276 ATGGCCAAGCTCGCCGAGCAGGCTGAGCGTTATGAGGAAATGGTAGAATTCATGGAGAAGGTGGCAAAGACCGCTGATGTTGGTGAGCTCACTGTTGAGGAGCGCAACCTGCTTTCTGTGGCTTACAAGAATGTGATTGGTGCCCGGAGGGCATCCTGGAGGATCATCTCATCCATTGAGCAGAAGGAGGAGAGCCGTGGCAACGAAGCTTATGTGGCTTCAATCAAGGAGTACCTCGGCCGCGACCACGCTAGAGCGGCGCCGGGCAGGTACGGCATGACTTGTCCAGGACAGTCTCACGGAAGGAGACAACAGGAGGGGAAACAATAATTTCAGCACCACCCATGAAGTCATCCTGCAGATCCTTCAAGCAAATCTCAAGGTGAAGCTCACCAGCTCCAGCAATGATATGCTCACCAGACTCCTCGATAGAGCAGAGGACCATAGGATCAGACTTGGCCAGCCGCTTCAAACCTTCAACAAGCTTGGGAAGATCAGAAGCCACCTTGCACTGAACAGCAACACGCACAACAGGGGACACAGAGAACTTCATTGCTCTGATTGGGCAGGCATCAACTTCCTTCTCATTTGTCAGGGTTGCGTTCTTCGTGATGAACTGATCCAAACCAACCAAGGCAACAGTGTTACCGCAGGGAACATCCTCAACAGACTCTTGCTTCTTTCCCATCCAGATAACA

>Isogroup_277 CTTGAACAGATATAAGAAATATTTGGTTCTCCTTTTACCAACACTTTCGCGCTGCCAAAGTCACATAATTTCAACTGATGAGTATGCGGATTCACCAGAAGATTCTGAGGCTTGATATCTCTGTGACACACTCCAATGCTGTTATGAATGTATGCCAACGATCTGCATATCTGATACATGTATAGTTTTGCATATATCAAGGGCATGCGTTGGTTCATCTTGTTGTAATGTTTAATGACACGATGAGCAGTCTCTGGCACATACTCAAGCACCAAGTTGAGGTAAAGCTCCTCCTTCTCAGTCTTTGAGAAAAAACAATGCTTTAAAGCCACAACATTTGGGTGGTCAAGAACTCGCATAGTCTGCAGCTCACGGTTCTTATATCTCTTGTCTTGAAGAACCTTCTTTATAGCTACCGTCTCGCCAGTTTCCAGACACTTGGCCTGAAAAACAGTTCCAAACGACCCATGACCTACCACACGCTCAGCCATGTAGCTAATGGTCTGTTTTGCTTGCCCATTTCTTCCCTCAATGCTTGTGACTATAATATGTCCTGGTTCCATCCCATTGCCGTTGACAACTATATCTTCAGGGTCCTGATCATCCCTTATGCTCATATTGCTCATCTGATTCGGAAGACCAGCATCACCACTCGTACCTCGGCCGCGACCACGCTAGTCGTGGTGTGGTCGGCGTCT

>Isogroup_278 ATCTCCATGGCATCCGTGAAGGAGAAGCGGGCTCGGCTTTCAGCCCAATTGCGAATTCTATCAGCCTCCGCAGCATATACTGCTCTCCCCTCTTCGTCAAACCATCGGCCCCGGCGAACCCATGCAGTAACCAGCGCAAGCAAGATTCTTGGTGGTGTCAAGTAGTTTACTGCCTTTCCACTTCCTTTCACAACTCGCATTCTAGGAACCAATGTCCTTGCTACTGTTTCTGGAAGCTCACATATTATATTGAACATCTGTTTATTTTGGAGAGATGAACCACTTAGAAGGAGATCTGTAAGGACCATTCCAGGAGAGGCAGTGTGAACTCCAACTTTTGATCTTCTGCTTTCCTTCATAAGTGATGCTTGGAACTGCCTTAGTCCACACTTAGTTGAACCATAAACAGCTGTCAATGGCGTGCTTGATCCTCCAGATCCTGCACCATCCATGTTGAATACATGACCACCCCTTTCTTGGTATTGCATGACATCCATAGCTTCTCTGGTGCAGAGCAAAGAACCAACTAGGTTTGTTGAAACAATCTGGGTAATATCTTCATCAGAAAAATTTACGAGTGGCCTGAAACCTTTATTTGTGCCAGCATTATTTATCCAAATGTCAATTGAACCCAGCTCTTCAACAGCAAAATTTACCAGCTTTTTTACATCTTCAGGTTTGCAAACATCACAAGACGTA

>Isogroup_**279** AGCGGCCGCCGGCAGTACTCATTTCTGAGAGCATGTCTGAGTTGGAGTATCTGCAGCCTTCTGTTGGTATTGTTTTGGGCTTCATAGGGACAAAGATGATCTTTGACTTTTTTGGTTATCATATACCAACCGAAGCTTCCCTTGCTGTCGTTACCACATGTCTTAGCGGGGGAGTAATATTGAGTCTTAGAAAAGCATCAGCTGAGGAACGTGACAAGTAGCCAGCGTGCTGGGGAGAAGTATGGCTAACTGAAGTTAGCCATGACCGGAGGATAGAAAGAAGCAGCGGCCTCCATTTGTTCGCAGTTTGTGTAACACAACAGCGCAAGATTTGAGGGCAATTCAGCTGACTGTGACCAAGATTGATGTTGAAGGCATGACATAGATGCCTGATACAAAACCTACAGGTTCCATAGTGTATGAGCTCCTTCTGAAGCAGAAGGTTTTGGGGGAGGTGATGTTAAGTCGGCAACAGGTGACAGCAAAACCACAGACAACTAGTATATACGACTTGTGATAGTCTTTGTTTATAAGCCTTTTTGCCATGGTGGTTAGTTGTGAAAACAGAACATTTAGCTTGTGGTAGACAACATGATGGCATGCGCCTCATTTGTGTAATTTAGCAATACTGTGCCTGATGTGCCCATGTAAGTACCCTCGGCCGCGACCACGCTAGTCGTGGTGTGGTCGGCGTCTCTC

>Isogroup_**280** CAGAATGTCTACCAGAAAGGCCGACACCACAAAGCCAATAGAAGCTAGTGTTACTGAAAATGGTATACTGCCCTTGTCCTTCCTCAGAAACATTGCTTCATATAATGGGATATTGATGATAACTAGCATGCCACAGAGAATGAGCTGGGGGGAAATTGCATTCAAGAGTGCATTCCAGCCACCTGTCATGAAATGGCGAAGCCCTCCCACCAGGCAGACGAGGTTGAGTAATGCGATAGTCGAAATGATCACATACTCTGGAGTTGATGACCCGAATTCCATAATTTCCAGCTCATACCTCTTGGACACGTCTTCATCACTTACCTTTGATGAAACTGCAAATGTCATCTGTGACAGTCCTAGTAACTTCCGGAGAGTGTCAATGACTCCAAAGAGATACGAGGTTATTCTCTTGACTATCCACATCCTTTGTCCATTCCACCATCCTTTCAACGTGTCTCCACATGATAATGCCTCATATAGGCTGTAAATGTTCTTCACACAGAAGACATATATGAAGGGTGCGATCCATGGACTTGTAATCTCTGGAAAAAGTGGGGTGCCTTTGAGAAGGGCTACTGAAGGGATGGTAACATAATATAGTGTAGCAAGTGAATTGGGTGCCCATAGTCCATAGATACAGTAGCCCATTTGATGTCGTAACTTAATTTTTCCATGTCCAAACAAGAAGGGGCAG

>Isogroup_281 CTCCGTCTCAGGGTGTCCAGGTGACTGGTGCCAAGAAGCGCAAACTTGGATCAGTGAGGAGGTTCAAGCAGGATGAAGCACAGCAAGATCAAGGTGAACTTCATACTGGCAGACGTGAGAAGCCTAGCAACGAGGATGTTGATTCCACAGAGGGAGAAACTGGTGATAGGACCAAGGGGGGGATTGCGCTAACCGAATTCATATTACTAAGATCATCAAACCAGTGCGCTATTATGCCACCATGACAGATGATGTGCAGCAAGTTTCAATAACATTCAAAGCACTCAGGTCTGATGGGCAGGAGGTCCTTGTGGATGACAAGGAATTGAAATCTAATAACCCATTGGTGCTTATAAACTACTACGAGCAGCATCTCCGTTATAATCCCACCTCGTGAAAAGGACGAACCGAACAAGTTCGGTTTCATTTGGTGCATGAGTTGAGATGTCTTTACTACAGCTTTCTTTATGCTAGAGAAAGTTGTGGTAATATAGTTTTGTCCTAGAAGTCGTGCTGAATGTAGGAGCTGAAATGGAAAACCAGGGATATGTAATATGTGCTGACAAGTTATTGCAGATTTTTTGTGGCCAGCATCCCAGTCCTAGGCCTCAATGTGAAAAACCAGGGATGTGTATTTGTGAAGTGTCGGTCCAATGGGGCTCCTGCCGTTCGACAGATGTAAAATGGAGCAGTTGT

>Isogroup_282 CACAATTTAACCAAACATTGTTTCTAGAAGCTAGGCTCCAGAAATGATCGAAGTGGACTTCTGACGGCCATCTGGAATAGAAAAGAGCACAATTGGTTTGGACGACCGGACAAATGGAACATCACACCTGTAATACCCTTTCCTTTCAAGCTGTATAACTTCTCCTTGCTTGACATTCCGCATGTTCGGGTCTCCAAGAGCTAAAGCTTCCCGTCGAGTGCAAGGGTTGAGATTGTCAAGGAAGTTCTCATCTTCTTCCAGCTTTTTTCTTATTAATTAGGTAGTCAAAATCTACCAAAGAGAGAGACACAAGGTCTTCAATATCTGATAGCCATGTCAGTTTCAGCTTTGTTGTCTTTACTGACCCTTCAAGATGGAGTTCACCAACTAGTTGAGTAATCGTCCCATTATCAGTCTTGATTTCTCTGATGATAGCATTTCCCCAGTCCATCAAAGTGACTTCCTCACCGACGCTAATGACTGATGCATCAGCATACTCTAGCAAAATTCTGTTTGCGAAAGTTGTAGCCTTCTTTCCAGCACCCTCATATTTCTTGTGCCTTGGCAAGATTCGAACAAATGGTTCGTCTGGACCATTAGTAAGTGTCAAGGGCACATGCTTGTCTTTCAGCACAGCGGTATGCCTTCCACACACTGGATCAACTATCTTCTTGTTGATTGTCCACAGTTTAACCT

>Isogroup_283 CTGTGTATCTTGACGTTGCATTCCTGCCTATTTCTTGTTTTGCTTGCAACCTAGACTTAACCACTAGCAATGGATATGTCACAACAGTAGCTCCCAATTTTGCCATTGCACCTAATAAGAATACCTCCATAGCAGTGATGTTCTTCTTTGGTAGTTGGTTTCCAGACCGCTTTGATCGAAGACGCTTTGAGAGCGTTTCAAATATCATAAACTGAATTGAAGGGTTGCATACCATGATTAGTGTTGGAACAAGTCCTTTCCAGAATCCACGTATGCCTGATTCACGATAGACCTCCCGAACCGCTTGAAGTGTGCCATATGGGTGAGGTTTTTCAGAATCAAGTTTAGCTAACCTAGCCTTTAGAATTGAAACATCCACTGAATTAGCCCTAGATATTTCCTTCAGCAAAAGCTCCCTCTTGGACTCTATCATCTTCCTTTCTGCCTGAGTATGTGTCTGCATACGTGTCACAAGAACCCATATTGGGATTGTAAGAAGAACATTGATCGACCCAGCAATAGCTGCAATAACAAGCCAAGAGAACATCCCTACAGTGCCATCCCCTAGGCCTTTCTTTCCCCGAGCAGCTGCCACATTTTCCACCTTGTTCTTGAGAAGCTGGTAAAAGTAGTAATAGATTCCCTGCGAGGCAGCGGTGCCAATAAGTGAGGGTTTGAGGCCGCTGTACCTGCC

>Isogroup_284 GACGCCGACCACACCACGACTAGCGTGGTCGCGGCCAGGTACACGATTAATTCCATATATGAGGCTTGCCCCTGTTTCTTCAATTTCAGAGAAGTTCCAGCTGGATGTATTTGCAACTTCTGTCGACTTCAACGGTTTACTCAGTAAACAGCACATAGAAGCCATTGAAGCCTTAAATATAAGTGGTGCTGAAGAAGTCATAGTTACTGAAGAAATGATGTCCCTTGTTTCTCTACAAAGGATGCAGCTTTGTCAGTGCAATGTTACGGACCAGAATCTTAGTAGGTTTCTCCAGGCTCTGCCTTGTCTATCCTCGATGGAGATAATAGACCTGCCTAGCGTAACATCTCTTCCAGCATCTGAAACACTCAGGTTCAGCACAATGCTCACTGAGTTGTCCATACGCAACTGTCAGTTGTTTCAGTCTCTGTCGTCATTGCAGTCTTTTGATTCACTAAAATATCTGGTGATAAAGAGATGTCCTAAAGTAACTGCAGCAACATTCCCGTTGAACTTCAAGAGCCTTTCATCTCTCAAGGTGCTGAGAATATCATACTGCTCAGAGCTCCAATCTTTACCGTCATGTGGTCTGCCATCCTCACTAGAAACACTTCACATTATTGGGTGCCACCCGGAGTTGTCCAGGCAATCAAGAAACATGAAAGGGCATTACTGTGAGAAGCTTGCAGTAG

>Isogroup_285 CAACGAGGGGGACGAGATCCCGCCACTAGTGATCGGGGTCAGCGCTCCCCAAGGCAGCGGGAAGACAACTCTTGTTTTCGCGCTTGATTATCTATTTCGAGTTTCTGGTAGGAATTCTGCCACATTGTCTATAGATGACTTCTATTTGACTGCAGCAGAACAGGGTAAATTGAGGGAAACAAATCCTGGGAATTCCCTTTTAGAGTTTCGTGGAAATGCCGGAAGCCACGATCTACAATTCTCAGTTGAAACACTTGAGTCACTGATCAAACTGACAAAGGAAGGTATGAAGATGAAGGTTCCACGCTATGACAAGTCTGCTTTCGGTGGAAGAGGTGACCGGGCTGATCCTTCAACATGGCCAGAGGTTGAAGGGCCCATAGAGGTTGTTCTATTTGAAGGATGGATGCTTGGATTTAAGCCTCTTCCAAATGAAGTTGTAACAGCAGTGGATCCTCACCTTGAGGTGGTTAATAAGAGCCTAGATGCATACTATGACGCATGGGACAGGTTCATCGAATCATGGATTGTCATAAAAATTAAGGAACCTAACTGTGTATTTCAGTGGAGACTGCAGGCAGAGGTAGCTATGAGGGCTGATGGTAAAGCAGGAATGTCTGATGAGGAGGTTATAGATTTTGTATCACGGTACCTCGGCCGCGACCACGCTAGTCGTGGTGTGGTCGGCGTC

>Isogroup_286 ACACCACGACTAGCGTGGTCGGGCCGAGGTACCTCAGGAAGGTGGACCTCAAGATGTATTCCAGCTACGACGACCTCTCCGCCGCACTTCAGAAGATGTTTAGCTGCTTCATCACTGGTCAAAGTGCGGTGCGTAAACCATCAACCAAGGACAGGCTTACTAATGGTAAGGCTGATTCCCTTCAAGATCAAGAGTATGTCCTTACATACGAAGACAAGGACGCTGACTGGATGCTTGTTGGTGACCTTCCATGGGATTTGTTCACCACTATCTGCCGGAAACTGAAGATCATGAGAGGATCTGATGCTGCTGGAATAGCTCCAAGATCCCTTGAGCAGACAGGTCAGAACAAATAAAATTGACCTTCATCTGCAGTAAAGAAAAAGAACCGAGCTAAGCCGTGAGATGATGTTGATGCCCTAAATTATTCATGCCAAGTATCTCAAATATCCCAAGTGTTAGTCTAGTGTCATGTATTTCTGAAACCTCTGCATTACACATGTCTGTAATGTGATCTGCTGTGATATTGCAATTGTGAGTCCCTTGGAATTGGAAGGTTATTAGTCATGTCAGCAAGCTCCGCGCTTCCTGTGTATGCCACTAGTATTCATGTCATATAGATTCAAGTGTTGCGCTAAGTTATTCTTCTGTCTTGAAATTATCCAAATGCCACAAGAACTTCATGGTGGG

>Isogroup_**287** TCACTTCCCTGATCATGCTGATGCTGATGGTGTATACTAGTGTTGAGTAAGCTGTGAACCGAGAATCATATGTTCTGAATTGCCAGTCTCGCGGCAATCATACCAATGTTGTGTTCAACATACTCGATTCCGCTGTTTAGTCTTTCCAGAGTGTCCGCTGAATCCATTGCCTTTATCTTGTATTTCTGCAGAGTAAGAAGTTCTGCATCTGACATACCTGAAACGGATTGGTATGCGGCGAAGGAGAGACGCTCTGGCAGACCAGGGATAAAATTTTCATCATACCCTTCGAATGTTTCTTTCTCAGACCATGATAGAGAACTTTTGATGTTTGTTTGCAGCGGTTCATCCTCGGGCACCCTCAGCTTCATTTGTAGGCTGTGCAAATTGCACATGGACTCCCTTAGTTTGGAAATTCTCACGCCCAATTCGATGTCATCGGAAGACCTGTCCATCATGGGAGAAACATCACCTCGCAAGTAGGGCTCCATCTGGAGAAGGTTCTTGATGTTCACACGGCATACACCTCTAATTGACACTAATGCTCCAAAGTCCATCTTTTGAACACTTTCTATCTGCACCAAACAGCCATATCTTACAGCAAAGGAATCCTTAGAAGAGCTTGACAAAACTGGATCAAGGACAAAGTGTACCTCGGCCGCGACCACGCTAGTCGTGGTGTGGTCGGC

>Isogroup_288 GACGCCGACCACACCACGACTAGCGTGGTCGCGGCCGAGGTACAATCGAGGCATTCTATAACGCAAATTTGGGAATTACCAAAAAGCCAATACCAGATTTCAGTTTCTATGACCGTTCTGCTCCAATTTACACACAACCTCGACACTTGCCTCCTTCAAAGGTTCTTGATGCTGACGTGACAGACAGTGTTATTGGCGAAGGATGTGTTATTAAAAACTGCAAGATACACCATTCAGTAGTTGGACTGCGGTCCTGCATATCTGAAGGTGCAATTATAGAGGACACATTACTAATGGGTGCAGACTACTATGAGACTGAAGCTGACAAGAAACTCCTTGCTGAAAAAGGCGGCATTCCCATTGGTATTGGAAAGAATTCACACATCAAAAGAGCAATCATTGACAAGAATGCTCGTATTGGAGACAACGTGAAGATAATCAATGTTGACAATGTTCAAGAAGCAGCCAGGGAGACGGATGGATACTTCATCAAAAGTGGCATCGTAACTGTGATCAAGGATGCTTTACTCCCTAGTGGGACAGTCATATGAAACAGATGCAAAATATGGGGCAAGTCACAGCGCTTCTTGCGCCATTATGGAATCAACCAATGAAGTCGCCAGAAGATCATAAGAGCAATAAAAAGGAGTGCCCTGCAAGGCACTTATCCACCTTTATTCTCCCTTAAT

>Isogroup_289 CAGGTACATGACCATAACAGCTTTAGGAGCAGATATGCTAGCTGATTGCATAACAGCAGGGCGTGATCCATCTCCATATAGCACCGGAAATCCTGATTTTCTAGCTGACTTAACTACTGCAGGATTTAGATCAAATGCTACATATGGCCATCCTTCCGTATCTTGCTCGAGCCCAAAAGATAATGGTGCGGCCAAAAACTTTGCAAGAACCTCTCCCATTTCTCCAAATCCGAGAATGACAATAGGTTCAGTCGCACCATAATTTGCCACCTCGGCTGGTTTTTCTTTTGTCTCAGATCGCTCATCAATGATTCCTGCTGCTTTTTTTCCAAGTTCATTGAGAAACGGAGTTAATGCCATTGACAACACAACAACGATAATGAGCAGCTTATTCAGCTCAAGCGGTAGGACGCCAAGCCTGTTTGCCAAAGAAAATACCACGAATCCAAACTCGCCTCCTTGAGAAAGCAGCAGTCCTATCCTTATGCTTTCTTGAAGAGTTAGCCCGACTCTTGTTCCTAATGCAGTAGTTATTAATGTCTTGATTGCAATAAGTCCAGCCAACAGGGATAGAACATTAGGCCACTCCCGGATGAGAAGCTGCATGTCGATGGAAGTTCCTGTAGTCACGAAGAACAATCCAAGCAGCAAGCCTCTGAACGGCCTTATGTCCGCTTCAATTTGTG

>Isogroup_290 GCCGACCACACCACGACTAGCGTGGTCGCGGCCGAGGTACTCCCAGGCGAATCTTGAACTGGTTGGTGAGGTTGAGCCCTTTGTTGTGTTGGGCATTGCATAGTGATGCCATTATGTAAACCAACAGTTTGGGTAGCTCCTTGCATACAGTGCTGAGGATTTGACTGAGAATTTACCATCTGCTGCTGCAAATCGAAAAGCAGTTTCTGCTGCTCGAAGGTCAGATGTTGCTGTGGCTCTAGAGCCTTTAATAGTTGAAGTTTCATTTGATAATCAGATAGCTGCGTATTCATGTTTGCATCACTGGACGCATGAACAGGTGCCATCTTCATTTGCTGCTGCTCCTGCACTGAGTGTTGAACCTGCTGATGATGTTGAGGTTGTTGCTGCTGTAACTGTTGCTGTAGATGTGGTTTCTGTTGCTGCAGAATCTGGATTTGCTGAAGGCCTCCCTGAAAGACAGAATGATTCTGGACAAGAGGTTGCTGTTGCTGAGACTGCAGAATCACTTGAGGTTGCTGGAGCTGAGCTTGGAACACATGTTGAGGTTGTGTGATGTTTAAGTTTGCTTGGCTGCCTTGCACTGATTGAGTAACCCCATGCTGCTTCTGGTCGGATTGGCGATCTTGATCTACCTTCTGTGGTACTGCCGGGCGGCCGCTCGAGTCGTGGTGTGGTCGGCGGTC

>Isogroup_**291** GCCGCCCGGGCAGGTACTGATGCAAAAGGATGCTTTCAGATTCAACACCAGCTATGTCGATTCGACAGTGTTTTACGAAGCATACAATGGTTCAGTTCCGGTTGTCCTAAACTGGGAAATACAACAGCTGACTTGCGAAGTAGCCAGGCAGAACATGAGCTCCTACGCGTGTATCAGCAGCAACAGTGAATGTCTGAATTCAACCAACGAGCAAGGCTATCGCTGCATTTGCTCCCATGGATACCAAGGCAACCCTTACATCAAGGATGGATGCACAGATATTGACGAGTGCCATGAAAATGATAATCCTTGCGGCCTTGAGATCTGCCAGAACACACTGGGGAATTACAACTGCTCATGCCGCCTAGGATATTATAAGACGGATGGCGTTTGCGTGCCAAATCCTTCCCGTTTTCGGCCCATGCCTGTTATTGGTGCAAGTATTGGATTTGTCGTCCTTGTGATACTCGTAGCATGTGCAAGCTTGATCCGAGAGAGGAGAAAGCTAGAGAACATGAAACAGAAATACTTTCGGCAGCACGGTGGTGTGATACTATTCAACGAGATGAAGTCAAAAGAAGGCAATGCATTCAAAATCTTCCGTGAGGAAGAATTACAGCAGGCGACAGACAACTTTAGTGAGAAAAAAGTGGTGGGCCGCGGAGGCCATGGAACCGTGTACCT

>Isogroup_292 GAGGTACACCTTCTTTTGCCTCAATTGCTTGGTGGAGTCCGTCACTCCATCTTCTGCCTGCCATAACTCGACCTGTGAACTCATCCACAATAAGCACTTCCTTGTTACGAACGATATAATTCACATCTCTAAGGAAGAGCTCCTTGGCTTTGATTGCATTTAAAACATATGAAGCCCACTGTTCACGAGGATCATATAGATCATTTATGTCCAGTATTTCTTCAGCATCAGCATAGCCTTGTTCTGTTAGCAGAACATTTCTCTGTTTTTCATCGACAGTGTAATGAATATCTTGTTCAAAAACTTCAGCTATTTTTGCTGCTTTGTAATACCGGTCACTTGGCTTTTCAGCAAGGCCCGATATTATAAGAGGTGTCCGCGCTTCATCAATTAGGATTGAATCAACTTCATCTATCACACAGTAATTGAAGTTCCTCAACACAAGCTCATCTACAGTCATCGCAAGGTTATCTCTTAGATAATCAAATCCAAGCTCACTATTGGTGACATAAGTAATATCACACAAGTAATTTTCCCTCCTTTGCTCAGGTGTCATGTTCTGTTGAATTAGGCCAACCTGAAGTCCTAGAAATCGGGGAACTTGACCAACCCACTCACAGTCACGTCTAGCGAGATAATCATTTACAGTGACAACATGGACTCCTTTCCCACTTAATGCATTTAA

>Isogroup_293 TTGCTGCCACTAATAGGCCGTTTGATCTTGATGAGGCTGTTATTAGGAGGCTCCCAAGGAGATTGATGGTGAATTTACCAGACGCGACCAATAGGAAAAAGATTCTTAGTGTAATACTATTGAAAGAAGATTTAGCAGAGGATGTAGATCTGGAGGCACTGGCCAACTTGACTGAGGGATATTCAGGCAGTGATCTGAAGAATCTGTGTGTTACTGCTGCACATCGTCCCATAAGAGAAATCCTTGAAAGAGAGAAGAAGGAGAGATCCTTAGCAGAAGCAGAAAATAAACCATTGCCTCCTAACTATTCTAGCAGTGATGTCCGTTCCCTAAATTTGAGTGATTTCAAACATGCTCATGAACAGGTATGCGCAAGTATATCATCCGATTCGACAAATATGAACGAGCTTATTCAATGGAACGACCTCTATGGCGAAGGCGGATCTAGGAAGAAGACACCTCTAAGCTACTTCATGTAGCTTATGTGTTGTAGTCTGTGTATATACAACAGAAATATTACAGCAGAAATATTAGCATGGAGGGACCAAGATGGACGATGAGTAACTTTCGTGTCGTCCTTGGTTGATAGGTGATGGCGCACGCAGGGCCTCGGTTTGCTGCGTGAACTGTCGAGTTTATTAGTTTCAATCTTTATTCATGTCAGTTCAAATTTCCAACAAAATAG

>Isogroup_294 CAGGTACTGGAGTGCTACATGGCCAAGAGAAGTTGAAACGTTGGCTAGACAATTTCTGCAAAACCCTTACAAGGTAATGATAGGAACGGCTGAACTTAAAGCTAACCATTCTATACAGCAAAATGTTGAAGTTATTTCAGATCATGAAAAGTATCCCAGGCTAAGTAAGCTCTTATCTGATCTAATGGATGGGAGCCGAATCTTGATATTTTTTCAAACGAAAAAGGAATGTGATAAGGTCACTCGACAACTTAGAATGGATGGATGGCCAGCATTATCTATACATGGTGATAAGGCTCAATCTGAAAGGGACTATGTTCTGGCTGAGTTTAAGAGTGGGAAGAGTCCTATAATGGCTGCCACAGATGTAGCAGCACGTGGTCTTGACGTGAAGGATATAAAGTGCGTGATCAACTTTGATTTCCCAACCACAATTGAGGATTATATTCACAGGATAGGTCGAACTGGTCGTGCTGGTGCAACTGGAATGGCATTTACTTTCTTCACACACTCTAATGCAAAGTATTCGAGGAATCTTGTCAAGATCTTGCGTGAAGCTGGCCAGACTGTGAATCCAGCGCTGGAATCTATGTCCAAGAATGCCGGTTCTATGGGAGGAGGAGGCAATTTCCGCTCAAGGGGTAGAGGCGGGTTTGGTAACCGTTCTGGATCAAATAGCATCCCA

>Isogroup_295 GGCTTTCTAGGGCACCTAGGGTTTGCTATGCTTTTTGGATTCACTGTAAAGAATGACTCCTATAACTGTGAGAGTGTAACCAAGCATTCCGGTTATAGACACAGGATTCTTGAATATCAGAATTGAGACGACAACTGCCACAGCACCTTTTGCATTACCAAGGACCTGTAGAGTTAATGCACTAGTATGTTTGGTCACCAAGAAATTGGTCAAATTGACGAAATATGCCAAGCAAGAGTTGAATAACAGCAACCAGACAATGGTGAAATCCTTCCTCGCTAGTTCTATTGTAACACCAACAACATTATCCTCCATAAAGAGAGTTGCTGTTATTCTCTTGCTTCCTGCTACTCTCTTTATGGAGGATAATGTTGTTGGTGTTATAACAGCAACCAGACAATGGTGAAATCCTTCCTCGCTAGTTCTATTGTAACACCAACAACATTATCCTCCATAAAGAGAGTAGCAGGAAGCAAGAGAATAACAGCAACTGGAGCCATGTACAAGAGCAGATTCATAGAATTAAGCTTCTCCCCCCTCAGAAGACAGAAGAATTCCTTGCAACACCGTCTTCAATGCCCTTGCAGCAGTGGCTCCAATGCACATGATGAATCCAAACAGATGAAAGCTAGGCTCGCTGCCGCTGGCGATGACGACGCCGGTGACGACGGGGACGAGAGTGAG

>Isogroup_296 CTGATGATAACAAAGAAGATCAATCGGAGCCGGAGGAAGAAGAGAATATCAATGATGATGGATACGGTTTTCAACATGGGATGGGGAGCGGAAAAGCTCGTAAGTCTGAAGAAGCTGGTTCTACAGGATCTTCATCTGGAAGCCGGAGATTACCACCACCTGCCCCATCCTCATCTTTGAAGAAGTTACGGTCTCTGTCTGCATTAGATGCCCGGCCAGGCACTTTTTCGAAAAGACCAGATGACCTAGAGGAAGGAGAGATTGCATTATCTGGGGACTCACCTATGGATCTACAGCAGTCAGGCAGCTGGAACCATGAACGAGATGACGGTGAGGATGAACAGGTGCTGCAACCGAAAATAAAACGCAAAAGGAGTATCCGTTCTCGGCCAAGACCTAATGCAGAAAAACAGGAAGATAGATCTGGTGTAGATGGCACTTTCCCCCAACGCGGTGCTCGCCTTGCATTCCCAGGAGATGGTGATTATGATTCACAGCTGAGGTCTGAGCAAGATGCCCATAATTTTGTGGATCCTACTTCCAGGCAGCAAGACACAGTTCATCCGATAGTGAAACAGAAGCGTAATATGCCATCTAGAAAGGCCTCTCCTGCTACTTCTAGGGCAGGGAAATCCACCCACTTGTCTGGGTCTGGCAAGGTCGGCGATTCTAAGATGTCTGACA

>Isogroup_297 GAGACGCCGACCACACCACGACTAGCGTGGTCGCGGCCGAGTTACATCCTTGAGAGATCCTCAACCTTGTCATCACGAAGTAATGCATGGCATCCAGAATGCTCCTTCTCCAGAAGTTGGCTTGCATACTGTGTTAACAACTCATGTTGCACTTTCTCCAATAGCTTCTGTTCACTACTAGAGTGCAGATAATGAGCAACCCGCTCCTTCTCCTTCTTCAAGCACTCCTCAGCCTTTAACATATAGTCAGGGCAAGAGTCTTCCACAATCCACGTTTGGGCCTTGATAGAGTAGTAATCTGCAGTATCCTTCAGTAAGAAATCTTCAAAATCATTCTCGTAACACTCCATACTGCCTAAGCCGATTTCAACAAAAATATCCAAGACGTTCTTTAGCAGAGCCCTATCAATTTGTTCACCCTCACGTTCGTGGTCTATCAAGGAAATCACTGCACTTTTCACTTTCCCTTTGATTTCTTGATATACCAAGTCTCGAAAGCAACTAAGCCCAACTTCTCTCAATGCTGGAAGTGATCTCCGTGAAATGAAATATCGATCAAGGTAATGGAAGAAACGTGATAGCCATCGAACCATCACTTTGTGGTTTGACCACCTCTTTACTAGCTCCCTCAACATAAATTCGTCATGCTTCTCTCTTAATGAAGGTAAGACCATGGAAGAGAT

>Isogroup_298 ATTACATAGTCCATGGAATCCTCTCGCAGCAATGTCTTGAAATCCTGGTATACGCGTGCTACATCCTGGAGGGAGCTCGGCAAACTGGTGTTGGTGTTAATGTTAAGTGGGATATTCTGTTTGGAGATTACATCACCAGATTTTGGTTGATCCTTCAGAGAAAATGCCTGACAGCGAGCATCAGTTAAACTGTAGACAATTTTCAAATGTCTCTTCAGTTTGAGAAGAAGCTGGAGAGCTATCGCATCATGGCAATCTTCCTGAAATTTGGCCATGTCCACACCTGACGTGCTGCACGAATTGTTAAGCATCGTTTGAGGGATATCTGCCAGATTATGGTCAGGATATCCCACAGGCTCATGCATAACATCACCAGTGTCCCCAGGCTCATGGACAACATCACCAGTGTCCCCAAGCTCATGCACAACATCACCAGTGTCCCCAGGCTCATGCATAACATCCCCAGTGTATCTCGGGATACCCACCATATCTTGCTGTTGATCTATGGTAGTCCAGTTCTTCAAATTAGACTCGATTGCTCCAGCTCTAATTTGAATAAATCGATTTATATCATAGACCAAGTAAAGCGGTTCATCAGGGGATGTGAACGGGAGGGACGCAAGAACTTCAACACAGTAGACAAGAAAGCCAATTGTGGGGCAACTCCGGTTGTCCGAAACAAA

>Isogroup_299 CTGAGGATGAGCCTTTGTTGTCTGTCGAGGGTGGTAAAAAGGGTGCTACTTCTGTAGGGTCAGAGATAATGAGCTATGCTGGATGTGTGGTTAAATTCCTGAAAGACAAGCAGCTTCTACAGCCACCAATTATTGCCTCTGTTTTTGCAATTGGCATTGGTGTTGTTCCAGTCTTGAAGAATTTGATATTCACGGATGATGCACCTCTATTCTTCTTCACAGACAGCTGTCTGATTCTTGGGGAAGCTATGATCCCTTGCATTTTGCTTGCTGTGGGGGGTAATCTTGTTGATGGTCCTGGCGAAGGAAGTCAAAGGCTTGGTATGCGAACCACCGTTGCCATTATCTTTGCACGGTTGATCTTGGTTCCAATTGCTGGTGTTGGCATCGTGATGCTAGTTGATAAACTCGGTTTCATTCCCAAAGATGACAAAATGTTCAAGTTCGTCCTACTACTCCAGCATTCTATGCCCACATCGGTGTTGTCAGGTGCTGTTGCAAATCTGAGAGGGTGTGGAAAAGAATCAGCCTCGATTTTATTCTGGGTGCACATTTTTGCTGTATTTTCCATGGCAGGATGGATTATTTTCTACTTGACCTTGCTCTTCTAAATACTGAACGGCAATATCCGGAAAAACTATGTACCTCGGCCGCGACCACGCTAGTCGTGGTGTGGTCGGCG

>Isogroup_300 GTCCGGACGCAAATTCTGATGAACGACAGCCGAAGAGACATCGGCAAAACTTAGGTCTGGGTATGGCATGTCACAGCAATAGATTTCCCACAAACAAATGCCAAAACTGTAAACATCACATTTCCTGTTGTATGGTTTACCATCAAGAACCTCTGGGGCCATATAACCAAGTGTGCCTGTCGCACCAGTCATATCCTTCGGGTTCTGAGCCTCAACACGAGCAACACCAAAGTCAGCTATCTTAAGGTTTCGCTGTGTATCGAGTAGCATGTTTTCGGTTTTCACATCACGATGCACAATCTTTCTTGAGTGTAGATAGCTCAATCCTCTGGACAGATCCAGTGCAAGCTGAACCACAACTTTGTATGCTAGCTTCCGCCGTCTGTTCTTTATCAAATACTGCTTCAAAGTGCCTCCCGCTAGATATTCTACCACAACACAGCACGCTCTGGCTGGCAAGTTGGCACGCGCACCTTTGTCGTTGGCTGGAATCTTAAGGTCAGTGGTCCCCATAGACGCACCAATAAACCTTGTAACATTAGGATGGCTGAGCTTATGCCAAACAGCAACCTCCTGCTTAAATGATGTTCGCAAAGCGGCAGTTTCGGCTTCTGTGGCAAAACCATCTTCTCCCCAATCCAATAGTTTCACTGCAACTTCCTGGCCGTCATAGGTGCCGCG

>Isogroup_301 GACGCCGACCACACCACGACTAGCGTGGTCGCGGCCGAGGTACCATAGAGATCTGAAGCCAGAAAATCTACTCCTTGACTCATATGGAGCTCTCAAGGTCTCAGACTTTGGTCTCAGTGCATTTGCACCGCAAACAAAAGAGGATGGACTTCTGCATACTGCATGTGGAACTCCAAATTATGTTGCACCCGAGGTTCTTGCTGACAAAGGTTATGATGGTATGGCTGCTGATGTGTGGTCTTGCGGCATAATCTTATTTGTCCTCATGGCTGGGTATTTACCCTTTGATGACCCCAACTTAATGACTCTGTATAAAATGATCACCAGGGCTAATGTTTCTTGTCCACCATGGTTTTCTACCGGTGCGAGAAAACTTATTAAGCGCATTATTGATCCGAATCCCCGGACCAGGATAACAATTGCAGAAATTTTGGAAGATGAATGGTTCAAAAAGGACTATAAACGACCACATTTTGAGCAAACTGGAGACGTGAGCCTTGAAGATGTCGATGCTGCATTCAATAGTTCAGAGGAAGATCTTGTGGCGGAGAAAAGACAGAAACCAGAGTCCATGAATGCATTTGCTCTTATTTCAAGGTCGCAGGGATTCAACCTTGGAAATTTGTTTGAGAAAGAGATGATGGGATTGGTAAAAAGGGAAACATCCTTCGCGTCCCAACG

>Isogroup_302 CAGGTACTCTAGTATCAACAGCATGAGGAAGAATCTCCATTGCTTGACTTGGGATTGGCTGAAGAACAACACTAGCATCATAGATTGCCACTGTGCTGTAAGGATGGGACACTGATAAGAGTGATCCAGGGTTGAATGGAGGTGCTGAAGCTGAAAGTTTGCTTCCACTTGTTTTCTTGTGTGGAGATGTGCTTCCGTCACTATTGCTGTCAGAAGCTTTCTCTGAATGACTGCCTCCCTCTGGCTGACTTGAGACTATCTCTTTGCTGCCCTCATCACTTGACTTCTCAACTTCATCTGTAAGCTTCCTATCTTCTTCCTCAGATGAGGGTGTTAAATCAGTATCTTGTTCATTATCCTTCTCCTTTGCTTCCTTCTGTTCCAAAATTGGTTTCCATACGGTGCCAGGCGGTGACGCTGCCACATCTTTGTATGATACCAACTTGGAAGCTATGCTGGTTGGCCTGTTAATTCCTGCAGGTTTTATGCTCCGTTCTGGCTTGGACATAGCATTACAAGACCTGTCTTCAGTAATGGAAGGATCGCCATTCAACTTGTCACTTTTCAAGAAGTTGCCAAATGATAGTTGGCTAGGGGAGTATATAGCTTCCTCTTTGTGCTCTTTTGTTTGTTGCTGTACCTCGGCCGCGACCACGCTAGTCGTGGTGTGGTCGGCGTCTC

>Isogroup_303

AGCGGCCGCCCGGGCAGGTACTCTTGAGCCATCTCTTCGAGATCAGCAGGTATGTCTTCGTATGAAAATTGTGCACCAAGTTCCTCTCCTGTCCATACAATAGCCTTCATTCTGACTAGATCAACAACTCCTTGGAAATTGTCCTCCGAACCTATCGGCAACTGTATCACCAAAGGATTTGCACCCAAGTTTGCCACTATCATGTCCCTAGTTCTATAAAAATTAGCTCCAAGGCGGTCCATTTTATTCACGAAACATATTCGTGGAACTCCATACTTATCTGCTTGGCGCCACACAGTTTCAGATTGTGGTTCTACCCCAGCAACACTGTCAAAGAGACATATAGCACCATCCAGCACCCTGAGAGCACGTTCAACCTCAAGAGTGAAGTCGACGTGCCCAGGAGTGTCAATGATGTTGATTCTGTGTTTGTCCCAGACAGCAGTGGTTGCTGCAGATGTAATAGTTATTCCTCTCTCCTGTTCTTGCTCCATCCAGTCCATAGTGGCTGTTCCCTCATGAACCTCACCAATTTTGTAGTTCCTTCCAGTGTAATAGAGAATGCGCTCTGTAGTTGTTGTTTTCCCAGCATCAATATGGGCCATAATACCAATATTACGGTAATCCTCCAAAGGTACCTCGGCCGCGACCACGCTAGTCGTGGTGTGGTCGGCGTCTC

>Isogroup_304 GCCCGGGCAGGACGTTTTTTACTGTGTAGTGACACTCTTAGCCAGGTTCCTTGGTTGGTCGACATCGAATCCACGAAGAACAGTAAGATGGTATGCGAGCAACTGTAATGGAATAATATTAATCACTGGCTGGAGACAATCAGCAACCTTTGGAACTTCAATCACTCTGCAAGATCCACTGGGACAGACAGCAGATGCATCTCCCTTTGAGCACATGATAATCAGACGCCCCTTACGAGAAAGGAGCTGCTGGATCACTGACTGTTGCTTGCTGAAGCATGCATCACGTGTTGCAATGACAATGATGGGAAGGTTTTCATCAACCAATGCTAGCGGCCCGTGTTTCATCTCACCAGCAAACATGCCTTCACTGTGCATCAGTGCAACCTCCTTAACTTTGAGAGCACCCTCCAAGGCAGTGGCATAGTTGTAACCTCTTCCGAAGACAAGGAGGGACTCCGAGTCAACCAATGAAGAGGCAAGTTCCTTCATTTCAGAATCAAGTTTCAGAACTTCACTGGCATTGCTTGGGAGACTCGAAAGACCACTGATGATAGCTTCCCTTCTAGGTTGAGTGGATATTTGGTCAGATCCAATAGCCAAGGCCAACATTGCCATGACTACTATTTGACTGGTGTACCTCGGCCGCGACCACGCTAGTCGTGGTGTGGTCGGCGTCTC

>Isogroup_305 AGCGGCCGCCCGGCGAGGTACATAGGATATGGATAGTCTTTTCCAATGCGATAAAATAAAGCGACATCGTGTCTATTTTTCTTTGCTAAAGGGGTATTTCCATGGGTTTACCTTGGTATCGTGTTCATACTGTCGTATTGAATGATCCGGGTCGATTACTTGCGGTGCATATAATGCACACAGCTCTAGTTTCTGGTTGGGCTGGCTCGATGGCTTTATACGAATTAGCAGTTTTTGATCCCTCTGATCCTGTTCTGGATCCAATGTGGAGACAAGGTATGTTCGTCATTCCCTTCATGACTCGTTTAGGAATAACGGATTCCTGGGGTGGTTGGAGTATTTCAGGAGGAACTGTAACAAATCCGGGTATTTGGAGTTATGAAGGTGTGGCAGGTGCACATATTGTGTTTTCTGGTTTGTGTTTCTTGGCAGCGATCTGGCATTGGGTATATTGGGACCTAGAAATATTCTCTGATGAGCGGACGGGAAAACCCTCTTTAGATTTGCCCAAGATCTTTGGAATTCATTTATTTCTTGCAGGGGTGGCTTGCTTTGGCTTTGGCGCATTTCATGTAACGGGTTTGTATGGTCCTGGGATATGGGTATCCGATCCTTATGGGCTAACTGGAAAAGTCCTGGCCGCGACCACGCTAGTCGTGGTGTGGTCGGCGTCTCTC

>Isogroup_**306** GAGACGCCGACCACACCACGACTAGCGTGGTCCCGGCCGAGGTACCTCTTCAACATCTACTTCAACATCTACAACAAGCAGGTCCTCAAGGTTTTTCCATATCCGATAAACATCACAACAGTTCAGTTTGCTGTTGGAACTGGCATTTCCTTGTTCCTGTGGTTAACTGGTATCCTTAAAAAACCAAAGATTTCTGGTGCACAGCTCCTTGCTATCCTCCCTCTGGCTATTGTCCATACCATGGGCAATCTTTTCACAAACATGAGCCTTGGAAAGGTTGCAGTCTCATTTACACATACCATCAAGGCCATGGAGCCTTTCTTCTCCGTTCTCCTTTCAGCAATGTTCCTTGGCGAGCTGCCTACTCCTTGGGTTGTTTTGTCTCTTCTTCCTATTGTTGGTGGTGTTGCACTGGCATCTATTTCCGAAGCTTCCTTTAACTGGGCTGGATTTTTGGCTGCGATGGCTTCAAATGTGACCTTCCAGTCAAGGAATGTCCTCAGCAAGAAGCTTATGCTGAAGAAAGAGGCATCTCTGGACAACATTACTCTCTTCTCTATTATTACCGTCATGTCATTCTTCCTCTTGGCCCCAGTTGCCTTACTGACAGAAGGTGTCAAGGTCACTCCTGCGTTTCTGCAGTCTTCTGGTCTGAACTTACAACAAGTGTACCTGCC

>Isogroup_307 GGAGTTCGACGCTAGATCTCAGTGGTCTGGTTGCAGCACAATCGGGACCATACTTGATCAAGGTCACTGTGGTTCTTGTTGGGCCTTTGGTGCTGTGGAATGTCTCCAGGATCGTTTCTGCATTCATCTCAACATAAGCATCTCACTTTCAGTCAATGACCTAGTGGCTTGCTGTGGTTTTCTGTGTGGTCAAGGCTGTAATGGAGGATATCCTATCAGCGCATGGCGCTACTTTCGTCGGAAAGGTGTTGTGACTGACGAGTGCGATCCATACTTTGATCAAGATGGTTGCCAACATCCCGGATGCGAACCTGCTTATCATACACCAAAATGTGAAAAGAAATGCAAGGTGCAGAACCAAGTATGGAAGCAAGAGAAGCATTTCAGTGTTAATGCATACAGAGTAAAATCTAGTCCACATGATATCATGGCAGAGGTCTACACAAATGGCCCTGTAGAAGTTGCTTTCACAGTTTACGAGGACTTTGCACACTACAAATCAGGAGTATACAAGCACATCACAGGTGGCGTGATGGGAGGTCATGCCGTCAAATTGATTGGATGGGGAACCAGTGATACTGGCGAGGATTACTGGCTTCTTGCAAATCAGTGGAACAGAGGCTGGGGTGATGACGGGTACCTCGGCCGCGACCACGCTAGTCGTGGTGTGGTCGGCGTCT

>Isogroup_308

TTCGATAAAGACGGCAGTGGTTACATTACTCAAGATGAACTGCAAAAAGCCTGTGAAGAGTTTGGTATAGAAGATGCACATCTTGAAGAAATTATCCTAGACATTGATCAAGACAATGATGGCCGGATCGACTACAACGAATTTGTAGCAATGATGCAGAAGGGAAATAATCCACTGGGAAAAAAGGGACAAGGGCAGATGAGCTTTGGTCTTAGGGAAGCATTGAAGATACGCTAATGGTAACTGGTCTGTGCTCTTCTGAGTGTTCTCCATTTCTGTTGGATGGTTTATGTAGCGCCTTACTCCTCATCAGTTTATCTTTCAACAGTCCATCGTTTGCTCCATGTGACCCGGTCATTTGCTATGCTAATGATAGCATGGCCCGGAGGACCTTCCCCCAATGATCTCGACAATTCTTGCTCCAGCTAACTAACGCAGGGTAGGATTGCTAAACTCTGAAGTCAACCAAAGAAGGAATTCTCTGATGGAGTTAGCAAAGAGAGATGCTAGATGGTTGTGCCTTTTTTGTTCAGATCACCCTGCTTGTAGATTGATGTGTCTAGTTTTGGCATTTTGAGCGATGAGTGAGCTGCCTGGATTTTGGGGAACTACATGTGAGCATGGAGTAGAGGACACCCAAGGTTGTGTTCAGTGACGGTGTGAGTAAAAATATGTAT

>Isogroup_309 CATTATTGATCCCGACAAGGAGAGATATCGCACACTCCTGCAATTTCAACTGCCTATTTTACTTGTTCGCACTGCTGTTGGGGCTGCTCACCTTGCCAAGCCCAAACAATATCTTTCAAAGCTGGCCTCATCTCATGTCTAACCTTCTCATACTCAAGAGTATCACCATTGGTCTTCTTGATTTCCACCACATGGAAGTTTGGCGTGATCTGAAATACCTCAGTATCGAGGGACATTACACCTTTCCTTCCTGGTTTTAAACTCTCCATCTTCAACAATCCGCCATCTTTTTTCATGAGTTTTAGCCGCAAGCTCTTTGCAATGTCCTCGAGCTTTGTGATGATGGTTGCAGCTGTGCTGGTAGATGTAAATTTTGACTCCTTATTTGACTTATCCTCAAACATACCGGAGAGATCAAACCCAGATGAAAGAGAAATTATGTCAAAAGCATTCAAGTTAGTGAGTTTCTCTTCTTCTTCTTCTTCTTGTGTCGCCTCGGTTGCAAATGGATCAGAAACCAGATCCATGTCAACGGGAACACCATCCTCTGTCTGTGTGCTATAACTAATTAGCTCTGAATCAAGGCCTCTTCTGAACCAACGATTTTCCAAGATCTCTGAAAATGAGATACGTGTACCTCGGCCGCGACCACGCTAGTCGTGGTGTGGTCGGCGTC

>Isogroup_310 GACTATGTGCCTGGATTGAAGGTCGACGGAATGGATGTTCTTGCGGTGAAGCAAGCATGCAAATTTGCGAAGGAGCATGCCATTGCAAATGGCCCAATTGTTCTTGAAATGGACACCTACAGATACCACGGCCACTCTATGTCTGATCCAGGAAGCACTTACCGCACCAGGGATGAGATCTCAGGTGTCAGACAGGAGCGTGATCCAATTGAGAGAGTTAGAAAGTTGATCTTGGCTCATGACCTAGCAACGCCTGCTGAGCTCAAGGACATTGAAAAGGAGATAAGGAAAGAAGTCGACACTGCTATTGCTAAAGCCAAGGAAAGTCCTATGCCTGATACTTCTGAGCTCTTTACGAATGTATATGTCAAGGGTTTCGGCGTGGAGTCATTTGGCGCAGACAGAAAGGAGTTGAGAGCTACACTCCCATAGGTCGCCAACCTGAAGAAGGGCACTCTTCTAATACGAGAGATGTCGAGGAGAAATAAGTGCTGCTGAACAAACGCTTGGGCATTGCTGGCAGTTTTGTGCAAAACGAACCTGTATAGAGATGCGATGTCAGGAGGATCTGCTCCTTATTTGTTTTGCTTCTTTTTCTTTTTTAGCAATTCATTTAAACTGCTGCTGGTTGGCTTCGTATTGAGGCTAGTGATTTGACCTGCATAACCGCTTCTGT

>Isogroup_311 TGTGGAGCCTAGACCTGGTGTTTTGCGGCTGATGGATGAAGTTAGGGGTGCGGGCATCAAGCTTGCTGTTTGCTCTGCGGCAACTAAAAGTTCTGTGGTTTTGTGCCTTGAAAACCTTATTGGACTTGAACGATTCACTGGTCTGGACTGCTTCCTCGCCGGCGATGATGTTAAACAGAAGAAACCTGATCCATCAATATATGTTACAGCAGCAAAGAAATTAGGTGTGGAAAGCAAGAACTGCCTTGTAGTAGAAGACAGTGTCATTGGACTACAGGCAGCAAAAGGAGCAGGGATGTCATGTATAATAACATATACACCTTCGACTGCTAACCAAGATTTCAAGGATGCCATAGCAACCTATCCTGACCTTAGTAATGTGAGGCTTGAGGACCTCAAGCTACTGCTCCAAGAATCTCTTGTCACTGGATAGTGAAGTTTCTTGCTTTCCTCAGGGAACACACTGACTGAATTTTTTATGCGAGATACACCATGGTAAATATTCAAAGCTTCATAATTAGGCATCTTCGTTTGCCCCCCTGGCATCTTCAGATCAACTGTAATATTTGGAAACGGAAATCAAGTGAGGACATTCTGATACATTCTTTGGGAATCTCAGCATAGTTTGTAAACGAAGGTGGATTCAATATTCGTTTCAGCTGAAGTGGATGGCGG

>Isogroup_312 AATCCCATAACAATGGAGACTTTTTCTACATTATACAATCTATACAATAGCAGAACAATGACATCTGGTGCTATCTTGAGTTGTCCTTTGAATCTTCTGGTATGGAGGCGCCATCTGAAATCCTTGCTTCAGATTTCTCGTTTGAGTAAGTTGACAAGATTGTGACCCAGATCAGTTCCACGCAGTCGACCCAGAGGAGTCTTTGTTCAAGGGGGACTACACCGTATGTAATTAAGTGTGCAAAAGGCCATAGCTTCCATCCAGCCTGTAATTTACAGTTCAATACAACAACATTTATGTCTTCTTCATGTCCGGAAGCAAGATAAAGATGAACATAGAATAAGATTGATCCACGAGCACTCCAATAACATAAGACATGATCCACTTAGAGTTTACATTTGAGTTACTTTTATCAAGTGACCAGTAAGCATGGGAAAGAAGGTGGACTTGAGCTCGCTGAATATGGTGGACGGAGACTCCCAGCGAAGGAAGCCTAGGGTGACGAAGTAGATGCTGTTCCATAGCGCAGACCAAGCAGTCTGATCAAACGCAACCTTCACTGGAACAGCCCACCAATCCTTGAACGGGAACAAGGACTCGCAGAAATGGTAGTAGTAGTGTGAGAGGGATCCGTGAAGCATGAACCCTACGAGGCCGGACCGGAACATGCGGG

>Isogroup_313 GGCCGCCCGGCAGTACTGAAGTAGAGGAATAGCCTCTGATGCAGCAACTGATTGAGCTTTAAATACTTCAGCTGGGCAAGCCGTCCACGCTTGCCTGAGCAGATATAGTGAATCCAGTGCTGCTTCTTGAGTTGCTTCAGAACCAGTTTTAAGGGACGTTACAAGATGCGGAATGCATATTGTAGCAGGTTCAGTGACTCTCAGACGAGGGAAGTTGCTAAGTAGAGCATTAAGCGCTTTAAGATACTCCTCGTTAGCACTCCCAGATGCCCATATATCCTTCTCAATTGAAGCAGTTATAACTCTGACTGTTTCACTCGTCGCATATTCTTGGATAGTGTGATTGTTGAATAGAAGTTTGACAAACATTGCTGCCTGAACAGAAGTGTCCGGATTGCTTGAGCTGATAAGGTCCAGCAAGACCTGGACACCACCAGATTCTGCAACTGCTCTTTTGTTTGCTCGGCTATACATGACTAGATTCTGCAAGGCACAAATGGCAACCACTTTCATCTCTTCAGTTGGCTGGTCTTCAAGAAGATTGACCAGAGCACGGCATGCTGCAACGGCATCAGTCGAACGAGCAAGACCTTCATTTTGGAAAAGATCTCCAAGAGCAAGAGCAGCAAGCAACCTTCCCTGTTGAGACTGTGTTTGTGGATCCAGAAGG

>Isogroup_314 ACGACCACTGCCTCAGCTTCAAACCTTCCCTCTTCCCGATAGTCCTGCTTCAACTTTCTTGATTTCAAATATTAGCATCCTCCACATCTTAAGGACAAACATTTCAGCCTCAACATCCAAAATTGATTGTAGGAGCTCCAACATCTTCGACGCATGCACATGATCTTTGGTGCATGAGACTATATATTCCACCAAAGTAGATTCCTCTTCACCAAGAAACTCGATTATCTTTTTGGAGATCCAAGGCCTCATTCTATCATGCAACTCATGCTTCTCATATACTGCCCAATTAACATCATAGGAAAAAAGCTCTTCCTTTGTCCTCGGAATCATATCAATCAATTGTTTTGCATCCAAAATTTTCTTGTTCTCTGACTTGGGCTCATGTGTCTTCTCCCTTCTCTCATCACTAGTGCGAGCACCATCTTCCTCATTTCGACTTTTATCCCTTTGGCTTGATCTATCAGTGGATCTCCTACTCCTATCCTTCTCAGTTTCAAGCTTCTCTTCCTTCTGGTTAGATACCAAGATGCGCTTGGCAAACTCAGCAGCTGCAACAATATTCTGTTGGCCAGCAGAAGGATCTGCCTCAACAGCTTGTAATTCCTCTGTTGAGTAATCAATGGGTACCTCGGCCGCGACCACGCTAGTCGTGGTGTGGTCGGCGTCTC

>Isogroup_315 CATGGATACCAGGTTTACATCAACCCAATCTATGAAATTGTTTACAGGGATAAGATACAACAACGGAACATGGTGTAACTACAAGCATTTCCATCCTCTCCAGAACCGTTCCGTCTGATGAGCAGTTTTAGATGGGGAAGATGCCACAGTGTGTTGAAGTCAGTCTTTGAGTAACACCTCCTTACAGATCGAAAGATGTTCCATTTGGCATCCTGCCCTTCCAAACTTCTGAGAACCTCTTCTGCATGTCGGCGAACAATTAGGCCAATTGGTGGAACCATTGGCCTCTTCAACACAATTTAGGAGAACCTCTCATTGAAGATTCCTGCAAGTTGGGCAAAACCATTTCCCCTTGAATCTCGTTTCTGGTGTCAGACCAACGCACGAGTAGTGGAACCATTCGCCTCCTTCACAATTATCGTTGTCGCAAGCAATCATATCACCATATGAAACCTGGTGGCATATACAGTATGTCGGTTCATTTGGATCAATCATTTGCTCCACATCCATAGATGCAGCTGTTTTCTTGCTGCTGCCAGGAGGGGGCATTAAGTCGAAGTCCATACCCCTTTCCCTTTCCCTATCCCATTCCCTCTCTCTGAACTTCTTTGTTGCTTGGGGAGTACCTCGGCCGCGACCACGCTAGTCGTGGTGTGGTCGGCGTCGTCTC

>Isogroup_**316** AGCGGCCGCCCGGCGAGTACTCTGCCTTCTTCCTCTCCAGCTGCTCTTGCAGCTTCTTGTGCTCGGCTTCCAGCTGCGCGGCCTTGGAGTTCTCCCACGAGGTGATGTCCGCTAGCAACTTTGCCGTCTTGTTCTCGACTTTGGCCTTCTCGTTCTCCTCCCATGCCTTGATCAGCGAAATCCTCTTCTCGGTGGCGACCCTCTCGAGGAAAGCGTCTCTTCCGTGTGAGCCCTCCTCTGTAGCTGCAGGCTTCTCATCATTCTCCGCGACAACGGGGGCCTTCTCCTCGGCGATGTCCTTGGCCGGCTCAGGCGCGGGCGCCGCCACCTCCTTCTTCGCTTCCTCCGCCATCTGATCGACCTCGCCGCCTCTGCAACGAACGACTCGACCAGGATATTTGGCAAGACTCAAAGTCGCAGGAGCTCAGGATATGTTGTGTTTGTGCTGGTAGGACGTGGTGGTCAGGAAACTAAAGCCAAGAGTATGTATAATGTAGCGGTCGGGAGAAGGGAGAGCGCATGGATCGATCAACTAATAAACCGGCCGGTGAGGTTGGCTCAGGCTGGATGTATCCAGATTGATTAGTGTAGCCGTCGTCTAGGTAAGGTGGTGGCACACCTTCTCTTCCGGCTGTCGCGTTATTTCTTCTCAGAGACGTAAGCAACTCAC

>Isogroup_317 CTAATACGACTCACTATAGGGGCTCGGCGGCCGCCCGGGCAGGTGTCGAAATAACAGTCTCTGATTACTTTCGGAGGCACAAAGGCATAGAACTCCTGTATTCTGGTAATTTCCCCTGTATCCATGTGGGGAGGTCAAAGCGTCCGACGTATTTCCCTGTGGAGCTTTGCACGCTTGTTCCTCTGCAAAGATACACCAAAGCTCTGTCTACTCTGCAAAGGTCCTCACTTGTCGAGAAATCAAGACAAAAGCCTCAAGAAAGAATGTCAACCCTCAATGATGCACTTAAACGTAGCAACTATGACTCTGACTCCATGCTGAAGGCATGTGGCATTTCAATTGAACCAAAGTTCATTCAAATTGAAGGGAGGGTCCTGCAAGCACCCAAGCTGAGAGCCGGCAACAGTGAAGTGAAGATGTGTTGCCTCGCAATGGACGTTGGAATCTTGCAAACAAGGCCTGTTCTGTCAAGAGGTGGTCGGTTGTTAATTTCTCTGCACGGTGTGATGTTCGGGGTCTTGTCCGAGACCTTAAAAGGCTTGCAACTGCAAAGGGAATTGAAATAGACGACCCTTATACTCTAATTGAAGAGATCCCATCAATGAGACGAGCGCATGTGTCAGACAGAGTGGATAAGATGTTTGCTGAGATAAAATCCAAGCTACCTGGA

>Isogroup_**318** AGCGGCCGCCCGGCGAGTACGACATGCAGACATAAACGATGGCAACTCGGGTTAAACTGATCTGGATTCTACTACGGGATCATTCCAGTCAGTCTGGCTTGGAGTCTCCATAACATTTGGCATCTTCTCTACCAAGGTAACTCCCTCCACTTGCACTCACCATGGTGGTCTCCTCGCGATCCTTGCTTTCAAGTCAACGCTAGTTTCAGGGTTATATCAGAACCGTCATCATTGTCTTGCGTCATCACAGATTCAGATGAAACAGCTTCGGTGACGGCATTTTCAGTATCGACAATGGTCATCATGCCAGCCGTTGACACCTGAGGCATTTGGTTTCTCAAGCGCATGTTTTCCTCTGCCAGATGTGTGCCCTTTTGTTGGAGGTCACTGATTTGTTGCATGAACTGGCGATCCTTTGTAAAAAGCAACCTCTGCAGTCCTGTTTCAAGATCCTTTTCCATCTGCTGCAGTTCCCCAACACTGAGTCCCCCAAGTTCCTCACCTCTCATGTGTCTAAGGTGAAGACTTGCTTCTGCGAGTTGTTCATTCAAACTGTTAATCTTGCTGTGCTCTAAATTCAAATCAAGTGCAGGCTGCTGATGTGACTTCCCCAAGTTCTTAGAATGAGTACCTCGGCCGCGACCACGCTAGTCGTGGTGTGGTCGGCGT

>Isogroup_319 CTACCAGCAAGGCGCCCGCTTCGCCAAGTGGCGCACTGTTGTCAGCATTCCTAACGGCCCATCTGAGCTCGCTGTCAAGGAAGCTGCCTGGGGTCTTGCCCGTTACGCCGCCATCTCTCAGGACAATGGGCTGGTGCCGATTGTGGAGCCTGAGATCATGCTCGACGGTGAGCACGGCATCGAGAGGACCTTCGAGGTCGCACAGAAGGTGTGGGCTGAGACGTTCTTCTACATGGCCCAGAACAACGTCATGTTTGAGGGCATCCTCCTGAAGCCAAGCATGGTGACCCCTGGTGCCGAGTGCAAGGACAGGGCCACCCCTGAGGAAGTGTCCAGCTACACCCTGAAGCTCCTCCAGAGAAGGATCCCCCCTTCCGTCCCCGGCATCATGTTCTTGTCTGGCGGTCAGTCAGAGGTGGAGGCGACGCTGAACCTGAACGCGATGAACCAGGCGCCGAACCCGTGGCACGTATCCTTCTCCTACGCACGGGCGCTCCAGAACACCTGCCTCAAGACGTGGGGCGGGCAGCCAGAGAACGTCGCGGCGGCGCAGGAGGCGCTTCTGCTGCGTGCCAAGGCCAACTCCCTGGCGCAGCTCGGCAAGTGTACTGGAAATCAGAATCAAACGAGCTTTTACCCTTTTGTTCCACACGAGATTTCTGTTCTCGT

>Isogroup_**320** GACCACACCACGACGTAGCGTGGTCGCGGCCGAGGTACTGTGTTTTATTTCTCGGAGTGATGGCAGGACATGGGGGCAGATCCAGCATTCCTCCACAAAGTCTCCCGTTGCCCCGAAGTGAGACGGAACTGGCATTTCCAAATACCCCGTTTCTTGGGACCCCACCTTCCAGATTATTATAAGACAGATCCAGCTGGGTGAGCTTCTGTAGCCCACCCAGCTCTCTCGGAATGAAGCCTGATAAACTGTTGTGAGAAAGATTTAGCATTTTCAGGGTTGGTAGGCCACCCAAAGTCACTGGAATGCTTCCCGTGAGAAAGTTTTGACCCATCTCAATTATAGTTAACTCTTGACAATTTCCCAAGCTAGTTGGAATGTCCCCGGTAATCTGATTGGAAGAAAGATATAATTCGTTTAGATGTTTAAGATTACTAATCTCTGATGCTATTGAACCTTCCAATTTATTGTAGGACAGTGAACATATTGTCAGTGTGGAGGCAGTGTGAAAGAGTTCTTTAGGAATGTTACCCTGCAGATTGTTATAACTAAGGTTCAACTCTGCAAGGATCATAAGGTTTCCAATGCTAGGTGGTATCGGACCTTCAAACTCGTTTTCTGCTAGATAGATATTTAACAAGTTAGTTAAATTGCCAATAGAGGATGGTACC

>Isogroup_321 GACGCCGACCACACCACGACTAGCGTGGTCGCGGCCGAGGTACTATCATCCCATCCGGCAAGGCCTCGTCTTTTCCCACCCACCGTCCACAAGATAATCAGGAAAGGAGACGCGTCTATGCCTGGGGTGTGCTTAAGCTTTAGCAGCACTAGACAAAGAGGTGTGTGCATGCCCATGGGTGTGCTTAAGCTTTAGCACAGCTACTCTTGAGGCTTCATGGCCGGGAATAGGAGGACTTCCTTGATGTTCTGGGAATCTGTCAGCATCATTGTAAGGCGATCAATTCCCAAACCCCAACCACCTGTTGGTGGCAGCCCATACTCAAGGGCAGTGCAGAATGTCTCATCCAAAGCCATAGCTTCATCATCACCAGATTGACGATCCTTTAGTTGTTCCTCAAACCGCTGCCTTTGCACAACAGGATCATTCAACTCGGTGTATGCATTGCAAACCTCATGTTTGTTAACAAAGAGCTCAAACCTTTCAGTCAACCCAGGCCGGGACCTATGCCACTTTGCCAATGGACTCATTATCTCTGGATGGTTGGTGATAAATGTTGGATTTACACATGTCTCCTCCAAGAAATGGCCAACCAGCTTGTCAAGCAACCGTGTTGTTGTCTGGGGAGGTGGACATTTAACATCATACTTGGCACAGGCATCTATTAA

>Isogroup_**322** TCAACACATGTTCCAACCTAGAATTTAGGGCACTTGTGCTTCAATACATGCCAAATGGTAGTTTAGAGACGCTACTACATAGGTATCAGAGTATGCACTTTGGTTTCCTCGAGAGGCTCGACGTGATGGTTGATGTGTCGATGGCAATGGATTATCTACACCATGAACATTACGAGGTTATATTGCACTGTGACTTGAAACCTAGCAACGTGTTGTTTGATGAGGACATGACTGCACATGTGGCGGACTTCGGCATTGCAAGGTTGCTTCTAGGCGGGGACAATTCCATGATTTGTGAAACCATGCCTGGAACAGTTGGCTACATGGCCCCAGAGTATGGGTCTCTTGGAAAAGCATCACGGAAGAGCGACGTATTCAGTTACGGGATCATGCTCTTGGAAGTCTTTACCAGAAGAAGACCCACCGACTTTATGTTCGGGGGAGAACTAACCCTTAGGCAGTGGGTTCAACGCGCATTCCCTAGGGAGCTTGTCCATGTCGTTGATGACCAGCTACTTCAACGCATGGCCACTTCTTCTAGCTACAACCTGAATGACGGATTCCTTGCATCGGTATTTGAGGTGGGTTTGCTTTGCTCTAGTGACTCGCCAGACCAGAGAATAACAATGAGTGACGTGGTTGTGTGTTTGAAGAAGATCAAAGCAGGG

>Isogroup_323 CTCCAACCCACGGGCAACATCTAGAGCGATGGATATGGTCTTCTCTAGCGGAATGGGGTGGTGCTCTGTGCTGTGCAGGTATGACCTCAAGGAACCTCCAGGAAGGAACTCGGTGAGAATGTAAAAAACCGGTTGGCATTTATAAGCCGCTACAAGCTTGATCACATTCTTATGATACAAGTGAGACAACGCATTGATTTCGGTGTTATACTGTCTTTCAATCATTGCGGCCATTTTTCCGTTGTCATCATTATCAGGCTGGCGTATGAACTTGATGGCAACTGGTTTATCATCATAGACACCTTTATACAGCCGGCTATATGCACCAGAAGCAAACTTCAGCCCAACAAGCAATTTGGATGGAATGAGTGTCCAATCAACCGTTGTTTGCACCGCCTGGCCCATGCTAACTCTACGTCCCCTCATACTATCTACTGCACTAACCTTGCTTCTTCCACCATTTTCAAAGTATTTCCTTGTATCAGAAAATTTCTTACCAAGCGATTTGCCATAGACACTCTTTTCAGATCCCTCTCGCTCTGGGGGAGGGCTTGTGAACCTCTGGCTTCCAGCTCTAGCTTCCCTGAATACTTCCGAAGGCACTACACTAGGAAGAGGGGACTTCGATCTTTGCTTGTGCCGTGAACCATCAACCTTCTGTACCTG

>Isogroup_324

GTTGAAGCAAATGAGGGAAAACCACTGCCTCTAGTCCAGAAAGCTTTAATTGGTCTCACTGCTGGAGCAATTGGAGCATGTTTTGGTAGTCCTGCAGATTTGGCACTCATTAGGATGCAGGCTGATTCAACCTTGCCAGCAGCCCAGCGCCGGAACTATAAGAATGCTTTTCATGGAATTTACCGTATTATTGCTGATGAAGGTGTTCTGGCACTTTGGAAAGGTGCTGGTCCAACTGTAGCTAGAGCCATGTCGCTCAACATGGGCATGCTTGCCTCCTATGACCAGAGTGTTGAGTTACTTAGAGACAAACTTGGGGCTGGAGAATTATCTACAATGCTCGGGGCCAGTGCTGTTTCAGGATTCTTTGCATCTGCTTGCAGTTTGCCCTTCGATTATGTGAAGACACAGGTTCAGAAGATGCAGCCTGATGCCACTGGAAAGTATCCATACACTGGGTCTCTTGACTGTGCAATGAAGACCTTGAAGAGTGGCGGTCCATTTAAGTTCTACACTGGCTTTCCCGTCTACTGCGTCAGGATTGCTCCACACGTCATGATGACTTGGATATTCTTGAATCAGATCCAGAAGGTCGAGAAGCGTGTCGGCCTTTAAGAACTGCATTATGCCATCCCTGCCTAAGAAATAATAATAGTGTGTCTTCTTT

>Isogroup_**325** TCTGGCAAAGGATCTTCCTGGGACATTGTCATCAAAGGCTCAAGCTTTAGTCGAAGCACTGGAGGGAAAGCGGTTTGATTCATTTCTGGACGCCTTAAGAGATATAGCAGAGGAAAGTGGCTTGTCATATAAGAAGCTTGATAAAAGATTAGAGCGATCATTGCTGCATTCCTACCGCAAGGATTTGATAGCACAAGTTTCTTCAGAGAGTGATCCAGTTTCCTTCCTCCCAAAAGTTGTTGCCCTACTTTTTCTTCAGGCATACAATAAGGCTCTTCAGGCACCTGGAAGGGCTGTTGGTGCTGTAATCACAATACTGAAGGACAAGCTACCAGCTTCAACCTTTAAGGTTTTGACTGATTATCATAGCACTACCGTGAAAGTTCTCTCTCTGCAAGCTGCTGCTACGGGTGATGAAGATGATTGCACGTCGGATAGATTGAGGGAGAGGAAGGAAGATCTGGTGGAGATGCTGATGCCGGAGCTGAAATCCCTAGCCCTCGGCACAAGTAAAGAGTGAGCGACAAAGATTGTCTGGATGCCGCGCCAATGCCCGTGTAAATACACTGCGCTATATGTCCTCGGTGCTGGAGCTAAGGGTTGTAAGCAGCCGGCCTGTGATACTTGAGTCAACTTGGTGCAATTTTTCACTTGAATTTATG

>Isogroup_326 |ACYL- N-ACYLTRANSFERASE ISOFORM 2

AAAGGTGGAGATCCGAGTCTTTGAAATGACAATATTGCCTCCTAGGGGCAAGGGATCACCCGTATTCCTACTGCTACTTTTTTTGGACATGGAATCCTACTGCTACTTCTGATTCATGTAGTATGGTCATATGCATAGCTACAAGTTGCTTTCCTTGTTTTACATTTCATTGTCTAGTTGAGGAGCAAATTCTTTCATTATGTCTTCCTTGTATGGACCTGAATCACGCAGGACATTGATGACATGCTTAACCTGAGCTGTCTCATGCACAAGAAAATTTCCTATAGCTGTCGCAAAACCAGGTTCTAAGAAGTAGTGGCAGCTGTATGTGGTCACTGGGAGGTAACCACGCTGGATCTTGTGCTCTCCCTGAGCACCTGCTTCCACCCTACTCAGGTTTAGTTCTATGGCTGCTTCAATTGCCTGGTAATAGCAAGCTTCAAAATGCAGATTGGGGAAATGAACATCTGGTAGACATCCCCATAAGCGGCCATATAATGTATCGCCTCCAATAAGATTAAGAGCTCCAGCAACTAGTTTATCATCATGTTCAGCAACAATAAGCATTACCTTGTCACCCATCTTTTCTCCCAAAAGGTGAAAGAACTCCCGTGTCAAGTACCTCGGCCGCGACCACGCTAGTCGTGGTGTGGTCGGCGTCTCTC

>Isogroup_327 CAGGTACTGTAGGAAGTGCAACCAGCTGAAGCCGCCTCGGTGCCATCATTGCTCTGTTTGTGGAAGATGTATCCTTAAGATGGATCATCACTGTGTATGGGTTGTTAATTGTGTTGGGGCGCTGAACTATAAATACTTTCTACTCTTCCTGTTCTACACCTTCCTCGAGACAACGCTTGTTACCCTATCTTTATTGCCTCACTTCATAGCCTTCTTCAGTGATGTCGATATCCCAGGAAGTCCTTCAGCACTTGCAACCACATTTCTCACATTTGTGTTGAATCTGGCGTTTTCCTTGAGTGTTCTTGGTTTTATGATAATGCATGTTTCACTAGTTTCTGCTAATACAACAACGATTGAGGCATATGAGAAGAAAACTAGTCCACGTTGGATGTATGATCTTGGCCGGAAGAGGAATTTTGCTCAGGTCTTTGGAAATGACAAGAAGTATTGGTTCATCCCCGCATACTCAGAAGAGGACCTACGAAGGATGCCTGCTCTGCAGGGCCTTGATTATCCTGTCAGATCAGATTTGGACGGTCAAGAATTGTGACAACACGGAATTTTCCAGGGCCCCTGACTGACATAGCAGTTTGGTCGGGCAAAATGTTTGATGTAAACTTCGATAGTTTCCTTAACAATATGCCTCGGTGCTCAACATCTT

>Isogroup_328 CCGAGGTACTGTCCACTTGATCATCTTGTCATGAACCAAAACGAGGAACTATTTACTGTAAGCAACAGTTCCCTGTCAGAATTTGCCGTTCTGGGCTTTGAGTTGGGCTATTCAATGGAGAACCCGAACTCACTGGTTCTATGGGAAGCACAGTTTGGTGATTTTTCAAATGGAGCTCAAGTGATATTTGATCAGTTCATTAGTAGCGGGGAGGCAAAATGGCTCCGCCAGACTGGGCTTGTTGTTTGCCTTCCTCATGGATATGACGGTCAGGGGCCTGAACATTCTAGTGCAAGAATGGAGCGCTTCCTTCAGATGAGTGATGATAACCCTTATGTTATACCTGAAATGGACCCAACAATGAGGAATCAAATCCAGCAGTGCAACTGGCAGGTTGTGAATGTCACAACTCCTGCAAACTATTTCCATGTTCTGCGTCGGCAGATACACCGTGATTTCAGAAAGCCTTTAATTGTGATGTCTCCGAAGAACCTCCTTCGTCACAAGGACTGCAAATCCAGTCTATCTGAATTTGATGACCTTGCCGGCCACCCTGGATTTGATAAGCAAGGGACGCGCTTCAAGCGTCTAATAAAGGACCGGAATGACCATAAGGATCTCGAGGAAGGAATCAGACGTCTAGTTCTTTGTTCTGGAAAGGT

>Isogroup_329 GAGACGACGCCGACCACACCACGACTAGCGTGGTCGCGGCCGAGGTACTACTTCAGCGATGAGAACCTTCCGACTGACGATTTTATGTTGAAATTTGTGAAAAAGAACAAGGATGGCTTTGTTCCAGTTGGAGTTATTGCATCATTCAGAAAAATGAAGAAACTTGTTCAGGACCACTCTATAATTGAAGCTGCTCTTAGGACATCATCGAAGCTGGTTGTGAGCTCTAACGGGAAAAGGGTTAGAAGGCTACATCCCTTGCCATGCAGTGAGTTGCAAGACATGACGAAAAGGACTGTTCTGGTGGAAAATCTACCTCTAGGTTTCTCCATGGAGAGTATACAGGAGAAGTTTGGAACAGTAGGCAAAGTTATGAAGATAACCATTCATGATCCTCACGCAGTAGGAGAATGTGCAGGATCCAAGAAGCCTGACTTCATGCTGAGTAACAAGGTGCATGCCATTGTGGAGTATGAAGCAGTGGAGGCAGCTGAGAAAGCTGTGACCACCTTAAATGATGAGAGGAATTGGAGAACTGGGATGAGAGTCATACTTCTGGCTAAACGAAGTGTAATGGGATCAGGAAAGCATGTCCAGTCTTCAAAGGAAAATCATGGAACAGTTTCCAAAAAGAAGAATGAGGGCCAGTCTTCAAAAGAG

>Isogroup_**330** TTGAGGGACGCCGACCACACCACGACTGTCGCGGCCGAGGTACGTTGGGTTATCCTCAACCGGATCATCATATAGATCAAGCTCAGCCACTGTAGGGAGCAATTACAGTTCAGGTGAACGTTATGGGAGTTTTGGTGGCACAAGGGAGGGCGATTCGTTCAGCAACAGTTACAAGGACAAAGAATCTGCTAAAACCTCTGCAGGTAGTAATGGCAGCAAGAAATCTGGCAGCAAGACAAGAAAAGATGCGAAGCATGATAGAAGCTCCTCGAAGCCACCATCTACTGCAAAAAGCAATGAAGATGACTTCGATGACTTTGATCCCCGTGGATCTTCTTCAAATGATGCAGCTAACAATGCGAAGACTAGCGAGGTCGATCTTTTTGGCCCAAACTTGATGGATGATTTCATGGACGAGCCTGCAGCCACTCCAGCAACAAAAGGTGTTGTAGAACCTCAGGTTGATCTATTTGGTGATGCAGATTTCCAATCTGCAACCCCAAGTGCAGAAACTGCTGCGCATCAGGATGTCCAGGACAATGTTGACCTTTTTGCGGGAAATGCAACCTTTGCTTCAGCATTTCCATCACAGACGGGGTTTATTCCACCACCAAGTTCCGGGACATCTTCTTCTGCCAATAGTTTTGTGTCCAAGAAGACA

>Isogroup_**331** TATGATAGTGGAGAGGTTTCGCAATTTTACAGCGATCTTTCACTTTTCGATCTTGATGGCAAGGAAGTAATGCGAAGAACTATCAAAGTGAATGATCCCCTTAGGTATGGTGGGATCACTATATACCAAACAGATTGGGGATTTTCAGCACTACAAGTGAAGAAAAATGGTGAAGGCCCTTTCAACTTGGCCATGGCCCCCTTGAAAATGAGTGGCAATAAAAAGCTATACGGAACATTCCTGCCACTTGAAGACTCTGATCCTTCTAGTGTCAAGGGAATATCAATGCTTGCCCGAGATCTGCAGTCTATTGTGTTGTATGATCAAGATGGTAAGTTTGTAGGGGTTCGTCGGCCAAGCTCAAAACTCCCCATTGAAATCAATGGTAATGAAATACTAATTGAAGATGCTATTGGCACTACTGGTCTGGATCTTAAGACTGATCCAGGAATTCCTATTGTGTATGCTGGATTTGGCGCTCTCATGTTGACGACCTGCATTAGCTATCTTTCGCATGCTCAGTTATGGGCGCTGCAAGACGGAACTACAGTAGTTGTCGGAGGGAAGTCAAACCGGGCCAAGATTGAATTTTCTGATGAGATGAACCGGTTACTTAATAAAGTACCTCGGCCGCGACCACGCTAGTCGTGGTGTGGTCGGC

>Isogroup_332 TACTTCTTATGCGTGTATTAATGCACAAGAAAGAGGTATACTATTTGTCAAACCAGGGCAGGATGTTTACAAAGGCCAAATAGTTGGTATTCATCAACGACCAGGTGATTTGGCGCTTAATGTGTGCAAGAAAAAGGCTGCAACAAATGTTCGGTCCAACAAGGAAACTACAGTGGTTCTTGATGAACCTCTAAGCTACAGTCTGGATGACTGCATAGAGTATATTCAAGAAGATGAGCTAGTTGAGGTTACACCACTGAGCATCCGCATGTCCAAGAACCCAAAGGTTTCGAAGAAGGGCAAGAACTAGGCAAAGTTAGTCCTGCAAGGATTCTATGCTATTGGAGCATTCAGGAAGCATCGGTTTCAGGTAGAGCCTGGCTGTTGCTTTGACTGGTTTTGATAATAACTCCTATACATGGTGGTCTACAGTTTCGAGATATAGGATTATACTAAAATTTGCAATGAGGCCGTGATAGTAGTTTTGGCCGCAGATAAGCTGTATATGTTTTTTTCTTCTGTGCAGCAAATTCAACTGTTACACGTGTCGCATAGTCGCTGTGCAATTTTGCAGTTTTACGACCTTGAAACAGACTTCATTGGACAGACGGGGTGTACCTCGGCCGCGACCACGCTAGTCGTGGTGTGGTCGGC

>Isogroup_**333** CTATCCACCTGGACAGCCAGATATTCTCCATTGTTCTGCCAATACATCTTACAGTCGCTAACACTGAAAAGGTTTTTCTGCCGTATCTCCTCTTTGCCAGGGATTTGGAAAAGACTCACCCTGGCAGGCTGGTTTCCACCACCCAATTCAGGCACAAATAGTGATATGATAGGATCAGTGGGCGACCAACTGAAGTCCACCACATTTTCTACCTTCAAGGATTTCTTATCCAGAAGAGAGAATGTCTCGGTCTCATAGACAGATATAACATTCTTCCCAAGTCTAGCAAAATATTTATCATCTTTTCCGCCACCCCACCTGAAGACAGGCCATGAAACACCAGAAACACCCATACTTCCACCAGTAGTGAACTCATCAGCACTTCCCTTGAAGTCACGCATTACTTTTCCAGTCCTCACATCAAATATATTCAGCACAACCCTATGTGTGTCACGAGGGTTGCTGGGCTCTTGGCTGCTATATGTGATCAAATATTTCTCACCGGGAGAGAAATCAATCAGTTTCACCTGTGGATGAGCAAAGCGCATTAGACGAACAAACTTATCATCACCACCCCACACCTGTGCGCCCTGCCTATGAACTGTGGCCAAGTTTGTTCCAAGAGGGGACCACTGAATGTAACTATCCGTCCAGTACC

>Isogroup_334 CAGGTACAAGGCTACGATTGGTGCCGATTTCCTCACCAAGGAGGTGCAGTTCGAGGACAGGCTCTTCACTCTGCAAATATGGGATACAGCTGGCCAGGAAAGGTTTCAAAGTCTTGGTGTCGCGTTCTACCGTGGAGCAGATTGCTGTGTTCTTGTTTATGATGTTAATTCTATGAAATCGTTTGATAATCTGAATAACTGGCGTGAGGAATTTCTGATTCAGGCTAGCCCATCAGATCCTGATAACTTCCCTTTTGTTCTGCTGGGTAACAAAGTAGATATAGATGGTGGAAACAGCCGTGTGGTCTCTGAGAAAAAGGCGAAAGCATGGTGTGCCTCGAAAGGGAACATCCCATACTTTGAGACTTCTGCCAAGGATGGATTAAACGTGGAAGAGGCTTTCCAGTGTATAGTAAAGAATGCGCTGAAGAATGAACCAGAGGAAGAACTGTATATGCCTGACACCGTGGATGTGGTGGGTGGCAACCGGGCTCAAGGTTCAGCAGGATGCTGTTAGGACACGAGGGCCACAACTGTTGGCTATCCTCTTGACATACTGTATCTCCATGGTGACACCGTATTTCATTTGTGACCTTTTTCAATCATCACCTCCTAAGTACCTCGGCCGCGACCACGCTAGTCGTGGTGTGGTCGGCGT

>Isogroup_335 ACGCCGACCACACCACGACTAGCGTGGTCGCGGCCGAGGTACATTAACAATATCACCATCCGCTAGAGACTCAGCAGCATGGGCAAGATAATTCATTCTTTTAACTCCACTGTCATCCTTTCCGACAGCACTAGGCCGATAATTGATATAATTTTCCTGTATGATCAAGGGGACTAAATCAGGATCGCTCATGCTCAGATCGATTCTCTCATCCATCCTTAGTCTCCCGCCATTGAAACCAAACAGCTTGTCAACAGCCGTAAAGGGAGAGATATCTTCATCTTTGGAACTACTATTAAGACGCAATCTTATATCATCATATTTGACCACAGACTGGGAGAGGCTCATGTATTGCAAATGGTTGAGTGCCATGCGAATATCCCCATGCACTCTTTCTGCAAGCTCTTCCATCGCATTCTCTTGAGCTTGAATACCTTCTTTTCTGGCAATCTCCATCAACCTCTTACCCATCTGCTGCTTTGTTGGTTTCCTGAAGTTGAGCAGCAAACAGTAATTTACAAGGCTCTTCAGTTTCTGGCTATAACGGTCATTGCAAATGCAAACTATAGGAATCTTGGATATCTTGATGCTGGCAATAAGATCAGCAACTCCACCTCTATCACCAGCAGACATACCATCAACTTCGTCCATGATGAC

>Isogroup_336 TTGAAGCGCTTGAGATGAATTACCAGGATGTTTGGTGGTTTCTTTATCTTCATTCTTTTCTGCGCTTCTTGTAAACTGCAGCATTTGTCACAGAAGAACTTATCCTCGGCATTCAAAGTTTCTGTTGAACTGAAGTTTTTAAGACAGCTGGTGATTGAACTATTCTGCTCTATATCCAGGCTAAGGTCGAGAAATGTTTCATCTCTATCAGTGACGGTTTCACATCTCAGGCATCTTGTTTGGTTAGTCAATATTCCCTGGAAACATTTGTGGACCCAAGTAGTATCCGGTTCTTTATGACTACCATTTGCTTGACCATTAACAGGACCATTCAAATTCTTATTCGAGGAGGAATTTTGAGGAGGTTCTTTAGCAGCATTACATTCTTTCTCAAGAATGTCAACTAGCTCATTAAGCAAAAAATTCAGAAATTCATGAGCATCCTGATGCATATAGCTGCGGAAAAGCTCATTCTGTTTCTTTAATCGTTGTATAAAACGCTTCGGAGCAATTACACCTGTTTTCTTCTTCTGGTTGCTGATCTGAGAGAAGAGGTCAGCTAAACAGGTTAACATGTTCTCTTCACCATCTCCGGCGCTTTTATTGTTAGCATAGTACCTCGGCCGCGACCACGCTAGTCGTGGTGTGGTCGGCGTC

>Isogroup_337 AAAGCTTACTGTTGAGGATTTCCAGCAGGAGTTGTCATGCAGCATTAACATCAAGCACAGGGAAGAGTTTGACGAGGAGAAAGAGCCTGATGGAATGGTTTTGTCTGGATGGTCTGCTCCAGTGGAGAAGCAAGTCAACAGTAATGGGGAGAACAAATCTGAATCTTCTTCATCGAGTGCACATGGCACTGATGATACTGTTGAGGATATATCTGCTAAACCTGGAATGAAGCGCAAGCTGGATGAGATATCAGAGACAAAGGAAAATTGCGGGGCATCCAGTAGTGCTCAGGTTGTTGAAGACAACGATGACCTTACGATGCTTGAGGAGGACCCAATGCTAAAGAAAAAGAGGTTGCAATAGAAGCCATTTCATCTTACTGTTCCAGAGATAACAGCTGTCGGCTCCTGCTGTCATAGCTAACACATTGTTCCAACTTAGCATCTTTGGTAACTTATCTATGTGGTGAATGTTAGGCCTGATGATATTGAGCCACCAGTGACTACGCAGGGACCATTTTTTTGGCTTTAGATACCACGCCATAGAAATGTAATTTGTAGTTTTGCTTGTAGGCACATGAGCAACTAATGCCCTGCTGACAGTCAGCTGTAACGTTGCACTTGTGTTTTCTCAAGAAAGGAGTATTGGTCATCGT

>Isogroup_338 AAGGCAAGGTTGCAAGCAGTCGATTGGAAGTTGCTGGGCTGCTTATAGAATTAGAGAAGGATAGGTTTAGTAAGAGGAGAATTGAGGATGACCTGGAACTGATGTCAAGGAAAGCCAGTTCTCTTAGAGCAAAAACAGAGGCATCATCAGTTCTGGACAAGGTTCAGCAGGAGGTGAACGAATACAGGGGGATATTGAAATGTGGTGTCTGTCGCGACCGACAGAAAGAGGTTGTGGTTACCAAGTGCTACCATCTTTTCTGCAATGAGTGTATACAGAAGTTGCTCAGAAATCGTCAGCGAAGATGTCCTTCCTGTGGATTATGCTTCGGGGTTAATGACGTGAAGTCGATTTATATATGATGCTGCAGTGGCAGCTGCCGTGGGGCATCTAGGACAAGTGACTTAATGCTGCTTGTTTGGTGGCATGTGATCCTCTTCAGCCGGAAATGTAGCTTAACATACCGAATTTCCGCAAGGAGGCACATGCCCAAGAGGCTTATGCTATGCCAGTGTGGCTTCCCGCTCCTTTGTAAAGTGTGGAGTTTTCTTGTTGGAATAGCCGGTAGTCTTAGCTTGCATATACATTCTATAATTTTATTTGACAGATCCATCCAAGGAAGTGGAGTTAGTGGTTCCCAAAAACATACCATAGATT

>Isogroup_339 TTGTGGAATCTTGCATTCAGGAAAGGCCAATTAGGATATATCAAGTATATCCTAAAGAGCAGCTTGATGAAAATACCTGTTTTAAGCTGGGCATTTCATCTTTTTTGAGTTCATTCCAGTAGAGCGGAAATGGGAGATTGATGAAAGAATTATCCAGAACAGATTGTCAAAATTTAAGAATCCCAAGGATCCTCTATGGTTGGCAGTTTTTCCAGAAGGCACTGACTATACTGAGGAAAAATGCATCAAGAGTCAAGAATATGCAGCCGAGCATGGTCTGCCTGTATTAGAGAATGTCCTACTTCCGAAAACAAAGGGGTTCATTACTTGTTTGCAAGAGCTTCGAAGCTCCTTAGATGCAGTTTATGACGTCACAATGGCATATAAACATCGACAACCGGATTTTCTGGACAATGTATATGGCATTGATCCTTCGGAAGTTCACATCCACATCAGAACTGTTGAGCTCAAGAATATCCCCACATCAGAAGATGAAGTAGCTGATTGGATGATAGAGCGGTTCAAGCAAAAGGATCAACTTCTGTCAGATTTTACCATCCAAGGTCACTTCCCTGAAGAAGGAGCTGAAGGAGATATATCCACACTAAAGTGCCTTGCAAACTTTTTTGCAATAGTCAGCTTGACAGGCACCTTCTT

>Isogroup_340 CATACGATGTGAACACAAGAACTGGTGGTGCAAATGGCTCAATTAGATACGAGGAGGAGTTCACTCATGGTTCAAATGCTGGCTTAAAAATTGCTATTGATCTACTTGATCCTATTAAAGCAAAGTGTCCAAAGATTACATATGCAGACCTTTATCAGCTTGCTGGAGTAGTTGCAGTTGAAGTCACTGGGGGTCCAACCGTTGAGTTTGTTCCTGGAAGACGTGATTCCTCGGTTTGCCCCCGGGAAGGGCGTCTTCCTGATGCTAAGAGAGGGGCACCACATCTAAGGGACATCTTTTACCGGATGGGCTTGACAGATAAAGATATTGTAGCTCTATCTGGTGGCCACAGTCTGGGAAAGGCACACCCTGAAAGGTCTGGATTTGATGGTGCATGGACCCAAAGATCCTCTGAAGTTTGACAACTCATACTTTCTTGAGCTACTGAAGGGAGAATCTGAGGGGCTTCTGAAGCTCCCTACTGACAAGGCATTGCTTGATGATCCTGAATTTAGACGCTATGTGGAGCTTTATGCAAAGGATGAGGACACCTTCTTCAAGGACTATGCTGAATCACACAAGAAGCTCTCTGAACTTGGCTTCACTCCGCGGAGTGGTGGCCCAGCATCTACAAAATCGGATCTTCCAACTGCTGTT

>Isogroup_341 ATAACTTATACCACACGGTAGCCCATCACGTGAATTATGGAGATCAGAAGCTCAGTTTGCACGGGCAAGCAGGAAACTCTGTTCAAGTGATTTTTGATAGCGGAAGCTCATATACATACCTTCCAGAAGAAATATATAAAAACATTGTTGCTGCTATTGAAAAAGACTTCCCCAGTTTTGTCCAAGATGACTCAGATACGACATTACCTCTATGCTGGAAAGCTGATTCTGCTGTAAGGCATTTGGGAGATGTCAAACAGTTCTTCAAGCCCTTAAACCTTCATTTTGGGAGAATATGGTTTGTTGTGCCCAAAACATTCACCATTCTTCCTGATGATTACTTAATCTTGAGTGATAAGGGCAATGTTTGTCTGGGGCTGCTAAACGGAACCGAGATCAATCACGGGTCAACAATAATAGTCGGAGATGTTTCTCTTCGCGGCAAGTTAGTTGTGTATGACAATGAACGGAGGCAGATTGGATGGGCTAATTCAGAATGCAGCAAGCCACAATCACAGAAGGGCTTTCCCTTCCTCTGAGGAGCATTGCACAATAAACATTGGTTAATATCATTGGAGCTGCTCACCGGATAGCAGCCGCCATCATGTGCCGTATCCCATCCACAGTCACTGACCATAAATCCCGCTGTTTGTT

>Isogroup_342 GAGACGCCGACCACACCACGACTAGCGTGGTCGCGGCCGAGGTACTTGAACATCCATGGTTACACGATTCCAAAAAGAATCCCGACATTCAACTTGGTGATGCTGTCCGAGCGAGACTGCAGCAATTTTCTGCAATGAACAAGTTAAAGAAGAAAGCCCTGAGGGTCATTGCTGAACATTTATCTTTAGAGGAAGTAGCTGACATAAAGCAAATGTTCGATGTCATGGATATAAACAAAAATGGCAAATTAACCTTTGAAGAGTTCAAGGTCGGCCTCTGTAAACTTGGAAACAAAATGCATGATTCAGATCTTCAGATGTTGATGGATGCTGCTGACGTTGATAAAAACGGGACCCTAGATTATGGAGAATTTGTTACCGTGTCTGTCCATGTGAAGAAAATTGGCAATGATGAACATATCCAAGAGGCCTTCTCATACTTTGATCAGAATAAGAGTGGCTACATAGAGATTGAAGAGCTTAGAGAGGCTTTGACTGATGAGTTGGAGGGGCCTGCTGATGAAGACATTATCAATGGCATCATTCATGACGTAGATACAGACAAGGACGGAAAGATAAGCTACGAAGAGTTCGCAGCAATGATGAAGGCTGGGACAGACTGGAGGAAGGCATCTCGACAGTACCTGCCGGGC

>Isogroup_343 CCTGCTGCATAGCCGCCTGCAAAAATATGGCTGAATCCACACAGGAATCTGTCCTCTGGTAGAGGAGCAAGAACCTGGGTTCGTTCTGCAACTTTCCGGTCAACATCATATACCGACACTGGTCCACTGGGATCATAAGTTGTATGCAGCTCCATATCTACACTTGCAAAACGTATCTGACGGAGACTGAAGGAGCCAGCACGGAAATTCTTTGCAGCCACAAGCTTAGCATAAATTTCCTCCGGAAGAACTTCACCAGTTTCATAATGCTTTGCAATGCTCAACAGAGTCTCCTTGTGATAGCACCAGTTTTCCATGAACTGAGAGGGTAACTCTACAGCATCCCACTCTATCCCACTAATACCAGCAACAAAGGCTTCATCTTGTGTGGTAAGCATATGCTGCAGCGCGTGACCAAATTCATGGAACACGGTTTCAACCTCACGGAAGGTCATAAGACTGGGCTTATCACCAACTGGTGGCATCTGATTACACACCATATGGGCAACAGGCAGCCTTACAGATGATCCATTGCGAGCTAGCACGCGACTACGAGAAAAAACCACATTCATCCAAGCCCCGCCACGTTTTTCAGATGGTCTTGAATATGGATCGAAGTAAAAGTAAGCAACAGGGCTATTGGAAGAATCTT

>Isogroup_344 TTTTCCCAGCATTTGTATATCCTACCAGAGAAACAACAGGAATAGGCACCGATTGTCGACGGTTGCGATAAAGCTTTCGATGTTTTCGGACAGACTCCAATTCTTTCTTCAAAGTGCTGATTTGTGTTCTCAAGATGCGCTTGTCAACTTCAATTTGTTTCTCACCCATACCCTTGACTTGGCCTCCTGACTGCCGCTCAAGGTGAGTCCACATCTTTGTCAACCTAGGAAGTTGATATTCCATCTGTGCCAAAGTGACCTGCAGAGAAGCTTCATGTGTTGCTGCCCTTTGATTAAAAAATGTCTAGAATAAGAGCAGTCCGGTCACAGACTCGAACACCTCCACCAAACGCTTCTCTAAAATTACGTAGCTGTCCAGCAGACAACTCATCATCGAAAAATTACAGTCTCAATATCGAGGCCCTGAATAGCACTCCTGATTTCAGCAACCTTCCCCGAACCAATATAAGTCCTTGGATTTGGGGTTGAAAGCTTTGATAGGTCGATCCAGCAACCGTGAGTCCAGCAGTATCAGCTAACTGCTCGAGCTCCTTAAGCGATTCCTCTATGCTGAAGTTATCGCCTCCGTCCCTCTTGCATTCAACTCCAACTAGGTAGGTCTTCTCTTGCAATATCTCTTTGCCATTAAT

>Isogroup_345 CTTTCAGAAGTGCAACCGTTAGATGTGCTTTACCGGATCCAGATATACCTCAAAGTTGTGCCAATTCATCAGAGATATCACCGCCTGGAAGCAAACAAGAATCTGCTTCTGATGACAGGGAGTCTGCTCTTTCTGCTTTACTCCAAGAAAAATCATGGGGAAGATTGGGTCCCCAATGGATACGGCCGACACCCCCAAGACTTCATATACTTGATGGGGAGCTACAGTGGCTGAACCCTGACAACAATCATGAGCTGTTGTGGGATTATAGCATGTGTGCTGACACAAGTCGTGGGGCCGCAATCCGAGATTTGATTGCAAGAGCCTTAAAGGGTCCACTTGGACCTGGTCAACAGGAGCAAGTTGTTATAGAATTGGCAAAGGACTCTAAGCTAGTGTATCATTGCGGGATGACGCCACAGAAACTTCCAGACCTTGTTGAGCATAATCCACTCATTGCTGTCGAGCTTCTCTCGAAGCTTATGAACTCTCCTGACATTGCAGGTTATTTCGATGTTCTTGTGCACATGGAAATGAGCCTGCATTCTATGGAAGTTGTAAACAGACTTACTACTGCGGTTGAACTCCCGACAGGATTTGTGCATGAATACATCTCAAATTGTATTCAATCATGTCAAAACATTAAGGATAA

>Isogroup_**346** GAGAGACGCCGACCACACCACGACTAGCGTGGTCGCGGCCGAGGTACTCCTCTGACCGACCGAAGCGCTTGTCAGCAGATGACTCAAGAAGTGGGATCCCGCTAACGAAAAGAAGCAACAATGTCAGGAAGATGGGTCCCAGGATTACAAGCCATTCAGCTCCTGAGAGAACTGGGGCTGATGCTACAAACACACCCCACCAAAGTAACATTTCACCAAAGTAATTTGGGTGGCGAGTATAACTCCAAAGACCCACGTTACACCACTTTCCCTTATTGCTTGGAGAATTCTTGAACTTAAGCTTCTGTTGATCTGCTATAGCTTCCACAGATAACCCGATGACCCACATTATCCAACCAATGATATCCCGAGCTTCAATCGAAGGGTTTCTGTCACTGGAATTCACAATTGTAACAGGCAAGCTGACAGTCCAAACCCAGACAGCCTGGAAAATCCAGAAGACTGCTAACTTTCCAAAGTTGCTGCGCATCTCATCAAACCGTTTATCCTCTCCCCAATTCAAAATCCTCATTAGTAAAAACACTGCTAAGCGAAGTCCCCAAACCACAACAAGCACTGTCAACACAACCTGACGGAAGTGCCATGTTCCCTTCAAAGCTGCTGTTAGGACGGCGATTATGACAAAATTTG

>Isogroup_347 AGGCAGTGTCACAATTGTCGGTGCTGTTTCTCCTCCTGGTGGAGATTTTTCAGATCCTGTCACCTCCGCAACCCTCAGTATTGTTCAGGTCTTCTGGGGATTGGATAAGAAGCTTGCTCAAAGAAAGCATTTCCCTTCTGTTAATTGGCTCATTTCTTACTCGAAATATGCCACGGCTTTGGAAGGCTTCTATGATAAATTCGATTCTAGCTTTATTGATATGAGAACAAAAGCACGTGAAGTGTTGCAGAGAGAGGATGATCTCAATGAAATTGTCCAGCTTGTTGGTAAAGATGCGCTGGGAGAAGGTGACAAGATTACGTTAGAGACAGCCAAGCTTCTGAGGGAAGATTATTTGGCGCAGAACGCATTTACCCCGTATGACAAGTATTGTCCATTCTACAAATCTGTTTGGATGATGCGCAACATTATTCATTTCAATACATTAGCGAATCAGGCTGTGGAGCGAGCAGCTAATGCTGAAGGACATAAGATAACCTACGCCGTTGTAAAGAGTCGCATGGGCGATCTATTTTACCGCCTTGTGTCTCAAAAGTTCGAAGATCCTGCGGAAGGTGAAGACGTCTTGGTCGCGAAATTCCAGAAACTGTACCCTGGCCCGCGACCACGCTAGTCGTGGTGTGGTCGG

>Isogroup_348 GAGACGCCGACCACACCACGACTAGCGTGGTCGCGGCCGAGGTACTCCCGATTACATTGCTCCAGAGGTTCTATTAAAGAAAGGATATGGAATGGAGTGTGACTGGTGGTCCCTTGGTGCTATCATGTATGAGATGCTAGTTGGTTATCCTCCATTTTATTCTGAGGATCCAATGTCAACCTGCAGAAAGATTGTGAACTGGAGAAGTCACCTGAAATTTCCTGAAGAGGCAAAGCTTTCTCCTGAAACTAAGGATCTCATCAGCAAACTTTTGTGTAATGTTGAGCAGAGACTTGGAACAAAAGGAGCCCATGAAATAAAAGCACATACATGGTTTAGAGGTGTCCAATGGGAAAAATTGTATCAGATGAAAGCTGCTTTCATACCAGAAGTTAATGGCGAGTTGGATACTCAGAACTTTGAGAAATTTGAAGAGACTGGAGCACAAGTTCAGAGTTCATCTAAGTCGGGTCCATGGAGAAAGATGCTTCCATCCAAGGATGCGAATTTTGTTGGATACACATACAAGAACTTCGAAATTGTGAATGATGATGAAGTTGCAGGGATTGCTGAGTTAAAGAAAAAGAGTTCCAAATCAAAACGGCCAACAATCAAGACATTGTTTGAGAGCATGGATGAAGATGAGCCTG

>Isogroup_349 TGAAGAGTGCTATCGGTGAGGGTATGACCCGTCGAGACCATTCTGATGTGTCTAACCAGCTCTATGCCAACTATGCTATTGGAAAGGATGTCCAGGCCATGAAAGCAGTTGTCGGAGAGGAGGCGCTCTCTTCTGAAGATCTGCTTTATCTAGAATTCCTCGACAAGTTTGAGAGGAAATTTGTGGCACAAGGGGCATACGATACTCGGAACATCTTCCAGTCACTTGATCTAGCATGGACATTGCTTCGCATATTCCCCCGCGAGCTTCTCCACCGTATCCCTGCCAAGACCTTGGATCAGTTCTACAGCAGAGACGCTACCCACTGATCTGTCTTTGTTAAGAAATTCCAGTGTTTCCTCAAAGTGCAGCTTGAATAAATTGAACAGTGAACTTCTGGCCTTTTTGCTGTAGTTATATATAGTCAGATCCCATTCTTTTGAGTCAGTGAGCATACTTTATTTGTAGATTTCTCGTTCACGGCAACCTCTGTAATATTATCTCCTGTATTGTTGAACCAAATACACTTGAGCTCTTTTTGGTTCCACATCCCAGAACTGTTAGCAGAAGCATTCTAGGCCAAGATGCTCGAGCACGCTGGCTGTATCTACTTTTGTGTATGAATAAAGATGGGGCAATAAACTGTTGTG

>Isogroup_350 AGAAGGTGTCCTTGCTCAACTTCCCATAGTTTGATAGATGAATCAAACGATGCGCTCGCCAAAAGCAGCTGCTGATTAGGATTATTTGTTCCTGGACCTGTTGGGCTCCATTTAATAGTGTATATTTCCTTAGTATGCTCTTTAAAAATCATATACACATTTGTCCTGCTTCAAACTCCATATCTTTGCAGTCCAATCATCAGAACAAGACGCCAACAAAGAACCACTTGGATCCCACTTGATACAATTAACTTCGCTCTCATGGCCGCTGAATGCTTTAACTGGGCGTGGATCCCCAATCTTGCAAACATAGATCATCTTGTCATTTGAGCATGTTGCAAAAGAGTTGTTGTTTCGCCAATCGACATCTAGTGTTGGAGCTGAATGAAATTCAAATTGCTGCTTACACTCCCATGTCTTTGTATCCCACACAACAGCAGTTTTGTCTACACTGCCACTTACAATAAAATCACCTTTCCTATTCCATTTCAAAGAAAATATAGGTCCCTTGTGTTTGAACAAAGTCTGCTTCAACTCCCCATCTCTACTCCATATTCTCGCCTGGCAATCATAGGAGCCCGTGGCTAATAGTGATCCTTCCCCATTCCAGTCAAGTGTGGTGACATCCTTACTCTTCTCGACCTGCCCGGG

>Isogroup_351 ATTCTGGTCATTCTATCCCTGTTGGTGGTCTTGCTTACTATGTGACTGCACCATCCAGTTTGGCTGATATTCTGGCGAATCCATTCCATGCATTGTTCTATGTGGTCTTTATGCTGTCAGCATGTGCTCTCTTACTCTAAAAACATGGATTGAAGTTTCNGGTTCATCGGCCAAGGATGTTGCTAAGCAGCTTAAGGACAACAATGGTGATGCCAGGCCACCGTGAGTCAAACTTGCAGAAGGAATTGAACAGATACATTCCATGCATTGTTCTATGTGGTCTTTATGCTGTCAGCATGTGCTCTCTTCTCAAAAACATGGATTGAAGTTTCTGGTTCATCGGCCAAGGATGTTGCTAAGCAGCTAAAGGAACAACAAATGGTGATGCCAGGCCACCGTGAGTCAAACTTGCAGAAGGAATTGAACAGATACATCCCTACTGCTGCTGCATTTGGTGGAGTATGCATTGGCGCATTGACTGTTCTGGCTGATTTCATGGGCGCAATCGGTTCAGGAACCGGTATTCTGCTGGCTGTTACCATCATCTACCAATACTTCGAGACATTTGAGAAAGAAAGAGCAACGGAGCTTGGCTTCTTCGGTTTTTGATAGCGTCAGGAGCATGTTATGGCTACAGCTCTGTCTTCCCA

>Isogroup_352 GCCCGGCAGGTACTCAACAACTGATCAATGAAAAGCTAAGCAAAGCTGAAGCCCTAGGTGAACAAGGGATGGTAGAAGAAGCACAAAAAGCTTTAGAAGAAGTAGAAGCTCTAAAAAGGTTGTCGGCATCACGGCAGGAACCGGCTTCTGATCCTTCTAAATACACTGTGGCTGATGTTCGAATTACTGATCAAAAGCTGCGCCTCTGTGATATATGTGGAGCATTTCTGAGTGTCTATGACAATGATCGACGTCTTGCTGACCATTTTGGAGGGAAGCTACACTTGGGCTACATGCTGATTCGTGAGAAACTGAAAGAACTCCAGGAGGAGAGGAGCAAAAGAAGGACTGATAAGTCTGAAGATGACAGACGATCAAGAGAACACAGCAAGGACCGCAATGGGCGGGCATCCAGGGATAGAGATGCAGAAAGAAAGGACAGAGTTGAACCTCGAGAAAGCAGAAGAGACCAGGATAGAGATCGTGATAGACGCCATGATAGGGACCGTCGCCATGACCGTGACAGGGATAGAGACTATGACCGCTCCCGTGGCCATGATTCGAGGAGAAGGGAACGTTCTCGGTCCAGGGAGCGCAGCAGGCGCCCTGATAGATACTGAATCATGGCTGGCTGATTCCGGTGTACCT

>Isogroup_353 CATAGTAAAGGTGCTCATCCAAAAGTGATAAACCATCCATGGCATGAACCAGAACTTGAACCATCCAGCTATCCCTGTTTGGAGAATGATTAGTGGCCATCCAATTGCCATGAATGCAAACACACAAGCCAAACTTATCTTCACCCTAGGGAGTTCATTTGGTCTGAATTTTTTTAGATCGAAGTGCCACATCAACCAGTGAGCAATAGACATCCATGGTCTGATAGGACCGTAACCAAATATAATTGCCTTCCTTAGGAAAGAAGACGATTCGATTTCCTTTTGCCAAACAGGTTGCCATGCAGTATCTTCTACTAACATATTTGTCTTGGCGTGATGTCTGTCATGCTTAAATCTCCAAGGTTCATAAGGGTAAATTAATGGCAAGAAAGCTAGAGTTCCAACGATATCCTCCACTAGCTTATTCCTCGAAAATGATTTGTGCGCACAATCATGACCTATAACAAAAAACCCAGATAATGTTACTCTTAAAGATGTCATGGATACTCTGCCCAAAGAGGTATTTGAGATCGACAACGTAAAAGCCTGGGGATCTGTTTTAATATCAGTTACTTCATATGCTTTTGGTATTTTCTTGATTTCAAAAGCTCCATGGTATCTACTTCCCCTGGCTTGGGCATGGGCTGG

>Isogroup_354 AATCTGGCGACCAATTTGGAAAATGGAAAGGATTGCGAGAAAGTGCTGACCGTGAGATAGTATGGCCACCAATGGTGATTGTGATGAACACCTTACTGGAAAAAGATGATGATGATAAGTGGTTAGGCATGGGCAACCAGGAGCTTCTTGAATATTTCAGTGATTATGCTGCGACCAAAGCACGCCATGCTTACGGTCCAGGTGGGCACCGTGGCATGAGTGTGCTAATATTTGAAAGCACCGCTGTGGGCTTTATGGAGGCCGAACGTCTGCATAAGCACTTTGTTGATCAAAGAACAGACAGGGACACATGGCAAAATCGCAGGGTCCCTTTCTTACCTGGTGGGAAAAGGCAGCTGTATGGTTTCTTAGCCAGAAAGGATGACATGGAGACATTCAATAGACACTGCCAAGGGAAAAGCCGCCTGAAATATGAGATGAGGTCTCATAATGAGATGGTAGTGGCCCAGATGAAACAAATGAGTGAAGACAATCAGCAGCTGAACTATCTGAAGAACAAGGTGGTTAAGACAGAGCAACGCTCTAAAGTTGTAAAAGAAACCCTTGAAGTTGTTACCCAGAAACTTCGGGAGACGATGGAAGAAAATATCTTTGTAAGGCGTAAAGCTAAAGAGAAGCACTCTGAG

>Isogroup_355 CGAAAGGCACCTTTGTCATATCCAACCAATCCGGAGGACATACAGAGTATTGAATCGCTCATTCTCGATCTGTCAACTCTTAGAGCCGCGACAGATAACTTTGATGAAAGCAATAAACTCGGTGAAGGAGGCTTTGGTATTGTTTATAAGGGAATCCTTCCTGGCGATGAAGAAATAGCAGTTAAAAGGCTCTCACTAAGTTCTCGGCAAGGGATAGAGGAGCTGAAAAATGAGCTCGTTTTGGTTGCTAAGCTTCAACACAAGAATTTAGTGAGACTTGTCGGTGTTTGCTTGGAAGAACATGAAAAATTACTTGTGTATGAATACATGCCCAACAAAAGCCTTGACACCATTCTTTTTGATACTTATAGGAGCAGTCAGCTAGACTGGGGGAAGAGATTTAGGATAGTTAACGGGATTGCTCGAGGCTTACAATATCTCCATGAAGATTCGCAGCTCAAGATAATTCACCGGGACCTCAAAGCAAGCAATGTCCTGTTAGATTCAGAATTTATTCCTAAGATTTCAGATTTTGGCTTGGCAAGGCTCTTTGGCAGTGATCAATCACAAGATGTCACCAACCGTGTCGTCGGAACCTATGGATACATGGCCCCTGAGTACCTCGGCCGCGACCACGCTAGTC

>Isogroup_356 |METAL TRANSPORTER NRAMP2

AAGTTGTATACTGGGGAGCAGCTTTCTTCCAATATTATCCAGTTCAGTTCATCTTCATGAATGAACAGTCTCGAAAGTTCTCATGAGCTCTTCGAGAATGATGAGCGAAGGCGAGAATACAGCGTAGTGTTTCTCACTATAAGATAGACGACGAATGTGAGGTAAACAACCAGCACAACCGACAAGCTTGAGCGAACAAATGCGCCCCGGACTTCATCGGTATAGAAGGAGAATACAAGGTATCCGTTGATGAGCATCAAGAAAATCGTGACGATCCAACTAATCACTTTGGTGATAGGACCAATCACGAATGACCCCATGAGCTGCTCACTCGAGACAAGGGTGATGAGGGGAATTAGTGCAAATGGTATCTGTATGGACTGAAGAACATTGAGCGACTCGTTCAGAATGTCCATGGTAGGATCTTCAGTATCAAAAAATAGGGCCACGATCATAGTTGGAATAATGGCAAAGCTTCGAGTAATGATTGCTCGTAACCACTTCTTCAATCGAAGATTAAGGAAACCTCCCATGACGAATTGGCCTGCATATGTGCCAGTGATTGTGCTACTCTGTCCAGATGCTAACAACCCAATAGCCCAAATATAGAGGACAGGAAACAGCGCGGATCCGTATTTCTCCTGTAA

>Isogroup_357 AATATGGTGGGATAAGCTACACAAACGGCCCAACATATCTGTTTGATACCATCCAGTTTATGGGCGGTCGCTACAAAAATTAGCAGTGACGCACCTCAGAAAGGATCAGGTGAGAGATCTGCCAGCCCATTCACGGTTTCTCTTTTTTAACTTCGCTTGCATCTTATTGAACTTCTGTTTTCCTTTCTCCAGAAGAGGATCAACCACTACATAATCATTTGGATCATCCTTCCTAGTCATACCATCCTTTGAAGATTCAGGTGCGAAAGTGAGACGATTGGCGTGCCACTCTTTGTAGCCCTCACCATCTTCTGCCTTTGTTTCCATATTAGCAGGCTTATTACTAAGAAAGGAAGCCTTGAACTTCTGTAACTTTGCTAAAGTCTCCTCTTCACGGCCTTGGAGAGTGCGTTTTCTCTGTTTCTTCAAATGTTTCTCTTGTTCAGCTGGATTTAGCAGTTGCAAATTAACATCTCCTCTGAACATCCTTTCAGTATTGGCTTCAGAGCCAATACCTTTTTTCTTCAAGGATAGTTTCCATGATTTAGATAACTGGTGATCATCATCTTCATTATCATCATCATCACTCCTGTGAGCTGGTAATTCACCTCGGCCGCGACCACGCTAGTCGTGGTGTGGTCGGCG

>Isogroup_358 AGAGACGCCGACCACACCACGACTAGCGTGGTCGCGGCCGAGGTGAGAGAACGAATACTAAAAGCAGGGGGTTACATCCAAATGGGACGAGTAAATGGAACTATAAACTTGTCAAGAGCAATTGGAGATATGGAATTCAAACAGAATAAATTCTTGACTCCTGATAAGCAAATGTTGACAGCAAACCCTGACGTAAACACTGTGGAGCTTTGTGATGATGATGACTTTCTTGTTTTAGCATGCAATGGCATTTGGGACTGCATGTCTAGCCAACAGCTGGTTGATTTCGTCCATGAGCATATAAACACAGAGAGCAGCCTTTCTGCGGTGTGTGAAAAGGTGCTCGATAGATGCCTGGCTCCATCAACATTAGGTGGAGAAGGATGTGACAACATGACAATGATCCTAGTGCAGTTCAAGAAGCCAATTTGCCATGGCAACAATGCCAGTGCTGGTGAACAGTCAGCTGTTGAGGACAAGAACGCCAATGCTGGTGAGCAATCAACTGTTGAGGACAGGAACGCCAGTGCTAGAGGACAATCGCCTGGTGACATTGAAGGGTTGTGATTCAACTCCACGGTTGTTGTTATTCTTCTTCTTTGCCATCGATCTTCCTGTTCCACACAAACATGTCTGCACTAACCT

>Isogroup_359 AAACCAAAACCAGGAAGGCATTCAGTAGATGTAGTAAGAGGGGATGAGAACAGAAGAGGCAAGGAAAGGGATTATCACAAACCTGCCACTTCAGAGAATGGACATAGGCGCAAAGAAAGCGAGTATAAGAAAGGTCTAAGGCCTGTTCTTTGGTTGTCCCCTAACTTTCCTCTTCGGACTGAGGAGCTCTTGCCACTACTTGATATTCTTGCAAACAAGGTGAAGGCAATCCGTCGTTTAAGGGATCTACTCACAACAAAACTGCCTCCAGGAACTTTTCCAGTCAAGCTCGGGAGGCATTATCACTATTTGGAATGGAGCTCTCTTCCATGGAGAAGTTATGGAACTCACAAATTTTGTTGTCCACATCAAGTTTTCTTCTCTGGTCTCTGGACAAAATTGGCACTTGCTTAATATCTACGGTCCTTGTGCCGGGCAAGCGCGGGAAACTTTTGTTGAATGGATGATGAACTTGGAGAACCCAGACGACGAACTTTGGCTCTTAGCTGGCAATTTTAATTTCTACAGATCTGAAAAGAACAGGAACAGGAACAGGAACAGGGAAGGTGGCAACGTGCAGGATATATGCACCTTCAATAAAATTATAAACTCACATGGATTGGTGGAAATTCCCATCTCTGGCC

>Isogroup_**360** GAGGCATCTTGGCCGTCTCCATCTCCTTTCTCATCGCTGAACCATGACCTTGCTTCTTTCCTCAAAGATTTCACCGCCAGCCTGATGGCATCGGCGTCACTGCTCTTGGTGAAATTCTTTGCCATCTTAAGAATGCATCCACTTATGATCTCAGCCTCAGTTTTTATCCCGTAGTGCTCCATCAGATTACACAACTTAAAGTCATACTCTTCCTTAAAGCTCGCGGCTTCTGCAATGTAGTCTTTGTAGCCATCAACAATCAAATCGGTGTCATAAGACCGCCTTGCCACATCCTTCGTGAAGTGCCCTACGTGAGGGTTCTGCTTCTTTATTTCTCTATACAGCTTCCCTATAACACCCTTAGAGTCATAGGTAGCTCTGTCGAGCTTCTCCATGAAATCTGGATACTCCCTCACATAGAGTTCGGATGGAATTTGAGCCGGCACTCCTGTCTTTGGGTAATCAACAGCAATGGAGAAGAGCTCGGCCAGCTTAATGCACGGCGCACTTTCGGCCTTGCGAGATTCCCTGTCTGCAAAGACTACGTGTGCATTCGCAATGATTCCCAGACTCTCATTGACTATGTAATTTGTGAAATACTCGTGTACCTCGGCCGCGACCACGCTAGTCGTGGTGTGGTCGGCG

>Isogroup_**361** CTGTATCTGGCTGCGCAGACAATGTAAACCAGCAAATTAACAAAGCTGATCCCGGCAATCAACCAAAAAAACCGGTCAAGATGGCCTTCATTCAGGTTATCCGGGATCCATCCAGGATCTCCCCCTTGGGTTGTAACATATGACACCAAGGTCAATATGATTGAGCTTAAATAGCTCCCCGCTGAGACCGTAACGAGCGCAAATGCAGCACATAAACTCCTCATGGCATCTGGGGCCTGATCGTAGAAGAACTCAAGTTGACCAATATTAGTGAAAACCTCAGCAGCGCCAACAAAGAAATATTGTGGTATCTGCCAAAGAATGCTCATTGGAACTGCAACCTTTTCATGGGTAAGACCTTCAGACATGGCAATCTCTAGGCGCTTCATCTCAACAAGAGCTGCAGATATCATCGCGATAATGGACAGGACTAGTCCAATGCCAATCCGTTGTAGCTCAGAGAAACCCTTTCCCCTTCCAGTGAACTTTCTAGCTACAGGTATGACAACACGGTCATATAGTGGAACCCATATAATGACACTGATTACATCAAAAGTCGAGAGGGACGCAGGAGGAACATTGAAAGACCCAACCCTCTTGTCAAGAACCATTCCCTGCTCTATGAACATGGAAGAGTTCTGAGC

>Isogroup_362 |EUKARYOTIC TRANSLATION INITIATION FACTOR 3 SUBUNIT F-LIKE

CAAAAAATGCATATTCTGCCAACATATTCTGATTTCTTCTCATTCAAGCAAACGAAAAACAAGCATAGACAGCATGGAAATCACAGGACTTGGGCAGCGGTGTTCAGCTTCTCGGCTACGGCGATCTGAGTCCGTGTGATGCTTGACAGGTATACTAGCGCAAGGTTATCCTGAATCCTGTCATTGAAGAGTCTGTCAAACGATGATGGAGACAGCTTTGGCATGGAGGAAACAGCATCAGCAATGAACCTGCCTATCTTGTTGTCCCCTGCCACACGGCCTTCCACAACATCATCAGAATATTTGTAGATCTCATCAATAAGAAGGTATAGCTTTTGCATCGAGGACTCCATTCCTTCCAAGTCGTTGGGCAGCTTATCCACCATCGTAGACTTCAACAACTCAAATCCAACTTTTTCTGCATCAAGCATCTTCAGATCCAGAGGAATTTCCTGAAATTGTGCGGCAAGGTGCCTATCTCCTAGAGACAGGTTGGATGAGACATAGGCTTTGATGGAAGCCTCGCCCATAGTGAAGCCAGTATCGACAGTAAGATGAATGGGGCTTTGCACTTCTCTTGAATAAAATTCATGGATAAGCGTACCTCGGCCGCGACCACGCTAGTCGTGGTGTGGTCGGCGTCTC

>Isogroup_363 GCCGACCACACCACGACTAGCGTGGTCGCGGCCGAGGTACCTCGAGTTCATAAGCTTGAGTGGTTTCTCGATCATCTTGGTGATCAGACCCTTCCTCCTGATATTCATCGTTCTCTTCTCTTTTATCCCTTTCAAGTATAACAAGCATTTTCCCACCTTCTTCTGGTATATAGCCAAAGGATTCACAAACAAAACCAGACACGGTCTCATACTGATGACCCTCTGGTATTTTAACACTAAGCTCTTCAGATAGATCGTCTATTGATGTATTTGCATCAACATCAAATGTTCCATCTTCCAGCATAACTATATTACCTGTTTTCTTCTGGATTTCCTCCTTTGAATCATTCTCGTCAAAAATTTCGCCGACAATTTCTTCCACCACATCTTCTAATGTCACAATACCAATAGTTCCACCATATTCATTGAGGACCACTGCCATATGAACCTGCCTGATCCTGAACTCTCTTAGCAGGTTCCAAACAGACATCGAATCTGGGACAAAGTATGTCGGCATATGTGCGATTTCCTTCACAGTGATTTCCTTCAACTTCTCAACCTCTTCAACATATTCAAGCATGTCCATGGCATAAACAATTCCGACGATATTATCTATGCGCTCCTCAAATACAGGAACTCTAGAC

>Isogroup_364 CCGACCACACCACGACTAGCGTGGTCGCGGCCGAGGTACATACGGACTTTCTAGATAATCATCATATGATTTTCAGATAATTATCGTGTGATATTCATAGACTTGTTGAGCCCACAACTTAACCAACTGATCCCTCAAGCTTCAGGTTCATGAGTGCATCTACCTCCTGGGCAAACTCGTAAGGAAGGTGCTCAAAAACAGCTCTGCAGAATCCACCGATCATTGCTGCAACAGCCTTTTCATGGTCTATACCTCTTTGCTGGAAATAAAATAGCTGGTCCTCTCCAATTTTAGAAGTGCTTGCCTCATGCTCAACACGGCTACTAGTGCAACCAACCTGAATAGTGGGATAGGTATTGGCAGCAGCGTTATCCCCAATTAGCAATGAATCACACTGAGAAGAATTATAAGCATTTTCTGCACCTGACATAATCTGGACCAGCCCGCGGTAGCAATTCCTTGACTTCCCTGCCGAAATACCCTTGGATATAATCCGGCTGCGTGAATTCTTCCCCTTGTGGATCATCTTCGTCCCTGTATCTGCCTGCTGGTAATCCTTAGTCAGTGCTACCGAGTAGAACTCTCCAACAGTGTCATCGCCATCAAGCAACACACTCGGGGTACTCGCCGGGCGGCCGCTA

>Isogroup_365 CAGTTCTTGTCACTGCTTACGTTTGCCATTATAATGTTCACCCAATTCATAACGAGCTCAAGGAGTCTAACCAGATCAAGCCAATAGTGCACACATCATTGGCTTTATGCTCAACTGTCTACATCACAACAAGTTTCTTTGGATATCTTCTGTTTGGTGAATCTACACTCTCTGATGTGCTCGCTAACTTTGATTCAAATCTCGGCATTCCATATAGTTCAGCTCTTAGCGATGCTGTCAGAGTGAGCTATGCCGTCCACCTTATGCTCGTGTTCCCCATGATATTCCACGCACTGCGGCTTAATTTGGATGGGCTTCTCTTTTCCTCTGCAAGGCCCCTAGCTTCTGATAACAGAAGGTTTGGCGTAATGACAGCAGTGCTCCTCCTCGTGATTTTCGTGTCTGCAAACTTCATTCCTAGCATCTGGGACGCCTTCCAATTTACTGGGGCTACTGCTGCTGTTTGTATTGCCTTCATTTTTCCAGCTGCGATCACTTTAAGGGATCCACATAGCATAGCGAAAAAGTGGGACAAGATTCTGGCCGTCTTCATGATTGTTCTTGCGGTCACTTCAAACGTAGTAGCTGTGTACCTCGGCCGCGACCACGCTAGTCGTGGTGTGGTCGGCGTCGTCT

>Isogroup_366 AGCGTGGTCGCGGCCGAGGTACATCCCAATAAAGGACGACCATACTTGTTCAACTTATCTCTTTCAACTTGGATACCATGAGGCGGACCTTGGAAAGTTTTTGTATAAGCAGGGGGAATTCGTAGATCCTCCAGACGTAGAGCACGTAGGGCTTTGAAACCAAATACGTTACCCACAATGGAAGTAAACATGTTAGTAACGGAACCCTCTTCAAATAGGTCTAATGGATAAGCTACATAACAGATCCATTGGTTGTCTTCCCCAGCAACAGGCTCGATGTGATAGCATCGTCCTTTGTAACGATCAAGACTGGTAAGTCCATCAGTCCAAACAGTTGTCCATGTACCTGCCCGGGCGGCCGCTCGCCTCAAACGTGCTACGTTCTGCACCTCAACTTCCCTTGTTCGAGACAGACAGATAATATCATATCTGAAAATTCTGAGAGCTCAAAGCGCAAGGAGTTCTGGCTCCTCTTCCACTGATGATTCGGTGGTGGAATGGGCCGAGCCGTTCTCGACTTGGTGCTCCCGTGTGTAGATTTTTGAGCCAGGCACGGGCAAACGGGAATCAGTCATGTGGACTAATCCAGGAGTGGCCGCTCCGTTTGAGGAATCTATGGCTGCTTGCAGCTTTTTT

>Isogroup_367 GGGGAGGACTCAATGAAGGATCCTGCTACTGACCCGAATGTTCCATTAGCAAAGGTTTTAGTTGATCAGGTGGATAGGAAGCTGGTCAAGCAGACAGTGATGACGTCAGTTTATGGTGTCACATTTATTGGTGCTCGCCAGCAGATTATGAAGAGACTTCAAGAGAAAGGGCACATTACAGATGAAAAATTGCTCTATAATGTGTCGTGCTATGCTACCAGGGTAACTTTGGATGCGCTAGGACAAATGTTCCAATCTGCCCGCGGTATAATGGCATGGCTTGGTGACTGTGCAAAGATGATTGCTTCAAAAAATCAACCAGTCAAATGGACAAGTGCTGTTGGTCTTCCAGTTGTTCAGCCCTACAAGAAATATAAAAACTATATGATACGAACTTCCTTGCAATGTCTGGCGTTACGAAAGGAAGGGGATGCAATCGCAGTCCAACGGCAGAAAGCAGCTTTTCCACCAAATTTTGTGCACTCTCTTGATAGTTCACATATGATGATGACGGCGATTACTTGCAAAGAGGCTGGTCTTCATTTTGCAGGGGTGCATGACTCATTTTGGGTGCATGCATGTGATGTTGATAAAATGAATCAGATCCTGAGAGAGCAATTCGTGGAGTTGTACCT

>Isogroup_368 TACTGACTCAAGTATGGACACAAATTACACAACAGCTTCAGATATACTTCTCCATCCGCAGGAAAACTGGCACACCCGTCCCGATGCAGTTCAATGCCACTTACAAGTTGATGTCAACGAACGAAAACTTGGCTAGTATACTTTACAAATGAGCACAGGAGGATGCAGATTCTGTTGGTTACCATCGATCATCTCCTCATTCAGAACCCTTGGAGGAGATTGGTGCATCATTAGATCTTTTTGAAGAGAGAGGCAGAGATAAATCTTTTTCTACAGATCAGTAAACAAGCTTGGGCTCAAATTTGGCAAAATCAACAGAGCCAGACTCGATCGGGGGCATGAGGTACGCGGCGATGATCTCAGACACCAGTTGGGCAATAAGAGGAACCCTCTTCAGGTTCTTCACAAGGGGGATGTCGTTGGACTCTCCAATAGCAATGATCTTAAGGTTGATTTCTACCATCCTGTCAAGCTTCCTCTTGAATTCAGGGTTCTCAACATCTGGGACAGCAGGGAAGATTCTCGCTGTTGTGCGATTGGTCTCTATGATCACATGCATGTCGAATTCTTTCGTGTCAAGACCAATTCCTTCGTAGAAGGCACTACGTTGGCAGTCATTCAGGTACCTGCCGGG

>Isogroup_369 TGTGCCCCAGAGTCACATAGATATATTTTATCAGCATCAAGCTCAGCACATGTATTTGCCTCAGGTGAATAGTGAATTATTGCTGCATTTGGGCCCACAGACGAAATCGTAGGAAAACTCAAACCTTTGAAATGCACATATTAGTCACCAATGTCATCTCAACATAGTAACTGCATAGTTATGCTTTACTGATCTACCTCTTTCTCCGCACGGAAGCCTTCCAGTTTATCACTAACAGAAACCTCGGTAAGTTTTATTTTTTCCAAATGCTCCTTTTTCTGTGATCCATTTGCCTCACTGAAGTAACCAGAGGCCCTATAGTGCTCTTGCATCTGATTGTCCAGCCAAGCCAGGTATTGAACAACGGCCGCTCCATCTCGAATGTGTGCTTTCCTCAAACCATCCAGCTCCATGGGATTCTTAACAGCCTTTGGTAGAGCAATAGGTGATTGCAGCATCAAAACTTGATCCTGTCTGAGCTTTGAGTAGAGTGCAAGGCAACAAGAATTTGAATCAATCCAAATTTTCAAATGCTCCACCCCCTTGATATCCTTTTCCACGTGCAAGTTACCATTAACTATAGAACCTTCTAGCTGTCCAGACGAAAGCAAGCTCACATCCGGTTGAACCATATG

>Isogroup_**370** ACGCAAACACCCATCGCCCTCTTCCTCCTTCCCCAGGGAGGAAAAAATAACTTCTCCGTGGGAGAGAGAAAAGTCGCGTGCCGTCTCCTCGCCCACAGCCCGTCAGTAGCAATGGCACAAGAGAGCTGGAAGGAGTCTGAGGAGACTGTCCAAACACCTGAGGCACCAATATTGTGTGTAAACAACTGCGGATTCTTTGGCAGCAGCATGACAAACAACATGTGCTCGAAGTGCTACAGGGACTTCATTAAGGTCACCACGCTGGCTGCCCCTGTAGTGGAGAAGAAAGTGTTCACAGCGGCGTCATCTTCTACGGTGCCATTGGAGCCAGCAAAGCCAGACGAAGCGCCCGCAGCTACTGCGGTTGATAGCCAAGCAGCACAGGAACCTCCGAAGCCTCCTAGCAACCGATGCCTTTCATGCCGCAAGAAGGTTGGGTTGACCGGTTTCCAGTGCCGCTGCGGTGGAACCTTCTGCTCCATGCACCGCTATGCGGACTCCCATGAATGCTCCTTCGACTACAAGAAGGCTGGCCGAGAGCAGATAGCCAAGCAGAACCCTGTTGTGATAGCTGAGAAGATCAACAAGATCTGATTGCTGGTTACAAGGGTTCACTTGCTTCCTATTCGTGGTGG

>Isogroup_371 CTCCTCGAGAAAGGCTACATCACAGAAAGAACTCCGTTAGCTGGCTCATCAGCTGGCGCCATAATCTGTGCAGTGATTGCATCCGGGAACACAATGCAAGAGGCTCTGCAGGTGACCAAGATTCTAGCTGAGAACTGCCGGAGTAACGGGACTGCCTTTCGCCTTGAGTCTGTTCTCAAGGATGTTCTGGAAAAGTTTCTCCCAGATGATCTGCACATCAGGTGCAACGGAAGGATCCGTGTTGCTATTACTCGGTTGTCCTGGAGGCCTAGGGGCTTACTGGTTGACCAATTTGACTCCAAAGAAGATGTGATTAATGCAGTTATTACATCATCTTTTATTCCTGGATACTTAGCTCCCAGGCCTGCAACTTTCTTCCGTAACAGGCTGTGCATTGATGGGGGCCTTACATTGTTTATGCCACCCACTTCTGCTTCCGAAACAGTTCGCATCTGTGCTTTCCCAGCTAGCAGACTTGGGCTGCAAGGGATTGGTATCAGTCCAGACTGCAATCCAGAGAACAGACCTACTCCCCGACAGCTGTTCAACTGGGCTCTGGAACCTGCTGAAGATGAAGTTCTGGGCAAATTGTACCTCGGCCGCGACCACGCTAGTCGTGGTGTGGTCGGCGTC

>Isogroup_**372** GACGCCGACCACACCACGACTAGCGTGGTCGCGGCCGAGGTACAGTTTTTTTGGACCCTTCTCCTGCTTGCTTCTTCATCGCCGAGGGTGACACTCTGTATTCCTCCAACAAGCATATCTAGTGATGGATTCATGGTCAGGTTCTCTATTGTTACACCGTGAGCGGAGGCAACAAGTTGAATACCCCGCTGTGCAATGGTGCTAGCAGCCATAGCCTCTAGCTTAGTTCCAATTTCATCAATGACAATTGCCTGAGGCATATGGTTTTCCACAGCTTCTATCAACACCTTATGCTGCATCTCTTGGTTAGGCACTTGCAATCTACGGGCATTGCCTATTCCTGGGTGCGGAATATCACCGTCTCCTCCTATCTCATTAGATGTATCAACAATCATCACCCTCTTATCATAATCATCTGCTAGCATACGGGCAATATCTCTTATGACAGTTGTCTTCCCCACCCCAGGTGGGCCGATGAGCAGCAACGACCCACCATCCTTGACCAAATCCTGAAGCAAATTAGCACTTCCGGCCACAGCCCTCCCGACGCGGCAAGTCAATCCCACAATGTCTCCCTTCCTGTTCCTAATCCCGCTGATCCGGTGCAGCGTCCGGCTGATTCCGGCACGATT

>Isogroup_373 ACGGAGCTGTCGCTAGTTTGCATCTGCCCATTTACCTTTATGCCACCTTTCTCCATCAACAGCTGACCTTCAAAAAGGCTGGTATTTGCACGGATCCCAATACCAACAACTACCATATCAGCGGGAAGATGGTTACCATCTTTTAGGATTACCGAAGTCACCTTCCCTGTTGAGTCTTTTTCAAAGGATGTAAGCACAGTTCCCTTGGTAAAAGTAACTCCTTTTGAAGTGTAGTAGTTTTCATAATATTCTGCAATTTTTTCTGTAAATAGCCGACCCATGCAATGATTTTCAGGGAAGACCATGGTTACTTTTATCTTATTAGTAACCAATGCTGCTGCACATTCCATTCCTATGTAGCCACCACCAATGACAACAGCATTTCCACCAGAACATGAATTCATCGCATTCACTAACTTATCTGCATCTTCAAGATTGCGTAAATAACATATATTTGCAGCATCTGAACCATTAATTCCAAACTCTTCAAGCTTCAGAGCCCGAGCACCTGTTGCAATTATAAGCGTCTTATAGCTGATAGTTTCTCCGGTGGCTGTAAGCAATGTCTTCCGTCTCACATCAGCTGATATCACTCTTGTTCCAAGAACAAGTTCAATACCTTGTTCCTTG

>Isogroup_**374** GGCGAGTACACATTATCATCATCACCAGGGGTGAACGTATTAGCTCTTCTAATAAGCACGGATTCCGGGTCGACTGGTTGCTCTGAATTATTATCATCATGCCAGTTATGTGCAGTATCATCATTGGACTCATCGTCCTCATGCCACACTTCATTTACTTCTGGAAAATGATCATCGCTCTGCCACTCGTCATGTGCTTCTGCAGGATCATTCCACTCTCCATGCGATTCTTGAAGATGATTATTGCTATGCAGCTCTTCAAGCACTTCAGGAAGACGGTTATTGTCATGCTGCTCGTCATGTGCTTCAGGAAGATTACCATCACTAGCTGACTCATCACTTGTTTCCGGAAGAGATGCATCTTGATGATCTCCAACCTCCGTCCTCTCATCGGATGAACTATTGTCCGTATTGTCATGCCAATTTCTTCTGGATACCTCCCCAGTAGTAACAGTATGGGAAAATTGATCCCAAACTCTTCTTCTATCCACCTCCAAATCTTCCTGCCAACTCTCCTCCTGGCTAGGAGGTTCGGACAAGCCTTCAGCAATACTAGGAGTATTACTTCCATAATCCGAGGCTGTTGCTGTACCTCGGCCGCGACCACGCTAGTCGTGGTGTGGTCGGC

>Isogroup_375 GTGGTCGGCCGAGGTACTACCCTATATCTAGGACGAGATAATAAACCGACCAGTTTACTCTGATCTCTATACTTCAGCCTTTAGGTCATCTGGCATTGGTTTCTCTGTCTTCCGAAACGATCCATGCAGCTGCAGAAGCAAAGAATTGCTGACGACAAAGATTGAGCTTAGGGCCATCAGTCCTCCTGACAGAGAAGGTGTCATTGCAAAATCAAAGTGAGGTAGTAGCGCTCCAGCCGCAACAGGAATTGCTACTATGTTATACGCCACTGCCCAGGCCAAATTCTGGTGAACTTTTGCCATAGTTGCTTTAGACAGGGATAGGGCATCAACAACCTGAGAGAGTCTGTTCCCTAATAGAACAACTGAAGCTGCATCAGAGGCAGCGTTCTCTTTTGAGTGAGTTCGCATAGCTATTCCAACATTAGCAGCTGCCAATGATGGCGCATCGTTTATTCCATCGCCAACCATCGCAACTCTATGCCCTTCCTCTTGCAAAGTTGTTATAATGCCTGCCTTTTCCTGCGGAGTGAGAGATGACTTTCTGTTTTCACTCCTGATTCCAACAGTTTTCCCAATGCCTTCCACTGCTTCTTTCCTGTCTCCTGATAGTATGTATGTCGTAATTCC

>Isogroup_376 CACGCGAGAACATGGACACATCATTGTCAAACAATCTGGAAGTAGAACCATGGAGCCTGGATTTAGAAGCTTCTAGGAAAGTTTCAGAAGACAAGTTCTCCCTGAAGTTGGCACTGGAAAGGAAGTCTTCCTTGGTGTCGACCTTTGATGGCGAGAACAGTTTAGACTTCCTTTCATAAACATTAATATGGACGCCACTTGAGGGTGGTGTTCTAAACTTCCGACTGCCTAAAAAAGGGGACTGCCATGGTGGCAATGACTGCATGTATGACTTTGCAACATCAACTGGAGAACCCTTGTCATCTTCGACGGGAGAATTAAGCATGTCAGTGTTCAATGAGCAAGGTCCATGATTTTCAGGCTTTGTGCCTAGTCCCTGCCTTTTCTCTTCCAACCATTTCTTTGCTTCCATAACTGCCGCACTGCATAGCTCAGGCGACGCTTGGAGAGGAGAGCCAGGAGAAAAGTAACCAGGTCCAATACTAGAAAATGGACCTGAATCAGGGAAATCTCTATTGTGATTCAAGGAACGCCAAGCTCCTGTAAAATCATGGCCAGCATCTGCCTTCCTTCTTTGAATTTCATACCTCGGCCGCGACCACGCTGCCCCTATAGTGAGTCGTATTAG

>Isogroup_377 CACTGCCAAAAACATACTTATAGCCGTTGGTGGTCGGCCATCGATGCCAAATATCCCAGGAATAGAGCATGTTATAGATTCTGATGCTGCACTAGACCTGCCTTCGAAACCTGAAAAAATTGCAATTGTGGGAGGTGGATATATTGCTTTGGAGTTCGCTGGCATTTTCAATGGCTTAAAAAGTGACGTTCATGTGTTTATTCGGCAACCAAAAGTATTAAGAGGGTTTGATGAGGAGGTCAGAGATTTCATTGCTGAACAGATGTCTTTAAGGGGTATCACATTTCATACTGAACAAAGTCCCCAAGCTATAACCAAATCAAATGATGGTTTGTTATCTCTGAAGACAAACAAAGAAACTATTGGTGGGTTCTCACATGTAATGTTCGCAACAGGTCGTAAACCGAATACAAAGAACCTTGGACTGGAAGAGGTTGGGGTCAAAATGGACAACAAAGGGGCTATAGTGGTCGATGAGTATTCTAGAACCTCAGTGGATTCAATTTGGGCCGTTGGAGATGTTACTGATAGGATCAATCTGACACCGGTTGCACTGATGGAAGGTGGGGCATTTGCGAAAACTGCATTCGGTGATGAACCTACCAAACCAGATCACAGAGCTGT

>Isogroup_378 TGCTGCCCTGAAGAGCCAAGATAATAGCTTGGAGACCCGTAGCTGGAAAGATGGATATGGGGTGAACCTAAGCACTGACAAGAAGATCCGTAGCCAGACCGAGAAACATGTTGTGATGTATATCTGATGCTGATCGGCGGAGACAAATGCCCTCCACCAACTGAGTTTCCAGATGCAAGGTGCGAAGTGTGAGACCATGGGGATGACATGTTTGCAAGGTCTACACTGCTCCTACTTACACTGATGGTAGGTGAGTTGGAATAAGATCTGCATGGATTATGAGTGCGGCGCATACTGGAATTGGAGTGTGTGTGGGATATGGACTGTGATGAAGGATACCGACTTATTGACCTGGGGGGAGATGCTGTCACCATTGACCGGAAAGAAGACAAAGCCGTCGATTCAGCAGGCAATTGGAAAACAGAAGAAAGCAAAGGGGTAGACTCTGATGCAGGGAGGTTTGACGCTCCAGCATTTGCATCCCGCTTGCATACAGGGCAGAATGTTCTCCATGAGGTGAGCCACAGGTCCACACAACCAGCATGAAACTTGTGACGACAAGGCAGCACCCTTATCTTCTCGCCAACACTGTAGTCTTCTAAGCAAATGGCACAAGTTGACGAT

>Isogroup_379 AAAATGAATGATAAACTGCACATCGAACTATTCCGAATGGCTTAACTTTCACCTGTGGACTACAATTGCATTTTCAACAAAAGAAGGCTGTGGAAGTAGGTGTTTGTCTTCAAAATCATCATCATCTTCAAACGGTTCCTTAAGGGTTACATTGGAACCATCAGAGAAATCACAGTCTGTGAGGTTCTTTGTCTCAGTAGACTCCAAACGCATACTTTCTGGTAGAAGGCAGTTGCAAAACGCACCTATCTTAGCGAGTCGATTAACCCAACCAGGAATTGGCTTTCCTGTCAATCTGGTGCTAAGATCATCAGTGAAGTGGTTACAATTCTTGGAAATGAGGTGGTATGTATCTCCATGATATTCCGAGGCCATTCTCTGAATGAAATCTCGAAATTCCAAGGGTTGTAAACTTGTACGCCCTATGAAAATCGAAGATCTGTACAAGAAGCCTGGGCAGTTTTTCGGTTCCACCTCAAAAACACCACTTGTTGGATAATCATGAGCTCCAAAACTGTACCCATATGCCACCGCCGCGAAATTTGCAGAGAGCGCGCAGCAGATGATCGGATGCAACAGTTTCTTGATACCAGCGGGCAACCCAGAACCAACCATGTATCCCA

>Isogroup_380 TCCATTTATTTGCAACCAGTTTAACAATCACATTTTCTGAAAAGACAATGCAAGAAGACGTGGTATACTACAGTGAATTACGAACAGTCAGAACTCTATGTCTGTAATTCTGGGGGATGCTGCCCCTGAAATTGTGCGTTTATCATCTCTGCTCAGATATCTCAGTCATGCTTACAGATCCCGGCCGCGTCTATCGCACACTGGTGTTTTTGCTTCCGATTCTGGCTTCTTGATCTGTTTCTGCATCTGTGGCAAGTCATGCTCAAACACATCACTGAGTGCTTTCGATGCTTCTGCGAGCTCCTCGGAACTGATTGAAAGAGGAGGGGCTAATCGAATTATGGTGTCATGCGTAGGCTTTGCTAGAATGCCTCTCTCCTTAAGCTTAATGCAAATATCATATGCTGAAGCAGGGTATAGAGCTTTGTTGCTTAGGTCTACTGCATTAAGCAAACCTCTCCCACGTATTTCCCTGATAATATGGGGAAATTTCTGTTGAACCTTTTGTAACTGGTTTCTGAACTCCTCACCTAAATCCGCAGCTCTTTGAACAAGACCTTCATCTTTGATCACTTTCAGTGATGCAACTGCCACAGCACTTGCCAATGGGTTTCCACCAAAT

>Isogroup_381 CGCGGCCGAGGTACACACATCAAGCAGCATGATACCAGATGAAGCAACACTTGGTGTCAACAAAAACTGAGCTTTATCGCAACACCCTATCCAGAGTAAGAGAGCTATCTTATGTGAATTATCATCTTGTCGAAGAGCGCCTTGTGCTCCGGGAAATACTCGAGCAACTCGTCCTTGGTCACCCACACATGGTCCTCGCACTTGCCGATATCGAGTTTTGTCGTGCCGATCACTTGCGACTTGAAGAAGAAACGCTTGAATGGTGAAACACTAGAATCTTCTGTTTGTTCAGCTACCATATGGGCCATTGGAGCATTGCCGACAAAATATGTGTTGTCAAGTCCGCCCAGAACAGAGTTCAAAGCCGACTCAGCACACAGACGCAAAGTGTCTTCATTCTCATACACTTTTTCTGGAAAATGCCAAACAGACTTGCCATCAGGTGCCCCAAAACTCTTGCCATACAGAAGAAGGTAGAGTTTATTATCAAGAGCTCGTTGTAGTGACTTCCGGTCATTTGCTTTGTCAGCTTCTGTGATTCTTGGAGCGGGCACATAATCTATCTGATAATCACCCTTGCCCCTTGCCTCCGCCTTGCCGAGGACATCGTCGGGGTACCTG

>Isogroup_**382** CTTCTCAATCGTCAGGTTTTCGAACAGCCATTCCTCCATCCATGAAGCCTGAATCTGCTCTCCCAACTTCTCAATCATCAAACAGTCTGTTAGGCGGGATTGATCTGATTAATCAAGCTTCAACCAGTCAGCCTTTCATGACTAGGCACGGAGGAAATCTTCCTGGTCTCATGAACCGTAACTCAAATGTAATACCATCACAAGGAATGAGTAGCTTTCAAACTGGGAATAATATGTATCTGGTTAACCCGAATTCCATGGGAATGGGCTCTAAACCACCAGGTGTTCTGAAGACTGAGAGCACTGACTCTCTTATCCAAAGCTATGGTTATGGTAGCAACCATATGGATTCTGGCTTGCTGTCTTCTCAGTCAAAGAATTCACAGTTCGGTTTTCTGCACAGTCCAAATGATGTCACTGGTGGCTGGTCTTCTTTGCAAAATATGGATAATTATAGGAATACTGTTGGGCCAAGTCACCCAGTGTCCAGTTCGTCTAGCTTCCAGAGTTCCAATGCAGCCCTTGGAAAATTGCCTGATCAAGGGCGAGGAAAAAACCTTGGGTTTGTTGGGAAAGGTCCTGGCCGCGACCACGCAGTCGTGGTGTGGTCGGCGTCTCTC

>Isogroup_**383** TTAGAGATAGAGGAAGCGAAATTGTCAGAAATATTTAAATCTACAAAACATTTGGATTTGCCAACACTCATGGGCTTATTCGAGGGTGGTCCTGTGGAGCGTCGAGTGATGGAGAAAGTTGGTTGTATAGACTACTCTATTACAGCATGGGAACCAGTCAGGGAAGATGTTTATCAGAGGCAAGTCCATTACAGGTTTGACAAGAAATCCGCGCGCCAGGAGGGGAAATAATGAGCACCCAACAGAAGTGCCCGTTGCCTGATAAGAAAGGATGGCTTGTTGAAGAAGTGATGACACTTGAAGGCATCCCTATTGGTGAATATTTCAATCTCCATATTCGATACCAGTTGGAGCAGATTGCGTCGAAGCAGAAGTCATGCACTGTCCAGGTATTTATTGGCGTGTCATGGTTAAAGAGCTGCAAGAACCGAAAGAAGATCACGCAAGAGGTGTCATCCAAATTTTCCTCTCAACTCAAGAAGATATTCAGCCTACTAGAGAAGGAATTCGTGCCAGCTAAATAGGAGCTTAACTTTTTTGCATCCTACTTTTGTTGGTGCACTTATGGATCACCGGAAAGCGTCTTCTGGTGATACCTTGATTCTGAGTCCCGGTGAGCAGT

>Isogroup_384 GGACCAGGTTATCAAGAGAAATTTTATTTCAATCCAGGTGACACTGGTTTCAAGGCTTTCAAAACCAAGTATGCAACAATTGGCGTTGGAATTTGCTGGGATCAGTGGTTCCCAGAGACTGCAAGGGCTATGGTGCTTCAGGGGGCTGAATTATTGTTCTATCCCACTGCTATTGGATCTGAACCCCAGGATACTAACCTGGATTCCCGTGAACATTGGAAGCGTGTCATGCAAGGCCATGCCGGTGCTAACTTGGTTCCTCTAGTCGCTTCTAACCGGATAGGAAGGGAAACTGTTGAGACTGAGCATGGAAACAGCACTATAAAGTTTTACGGGAACTCATTCATTGCAGGGCCAACTGGAGAAATCGTCAAGCTTGCAAATGACAAAGACGAGGAGGTGCTCGTGGCGGAGTTTGACTTGGATGAGATCAAATCTATGAGGCACGGTTGGGGGATATTCAGGGACAGGCGTCCTGATCTATACAAAGTGCTATTGACATTGGACGGCGAGAAATCGTAGGCTTCCACTTTGAGGTTATCCGTCCATGAAAGGGACAAGAACCAGTTCCGCAATATTGGGAAAACACGGGTCCATGAATAGTTCACCAACCCAAATG

>Isogroup_385 CTCTGCTTCATGGGTCTCCGGGCACTGTAGACACTAATCCACTGAGTCGCTGGCATTCCCATTCTAATCTTCTTCTTAGGTTGCTCATCATCTTCCTCAACTATAAGGCGGCCTCGCTTCTGACCAACCTGATATATAAGCTTCTGAGCACCATCAGTGTTTATTGGTCTGATGTCTGGATTTGGACCAACAATGCCATCAAAAAGAGAGATGCACTTTGCGTAATTGGGTTCTTCGTCAAACTTCAAGTTCACGACATACTCAACAAATTGTCGAAAAGATTGTGGACAAATGCCACAGAGAGATTCTGGAGAGGTCGCCATCTTTTTCTTGCTGACAAGAAAACCTTTGTTGTCTCCCTGGTATCCTTGCCAAGGCAGGCGGCCACGGAGAAGAAAAATAAGTGTGTATGCAAGGGATTCAAGATCATCTCTCCTACTTCCAGTTCTTCCCAAGTGAGCATGCACACTAGCGTAGCGAACAGTTCCCCTAAATACATCAGGTCTCTGGTCATATTCAACATGCTGCCCATTACCGGCCCCTTTCCATTTCGTAGCCAGTCCAAGGTCAACAAGAAAAAGTCTCTTCTCTTCCAGGGTACCTCGGCCGCGACCACGCT

>Isogroup_386 AAGCATCTGTTCAATTCAAAACCTAAACTACTAGTGGCCCTGCCAAATGACATACCATCAAATGGCCGAATACATGCACTGACTTCTCTATACTTGGAAAGGACATGGAGTATCAATATTATCCATTTCCATGTCAACTTCCGTATGGGCAGCTGCTGAACACAAGCAAGTGAACTCGCCGTCAAAGTAAATCAACGTTCTTCCCGCTGATTCCTGTTAGGGTCAGAGACCATGTCCATCATCATCTTCTCTGGAGAGTCTTCCCAATTGCCAAAAGCTGCAAGATCAATCTTTCTTCGCTGCCCTGCAAAAATATCCTCCCTCTTCTTGTATATATCCTGTTGCCGCCTTGTTGTTTGTCCACCAAGTCCCCAAACTTCCACATCAAGTATCGAGGCCACAACAGGTAAATAACCCTGATTGGGAATGAGAGAACCATGTTGGTAAGTCTTGTCAACAGCGTGATGGCGAACTGTAACTTTAGAAAAGTCTTCATCTATAAAGATCCTCTCATTTCCAATTGATCCACCGAATGCTAGGCCAAGAGGCTTTGGGTTTGCCTCATACGTCTTTAACTGAGTTTGCAGGTGGCAATACATGATATTCTTCTCCGTACC

>Isogroup_387 GGGCAGGTACAACTATGCATGCCACAAGGCTCATATATCCTACTACTCTACAACTTGAGCTGCCTATACATACGTATTACATCTGTGGTCTTGTCTGGACTATGTAGATGTTCCCATCCTTCACTACTCCCTCAACATCCTGTGGTGACCCATAGAGCTCCTCAATGGCATGGCCGGCCCGTGCAATGCTTGAGAGGACTGAGTTTCGGAATCCAGAGTCTGTGATGAGAGCGTCGGTCGTGTAGTCAAGCACAACTTGGTCTTCCTCGTCCATAGGGACACTATCATACAGTCCTGCTCCAGCATAACCTTCCAGATCCTCGCCGTTAGAATCTGAACGGAAGATGATGGATCGCTTTATGAAGAGACCAATTGGCTTGCTTGGGTAACCCAATACCTTGGGAGAGTCAAGGTCATCTTTCTTACACACGAAGCTCATGGCACGGCCAGGATAGGCTCCCACAAGTGTCTCTCCGAGTCCTTTCACCACTTCAGCGTATATCTCAGAAGAATCTCCAGATGATGGGTTCGTAGTATGAATGACAAAGGCATAGTCTGCGTTGACAATTTCTTGTACCTCGGCCGCGACCACGCTAGTCGTGGTGTGGTCGGCG

>Isogroup_388 CAGGTACGTCTACCCTTCAACTTCATCTCCCATGCAATTCCAGTTTAAATTTTGTTGAATGCCACAATATCACAGCTCTGGATGTATTCATTGGCTGGCTCAACTTCAAAATGATCCTCAATGAGATTGTCAACCGAAACAAGGTCATCCACAATGGTCATCATAATTTGTAGCTTCTTGATGCCATACCCAACTGAAACAAGTTTGGAAGCACCCCAGAAGAGGCCTTCCATCTTCACATTCCTAACGGTTTCCTCAAGCTTTTTCATGTCAGTTTCATCATCCCATGGCTTCACATCGAGCAGAACTGATGACTTCCCAGATTCTTTCTTCTTGGTAGAAGCCTTGACAGACGCCGCACGTTCTTCTGCTGCTTTCTTCTCCTCTTCAGTCTCCTCGCCAAATAGATCAACATCAGAATCATCATCTTCATCAGCCGCTGGTGCCCTTTGGTCAACAACTTCAGGAGTTGATGCTACAAAAGAAGCAGATGACTCAACCTTGACACCTTCACCCTCAGCAGCCACTCCACTGGACCTTAGTAGAGCACTGATATGATCATACCACCTTGTGGCATTGACATAGCTTGACTTGGGAGCAGATGAAAGTGCAGT

>Isogroup_389 GAAGCACAACAGGTCAAGGCCCCGCCTGGAATCATCAGATCAAGGGTCTTCTTGAAAGTCGGCGATGCCTGTCCTGTCTTTGACAGAAGCACTTCTTAGAGAGCTCTTGTGGGGTGATTGAATTTGTCGTCTCACATGAAGTCCTCTTCTAGTTCCTGCAAGGTTTTGCTACCAGACGCAACCTGCTTGTTGGAGGTCATCTTGTCCTGGACCATCAGGCCCTTGTAGCTCCTTATTTCAGCCTGCTTCTCCTTCTCGAGCCTCTCCATCTCTTCCTTGCGTTTTTTGTCTCTAAGCTGCAACTTTCTTTCTGCCTTCTCAGCAGCACTCACGGCTTCTCTTTCAGCCTTCAGGTCAGGCGTCCGCTCCACCTTTGTCTTGTTCAAACGGTTCACCACCTCATTGAGCCGCTTTTCTACTTGAACAGTCCGAACCAGTTTAGAGTTGTGAAAACCAACTTGACCCACATCCATGGATGGTGTCTTCTTCAGATTGAACCATGGTGTATAAACCACATCAACGTTATTCACCTTATTACCTTGAATGGAATTCGCTTTAACAAGCTGGGCACAATCTTCCAGCACCCCTTCACTTATGTCATCCATTGTCTGACCT

>Isogroup_**390** AGCGGCCGCCCGGCGAGTACACCAGTGCTACCAGCTCACCGGCAAGAGCGATGTATACAGCTTCGGGGTCGTCCTGGTGGAGCTCATCTCGTCCAAGCCTGCCGTCGACATCACCCGGCAGCGCAGCGAGATCAACCTGGCCAGCATGGCCATCAACAGGATCCAGAAGTGCCAGCTCGAGCAGCTGGTGGACCTCGAGCTCGGCTACCAGTCCGACCCAGTGACTAGGAAGATGATGACCATGGTGGCCGAGCTGGCGTTCCGGTGCCTGCAGCAGAACAGCGAGATGCGCCCACCGATCAAGGAGGTGCACGATGTGCTCAGGGCCATAAGAGATGGAAGCCTGGTGGAGAAAGACGGAGGAGGCAAAGACAAGGACCTTGTCGCGCCATTCTCGCCTGACACGGTGCACGCTCCATGGGACAGCAGGAGCACCACCCCTAACACTAGCCAGTAGATCAGTGTTTGCAGCCGAGTTCTATTCATGTCTCTAGTATGCTTACTACCGAGAGCTTCTTGCCATTGCCGTGTATGAGCGACATTTCTCTTTTTTTCCCTCCAGGTAATGTAAATATGTGTTTGTTTGGCTACCAGAGCAGAGGCATTGTACC

>Isogroup_391 GCGGCCGCCCGGCGAGTAGTGATTCCCGGGGTATCCCTGGAGTTAGGAAGGAAGTCGCCGAGTTCATTCAGAGGCGTGATGGCTATCCGAGTGACCCAGAGCTTATTTACCTGACAGACGGTGCCAGCAAAGGTGTGATGCAAATGCTCAACGCCATTATCAGAAACAACAGGGATGGGATTTTGGTCCCTGTTCCGCAGTATCCGCTTTACTCTGCTGCCATTTCCCTCTTTGGTGGTTCTCTTGTCCCATATTATTTGGACGAAGAGGCTAACTGGGGACTTGATCTTGTCAATACTCGCCAATCAGTGGCAGCAACACGGTCAAATGGAATCACTGTTCGAGCAATGGTGATAATAAACCCAGGAAACCCTACTGGCCAATGCTTAAGTGTAGAAAATTTAAAGGAAATTCTGAACTTCTGCTATCAGGAAAACTTGGTTCTGCTTGCAGATGAAGTTTATCAGCAGAACGTTTATCAAGATGAACGCCCATTTATAAGTGCAAGAAAGGTTTTGTTTGACATGGGTGGTCCAATAAGCAGGGAAGTTCAGTTGATTTCTTTCCATACTGTGTCCAAAGGTTATTGGGGCGAGTGTGGACAACGTGGAGG

>Isogroup_392 TGCATCGTTCTTGAGACGTCTGAAAAAAACATACCCTTCCACTTATCTCCGGGCCCGTTTCTGTTTCTTATCTTCTTCCCTGTATCTCTCACGCCTATCTTCCCTCTGTCGCTTCTTAGTTACCAGATCGGTCTTGGCTTTCTGCACCTCATGTGCTTTCTTCTGCAGCCCGCGCTTAAGTTTCTCCTTCCTCATCTTTTCATGCTTTATTTGTATTAACTGTTGAATAGCTGCACTCCTCTTCTTCTCCTCAGTATTCATGATCACTGGAATCTTGTCCGTGGTTGGTTTCTTACTCTTAGGTCTATCCTTCGGCTTAGAACCAAAAGGAAGTTTTTCCTGCAGTTTTTTAGGGATTTCTATAGAATTGAACTTCCGCGCTTTCCGCTCAATATCCTTGTAAACAGAGTCTTTGTTGTGTGGAATAGATAAGTTGTTCGCCCTCCGCAACTCAGCCACAGTTCTCATGCCTTGCCACATCTCACCTCGGGGGTTGAAGAGAAGTGGTCACAAGATTACAGTAGGTGGGAACTTCAACGTTGACCCAAGCACGCATGAAGACAATGTCACTCATAAGAACTTTGTCCTCAAAAGTGCACCTCGCAATACCGTCT

>Isogroup_393 TCTCCCAAGCTCTTCTCGATGCTGTAAATGGTAGTGTCTGCGGTGTTTCTGATGTCAATGAGGGCTTTACGTTCCTGGTCCTTCTGTGCATGCAACTCAGCCTCGCGCACCATCTTCTCAATCTCCGAGTCAGAAAGCCCACCAGAGGACCGGATGGTGATCTGCTGCTCCTTTGCGGTGGCCTTATCTTTCGCAGAGACTGTCACGATACCATTGGCGTCGATGTCGAACGTCACCTCGATCTGGGGCAGTCCTCTTGGTGCTGGTGGGATGCCGACAAGATCAAACTCGCCAAGAAGTTTGTTGTCTGTCGCCATCTCACGCTCACCTTGTAGGACACGAATACCCACTTGTGTCTGGTTATCAGCAGCAGTTGAGAACACCTGGCTTTTCTTTGTGGGGATTGTAGTGTTCCTGCTTATCAGTCTGGTGAAGATACCGCCAAGAGTCTCAATACCAAGTGAAAGGGGGGTTACATCGAGAAGAAGAAGCTCTTTGACATCTCCACGGAGAATGCCACCCTGAAGAGCCGCGCCCATGGCCACAGCTTCATCTGGGTTGACTCCTTTGCTAGGGGCCTTACCGAAGATTTCTGAAACCACTTCCTGCACA

>Isogroup_394 CGGCCGCCCGGGCAGGTACTTCTGGTAACTCTTTTGCCAACATGTCGAATGGTGTGTCACTGGCTCCTGCAAATAGGTCAGTTCAGCCTCTTGAATCAAGCAACAGGCAACACCTAGGTCGGATAAATTCATCTTCAACAGACTCATTTAGTTCATTTGCTGGCGAGTCTCCCCACTTTCCAGATCTTGGAAGAAGTAGTAACACCTGGCAAACTGCAGTTCCATCCAACATTCAGGAACTTGGTCAGAATGGCAGCATGTCCCAAGCAACCTTGCATGTGAATGGCCCCAGGATGGAATCTGTCTCAAGCTTTACATCACCAGCAAATCAGATTACCTCTCTGGGAAATGAGATGCAGAGCCAAGTAGCATCACTAGCTAGCAATACCCTTCCGATGGTATTCAAACAGGATGCAGCGCCGTTCACCTTTACAAGCAGCACAAACTCAAGAGAAGTGCTGAATAGCAACCTTGCCTTCAGCAATGCAGGCATCAACACTTCATTTCCAAACCTTCACATAGACAGTTCTGTTATGCCAAGGCAGGGTCTGGATTGCGGGAATGCAGGCGGTGTTGCCTCTCTGCAGGATGGCAGGATTGGTCAGCAAAGT

>Isogroup_395 GATAATCGATTACAGCAAAATCATGTGAGGATCTCTACAGAAAATACAACCGACAGATAGTTCAGCACTTCTATCAGACTATTGCCACGACAATGTGAGGCGCACTCAAGGAAGAATAGATACAGATGAATATACATTAACTAAACAGGTCTACTTGAAGAATAGGCCCTTTTTTCTTCCTCTCCTCTTCTCCACAGCCATACTTCTGAACAATCTTAATACTAGAACAATTGTATCCCGTGGTGGCGTGTTTTCTGCTGGCTGGAGATTATATTCGACACCATCGGCAGACACAATCACAAACTCTGTTGCACGCTCGAATCCAGAAGGAATATTGATCGCAGTTGCTTGCTGGAATTTCTCCGTGAGAACAACACCACGCTGCCCGTTACATTTAATGCTGTAACCTTCCCTCTTTATTGCCAAAACAGCCGGTTCCCACATGTCTAAAAATCTCACCTGAGGTAGTTGGACTTGATAAGACACATGCCCAGCTTCAAGAGTTTTCCTAATAAGTTCCTTTGTCTCAGGATCACAAGTAATTTTTGCTTGGTCGTTAGCATAAACCTTGACAACCTCACCCTTTCTTTTTCTGTCGTCTAGGGGTT

>Isogroup_396 ACAAAGTCCCGATGAACGAAGAAGCACATAGGGTGGAACCAAACCAATGGTAAAAGCTATGAAATACTATCCAAAACTGACTGAATTGAACTCCTTCCATGCCTCCTGTAGAATGTCAAAAACCTTTTCTGCTATGTATTTTTGTTGCATGGCAGCTCCTCGCTGTTGCAACTTATCTGGATGAAGACATAACAATGCCTTCTGATAAGCTTTCTTAACCCCAGCTCCTTCAATTATGTTAACCAATGGAACTGGTTTCCATCCACTCTCAGGCCAGAGAACAAGTTGCAGTGTAGAAAGCAATGACCGGATATTTCCTTCTTTTCCCCTTGACCATTCCCGGATTTTTAATTCAGATATCTTTATTTGCTCCTTTTCCTTGTCATCATGAAGAATCTGACCTTCGTCGAAATGCTCAACCACGCATCCTTCAAGATCTTCCATGTGACTTTCTTCACGAACTATTGTGTCACTCATTTTTTCTTTTGTTTCAACATGGATCGACTCATCAGAAGATGGTTGTTTAACATCTTCATTTCCTGAGAGTTTGCCTGTTCTGGTGTGTATTTCATCATCCACTGTAAGAACTGGTTTCTCCATCCTACCTT

>Isogroup_397 AGAAATGCTGCTGGGTATGCTTCACCAATGCAGTCAAACAGTCCATCAAACCCGAAGCACAGGGCAGCTGGAATGCAGACTCCAGAAAGGAGGGTTTCGACAGACACACATGGCCAGCAAACTCCTGGAAGGAGCAGAATGAGGCCAAGTAACCAAGGCTACAACCCTGAAGAGGAAGTGGCTGTTCCACCATTTGGTGAATGGGATGCGGGTGATGCGGCATCAGGTGAAAAGTATACAGGTATCTTCAACAGGGTGAGAGATGACAAATTATCGCCTGACTCTTCTTCCAGGCAGCAGCAATCTGGTAACCGCAGACAAGACAATAAGGTTGAACAGGCGTGCTCTTGCTGTATACTTTGACAAAATGGGGATATGATCGCAGGGCTTCTGAAGAACTTCCTGGATTACTTGTGGAACAAATTGCCATTCTTGTGAAGAATTTGTCACAAAACTCCTTCATGTGTTACTTTCCTATGCTGCTTAGTTATTACTGTTGGTGTGAGTATGTACCTGCCCGGGTCGGCCGCTCGAAAGCGTGGTCGCGGCCGATGTACGAGGTGCATCGGCTGGTTGTCAACAGGGACCCCAAGTTCACCAACTTC

>Isogroup_398 CGCGGCCGAGGTGCCCGATGCTCACGGCCGTTGCCCTCAGCGATGGCGGTAGTTTCTTCGACAAGCTACCCAGAGCATGGTTCATCTATTTGTTCATCGGTGTTGGTGTTGTTGTCTTTCTCGTCTCTCTATTTGGCTGCATTGGAGCAGGGACAAGGAATACTTGTTGCTTGTGTTGTTATTCTTTCTTGGTCCTATTGTTGATCCTTGTTGAGGCTGGAGCTGCTGCATTCATATTCTTCGACCGTAGCTGGAAAGATGTAATTCCAGTGGATAAGACACAGAACTTTGATGAAATGTATGACTTTCTGAAACATAACTGGGAGATTACAAGATGGGTTGCTCTTGGTGTTGTTGTTTTTGAGGTATTGCTTTTCTTGTTAGCTCTGATTGTTAGGGCAATGAACAAACCTGCTGAGTATGACAGTGATGATGAAATCATTGGAACTGCCCGAAACAATAGCATCCGTCAGCCTCTTGTCCATTCCCAGAATGCTCCGGCTACTGGTGTTCCTGTCCCAACACTTGACCAACGTGCGAGCAGAAACGATGCATGGAGCCAAAGGATGCGAGAGAAGTATGGTCTGGACACGAGCCAGTTTACGACC

>Isogroup_399 CTCATTCCTAGATCCGCTTTCCCATTCGATTCCCGCGCATTTGACTCCAAAAGCAATCCTAGCAAGTCATCACTGCTAGCTTCACCATTTTTAATAGCTCTGTCTTTTTTTGCGATAATCCCTCGCAGAATTGAGCGGATCTCACGGTCAATTGCTCTCATCCTTCTGTTGTTTTTTGTAGGTAGGAACCAATAGCCTGGAATAAAAAGTGTCTGAAAACCCTGTATTAGGCGTTCGGCTAGCTCCCCTTGCAGCTGGAATATTTTCATCCCCTCCTGATAATTGCTACCAAATGCTGTTCGCGAAATGACATCTCCAGTAAGATTCTGGAACTCCGGCCAGATGTCAATCTCAGATAATCCTTCGGCGGACATCGAATTCTCCCATCTTGTAATCATTTCTTCGCAACAGGTAGAAAAGACTGGCAGCATCCTCTTTATCTTCTCATGGTGAAAAGCAGGATTCAGGATTCTCCGGTGCTTTGCCCATTTCTCGCCTTCATGATTTACAACCCCGTTGGCAAGCAGCTTCCCCATACGGCTAGGCTTTTGTTTTCCATAGTGTCCAAACTTGTTGGAAAGAACTTCTCTCACAAATTCCGGGTCAG

>Isogroup_400 CTTTACTGGCAACTTCAACGAGCTTCAGAAGCACACTCAACTAAAGCATCCAGATTCACGCCCTTCGGAAATCGATCCTGCCAGGCAAGTTGATTGGGACAACTTCCAGCAATCTTCTGATATTGTAGATGTCCTCAGCACAATACATGCACAAGTTCCCAACGGTATTGTTCTCGGAGATTATGTCATCGAATATGGGGACGATGAAGCTGGAGAAGACTACGAGGTTCTCCGCAGGGTTAGGAGGAAGTGGTGGTCCTTTATCTGTTGCAAAGCCTTCTGCAGATATCCAAGACGCCGAAGAAGAGGAAGATCAAGGGAAAGTAGAGGCAGTGGAAGGAGGAACAGTAATCAGGCTCATCTGGAGAACTTCAATCTCGAGGTTCCGACACAAGCTGTTGACTTGAGGGAGCTCAGATTTGATGAAATCGATGATGAATACATTGTCACGGGGGCATTACCTAGTATGTCAACGCCTGGAAGAATGGCCAGTTTCCACTACAGGGATACAAGATATGGCCGGTGATCTGCCTACTTATAGAACCAGCTATTAAAGGAAATGAGGATCTGTAGCCCACATGTGATGCCGCAACCGTCAAGACATCG

>Isogroup_**401** AGGTACATCTTCGCCTTCCCTGGCGATCACGTCGAAACGAAGAAGCTGAGGTCACTGAGTTTCCGTCTCTGGACTTTTCCTGTTCTTGTGGATGGCCCGTTCGGGGTTGTCTCTGCTGTTGAATTCATCGGGATTGTCCTGTTTATCATTTATGTTGTCTTCTCAATGACATATTATGTTGTGGAGACCGTGAGCCTCGTCTCAAAATCTCACTTGTCGCCGATTACTAGCAGTGAGTTGATACTGGATTTAGTTGGCGCCCGTTTCGGGTCAGTTGGGTTGTTTTGCATGATCTTCCTGTTCCTGCCTGTCTCGAGGGGTTCAGTTCTTCTCCGCCTTATCGATATTCCATTTGAGCATGCTACTAGATACCATGTCTGGTTGGGACATCTCACAATGGCTCTGTTTACACTGCACGGCCTGTGCTATGTTGTCACATGGTTCCTCGAGGGGCGCCTAATTGAAGAACTGATCCAATGGAAGGAAATCGGAGTGGCAAACTTAGCTGGCGTGATCAGCTTGGTGGCTGGTCTGCTGATGTGGGTGACATCGCTTCATCCGGTACCTCGGCCGCGACCACGCTAGTCGTGGTGTGGTCGGCGTCTC

>Isogroup_402 TGTCCTTAACCTTCTCTACTGTTTTGGTAATAAAACTTTCTTTCTTCATAACTGGACCAGAAGGCCCTGAAGGAAGAGGCGGCGATGCAGAAACATCTATTATTTCATCTGGAACACCCTTTTCCTGGAACTCCACTTTTTCAGAATCGCCTGCACTCTTCTGCACTCTCCTTTCTAACTCAATCAACTCACGTCTCAACTGTTCGAAACGGCGGATGTCGTTTGACTCCATGTCTTCTTTATCCACCTTGGCAGCAGAAGCCTCCAGATATTCCGCTTTCGCTCGAAAGTAAGCCTCAAGAAAGTCCGCTTCTTTCTTCAGTCTTCTGATCATTTCAAGATCCGAACATGCAGCCTTTAGATCCTCCTTACCAGAGTTGGAACTTGAAATATGCAATTCTTGAAGCAATTTTTCCAACTTAACCAAAGCTTCCTCAACACTTTCAAGCGACTTGTCAATGGAAGCTAGCTCTGTCCCAGCAACCAATACTCCGTTGTTATTTTTTGATACATCAAGCTCATTCATGCAGTCGCTCAACATGGCATGTCCCTTAGATAGGAATCTTGCTGCCTTCACATTTGAAGGGTTCTCAAGCCATGAGCTG

>Isogroup_403 TCCCTGGAGTATGGCTTAGTCCATGCGTCACTCATCAGGAGTTGGGGTGGGTGGGGAGCGCCCTGAGTAATAGAACAGGAAAACACATGTCAACTACCATGTTCATGTGTATTGAGTTGCATGCACCCAGCCAGTTAAGCCAAAAGCTCATGCTATTGGAAAAATGTTTTAGTTATTTACTGAGACAGTTTGGAGTAGATTACCTTCAGGACATTGTTGTTCACATCTGCTATGCCATTTTCTACCTGTGCAATTTCTTCCCTGATGGAAATAAGGGCATCACAGAACCTGTCTAGTTCAGCCTACAAGACAAATGATGTGGCAGTAACCAGTAAGCTAATGCAAAAACAAACGTTGGTGCAAAGTCAGGCAATAGATTAAATTGTATATGCTTACCTTGCTTTCACTTTCAGTGGGTTCAATCATAAGTGTGCCTGGAACAGGCCATGACATGGTTGGTCCATGGAATCCATAGTCCATCAAACGCTTCGCAACATCCTCAGGCTCTATACCAGCAGTTGCCTGTAAAAACAATTATCCAAGATTCAGTCAGAGAACTGAAATGGATAAACGCAGGGTTCAAGTGCTAACATAGTTCGGGCAA

>Isogroup_404 AAGCTGGAACTGAGAAAATCACCAGGGATTGAAGAGATCTCAGCTCCCAACCGATAATTTACCCTTCAAACGAATGCATCGAGAAGTTGATCCCTGCCCTCGAAAAACCTTCTCATTTAGATATCCTGGTGTGCACTTTGGTTAATTGGACACTGGCTCCAGTTCTAAAATTTGTTCTATTCCAGCTGTAACCAGGCTTCGACTTGTTGGCGCATTTACCTCATGCTAAGGTTGCTCAGGTCATCCACAATATGCACAGTCCCATTCAAGCTGCCTGCATCTCCCTCTTTGATTTTTGCTTCCGGGTTAAGCACATTGCCATTTTCTGTTGAACTCTCGAAACTACTGTCAGCAATATATGACGTAGCACTCCTTGCACCTTCAAAGTAGGTTGAGCATGGAAAGTATTCAGCACAGCGTATCTTCCACCTATTTGCAACATCCTGTTCAGCAAAAACAGTATCCAGCAATTTATGAACATTAATACAATCTTGAATGCCCCATTCCTTAATGGGACCAAGCACACCGTTTCCTGACGCTGTGCTATTTGTTGAGTCTTCGATTAATTTAATAGCCTGCAGCTTCTTATCTGGCTCCAAGAG

>Isogroup_405 ACCATTTTCCCGGAGCATCATAGTCCACCCTAGATCCCATATTATCATAAAGCCATGCTTTAATCTTCCCGTCAAGGGAAGTTGAGAAGATAAACTGAATACTCTCTTTGTGGTGAGGGCAAATAGAATACACAGGTGCCTCATGCCCTTCAAACGAAAATACTTTCTGACCATGCATATCCCAGACCCTTATCAGCTTGTCATCTCCACAAGTGACGACACAAAGTTGCTTATTTGGCCGGGAGAATGCTATGTCATTAACTCCTCCAGAATGAGCCTCGATCTCTAGAACTTGGCGTGTTTCATTTGGTTGCTGATATGCGTGCAGATGGATCAAGTGTTTTGTGAATGCAACTCCTATCAAGTCTCCATCTGGGCTCCAAGTAACTCGGTTAATGGACATGGAAGGGTCCTTAGCCATGGCACTCTGAAATTGTGGTGAACATGCTTGCATGTCCCAAATTTTGAAGGGCTTTGAGACCAGCCTCTCACGCAGACCAATCTCCCAAAGCGTAATATCACCATTAGAAGATCCAACTAGTAGTAGAGTATGACGAGAAGGATGAAAGTCCATGCTAGTTACATTAGATCCCTGTGATAAT

>Isogroup_406 AGGTACTACATCTAAGTTAGCGCAAAGGCTTCTTCTTGGAAGGGAAGCTGGGGTTCTTGGTAGGGATGGACTACTTTGTTGATCTCATCTGCGTTGAGAGTCTCACGCTCAAGCAGGGCATTTGCTAAAGCATGTAATTGCTTCTCGTGCTTCTTTAGCAAACGTGTGACCCGCTCATAAGCTTCTCTTAGGAGTTTGACCACCTCTTCATCTATTCTTGATTGCATGTCAGCACTTGGCCGTTCTCTCACATGCACAGGACCAATAGCATCACTCATCCCACAGTTTGATACCATATACTGAGCAAGCTCTGTTGCAGTATGAAGATCGTTTCTTGCACCAGTTGTAACGTTTTCTTCCCCAAATATAAGCTCTTCAGCAACCCTTCCGCCCATGCAAACATCAAGACGTGCCAGGAGTTGTTTCTTGCTGATAGAAGTCTCATCCTGTGAGGGAAGTTGTGTAACCATTCCAAGGGCAGATCCACGAGGCAGGATAGTTGCCTTGTGAATGGGATGAGCACCCTTGGTATTAAGTGCAACAATAGCATGCCCACTTTCATGGTAGGCAGTAAGCTAAGGAACCAAAAAAAACAGTTA

>Isogroup_407 AGTAGGGGCGAATTAACAACTTACCCGACCAAGAGCACTGTGGCAGCCTGGAAGTCTGTTCTAGCATACAAATCCTGCTTGACCTCGGAGATTGGATTATGTAGATCGAAAGCCAGGAACTTTACTAAGACCCATCTTTCGAAGAATGAGTTTGGGTATGGCTGGTGGGACGGCAAGCCCCATGCTGCGGCTGAACTCTTCACCGAGACTAACAAGTCTGGACTTGTTACCGCCGATGGTCTTGTTCGAATTGTAGTACCTGCCCGGGCGGCCGCTCGACTGGTGCTGCCAACATTGGGGCTCTTGGCACTGCACTTCTTTGTTATGTAACACCAAAGGAGCATCTTGGGTTGCCTAACCGTGATGATGTTAAGACAGGTGTGATATCCTATAAAATTGCTGCTCATGCTGCTGATTTGGCAAAGCGTCACCCCTACGCACAAGCATGGGATGATGCATTAAGCAAGGCAAGATTTGAGTTTAGATGGTTGGACCAATTTGCTTTATCTCTGGATCCAGTCACTGCTATGTCTTTCCATGATGAAACATTACCATCTGATGGGTGCCAAAGTAGCACATTTCTGCTCGATGTGTGGTCCC

>Isogroup_408 ATAACAGACAGGGGTGGTAGGTTTCACTTAAGGTGGGGTTGTAGAGAGGTTATCTACGGGAAGTCGCCTGATGGCGAGACCTATGTTAAAGGCCTTCGCATCTCCAAGGCTACGAGTAGCGAGATAATCAAAGCTGATGCATATGTTGCAGCTTGTGATGTCCCAGGGATCAAAAGATTACTACCATCAGAATGGAGGGAATGGGATATGTTTGACAACATATACAAGTTAGATGGTGTTCCTGTAATCACTGTTCAGCTTCGCTACAATGGCTGGGTTACTGAAGTTCAAGACCTTGAGAAATCAAGACAATCACAAAAGGCAGTTGGCTTGGACAATCTGCTCTATACTCCAGATGCGGACTTCTCCTGTTTCTCAGACCTCGCACTTTCATCTCCTGCTGACTACTACATTGAAGGACAAGGCTCCCTGATCCAAGCTGTGCTAACTCCTGGTGATCCTTACATGCCATTGCCGAATGAGGAGATCATAAGCAAGGTTCAGAAACAGGTCTTAGATTTGTTTCCATCAGCTCGAGGGTTGAAAGTTACATGGTCCAGCGTGGTAAAGATCGGACAATCTTTGACCTTGCCCGGG

>Isogroup_409 TTGGATACAAAATATTCTCTAGCAATATCATATCCTGACATCTCCTGGCAAACATCGTTAAGAAGGCAACCTCGAAAGCTTGATCTGTCAGAAGTTTCTTCTCCATAGCACGTTACCAGTGCCTGAATGACACAAACAATATAGAACAGATGCACAACAGGGACGAAGAATTCTGAGGATGTTAGAACTTGAACAGGCAGACAGAAAAGTGCTGACATCAATGATGAAAACGGATCTTGAGCAAGGACTGGATCAGCACATTGCTTCCAAAATTGAATATCAGGAAATATTTCACCCTCACTTTCTCGATCAACCACAGGTAGCAAAGTTCCTTTTCTCTTCGTAGCATCTAGAAGATCTTTATCACCAGAAATACCAGAGCAAATAGACCCAACTAACAGTTGGATACCTTCATATCTCAGAAGAACCTCACGGCGATTCAGAACACGTGCCGAGTGAGAAACACGAAACAATAGAGACAATATGAATCCACTCGATGAATTCAATTCACTGCGCAGAGATTCTAAGCAAGATGTTGATTGAGCAGAATAGTTTGACATTCTACCACGAGAAGCGATCTCTGTAGAAACGACAGA

>Isogroup_410 TTGTTAGGGTAATAGCCCGGATAACATTTATCTTGGAAGATGATCCAGATATCATTGGTGTTGCTCGTGAATACAATCTCACCGCACTATTTACTGATCTGCTTCAAATGAATGGTCTTGATACTGTCCAGATTGTCTCTGCCACTGCATTGGGGAATCTCTCTCACCAATCAAAGCATCTAACAAAGATACTGCCACCTCCCACCCGAGGATTGTGTGTTTCGATATTTCCATGCATCAGTCAGAAGTCTGTAGCAAGCGGGGAGTGCAGAGTTCATCATGGGATATGTTCTTCAAGGGAGAGCTTCTGTCTCTTGGAAGGGAAGGTTGTGGAGAAGTTGGTTGCTTGTTTGGACAACAACAATGAGAAAGTGGTTGAAGCCTCTCTAACAGCATTGTCCACATTATTGGAGGATGGAGTGGACATTGATCAGGGTGTGATGGTCTTGTGCGATGCAGAAGGGGTCAAATTGATTCTTGATGTGTTGTGTGAGAATCGGACTGAGGCACTTAGGCAGCAAGCGGTCTGGGCCGTGGAGAGGATTCTCAGGATGGACGAAATAGCTTACGAGATCTCAGGAAACCAAAATGTTGG

>Isogroup_411 CTAGGTTCAATGCTTCCTATTGAGGAGATTGTAGCATGTTCAAACAGAGTCGGAGCTAAAGTTCTTGTAGATGCTTGTCAAAGTGTCCCCCATATGCCAGTTGATGTTCAAAAGCTTGGTGCAGATTTTCTTGTTGCATCCTCACATAAGATGTGTGGCCCTACAGGCGTCGGATTCTTGCATGGAAAATTTGAGATCTTGTCATCTATGGAGCCTTTCTTAGGTGGTGGTGAAATGATTGCAGATGTGTTCCAAGACCAATCTACATATGCTGAGCCGCCTTCTAGATTTGAGGCTGGAACTCCTGCCATTGGAGAAGCTATAGGATTGGGGGCAGCAATAGATTATTTGTCAAACTTTGGCATGCAGAGGATCCATGAGTATGAGAAAGAGTTGGGGGCGTATCTTTATGAGAGCCTTCTTTCCGTTCCAAAGGTTCGGATCTATGGTCCAGCTCCTTCTCAAAGTGATCACCGTGCTCCTTTATGTTCTTTCAATGTTGAGAATGTCCATCCAACCGATATTGCAGAAATTCTTGATCTCCAGCACGGCGTGGCGATTCGGTCGGGGCATCATTGCGCACAGATCCTGCACC

>Isogroup_412 CAAAATATGAGCGATCCAGCATTCCACGCTTATTTTCCTCCTATTTTTACACAGCACTGCTCACCGGTGAGATATCTTTACCAAAAAGCCCAGCGATTGCCTTCCCGGGATCGTCTTGCTTGATGAGGGACTCTCCAACGAGGACCGCTTTGACTCCGGCATTCTGAACGAACGAAACATGGTCAGGCGTGAACAGTCCAGATTCTCCTACAACAATAATGTCCTTCTGGGCTATAAGCTGTCCCCTTTCACCCTCCAGAAGCTTTTTCGTGTTTGAGATATCGACTTCAAAAGTCGCAAGATTACAATTATTGATGCCAATGAGCTGAATTCCATCTATCCCTAGAACACGGTCCATTTCCCTTTCATCATGCACCTCAACTAAAGCAGCCATCCCAAGTTTTTTGCATATTTTCAGCATATAATTGATATCACGGTCAGGTAACACAGCAGCAATCAGAAGAACAGCGTCTGCTCCTTTGGACCGTGCATAGTAAAGTTGCCAGGCATCGATGATGAACTCTTTGCAGAGAAGAGGACACTTTACTCCGGCATTGCGAATAGCCTCCAAGTTTTCATAACTTCCCTGAAA

>Isogroup_**413** CCCGGCGCAAGATACTATGGTGGAAACGAATACATTGATATGGCAGAGTCGTTGTGTCAGAAACGTGCTCTGGAGGCTTTCAATTTGGACCCAGAGAAGTGGGGAGTGAATGTGCAGCCACTATCGGGTTCACCTGCCAACTTCCAAGTATACACTGCTGTGCTGAAGCCACATGATAGAATCATGGCTCTGGATCTTCCTCACGGTGGACATCTTTCTCATGGTTACCAGACTGACACAAGGAAAATCTCAGCAACTTCAATATTCTTTGAGACAATGCCTTACCGACTGGATGAGAGCACTGGTTTGATTGATTATGACCAGTTGGAGAAAAGTGCCGTTCTTTTTAGGCCAAAGTTGATTGTTGCTGGTGCAAGTGCGTATGCTCGCCTTTATGATTACGACCGCATGCGCAAGATCTGTAACAAGCAGAAGGCAATACTTCTCGCAGACATGGCACATATCAGTGGCCTAGTTGCTGCTGGTGTCATCCCATCTCCTTTTGAATATGCAGATATCGTTACTACCACTACTCACAAGTCACTCCGTGGACCACGTGGAGCCATGATCTTTTTCAGGAAGGGTGTGAAA

>Isogroup_**414** CAAGTTCTCAAAGCGATTGGTAGTTGATGAAGAAGGGTGCTTGTCGTTCCCTGGGATATATGCCAATGTGCTGAGACCAGACACTGTGAAAATTAATGCTCAAGATGTTAGTGGGGCAAAGATCAAAGTTAGATTATCTCAACTATCTGCAAGAGTGTTCCAGCATGAGTTTGATCACTTACAGGGGGTTCTTTTCTTTGACAGAATGACGATGGATGTTGTTATGAGCATACGTGAACAGCTGAAGGATCTGGAGGACAAATACGAGGAAGCAACTGGACAAAATAGCCCTGAAACTGTTGAAAACTATAGAGGGACAAAGGATGCCATTAGTTTTTCAAGATGATGATTTTGCACCGGTTCTGCTCCAAATGCGTCATCAAGCCAAGCTTCACCTTGAAGAGTTTGTGCAACAGTTTGTTTGTGCATACTGCTGTCATTCTAGTTTGGGCAGTATTTTATGTCTTGATATTGCAATATCATTTAAATCACATGAGGAAATCTTACAATGTATGAATGAAGTATGATTCCCAAGGATGAGTATCTGAATAGTATTTTTGTTCACGTGCTATGATATTTAGGATATATT

>Isogroup_415 CGAGGTCAAGAAGTTCTGCCTCGAGCCCACCTCCTTCACCGTCAAGGCGGAGGGCATCCAGAAGAACGAGCCGCCGGCCTTCCAGAAGACCAAGCTCATGACCCGCCTCACCTACACCCTCGACGAGATGGAGGGCCCCCTGGAGGTCGGCGCCGACGGAACCCTCAAGTTCGAGGAGAAGGACGGCATCGACTACGCCGCCGTCACCGTGCAGCTCCCTGGAGGCGAGCGCGTGCCATTCCTCTTCACCGTCAAGCAGCTCGTCGCCACCGGCAAGCCCGAGAGCTTCAGCGGGCCATTCCTCGTGCCCAGCTACAGGGGCTCCTCTTTCCTCGACCCAAAGGGCCGTGGTGGCTCTACTGGCTACGACAACGCCGTGGCGCTGCCTGCCGGAGGAAGGGGAGACGAGGAGGAGCTCGCCAAGGAGAACGTCAAGAACGCCTCATCGTCCACGGGCAACATCACCCTCAGCGTTACCAAGAGCAACCCAGAAACCGGCGAGGTCATCGGCGTCTTCGAGAGCGTGCAGCCGTCCGACACCGACCTCGGGCGCCAAGGCGCCCAAGGATGTCAAGATCCAGGGTGTGTGG

>Isogroup_416 AAAAATCTTGGAGACAAATTTGCCGAAAAAACAGGGGAAAGATTAACGCAAAAACAATTCAAGAACAAATGGGATTCTTTAAAGAAGGATTACACGGGGTGGATGGAACTGCAAAAGGCGACCGGGCTTGGTTGGGATCCTATAACAAAAACTATGGATGCAGATGATGATTGGTGGAAGAATCACATAGCTATTCGTCCTGACCATGCAAAATTTAGGAATGGGCCACCTCCCAACTTGGAACAACAGGATGTTATGTTTAGGAAGGCACATGTCACAGGACAATCAGCTGCCATTGCAGGTCAGCAAGAAGGAAATGGCAATGAGGCTCCAATAATGCTTGATGATGATACCTCGCCTTCCAACAATGCAATAGCGAAACGCAAGCATGCAGAGAATGAAATGGGAACTGGGAAACGCAAGCATGTGGGCTCGGATTTTTTCTCGGCTTACAACAATGCACTGAACACAATTGTATCAAGGCACACCGAAGGAAGTTCTAGCAGTAAGGATGATCAGGTCCCAACTATGAAAGAGTTTTTAGGAATGGTTCGAGATTGTGGAGTGAGTGAAGGAACTGCTATCAT

>Isogroup_417 GCCTACGGGTTCGCCATCATTCTGCTCACCGTCGCCGTGAAGGCAGCGACGTTACCGTTGACGAAACAGCAGGTCGAGTCAACTCTGGCAATGCAGAATTTGCAGCCACAGCTCAAGGCGATCCAGAAGAGATATGAAGGCAATCAGGAAAGAATACAGCTGGAGACTGCTCGGTTATACAAGACAGCTGGAGTCAATCCTCTAGCAGGATGTTTGCCAACTTTGGCGACGATACCTGTCTGGATTGGACTTTACCAAGCTCTTTCAAATGTAGCAAATGAGGGGTTGCTGACAGAAGGATTTTTCTGGATTCCATCTTTGGGAGGCCCTACAACAATTGCTGCTCGTCAAAGTGGTGCTGGCATTTCATGGCTCTTCCCTTTTGTGGATGGTCATCCACCAATAGGCTGGCATGACAGCATATGCTATCTTGTTTTGCCTGTGCTACTTGTTGCTTCTCAGTTTGTCTCCATGGAGATCATGAAGCCACCCCAGAGTACCTCGGCCGCGACCACGCTTTTCTTTGAGACAATGCCTTACCGACTGGATGAGAGCACTGGTTTGATTGATTATGACCAGTTGGAG

>Isogroup_418 TATCAAGTCAGTCACAAATGAGGTTCTAATTCTGGCGGCGGAATCAAGAAAGATGAACTCTGATGTTTACAATATATGGTCACTGGCCAACGATACAGAGAAAACTGTTGAAGCTCTACATTCAGATGTCAAAAAGGTTCAAGTTCTAACAGATGAATCAAGAAAGTTGGGTTCTAGTATTCGCCAGGTATGGTCATTGGCAAAGGATACTGGAAAGAAGGTCGAAGCTCTGTATGCAGATATAGAAAAGGTTCAAATTGTAAGTGATATGTCAAGAAAGATGGAGTCTAATATGCATAAAATGTGGTCTTATGCAAAGCAGACAGAGAAGAGGGTTGAAGACCTATATTCAGATGTCAAAAAAGGGCTTCAAGAAGGGAGCGAAACTTCCATTCTGGCAGAACTGATAATGCCATCGACTTGATGGACTACCATTGGTCTGGGCACGGAGTAATAATATGGAAAAAGCTAATGGGCGTTGTTTCAGCGAATATATGTTACGCCCATCTTTGATATCGTTGGAGGGAGCTTGTCTTTTTTATTGACATGGTTAGGGATGCAGCGAGATGTAGTCTCGAGTGCAATC

>Isogroup_419 AGCAGAAAGTAGACCCTAAGGTTGCGTCTGCTGAGGCCAGAGAACAGTATCTAAAGTTGAAAGAAAATTTGCAGTTGCTTACTTTAGGTATTGGTGGCATTGGTTTGGTTTCTGCTTATGTGTCATACACTCCTGAAATTGCTGCGAGCTTTGGTGTAGGCCTGATTGGATCTGTGGTGTATCTCCGTATGCTTGGAACCAGTGTGGATTCTCTAGCAGGTGGAACTAAAGCAGCTGCCAAGGGCGCAGCTGCACAGCCAAGATTGCTTATTCCAATGGTTCTTGTGATGATGTATAATCGATGGAATGCGATATTAGTTCCAGAATATGGCTTCATGCATCTTGAGTTGATACCCATGCTTGTTGGGTTTTTCACCTACAAGATTGCTATGTTTACTCAAGCGATCCAGGAATCTATACCTGATGTTGGAAACCGTGAAGTTTGAACACTGCTCTGGTTAATACTCATAGAGAAAATAAAGAGGAGGCTGTTGCCCTGAGGGAAGATTGGTTACCACTCATCAATATAGTAAGGTCTTTATTAGGTTTTAGAAGAGTCTGATCAGGACATCTCGTCAAGTACCT

>Isogroup_420 GCGGCCGAGGTACTTAGAGACCAACGGTTTGAACTTCCCTGTGATCCATCACATTGATTTCCCTAAAAGCATCGATAGAGATGGTCTTGTTATTGGTGCTGGGGCCAATGTTGGTGCTCTTCTAGTTGATGGTCTTGGTGATGGTTTACTTCTTGAAGCTAATAACCAAGAGTTTGAATTCTTGAGGGATACATCCTTCAACTTGCTACAGGGTTGCCGGATGCGCAACACAAAAACGGAATATGTGTCTTGCCCTTCTTGTGGGCGAACACTGTTCGACCTCCAAGAAATCAGCGCTGAGATCAGAGAGAAGACCTCTCATCTGCCAGGAGTCTCTATCGCTATCATGGGTTGCATTGTTAATGGGCCAGGAGAGATGGCCGACGCCGACTTTGGATACGTCGGAGGTGCTCCCGGGAAGATCGATCTTTATGTCGGCAAGACTGTTGTGCAACGGGGGATTGCAATGGAGGGTGCCACTGAAGCCTTGATTCAGCTCATCAAGGACCATGGCCGTTGGGTGGATCCTCCTACTGAGGAGGAGTAGGCTTCAGGCTGTTCGCCGTAGTTCGTTTGTACCTG

>Isogroup_421 ACAGGAATCTCTTCTGACTCGGTGCAGCTATCAATATTAAATCATCCAAAATTGAAGTTAAAATCTGAGCATGTTCGTGTAATTGATGGAGCCAAGTTGAGAATATATCCAGCAGGAACAGATAAGTCCAAACTTCAGTTTGAGCTGCACAATCTCAAATCCATGCTTCCAAAAGTGATTGTGAAGGGTATTCCAACTGTTGAAAGAGCTGTTGTCAACCCTGTTAGAAGACGTGACAAAAGTATCGATAGATACAACCTATTGGTCGAAGGAACAAACCTCTTAGCAGTATTGGGTGCTCCAGGAGTTGATGCTATGAAAACAAAAAGTAACCACATCATGGAAGTGAACCAAACACTTGGAATTGAAGCTGCAAGGAGATCCATCATCGATGAAATCCAGTATACATTTGAAAGTAACAACATGATCATCGATCTGAGACATATGATGCTCCTGGCAGATCTGATGACATACAAGGGTGAAGTTCTCGGCATCACCAGATACGGCATTGCGAAAATGAAGAGCAGCGTGCTTATGCTGGCCTCTTTCGAGAAAACCTCGGAGCATCTGTTCAACGCTTC

>Isogroup_422 ACAAAGCCGCAGGGTCAGCAGCCTGATTTTGGAGACCCGGTTGTTCTTTCTACAGACCGGGGTGGTTGCACAGTTGAAGACTTCTGCAATCATATCCATAGGAGCTTGCTTAAGGATGTGAAGTATGTGCTTGTGTGGGGAGTCAGCGCTAGGCACTATCCACAGCATTGTGGTCTTAGCCATGGTCTGCAAGATGAGGATGTCGTCCAGATAGTAAAGAAGAAGGAAACGGACGAGGGCGGGCGAGGCCGTTTCAAGTCACACACCAATGCACCTGATCGGATATCCGACAGGGTGAAGAAAGCTCCTCTGAAAACATGATCCGGTGGTGAGTGGACATCTCTGAATGGATGATGATGTGGAATTTGGACATCTCTGAATGGATGATGATGTGGAATTAGTTGAACCGGATGCTACTTTTTGCAACCCTATGTTCTGCCAGAAGGGGCCATACTTGAGCACCTAACCATCCACCATATGAGAAATGTTGCTTTTTATTCAGGCAGTAGCTCGTGCTATATATGAGGAGGCTTGTGTTGCCATACGAATGTTTTTGCCAGGAAACTTCGCCAAAAGGAGTT

>Isogroup_423 AGCGGCCGCCGGCGAGGTACACAATCTCAATGTCTTTCATCTCTTCGCGAATGGCATCCACCCTTTCCACTGAGCGGTTCACCACCACAACCTTCGTGCATCCTTTGGCAATCAAATGTTTGACCACTAGTTTGCCCATTTTGCCAGCACCAATCAAAAGCATCCTAGCTGACAAGCATTCGGACTTTGGAAGCTTCATCAGGGCCAGTTCAACTGCAGCTGAACTGACAGAAACAGCACCAGCTGATATGTTAGTCTCACAGCGGACACGCTTTCCAGCTGTGATTGCATCCTTGAACATCCTATCAATGTTCTTCCCCAAGCCTCCACTGTTTTGCCCATTTCTGACAACTTGCTTAACTTGAGCAAGAATTTGTCCTTCTCCAAGAACCAAAGAGTCGAGCCCAGATGACACTTCAAACAGATGGCGTGTAGCATCACTGTCACGCAACATAAAGAGATGCTCCCTGAGCTCAGAAGCAGGAATTCCACTCTTCTTTGACATCCAGTCAACAACTTCTCTAATACCACGGTTCCACGATAAAGCCACCACGTATATTTCCATTCTATTGCAGGTACCTG

>Isogroup_424 AACCAGCGGCTACCTTGGTGCTGAGTATCAGGAATCAATGGACTCATCTTTCTCTCAAGTTTCACCTGAAACACCAAGTTCTGCATCTGAAGCTCGGCAGTTTGGCTCTCCAGCGTTTGGGCTGAGGCAACATTTTGCAGCTGAGAGGAAATGGGCTCTTGGTCTTCAGTCTCGAGCACATCCACGGGAGATTATAACCGAAGTGCTCAAAGCTCTGCAAGAGCTAAATGTTTGCTGGAAGAAGATTGGGCACTATAACATGAAGTGTAGGTGGAATCCTGGCTTTGTGGAGAATATGATGCATAATAACCATGGCTTCGGTGTGGAGTCTGCTATAATTGAAACAGATGACCTCAGTGAGAAATCAGCACACATCGTGAAGTTTGAAATTCAGCTATACAAAACAAGGGATGAGAAGTATCTTCTCGACTTGCAAAGGGTCAGTGGACCACAGCTCCTCTTTCTGGACTTGTGTGCTGCCTTTTTAACTCAACTGAGAGTCCTTTGAGTTCTGAAGATGGTGACTTCCCAACTGCAATCTCGTCTGCATCCTGTGAAGTGGGAAGTTACAATCAAGATG

>Isogroup_425 AGCCTTTCCAATTCTTTATGCTTGTGTTCCTTATCTCTATCTCTCCTGACTCTTTCTCGTTCACGCTCGCGCTCATATCTTTGAAGTTCTTTTTCTCGCTTGTCCTTATCATGCTCTCTATCCTTCCTCCTATCAGGGGAGCCAATTTCTGTTTTTTCAGTTTCATTAACTGTCCTCTTGTCGATGCCATCATCATTCTTGTCTTCAGAATCAGTTTTTTTCATGTCTACACCAGACTCTCCATTATTATGTTTGGATGAGATGTCGGCAGATGGTTGAACCAGCGGTGGTGGTGGTGGTGGTGGCGGTGGTCGAGTCTTTAACCACTCTTCAATCATACTATTTATTTGTTCTGCAGCCTCCTTATCGGCCTCAGAATCTTCATTTGTAATAATCCCAAATTTCTTAGTATTTTCCTGATCGTCCTTGTCTCCAACATCTTCTGTTTCATCTGTTTTATCAGAAACAGGTTTCAATGATTCATGTTCAGCAACAGCAGAAGTTCCATCCCCGCCTCCATCTTCCTTTTCTTTAGCATTCTCATGTGCCTTCTTTTTTTCTTCAACATGTTTTTTCAG

>Isogroup_426 GGCAGACTAGGGAGGTAAAGGTTATTTACATGGAGGCTGTTGTTGATAACATCTCAAGTTATCAGAAAGAGGATGAGTTAAGGAATGGAGGAAGTGGAGATCTGGAGGACGATCTTGCTGGAAAGGACAGATACATGGGTTCAATCGAAAGTCTCATCCGCAGCAATATCCAACAGTTCAAGATTGATATGGCAGATGAAGTCATTAATGCTGGTCGTTTTGATCAAAGAACGACCCACGAGGAAAGGCGCATGACTTTGGAGACCCTACTGCATGACGAAGAGAGGTATCAGGAAACTCTTCATGATGTTCCTTCACTACAGGAAGTTAATCGTATGATTGCTCGGAATGAAGATGAAGTCGAGCTCTTTGATCAGATGGATGAAGAATTTGATTGGACAGGAGATATGATGAAACATAACCAGGTTCCAAAGTGGCTCCGCGTCAGCTCTACAGAGCTTGACTGTGTTGTGGCGAGCGTAACCAAAAAGCCTTCCAGAAATGCGTCTGGCAGCAGTGTGCCTGACACCAGTGACAAACTCGAAAAAAGAAGGGGCCGACCGACGGAGGCTGGTAA

>Isogroup_427 TGCCTGTGCAGCACCAAGGATAGTGTTGCCCTGGGTCTTGGACTGGCTAGTGTCCTCAGCTGGGGTGTCGCCGAGGTGCCGCAGATCATAACAAACTACAAACAGAAGTCAACAGAAGGGCTTTCCATTGCTTTTCTAATGACTTGGGTAGTTGGGGATTTGTTTAACCTTGTCGGCTGCTTCCTGGAACCTGCTACTCTGCCGACACAGTTTTACATGGCATTGCTATATACCATCACAACTCTGATTCTCACTGGACAGACAATATACTATAGCCACATCTACTGTCTTAAAGTAAAGAAAACTGGGGCAACTGGCAAGCCTCAGAAGCATCATCGAGGGGATGCTTCATTGCGTGAAAAGCTTTTGGGGCAAAAGGGATGATGCATCCAAAAATAACATCCAGTCAGGTGTGACTATTCCTGTCCCAAGCTCACCAATCCAGGTGAACGCAGAAGTATTCTGGCAGCGCCATGGTGCAATCAGCCCCAGCTCAGACTACTACTATGTGTCTGCAAGATCTCTATCAAGGAGCCCAGTGCCTATTGCAGGTACCTGCCCGGGCGGCCGCT

>Isogroup_428 AGCGTCATTACAGGTGCTTCAGTGCAGAATCTCACAAGTTTGTGACATCCTCTTACTTCTCGCCTACAATACTCTTGTAATTAACCATATAGATGGCTACCAGTGTCAGAAGTGCACCACCAATCTGCTCAGATGAGAAGGTCTCTCCCAGATAGAGGAACCCAAAAATGGAGGCAAACATAGGAGTTAAGAAAGTAAGAGAGCTAAGTGTGGTCAAACTACCTCTTGTAGCATTGTAGAAGTAGACACCATAGCTAATAGCACTGCCGAATATAGAAGTATAACCCAGCGCTAATATGTCACTCCATGTAAGCTCTTGAATATGTCCATTAAGAGCAGGATCATGATTAAGAACAGATATAACCAACAAAGGTATCCCGCCTATTACCATGTGCCATCCTGTTGCCATGATTGGATCAGAATATTTTGATACCCAGCGAACCATGATAGTTCCAATCGCCATGCTTTGAGCTGAGAGGAACATCCACCATTCTCCACTTCCCCAGACTGTTGAGCCATTTTCTTCAAGTGAAAGTGCTGGAACCTCTAGGAGCAATAACCCAACAACACCCA

>Isogroup_429 GGTGTTCCTTTAGACATCACTACCAGGGAACTCCCATTGTTTGCTCCAGCAAGTGCAGATATATAGTAGTTCTTATCCCATTGGTCCATTATCCATTCTTTGTGAAGAAAATGTGGTGAAAGTTCATAAACTTGAGAAGAAAACCCAGTGCCAGCATCCATGATTAGTGCCCAGAGATTTGAACAAGATGATATGCTACTGATAAACAGTCCATCTTCATTCCCTTTGTCAATGTGTGGTTCAAGCCTAGAATCTGCAACATTATAGTGATACCTTTGTTTCATAGACCGCCTTGCATTATAAACACTTATCCACTGCGTTGCAGGCATTCCCATCCTGATCTTCTTCTTTGGTTGTTCATCTGTCTCTTCTTCCGACGATATGCGCCCTCTCTTTTGGCCAACTTGGTATATAAGCTTCTGAGCACCATCCGTGTTAATTGGCCTGACATCTGGATTTGGGCCCACTATACTATCAAATAGGGAGATGCACTTTGCATAGTTGGGTTCTTCTTCAAACTTCAAGTTGACCACATACTCTATAAACCTCCTTGAAGGGCTGAGGGCAGAAGGT

>Isogroup_430 TCAAAAGGCAGGACCCTAATAGCATGCATCTTTTATTCAATATATATTGCATACAGCAAGCAAGCACACTCCTGAGTTTCTGAGATTTCATCTTCCATGCTGATGATGCAGTAGTTTGCCGACCTCTTGTTCATCCTTGGTCACACCTTCACTCAATCCCAAGCCGCTTCTTAGTTTGTTCAAGCAGAAATTGCGGGTCCAGTTTGGTTTGTGTATAAGCATCGATCTTGTTCACCTCCTCAATGAATTTATGAATGTTTGGTCTGCCCTTGGTGATATCATAGTTCTTGATGCCAGAAAAGAATATCTGAAACCTTTCAATAAATGGCACATATGCAATGTCCACCAGACTGAACTGGCCAAGGAAGAAAGGGCCATCTGTGAATTTCCCGAGTGCAGCTTCTATTTTATCCAGCGCAGCAACAGTTTCCTCTGACACATCTCCCTTGGAGATGATAGATGAGTATGCTGCTTTACCGAACTCATCGGTATACGCAAGCAGCTCCTCAGCGAACTGCTTCTTTTCAGAATCATCAGGAAGCAATGCTGGGCCATCGAAGTTGCTGTCGAT

>Isogroup_431 ATTGCTGAGCTTAACCTTCCAAAGACAACAAAAATTTCCTTCCCAAATGGAAAGGATGATCTCATGAACTTTGAAGCTACTCTACGACCTGATGAAGGATACTACTTAGGTGGGGCATTTACTTTCACCTTCCAAGTATCTGCTTCCTACCCTCATGAGGCTCCTAAGGTCAAGTGCAAGACTAAGGTTTATCATCCCAATATTGACCTAGAAGGAAATGTCTGCCTGAACATTCTGCGTGAAGACTGGAAGCCTGTCTTGAATATCAACACCATAATATATGGTTTAAACCCTTCTTTTCTCTCAACCTAATGACGAAGATCCCTTGAACCATGAAGCTGCGGCTGTCCTCCGAGACAACCCACAGAAGTTCCAGAGAAATGTTCAAATGGCAATGTCGGGAGGTTATGTTGACAACACCCATTTCCCGAGGTGCAAGTAATGCTTGCTAGGCGATGCTCTGCGTCCAGCTTATGCCTACCTACTACCCCCTGAATCATGTGGCATACCCAGGTGAAATTGTATCGCAGTTCTGTCAGACGTGACTGTTATTATGCTTGTTTGGTGGAT

>Isogroup_432 TCCTTCTTCACCTCCTCCACCTCCTCGATGACCTGTGAGGCGTCGGTGCAGCCGAACATGGGCAGCTTCCACATTGTCCAGTACCCGCGCACCGCCTCCCTCGGCGGCCGCAGCCGCCTCGGCTTCTCCTCCACCAGGAAGTTATTCCAGGTGAAGGCGTCAAGGGTCGACAGTTTCTCCAAGAGTGACATCATTGTGTCCCCTTCCATTCTTTCTGCAAACTTCGCCAAGCTTGGTGAACAGGTAAAAGCTGTGGAGCTGGCAGGATGTGACTGGATTCATGTTGATGTCATGGACGGACGGTTTGTGCCAAATATCACGATTGGGCCGTTGATTGTTGATGCTTTGCGTCCAGTGACTGATCTCCCATTGGATGTGCATCTGATGATTGTGGAACCTGAGCAGCGAATTCCAGATTTTATCAAGGCAGGTGCAGACATTGTTAGTGTTCACTGTGAGCAAACTGCAACCATCCATCTTCACCGAACAGTCGACCAGATTAAAAGTCTAGGAGCAAAGGCTGGAGTTGTATTGAACCCTGGGACCCCACTCAGTGCGATTGAATAT

>Isogroup_433 ATCGTTTCCTCACCATGGCGTCGGAGAAAGACGCGGCGCTCGCCGCCGTGCCCGACGACAACCCCACCATATTTGACAAGATCGTTAAAAAGGAGATACCTTCCACTGTGGTGTATGAAGATGAGAAGGTTCTCGCTTTCAGAGACATAAGTCCTCAAGCTCCGACCCACATTGTAATCATTCCCAAAGTCAAGGATGGATTAAGTGGCCTTTCGAAGGCAGAAGAGAGGCATGTAGAGATACTTGGCTCCCTCCTCTATGTTGCAAAAGTTGTCGCAAAGCAAGAAGGACTGGAAGATGGCTATCGTATCGTCATCAATGATGGCCCTGGTGGATGCCAATCTGTTTATCACATCCATGTCCATCTGCTTGGAGGCAGGCAGTTGAACTGGCCACCGGGCTAAGGAACTCCCGCTGGCTGATCAGTGCTTCATGGCAAGGATGTTTGTTGCATCTCCGTTGAATAATAATCCTGTAATAACTGTTCGTGTGCTACATCTATGGTAACTACCGGGACCATCCTCCTGTGATGTATGGACTTGATGAGTGCTGTGTTCTTACTGGTACCT

>Isogroup_434 CGTGTGATCGCACTGTCTCTAATTCCGGCAAATGAGCCGTCAGGGCCAGCAGGAAGCCAAGGAGGCTCCCCCAGAAGGAGCATTGGTGATACCCCACAATCCTGGTATTCTGCAGGAATGCTCTCACCAGTAAAGCCAGAATAGGAAATAGCTTGCCTCACAGGGATACAAAGACTGGATTCATCTTTTACTCCTGGATTTGACATCCCAGAGTCGACAGATACTGCATTGCTAGCTGCTGGCTGTGGCTTGGGCTGCTCATCAGAACTCCCATAGGGCACTTCCTTCATTTCAAAGTAACTGTCAATGCCAGCATCATCAAAGAGTTCCTCAGTCTGAATATGGGAGTTACCAAATAGCTCTTCATAATTTTCGTAACTCAGGTCAATATCATCCATGGAGAAATCTTCATATATGCTGTCCTTGCTGAACATATGTTTGTCGGGTGTATACTTAGCAGTCGTCGAATCCACAGATACGGCTGCCTGATCATTAGCATGTGGAAGAGGTTTCAGGCCAGCTTCAGAAGATGAGCCCATCACAGACTTTTGCGTGTCGTCATTATTAA

>Isogroup_435 TCGTGGCGAGCACAATATGCTACTTCAGCTTCTAAGAATGGGTATCCATGAGCAAGTCGCTTACCCAAGCCTTCGTTCTGAGCAATTGTAGCCACTCGTTCAGCCAAAGTTCCATATGCATGTGACAGATGTTTCGATACAGCACTATCCATTGCACCTGGAATAACTTTGCCACCATATGTCTTTTTCATTCGCTTGTAATTCTGAGCAAGAACAGTAAAAGAAGCAGGATCCCATCCATATCCACCAACTATATGCAAATGATCGGTCACACAGCCATTTGCTGGTTGCAAATTCCCTGACCTTATTGCTGCATTAACAGCATCTTCAGCCATGCTTCTATAGGTTGTCCATTTTCCACCTGTGATAGTTATAAGCCCTGGGTAGTCTTCAAATACAACATGATCTCTGGAAATACTTTCCGTGTTCTTTGCGGATGGATCCATGGCCAATGGACGAATACCACTCCAGGCAGAAAGAACATCTGAACGCCTCACCTGAACATTAAGGTAATCACATATCGCGTCCAATATGAACTGTATTTCATCCTCATGTGGTTCAGGAAGA

>Isogroup_**436** CAGTTTAGTTCCTGCATTTCCAATGACCACATCACAACCTGAGAACCCAATTCAAGCTCGGGCTGCTCTCGGTTTTCTACTCTCTGATAGGGGTGACTTCTTCCGGGAGTTCATTCTTGACGAGGTTGTGAAAGCTATCGACGCAGTTTCAAGGGAGCAATTGCTACAAGTTGCTGGGTCTTTTGGGATCAGAAATCCCACCCCAGTTTTTGGTATGGTTCCTGTCAGGTCCGGTGCATTGCTTCCTACAATCACAGAGGAAGACAGGGTCATCTTGAACAATGTCGAGAAGGTTGTCAAGTTCTTAACAGCTGGGACAGCAAGTCCAACAGTGAATGGGGATGTAAATGTCATGTCTCTAGTGCAAGAGCTCCTACCTGTTCTGCCGAGCATCTCGTCGAAGATCCTACCAGATATCTTGAGCCGGTTATCATCAAGGGTGTTTGCACGGGTGATCCGGGAAGCATTTTTGTGAAGCACTTTTCTTTTCTTCTGACAGGAAACTAGAGAGTTGTTTCGCCTTTTCATTTTATACATAGAACACACACACACGGTGATAGAATGATG

>Isogroup_437 ATCATCGGCCTCGCGAGGATGTTGCCATCCACCACCACAGGGCGTTGCACAATGGAATGCATCCCAAGAATCGCTGACTGCGGGGGGTTGATGATAGGGGTGCTAATAAGGCTTCCATAGACACCACCATTGGAGATGGTGAATGTTCCTCCTGCCATCTCATCAATTGACAGAGCTCCCTCAGTAGCCTTCTTTGCAAGGTTGTTTATCCCTTTCTCGATGTCAGCAAAGTTCATTGTATCAGCATCACGAATAACAGGCACCACAAGACCCTTGGAAGTGCCAACAGCAACACTAACGTCAACATAGTCTCTGTATATGATGTCATCGCCGTCAATGACAGCATTCACGATTGGCTGGTTCTGCAGTCCAGAAACAGCCGCCTTTACAAAGCAAGACATCAGACCCAACTTCACACCATGCTTCTTGACAAATTCATCTTTATAATCAGACCGTAGCTTCATCAAATTGGTCATGTCAACTTCATTGAATGTGGTCAGCATTGCAAATGTGTTCTGGGAATCCTTCAAACGGTTGGCAATGCGCTTCCTGAGCCTTGGCATTG

>Isogroup_438 CCAACAGCTGAAGCCAGTTCTACTACAAATGCGTGGGCACCGTCGCATCAGTTCTTCCCCTCGGTGAGCCTGATTCTTTGTCTTTCTTAAAGGTCTTCTTCAGGAAGAACGCCATCGCAAGCTGAGCGCCAAGGTTTGATCGGCCTTTAGACAGACAAACGATGCTTCCAATAAGCAGAAGTCCACTTGCAATCGTTTCCCGTGTTGGCTTCTTACTTAGCTGCTTTCGGGGTGAAGTTCCGGACGAATCTGTAGATGGAACAACAGCTGCAGCTGTATTCACTGGAGATCGACAACTTTCATCCACGTTGGTCTGCAAGGCCTGGGGAAACTTCTTGATAAGTCTTTGCATAGTCAAAATTGATACTGACGGACCATGGTGCTGAGGATACCGGCGTAAAGCATTGTTCATTTTGCAAATATATTTCCAGATTCCCTTGGAAAAGGACAGCTTTGCCATCTCGATGTTCATGCCATTGTCCTCATGATGCAGCACTGCGATTTGGCATGCATCTCTTCTAGGGATTTTCCTAATACACCATCCTGATCTCAGAAGTCGTAC

>Isogroup_439 ATAAGGCATGAAAATGTTCAATTCTTGGAGAAGCTTAAGCAATTGGTTCTATTTATTGAGAGGAAACTCGAGAAGAAGGATTTCATTCAATTAAGTTTCTACTCTGAACCTGATGGTCCTATAGTTGGAAATGCCACCTTTAAGTCCTCTATTTTAGTTCCTGGAGAACCTGAAGCATTCTATGTGGGTCCGCCATCTACAGAAAAACTTCCAAAGAGTTCTCCACCTGGTTCTGTTTTAGTGGGCTCCATAACCTATGGTGTAGTAAGCTCGTTCAACAAGAAAGAAGAACAAAACCAGCATGCTCCAGCTTCTTATAGTATTTTATGTGTCATTCCACCATCAAAGGTCGATGATAATAAAGAAAAGGGGGTTTCAGTTGGAACAGAGAAGAGTGTTTCTGAGCGGTTAAATGACGAGGTTCGGGACACCAAGATAAAGTTTCTTTCCAGCCTTAAGAAAGAAAAGGAAGACAACAAGTCTGCCTGGACTGAGCTTGTCGCTTCTCTCAAGTCCGAGTATCCAAAATATACACCTTTGTTTGCCAAGATTTTGGAATGT

>Isogroup_440 CAGGGTCGTCGAATCCCCAACGTTCGGTGGCCGTAAGGGATTTTGAAGAGCGTCGAAGAGGAAAATCACAATTTTCAGATAATCAAGTTGCAGGACAGAAAAAACCACTCTATTCCAGCAAAGGGCTGTCTAAAGCTTGCGAGTTTGTCTACAATGATGCGAAGTTTGTGAATGAAAGAGCTCAAAGTGATATTATTTTGCTTTCACGTGGCATCACAAGGTTGAACAAACGTGCATCTCAGGATGTTGCTGTGTTAGGGTTAGGGTTTCTCAAGCTTGATGCTCGTGCAAGGAAGGACACCCAAAAGATTGACAACAGTGTGAAGGAGCGGGCAGCCCACCTGAGTAATTTTGCTAGAATACTTAAGGAGCGGGCTCAGTCAGACTTGAAGAAAGCAGCGGACCAACATTGGAGTGATGGTGCTTTAGAGGCAGATCTGCGACGAGCTGACATGGTTGTTCGACGACGTGCCATGGAGGACGCTTTCATGGCTTTAAAGTTTGTTCGGGATATCCATGACATGATGGCAAAGAAATTACAAGATCAGCTTCCAACCCATG

>Isogroup_441 AACACCATTGCAAGAGTGACAGATGGTATTTATGAAGGAATCGCAATTGGAGGAGATGTTTTCCCTGGTTCAACTCTTTCAGATCACATTCTTCGTTTTAATAACATACCTCAGGTTAAAATGATGGTTGTTCTTGGGGAGCTTGGAGGAAGCGATGAGTATTCACTTGTTGAAGCATTGAAACAAGGAAAGGTTCAGAAACCTGTAGTTGCTTGGGTTAGCGGGACATGTGCACGTTTATTCAAATCTGAGGTCCAGTTTGGCCATGCTGGTGCAAAGAGCGGTGGTGAGTTGGAATCAGCACAAGGTAAGAATCAGGCACTAAGGGATGCTGGGGCAGTTGTCCCTACTTCATTTGAAGCTCTCGAAAGTGTGATTAAGGAGACATTTGAGAAGCTGGTTGAGGAAGGAAAAGTTCCTATTGTCCCTGAGATTACACCCCCTCCCATTCCCGAGGATCTTAAAACTGCAATTAAAAGTGGGAAGGTCCGAGCTCCCACGCACATTATCTCCACTATCTCTGATGATAGAGGTGAGGAACCTTGCTATGCTGGT

>Isogroup_442 AGAATTGGCAATGTTTTCAGTCAAGAAACATACATGATCTTGTCCAACAATTTAACATTTTCTGTCAACAAAGGAATGCAATGTCTGATCAACATAGCAACTGGCCTTTTCGGCACCATCGACTGCTCTTGAATAGTTCCCCGTGGAGGAACCCCTTCAAAATTACAGGCAGCATCAGTAAAGGCTTGAATTTACCTTTTGCGCACGTAGCTGTTGTATCCTCTTCTTGTCCTGCTCAAACTTGCTCTGCAGCATCGCCAGTTCGGTCATGTGAGCTTCTCGTTTCTGATGTCTATAGAAGTTCAGACCAACTTCCTTAGGTTCTTTTGAGCCATTTTCTCTTGCATTGCAACCAGAGATACAGAGCCAACTGCTGTTCCAGTTTCGGCATCTGTAGTCTTTTTCCTACCCTTATGATGCACCACAACTGTCCACCCATCTTCCGCAGCTGCTGCCTCTCGTTCCTTCTTCTCCTGCTCTTGTCGTTCCTCGTGCGCAGTTATAAACTCATCTATCTTTTGCTGCAGAACTTTCAAGCCTGGTCTCTTCTGTTT

>Isogroup_443 ATTGACGTGACACCAGATGTAGACACCCGTATTGAGCTTATCAAAACATTGAGCAGTGTTGCTGCTGGAAAAATATATGTGGAGATAGAGAGAGCTAGATTGATCAAAAGACTTGCGAAAATCAAAGAGGAGCAGGGAAAGATCGACGAGGCAGCTGATTTGATGCAGGAAGTTGCTGTTGAAACATTTGGCTCAATGGCAAAGACAGAGAAAATTGCATTCATTCTGGAGCAGGTTCGGTTGTGCCTGGATCGTCAAGATTATGTTCGCGCACAAATTTTATCCAGGAAAATTAGCCCTAGGGTCTTCGATGCAGACACAACAAAGGAAAAAAGAAACCAAAAGAAGGTGACAATATGGTTCAGGAAGCTCCTGCAGAGATACCTTCACTCTTAGAACTGAAGCGCATCTACTATGAACTGATGATACGCTATTACTCACATAACAATGATTACCTGGAAATATGCCGTTGCTACAAGTCGATTTATGAAATTCCAGCAATAAAAGAGGATCTGGCAAAGTGGATACCGGTTCTTAGGAAGATCTGTTG

>Isogroup_444 TTGGTATATGTCTTGGAATGCAGATGGCTGTCGTAGAGTATGCTAGGAATGTCACGAATCTCTCTGATGTGAACAGCACTGAATTTGATCCCAATGCCAAGACCCCATGTGTTAGTTTTTATGCCGGAGGGTTCCAAAACACATATGGGTGGCACAATGCGCCTAGGATCAAGAAGGACATTCTTCAAGGTTGCTGATTGCAAGTCTGCTAAATTGTATGGTAATGTGGATTATGTAGATGAGAGGCACCGGCACCGCTACGAGGTAAATCCTGATATGGTGCCTGAGTTTGAAAATGTTGGACTTAAATTTGTTGGCAAAGATGAGAGTGGCAGGAGAATGGAGATAATCGAAATACCGAATCATCAATATTTTGTTGGTGCACAATTTCATCCCGAATTCAAGTCAAGACCATCGAAACCTTCTCCTTTATTTGTTGGGCTAATAGCTGCCTCATCTGGGCAACTAGGCAAGGTTCTGCAGGACTGCTCGAGTGACCATGTGATTCCTGCTAAACATCAGTTGAACAATGGTCCATACATGCCAGCT

>Isogroup_445 CCTGACGCCTATGTCCGCATCATTGGCTTTCGACAACATCCGTCAGGTGCAGTGCGTCAGCTTCATCGCCTTCAAGCCACCAGGCTGCGAGGAGTCCGGCAAGGCCTAAGTTGTCAGCTGAAACAACGGTCACTATATACTTTTGCCGACGAACTCTGCTCCAGGGTTTGTTTTGCAATCAATATGATTATGTGCGTATATATGCCTGTATTTGTATGTATGTACCTGCCCGGGCGGCCGCTCGAGAGCGTGGTCGCGGCCGAGTACAGTCCTATTCTGATATGTAGGAGTAGCTGGCGCTGTTGTAGTTGCTGGTCTCCGGTTATACACGCCCGTCTGCTGCGGTGTGGGGCTCTTGTTCTGATACCTTGCTGAAGAGCCATTGCTATACGCTGAAGACGGGGTGTGACTAGCTGTAGTTGTTCTTTGCTGTGCATTAGTATAAGCAGAACCATCATACCATGCATAGTGTGCTCCTGTGGTGTCATACCCATAGGTTGAGTCCACATAACCAGTAGAGGGAACTCCACCTAGCACAGGTGCCCTAT

>Isogroup_446 GCTCGACTGTCTAGGTGCTTGATGAGTTTCATGGGAAGGAGGCTCCTCTTTCACAGGACCACGCACACTTACAGAGCTGCCTTCGGTATCATTACCTTGAGATGGTGGTGGCCCAACACGGTTCTGCTCATAGAGCTCCAACATCTGGTTGCTAACCTCTTCCAACTGACGTGGGGTTACATCAAACTCTTGCCACCAGACCTTCTCTCCATCTGCTGGAAGCTTGACTTTGAGGAACTTTGCAGCAAGGAAGATTGCGCCAGCCGCAATATGATGGGGCTTAAATTGCAGGCAAAGCGAGGTGCGTAGCCCATCGTTAACAAAATTCCAGGCAACTTGAGCAAGTGCATTTTGAGCAACCTTGAATTTTTTAATTGCTTCAACCAAGGGCTTGTAAGGGTGGTGCACATTCATGTCAAAACCAAGTGTAACAAGCACAAGGCGCTCTCCAATCAACAGAAGTTCCTTCTGTTGCTCATACACTTCCTTCTGCTTAATTCGAGCAACTGCAGCAGGATCTTTTTTGTGGATGATCTCGTAAGAGATGA

>Isogroup_447 CATCCCTCTGAGGCAGCTACCAAGGGTCCAAAGTGGGCAGAACTATTTGAACCCGGAGTGAAGCATGCACTTTTTGTTGGCATAGGATTACAGATCCTGCAACAGTTTGCTGGTATCAACGGAGTTCTCTACTACACCCCTCAGATACTTGAGCAAGCAGGTGTCGGGATTCTTCTGTCAAACCTTGGACTAAGCTCTTTATCTGCATCTATTCTTATTAGTGCCTTGACAACCTTGCTGATGCTTCCTAGCATTGGTATTGCCATGAGGCTCATGGACATGTCAGGAAGAAGGTTTCTTCTCCTTTCGACAATCCCTGTCTTGATAGTAGCACTGGCTATCTTGGTTTTGGTCAACATTCTGGATGTCGGAACCATGGTGCATGCGGCACTCTCAACGATCAGCGTGATAGTCTATTTCTGCTTCTTCGTCATGGGGTTTGGGCCTATCCCGAACATTCTCTGCGCAGAGATTTTCCCCACCTCCGTCCGCGGCATCTGCATAGCCATCTGCGCCCTAACCTTCTGGATCGGTGACATCATCGTGACG

>Isogroup_448 AAGGTTATTCAGTTACTAGATGGAAAATGCGAAGGAGATGCAACAATGAAGGACTATCGGTTAGTGAGAAACTGTATCTCTGGGCAAATCTATGATTCTGGTCCAGTGGATTTTGTGAGCTATGTGGGCACCCAGTTTCTCCAAAATCCTGTGATTGGTATGTCGTCTCAACCATCCATGATACGCTCCTTCATGGCAAAGGCTCTTGCATCTGGAATGGACACTCTCTTTCCAAGCAGAATTGAAGCTCAGCGCGCAGAGTATTGGCACACGCTGTATTCATCGGCAGGGTTGGGTTCAGTGCTTATATTCTGCTCAGAAGATGATGAGCTTGCTCCATGCCATGTTGTCTGTGGTTTCGCTAGGCGTTTGGTCGAACTAGGCACAGATGTTAAAATAATCAAGTGGAGCGACTCCCCTCATATCGGCCATTACAAGTTACATGAAGCGGAATACAGAAGTGCTGTAGATGATATACTGAAGAAAGCACTTGTCACCTTCTGCCACAGAAGCCAGCTGAACCGCACAAGTGCTGCAGGTGATCAGGA

>Isogroup_449 AACGCGTGCTACTTTGCTAGTGGAAGAATGTCCCTCTTGTCTATGTAATCCTACCATTCTATTCATCAATATCCTTTACAGCGTCCTTCGCAAGCCTTGTTACGTAAGTAGCAGCAATGGCTGTAACTAATAGTCCTATGCCCAACGTCAATAACTGGCTGTTTCCTCCCAAACTAATTTCCGTCTCATCTTGAATTATAGCTCGCCCAAATGCACCAGCACTGACATAAGCCCATGTTCCTGGAAGCATTCCCAACCAACTGCCCACCACGTAGGGCAAGAATTTGACCGAAGTCAAGCCATACAGGTAGTTCCCAAGGGAGAAAGGTAATAGGGGACTCAACCGAAGCAGAGTCACAACCTTGAATCCATTTTCACCTATTGCTTTATCAATCGCCAGAAACTTCTTATTTCCTTCAACCATTTTAAGAATACGCTCTCTAGCAAAGTATCTAGCGATGAGAAAGGCCACTGCAGCAGCTAGTGTCCCACTGATTGAAACCATAATAGTGCCAGTAACACTACCGAATAATAGACCTGCAGACATA

>Isogroup_450 TTCTTCCATTGTTGGAGGGCCAATAAACTCAAATCTGCATCCAACATCACGTTCAAGTGATTTAACTGTCCTTCGCTGATTGCTTGTAAACATTAAGATTGCATTTCCTGCTTTCCCAGCACGCCCAGTGCGTCCAGAACGATGAACAAATGTCTCTGGATCATTTGGCAATTCATAATGGATAATCAAATCAACATTTGGTATATCAAGACCACGAGAAGCAACATCAGTTGCAACAAGAACAGTAAATTTCCCTTGGCGAAAACCATTTAACGTCCTCTCACGCTGATGTTGTGAAATATCACCGTGAAGTGCCTCAGAAGCAATACTGCTTGTCAATGACAATGATACCTCGTCTGCATCGCGTTTCGTCCGAGTGAAAACAATAGTTTTCCCACCCTTTGCATATACCGTAATTAGATCACTAAGAATGGTGCGCTTTGATGTCGCCGTGAGTGGGATAGCGTAGAGTTTGATTCCTTCAGCTAGTTTTTCATCTTGATCGCCAACCAAATCAATCGTCAAAGGGTTGTTCAAGTACCTG

>Isogroup_451 AGAGCCTGTTGTTTCATGACCAGCAACTAACATTGACAATAGATCATCCCGTAACTGCAAACTGTTGACCTCTTCACGGCTAGCAAGTAAGAACCGCAGGATGCTAGGATCTGCCTCATTTACATATTCGTCGCCGTCAATCTGTTCATTTTCAGCATCTACAATAGCCTTGCACTTTGTAATTAGCTCTTCAACAGTATTCCTTATTGTGCTAACTGCCTTTTCTGCTTTTATCTGTCTAGGAACAATCTTGCACAGCAAATCAATCTGCCAATATGGTAAAAGATCTGTTGAACGAGCCTCTGCTTCTTTGAGTGCGGTGTAAACAGCATCAATAACAGGACTATCTGATGTGAGGGAGTCAAAGTTGTAGTTGAACAAGGACAAACCAATCACATCTAGAGTCATTTGAGAAAACCTCGCTTCCATATTAACAGGTTCACCACTCAAAGCATATGTCTCAAGCTTATCCACTAATCTCTCAGCACATTTACAAAATACTTTATCAACCATCACTGAGAGAAATCTTTTGTGTAGAGATG

>Isogroup_452 CCTCCCTCTGTGGTAGGTTCGTCTTCACGTCCATCCACGCCCACCTCTTCCAGTGCTAGTCCACATCAAAGAGCTTCAGATCATCCTCAGGCTTCGTCATGTGGCCGGCCATCTCCAGCTGAGGCTGTAGGGATTATTGCTCGTTTGAAGGATAAAAGTGTTGATGAATTGCAAAGGCTGCTAAAAGACAAAGAGGCATATAATGCTTTTTTCAGTTCACTCGATCAAGTGAAAACCCAAAACAATCTACGGGGCGAGCTTAGAAGGGAGACACTGCAGCTTGCGAGAGAAAATTTGGAAAGGGAGCAACGGATTTCGGAGCTCAGGAATCAGTGCACTATAATAAGAAGTGCAGAACTGGCTGCTGCTGAGGACAGGTTGGCCGACTTGGAAAGGCAGAAGGACGAGATTATGAGGTCCTATTCTCCTGCTGCACTACTCGACAAACTGCGAAGTTCAATGGCCAAGTTGGACGAGGAGTCGGAGGAGCTGCATCAGAAGTTACTGGAGAAGGACATGGACCTGCCCACCTTCGTGCAAAA

>Isogroup_453 CCATGCAGGAGCTAATGCCATCCATGATGAGTGCCGTAAGAGAAAGCTCAAAGTTTCAGTTGTGGCAGTTCCAAAGACCATTGACAATGATATACCTTTAATGGATAAGACATTTGGTTTTGATACAGCTGTTGAAGAAGCTCAACGGGCCATTAACTCCGCCTATATAGAGGCACGTAGCGCATACCATGGTATCGGTTTGGTCAAATTGATGGGAAGAAGCAGCGGCTTCATTGCAATGCATGCTTCCCTTTCAAGTGGGCAGGTTGATGTCTGCTTAATACCAGAGGTCCCGTTCACACTAGACGGAGAATATGGTGTCTTACAGCACCTTGAGCATTTATTGAAGACCAAGGGATTCTGTGTGGTTTGTGTTGCTGAAGCTGCAGGACAAGAATTATTGCAAAACTCAGGTGCAACTGATGCATCAGGAAATGTAATACTTAGCGACATTGGTGTTCACATGCAACAAAAGATTAAGATGCATTTCAAGGGCATTGGTGTTCACGCTGATGTAAAATACATTGATCCGACATACATG

>Isogroup_454 AGCGGCCGCCCGGCGAGTACAGCTTGGTCGAGTCGATGATGCAAAGGCAATTGCTCTTGAGGTGCAAAGCGAGTCTAAGTGGAAGCAGTTAGGGGAGCTAGCTATCTCTACTGGAAAGCTCGAGATGGCAGAGGAGTGTCTTCTACATGCCATGGATCTCAGTGGTCTATTGCTTTTGTATTCATCCATCGGGGATGCTGAAGGGATAACAAAATTGGCGTCTATGGCCAAAGAACAAGGAAAGAACAATGTTGCCTTCCTTTGTTTGTTCATGCTAGGCAAGTTGGAGGAATGCCTTCAATTGTTGATTGAAAGCAATCGTATTCCGGGAGCAGCATTGATGGCGAGATCTTATCTTCCAAGCAAAGTCCCTGAGATTGTAGCATTATGGAAAAAAACGTCTCCAAAAAAGTCACTCAAAAGCAGCAGAGTCCTTGGCTGATCCTGATGAGTATCCAAATTTATTGAAGCTGGCAGATTGCTCTCAATGTTGAAGCTGCCGTTGCTCCCAAAAGGGGTTCTATCCACCTGCTGA

>Isogroup_455 AGTCCTGGAGCTCAAACCAGCCATACTGATCCTCCCATCAGATGTCATGTAAATGTGGAATTCCTGCCTCATGAAGGCTACTTGATCACTGTTGAGCCCAGTGAAAGTAAACATCCCAATCTGCTTAATGATGTGGCTCCAATCTCCAGGAGTGCCTCTGCTTGTCAGCGCATCAAAAAAGCTGTTGCCTCATGCTAATGATCCTATCAGCCATGGCCTTCAGCTCCATAGTCCATTCGTCGAACATACCACTAGTAAATTGAGGAAACATCATCAATAACAAGAACTAAGCACTACCTACTGTTCCCTAGATTTGTCGATCCGCCAATAAAGTTCATCTCAACATAACTCACTTTCTACAATTAACTAGAATTAGGGCAAGAATATTACCTGTCCTTAAGTATGGTAGCCACGATAGAGGCACCATGAAGAGGAGGGTTTGAATACATAGGCCTAATTACAAGCTTGAGTTGACTTTCAACCTTACCAGCTACATCAGCACTCCCGCAAACAATGCTTAAAGCACCGACACGCTCTCT

>Isogroup_456 TAAATATCGTGGAGAGTTTGAAGAAAGGCTGAAGAAGCTTATGGAGGAAATTAAGCAAAGTGATGATATTATTCTCTTCATTGATGAAGTGCACACTTTGATTGGGGCTGGTGCAGCTGAGGGTGCGATTGATGCAGCCAACATCTTGAAACCTGCTCTTGCAAGAGGTGAACTGCAGTGTATTGGTGCCACAACGCTAGATGAGTATAGAAAGCATATTGAGAAAGATCCAGCACTGGAAAGAAGATTTCAGCCAGTCAAGGTTCCAGAACCAACTGTTGATGAAACCATACAAATTTTGAGAGGTCTTCGTGAGAGATATGAGCTTCACCATAAGCTGCAATACACGGATGATGCTCTTATTGCTGCTGCGAAGTTGTCGTATCAGTATATCAGTGATCGTTTCCTGCCTGACAAAGCTATCGACCTGATTGATGAAGCTGGATCACGTGTCAGGCTTAGGCATGCTCAGCTCCCTGATGAAGCCAAAGAATTGGACAAAGAACTCCGCCAAGTTACCAAGGATAAGAATGAGGCA

>Isogroup_457 TCCGTTTTCTCAACCATTCCAACCTTATGTATGTCATTAAGTAGCTCTCCTTTGTCATATGGAACAAAAAGCTTCTATGGGCACCAGCGAGTCTTTCAACTTTGCTTGGATTGCATTGCAGAACTCCTCCAGACCATCACCATTCATGGCTGATATGCAGATAACCCCTTGTTTCTCAGCTTCCTCTTTCACTCTCAACGGGTCATCAGTATTGTCAATCTTATTCCACACAACCAACTTTGTAATCGACTCTATATCCAACTCTTTCAGCACTTTGTCAACAGCTTCTATCTGTTGCTGAGCTAATGGATGGCTGATATCTACAAGATGAACTATAATTGATGATTCTGATATCTCCTCCAGTGTTGCTCTAAATGCTGCTACCAGCATAGTGGGTAGCTTCTGGATGAACCCGACAGTATCTGTTAGGAGGAACTCAGTGCCGTTCTTCATCAAAACCCTCCGTGTTGTTGGATCTAATGTGGCAAATAATTTATCCTCAGCAAGCACATCAGCTCCAGTTAAGCGGTTCAAGAGT

>Isogroup_458 TTGAGAGTTATTGGGAAGTTCGTCTTCTTCTTGAAATGATCCTTGATCTTCTGCGATAGCCAAAGGCCAACATCCAGAAGCAGCTTATTTCCAGAAGCATCCTGGGTGTCCACAAAATTCATGCTTTGGGCGATGAGATCTTGCCCAGCACCCTCGGCCACCACAATGACCATATGACCACTGTCCTTGAGACGTTTCTGAATGAACTCAAGGAGGCCTCCTTTTCCTTCAAGGTAGAATGGAGACTCTGGGATTAAGCAACAGTCCACATCACGACTAGCTAGCGTAGCATACATTGCGATAAAACCACTGTTGCGACCCATTAACTTCACAACACCAATGCCATTCTCTGCACTTTCAGCTTCGACATGCGCAGCGTTGATGGCTCTTTGAGCCTCCTCCACAGCAGTGTCAAATCCAAAAGATTTGTCAATCACCGCAATATCATTATCTATGGTCTTTGGGATACCGACCACTGAACATTTGAGGCCTCGTCTACGGACTTCCTCATAAATCACGGAAGCGCCTTGCTGAGTA

>Isogroup_**459** TGGCTTTGGATCTTTCCACAGGCTGAGACAAAATGCTTAGGGGCAGCTAAGTATCCAAGTCGCCAACCAGTCATAGCAAAAGCCTTAGAAAACCCATTTACAGTTAATGTTCTGTCCCACATTCCAGGTAATGCAGCAAAACTTGTGTGTTTAGCAGGATGATAGATAATATGCTCATAGATCTCATCAGACAGAACCAGGAGCCTAGGATACTTCTTGACTATAGCAGCAATCTCCTCAAGCAGCTCCCTAGGATACACAGAGCCTGTTGGATTAGATGGAGAGCATAGAATCAAGAGCCTTGATTTTTCAGTAATCACTGAGGCAAGTGAATCTGGCTTTAGCAGGAAATTGTCTGATATGCTTGTCGGAAGGATAACTGGTGTTGCACCAGCCAACCTAGCCATTTCAGGATAGCTGACCCAAAATGGTGCTGGTATCAAAACCTCGTCACCAGGCGAGCAAACAGCAAGAACAGCTTGTGTAATGCACTGCTTAGCTCCATTGCTCACTAGCACCTGGTCTGGGCT

>Isogroup_460 GGAACACTCTGCATACGTGAGAATCCTGGGTGCCTTTTCATTGCAACAAACAGGGACGCTGTTACCCATCTAACTGATGCCCAAGAGTGGGCAGGTGGCGGGTCGATGGTCGGCGCTGTTCTTGGTTCAACCAAACAAGAACCACTCGTCGTTGGAAAGCCGTCGACATTCATGATGGACTACCTGGCAAAGAAGTTTGGAATCACAACATCTCAGATATGCATGGTAGGTGACCGTTTGGATACTGATATTCTGTTTGGGCAAAATGGAGGCTGCAAAACTCTTTTGGTTCTCTCAGGTGTGACTTCTGTGGAGACGCTACAGAGCCCCAACAACACGATCCAGCCAGATTTCTACACCAACCAAATTTCTGATTTTCTCACCCTCAAAGCAGCAGCAGTATGAACAAATATGTGAACAACTGTTTTAGCTGGGGGAGGGCCCTTACTTTTGTATAGCAACTAAGAGATAACAAGGATTGTTTTTCTCTTTCACGTCGATTTGTAACCGTGAGACCAGATGTTTATATGCAAGTAT

>Isogroup_461 AAGAAAAAAGCTGCAAGATATTGACCCAGTTAATAAGTGCGAGACCTAAGCTTCAGAATGGCATGGTTCCAAATGGAGAGGCTTCAAATTCAAAGAGCAAGCTTACATCAATTCATGATGTGTTGAAGGGTTTGGTTGATTGGCTCTGTTCTCAGCTGAGGAGCCCTACCCACCCGAACTGTTCTATTCCTACTGCTACACATTGTCTTGCCACTTTGTTAAAGGAGACATATGTTAGAACTTTATTTGTTCAGGCAGATGGTGTAAAACTGCTCATTCCTTTAATATCTCCAGCTTCAACACAACAGTCAATTCAGCTCCTCTATGAAACTTGCCTTTGTATCTGGCTTTTATCCTTCTATGATGCAGCCGTTGATTATTTGTCCACTACAAGGGTTATGCCAAGACTTGTAGAAGTTGTCAAAGGATCTACTAAAGAGAAGGTTGTCAGAGTTGTTGTGATGTCCTTCCGTAATTTGTTGGCGAAGGGCGCATTCGCTGCCCAAATGATTGATCTTGGGTTGCCGCATATA

>Isogroup_462 CTTTATGAGATGTTCAAGGTCCGAAAAACGCTCCTGCACATTTGGCAGCAAGAATGGCCGCCTATGCAGGGATACTCGAAGCAAACTTAGTTTTGTGAATGAAGGACAGCTGCTACTAGTATCTGAAGACAGTGTCTCTGATCTCAACAGGCGGCTTAGTTCAAGTAATAATGGAAATGGCAAGCAACGGGTTGTTGTTGATGCAATGAGATTTCGCCCCAACATTGTTATCTCTGGGTCCACGCCATACGACGAGGACAACTGGAGAAGACTTCACATTGGAGATGCCAATTTTACTTCCATGGGAGGATGCAACCGTTGCCAGATGATCAACCTACAGCAGAACTCAGGACAGGTGGTCAGGTCGAAGGAACCGTTGGCAACTTTAGCATCTTACAGACGAGAAAAGGGCAAGATTTTGTTTGGTGTCCTGTTAAACTACGAAGACGGCTTGGATGGGGAAGAGGAGATCATCGCAGAGAGATGGCTCCAAGTAGGGCAAGAAGTGCACCCTTCGACAGAGTGATTTTGT

>Isogroup_463 TTCTCAACAATCTTTTGAGTATCATCATGAGTGTTGAAAGAGTTCATTAGAAGCAAAGGGACATCACATCCGTACTCCTTTTAAGGCCCCATTTTCGCTTTAACTTTACATTTTTGTTGATTACTGTAACTGCTTTGGATAAATGTGTATATACATTCCTGTTTACAGCAGACAAATGCATTAAGATCACATACTGAAACCTAAAAGACAATTGCAGAACTTATCCTTCAACGATCTTCACAGGACTCCCAAATGGACTCTGTTTGATTGATAGAAGTTCAACATCCAGCTCGATAATTGCATTAGGAGGAATTTCTTGAACGCCTTTTTTCCCATAAGCGAGCTCAGGAGGAACTATGATCAACCTCTGACCTCCAACTTTCATTCCCTCAACCCCTAGATCCAATCCTTTGAGAACATTGCCTCTTTCGGAATTGCCAACATCAAACCCATATGGCGATCCACCAGTGACACCAAGACCCTGCCTGCTTGTCATGAATGTTATGCCCTTCCACTTGGCCACGTAAT

>Isogroup_**464** TTCTCCATCCCGATTTTCAGCCAAGGCTTCATATTTCCGTTGTAATGCAATACTGCGCCTTCCTTAATAGTAGCAGGATCAACAGTTGTATAGCCTAACCCCAATACATGCCATTTTGAGTCCAATGCTTCAACCAAGCCATAGAAAGCAAGAAGCCCAGGTGGTAGAGACCCAAGTTTCCATAGGGTATGATCAGCATTGCGCTCTTGCCAATAATGATATATACCTGTCACATTCTTGTTCCTCCATTCAACAAGATCAAGCACATTCATACCAAATGCCCAGCCACAAGCATCAGGATCAAAATGAGCACGAATTAGCGGATGAGAATGATTGAGATACTTATGAAATCTGTGGAACGTCTCCATGCAAGTCTCAACTGCCCCCATAACATTGCCGTTGAGGTTGATTGTAAACAAATCTGATAGATCCTTCTGAACAACAATGTCATCATCAAGAAATACCACCTTGCGGAGTTCTGGATATATTTCTGGGATGTAAAACCTCAGGTGGTTGAGCATTGATAA

>Isogroup_465 GACGCCGGCAAGGGGACCTACTTCGCGTCGTCGTCGACCCTCTTCGTCATCGAGTTCATCCTCTTCCACTACGTCGAGATCCGGCGGTGGCAGGACATCAAGAACCCTGGGTGCGTCAACCAGGACCCCATCTTCAAGAGCTACAGCCTGCCTCCTCACGAGTGCGGCTACCCCGGCAGCGTCTTCAACCCGCTCAACTTCGCGCCCACCCTCGAGGCCAAGGAGAAGGAGCTCGCCAACGGGAGACTGGCGATGCTGGCCTTCCTGGGGTTCTCGGTGCAGCACAGCGTCACCGGCAAGGGGCCACTCGAGAACCTGTTCCAGCACTTGTCTGACCCGTGGCACAACACCATCATCCAGACCTTCTCCGGCCAATAAGTCGTATGACCTGGAAGGCTGCAGGTTGATGTAAAATTGATGGATGTCACATGATATGTAAAAAATATGATCGATGCATTTTGTGTTGTAAACTAGTAGCGCTATGCACGCACACGGCTGCATGGTATGGAGTGGATGATTTGTGT

>Isogroup_466 TGCGGGAACGTGGAGCTGTGCAGGCTCACGGAGCAGGTTGTGTTCTCGGACCCATACATGGTCTCCAAATTTAACCACTGGACCTCGCCGTTTCTCGACAAAGATGCCGAAGCGGTTCGAGAGGATGATGGGCTCAAGTTGGAAATTGCTGAATTGAAGTCGATGTTCATCGAGAGAGCGCAGGCTCTGATTCATGGAGATCTCCATACTGGTTCCATCATGGTGACCACAGACTCCACTCAAGTGATTGATCCGGAGTTTGCTTTCTACGGACCGATGGGTTACGACATTGGAGCCTTCTTGGGGAACCTGATTCTGGCATACTTTGCACAGGATGGGCATGCTGATCAAGCAAATGACCGTAAAGATTACAAGGAGTGGATATTGAAGACCATCGAGGAGTCATGGAATTTGTTCCAACAGAAATTTGTTGGACTGTGGAACAAACACAAAGATGGGAATGGGAGGCATACCTGCCCGCCATATACAACAACCCAGAGCTTCTGAGCATTGCACAGAAGAA

>Isogroup_467 ATCTGGGCCAAGCTTGTAAGCAATGGTGCTAAGAAGACCATGGGAGGACTGGGTAACCTCCTCCCCTGTGTTGAGAAGGATGAAGGCACCAGTTCCATAGGTGCTTTTTGCTTCACCTTTCTGGCATAGCTGCCCAAGCATTGCAGCATGCTGATCACCTAGACAACCAGAGATGGAGACGCCAGCCAAAGGGAATCCACTGGCAACCACACCAATTTTCTCTGAGTTACTGATAATCTTCGGCAAGATGCTGGCAGGAATGCCTAATGCCTCAAGTATAGTCTTGTCCCAGTCAAGTGCCTTGAGATTCATAAGCATCGTGCGAGATGCATTTGAGCAGTCTGTGACATGTTGCCCGAGTAATTCTTTTCCATCAGCGTCTTTCCCACCAATGCCTCCTGTGAGGTTCCAGATCAACCAGGTGTCGATTGTGCCAAATAATGCATCACCAGCCTGGACTGCAGCCTTGACAGCGTCAACATTCTCCATCAACCATAATAATTTCAGAGCGCTGAAATAT

>Isogroup_468 CAAACCATTTACACAATTAGAATAGCACACTTACAAAGACTCGGGCATAATTAGATAGAGCTTATGTCCCGTAGGAGATTTGTCGACCCTAACATAATACAATACGATGTTTTACGTGTTGGACATCAAACAGTGTAGTGCAGATAATAGGGCGGAATCTGTCTTAAAACTATAAGACAGTTCTGCCCTGAGGCTAATTGTCTCCATCTTGCTTTTGACTGAAGGGAGCGACCATTTTAGTTGCGGTTCTTGGATTTCTCCACTGCCCTTGGAGCAAGTCCAATTGCATCTGAACCTTTCATGATCCTGAGCCTACGACAGGAGTCAGCGAACATCTCCCATGGAACATCACCAACGAGCATCCAGTCACCATCCTTGTCTTCAAAAGTCAGCACATATTCGCCATCCGTGCGTCTCTCAGATAAACCATCCTTGCCAGTTGTGAAGCCAACAAACATCTTCTCGAGTGCAGTCGACAGGTCTTTGTAGTTCTTGTAAGTCTTGAGGTCGATCTTCCTCAG

>Isogroup_469 TTCTTGGACGATCTCTTGAGGAAAGTGCTAGGCTTAAGGCATCCAGCGATATGAATGAGCTCATTTCACTCTTATCTCCTCAATCAAGGTTAATTGTTACATCCTCAAGTGATGACCCTTCATCAGATGACATTTTGAATTCGGATGCAATCACAGTTGAAGTTTCTGTCGATGATGTTCGTGTTGGAGACTCAATATTGGTTCTTCCAGGAGAAACTATTCCTGTAGATGGAAATGTCATTGCGGGCTCAAGTTTTGTTGACGAATCAATGCTTACTGGAGAATCCTTGCCAGTAGCCAAGGAAATAGGACGTCCTGTATTTTCTGGAACTGTGAATTGGGATGGGCCTCTAAAGATCAAAGCTACAACTACTGGACCATCGTCAACAATTGCCAAGATAATCCGTATGGTTGAGGATGCGCAAGCACGAGAAGCTCCTGTTCAAAGGCTTGCAGATTCAATTGCAGGGCCATTTGTGTATACTGTTATGACATTGTCTGCAGCAACCTTTACTTTCTG

>Isogroup_470 CTCCGGCTTCTAATTCTCGGATGCCACCTCTTCCTCATGAACCAGCTGGATTCTATAATGACCGTGGTGCTACTGTCGACATACCTCTTGATTCAACAAAGGACTTAAAGCAAAAGGAGAAAGAACTGCAGTCGAAGGAGGCTGAGCTTAACAAAAGAGAAAGGGAATTGAAAAGAAGGGAAGAAGCTGCTGCTAGAGCCGGTATCGTCATTGAAAACAAAACTGGCCACCATTTATGCCCATCATTCATCATGACATTTCAAATGAAATACCAATCCACCTACAAAGGATGCAATACCTTGCATTTTGTTCACTCTTGGGATTGACGCTATGCCTTTTCTGGAATATCATCGCAACCACAGCAGCATGGATTAAAGGGGAAGGTGTGATCATCTGGTTGCTAGCCATTATCTACTTCATCTCTGGTGTCCCTGGGGCCTATGTGTTATGGTATCGTCCGCTTTATAATGCCATGAGGACTGAAAGCGCTTTGAAGTTTGGATGGTTTTTTCTGTT

>Isogroup_471 TCCTGTAGCATCTGTGGATGATACTCCAAGATCTCTCGGTAAATTAATTCTCTAACATCATCTTTTGTCACCTTTCGTCTCTCAAACTCAAACTCAAGTTTCGAAATCAGATTTCTTGCAGGCTCACGTTCCACATTAGCAAGAGTTGCAAAATATGGATCGGCCAAAGCCTCTTCAGCTGAAGGGCGGTCTTTAGGATCAAATGCAAGTAAACGCCCTAGCAGGCGAACCGCCAACGGATCAGCATTAGGAAATTTCTGAGTCAAGGGGACAGGATTTTTTTTCCGCATGCAACTTAAGTATCTCCTGGCCTTCTCATTTCGAATCCGAGATAAGGTTTCTGATGATGGAGTTCCAAGAAGATCTGTTATTATATCTAACTGGTGGACGACATTCTTCCCAGGAAAAAGTGGTCGTCCAGTGAGAAGCTCAGCGAATATGCATCCAATACTCCAAATATCAACAGCAGGAGTGTATTTCGAGAAAAAAGAGCCGCATAATTCAGGTGCTCT

>Isogroup_472 CAGTCCGAGCATCCCAAAGAATAAGACAAGCATCATCACCCACACTACAGAACTCCTGTGCACTTGAAGGGCAGAACTGGACATCTTCAACAGTGCTATCATGCCCGTGGAATACACCTCGAGGATCAACTTTAGGACTATCCTTTTCGTTTGCTGTTTTGCTATGCTTGCTGCCTGATGCCCCTGGCGAAGTCTCACTTTTTGAGGAATCTCCAAGTCCAGATATGTGATCTTGGATGCTCCACCAGACCACAGACTTGTCCTTTCCTCCTGAGAGCACATATGGTTCTGCTGGACACATGGCAAGAGCAAACTCAGCATTTTCCTCATGTCCTCTTAATATCAAATCAGGGCGAGAATCAGTTGCTCCTAGGACAGCATGTCTATTTGGCTGGGACTCAACATCCCAAATAAGCACATCTGGACTGTCCGTATGAGTGGCGATGATCCTACTGTTCTGTGGAAGCTCCCTGATTCGGTTAACCTCTCCAGGATGAATGATAGTCTT

>Isogroup_473 CATCAGTTGCTATTGGCAGGGACCTTCAAGCTGCCCGTGTCACAGCAGCCAACAAAATACGCCTATGGAACAAGGGTGTGGACTCAGAAAGCTTCCATCCCCGTTTTCGTGATCAGGAAATGCGTTCAAGGCTAACGGATGGTGAACCAGAGAAGCCACTGATAATCTATGTTGGACGCTTAGGAGTTGAGAAAAGCTTGGATTTCCATAAGAGAGTCATGGACCGACTTCCAGGATCAAGAATTGCGTTTATCGGAGATGGCCCATTCAGGCCTGAACTTGAAGAGATGTTTTCCGGAATGCCTGCAGTGTTCACGGGTATGTTGCAAGGCGAAGAACTATCGCAGGCCTATGCCAGTGGGGATGTGTTTGTGATGCCTTCCGAGTCAGAGACACTTGGTTTTGTTGTGTTGGAGACAATGTCATCCGGAGTTCCGGTGGTCGCTGCTCGAGCTGGAGGTATACCTGACATTATACCCGAGGACCAGGAGGGAAAGACCAGCTTCCT

>Isogroup_474 TCGGCTACTGCCTGTATCAGGTTTTCCAGCCTTGGATTCAGAAGAGGAAGCTTGCATATGCAAAGCACAAGCATGTGATCTCTGGGATTTTAAAGCATGCCCAAATGCAAGCACTGGGCCGGCTGCTTAACGATGATGGCACACCCAATGAGAACGTCATAAGAAAATTGTTCCACAAGATCGACATGGATGAGAGCCGTAATCTGTCCCGAGCAGAGCTCCATGCACTTATTATCGGGATCAACTTTGAGGAGATTGACTTTGACAAGGATGACGCAGTCGGCAAGATCATGGATGACTTCGACACTTCAGGCAACGATATTGTGGAGGAGTCTGAGTTTATCGAAGGAATGAAAGTATGGCTTAATGAGGCCAAGCACAAGGTGCCGGCTAGTGGTGCCTACTCCAATAAGTTTGTCAGTGACTACCACACGAGAACAAGGGAGGAGCACGACCAGCTGGTTGACAAATCTGACGAGGCAGTGGAGAGTGTGGAGAACCCTGGCCTT

>Isogroup_475 TGCATCCACTTTAAAAGGAAGAGAGCAACTAAATCCTGAAAAATCTGGAGCTGAATATGTGTCAAGGAATCTAGCGAGATCGTCTGCCAGAAGAGGTAGATAAACAGCATTGTATCCAACAGACTGAAGACATTTATTGTGCAAAATTGGACTCTTGCTCTGCTTTACTGGGTTGGCTATAAGACCAAGAACCGTTGTATCAGGTCCTATACACCTTATATTGTAAATATCCAATAATTCTTCGAGTGTTGGCTGCCCAGATGCTGATGTCTTTGTAGCATCGAGTATTCCAAAGGTAAGATATCCACCAAATTTTGGGGATAACACCCTTGACATCAAACCTCTTTCACTCATCACCAATCCAATTATAGGCACTTGGCAGTGCACCATCACTTGGAACATCCTTGACACATCAGCAATGTCTGTGGCAGTGGTTGCAACTTTAACTATGTCTGCTCCAACTGCTTGTATTCGAGCTACAAGGTTTGTAAGCTCCTCACAGGATGGG

>Isogroup_476 TTGCCCACACACCATACTTTATACTCTTGTGGATATCATCCTCACTGTATGATTTAATGACAAAGAACTTTGCATCATCATACTGGACAGGGAAATCATCTAGGTTGAACTGTGCCTTATCAGGTAAATCACTAGCGTCCTTGGTTTCACTTGAAGGAAGGGTCTGCCCTTTCACAGCGATAGTAACTGTATGGCCAAATGTTTTCTGGTTCTTGAAACGACCTGACCTAGGACCTCTGTTCAGCTCAATTGTTCCATCTTGACTCTCGTTGCCAAAAACCATAATATCCGTTGCCACGGCCTCTGGGCCTGTTGTATCTGTTATCCATAGTAACACCCCACCGACCATATAACCTGGAATCATAGCCATTGCTTCCGTAGCCACCACCATAGCCGCTGCTTGCGTATCCATTGCTTCCGTAGCCACTGCTTCCGTAGCCATTGCTTCCATACAGAAGACCGGTTTTGACAGAATCCCATACTGGCTGTAAGACCGGGTGCTCGG

>Isogroup_477 CTTTTGATGTCTCTGTATTGGAGGTTGGAGATGGTGTGTTTGAGGTGCTTTCCACATCTGGGGACACACACCTAGGTGGTGATGACTTTGATAAGAAAGTTGTAGATTGGCTTGCGAGCTCCTTCAAGAATGATGAAGGCATTGATCTTCTGAAGGATAAACAAGCCCTTCAACGTCTTACCGAGGCAGCAGAGAAAGCTAAGATGGAATTGTCAACACTGACGCAGGCGAACATTAGCTTGCCATTCATAACTGCTACTGCTGATGGGCCAAAACACATCGAGGCAACTCTCTCTAGAGCCAAATTTGAGGAATTGTGTTCAGATCTCATTGATAGGCTCAAAACTCCTGTTATAACGCCTTGAAAGATGCCAAGCTATCTGTTAGTAATCTAGATGAAGTGATTCTTGTGGGTGGATCCACCCGAATCCCTTCAGTGCAAGAACTTGTAAGGAAGATAACCGGCAAGGATCCCAATGTTACAGTCAACCCTGATGAGGTT

>Isogroup_478 AACAACACTATGATCAGCTTCAACATGCTTGAGGCCGGTAGAATCAATGGTGTCAAAAGGTTCTTTTATGCATCAAGTGCTTGTATCTACCCTGAATTTAAGCAGCTTGAAACTGTTGTTAGCTTGAAGGAGGCAGATGCTTGGCCTGCAGAGCCTCAAGACGCTTATGGCTTGGAGAAACTTGCTACAGAGGAACTGTGCAAGCACTACACAAAGGACTTCGACATAGAGTGCCGAGTTGGTCGGTTTCACAATATTTATGGTCCATATGGAACATGGAAGGGTGGAAGGGAGAAGGCACCTGCTGCTTTCTGCAGAAAGGCTCTAACCTCCACTGACCGCTTTGAGATGTGGGGAGATGGTCTGCAGACTAGATCCTTCACATTCATTGATGAATGTGTGGAGGGTGTCCTCAGGCTAACGAAGTCAGATTTCTGTGAGCCTGTAAACATTGGAAGCGATGAAATGGTGAGCATGAACGAGATGGCTGAGATA

>Isogroup_479 AAGATTGTCATTGCCAAACTCCGAACCATCAAAATGAACAAGTAGATCGAATCTAGATGGCAAAAAACCATCAATGCACAGTTCTATGCAACACAACTATTGGCAGATCTATCCCGACACCATTGTTACCCTTATTACACTCCGAAAGAGTTCCTTGTAATGGTGCATTTAACATGGAACATGTTTTGAAGTGATATGTCCATCTAGCAACAATTGAATCGCGCTGCACAGTTGCTTCTCTTATTTCAACTCGGGAGCATATAGAGCATGGCAAGGTGACACTGTATGGTCAGGCAAGCCGCTTATAAACTCGTTAGCAGCCTGAAGACAGCTATGCAGCATTTTCTTCTCTAATCTTACAAGTCTAGTAGCAACTTCTTTCTTTGGTTCCAAATTACCATCTGCCAACATTGATTCATCCTCATCCAAAGTAGTTGGATAACCAGCCAACCTTGATTTCAGATACACAACAAGTTGGTCCA

>Isogroup_480 AGCAGGACATGGGCTAGCGCGACGTCTTCCACGTGCACTGGCCCGATGAAGAAATCTTTATACTCCTCTGTGCAACCTTCAAGTAACCGAAGAAACATGGCCATGCTAGCATTGACCCTTGGCGGAATCATCTCGCCCAAAACAGTCCCTGGATTGACCGCCACCACATCCAAACCATTCTCCTCTGCAAACTTCCATGCGGCCTTCTCTGCCAGTGCCTTCGATGCAGGGTACCTGCTCCTGGGTGTATTGTCTGAGCAACAAATGCGGGTTGTTTCCCAAAACAATCATTTGCTTCGTTCTTAGCAGACATAGATGAACCAGCACATGGTGAGACAGAACCAGAAAACCCAGGTAAATCTCCATGTTCATCTTTCAAATTAGCAAAGTCATGACACTTCAATTCCACCGGATCTTCAAATATAGAGCATCTAGGTTCAAAAACTGGAGAGTCCAAGATAATATCCGGTT

>Isogroup_**481** GGGAGCTATGTGACCCATTGTCCCGCGGATCCCTGTCGTCACTGTGTTTCTCCCCATGTCCATCATCTTTGCCAGCCCAAAGTCTCCTATGACTGCTTCAAACTTCCCATCGAGTAGGACATTGGCAGCCTTGACATCACGGTGGATGATCTTGGGGTTGCAATGCTCATGAAGATATTCCAAACCACGGGCAGCACCAAGTGCGATTCTCATTCTTTTTGGCCAATCTAATGTTGGTTCCTTAAGTTCTCTTAAACGGGAAGCAACACTTAGGTTCTCCATGAAAGGGTATACCAAGAGCCGCTCTGTTGGTGTCGTGCAGAACCCTATCAATCTTAATATGTTCTTATGCACAGCAATGCTTATCAATTCCACTTCTCTGAGGAAAGCCATTTCGCCCGTCGGGACTTTCCACTTCAAATAGTCGTTTGATTGCAACCTTTTGGCTATCTGGACCTGGAAGCACTCCTTTG

>Isogroup_482 CATCATGGAAATGTTGGTGGGCAGGAGCATGAGGCAGTCTATGTAGATCCGTCTAGTTCCTCCTCATACCCTGAAAGTGACGACTGTTTTGAAATGGAGGAAGAAGTAGGGAAGAGATTTTTTCCCCATGGTCCCAGTTCCCCATGTCCCTAAAATTAATGGGCAAATTCCATCATTCGATGAAGCCACCATGGACCATGAAAGGCTGTCAGACAGGTTGAGGCTGTATGAACTAGTTGAGCACAAGGTGAAAGGTGATGGTAACTGTCAGTTCCGTGCGCTATCGGATCAGCTTTACCAAACTCCCGACCATCATGAGTTTGTGAGGGAGCAGATAATTAGTCAGCTTAAAAGTAACCGCGATGCCTATGATGGATATGTCCCCATGGCATATGATGAGTATTTGGAGAAAGTATCGCGAAACGGTGAATGGGGTGACCATGTGACTTTACAGGCAGCTGCTGACAA

>Isogroup_483 ACGGGCTGCCACGCCTTCACCCTCACGAAGCAGTCCAAGAAGCAAGTGCTCAGACCCAATATAATTGTGTCCTAGTTGGCGAGCTTCCTCCAATGAAAGCTCTAAAACACGTTTTGCGCGTGGTGTGAATGGTATTTCGACCGCAACAAAACCATTTCCCCTCCCGATGATCTTTTCCACTTCCACGCGAGCATCCTTAAGATTGATACCCATGGACTTGAGCACCTTTGCGGCAATACCAGTGCCTTCTCCGACCAAACCCAATAGAACTTGCTCGGTTCCGACAAAGTTGTGGCCTAGGCGCCTTGCTTCCTCTTGTGCAAGCATGATGACCTTTATAGCTTTTTCAGTAAATCTCTCGAACATTGCTCTGGTTACAAAACGACAACGTCTTCCGCGTCTCGAGGATCTGTAAGATGCCGCTGTCAATCTGAAGCTGGACGAGGGCATTGTTGTGAAGCTAT

>Isogroup_484 AACAGGTATTGTGTTGGATTCTGGTGATGGTGTCAGCCATACAGTGCCAATCTATGAAGGATATGCCCTTCCGCATGCCATCCTTCGTCTTGACCTCGCTGGACGTGACTTAACTGACTGCTTGATGAAGATTCTTACCGAGAGAGGTTACTCCTTCACCACCACTGCCGAGCGGGAAATTGTAAGGGACATCAAGGAGAAGCTCGCATATGTGGCTCTTGACTATGAACAGGAGCTGGAGAATGCCAAGAGCAGCTCCTCTGTGGAGAAGAGCTATGAGCTGCCTGATGGGCAGGTGATCACCATTGGGGGCAGAGAGGTTCCGTTGCCCTGAGGTCCTTTTCCAGCCATCCTTCATTGGTATGGAAGCTGCTGGAATCCATGAGACCACCTACAACTCTATCATGAAGTGTGATGTGGATATCAGGAAGGACCT

>Isogroup_**485** AAGGCGTCGCTGTCTGACCCCAAAATGGCTGAGAAGCAAGGTGATGTGGTCGTGAAGAAGGCAACAGAGTATGGCGCAGTGGAGATATGGATGAATGAGCGTGTCAGGAGGGCATGCGCTAGCAGCTGCGCAGATTTTCTCTATGGCTTTCGTGAGAACAAAGCCAAAGGTAAGGGGGATGAATACTGGCTTATTTGGCGTTATGAAGGTGAGGACACACTTTCTGCTCTCATGCAAAGCAAGGAGTTCCCCTACAATGTCGAGACTAAGATTCTCGGAAATGTTCAAGATTTGCCAAAAGGAATAGCGAGAGAGAATAAGATCGTTCAAACGGTGATGAGACAACTCTTGTTTGCTCTTGATGGACTTCATTCGACGGGGATTGTTCACAGGGACATAAAACCTCAAAAATGTGATATTTTCAGAAGAGTCTC

>Isogroup_486 TGATGGCCAAGCCTGCAGCATCAACAGGGACTAGTTGGCCAGCAAGGAGTTTAGTAAGGAGTGGTGTCATCGCTATAGCACCAATAGTCGAACAAGTGGTCATAAGAACTGAAAGTGCAACATTTCCTTTGGATATGTAAGTTGCAACATTTGATGCTTGACCACCAGGACAACATGAGACTAAAATAAGACCAGTTGCAAGAGGAGCAGATAACTTCAATGTCAATGCGATAGCATATCCTAGCATAGGTTTGATCAAATACTGCGCGAGAAATCCCACACCAACCGTCCATGGATTCCTCAAACATCTCCTGAAATCTTCGAATGTCAATGTTAGTCCCATTGACAGCATTAGGAATCCCAGGCCCACAGTAAAAAGATCAGTCTCCAACCAGGTGACCATCGACGGCTTGTAGATGCCAATCACG

>Isogroup_487 AAGGATGATGTCAAGCTCATGGCTGACACTAACCTAGAGGCTTACAGATTCTCCATCTCCTGGTCAAGGCTCATACCAAATGGGAGGGGAGCTGTTAATCCAAAAGGACTGGAGTATTACAACAACGTGATAGACGAGCTAGTGAAAAATGGGATTCAAGTTCATGTTATGCTTTCCCATCTCGATATCCCACAAGTTTTGGATGATGAGTATGGAGGATGGTTAAGCCCTACAATTGTGGAGGATTTCACAGAATTTGCAGACGTGTGCTTCCAGGAGTTTGGAGACAGGGTTTCATATTGGACTACAATAGACGAACCTAATGTCAGCGCATTAGGGTCTTATGATAACGGACTATTTGCACCCGGACGTTGCTCTGATCCTTTTGGAATAACAAAAT

>Isogroup_488

TCCATAAAGTTTGAGCAGGACATTGAGTGTGGTGGCGGATATATTAAGCTAATGTCTGGTTATGTCAACCAGAAGAAATATAGTGGAGACACTCCATACAGCTTGATGTTTGGACCAGATATATGTGGAACTCAAACAAAGAAGCTGCATCTTATACTTTCTTACCAGGGGCAGAACTACCCTGTCAAGAAAGATCTACAATGTGAAACTGACAGGTTGACGCATGTTTACACGTTCATTCTTAGGCCCGACGCATCTTATAGTATACTTGTTGATAATCGTGAAAGAGAATCTGGGAGCATGTATACTGATTGGGACATCTTACCTCCTCGTAAAATTAAGGAAGTTAATGCCAAAAAGCCTAAGGATTGGGATGACAGAGA

>Isogroup_489 AGCAACAAAGAAAAGACAGATGAAGAAAAAGTGCAATGGAGAAGAAGAAGGGCCAGTATGCCAAGTTCTGGAATGAGTTTGGCAAATCAATAAAGCTGGGGATCATCGAAGATGCAACAAACAGGAACCGTCTTGCGAAGCTTCTGAGATTTGAGAGTTCCAAGTCAGATGGCAAACTTGTCTCTCTCGACGAGTATATTTCAAGGATGAAGTCAGGACAAAAGGATATCTTTTACCTCACTGGAAGCAGCAAAGAGCAGCTAGAGAAATCTCCATTCCTTGAGCAGCTAACCAAGAAAACTATGAGGTTATCTACTTCACCGACCCTGTGGACGA

>Isogroup_490 AAAAACCAATAGATTGGTTACTGTTGAAGAAGGCTTCCCACAACATGGGGTTGGTGCTGAAATATGCATGTCTGTTATGGAGGACAGTTTTGAGTATCTTGATGCACCAGTTGAGAGGATTGCAGGAGCTGATGTGTCCACGCCCTATGCTGCAAACCTTGAAAGGTTGCGTTTCCACAGGTTGACGACATTGTCCGTGCAGCAAAGCGAGCTTGCTACAGAGCAACGTCAATGGCAGCAACCGCCTAAATTCGAATGGATCTTTTGTTTCTCGGCCTTCTCTTTTCTTCTCTGTCCAGAAAACAT

>Isogroup_491 GCGTGGGTGCTTGACAAGCTCAAGGCTGAGCGTGAGAGGGGTATCACCATTGATATTGCCCTCTGGAAGTTCGAGACACCAAATACTCCTGCACTGTCATTGATGCTCCTGGACACCGTGACTTCATCAAGAACATGATCACTGGGACCTCCCAGGCTGATTGTGCTGTGCTTATCATTGACTCTACCACTGGTGGTTTTGAGGCTGGTATCTCCAAGGATGGCCAGACCCGTGAGCATGCTCTCCTTGCTTTCACTCTTGGTGTCAGGCAGATGATCTGCTGCTGCAACAAGATGGATGCCACCACCC

>Isogroup_492 TTACGATGCCCGCCTCCATGAGTATGGAGAGGAGAATCATATTGAAGGCTTTCGGTGCTGAACTGATCCTGACTGACCCACTCTTGGGAATGAAAGGAGCTGTCCAAAAGGCGGAAGAGCTGGCAGCAAAGACACCCAACTCATACATCCTTCAACAATTTGAGAATGCCGCCAACCCAAAGATTCACTATGAGACAACTGGGCCTGAAATCTGGAAAGGCACAGGCGGGAAGATTGATGGCCTTATATCTGGCATCGGGACAGGTG

>Isogroup_493 TTGTGAGAATTCTTAATTCAAGAGTTGTAGGGAGGGACTTATGTCACCACAAACAGAAACTAAAGCAAGTGTTGGATTTCAAGCTGGTGTTAAAGATTATAAATTGACTTACTACACCCCGGAGTATGAAACCAAGGATACTGATATCTTGGCAGCATTCCGAGTAACTCCTCAACCTGGGGTTCCGCCGGAAGAAGCAGGGGCTGCAGTAGCTGCCGAATCTTCTACTGG

>Isogroup_494 TGCACTTCTCAACATACTTTGCAGTAGGCGTGTCCCAGTTCTTGGCGTTCCCTTCATTCACCGAGTAAATCTTTCCTTTAGGAGGTATCTTGATTTCTGGGTGCGTCATGATGAACTCCCCAAGAGAAGGGTCAAGCGTGAAGCCGTTGACACCACTCCCAGTGCTCAGAACGAGCGTGCATGAACTCCCATACATGCA

>Isogroup_495 GGCCACATGCTTGTCCAGGAGCAGGACAATGTCAAGCGTGTGCAGCTTGCTGACACTTACATGAGCCAGGCAGCTCTGGGTGATGCTAACCAGGATGCCATGAAGACTGGTTCCTTCTACGGTTAGAACACTCTCAATACACCCACCATCTCTTGCTGCATAGGAGGAGGTAAAGGAGCACAACAAGGTACCTG

>Isogroup_496 ATGGTGTCATGGTTAGCAATGCACAAGAGGAGCTACTCCAATGGCATTCTGAAAATGCGAAGGATAATCCTAAGGTAATTCATGCAACCGAGAGGTGTGCTGCTGGTATTATTCAAGCTATCGGGCACTTCAAGCTAGGACCTAATGTTTCTCCAAGAGACGTTGAGTTTCCTTACGTCAAGGACGACCC

>Isogroup_497 AGCAGGACAGTTACATGCAGAGCAGAGCCGAGGCTCTTCAAAATGTGGAATCAACCATACATGAACTGAGCAACATCTTTACACAGCTGGCAACCATGGTGTCTCAGCAGGGAGAGCTAGCAATCAGAATCGACGAGAACATGGAGGAGACAGTAGCTAACGTGAGGGAGCACAGGGTCAGCTCCTGAA

>Isogroup_498 TTAGGGTCGATGGTGGATTCAGGAGTGGCCTAGATGTCCTTTTGGCAGCTGCCATGGGTGCGGATGAATATGGTTTTGGTTCTGTAGCTATGATAGCTACAGGATGCGTCATGGCACGCATTTGCCACACAAACAATTGCCCAGTTGGAGTTGCTAGTCAGAGAGAAGAGCTTCGTGCTAGGTTTCCTG

>Isogroup_499 TTAAGATAAGCACCTCCGCATTCGAGGCCATTCTGAAGCCTCACTTCAAACTGCAGAACAACTGTGCCATCCTTCAAAGTAATTGGGTAATCAAGCTCTTTGATTATGGCATATTTCTTGGCTGGCTCACTAACAAGGAGGCCAAAGTCTTCATGGCCATCACTCTTGGCATGCTTCCATACACCTTT

>Isogroup_500 ATCAGGTGGTAGGGTGATTGGTGCCCATACGGCCCTTCGATGGAGGCGGTTATGCGATCGGGGTCGCTCCTGGTCTTCTTCTCCTCGACATCGTTGATGATGCTCTTCAGCTTGTCAGTCCATGTGCCAAGAACTTTTATGAGGATCGACATGTG

>Isogroup_501 AGCGTGGTCGCGGCCGAGGTACATCCCAACACTGACAAGGACGAGCAGAGCCACCGAAAGAACTGGCCAGATAATGTGTGCTGGGAAGCCTCCCCTCTTACTTGGTGCTCTCAAGTGTGACTGAAGACCACACTCCTGCTTCTCCTTGTCGCTTTTCTCCAGTGTCTTACGCTGCCTCATAACTGAATTGGTGTCTGGAACAAGCATTGCATCATCGTTATCAGGAATGTCCGTTTGTGCATCAGCCTGGCTTCTGCCACCAGAACCAACATTGCCAGAGAAGTTTGTTTCCACACGCTCTTCGATCTCAGCTTTCATCCCTTTGTCACTTCCGTTTCCTTCCATAATGCAATTTGACAAACTATCTACACTAGAACATGATCCTGCATTAAGGTCCAATTGTAGCCGCAGCCTTCGCACTGCAGCCCCCTGTTGAGCTAATGAACTATCTGTGCTTGAACTTGGAGCAGGCTCTCGGCTCATAATATAAACACCTGAGGACACATCATCATCTTCATCTGAAGGCACATTTGCACTTGGAGCAGGGCATCGGTTCATAATAGTACCTGCCCGGGC

>Isogroup_502 GAATGCAAAACTACATGGCAAAGCGTCTGGAGAAACACTATCCAGTTCTTTTCCGTGGAGTCAATGGAACTGTTGCCCATGAGTTCATCATTGATTTAAGAGGGTTCAAGGCAACTGCTGGTATAGAGCCTGAGGATGTTGCGAAGCGTTTGATGGACTATGGATTCCATGGACCAACCATGTCATGGCCTGTTCCAGGCACACTTATGATTGAACCCACTGAAAGTGAAAGCAAGGCTGAACTAGACAGGTTCTGTGATGCCCTTATTTCCATCAGGGAAGAAATTGCACAGGTAGAAAATGGCATAGCAGATGTGAACAACAATGTCCTG

>Isogroup_**503** AGCGTGGTCGCGGCCGAGGTACATGCTCAAGCCGCTGCTGGGTGTGCTGATTGCAAGGGTGTTCAAGATGTCATCGACCTTCTTTGCAGGTTTTATGCTTACATGCTGTGTCTCGGGTGCGCAGCTTTCGAGCTATGCTAGTTTCCTCGGGAAAGGAGATGTTGTGTTGAGTATTCTGCTGACAAGCTACTCAACCATATCTTCGGTGATTGTGACGCCTATTCTCACTGGATTATTGGTTGGTTCAGTGGTTCCAGTGAATGGAATCGCGATGGCGAAATCTATTTTGCAGGTGGTTCTTGTTCCTGTGACACTTGGTCTCCTGCTGAATACCTATGCAAAACCAGTTGTTAATTGCATACGGCCAGTAATGCCATTTGTTGCTATGGTGTGCACATCACTCTGTATTGGGAGCCCTCTTGCTATAAATAGGAGCATGCTCCTGTCAACACAAGGACTCATGTTACTTCTTCCCATAGTGACTTTTCACATTGCAGCCTTTGTTGTGGGTTACTGGGTTTCCAAGTTGCCTCAGCTGAGACAAGAAGAGCCTGTTTGTAGGACTATTTCAGTGTGTACCTGCCCGGGC

>Isogroup_504 AGCGTGGTCGCGGCCGAGGTACTTTGCAAGACGCTCTGATCTGCAAGGTGCTCCGGTCTTGATCTGGCCCGTAGATAAACCAACCGCCAAATCAGCAATGAATGTATCCTCAGTTTCACCACTTCGGTGACTGGTCATCACACCCCAACCAGCACGTTTTGTACATTCTCACAGCCTCAATGCTCTCGGTCACAGATCCAATTTGGTTAACCTTGAGGAGAAGAGCATTGCATGACTTCTCTGCAATCGCCTTAGCAACCCTAGTTGGGTTTGTCACGAGGAGGTCATCTCCGACAATCTGCACTGGCTCTCCAATTTCCTCAGTCATCTTAGCATAGTGAACCCAGTCATCCTGATCAAATGGGTCCTCAATGGAGACAATTGGGTATTCACTGACAAATGACTTGTACCTGCCCGGGCGGCGCTCG

>Isogroup_505 ATGCTAGAAGAAACAGATTCTCTTTCATCATCCTTACTACTGTAGGACTCCTCTAAGATAGGATGCACGATCAGCTCAGGGCGTATAGCTTTTGTATCACCGTTGCAGCATCTTTTGTGCAGAAAACGTGAGAGAGATCTGCGCCAACTTTCAAGGCAGAGATAGCAGCAAAATAAGGAGCGCCGGTGTACCTCGGCCGCGACCACGCT

>Isogroup_506 AGCGTGGTCGCGGCCGAGGTACTTGGATTCCGCATCTCCTTCAGCCCTCTTGATCTGGAGTATCTTCTCCGCTTCAGCTTTCTCAGTGGCCGCCAACCTCATCCTAGCAGCTGCATTGATCTCATTCATGGCTCTCTTCACATTTACATCTGGCTCAATATCAACAATCAGAGTTTGCACAATCTCATACCCACTA

>Isogroup_507 CGTGGTCGCGGCCGAGGTACGTATAAATCTGGATCATCCTCAAACTCTGGATTATCAATCCATGGGGTCGTTCCATTTACCCTTCGTTAGTTAGGGTTCGTTAATTTTACTTTGTCGCTTCCTACTGTTCCTTTCGTTATGCTGAATTACGGTTAGTCCTTCCTTAGGTTTCCGTAAACTAGCCACTCATCATCGTC

>Isogroup_**508** AGCGTGGTCGCGGCCGAGGTACAGCTGGGACTTACACTTACATGAATAAGCGAATGACTGGTCAGAATGTTACTGCTGTTATAGTTTTACTCTTAGTTATCAGTTCCTACAACTGCAGTCTGATTCCTTCTCAGCAGCTGTATTGACTTGCCGGCTCATATAGGTTTCATAGATCTCCACAACGATCCTTGGGTCCTTGTCCACAAGTTCCTTGTATTCATCCCTCTCTGCCAGTGTGGGCATGCTTTCAACGAGTGCTGACATAGAGGCCTCAAGCAGCTCCTTCGCACTGTGCCTATGTGCAAACGCGTAATGGACAATGGCATTATCGTTGTTCACCTTGGATGTAATGAATTTTTCACAGTAGGCCTTCAGGTGCTTTACTTCATACTTCTCAGCCAACACCAAGAGATCACAAGCCATTTGCTCGTCTAGGAGAGCTTCAGCTGTGTACCTG

>Isogroup_**509** AGCGTGGTCGCGGCCGAGGTACTCGTCCATGTCGACGTAGTTCTTGTTGATGGAGTCCATCATGAGCTTGTTGCACTCTGGCTCCATCCAGGTGGTGACGAAGGAGGCGAGGTTGAGGCGCGGGTTCCGTCCACGCATGAGCTCGTCGTT

>Isogroup_510 TCGAGCGGCCGCCCGGGCAGGTACTTCCCAATCAGCTTCTTCTCCTGTATGAACTTCACATTTGCTAGAATCTCAGCTGATAACTCAATGGCTTGGTTGAAACCGTTCTCACCACCATATGAAACATCAACCACATTAAGGATTTTGGCCTGAAGGCGCTGATCAAACATGTCAGATTGGCTCAGTTCTGTTTTGAAATCAGCAGAACCAGCCAATATTAGCCCCGCAACATTAGGCTGGCTGGTAGCTGGGTTGATGAAGAACTGAGTAGCAAGTTCAGCTGTCTTTCGGACATAATTATGACGCTTTTCCATCCGAAGACGAGCAAAACGCAGAGCAGATTGCCCTCCTCTTCCATGCTTCTTTGGGAGATCTACAGTAAATTTGTGAAGCACTTCACGAGTATTGCCACTAAGTGTGCCAAATAGGGTTCCATTACCATCCATAATAATGAAGCCAAACTTGTCATCAGATTCCAAGAGCTCACTCAAGGCTTCAGTGTGAAATTTGTTGTCGCAAAGGTACCTCGGCCGCGACCACGTTGCATC

>Isogroup_511 AGCGGCCGCCCGGGCAGGTCCGGACGCAGAAACTACCCTCGAGCTTCTAAAGGACGCAAAACACAGCGGCGCAAAACTCAGTATAGAGACCTGTCCTCATTACCTCGCATTTTCGGCTGAAGAAGTTGCAGATGGAGATACTCGTTTCAAGTGCTCTCCTCCTATACGTGATGCTGCCAACAAGGAAAATCTCTGGGAAGCTCTGCTTGATGGGCACATCGACATGCTCAGTTCGGACCACTCTCCGTCGACTCCTGATCTCAAGCTAATGGAGGAAGGCAACTTCTTGAAGGCGTGGGGAGGAATATCATCTCTGCAGTTTGTCCTCCCGATAACATGGTCACATGGACAAAAGTACCTCGGCCGCGACCACGCTAAGCGTGGTCGCGG

>Isogroup_512 AGCGTGGTCGCGGCCGAGGTCAGACGGCCGGCCGAACCCAATCCAACCAATCTCGAGAGCAGGCAGCAGCAGCCCGCGGTGTTCATCCTACGATAAGCAGTGGGTCAGGCAATTGGTTTGATACAGATTGATCAATCAACAGTAGCCATCAAGGAAACTTTTGGAAAGTTTGATGCTATTCTGGAGCCTGGATGCCACTGCCTGCCACGGTGCATAGGGCAGCAGATTGCTGGATATCTTTCGCTGCGTGTGCAGCAGCTTGATGTCAAATGTGAAACAAAGCCAAGGATAATGTCTTTGTCAATGTTGTGGCATCTGTGCAGTACCTGCCCGGGN

>Isogroup_**513** AGCGTGGTCGCGGCCGAGGTACCAGAGATTCCTAAAGGCATACCATCAGAGAAGCTTCCTTGACCAATAGGGTAAATCAAGAAAACAGCAGTAGCAGCTGCAACAGGAGCTGAGTATGCAACAGCAATCCAAGGACGCATACCCAAACGGAAACTAAGTTCCCACTCACGACCCATATAACAAGCTACACCAAGTAAGAAGTGTAGAACAATTAGCTCATAAGGACCACCATTGTATAACCACTCATCAACAGATGCAGCTTCCCAAATTGGGTAAAAGTGCAATCCGATCGCTGCCGAAGTAGGAATAATAGCACCAGAGATAATATTGTTTCCATAAAGTAAAGAACCAGAAACAGGCTCACGAATACCATCAATATCTACTGGAGGGGCAGCGATGAAGGCGATAATAAATACAGAAGTTGCGGTCAATAAAGTAGGGATCATCAAAACACCGAACCATCCGATGTAAAGACGA

>Isogroup_514 AGCGTGGTCGCGGCCGAGGTACTTTTGTCAGTATCACTTCCATCTGGTTATATGGAGCTACCATCATGTGGAGAACCAGGAGGAAAATCATCAAGTTATCAAAGTATGACATCTCCATAAGATCTGATATATCTAGCTCTAGTGAACATGGATCTGAGAATTCTGTGGAATAAATATCACTGGGTTGGTAACTACCAGTTTGTGATAATACTGTGTCTGCACATTCTGATGGGACTTCTGGTAAAATATCATGGTATCCATTTAAAGGGGAGATACCACCAACGGCTGGACTTACTAGTTCCCTTTCTTGTAAAGGCCTTGGAGAATTTACTGCAAAAGTGGACTCCTTGAGCTCTACTGGGCTTGACAGAAAATCCAATTTCCTTTGGTTTTTGCATGCTGCTCGGATCTTTCAATTAACTTTTGAGGGCTTTGGCTGATGAAGCATCAACTCCTCCAACATGCATCAAATCTGCACTTTC

>Isogroup_515 AGCGGCCGCCCGGGCAGGTACCCGCATCTGAACAAAACTTGCAATCTTTCACAATGGGAAAGATGGATCTCAACCATATTCTGCAACATACCTATGCACTTTCCTCCATAGGAATATATCCTTAGAACCTGCAATCCATCATGAGGTTTGAAACTGTCGAGCACCCCGCTGTCACAAACAGAAGTCCATCTTAATCTCAGATCTATAAGATCCTTCTTGTTTCCTAAGATCTGCCACTTTTGCCTCTGCTTCTGTAACATTCTCTACCTGACATAGCTCTAGCTGACCACCAAGGTTTAAATTTTGTAGCTCTGCAACATCACTGCAATCAGGGCTACTAACTGCCGCTACAAAATATGTGAGTGTCTGCAACTCAATGAGTTTTTC

>Isogroup_516 AGCGTGGTCGCGGCCGAGGTACTGACAGTATCCATTTGCAGATTGGAGGCACAGAAGACACAACAATAGTTGCCGGTGGAGTGTAATGATCACCAAGCTTGCCCATTGCAAATCGAATGGCATTCCACAATTTATTGCACCATTGGCGATATCCAACAACTCTCTTGATATCCAGATTTATCCTGTCAGACTGAGAAGTGTAAGAAATCAGCGCAAAACGTAGTGCGTCAGTGCCGCATTCAGGAATGCCATCAGGGAAAATCTTTTATCTTCCCTTCTCTTGCAATAATTAATTCATTTTTTANCCAGATTGCCTTCTTCCAAAACGTTTTGAGGAGCTCTTCAAGGGGTCNCACCGGTTTACACCTGAGGGGGTCAACACGGTTACCAAGTGACTTGGACATTTTTGC

>Isogroup_**517** TCGAGCGGCCGCCCGGGCAGGTACAGGTCGCGAGCAGGACCCTGCCAGGGACCGGAGATTACAAGCGTGCGCGACATGAACATGACCGTGGTCACTTGCAGGAAACTGATGCTGAATATGGTAATGGTGAGCGCAGCTATAACCAACATGAACATCACAGAAGTCATGAGCAGTATGGTTATAGCCAAGATGGATATGGGCCTGAGACTGAGCGCCCAAAGCGACATGATCATTACCCTGATGATTCACACAGTAAAATGGCAACCATTTATCAGGCGCAACCTGACGATGCAGAACCTGAAGGCCCCGAGGAAGGTGAGGCATACTAGGGAGGAACTACCAGCTTAACTGGGCTGGTGAATACATGAGCTGAAACTTGAGAGCTCTGGCAAGTCCTGTTAAACTCTGGAATATCATCGTCATGAATATCATCTTCATTTCAGCAGTTGCCAATGCTTGTCACGCTGCCAATTTGTTTCTTGTGGTCATTTTAN

>Isogroup_518 AGCGTGGTCGCGGCCGAGGTACTTATTTATGCATCAGCAACCTTCCATGACTGTTGATTCAAGCACAAAGATGCAAACAGAGGCCAATGAGTTGCATTTCTGGAATGGTCAGAGCACTGTGTTTGGTAACTCAAGAGATCAACCACAAGGTTTCAGATATGAAGAACACCCATCAACTTGGTTAACTCCGCAGTTCTCCAGGCTGAACACCCACGAGTGATCA

>Isogroup_519 AGCGTGGTCGCGGCCGAGGTACTACAACTACAATACATAAATAGAAGAACATACACACTTCACCTAGATTAGGGACAGGCACGACACAGACAGGCTCACATCTTTAGAGGCCTTCCAAGCACCAGGTCCTTTCTGGGGAAAATGCAACCAACAACAGCTTGCGCTTCATGAACTGAGAGGACGTTGCGAACCACCTTCCACCACAACATTACAGGGATGATATTTACTAAAACCACAAGGCAACCACCTCTGCAGCAAGCTCAGATGTCTTCTGACTTGCACAGGTCTAGCAGTGACAGCGTCTCTGCTATGGAGAGCTCAAGGCGAAATTTAGGGCCATCATAGTGGTGCAACACTGGCCTAAAAGCATCTTCTTCCAGAGCCTTACAAAGCTTTCTAATGCGGATCCTCACCTGTGCTGGGAATCTGGATTCACCTTTACACTTCTTATCTTCCCATGCGGTGGGATCAATGTTGGATCCTCCAAAACTAGCAGCCTCAAACACCCATGGAGCCGATGGGTCGTGTAGTTGTAGAGGAAAGAGGTAGACCAGGAGTAATTGCTCGGACTGAATCACGATATCTTGCTGGCAAACCAAAAGCTGCCTCTTGAGATCCTCCTGCATGGTATCATTGTTGCAGACAAAGATGTATCCACCAAGAACTTCATTCCTTGGTAGCATCTCGCTTGTTGGCAATGTTTTAAACGCGCTTGTCAATGGAGTTGTTGCTGTTGCTACCATTGTTGTTGTTACC

>Isogroup_**520** AGCGTGGTCGCGGCCGAGGTACTGTTCTTCCAGTAAGCACTGACTTCATCCATCAACAACAACTGCATTTTCTCATCTGGACATAGTTTGGAAATTATTTGACCATATCTCTCCAAAAACGGTGATAAGATGGGTGAAACTTTTTGAACCAATGTCAAGAAGTGTCTGGCTAACAACACCAACCGCGAATTTGGCACCATTTGCAGGAACTATTTGTTCCTCAACCCACAAAATAATATCACGTGTAGTCTTCCTTCCTCTAACCATGCCAAC

>Isogroup_521 AGCGTGGTCGCGGCCGAGGTACTGCCTCATCAGCTGGGCCGTCGGTCTCCCAAAGCGCGCGCCTATTAATTCTACGCTCCTGAAGCTGCTCTTCCCTGTGGCTTTGTGCCATGCTCTTGGTCATGTCACAAGCAACGTGTCCTTTGCTACTGTTGCAGTCTCATTTGCCCACACTATCAAAGCTTTGGAGCCCTTCTTCAATGCAGCTGCTACCCAATTTGTTCTTGGGCAGACAGTTCCCTTGTCTCTGTGGCTATCTCTTGCCCCCGTCGTGCTTGGTGCTTCAATGGCATCCCTCACGGAACTTTCATTTAACTGGAAGGGTTTCATCAATGCCATGATCTCAAACATCTCCTTCACTTACCGTAGCATTTACTCCAAGAAAGCCATGACCGACATGGATAGCACCAACGTGTATGCTTACATCTCAATAATTGCTCCTATCGTCTGCATACCACCAGCAATTATTATTGAAGGACCCCAACTTATGCAGTATGGGTTAAATGATGCAATTGCAAAAGTAGGTCTGCAAAATTGTTTCAGACTTTCCTGTAGGACTGTCTACCATCTCTATAACCAGCTTGCTACAAACACATTGGAGCGGGTGGCCCCGTCTGACACATGCTGTTTGGGAATGTGTTNAAAAGGGTGTTCGTCATGGTTTCTCGATCATCATTTTGGTCACAGAATTACCACACAGACTGGTATCGGGACGTGTGTTGCAATAGCTGGTGTTGCCATCTACTCATACATCAAGGC

>Isogroup_522 AGCGTGGTCGCGGCCGAGGTACTGTTTCAGGTATGCTAGGTGCACCCGCTCTGTATCAATTGTGCCTCCGGCATATTATGTCACATTTGGTCGGCCTTCCGGGCTAGATTTTACTTTGGAGCCAGACACCTCTGACAGTGGTTCTATGGCGAGTGGTGCTCGTGGCCTCCACAGGGTGGACGCACAACCAGAGCGTTTGGGAATGCTGCTG

>Isogroup_523 AGCGTGGTCGCGGCCGAGGTACGCACGCTTAATAAGGTTGCATCAACTCATCCTTTGGCTGTTACTAATTGTAACATAGATATGGAGAGTTTAATCTCTGACCAGAACAGGAGTATTGCAACACTTGCAATTACCACATTGCTCAAGACGGGCAATGAATCAAGTGTTGATCGTTTAATGAAACAGATGACCAACTTTATGTCAGACATAGCGGATGAATTCAAGATTGTCGTTGTAGAAGCAATAAGATCCTTGTGCTTGAAGTTCCCCTCTGAAATATCGCTCACTGATGAACTTTTTTGAGCAATATCCTACGTGAAGAGGGTGGTTTTGACTACAAGAAAGCTATTGTTGATTCAATTATCATTCTCATTAGAGATATTCCTGACGCTAAAGAAGTGGGTTATTCATTTANGTGAATTCATGAAAGCTGTGAGTTCACATATCTGTCCATCAGNT

>Isogroup_524 TCGAGCGGCCGCCCGGGCAGGTACATTGAGATGGGAAAGTATGATGAGTGCATCAAGGACTGTGATAAGGCTGTGGAGAGGGGAAGGGAACTTCGTGCTGATTTCAAGATGGTTGCAAGGGCACTGACAAGAAAAGGAACTGCTCTGGCCAAACTGGCTAAGAACTCCAAAGACTACGATATTGCCATCGAGACTTTCCAGAAGGCTCTAACTGAGCATCGGAATCCTGACACTCTCAAAAGGCTAAATGAGGCTGAGAAGGCAAAGAAAGACTTAGAGCAACAAGAGTATTATGACCCGAAGGTAGCAGATGAGGAGAGAGAGAAAGGTAATGAGATGTTCAAGCAACAAAAAGGTATCCAGAAGCAATAAAGCATTACAATGA

>Isogroup_**525** AGCGTGGTCGCGGCCGAGGTACTCCATTAGGCCTTGTTGTGGATTTATGGCTGTTATGAGTTCAAACAGTATTATGCCGAAACTGTAGACGTCACTCTTCTTTGTGAACTTGTTGGTGGTGATGTAGTCAGGATCCATGTATCCATAGGTGCCCTTGAGACCTGACTTACTCCCATCAAATACTTCCTCCTTTGATAGACCAAAATCTGCAACCTTGGCCCTCATTGAATGGTCCAAGAGTATATTAGCAGACTTTAGGTCTCGGTGAATAACAGGCGGGACAGCCCCTTCATGCAGGTACCGTCG

>Isogroup_**526** AGCGTGGTCGCGGCCGAGGTACCTGACTACAGCTTCTCCTTCGCTATGACTTCTTGCTTGATTGCAATGCTGCCAGAAGGGTTTTATGACAGGGTTGACGAGGGCAGCATTGTTCCCAAGAATTCGAAGAGGTTCAGCTTCTGTAATGATGGAATCGTCCTGGAGGATGCAGATGAATACATAAAGAGTGACATTGTAATTCTAGCGACAGGATTCAGAGGAGACCAGAAACTTAGGGACATCTTCACAGCAAACTGGTGTAAAAAACTNTAAGTGGCAGGATCATCACACACGTCAGTTCCTCTATACAGAGAATGCATCCATCCTC

>Isogroup_**527** TGAGCGGCCGCCCGGGCAGGTACGCTCTTGCATGTCGTAGATCGCCGGTATTTGGTTCGGGGCGAGCGTAGACATTGTTTACCAAATTGAAGATCGCCTCATCCATGGGACCCAGGTCACGGACGTCTGTTTTCGTAGTAGGAATGAAGGCCACACGGACAGACTGCAGCTGCACGTTACGATCTTTGAACCACCGAATCATGGCCCAAGAACTCGAAATCCCTCCAATCAGATGGGTAGAGGTACCTTCGGCCGCGACCAGCTTTCGAGCGGCGCC

>Isogroup_528 AGCGTGGTCGCGGCCGAGGTACACTTTATTTTCAACAACCATTGGTCCGATATATTTCTTTACATGTTCGATGGAACTCTCTCCACCCTGGGACATAAGATTAAAAAAATTGGCATCACTATTCAAGAACTAAATCAGCCTCTGAATTCTCTAGCCTCTCCATTCCTCTTCATTATTATTGTTCACTATTTGCAGAAGAGAGTCTCCTTTGTGGCGTTTCCGGTAGTTGTATTTATCCCGTCCGCAATTTTGCATAGGTAGTCCCGCTGATCATCTCGCAGTGGCACATCGGTAAAAATCTGCCCCGTATATGTTGGAACCTTTGAAGGAAGTGTTCCCTGTCACCAGTGCCCCTTCCAAGTTGGCGTTCGTCAGA

>Isogroup_529 CGAGCGGCCGCCCGGGCAGGTACAGTGACAACAGCATTCTTAATAGTTACTCCTAGGTAAGCTTCAGCAATCTCGCGCATCTTGATAAGAACCATGGAAGAAATTTCTTCAGCTGAAAAACTGCTTCTCCTCACCCTTGTACCTCGGCCGCGACCACGCT

>Isogroup_530 TCGAGCGGCCGCCCGGGCAGGTACAGTGCAGACATCATCTCAAACCTCGCTGTCCCTGATTGACCTGACACCGAATTTGTTTGTGAAGAAGTCAGTCTTGTGCATGGCCCAGAAGAAGACAACAGTCATGAGAGCAAGGTAGGTTCCGAGCACAACACCAGTGGCAAAGATTTCATTGAGCTTCCAGCTGTCGGGCAAGGGTAGATGGGCTTAACTCTGTCCTTAGATATGGTCATGATAGTACCTCGGCCGCGACCACGCT

>Isogroup_**531** AGCGTGGTCGCGGCCGAGGTACAGAGAGGGTGGTGTTCTGAAACGGGCTGGGCACACTGAAGCATCTGTTGATCTAGCTGTGCTGGCTGGGTTACCTCCTGTCGGAGTGCTCTGTGAGATTGTGGATGAGGATGGCTCTATGGCTCGTTT

>Isogroup_532 AGCGTGGTCGCGGCCGAGGTACTTGACAGCAGCAGGCGGCATGCACAGCTCCTCAGCAAGCTCAAGAATCCTTGGAATGGTGAGCAGGAGCATGGTAGTCCCACAGGTCTTGATCACAATCTTCTGAGAGTAGATAAACAGGCTAGACTCAGAGAGCACGTGATGATGCATGCCAGATAAAATAGCTGCTGCTTCTGTGAGGATAGTTTTCCTTCGCTCAGGCACCTAGCTGATATGTCTTCTTAGCGACTACATACAACAGCTAGCGGATTACTATAACTAGTAGTCTACTAAGGACACAGTATTTATTT

>Isogroup_AGCGTGGTCGCGGCCGAGGTACAAAATTGAAACACCTCTATTACTGTATGGGTTAACTTTGAGCGCAAAGGTGAAAACTGCTCAAGCTGCAGTCCCAGTTCAAGTGAATCGTCCATTTTTCGCGTGCTCTGCTCTGAAAAGCATTTGTGCCATGTGGAACAATGGCCAAAGCTTCGGGTGCTCTCGCAGAATGCTTTGTCCGTCGGTCTCATGGTATATTCAGTATTTTCTTCAGCTTCGCGACAAGCTTCCAGTCATCCATTGACAACTCATCCCTGTAGTTTGCTTTCAGAAGATCAATAGAGCGCCTCGAAATGTGGCTGATAAGCTCATCTTCCATCTCAAAATGCCTGCCAAAGCTCTTCTGCTGCTCATCTGAATTGCTCCCGATTATCTTTATAGCCACCTTTTGCCCTTTTGTAGCCGTATCGACTTGCTTGTGATTAATTTCAATAGAGGCAATCTTCCCAATATCAATAAATTCCTTCGAGGGTATGCAGATAGGTGTCCCGATCTTAGCTATTCCTTCAAGAATATCAACACCCAGAACGATTGGATCCTTCTTGTTGAAGACACAATTCGGCATAATCTTGAGAACACAGGGGAAAACAAGCTTCCTCAGCGCTTCCTTCTTCTTCTCTTCCTTTACGTTCTTAATGTATACTGTAAACTGGTC

>Isogroup_534 AGCGTGGTCGCGGCCGAGGTACGAGATATAATGGATGGATCGATTGGACTGGGTTTGAACGATTAGTAGCTGTAGTTCTTGACGAACATGCCCTCCTTGGCCTCGGCGGCCTCGCCGTCGCTGGTGTACCTGCCCGGGCGGCCGCTCGAAGTCGT

>Isogroup_535 AGCGTGGTCGCGGCCGAGGTGGGCAACTGGAAGTGCAATGGAACAAAGGAATCCATTAGCAAGCTTATATCTGATTTGAATGCCGCCACACTCGAAAACGATGTGGATGTTGTGGTAGCACCACCATTCATTTACATCGATCAGTCAGTGAACTCTTGACCTGGTCGATGTAAATGAATGGTGGTGCTACCACAACATCCACATCGACCAGGTCAAGAGTTCACTGACTGATCGCATTGAGGTGTCTGCTCAGAACACATGGATTGGAAAACGGTAGGAGCTTTCACTGGAGAGATCAGTGCGGAGCAATTGGTGGACATTGGCTGCCAATGGGGTTATCTTGGTCACTCTGAGCGTAGACATGTTATCGGCGAAGATGATCAGTTTTAATTGGGAAACAAGGGCTGCATATGCCTTGAGTCAAAAACTCTTAAC

>Isogroup_536 CGAGCGGCCGCCCGGCGAGGTACCTCGCACCGGTAACCTCCTCCGCCACGCCTTGCACCGATATTCTGTAGGAAATGGCTGACGGTGAGGACATCCAGCCTCTTGTCTGTGACAATGGTACCGCTATTACGATCCGAAGACTGTTGGGCTGGATTTTGAGGGAATGATAGCTGACATACAGGCTGCCCCAGAAGGGTCTTTTGTTCTGCTACATGGTTGTGCTCACAATCCAACTGGAATAGACCCAACTCCTCAACAGTGGGAGAGACTTGCAGATGTGATTCAAGAGAAAAACCCATATGCCTTTCTTTGATGTTGCATATCAGGGGTTTTTGCCAGTGGAAGCCTTGATGAGGATGCATTTTTCTGTCAGGCTTTTTTGAGAGCGTGGCCTGGAAGTGTTTGTTGCGCAGTCTTACAGCAAGAA

>Isogroup_537 AGCGTGGTCGCGGCCGAGGTACATGTTCCCACAAATTAATAAAACTAATAAGATGCATCTTTAAACTAATACGTAGGGAGGTAAACAATGATCACTACACTGTAATGGGAAACGATTCAGTTCTGTCACAGGGCGATGCATGATCTGAAGTCTGGAATGGCGCAATTCCGCTTTGGGCTAAAGTGTCACAAAAATGTTCTTGCCATTCCTCTTCAGTTTACACGAGCTACAGAAGATCCAGCAGGTGTTAAGGAATGTGTAACTGTAGCTCATAAGAAGTATTTCATCCTGTTATCTCTTCTCATGGCGCGGTAGACAGCCTGGACCTGCTCACTAGAAAACCACACGAATTGGTCCGATCTTTACAGAAACATATTTTCCCCCCTGAAGA

>Isogroup_538 TCGAGCGGCCGCCCGGCAGGTACGAGCTCGTCCGTGTCGGCCACGATAGCCTCATTGGTGAGATTATTCGTCTTGAGGGCGATTCAGCTACAATTCAAGTTTATGAGGAAACAGCTGGGCTCATGGTCAACGATCCGGTGTTGAGAACAAAAAAGCCTCTTTCATGTGAATTGGGACCTGGTATTCTTGGAAACATCTTTGATGGAATCCAGCGCCCTCTGAAAACCCATTGCTATTAAATCTGGAGATGTCTACATTCCACGTGGTGTTTCAGTCCCTGCCCTTGACAAGGATCAGCTGTGGGAATTCCAGCCAAACAAGCTAGGTNTTGGGGATGCTATCACAAACGGNGATCTATATGCCACCGTATTTGAGAACACACTGATGAAAACNTCATGTTGCATCCCCCCNGTTGTACTGAAGGAATC

>Isogroup_539 AGCGTGGTCGCGGCCGAGGTACTTGTCGCTTAAAGGTGGTGCTGTCCGGGTTCTCTGAGAAGACCACCTGCGTGCTGGGGTCGACGGTGGACTTGATCGCCGAGAGGATTGTCGTCCCAACAGTGTTGTTGTTGCCAGTCAATCCTTGCCATTCGATTGTCCATCCTCCACACTGGTTGCCCAAGTTGTCGGCGTGGCTTCCGGCGACGAGGATCTTACCAGCCTTCTTTGGGAGAGCAACAACGGAGTGGAGGCAGATTTTCCATTTTTCAGCAACACCAATGATTTCCTGGCGGCTTCCCGAGCGAGTTCTCGGTGTTCTTGCTTTCCGAGTTCACCAGCAAGACTGGGATCAGCATAAGGGCTCTCAAATAGCCCAATGGTGAACTTGACCCGAAGAATCCTGTAAACAGCATCGTCGATTCTGCTCATGGGGGATAACCACCGCTCTTAACTCGGGGTCTGTCAGATCATCAATGAAAATCTGTGTAGGCATAAGGAATCATGATCATGTCAATCC

>Isogroup_540 TCGAGCGGCCGCCCGGGCAGGTACCCCTGGCCCCCAGGGTCCATACTACTGCGCTGTGGGATCAGACAAGTCATTTGGCCGTGACATATCGGATGCTCACTACAAGGCATGCCTTTACGCTGGAATCGAGATCAGTGGAACAAACGGGGAGGTCATGCCTGGTCAGTGGGAGTACCTCGGCCGCGACCACGCT

>Isogroup_541 TCGAGCGGCCGCCCGGGCAGGTACTCTGGCACAAATAGAATTGGTTTTGAATGTGCATGGCTCCTTGCTTTTGCAGATACTGAGTCTACTGGAAGGAGGATATGTGCGGAATGTGGCCGCAAAGGTAAATTCGACAACATCAATTGGGAAAATATTGAAAAGAATCTGGATGAATCTGGAGAAACTACTAACCCGTGTGACCCGTTCACTATGACCTCGGTCTATACTAGCATTGCCAACTCTAGCGGGAATTCTACTGTTGATGCAAAATTCCTTGGCTAAACGAGCTCCACTGATAATTAATGGATTAAGTCTTATGGACTATGATATATTGTAATAATCTTGCAACTATGTTGCCTGTAGCCATCATGTGTGATGTGCTACCCAAAACTTTGATTTTCATGTCTTTCCTTCAGACTATGTAATAAATAAGGCTCGGAGCACCCTAGGGTGCAAGGTGAGAAGTGTTGCTCCTAAAAAAAAAAAACCGAAAAAAACGAAAAACGAAAAACGTGAAC

>Isogroup_**542** AGCGTGGTCGCGGCCGAGGTACTTGTCGGACCAGTGTCATACTTGTTGCTCTCAAAGCCTGCCAAGGGTGTGGAGAAGTCGTTCTCTCCTCTTTCCTCTTAGCAGCATCCTTCCTGTCTACAAGGAGGTTATTGCTGAGTTGAAGGCTGCTGGGGCGTTCATGGATTCAGTTTGATGAGCCCACTCTTGTCAAGGACCTTGAAT

>Isogroup_543 AGCGTGGTCGCGGCCGAGGTACAAGGAGGAGCTTTGGGAGCTGCTCAAGAAGGACAACACCTACGTCTACATGTGCGGTCTCAAGGGCATGGAGAAGGGCATCGACGACATCATGGTCGACCTCGCCGCCAAAGACGGGATCGTCTGGAGTGACTACAAGAAGCAGCTGAAGAAGGCCGAGCAATGGAACGTCGAAGTCTACTGATCATCATCAGTTCACCACTCATATTGCCATGGAAGGACGGCACATGTATATGCTGCACATGTTTCTTCCCACCTAGTTTTTCCCCTCCTTTACTCCGCAATTGAGCCGTGCAAAGCTCAAGGCTTGGATCGTATATACACCTTTGTATCTAGCGAGCGAACGAGAGTGAGTGAACACGGTGAGCTGTAATGAATTCTCCTTGGATCCTCTTATGGCTTTTTTATTGTCATTGATCATCGTGGCAAGATTAGGAAAAATTTACCCTACTTGAGATGGCTGAGATCCATGCACCAGTCATTGCAGCAGACATCAACGATGATGGGAAGATCGAAATGGTCACTGCTGATGTGCATGGCAATGTAGCAGCTTGGACTGCAGAGGGAGAAGAAA

>Isogroup_544 TCGAGCGGCCGCCCGGGCAGGTACAACACTCGAGTTGTAAAACCATCAATTCTCCATTTTCAGCTTCGGAATTTGTTGTGGGCAACTTCAAAGCATGATGTGTATTTCATGTCGAATTCCACAGTAGGCCACTGGTCATCATTGTCTCACAAAATGTCAGATGTTCTTGATTTCTCAGGGCATGTTGCTCCAGCGCAGAAACATCCTGGCAGTTTACTAGAAGGGTTTACTGGTGTTCAAGTTAGCACACTTGCAGTGAATGAGGGTTTATTGGTTGCTGGTGGTTTTCAAGGAGAACTAATTTGCAAGAGTTTAGGAGACCGTGATG

>Isogroup_545 AGCGTGGTCGCGGCCGAGGTACTTGCTGCAATGCACCATCTGGGTCAACAAGAGCTCGAACAACTGGGCTTAGAGCAAGAGCAACTGCATTCCTTGCTTTATCATAAACCTTGAGTTGGTTAGTTCGTAAAAATGCTTGGATACCATGTAATGGAGCTCCGACAGGGAACCGAAAATCAAGTTCACCAACGACGCCCTCC

>Isogroup_546 AGCGTGGTCGCGGCCGAGGTCAAAAAGCAGGTGCCAAGAAAGTCTTGATAGTTGACTGGGATGTGCACCATGGAAACGGCACACAGGAGATATTTGAAGGGGACAAATCAGTCCTATATGTATCATTACATCGCCATGAGGATGGAAGCTTCTACCCTGGAACTGGAGCAGTGCACGAGGTGGGGTTTGGTAGTGGCGAAGGATTCTCAGTCAATATACCTTGGAGCTGTGGAGGTGTAGGAGACAACGACTACATATTTGCTTTTCAGCATGTGGTGCTTCCGATTGCTGCAGAATTTGCCCCAGACATCACCATTATATCTGCAGGGTTTGATGCAGCAAGGGGTGATCCTCTCGGTTGTTGTGATGTCACTCCTGCTGGATACTCTCTGATGACGGCCATGTTAACTTCTTGTTCAGAAGGAAAATTATTGGTTATCTTGAGGGAGGATACAATCTTAGGTCTATATCCTCATCGGCTACTGAAGTTG

>Isogroup_547 AGCGTGGTCGCGGCCGAGGTACAGCTGGTTCTTTCAACCGGAGCAAACACTGAACAACTAGTTCTTTTAACAGGCTCCTGTATTCATGGTGGTTGCTACTGATATTCAGGAGTTCCTTCATTGCATCCTCTTTCATTTGATTAACCAAATCATCCTGAGCCTGAAGCACCTTGATGCGAGAAGCATTCAGCTGCATAGAGTACCTG

>Isogroup_548 AGCGTGGTCGCGGCCGAGGTACGCATCAATTTGCCTGTTTGATTGGATATGGAGCCAGTGCTGTATGCCCATATCTGGCATTAGAAACATGCCGACAATGGAGGCTGAGCAACAAAACTGTTAATTTGATGCGCAACGGCAAGATGCCCACAGTGACCATCGAGCAGGCTCAAAGAAACTTTATCAAGGCTGTAAAATCTGGCCTGCTCAAGATACTCTCAAAAATGGGGATATCATTGCTCTCAAGTTACTGTGGAGCTCAGATCTTTGAAATATATGGTCTTGGCCAAGAAGTTGTCGACCTTGCATTCTGTGGAAGTGTATCGAAAATTGGAGGACTCACCCTTGATGAGCTGGGCCGGGAAACATTATCATTCTGGGTGAAGGCATTCTCGGAAGATACTGCGAAGAGGCTGGAGAACTTTGGATTCATCCAGTCCAGACCTGGAGGTGAATTTCATGCAAATAATCCTGAGATGTCAAAGCTGCTGCACAAAGCAATTCGTGAAAAAAA

>Isogroup_549 AGCGTGGTCGCGGCCGAGGTACAGGCTGATCAGGAACGGTCTTGCAACAAAGGTAGAGAAGTCACGTAAGCAGATCAAGGAGAGGAAGAACAGGACCAAGAAGATCAGTGGAGTGAAGAAGACCAAGGCTGGGGAAGCTGCGAAGAAGTGAGGAAAGATAGTTACTTACCTTGTCCGCCTCCAGTTTCTATCTGTTCACTGTCTATTTTTGTACCTGCCCGGGCGG

>Isogroup_550 AGCGTGGTCGCGGCCGAGGTACAAGATTTTGTCAGAGAGAGAAAAGGACGGATATGGAGGGTGAACAACTGGGGACTGCGTAGACTTGCTTACAAGATAAAGAAAGCAACCCATGCCAACTACGTCCTAATGAACTTTGAGATACAGGCAAAATGCATCAACGATTTCAAGACCCTGCTTGATAAGGATGAAAGAATCATTAGACACCTTGTTATGAAACGAGATGAGGCAATTACGGAAGACTGCCCTCCCNCCAC

>Isogroup_**551** AGCGTGGTCGCGGCCGAGGTACTGCTCCTTTGTTCCTTCAAGGAACAACTCCAGCAGCATGTGCGCGTTCACGCCATGGAAGCAGTCATGGCTTGCTGGGAGCTCGAGCAAACTTTGCAGAGTCTTACAGGGGCATCTCCTGGTGAAGGCACCGGGGCAACTATGTCTGACGATGAAGACAATCCGGTCGACAGTGAGAGCAACATGTATGATGGAAATGACGTATCAGATGGTATGGGATTTGGAATGCTAACCGAGGGTGAGAGATCCTTAGTTGAGCGTGTAAGGCAAGAGCTGAAGCATGAGCTTAAACAGGGGGTACCTGCCCGGGCGGCCGCTCGATAGCGTGGTCGCGGCCGAGGTACCACACGAGACGCACGGAGCGTTCTCCGTTACCTACACAAGTTGCAAACCGAGGATCTCTCACTATGCCACAGTATGATTCCTCTTGGTTCTTGCACCATGAAACTAAATGCTACGGTCGAGATGATGCCTGTCACCGATCCCAAGTTTGCCAACATGCACCCGTTT

>Isogroup_552 AGCGTGGTCGCGGCCGAGGTACATCAAGTCAAATAGGAACACCGGGAAAGAAAACCCAGTTTGTATGATTTGTCATCCTTTCCGGCTATTTAAGATTTCTGCTGAAGTCAGGGAGATCAATGACTTTGACTCTTGATATGTCGCTTGCTTCGTCCCTCTCTTTTGGTGTGTTGTATCCCCAATTCACTGCAAAAAACTGCGTCCGAAACACTCAGCTAGCTAGATAATGTACCTG

>Isogroup_**553** AGCGTGGTCGCGGCCGAGGTATCACCCACCAAGCTCCCCTCTTCTACCCACTGGAGTCCCTCGAGAATAATCAACTAGGCCGACGTGAGGAGTTCTACCCTCGCCGACGGCCGTCGCTGAGTCGCCGGTCTGTTCCTTGCTTCTTTCCCTCCTCGGCTCATTTCCTTCCGCCGGCAGCTTGGGTCGTGTGGGCGAGCATGAGCGGCATGTCTCTTGATCTCCTCTCCTGTAGATTCTTGTTCTCTTCGTGCGCTGCAAAGAAGCCCTCGAGCAGGACAAGGCTCACGTTTCTTGCTGCTGCTCCTTCGTCTCCGTCAACACCGTCGCGGTCACCGGGTAACTCATCTCCCAGGCAAAGGCCTGTGCGAGGTGTGCCATTCGTTCCCAG

>Isogroup_554 AGCGTGGTCGCGGCCGAGGTACGAGTTTGGCCACACTTTGCATGCCAACCACCTCCCACGTGGTGATGCCGCAGTCGGGAGGCCATTGGTTAGGCTGTTCAAGCCATTTGATGAGCTCGTTGTTGACAGCAAGGATTTTGATGTTTCTGCTTTGGAGAGCTTCATTGAAGCTAGCAGCA

>Isogroup_555 CGAGCGGCCGCCCGGGCAGGTACATCAGGAATCTTGATCCAATGAGGGATGCTACAATGCTGCGGAGAGAACTGCCCATGATACGCGAGGCTTGCCTCCGAGTCCTTATCCTGTGCACAATCTTCCTCAAGGAGGCTGCAACTTCTGGGCTTTGCTTGGCAGAGATAGGAGAAATGATGACCAGGGAGTTCAGAGGGATGGAGGAGGAGCCAAGCAAACTGGAGGTTGTCTGCATGGAAGCTAGGAAGAAGGTAGCCGAATGGGAACCGTTCTCCCCCGGGGTTGAACAAGGGGAGGACATAGATTTCCAGTTCTCGATGGATGTCTTAGGAGGGTACCTCGGCCGCGACCACGCTATGG

>Isogroup_**556** CGAGCGGCCGCCCGGGCAGGTACTTCAACGATGCCCAGAGGCAGGCGACCAAGGATGCTGGTGTCATTGCTGGCCTGAATGTTGCTAGGATCATCAACGAGCCAACTGCTGCTGCTATTGCCTATGGTCTGGACAAGAAGGGCAGTGAGAAGAACATCCTTGTCTTTGACCTGGGTGGTGGTACCTCGGCCGCGACCACGCT
[truncated: 190,886 more chars]
